# Chromosome‐level assembly, genetic and physical mapping of *Phalaenopsis aphrodite* genome provides new insights into species adaptation and resources for orchid breeding

**DOI:** 10.1111/pbi.12936

**Published:** 2018-05-23

**Authors:** Ya‐Ting Chao, Wan‐Chieh Chen, Chun‐Yi Chen, Hsiu‐Yin Ho, Chih‐Hsin Yeh, Yi‐Tzu Kuo, Chun‐Lin Su, Shao‐Hua Yen, Hao‐Yen Hsueh, Jen‐Hau Yeh, Hui‐Lan Hsu, Yi‐Hui Tsai, Tzu‐Yen Kuo, Song‐Bin Chang, Kai‐Yi Chen, Ming‐Che Shih

**Affiliations:** ^1^ Agricultural Biotechnology Research Center Academia Sinica Taipei Taiwan; ^2^ Department of Agronomy National Taiwan University Taipei Taiwan; ^3^ Taoyuan District Agricultural Research and Extension Station Council of Agriculture, Executive Yuan Taoyuan Taiwan; ^4^ Department of Life Sciences National Cheng Kung University Tainan Taiwan

**Keywords:** plant genome, orchid, *de novo* assembly, genetic mapping, restriction site‐associated DNA sequencing, fluorescence *in situ* hybridization

## Abstract

The Orchidaceae is a diverse and ecologically important plant family. Approximately 69% of all orchid species are epiphytes, which provide diverse microhabitats for many small animals and fungi in the canopy of tropical rainforests. Moreover, many orchids are of economic importance as food flavourings or ornamental plants. *Phalaenopsis aphrodite*, an epiphytic orchid, is a major breeding parent of many commercial orchid hybrids. We provide a high‐quality chromosome‐scale assembly of the *P. aphrodite* genome. The total length of all scaffolds is 1025.1 Mb, with N50 scaffold size of 19.7 Mb. A total of 28 902 protein‐coding genes were identified. We constructed an orchid genetic linkage map, and then anchored and ordered the genomic scaffolds along the linkage groups. We also established a high‐resolution pachytene karyotype of *P. aphrodite* and completed the assignment of linkage groups to the 19 chromosomes using fluorescence *in situ* hybridization. We identified an expansion in the epiphytic orchid lineage of FRS5‐like subclade associated with adaptations to the life in the canopy. Phylogenetic analysis further provides new insights into the orchid lineage‐specific duplications of MADS‐box genes, which might have contributed to the variation in labellum and pollinium morphology and its accessory structure. To our knowledge, this is the first orchid genome to be integrated with a SNP‐based genetic linkage map and validated by physical mapping. The genome and genetic map not only offer unprecedented resources for increasing breeding efficiency in horticultural orchids but also provide an important foundation for future studies in adaptation genomics of epiphytes.

## Introduction

Orchidaceae, the orchid family, belongs to the monocot class of angiosperms and is the most diverse family of flowering plants with more than 26 000 species found in nearly all terrestrial habitats except true deserts. Orchids display many unique features that are not found in other plants, including flowers with pollinia, a column (fused pistil and stamens) and a lip (labellum), and a high level of pollinator specialization, and orchid mycorrhizae. Epiphytic orchids, which represent about 69% of all species in Orchidaceae (Zotz, 2013), are important to rainforest ecosystem because they provide unique microhabitats and contribute to hydrologic and nutrient cycling in the canopy. Orchids have been widely admired for their distinctive beauty and have a long history of use in food flavourings and fragrances (pods of *Vanilla planifolia* and aromatic leaves of *Dendrobium salaccense*), beverages (a traditional Turkish beverage Salep is made from the *Orchis* orchids) and herbal medicines (the use of *Dendrobium* orchids, *Gastrodia elata* and *Bletilla striata* as Chinese herbal medicine). Wild orchid populations have been declining rapidly because of over‐collection and habitat loss. Nowadays more and more countries are engaging in orchid breeding and large‐scale commercial production of orchid hybrids.

The genus *Phalaenopsis* belongs to the subfamily Epidendroideae of Orchidaceae, and the commercial hybrids in this genus are among the most traded flower crops. *Phalaenopsis aphrodite* is an epiphytic orchid that grows on trees in open forests of southern Taiwan and the Philippines. *Phalaenopsis aphrodite* produces blooms that last for up to 3 months. It was one of the earliest orchid species to be imported to Europe in the Victorian era. Since then, *P. aphrodite* has contributed to the genetic background of numerous outstanding large‐flowered or white‐flowered hybrids. Even though the orchid family is economically and ecologically important, neither a genetic map nor a high‐quality reference genome is available for any orchid. The draft orchid genome that was previously generated (Cai *et al*., 2015) is highly fragmented, having incorrectly assembled scaffolds (see Appendix S1 for details), and provides neither genome architecture nor chromosome information. These draft genomes are thus unsuitable for genomics‐assisted breeding purposes, and their utility for cross‐genome comparisons is seriously limited. In view of its importance in orchid breeding programs and its relatively small genome (1.2 Gb) in comparison with other orchids, we decided to sequence the genome of *P. aphrodite*. *Phalaenopsis aphrodite* has 19 chromosomes that are small in size and have similar morphology at the metaphase stage, and the genome contains a high proportion of repetitive sequences. We performed *de novo* assembly of whole‐genome shotgun sequencing data and then anchored and ordered the scaffolds along linkage groups to achieve a chromosome‐level assembly. We constructed a high‐density genetic linkage map and a high‐resolution pachytene karyotype for *P. aphrodite*. We completed the assignment of linkage groups to the 19 chromosomes using fluorescence *in situ* hybridization (FISH) and used FISH to evaluate the accuracy of the genome assembly. This high‐quality reference genome and genetic map of *P. aphrodite* provides new insights into the orchid genome architecture and will serve as a crucial resource for orchid breeding, orchid species conservation and comparative genomic studies.

## Results and discussion

### *De novo* assembly of *Phalaenopsis aphrodite* genome

The nuclear genome of *P. aphrodite* was estimated to be 1.2 Gb based on flow cytometric analysis. Our initial *de novo* assembly obtained 13 732 genomic scaffolds (N50 = 0.95 Mb) with a total length of 1025.1 Mb (for details, see the Supporting text and Tables S1–S4 in the Appendix S1), corresponding to 85% of the *P. aphrodite* genome (Table 1). The largest scaffold was 10.4 Mb, and the 1686 largest scaffolds represented 90% of the assembled genome. After integration with the genetic map (see below), the largest scaffold is 44.5 Mb and the N50 of final scaffolds reached 19.7 Mb. The accuracy of the assembly was validated by bacterial artificial chromosome (BAC) end sequencing, genome survey sequencing and fluorescence *in situ* hybridization (FISH) imaging. Compared with other draft orchid genomes of similar size, our *P. aphrodite* genome assembly is more accurate, complete and highly contiguous (Table 1). BUSCO (Simao *et al*., 2015) assessment indicated that the per cent assembly completeness is ~95% (Figure S1 in Appendix S1).

**Table 1:** Comparison between genome assemblies and protein‐coding gene annotations for three orchid species

	*Phalaenopsis aphrodite*	*Phalaenopsis equestris*	*Dendrobium catenatum*
Estimated genome size (Gb)	1.20	1.60	1.27
Chromosome number	2*n* = 2*x* = 38	2*n* = 2*x* = 38	2*n* = 2*x* = 38
Reference version	This study V1.0	Cai *et al*. (2015) V1.0	Zhang *et al*. (2016) V1.0
*De novo* assembler	ALLPATHS‐LG	SOAPdenovo2	Platanus
Total scaffold length (Mb)	1025.1	1086.2	1008.5
Total number of scaffolds	13 732	236 185	72 901
Scaffold N50 (kb)	946.4	359.1	391.5
Scaffold N90 (kb)	69.4	11.5	15.4
Number of scaffolds ≥ 1 kb	13 485	25 741	36 724
Total bases (Mb, % of genome) in ≥ 1kb	1024.9 (85%)	1038.3 (65%)	983.3 (77.4%)
Assignment of scaffolds to chromosomes	Yes	No	No
Scaffold arrangement on chromosome	Yes	No	No
Final assembly max scaffold (kb)	44493.8 (Chr.2)	NA	NA
Final assembly scaffold N50 (kb)	19680.0	NA	NA
Number of protein‐coding genes	28902	29431	28910
Total length of protein‐coding genes (Mb)	333.45	283.85	294.66
Total length of coding sequence (Mb)	31.19	26.30	28.98
Average exon length (bp)	283	228	243
Average number of exons per gene	4.82	3.93	4.13
Average intron length	2665	2922	2575

### Linkage map for scaffold anchoring and ordering

**Figure 1:**
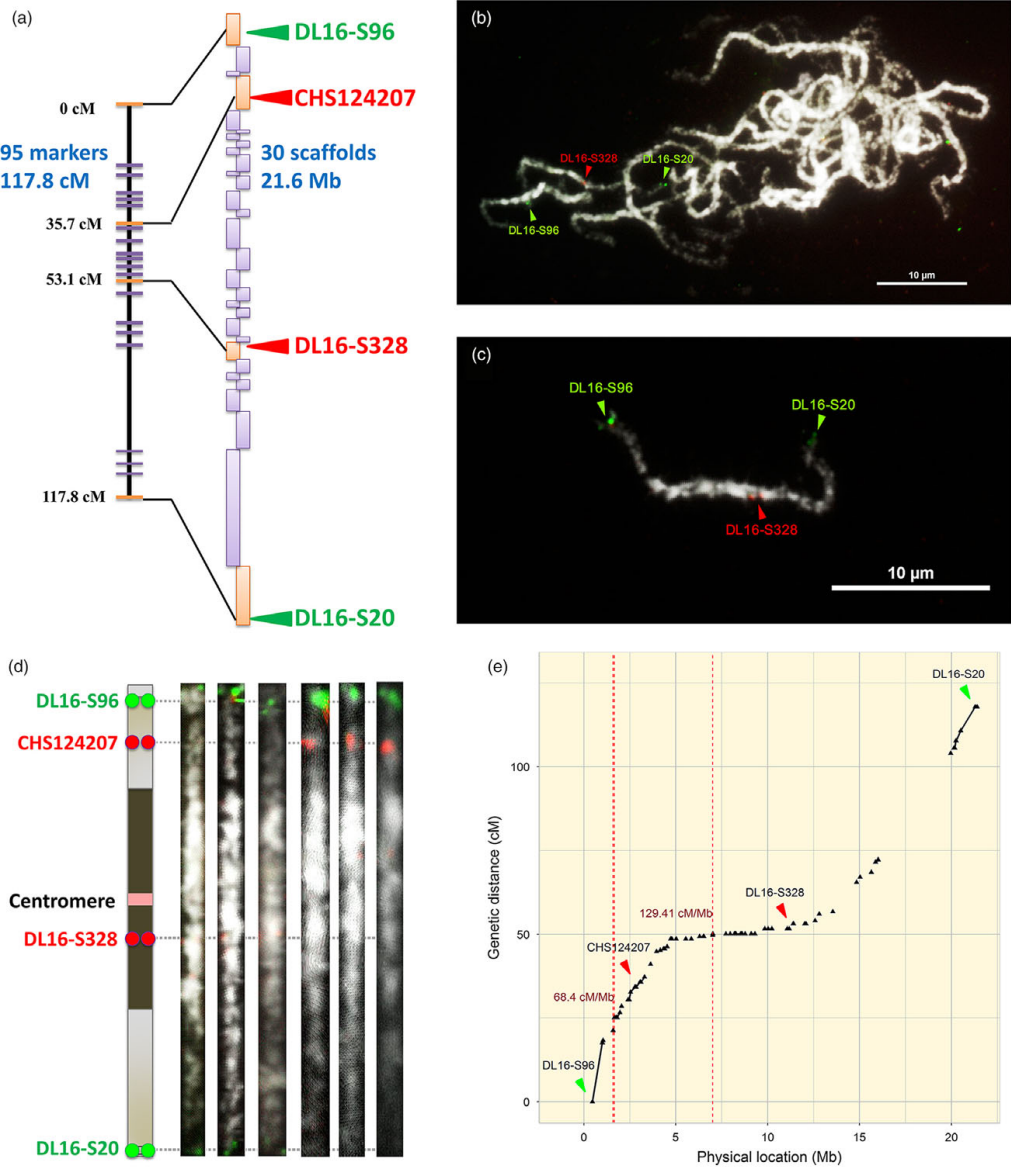
The integration of genetic map, genome assembly and FISH mapping. (a) Linkage group 19 with 95 RAD markers spanning 117.8 cM (not all markers are shown on the map). A total of 30 scaffolds were anchored and oriented within the linkage groups. Both purple and orange rectangles represent the anchored scaffolds. Triangles indicate the physical positions of the linkage group 19 specific FISH probes. (b), (c) FISH mapping reveals where DL16‐S96, DL16‐S328 and DL16‐S20 are located on the same chromosome. The chromosomes were stained with DAPI, and images were converted to black and white. Scale bar = 10 μm. (d) An ideogram of straightened pachytene chromosomes with FISH signals corresponding to DL16‐S96, CHS124207, DL16‐S328 and DL16‐S20. Blue and light blue blocks represent heterochromatin and euchromatin, respectively. (e) The locations of recombination hotspots and genetic markers (black triangles) on chromosome 19. The cumulative genetic distance from the end of the short arm to the end of long arm is shown. The locations of the FISH markers are indicated with triangles. The recombination hotspots (>55 cM/Mb, 10 times higher than the average) are indicated with red dashed lines, and the corresponding recombination rates (cM/Mb).

A dense genetic map can be used to anchor whole‐genome shotgun assemblies and create chromosome‐scale pseudomolecules (Figure 1a). However, the genetic mapping of orchid genomes has greatly lagged behind other horticultural species. We built a high‐density linkage map for orchids using restriction site‐associated DNA sequencing (RAD‐Seq) genotyping data for the parents and 184 progeny of a cross between *P. aphrodite* and *P. modesta*. The resulting map is composed of 2905 RAD markers that were organized into 22 linkage groups covering at least 3075.8 cM (see genetic map in Appendix S2), with an average distance of 1 cM between markers on the linkage map of *P. aphrodite*. A total of 522 scaffolds, with an average scaffold length of 1.1 Mb, were anchored to the linkage groups and oriented by RAD markers. Chromosomes 5, 8 and 10 were each resolved into two linkage groups. The number of RAD markers assigned to each linkage group ranged from 5 markers (linkage group 5a) to 263 markers (linkage group 2).

### FISH mapping of high‐resolution pachytene chromosomes

Because of their small, even size and uniform morphology, orchid chromosomes cannot be distinguished by karyotyping at mitotic metaphase. Therefore, we established a karyotype of *P. aphrodite* based on high‐resolution imaging of 4′, 6‐diamidino‐2‐phenylindole (DAPI)‐stained meiotic pachytene chromosomes from pollinia (Figure 1b–d). High‐resolution FISH mapping of meiotic pachytene spreads revealed a clear distribution of heterochromatic and euchromatic regions, which made it possible to discriminate between the 19 chromosomes by comparing centromere positions and chromatin size. The pachytene chromosomes range from 17.0 to 40.6 μm in length, and all chromosomes are metacentric or submetacentric, with heterochromatin distributed around the centromere (Figure 2 and Figures S2–20 in Appendix S1).

**Figure 2:**
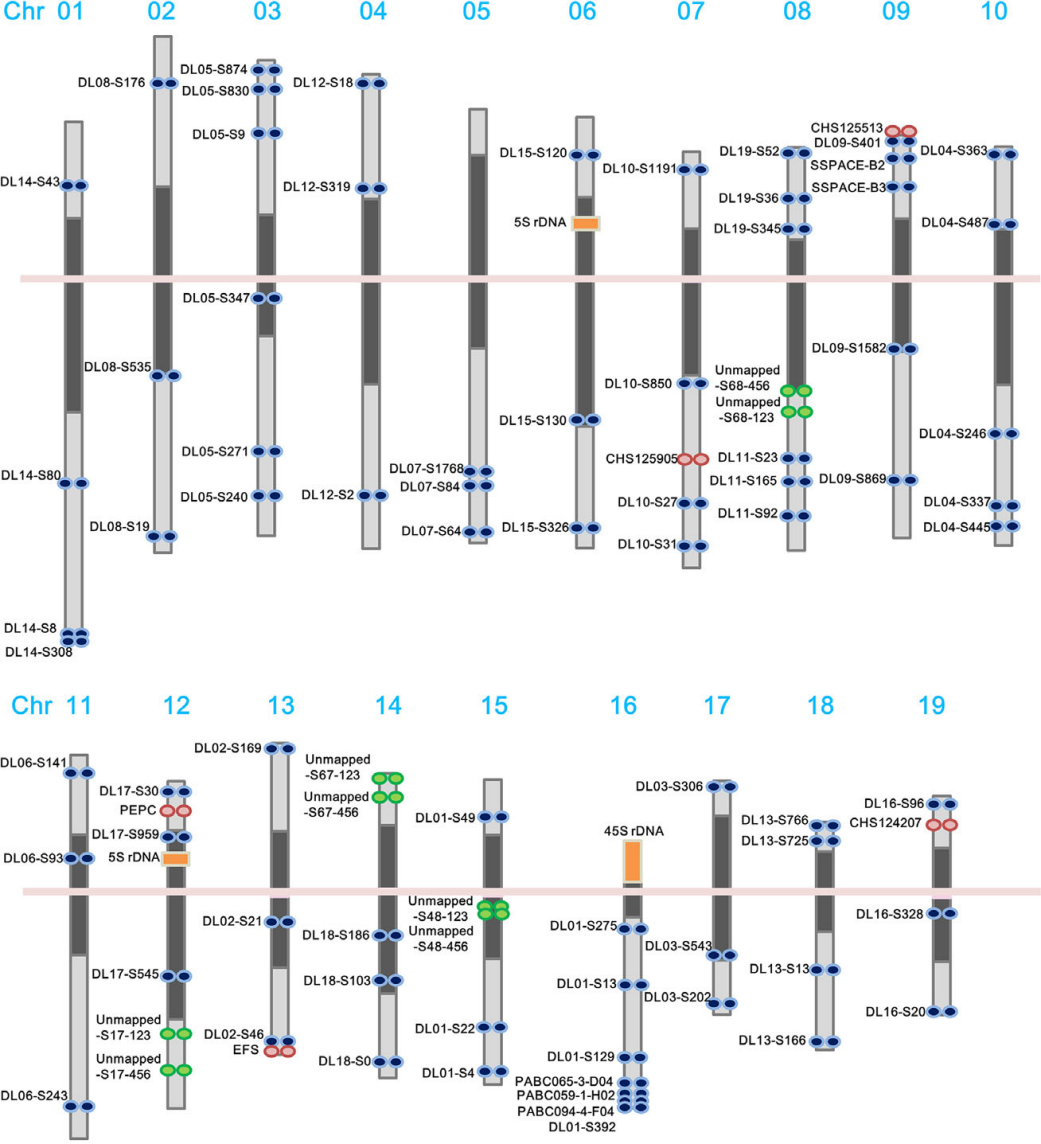
Chromosomal distribution of linkage group‐specific FISH probes. Ideogram showing the location of FISH signals and 90 genetically mapped and unmapped DNA markers on a high‐resolution map of pachytene chromosomes. Dark grey blocks indicate heterochromatin, light grey blocks indicate euchromatin, and the pink line indicates the centromere.

FISH was applied to integrate the genetic and genomic data sets with cytogenetic information. We designed primers to construct three to six sets of linkage group‐specific genomic clones for each linkage group and subsequently localized the clones on *P. aphrodite* pachytene chromosomes by FISH mapping. All linkage group‐specific FISH markers were detected on only one distinct chromosome (Figures 1 and 2, Tables S5 and S6 in Appendix S3, and Figures S2–S20 in Appendix S1); therefore, these markers can be used to assign each linkage group to a *P. aphrodite* chromosome and orient linkage groups on the chromosome arms. At least 90 DNA markers, including genes, BAC clones and unmapped scaffolds, were assigned to all 19 pachytene complements (Figure 2). The relative chromosomal order of all mapped linkage group‐specific markers was consistent with the order on the genetic linkage and physical maps. FISH mapping identified the localization of five unmapped scaffolds that have a total length of 12.9 Mb. A few BAC clones were used as FISH probes to further validate the within‐scaffold arrangement and orientation. Through linkage analysis and FISH mapping, a total of 528 genomic scaffolds were assigned to chromosomes, accounting for 57% of the assembled genome. We have used some of these FISH markers to investigate the chromosome colinearity between *P. aphrodite* and *P. equestris*. The conservation of marker order and localization displays a high degree of chromosomal collinearity and indicates the absence of large‐scale chromosomal rearrangements between these two species. FISH analyses also revealed potential misassemble errors of *P. equestris* draft genome (see Supporting Text and Figure S21 in Appendix S1).

### Recombination hotspots

Recombination frequency varies greatly depending on genomic location and consequently influences the probability of gene introgression during hybridization (Chang *et al*., 2007). We calculated the recombination rates along the 19 assembled chromosomes and found that the whole‐chromosome average recombination rate is 5.5 cM/Mb, which is about 20% higher than the average rate in *Arabidopsis thaliana* (4.6 cM/Mb) (Mezard, 2006). We also identified 74 recombination hotspots distributed across the 19 chromosomes with recombination rates greater than 55 cM/Mb (10‐fold higher than the average; Figure 1e, Figure S23 in Appendix S1, Table S7 in Appendix S3). Among the 74 hotspots, the highest recombination rate is 3478.26 cM/Mb (63‐fold higher than the average) for a site located on chromosome 2. There are 60 (about 81%) recombination hotspots located in regions that have gene density greater than three genes per 100 kb (the whole genome average gene density is 2.82 genes per 100 kb). In total, there are 135 genes located within the regions of recombination hotspots (Table S8 in Appendix S3). On the other hand, 87% of the recombination coldspots, which have recombination rate of 0 cM/Mb over large segments (> 1 Mb) of the genome, have gene density below the whole genome average gene density (Table S9 in Appendix S3). The genes within the recombination hotspots and coldspots are listed in Tables S8 and S10, respectively. Commercial orchids are developed through interspecific (between species) or intergeneric (between genera) hybridization. DNA segments that are located in regions with higher recombination frequencies have a higher probability of being exchanged between homologous or homoeologous chromosomes. The identification of recombination hotspots and coldspots along the 19 chromosomes can aid in predictions of the likelihood of obtaining offspring with the desired genes through meiotic recombination.

### Genome content and structural annotation of the *P. aphrodite* genome

Based on *de novo* structure prediction and subsequent homology searches, we determined that at least 60.3% of the genome assembly consisted of repetitive elements. The majority of transposable elements (TEs) in the *P. aphrodite* genome were Gypsy‐like elements making up 69.9% of the repetitive sequence, followed by Copia‐like elements (10.6%).

**Figure 3:**
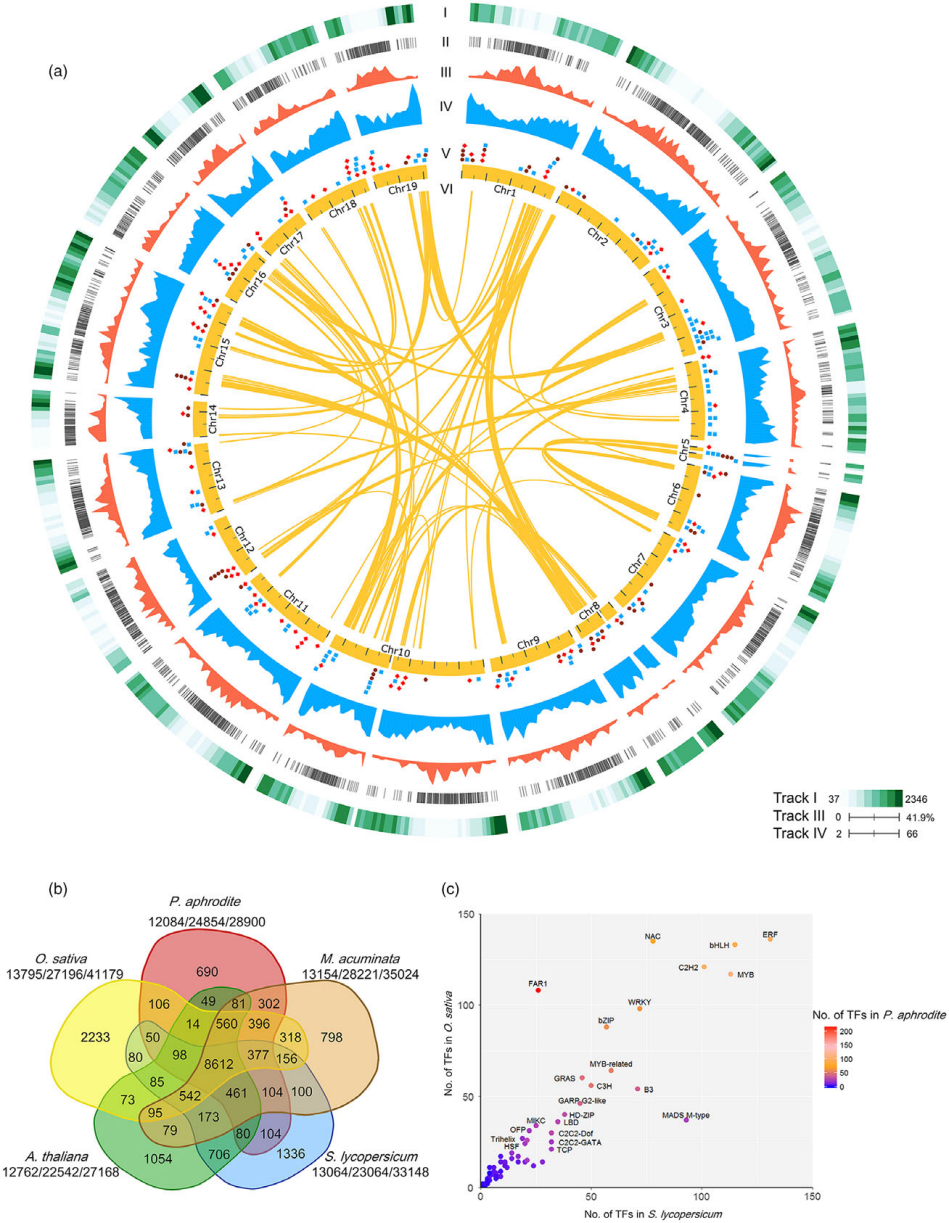
Genomic composition of *P. aphrodite*. (a) Genomic features of 19 *P. aphrodite* chromosomes. Track I: density of ESTs (No. of EST/Mb) from 17 orchid species belonging to 12 genera in five subfamilies of Orchidaceae are plotted for nonoverlapping 1‐Mb windows. Track II: chromosomal distribution of noncoding RNA. Track III: base coverage of TE‐related gene models and pseudogenes for nonoverlapping 1‐Mb windows. Track IV: gene density (no. of protein‐coding gene/Mb) for nonoverlapping 1‐Mb windows. Track V: positions of genes associated with important floral traits. Blue dots indicate genes involved in flower pigment biosynthesis and floral scent. Brown dots indicate genes related to flower development. Red dots indicate genes that control flowering. Track VI: yellow lines indicate chromosome regions that contain at least eight paralogous genes. (b) Gene families unique to *P. aphrodite* and those shared with four other species. The digits under each species name indicate the number of gene families, the number of genes per family and the total gene number (separated by slashes). (c) Comparison of the size of transcription factor families in *P. aphrodite*, rice and tomato. The colour of each dot indicates the number of genes in each family in *P. aphrodite*. Families with more than 20 members in *P. aphrodite* are labelled.

We have built a web‐based transcriptome database for the orchid family, Orchidstra 2.0 (Chao *et al*., 2017), to facilitate *P. aphrodite* genome annotation. Orchidstra 2.0 includes transcriptome data from various tissues of 18 orchid species belonging to 12 genera in five subfamilies of the Orchidaceae. The cross‐species transcript alignments were also used to build gene models. We were able to map 91.9% of the ESTs from the other 17 orchid species to the *P. aphrodite* genome assembly (E‐value cut‐off 1E‐10) (Figure 3a and Table S11 in Appendix S1). A total of 28 902 non‐TE protein‐coding genes were annotated in the *P. aphrodite* genome (Figure 3a). Of these, 90.3% included both start and stop codons. Of the annotated non‐TE protein‐coding genes, 23 188 are supported by both mRNA evidence and protein homology.

The orchid genome has a gene density of 2.82 genes per 100 kb, which is 30% lower than that of the tomato genome (~4 genes per 100 kb, calculated using data from ITAG3.10), an average gene length (including introns) of 11 538 bp and an average coding sequence length of 1079 bp. The GC content is 34.4% and 44.3% for the whole genome and coding regions, respectively. The better accuracy and completeness of the *P. aphrodite* genome sequence and annotation are reflected in the larger coding sequences with greater exon length and higher mean number of exons per gene, which is ~20% higher than previous studies (Table 1). We functionally annotated 28 753 (99.5%) protein‐coding genes (Table S12 in Appendix S1), including 1615 transcription factors and 1185 transporter genes (Tables S13 and S14 in Appendix S3). Moreover, we predicted an additional 10 199 TE‐related loci (Figure 3a) and identified different types of noncoding RNA genes, including transfer RNA, microRNA, snoRNA and snRNA (Figure 3a and Table S15 in Appendix S1).

### Gene family analysis

#### Gene families associated with adaptations to life in a rainforest canopy

**Figure 4:**
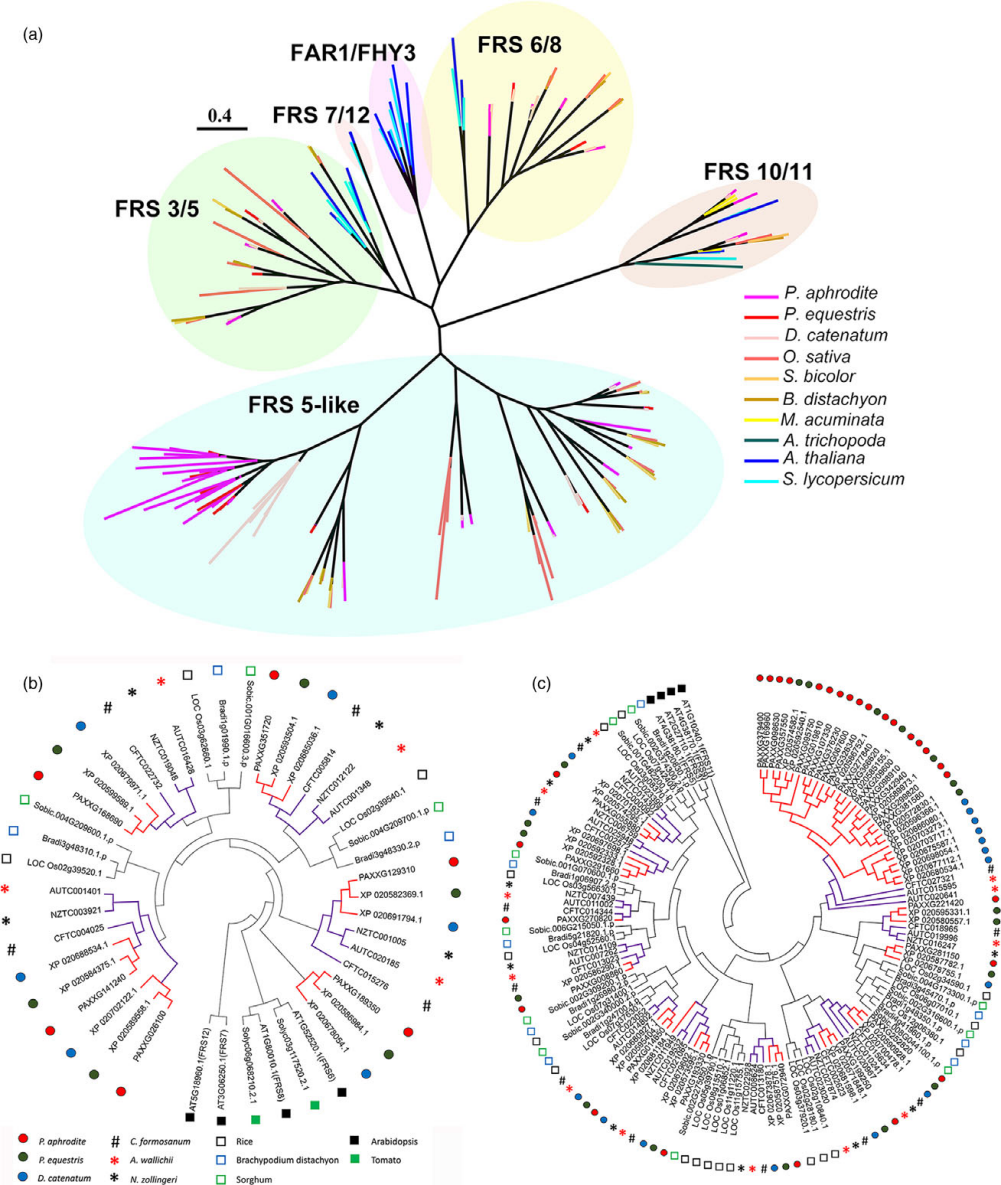
Orchid lineage‐specific duplication of the *FAR1*/*FRS* gene family. (a) Maximum‐likelihood phylogenetic tree of 202 full‐length FAR1/FRS proteins (sequences with length > 400 aa) from 10 species. (b) Phylogeny of the FRS6 subfamily, including 45 amino acid sequences from 11 species. *Arabidopsis* FRS7 and FRS12 were used as outgroups. (c) Phylogeny of the FRS5‐like subfamily, including 134 amino acid sequences from 10 species. *Arabidopsis* FRS11 was used as outgroup. In (b) and (c), the purple branches indicate the orchid clade, and red branches indicate the clade of epiphytic orchids.

Gene family analysis and clustering of protein‐coding genes were conducted for *P. aphrodite* and nine other sequenced plant species (Table S16 in Appendix S1). By comparing *P. aphrodite*, rice, banana, tomato and *Arabidopsis*, we identified a total of 19 912 orthologous groups (each with at least two members; Figure 3b), with 8612 groups being shared among the five species. Of the 28 902 protein‐coding genes in *P. aphrodite*, 24 854 were clustered into 12 084 groups, with 690 groups being specific to *P. aphrodite* (Figure 3b). In the *P. aphrodite* genome, the FAR1 (far‐red‐impaired response 1)/FRS (FAR1‐related sequence) is the largest family of transcription factors with 216 loci (Figure 3c), which is much higher than the number of *FAR1*/*FRS* genes in other flowering plants with whole genome sequences. For example, there are 8.3 times more *FAR1*/*FRS* genes in *P. aphrodite* than in tomato, and two times as many as in rice. Epiphytic *Phalaenopsis* orchids are very shade‐tolerant and adapt well to being grown indoors. In their natural habitat, epiphytic orchids grow on tree trunks and are shaded or semi‐shaded by the canopy. The ratio of red to far‐red (FR) light can be very low because the majority of blue/red light is absorbed by the leaves of the canopy before reaching the orchids, whereas FR light can penetrate the canopy. Phytochrome A (*PHYA*) is known to be crucial for de‐etiolation in an environment dominated by FR light. *PHYA* mediates FR high irradiance response (HIR) enabling plants to develop under shaded conditions (Casal *et al*., 2014). *FAR1* and *FHY3* (far‐red elongated hypocotyls 3) are transposase‐derived transcription factors that regulate *PHYA*‐mediated FR light‐sensing (Lin *et al*., 2007). In *A. thaliana,* the *FRS* genes are known to be involved in a light signalling pathway controlling flowering time and development (Lin and Wang, 2004). The FAR1/FRS family is composed of six major clades (Figure 4a), including two clades (FAR1/FHY3, FRS7/12) with dicot members only, three clades (FRS6/8, FRS3/5/9, FRS10/11) with both dicot and monocot members, and one monocot‐only FRS5‐like clade (Figure 4a). We further incorporated *FAR1*/*FRS* genes from the terrestrial lady slipper orchid (*Cypripedium formosanum*, subfamily Cypripedioideae), *Apostasia wallichii* and *Neuwiedia zollingeri* (both in the subfamily Apostasioideae) in phylogenetic analysis of each major clade and revealed orchid lineage‐specific duplication events in the FRS 6/8 clade (Figure 4b). Moreover, we found an expanded subclade composed of 17 *P. aphrodite* genes and 14 genes from *P. equestris*/*D. catenatum* in the FRS5‐like clade (Figure 4c). Our results suggest that in *P. aphrodite*, and very likely all epidendroid orchids, most of which are tropical epiphytes, there have been lineage‐specific expansions of *FRS5*‐like genes (Figure 4c). The orchid *FRS10*,* FRS11*,* FRS3* and *FRS6* genes are widely expressed in both reproductive and vegetative tissues but, with the exception of *FRS11*, are not highly expressed in the pollinia (Figure S24 in Appendix S1). In contrast, the expanded FRS5‐like subclade contains genes expressed at lower levels but exhibiting more tissue‐specific expression patterns, especially in flower buds. The retention of lineage‐specific expansion of the FRS5‐like subclade might be a result of selective pressure due to the very unstable light quality that is associated with the epiphytic lifestyle to increase the number of transcription factors/transcriptional activators involved in the regulation of light‐sensing systems or flowering time. It was reported that gene duplication is related to higher expression diversity in *Arabidopsis* (Ha *et al*., 2009). Previous study also showed that duplicate genes could contribute to tissue‐specific transcriptomes that are important to the evolution of phenotype (Guschanski *et al*., 2017). We observed the tissue‐specific expression of the expanded FRS5‐like subclade in *P. aphrodite*, suggesting these genes may play roles in the adaptations to unstable light condition in a rainforest canopy.

#### Orchid lineage‐specific duplications of floral‐organ identity genes involved in lip and pollinium development

The *P. aphrodite* genome has a total of 56 annotated MADS‐box genes, which is slightly higher than in *P. equestris* (Cai *et al*., 2015) and *D. catenatum* (Zhang *et al*., 2016) (Table S17 in Appendix S1). However, the difference in number is partly due to the fragmented assembly and incomplete gene annotations of *P. equestris* and *D. catenatum*. For example, we identified the orchid M‐beta type and P‐class MIKC* MADS‐box transcription factors that were proposed to be absent from the orchid genome (Zhang *et al*., 2017).

**Figure 5:**
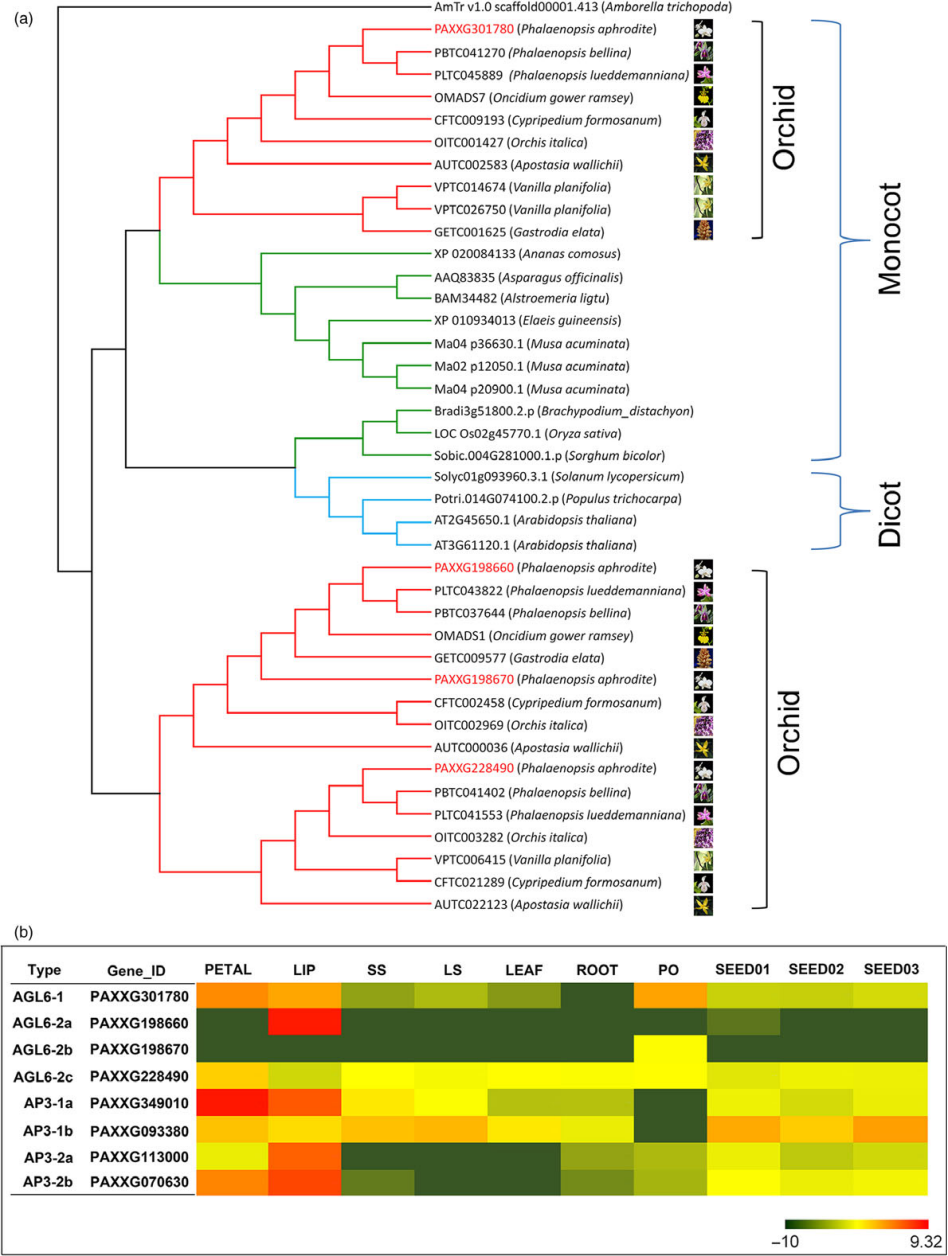
Phylogenetic tree of *AGL6* and expression patterns of *P. aphrodite AGL6* and *AP3*. (a) Red clades indicate orchid family members, and green and blue clades indicate proteins from other monocots and dicots, respectively. The phylogenetic tree was generated from 41 amino acid sequences from 21 species. The nine orchid species belong to five Orchidaceae subfamilies: *P. aphrodite*,* P. bellina*,* P. lueddemanniana*,* Gastrodia elata* and *Oncidium gower ramsey* (subfamily Epidendroideae), *Apostasia wallichii* (subfamily Apostasioideae), *Cypripedium formosanum* (subfamily Cypripedioideae), *Orchis italica* (subfamily Orchidoideae) and *Vanilla planifolia* (subfamily Vanilloideae). (b) RNA‐Seq‐based gene expression heatmap (TPM on log2 scale) of the *P. aphrodite AGL6* and *AP3* genes. PETAL, the lateral petals; LIP, the labellum; SS, short stalk; LS, long stalk; PO, pollinia. SEED01, protocorm formation; SEED02, protocorm development; SEED03, seedling formation.

Orchids are known for their prodigious diversity of flower morphology, especially the greatly enlarged showy bottom petal (labellum). *P. aphrodite* has four *AP3* genes, and phylogenetic analysis revealed that there are two major AP3 clades, AP3‐1 and AP3‐2. The AP3‐2 clade contains two orchid family‐specific paralogue subclades, including members from all five subfamilies of the Orchidaceae, while the AP3‐1 clade has members from both dicots and monocots (Figure S25 in Appendix S1). The genes of AP3‐2 clade (*PAXXG113000* and *PAXXG070630*) are highly expressed in the lip (Figure 5b), suggesting that the AP3‐2 clade might be closely related to the morphological diversification for developing lip structure. Moreover, a phylogenetic tree of 41 *AGL6* genes from 21 species revealed two major clades (Figure 5a); the AGL6‐1 clade comprises members from all 21 species, and the AGL6‐2 clade contains two orchid‐specific paralogues. Similar to the pattern of duplications observed in the AP3 family, two AGL6‐2 paralogues are found in all five subfamilies of the Orchidaceae. Within the AGL6‐2 clade, both the *OMADS1* (Hsu *et al*., 2015b) from *Oncidium* (Figure 5a) and *PAXXG198660* are lip‐specific genes (Figure 5b). It was reported that lip formation involves competition between two protein complexes containing different AP3/AGL6 homologues, namely AP3‐2/AGL6‐2 vs. AP3‐1/AGL6‐1, the former of which is exclusively required for lip formation (Hsu *et al*., 2015b). This suggests that the duplication of both *AP3‐2* and *AGL6‐2* genes shared by the orchid family led to the development of the unique lip organ.

**Figure 6:**
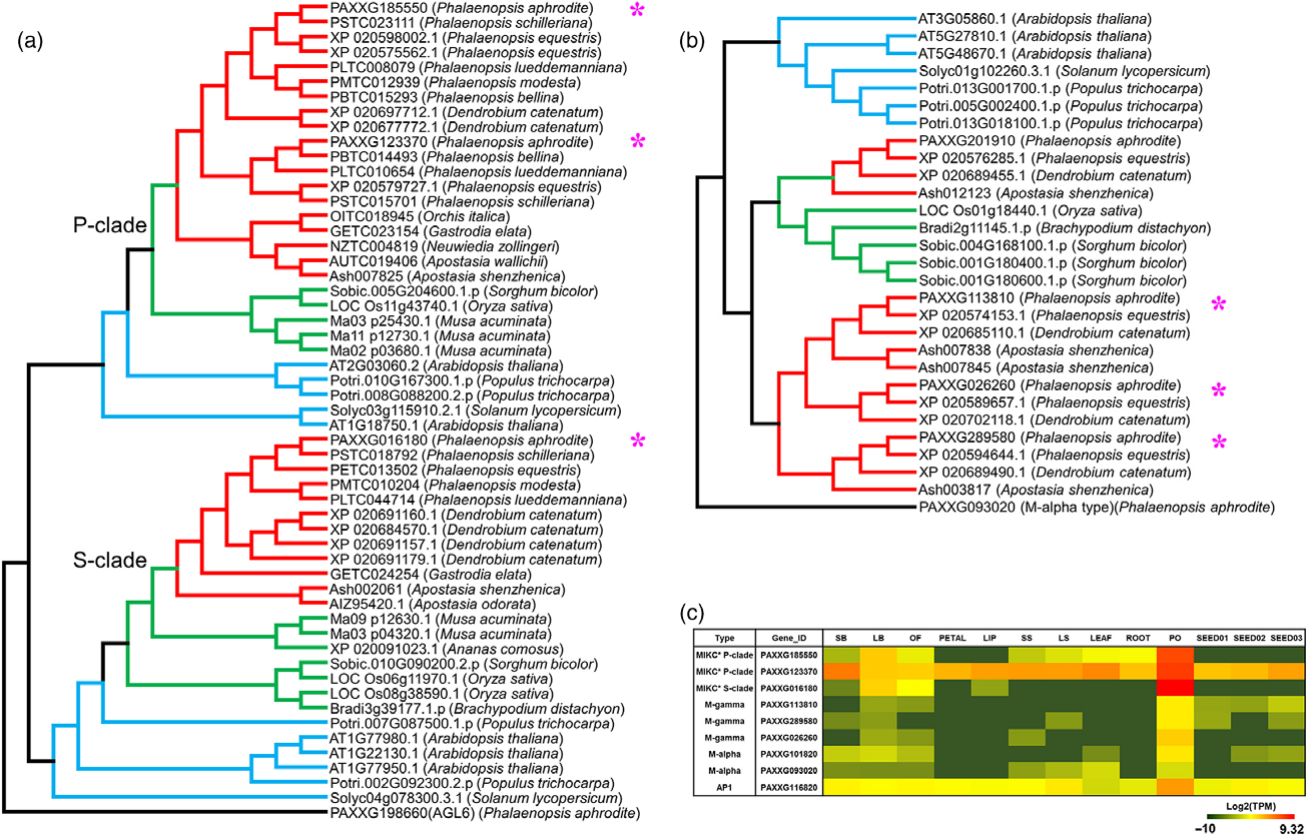
Orchid lineage‐specific duplications of MADS‐box genes involved in pollinium development. (a) Maximum‐likelihood phylogenetic tree of MIKC* type MADS‐box proteins from *P. aphrodite* and 12 other orchid species and eight plants with sequenced genomes. The pollinium‐specific *P. aphrodite* genes are indicated by pink star next to the sequence ID. (b) Maximum‐likelihood phylogenetic tree of M‐gamma‐type MADS‐box proteins from *P. aphrodite* and nine other species. (c) Gene expression heatmap of pollinium‐specific MADS‐box genes in *P. aphrodite*.

One of the distinctive features of epiphytic orchids is the pollinium, which is formed by the fusion of millions of tiny pollen grains with specialized structures that attach to pollinators and ensure the complete removal and efficient transport of pollen grains during floral visitation (Johnson and Edwards, 2000). Based on genome annotation and RNA‐Seq data, we identified nine MADS‐box genes in *P. aphrodite* that are specifically expressed in pollinia (Figure 6). Among these genes, there were two P‐class MIKC*‐type, one S‐class MIKC*‐type, one AP1, two M‐alpha type and three M‐gamma‐type genes. A recent study (Zhang *et al*., 2017) reported that P‐class MIKC* genes are missing from *P. equestris* and *D. catenatum*, and proposed that the absence of P‐class MIKC* genes in all orchids except *A. shenzhenica* is related to the evolution of the pollinium. However, our results do not support their conclusion. In fact, both the epiphytic orchids with true pollinia and the Apostasioideae species with loosely aggregated monads have S‐ and P‐class MIKC* genes (Figure 6a). *P. aphrodite* and other epiphytic orchids (including *P. equestris* and *D. catenatum*) have two P‐class MIKC* genes, while the Apostasioideae has only one P‐class MIKC* gene. Previous studies have shown that S‐ and P‐class MIKC* genes form heterodimers to bind their DNA targets and are required for pollen maturation and tube growth in *Arabidopsis* (Adamczyk and Fernandez, 2009). Our results that showed the existence of both S‐ and P‐class MIKC* genes in the orchid lineage also confirmed that the function of MIKC* heterodimers in pollen development has been conserved across monocots and eudicots (Liu *et al*., 2013). Moreover, phylogenetic analysis showed that one M‐gamma subclade is formed by pollinium‐specific genes that appear to be orchid‐specific (Figure 6b). In summary, all epiphytic orchids have one pollinium‐specific *AP1* gene, one pollinium‐specific *M‐alpha* gene and one pollinium‐specific *M‐gamma* gene, but these genes are absent in the subfamily Apostasioideae. These orchid lineage‐specific duplications of MADS‐box genes might have contributed to the variation in pollinia morphology and its accessory structure in the orchid family.

#### Genes involved in the biosynthesis of flavonoids

Floral traits, including flower architecture, flowering time, and flower colour and scent, are of particular interest to orchid breeders. We manually annotated gene families known to be associated with flowering time regulation, biosynthesis of floral pigments and scent (Tables S18 and S19 in Appendix S1, Table S20 in Appendix S3). Most of the annotated flower trait‐related genes have full‐length models and are located on scaffolds that have been placed within a chromosome. The anthocyanin biosynthetic pathway in orchids is of particular interest because it is associated with important floral traits and phenotypic adaptations. There is no difference in the number of genes encoding flavonoid biosynthesis‐related enzymes between white *P. aphrodite* and pink *P. equestris* (Table S18 in Appendix S1) (*P. equestris* has more partial‐gene models due to its relatively incomplete assembly). We investigated the gene expression patterns of orthologous gene pairs using the RNA‐seq data set (Chao *et al*., 2017) that we obtained from samples of compatible tissue stage of *P. aphrodite* and another pink orchid *P. lueddemanniana*. For genes involved in the first three steps of the anthocyanin biosynthetic pathway, which are common to the synthesis of various compounds such as flavonoids and lignin, the expression patterns are similar in white and pink orchids (Figure S26 in Appendix S1). However, flavanone 3‐hydroxylase (F3H), a key enzyme that acts at the diverging point for different pigment production pathways, has different expression patterns across different tissues in white orchid and pink orchid (Figure S26 in Appendix S1). In *P. aphrodite*, F3H was expressed significantly higher (adjusted *P *< 0.001) in the open flower and large bud than in the leaf and root, while in *P. lueddemanniana,* F3H was expressed significantly higher (adjusted *P* < 0.001) in the flower bud than in the open flower, root and leaf. Tables S21 and S22 in Appendix S3 show the p‐values for DESeq2 Wald test (Love *et al*., 2014) for differential expression. In *P. lueddemanniana,* the expression levels of both dihydroflavonol 4‐reductase (DFR) and anthocyanidin synthase (ANS) were significantly higher (adjusted *P* < 0.001) in the flower bud than in root/leaf (Figure S26 in Appendix S1, Tables S21 and S22 in Appendix S3). Moreover, the orthologs (reciprocal best match with > 95% nucleotide identity) of known R2R3‐MYB regulators of the anthocyanin biosynthetic pathway in hybrid orchids (Hsu *et al*., 2015a) had different expression patterns in *P. aphrodite* and other coloured *Phalaenopsis* orchids. These results suggest that the white petal phenotype of *P. aphrodite* is not caused by the absence of structural or known regulatory genes. The *P. aphrodite* genomic sequence will be a useful resource for studying the regulatory elements controlling orchid flower colour.

## Conclusions

The advancements in sequencing technologies have been greatly spurring draft genomes generation for many nonmodel plant species; however, it remains a challenge to achieve chromosome‐level genome assembly for large and complex plant genomes. In this study, we present the first cytogenetically validated chromosome‐scale orchid genome assembly and the genetic linkage map. The high cross‐species mapping rate of transcripts to the *P. aphrodite* genome indicates the usefulness of this genome for developing markers representing important genetic regions across orchid species. The *P. aphrodite* genome assembly would provide an invaluable resource for identifying the genetic variation underlying ecological traits and towards genomics‐assisted breeding of orchids.

## Experimental procedures

### Plant material and genomic library preparation

Dr. Tsai‐Mu Shen from National Chiayi University, Chiayi City, Taiwan, and Dr. Yao‐Chien Alex Chang from National Taiwan University, Taipei, Taiwan, kindly provided us the mature plants of *P. aphrodite*. The mature plants were propagated vegetatively and grown in a green house facility at Academia Sinica. *P. aphrodite* genomic DNA was isolated from orchid petals according to a previously published protocol with modification (Carlson *et al*., 1991). The preparation of genomic libraries is detailed in Appendix S1.

### Estimating genome size based on flow cytometric data analysis

The extraction of nuclei and the estimation of nuclear DNA content were conducted as previously described (Dolezel *et al*., 2007). Fresh leaf sections (0.5 cm^2^) were finely chopped with a new razor blade in 1 mL cold Tris–MgCl_2_ buffer (200 mm Tris, 4 mm MgCl_2_‐6H_2_O, 0.5% triton X‐100, pH  7.5). After filtering through three 40‐μm cell strainers, the sample was stained with propidium iodide solution (50 μg/mL) containing RNase. All samples were analysed on a MoFlo XDP Cell Sorter (Beckman Coulter, Brea, CA) at the Flow Cytometry Analysis and Cell Sorting Facility at the Institute of Plant and Microbial Biology (IPMB), Academia Sinica, Taiwan. The results of flow cytometry analysis showed four peaks in *P. aphrodite* leaves, corresponding 2C, 4C, 8C and 16C nuclei (Figure S22a in Appendix S1), and this endoreduplication pattern was also observed in *P. equestris* leaves (Lin *et al*., 2001). The 2C peak position of *P. aphrodite* overlapping with the peak of chicken erythrocyte nuclei singlets (Biosure P/N 1013) (2.5 pg/2C) indicates the genome size of *P. aphrodite* approximately equal to the genome size of chicken (Figure S22b in Appendix S1). The 2C value for *P. aphrodite* is estimated to be 2.445 ± 0.199 pg using Rice TN67 (0.82 pg/2C) as an internal reference with three biological repeats (Figure S22c in Appendix S1). The number of base pairs was calculated by multiplying the mass in picograms by 0.978 × 10^9^.

### Construction of an orchid BAC library

DNA isolation and customized BAC library construction were completed by Lucigen (Lucigen, WI). In brief, isolated genomic DNA was randomly sheared, end‐repaired and ligated with adapters. The fragments were then ligated to a NotI‐digested vector, pSMART^®^BAC. The completed *Phalaenopsis aphrodite* BAC library was packed and shipped on dry ice as a glycerol stock in 326 384‐well plates (a total of 125 184 clones).

### *De novo* genome assembly

The assembly of *P. aphrodite* genome is detailed in Appendix S1. Briefly, adapter trimming and quality filtering of genomic DNA reads were performed using Cutadapt for paired‐end reads and NxTrim for mate‐pair reads. *De novo* genome assembly was performed with ALLPATHS‐LG using 52X overlapping read pairs and 54X mate‐pair reads (Table S2 in Appendix S1). Then, the ALLPATHS‐LG scaffolds were linked and extended using SSPACE with several mate‐pair libraries ranging from 8 to 15 kb (a total coverage of 49X) and 40 kb fosmid library (1.5X) (Table S2 in Appendix S1).

### Assessment of the genome assembly

Several full‐length cDNAs with known locations in the assembled genomic scaffolds were used to design PCR primers for the identification of *P. aphrodite* BAC clones containing the cDNA sequences. The screening strategy was as described previously (Hsu *et al*., 2011). After super pool PCR, plate PCR, row PCR and spot PCR, a total of 34 BAC clones were identified, while some of the BAC clones were also found to be clustered together in a fingerprinted contigs map (data not shown). A total of 67 BAC‐end sequences were obtained from the 34 BAC clones, and 14 genome survey sequences were obtained by sequencing BAC clones using primers designed based on the cDNAs. With an E‐value cut‐off of 1E‐10, a total of 63 BAC‐end sequences (94%) were mapped to the genome with over 90% sequence identity and over 90% of their length. Meanwhile, 3% of the BAC‐end sequences were mapped to the genome with 89% sequence identity. Both ends of 26 BACs were mapped within a single scaffold and with the correct relative orientation, which resulted in an estimated average insert size of 91.7 kb of the BAC clones. All genome survey sequences could be aligned with at least 95% sequence identity over the entire query sequence to the genome, and their positions were concordant with respect to the flanking paired BAC‐end sequences in scaffolds, indicating high concordance between the genome assembly and BAC clones. Three BAC clones were used as probes in FISH analysis to demonstrate the accuracy of the scaffolding in the *P. aphrodite* genome assembly (Figure 2).

### Construction of RAD libraries

*PstI*‐digested RAD libraries were prepared following the protocol outlined in Etter *et al*. (2011). Total genomic DNA was isolated from young flower buds using the Plant Mini Kit (Qiagen, Venlo, The Netherlands) following the manufacturer's instructions. One microgram gDNA was digested with 20 units of *PstI*‐HF (New England BioLabs [NEB], Ipswish, MA) overnight in a 50 μL reaction volume. Samples were heat‐inactivated for 20 min at 80 °C. Digested DNA was ligated to 2 μL 100 nm P1 barcoded adapter, which is a modified Solexa adapter, along with 1 μL 10× NEBuffer4 (NEB), 0.5 μL 2000 unit/μL T4 DNA ligase (NEB), and 0.6 μL 100 mm riboATP (Promega, Madison, WI) in a 60 μL reaction volume for 1 h. Samples were heat‐inactivated for 20 min at 65 °C. For one RAD library, 32 or 18 samples (20 μL each) were pooled together. Each pooled sample was divided into several 50 μL aliquots in 0.5‐mL PCR tubes (Axygen catalog # PCR‐05‐C, Corning, Tewksbury, MA). DNA was sheared using a Bioruptor UCD‐200 sonicator (Diagenode, Liège, Belgium) set to high for three runs of 7 min (30 s on/30 s off). The peak size of most DNA aliquots was approximately 300 bp. If the peak of sheared DNA was over 500 bp, then additional sonication was performed until the peak was less than 500 bp. Sheared DNA aliquots were pooled and concentrated using two MinElute columns (Qiagen) and eluted with the addition of 40 μL EB buffer (10 mm Tris–HCl, pH 8.5) to each column. Eluted DNA samples were combined and size selected using Agencourt AMPure XP magnetic beads (Beckman Coulter) with a DNA: beads ratio (v/v) of 1:0.65; this removed DNA fragments less than 300 bp in size. Recovered DNA was resuspended in 20 μL EB buffer and treated using a Quick Blunting Kit (NEB) for end repair. One microlitre of Blunt Enzyme Mix, 2.5 μL 10× Blunting Buffer, and 2.5 μL 1 mm dNTP mix were added to the 20 μL DNA solution. The mixture was incubated at 25 °C for 30 min. Agencourt AMPure XP magnetic beads (Beckman Coulter) were used for reaction clean‐up with a DNA: beads ratio (v/v) of 1:1.8; this removed DNA fragments less than 50 bp in size. The repaired dsDNA was suspended in 20 μL buffer EB and quantified using the Quant‐iT dsDNA HS Assay Kit (Life Technologies, Carlsbad, CA). Adenine was added to the 3′ ends of dsDNA fragments in a 50 μL reaction volume containing 1 μg dsDNAs, 5 μL 10× NEBuffer2, 1 μL 10 mm dATP and 3 μL of 5 unit/μL Klenow fragment (NEB). The sample was mixed and incubated at 37 °C for 30 min. DNA was cleaned using 90 μL (1.8× volume) Agencourt AMPure XP magnetic beads (Beckman Coulter) and resuspended in 45 μL EB buffer. Reactions for P2 adapter ligation were performed by adding 1 μL 10 μm P2 adapter to the dsDNA solution along with 5 μL 10× NEBuffer2, 0.5 μL 100 mm riboATP (Promega, Madison WI), and 0.5 μL 2000 unit/μL T4 DNA ligase (NEB). The mixture was incubated at 20 °C for 3 h. The P2 adapter‐ligated dsDNA was then purified using 35 μL (0.7× volume) Agencourt AMPure XP magnetic beads (Beckman Coulter), resuspended in 20 μL EB buffer and quantified using a Quant‐iT dsDNA HS Assay Kit (Life Technologies). Fifty nanograms of DNA was PCR‐amplified using 4 μL 10 μm modified Solexa primer mix and 50 μL Phusion High‐Fidelity PCR Master Mix (NEB) in a 100 μL reaction volume. The PCR settings were 98 °C for 30 s, followed by 18 cycles of 98 °C for 10 s, 66 °C for 30 s, 72 °C for 30 s and a final extension at 72 °C for 5 min. The PCR‐enriched product was purified with 70 μL (0.7× volume) Agencourt AMPure XP magnetic beads (Beckman Coulter) and diluted to 10 ng/μL. One RAD library was sequenced in one lane of an Illumina Hiseq2000/2500 flow cell (100 bp single‐end reads) (Illumina, San Diego, CA).

### Analysis of RAD sequencing reads and construction of a genetic linkage map

Nine RAD libraries including barcoded DNA from the two parents and 184 F1 progeny were sequenced. The raw sequencing reads were processed using the ‘process_radtags’ command in the Stacks (Catchen *et al*., 2011) software version 1.30. Low‐quality reads (quality score less than 20) were removed, and the RAD sequencing reads for each sample were sorted. The processed RAD reads were 95 bp long after removing barcode sequences. The average RAD read number for the 184 F1 samples was 7 362 090. The RAD read number for the parents *P. aphrodite* and *P. modesta* was 19 689 798 and 18 050 473, respectively. It needs to be noted that two genetic maps corresponding to each of the two parents can be generated from the F1 mapping population. In the current study, only the *P. aphrodite* genetic map was generated and used.

RAD reads for each sample were mapped to scaffolds using BWA (Li and Durbin, 2009) with the parameter of maximum edit distance in seed set to 3. Local realignment of reads around indels was performed using GATK (DePristo *et al*., 2011). The resulting Sequence Alignment/Map (SAM) files defined the positions of RAD reads on the scaffolds. The RAD markers for the *P. aphrodite* genetic map were defined by single nucleotide polymorphism (SNP) sites within the *P. aphrodite* RAD reads which were mapped to the same locations in the scaffolds. An additional criterion for the RAD markers was that the SNP sites had to show no polymorphism within the *P. modesta* RAD reads mapping to the same positions. The chosen RAD markers were then named with prefix ‘UM’ following six digits. RAD marker genotypes for the 184 F1 samples were obtained using the ‘ref_map.pl’ command of the Stacks software with default parameters, except that the ‘CP’ cross‐type was chosen and the minimum read depth was set to 3.

There were two different stages of genetic linkage map construction, each with a different purpose. During the first stage, the maximum‐likelihood mapping algorithm of the JointMap software version 4.1 (Van Ooijen, 2006) was used. This step allowed the simultaneous identification of a large number of closely linked markers, especially under the condition that a substantial number of missing genotypes is typical of RAD‐seq genotype data. The purpose at this stage was to anchor and order the scaffolds and verify the scaffolding. In the second stage, in which the validation of linkage groups with FISH mapping was carried out and the final version of the pseudomolecules was available, a genetic linkage map was constructed to estimate genetic distance. After removing duplicated individuals, which were identified based on their marker genotypes, and individuals with too much missing genotype data, the remaining 136 F1 individuals were used to build the final genetic linkage map. The linkage groups were validated using the R/qtl version 1.40 software (Broman *et al*., 2003) with the criteria recombination fraction < 0.4 and LOD > 10. The genetic distance between flanking markers was estimated using the Mapmaker version 3.0b software (Lander *et al*., 1987) with the Haldane's mapping function.

### The preparation of meiotic pachytene spreads

Chromosome spreads were prepared mainly according to a previously published protocol (Kuo *et al*., 2016). The fixed pollinia (glacial acetic acid: ethanol, 1:3 (v/v)) with chromosomes at the pachytene stage were first rinsed for 5 min each in distilled water and 10 mm citrate buffer (4 mm citric acid and 6 mm tri‐sodium citrate, pH 4.5). The pollinia were cut into small pieces in 10 mm citrate buffer using a grinder in a microcentrifuge tube. The pollinia cell walls were digested in an enzyme mixture in 10 mM citrate buffer containing 1% (w/v) pectinase solution (Sigma), 1% (w/v) pectolyase Y23 (Seishin Pharmaceutical) and 1% (w/v) cellulose Onozuka RS (Yakult Pharmaceutical) at 37 °C for 30 min. The protoplast cells were washed twice with distilled water, and then, the water was replaced with freshly prepared Carnoy's fixative by centrifuging at 3615 ***g*** for 12 min. The suspension was dropped onto a wet and inclined slide using a sigmacote‐treated glass dropper to spread the chromosomes. After air drying, these slides were further fixed in Carnoy's fixative and then dehydrated in 95% ethanol for 3 min. Good pachytene chromosome spreads were selected and stored at 4 °C for later use.

### Fluorescence *in situ* hybridization

After heating at 65 °C for 30 min, the selected slides were rinsed in 0.01 m HCl for 2 min, incubated in 5 μg/mL pepsin in 0.01 m HCl at 37 °C for 8 min, washed once in distilled water and twice in 2X SSC (15 mm sodium chloride and 1.5 mm sodium citrate) for 5 min each, incubated in formaldehyde buffer (1X PBS, 50 mm MgCl_2_ and 1% formaldehyde) for 10 min at room temperature, washed three times in 2X SSC for 5 min, dehydrated in an ethanol series (70%, 90% and 100%), and finally air‐dried. Meanwhile, the hybridization mixture (20 μL per slide, containing 50% formamide, 2X SSC, 10% sodium dextran sulphate, 50 mm phosphate buffer pH 7.0, 100–500 ng DNA probe) was boiled for 10 min, transferred to an ice‐bath for approximately 10 min and added onto the pretreated chromosome slides. The slides were then heated at 80 °C for 2.5 min to denature the DNA probe and chromosomal DNA. *In situ* hybridization was conducted at 37 °C overnight, followed by three posthybridization washes with 2X SSC for 5 min each, three stringent washes in SF50 (50% formamide in 2X SSC) at 42 °C for 5 min each, three washes in 2X SSC for 5 min each and one wash in 4T (0.5% Tween‐20 in 4X SSC) for 5 min. Then, the slide was treated with TNB (1% blocking reagent in 1X TN buffer, which contained 0.1 m Tris–HCl and 0.15 M NaCl, pH 7.5) at 37 °C for 30 min, washed in 4T for 5 min at room temperature, incubated with Avidin Texas‐Red (Vector Laboratories) diluted 1:800 in TNB at 37 °C for 1 h, and then washed once in 4T for 5 min and twice in TNT (0.5% Tween‐20 in 1X TN) for 5 min each. The slide was then incubated with biotinylated anti‐avidin D (Vector Laboratories) diluted 1:100 in TNB or sheep fluorescein isothiocyanate (FITC)‐conjugated antidigoxigenin antibody (Vector Laboratories) diluted 1:200 in TNB at 37 °C for 1 h and then washed three times in TNT for 5 min each. The slide then was incubated with Avidin Texas‐Red diluted 1:800 in TNB or anti‐sheep‐FITC (Vector Laboratories) diluted 1:800 in TNB at 37 °C for 1 h, washed twice in 2X SSC for 5 min, dehydrated in a graded ethanol series (70%, 90% and 100%) and finally air‐dried. Coverslips were mounted on slides with Vectashield (Vector Laboratories) containing 5 μg/mL 4′,6‐diamidino‐2‐phenylindole (DAPI). Markers for FISH mapping of *P. aphrodite* are listed in Table S5 in Appendix S3. The LG‐specific markers for FISH mapping in *P. equestris* are listed in Table S23 in Appendix S3).

### Microscopy and image capture system

Microscopy and photography were performed using a Nikon DS Ri1 CCD camera attached to a Nikon ECLIPSE 80i microscope equipped for phase‐contrast and fluorescence microscopy with DAPI, FITC and Texas‐Red. Images were captured with the NIS‐Elements microscope imaging software (Nikon). Chromosome straightening was performed using ImageJ (http://rsb.info.nih.gov/ij) software.

### Annotation of transposable element‐related sequences and pseudogenes

Transposable element (TE)‐related sequences were identified by both *de novo* structure prediction and RepeatMasker (http://www.repeatmasker.org) searches. *De novo* detection and classification of LTR‐retrotransposon‐like elements were performed using LTRharvest (Ellinghaus *et al*., 2008) and LTRdigest (Steinbiss *et al*., 2009). Moreover, we performed PseudoPipe (Zhang *et al*., 2006) to detect potential pseudogene sequences and then used the resulting candidates as queries in searches against the Pfam database to identify transposon‐related pseudogenic regions.

### Gene annotation

The Orchidstra database contained 190 065 transcripts that were generated from *de novo* transcriptome assembly for various tissues and growth stages of *P. aphrodite*. The transcripts were mapped to the genome assembly using the splice‐site aware program Exonerate (Slater and Birney, 2005). Of the 190 065 transcripts, 173 500 transcripts (91.28%) aligned to the *P. aphrodite* genome assembly with > 90% sequence identity and over 90% of the transcript length covered by a single scaffold. After searching against the NCBI nr database and manual annotation, we identified 10 398 transcripts that are likely to contain full protein coding sequences. These transcripts were subjected to GenomeThreader (Gremme *et al*., 2005) to predict gene structure. We performed manual curation of the GenomeThreader predicted gene models and then used the manually curated gene models to compile the data set for training gene prediction program Augustus (Stanke and Waack, 2003). To avoid over fitting, the amino acid sequences of the multi‐exon genes were clustered based on global sequence similarity using CD‐HIT (Li and Godzik, 2006), and sequences with more than 70% sequence identity were removed. The remaining 8074 gene models were divided into two separate gene sets, one with 7924 gene models for training Augustus and another set with 150 gene models (containing 864 exons in total) for accessing the accuracy of the model built on the training set.

The RNA‐Seq reads (Chao *et al*., 2017) were aligned to the *P. aphrodite* genome assembly with TopHat (Trapnell *et al*., 2009), and the resulting alignments (bam files) were used in genome‐guided *de novo* transcriptome assembly by Trinity (Grabherr *et al*., 2011). The transcript contigs, pre‐annotated full‐length gene models and orthologous sequences from other species were used as the input for the MAKER (Cantarel *et al*., 2008) and PASA pipeline combined with EVidenceModeler (EVM) (Haas *et al*., 2008). During the annotation process, all *ab initio* gene prediction programs were run with the pre‐trained parameters. The gene models from the low‐complexity sequences without any transcript or protein homologue evidence, mainly generated from the MAKER pipeline, were filtered out. After performing protein domain/family analysis, we further manually reviewed the gene models, paying particular attention to genes corresponding to floral traits, transcription factors, transporter proteins, and the gene families discussed in this manuscript. Noncoding RNAs were identified using the Rfam database (release 12.1, http://rfam.xfam.org/) and previous known orchid microRNAs (Chao *et al*., 2014). Phylogenetic analysis was performed using MEGA 7 (Kumar *et al*., 2016). The fragments per kilobase of transcript per million mapped reads (FPKM) and transcripts per million (TPM) value for gene expression in *P. aphrodite* were computed using RSEM (Li and Dewey, 2011). Differential expression analysis is performed using DESeq2 (Love *et al*., 2014).

### Accession numbers

The raw sequencing data and the whole‐genome assembly of *P. aphrodite* from this study have been submitted to NCBI BioProject database (https://www.ncbi.nlm.nih.gov/bioproject/) under Accession number PRJNA383284. The data sets supporting the conclusions of this study are included within the article and its Appendix files, and are also available in http://orchidstra2.abrc.sinica.edu.tw/orchidstra2/pagenome.php.

## Conflict of interest

The authors declare no conflict of interest.

## Supporting information


**Figure S1** BUSCO analysis results for *P. aphrodite, P. equestris and D. catenatum*.
**Figure S2** FISH mapping of linkage group‐specific genomic probes on *P. aphrodite* chromosome 1.
**Figure S3** FISH mapping of linkage group‐specific genomic probes on *P. aphrodite* chromosome 2.
**Figure S4** FISH mapping of linkage group‐specific genomic probes on *P. aphrodite* chromosome 3.
**Figure S5** FISH mapping of linkage group‐specific genomic probes on *P. aphrodite* chromosome 4.
**Figure S6** FISH mapping of linkage group‐specific genomic probes on *P. aphrodite* chromosome 5.
**Figure S7** FISH mapping of linkage group‐specific genomic probes on *P. aphrodite* chromosome 6.
**Figure S8** FISH mapping of linkage group‐specific genomic probes on *P. aphrodite* chromosome 7.
**Figure S9** FISH mapping of linkage group‐specific genomic probes on *P. aphrodite* chromosome 8.
**Figure S10** FISH mapping of linkage group‐specific genomic probes on *P. aphrodite* chromosome 9.
**Figure S11** FISH mapping of linkage group‐specific genomic probes on *P. aphrodite* chromosome 10.
**Figure S12** FISH mapping of linkage group‐specific genomic probes on *P. aphrodite* chromosome 11.
**Figure S13** FISH mapping of linkage group‐specific genomic probes on *P. aphrodite* chromosome 12.
**Figure S14** FISH mapping of linkage group‐specific genomic probes on *P. aphrodite* chromosome 13.
**Figure S15** FISH mapping of linkage group‐specific genomic probes on *P. aphrodite* chromosome 14.
**Figure S16** FISH mapping of linkage group‐specific genomic probes on *P. aphrodite* chromosome 15.
**Figure S17** FISH mapping of linkage group‐specific genomic probes on *P. aphrodite* chromosome 16.
**Figure S18** FISH mapping of linkage group‐specific genomic probes on *P. aphrodite* chromosome 17.
**Figure S19** FISH mapping of linkage group‐specific genomic probes on *P. aphrodite* chromosome 18.
**Figure S20** FISH mapping of linkage group‐specific genomic probes on *P. aphrodite* chromosome 19.
**Figure S21** FISH mapping of LG16‐specific genomic probes and 45S rDNA on *P. equestris* chromosome.
**Figure S22** Flow cytometry analysis.
**Figure S23** Locations of recombination hotspots along each chromosome.
**Figure S24** Gene expression heatmap for the full‐length *FAR1/FRS* genes.
**Figure S25** Phylogenetic trees of the MADS‐box gene family in orchids.
**Figure S26** Expression of flavonoid biosynthetic pathway genes in *P. aphrodite* and *P. lueddemanniana*.
**Table S1** Metrics for sequencing data used in SOAPdenovo2 assembly.
**Table S2** Metrics for sequencing data used in ALLPATHS‐LG assembly and SSPACE scaffolding.
**Table S3** Quality control metrics for the SOAPdenovo2 assembly.
**Table S4** Quality control metrics for the ALLPATHS‐LG assembly and SSPACE scaffolding.

**Appendix S2** This file contains genetic map and scaffold arrangement of chromosomes 1–19.
**Appendix S3** This file contains Tables S5–S10, Tables S13–S14, and Tables S20–S23.

**Table S5** List of FISH probes.
**Table S6** Relative positions of FISH probes on pachytene chromosomes.
**Table S7** Coordinates of recombination hot spots, list of genes within the hot spots and gene density.
**Table S8** Annotation of genes within the recombination hot spots.
**Table S9** List of recombination cold spots and gene density.
**Table S10** List of genes within the cold hot spots and gene annotation.
**Table S11** Orchidstra 2.0 ESTs that map to *P. aphrodite* genome.
**Table S12** Annotated protein‐coding genes in *P. aphrodite*.
**Table S13** List of transcription factors in *P. aphrodite*.
**Table S14** List of transporters in *P. aphrodite*.
**Table S15** The number and type of non‐coding RNAs in *P. aphrodite*.
**Table S16** The number of genes in shared and species‐specific gene families in ten representative plant species.
**Table S17** Number of MADS‐box genes in different species.
**Table S18** Number of flavonoid biosynthesis‐related genes in different species.
**Table S19** Number of carotenoid biosynthesis‐related genes in different species.
**Table S20** Annotation of color, scent and flowering genes.
**Table S21** Differential expression analysis with DESeq2 for comparison between the tissues in *P. aphrodite*.
**Table S22** Differential expression analysis with DESeq2 for comparison between the tissues in *P. lueddemanniana*.
**Table S23** Using inkage group‐specific clones to identify the misassembled scaffolds in *P. equestris* draft genome.
**Appendix S1** This file contains Supporting Text, Figures S1–S26, and Tables S1–S4, S11–S12, S15–S19.
