# Supplementary material for: Chromosome‐level assembly, genetic and physical mapping of Phalaenopsis aphrodite genome provides new insights into species adaptation and resources for orchid breeding
Source: Plant Biotechnol J. 2018 May 23;16(12):2027–41. doi: 10.1111/pbi.12936 (PMC6230949; doi:10.1111/pbi.12936)
Supplement: Supplementary file 1 — Figure S1 BUSCO analysis results for P. aphrodite, P. equestris and D. catenatum. Figure S2 FISH mapping of linkage group‐specific genomic probes on P. aphrodite chromosome 1. Figure S3 FISH mapping of linkage group‐specific genomic probes on P. aphrodite chromosome 2. Figure S4 FISH mapping of linkage group‐specific genomic probes on P. aphrodite chromosome 3. Figure S5 FISH mapping of linkage group‐specific genomic probes on P. aphrodite chromosome 4. Figure S6 FISH mapping of linkage group‐specific genomic probes on P. aphrodite chromosome 5. Figure S7 FISH mapping of linkage group‐specific genomic probes on P. aphrodite chromosome 6. Figure S8 FISH mapping of linkage group‐specific genomic probes on P. aphrodite chromosome 7. Figure S9 FISH mapping of linkage group‐specific genomic probes on P. aphrodite chromosome 8. Figure S10 FISH mapping of linkage group‐specific genomic probes on P. aphrodite chromosome 9. Figure S11 FISH mapping of linkage group‐specific genomic probes on P. aphrodite chromosome 10. Figure S12 FISH mapping of linkage group‐specific genomic probes on P. aphrodite chromosome 11. Figure S13 FISH mapping of linkage group‐specific genomic probes on P. aphrodite chromosome 12. Figure S14 FISH mapping of linkage group‐specific genomic probes on P. aphrodite chromosome 13. Figure S15 FISH mapping of linkage group‐specific genomic probes on P. aphrodite chromosome 14. Figure S16 FISH mapping of linkage group‐specific genomic probes on P. aphrodite chromosome 15. Figure S17 FISH mapping of linkage group‐specific genomic probes on P. aphrodite chromosome 16. Figure S18 FISH mapping of linkage group‐specific genomic probes on P. aphrodite chromosome 17. Figure S19 FISH mapping of linkage group‐specific genomic probes on P. aphrodite chromosome 18. Figure S20 FISH mapping of linkage group‐specific genomic probes on P. aphrodite chromosome 19. Figure S21 FISH mapping of LG16‐specific genomic probes and 45S rDNA on P. equestris chromosome. Figure S22 Flo [file PBI-16-2027-s002.pdf]

Chromosome-level assembly, genetic and physical mapping of *Phalaenopsis aphrodite* genome provides new insights into species adaptation and resources for orchid breeding

Appendix S1

Table of Contents

Supporting Text **3**

Genomic library preparation **3**

De novo assembly of *P. aphrodite* genome **4**

Misassemble errors in the *P. equestris* draft genome **5**

Supporting Figures **6**

Figure S1 BUSCO analysis results for *P. aphrodite, P. equestris and D. catenatum* **6**

Figure S2 FISH mapping of linkage group-specific genomic probes on *P. aphrodite* chromosome 1 **7**

Figure S3 FISH mapping of linkage group-specific genomic probes on *P. aphrodite* chromosome 2 **8**

Figure S4 FISH mapping of linkage group-specific genomic probes on *P. aphrodite* chromosome 3 **9**

Figure S5 FISH mapping of linkage group-specific genomic probes on *P. aphrodite* chromosome 4 **10**

Figure S6 FISH mapping of linkage group-specific genomic probes on *P. aphrodite* chromosome 5 **11**

Figure S7 FISH mapping of linkage group-specific genomic probes on *P. aphrodite* chromosome 6 **12**

Figure S8 FISH mapping of linkage group-specific genomic probes on *P. aphrodite* chromosome 7 **13**

Figure S9 FISH mapping of linkage group-specific genomic probes on *P. aphrodite* chromosome 8 **13**

Figure S10 FISH mapping of linkage group-specific genomic probes on *P. aphrodite* chromosome 9 **15**

Figure S11 FISH mapping of linkage group-specific genomic probes on *P. aphrodite* chromosome 10 **16**

Figure S12 FISH mapping of linkage group-specific genomic probes on *P. aphrodite* chromosome 11 **17**

Figure S13 FISH mapping of linkage group-specific genomic probes on *P. aphrodite* chromosome 12 **18**

Figure S14 FISH mapping of linkage group-specific genomic probes on *P. aphrodite* chromosome 13 **19**

Figure S15 FISH mapping of linkage group-specific genomic probes on *P. aphrodite* chromosome 14 **20**

Figure S16 FISH mapping of linkage group-specific genomic probes on *P. aphrodite* chromosome 15 **21**

Figure S17 FISH mapping of linkage group-specific genomic probes on *P. aphrodite* chromosome 16 **22**

Figure S18 FISH mapping of linkage group-specific genomic probes on *P. aphrodite* chromosome 17 **23**

Figure S19 FISH mapping of linkage group-specific genomic probes on *P. aphrodite* chromosome 18 **24**

Figure S20 FISH mapping of linkage group-specific genomic probes on *P. aphrodite* chromosome 19 **25**

Figure S21 FISH mapping of LG16-specific genomic probes and 45S rDNA on *P. equestris* chromosome. **26**

Figure S22 Flow cytometry analysis **27**

Figure S23 Locations of recombination hotspots along each chromosome **28**

Figure S24 Gene expression heatmap for the full-length *FAR1/FRS* genes **39**

Figure S25 Phylogenetic trees of the MADS-box gene family in orchids **40**

Figure S26 Expression of flavonoid biosynthetic pathway genes in *P. aphrodite* and *P. lueddemanniana* **41**

Supporting Tables 42

Table S1 Metrics for sequencing data used in SOAPdenovo2 assembly **42**

Table S2 Metrics for sequencing data used in ALLPATHS-LG assembly and SSPACE scaffolding **43**

Table S3 Quality control metrics for the SOAPdenovo2 assembly **44**

Table S4 Quality control metrics for the ALLPATHS-LG assembly and SSPACE scaffolding **45**

Table S11 Orchidstra 2.0 ESTs that map to *P. aphrodite* genome **46**

Table S12 Annotated protein-coding genes in *P. aphrodite* **47**

Table S15 The number and type of non-coding RNAs in *P. aphrodite* **48**

Table S16 The number of genes in shared and species-specific gene families in ten representative plant species **49**

Table S17 Number of MADS-box genes in different species **50**

Table S18 Number of flavonoid biosynthesis-related genes in different species **51**

Table S19 Number of carotenoid biosynthesis-related genes in different species **52**

References 53

Supporting Text

Genomic library preparation

The orchid petals were frozen and ground in liquid nitrogen. Genomic DNA was extracted by mixing with 10 volumes (w/v) of CTAB buffer (2% hexadecyltrimethylammonium bromide, 1% polyvinylpyrrolidone 40, 100 mM Tris–HCl pH 8.0, 25 mM EDTA pH 8.0, 1.5 M NaCl, 0.2% beta-mercaptoethanol, and Proteinase K 0.15 mg/ml) pre-warmed at 65°C. The homogenate was incubated at 65°C for 15 min with frequent mixing. The homogenate was centrifuged at 3,000 × g for 10 min at room temperature. The supernatant was extracted once with an equal volume of chloroform: isoamyl alcohol (24:1, v/v) and centrifuged at 12,000 × g for 10 min. One-half volume of 5 M sodium chloride was added to the aqueous phase and then mixed with 0.6 volume cold isopropanol and incubated at room temperature for 1 hour to precipitate genomic DNA. The DNA pellet was harvested by centrifugation at 12,000 × g for 20 min at room temperature, washed with ice-cold 75% ethanol, and suspended in high salt TE buffer (~1 ml buffer per 1 g tissue) using tips with the ends cut off. The resuspended solution was incubated in a 60°C incubator for 15 min to dissolve the pellet completely. For removal of RNA from DNA samples, RNase treatment was performed for 20 min at 37°C. After RNase treatment, an equal volume of chloroform: isoamyl alcohol (24:1, v/v) was added, and the DNA samples were centrifuged at 12,000 × g for 10 min. Genomic DNA was recovered by EtOH precipitation followed by washes with 70% EtOH. Finally, the DNA pellet was resuspended in DNase-free water.

DNA purity and concentration were determined by Nanodrop 2000 (Thermo Fisher) and Qubit (Invitrogen) measurements. The quality of DNA was evaluated by separating 100 ng DNA on a 1% TAE gel and staining. Paired-end genomic DNA libraries were created from 2-5 µg of genomic DNA using the Truseq Paired End library preparation kit (Illumina). The mate pair libraries with 3, 5, 7, 8, 10 and 15 kb inserts were created using the Nextera Mate Pair library preparation kit (Illumina) from 2-5 µg genomic DNA. For all procedures the protocols provided by the manufacturer were used. Another 3 kb mate pair library was generated from 5 µg of genomic DNA using the Mate pair v2 Kit (Illumina). The 40 kb mate pair library was generated by Lucigen Corporation, USA.

***De novo* assembly of *P. aphrodite* genome**

We generated 21 Illumina genomic DNA sequencing libraries with insert sizes ranging from 150 bp to 15 kb, and a 40 kb fosmid library. Genomic DNA read trimming and quality filtering were performed using Cutadapt (Martin, 2011) (paired-end reads) or NxTrim (O'Connell et al., 2015) (mate pair reads). Reads shorter than 30 bases (paired-end reads) or 40 bases (mate pair reads) after trimming were discarded. After filtering out adapters and low-quality sequences, several *de novo* assemblies were generated independently with ALLPATHS-LG (Gnerre et al., 2011) and SOAPdenovo2 (Luo et al., 2012) using reads from different libraries with different coverage because these programs require different types of libraries as input (Tables S1 and S2). The libraries used for the SOAPdenovo2 assembly included ten paired-end genomic libraries and three mate-pair libraries with a total coverage level of 310X and one 40 kb fosmid library with ~1.5 X coverage (Table S1). The SOAPdenovo2 assembly was highly fragmented (Table S3). The best assembly from SOAPdenovo2 contained 67,500 scaffolds (> 800 bp) with an average scaffold length of 19.8 kb and a scaffold N50 of 187.8 kb (Table S3). To meet the specific sequencing library requirements of the ALLPATHS-LG assembler, we created two small-insert genomic libraries (fragment libraries) which resulted in overlapping sequenced paired-end reads. The ALLPATHS-LG assembly was performed with 52X overlapping read pairs from fragment libraries and 54X mate-pair reads from libraries with insert sizes ranging from 3 kb to 7 kb (jump libraries) (Table S2). The ALLPATHS-LG assembly consisted of 20,848 scaffolds (> 800 bp) with an average length of 47.2 kb and an N50 of 417.5 kb (Table S4). As another measure of assembly quality, we determined the percentage of full-length protein-coding *P. aphrodite* ESTs that map to the ALLPATHS-LG assembly. We found that 96.7% of these ESTs align over 90% of their length within a single scaffold. Only 41.8% of the full-length protein-coding ESTs aligned over 90% of their length to the SOAPdenovo2 assembly. Based on genome assembly statistics, including N50 and the number of scaffolds, and the high percentage of mapping full-length protein-coding transcripts, the ALLPATHS-LG procedure resulted in better assembly quality. To further improve continuity, the ALLPATHS-LG scaffolds were linked and extended using SSPACE with mate-pair libraries ranging from 8 to 15 kb (a total coverage of 49X) and the 40 kb fosmid library (1.5X) (Table S4). The resulting assembly had 13,732 genomic scaffolds with a total length of 1025.1 Mb. Moreover, the number of full-length ESTs that aligned over 90% of their length to a single scaffold reached 10,199 (98.1% of total full-length ESTs).

Misassemble errors in the *P. equestris* draft genome

In this study, we have successfully developed linkage group (LG)-specific FISH markers for *P. aphrodite* (Figures S2-S20). Besides using the LG-specific FISH markers to distinguish particular chromosomes and assign genome sequence assemblies to chromosomes in *P. aphrodite*, we have used some of these FISH markers to investigate the chromosome colinearity between *P. aphrodite* and *P. equestris*. The FISH mapping in *P. equestris*, which has the same number of chromosomes as *P. aphrodite*, showed that the LG16-specific markers 45S, PABC065-3-D04, PABC059-1-H02, and PABC094-4-F04 were detected on the same chromosome of *P. equestris* (Figure S21), and the markers' relative positions are also consistent with the FISH mapping of *P. aphrodite* chromosome 16 (Figure S17). Moreover, the LG12-specific markers DL17-S30, PEPC, DL17-S959 and DL17-S545 were located on the same chromosome of *P. equestris* and their relative locations were consistent with the mapping result of chromosome 12 of *P. aphrodite*. LG13-specific markers DL02-S169, DL02-S21, and DL02-S46 were mapped to the same chromosome of *P. equestris* and their relative positions on chromosome 13 were preserved (data not shown). The conservation of marker order and localization displays a high degree of chromosomal collinearity and indicates the absence of large-scale chromosomal rearrangements between these two species.

After in silico alignment of the linkage group-specific FISH markers with *P. equestris* genome assemblies, we found that 74 out of 76 FISH markers were uniquely mapped to *P. equestris* genomic scaffolds, indicating that the markers are highly specific and conserved across the two species. However, we found that the largest scaffold of *P. equestris*, a single scaffold that accounts for more than 10% of the genome assemblies, could be aligned with sequences of several markers belonging to five different linkage groups (LG01, LG02, LG03, LG16, LG19) (Table S23 in Appendix S3). Moreover, there are many large genomic scaffolds each aligns to two different chromosomes, in other words, these scaffolds contain markers of two different linkage groups (Table S23 in Appendix S3). The results show that the *P. equestris* draft genome are severely misassembled.

Supporting Figures

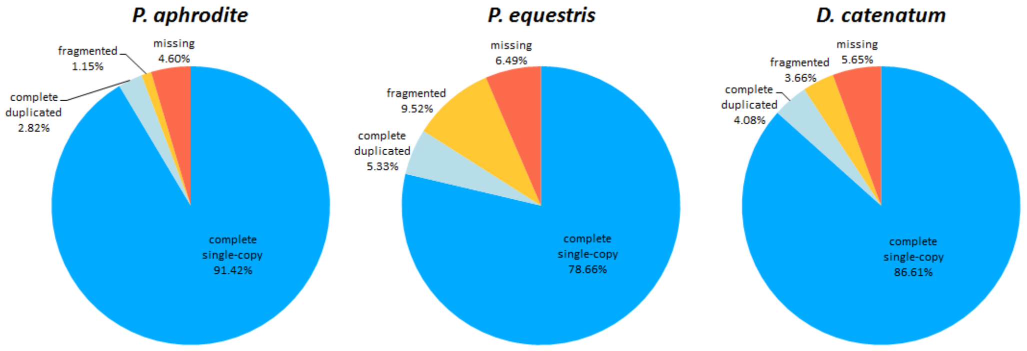

Figure S1 BUSCO analysis results for *P. aphrodite*, *P. equestris* and *D. catenatum*.

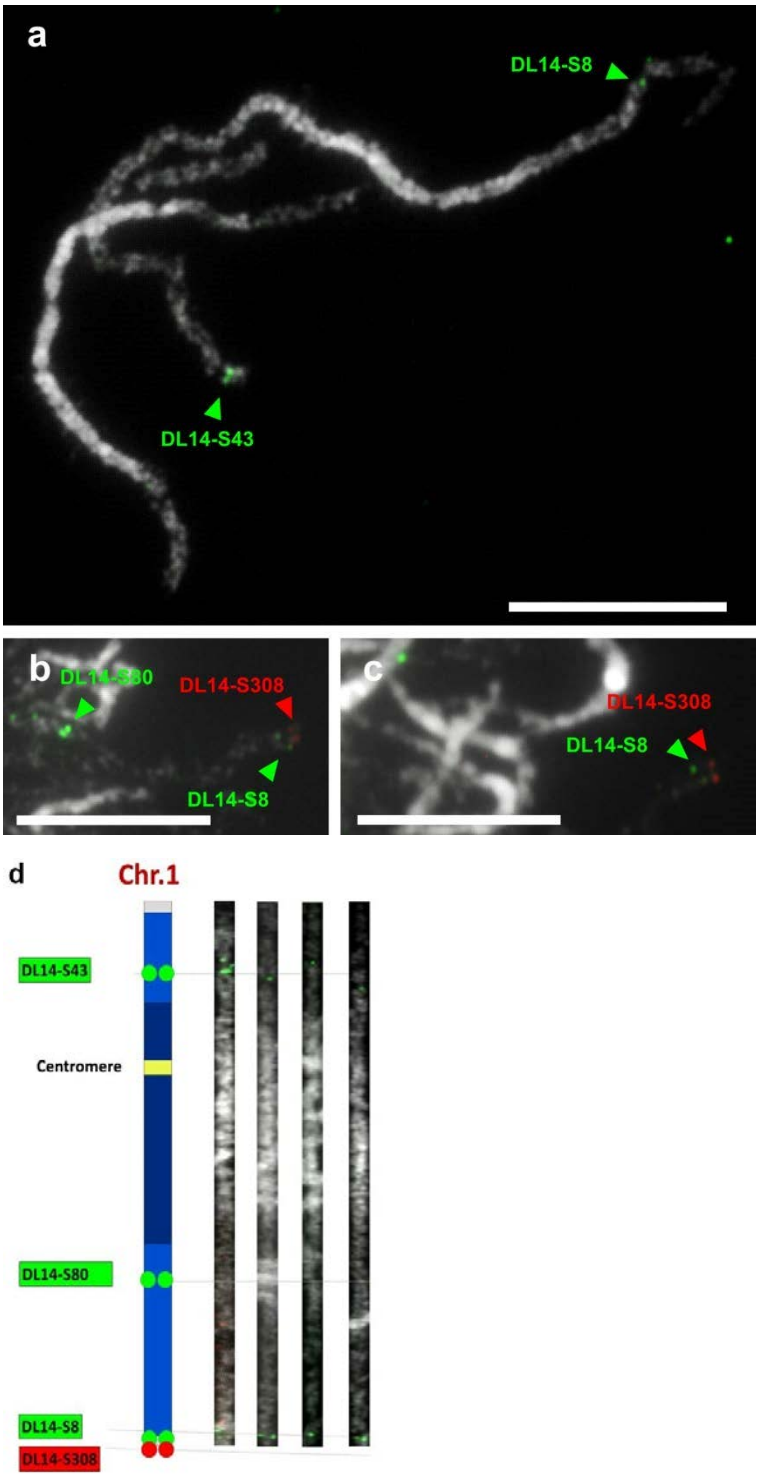

Figure S2 FISH mapping of linkage group-specific genomic probes on *P. aphrodite* chromosome 1. **(**a) FISH mapping of DL14-S8 and DL14-S43 probes (b) FISH mapping of DL14-S80, DL14-S308 and DL14-S8 probes (c) FISH mapping of DL14-S308 and DL14-S8 probes on the pachytene chromosome 1. The images of DAPI-stained chromosomes were converted to black and white. Scale bar = 10 µm. (d) Four computationally straightened chromosome 1.

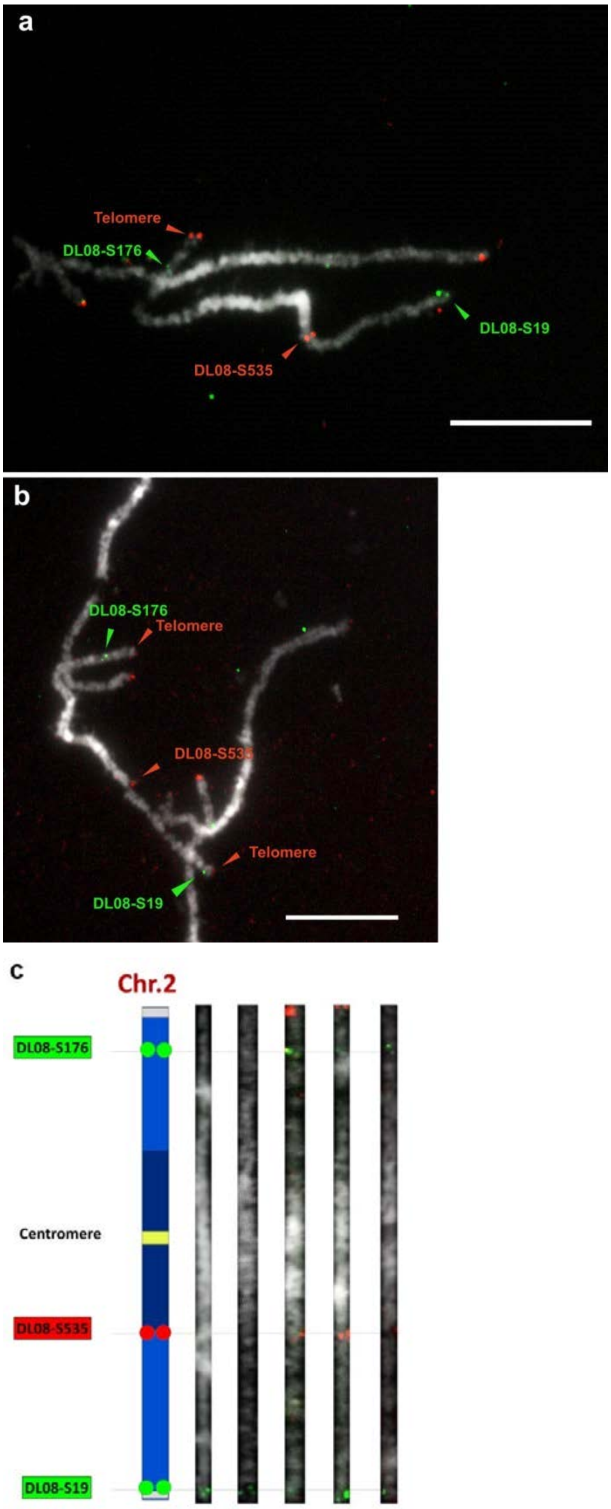

Figure S3 FISH mapping of linkage group-specific genomic probes on *P. aphrodite* chromosome 2. **(**a) FISH mapping of DL08-S176, DL08-S535 and DL08-S19 probes (b) FISH mapping of DL08-S176, DL08-S535 and DL08-S19 probes on the pachytene chromosome 2. The images of DAPI-stained chromosomes were converted to black and white. Scale bar = 10 μm. (c) Five computationally straightened chromosome 2.

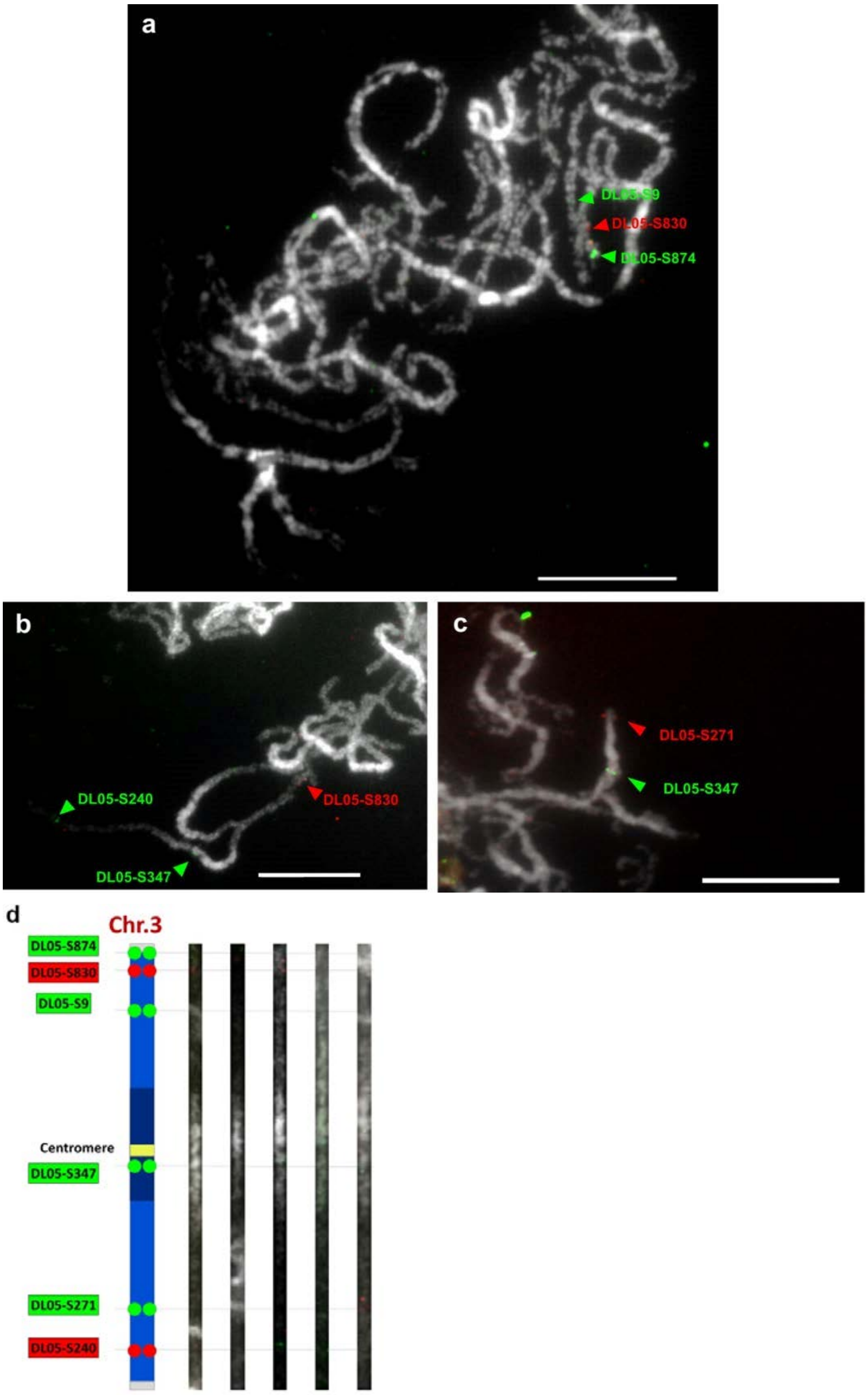

Figure S4 FISH mapping of linkage group-specific genomic probes on *P. aphrodite* chromosome 3. **(**a) FISH mapping of DL05-S9, DL05-S830 and DL05-S874 probes (b) FISH mapping of DL05-S240, DL05-S347 and DL05-S830 probes (c) FISH mapping of DL05-S271 and DL05-S347 probes on the pachytene chromosome 3. The images of DAPI-stained chromosomes were converted to black and white. Scale bar = 10 µm. (d) Five computationally straightened chromosome 3.

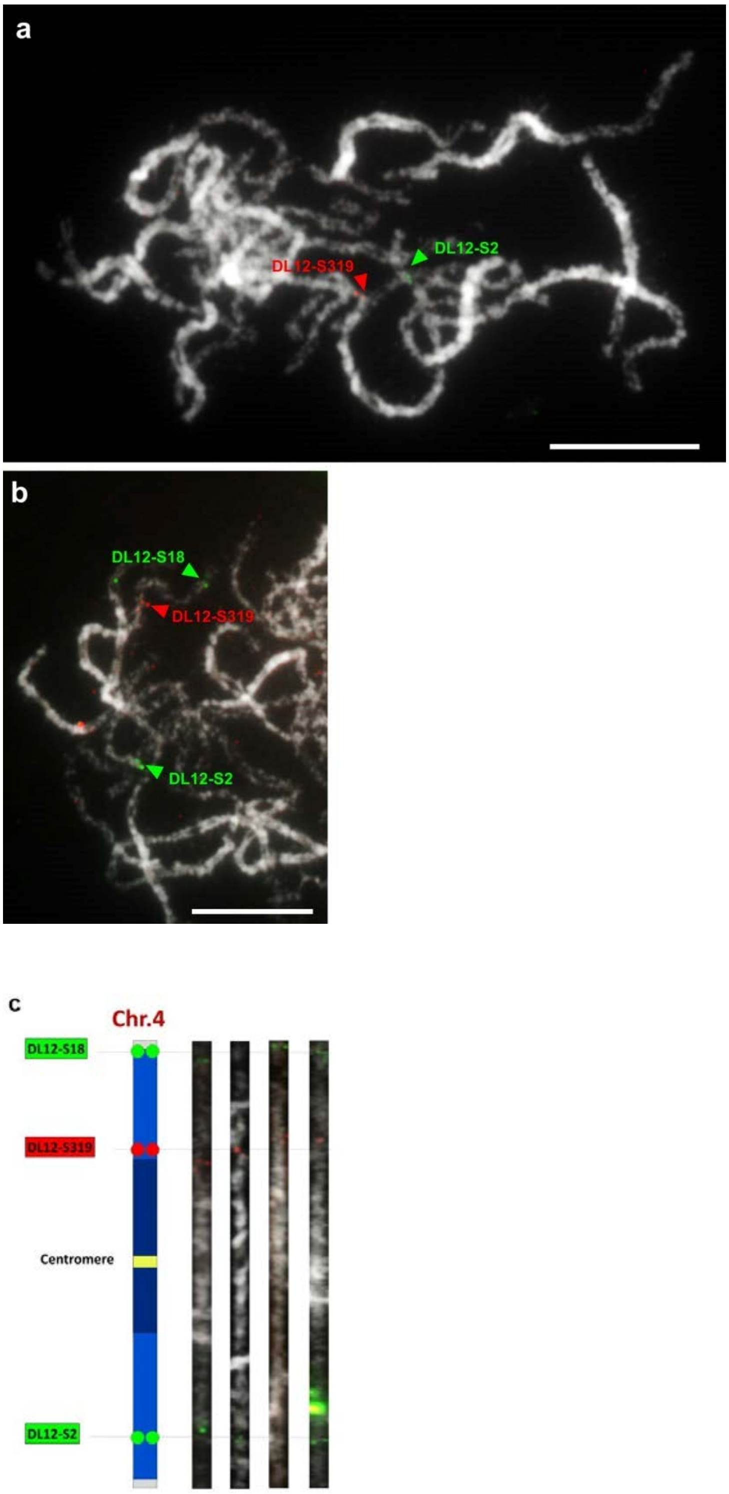

Figure S5 FISH mapping of linkage group-specific genomic probes on *P. aphrodite* chromosome 4. (a) FISH mapping of DL12-S319 and DL12-S2 probes (b) FISH mapping of DL12-S18, DL12-S319 and DL12-S2 probes on the pachytene chromosome 4. The images of DAPI-stained chromosomes were converted to black and white. Scale bar = 10 μm. (c) Four computationally straightened chromosome 4.

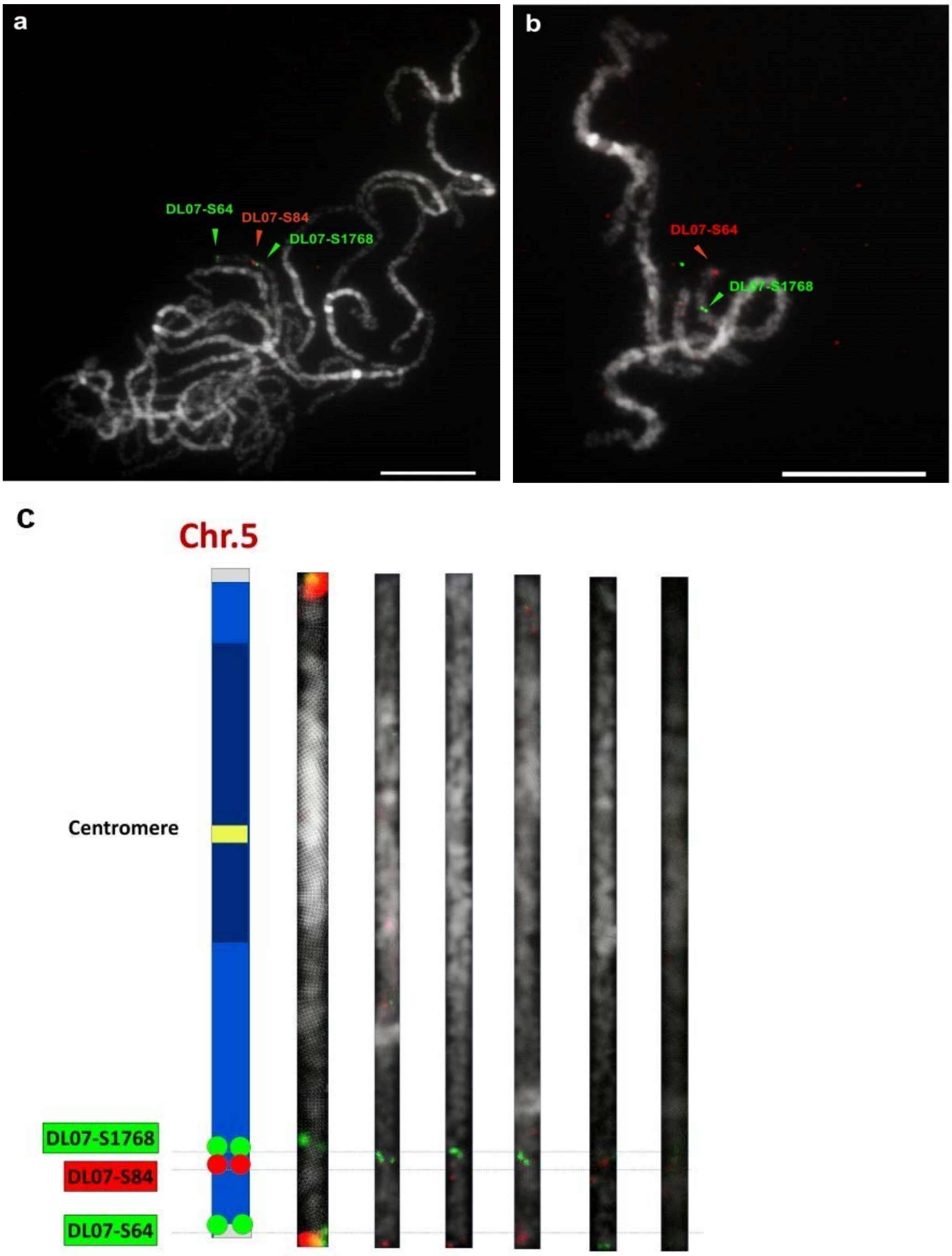

Figure S6 FISH mapping of linkage group-specific genomic probes on *P. aphrodite* chromosome 5. (a) FISH mapping of DL07-S64. DL07-S84 and DL07-S1768 probes (b) FISH mapping of DL07-S64 and DL07-S1768 probes on the pachytene chromosome 5. The images of DAPI-stained chromosomes were converted to black and white. Scale bar = 10 μm. (c) Six computationally straightened chromosome 5.

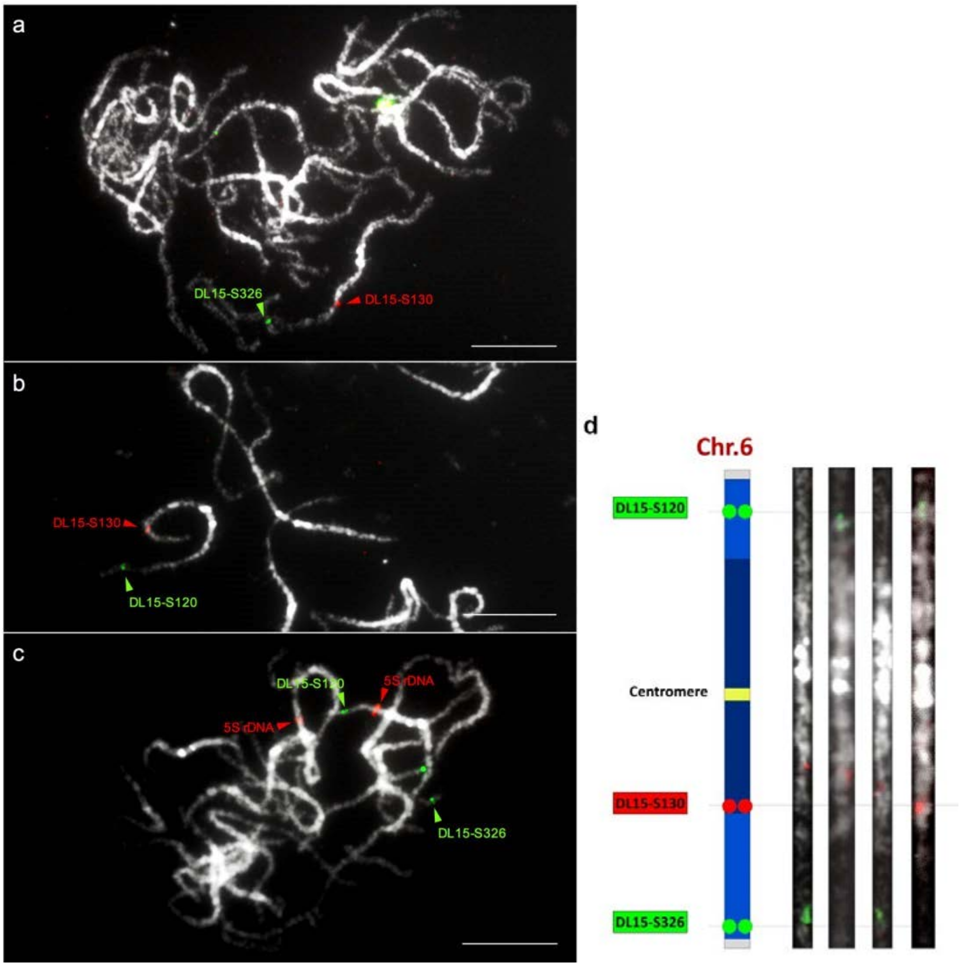

Figure S7 FISH mapping of linkage group-specific genomic probes on *P. aphrodite* chromosome 6. (a) FISH mapping of DL15-S326 and DL15-S130 probes (b) FISH mapping of DL15-S130 and DL15-S120 probes (c) FISH mapping of DL15-S120 and DL15-S326 probes on the pachytene chromosome 6. The images of DAPI-stained chromosomes were converted to black and white. Scale bar = 10 μm. (d) Four computationally straightened chromosome 6.

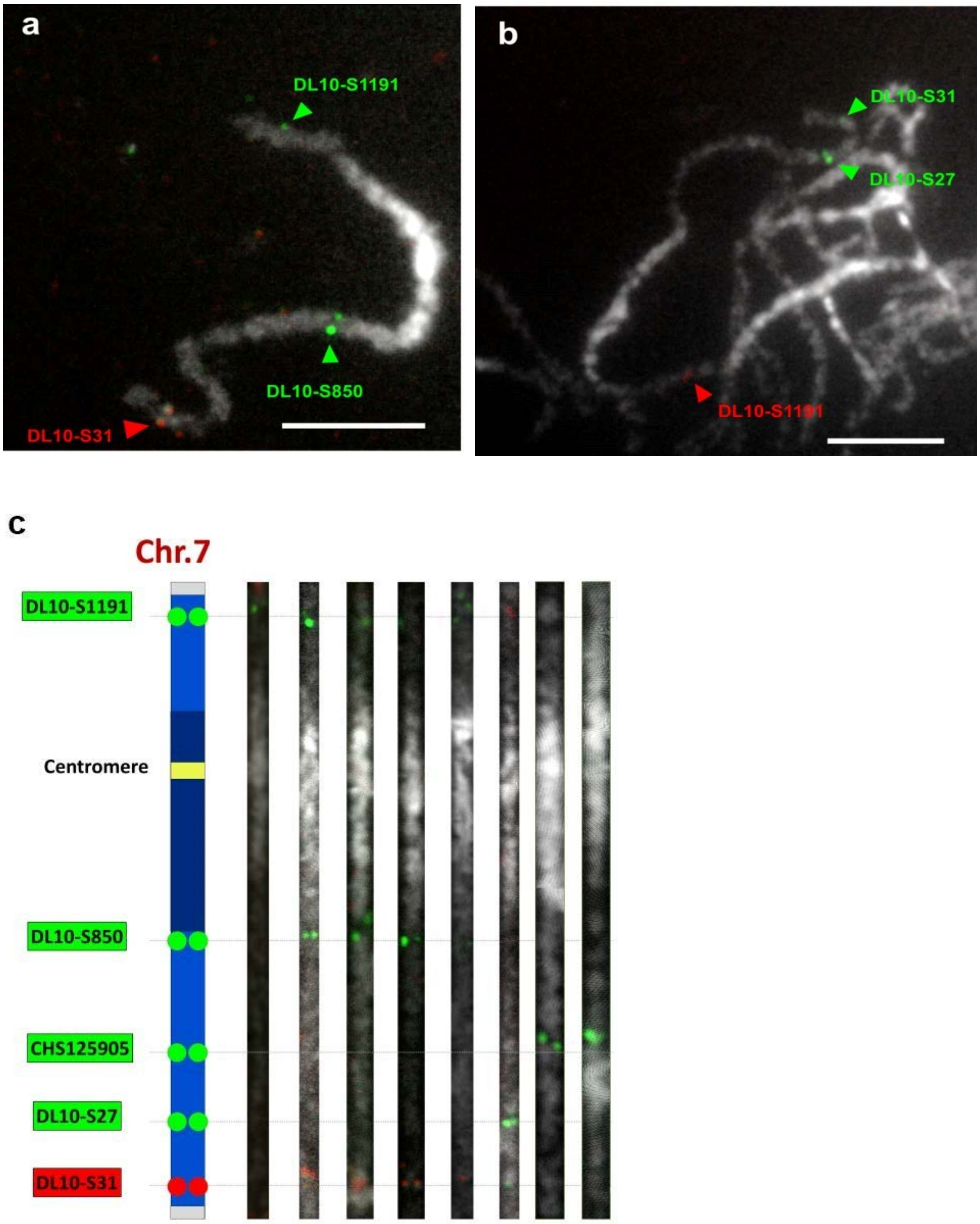

Figure S8 FISH mapping of linkage group-specific genomic probes on *P. aphrodite* chromosome 7. **(**a) FISH mapping of DL10-S1191, DL10-S850 and DL10-S31 probes (b) FISH mapping of DL10-S31, DL10-S27 and DL10-S1191 probes on the pachytene chromosome 7. The images of DAPI-stained chromosomes were converted to black and white. Scale bar = 10 μm. (c) Eight computationally straightened chromosome 7.

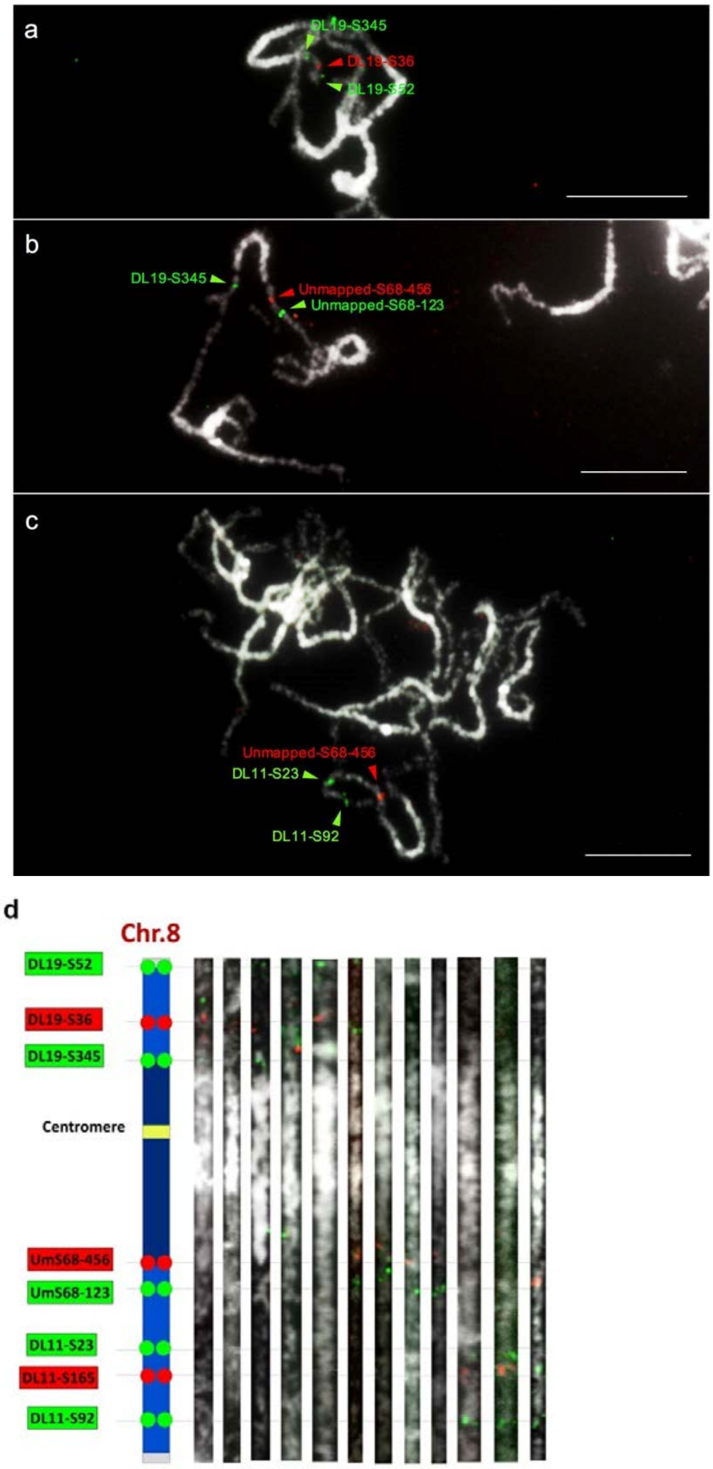

Figure S9 FISH mapping of linkage group-specific genomic probes on *P. aphrodite* chromosome 8. **(**a) FISH mapping of DL19-S345, DL19-S36 and DL19-S52 probes (b) FISH mapping of DL19-S345, unmapped-S68-456 and unmapped S68-123 probes (c) FISH mapping of DL11-S23, DL11-S92 and unmapped-S68-456 probes on the pachytene chromosome 8. The images of DAPI-stained chromosomes were converted to black and white. Scale bar = 10 μm. (d) Twelve computationally straightened chromosome 8.

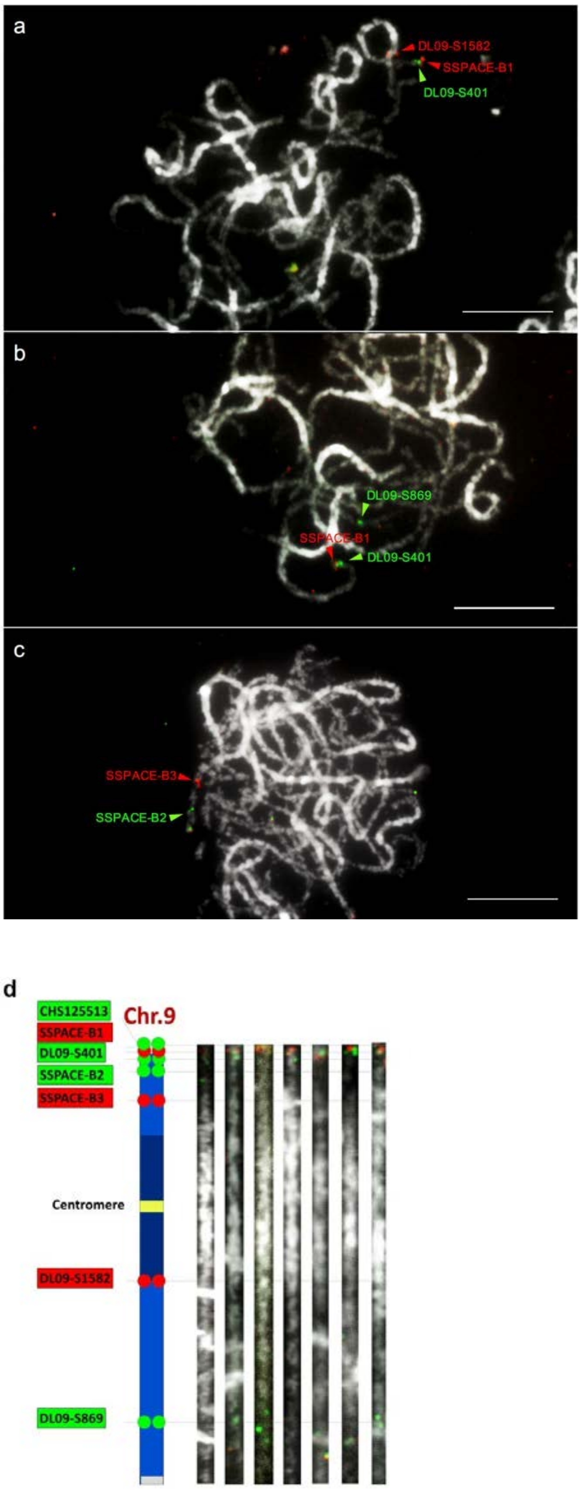

Figure S10 FISH mapping of linkage group-specific genomic probes on P. aphrodite chromosome 9. (a) FISH mapping of DL09-S1582, DL09-S401 and SSPACE-B1 probes (b) FISH mapping of DL09-S869, DL09-S401 and SSPACE-B1 probes (c) FISH mapping of SSPACE-B2 and SSPACE-B3 probes on the pachytene chromosome 9. The images of DAPI-stained chromosomes were converted to black and white. Scale bar = 10 µm. (d) Seven computationally straightened chromosome 9.

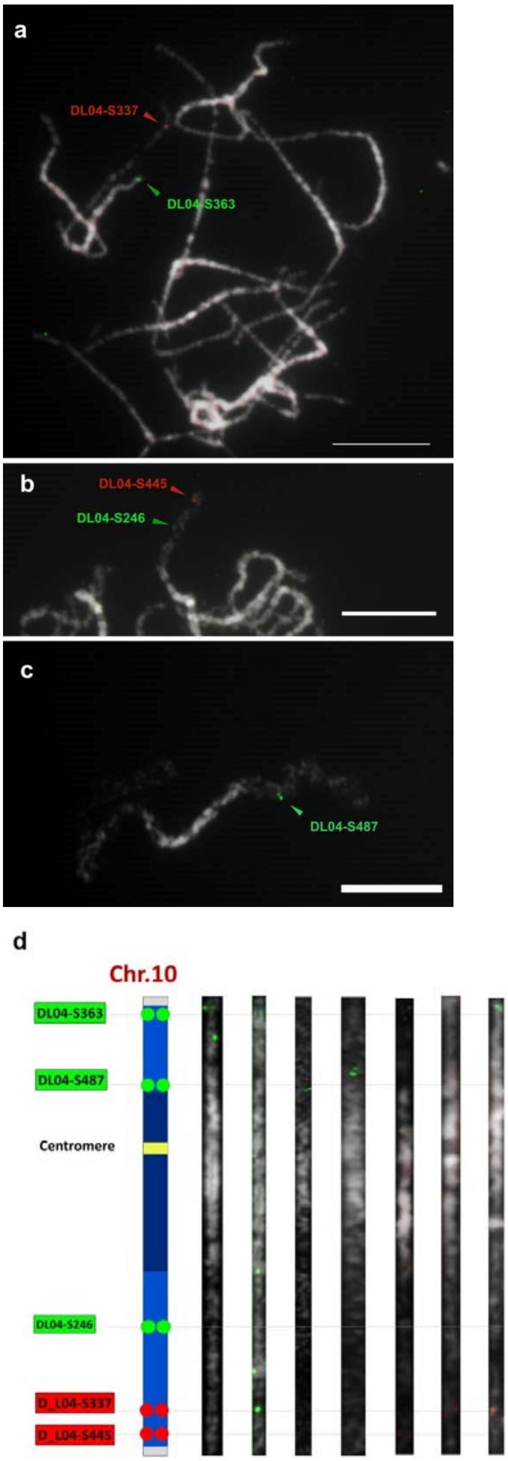

Figure S11 FISH mapping of linkage group-specific genomic probes on *P. aphrodite* chromosome 10. **(**a) FISH mapping of DL04-S337 and DL04-S363 probes (b) FISH mapping of DL04-S445 and DL04-S246 probes (c) FISH mapping of DL04-S487 probe on the pachytene chromosome 10. The images of DAPI-stained chromosomes were converted to black and white. Scale bar = 10 μm. (d) Seven computationally straightened chromosome 10.

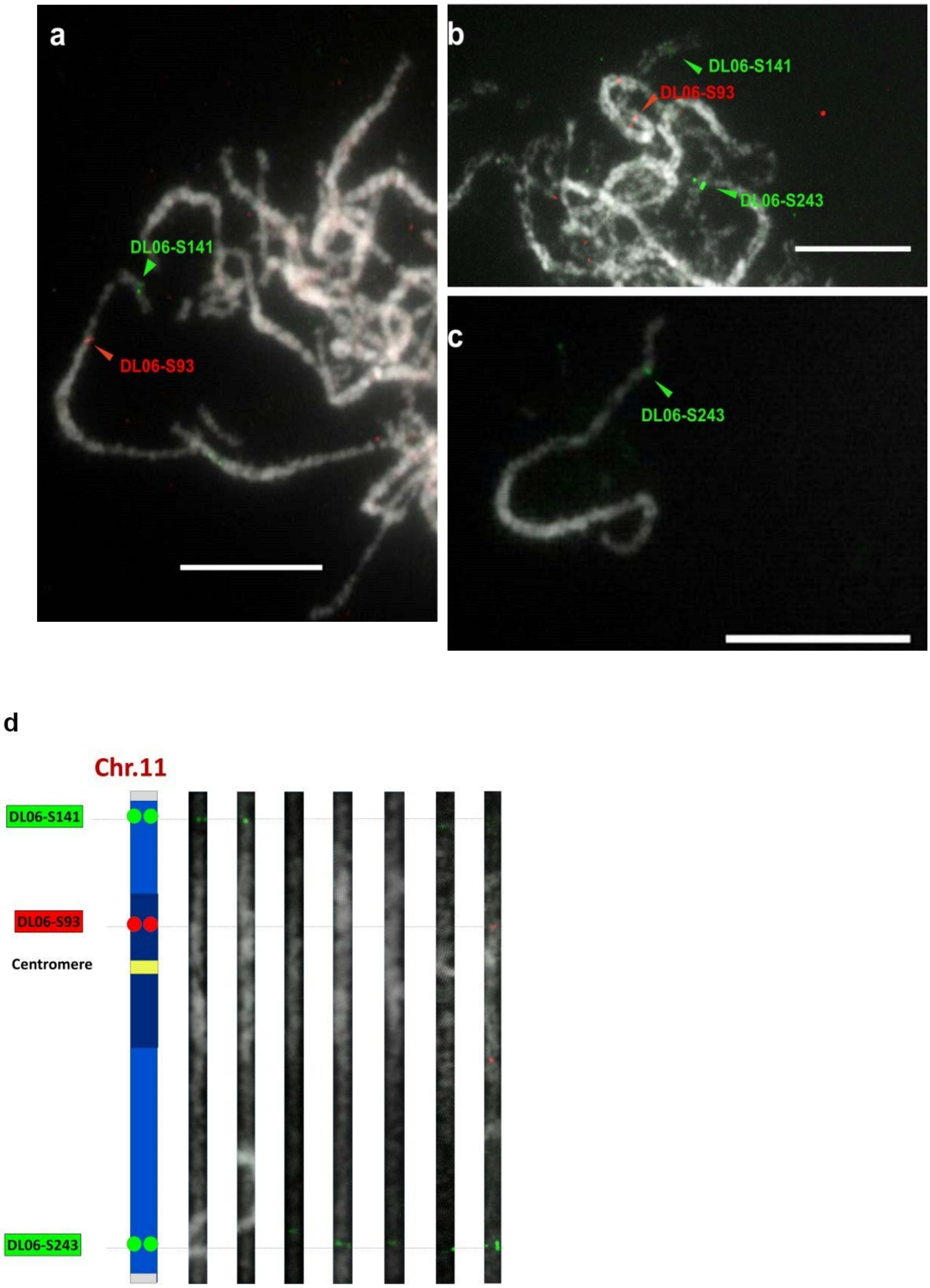

Figure S12 FISH mapping of linkage group-specific genomic probes on P. aphrodite chromosome 11. (a) FISH mapping of DL06-S141 and DL06-S93 probes (b) FISH mapping of DL06-S141, DL06-S93 and DL06-S243 probes (c) FISH mapping of DL06-S243 probe on the pachytene chromosome 11. The images of DAPI-stained chromosomes were converted to black and white. Scale bar = 10 μm. (d) Seven computationally straightened chromosome 11.

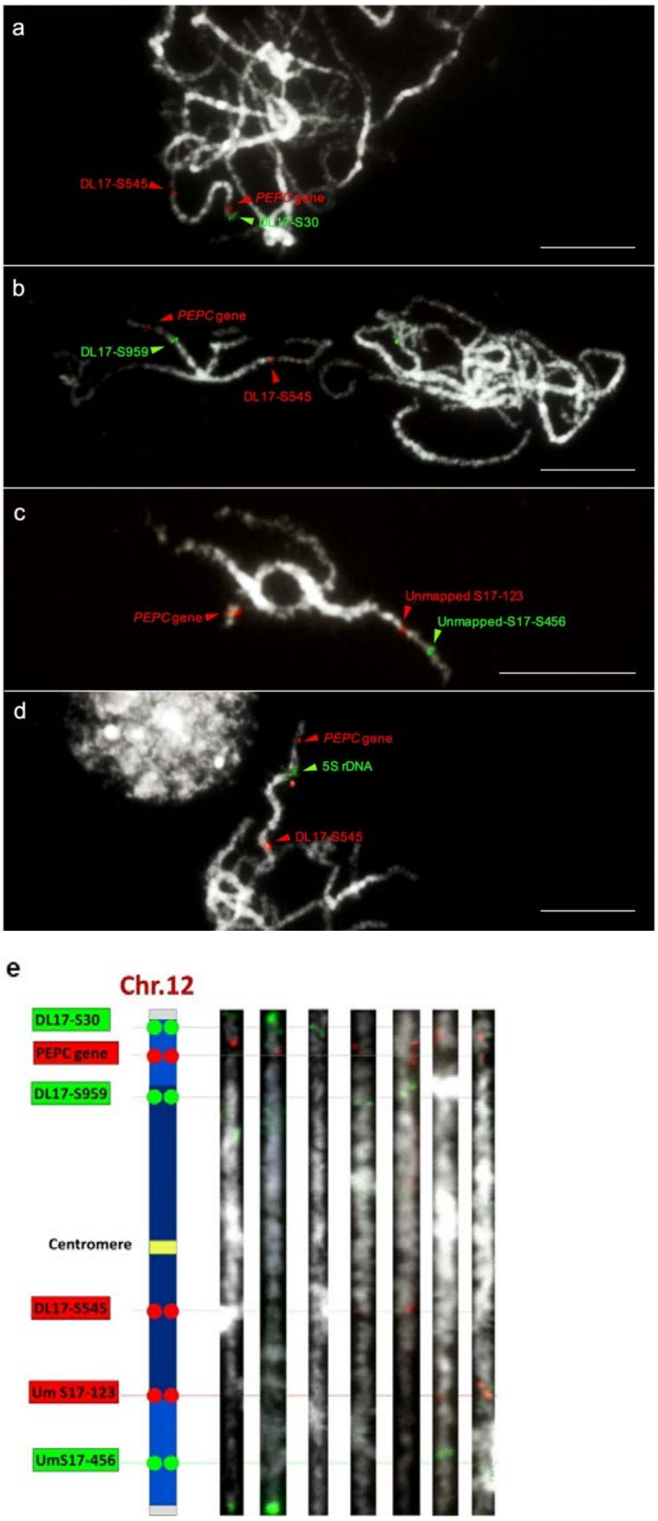

Figure S13 FISH mapping of linkage group-specific genomic probes on *P. aphrodite* chromosome 12. (a) FISH mapping of DL17-S545 and DL17-S30 probes (b) FISH mapping of DL17-S959 and DL17-S545 probes (c) FISH mapping of unmapped S17-123 and unmapped-S17-S456 (d) FISH mapping of DL17-S545 probe on the pachytene chromosome 12. Scale bar = 10 μm. (e) Seven computationally straightened chromosome 12.

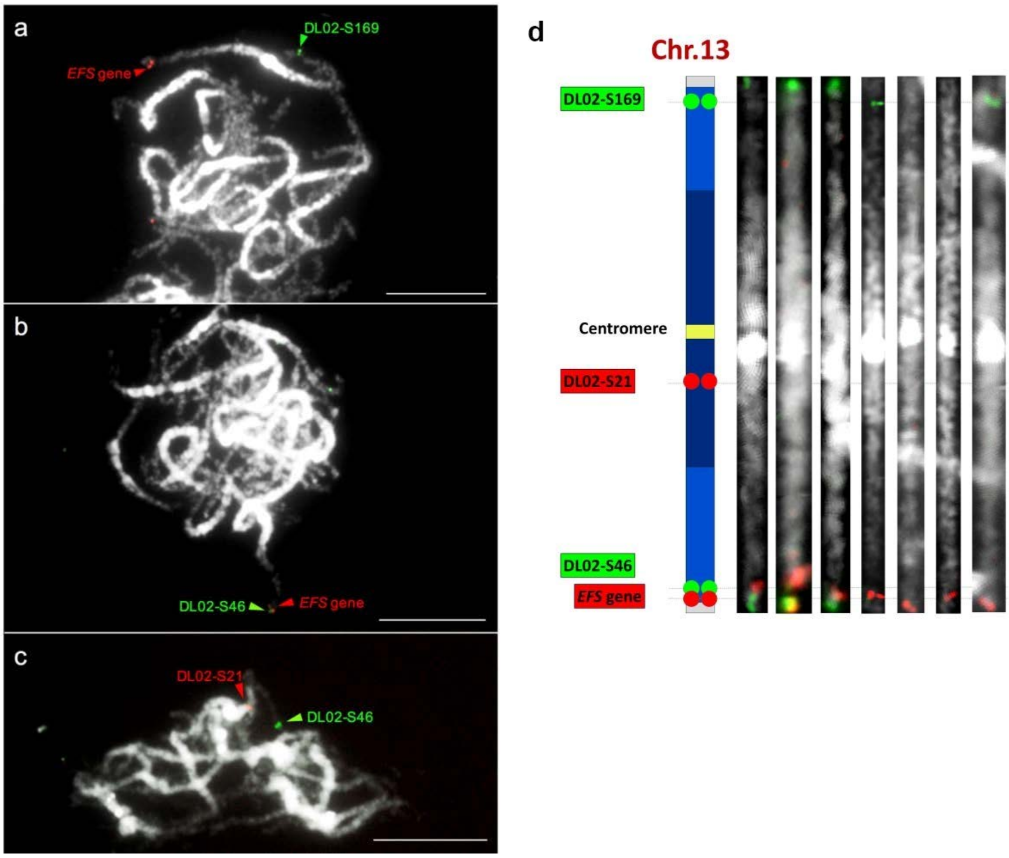

Figure S14 FISH mapping of linkage group-specific genomic probes on *P. aphrodite* chromosome 13. **(**a) FISH mapping of DL02-S169 probe (b) FISH mapping of DL02-S46 probe (c) FISH mapping of DL02-S21 and DL02-46 probes on the pachytene chromosome 13. The images of DAPI-stained chromosomes were converted to black and white. Scale bar = 10 µm. (d) Seven computationally straightened chromosome 13.

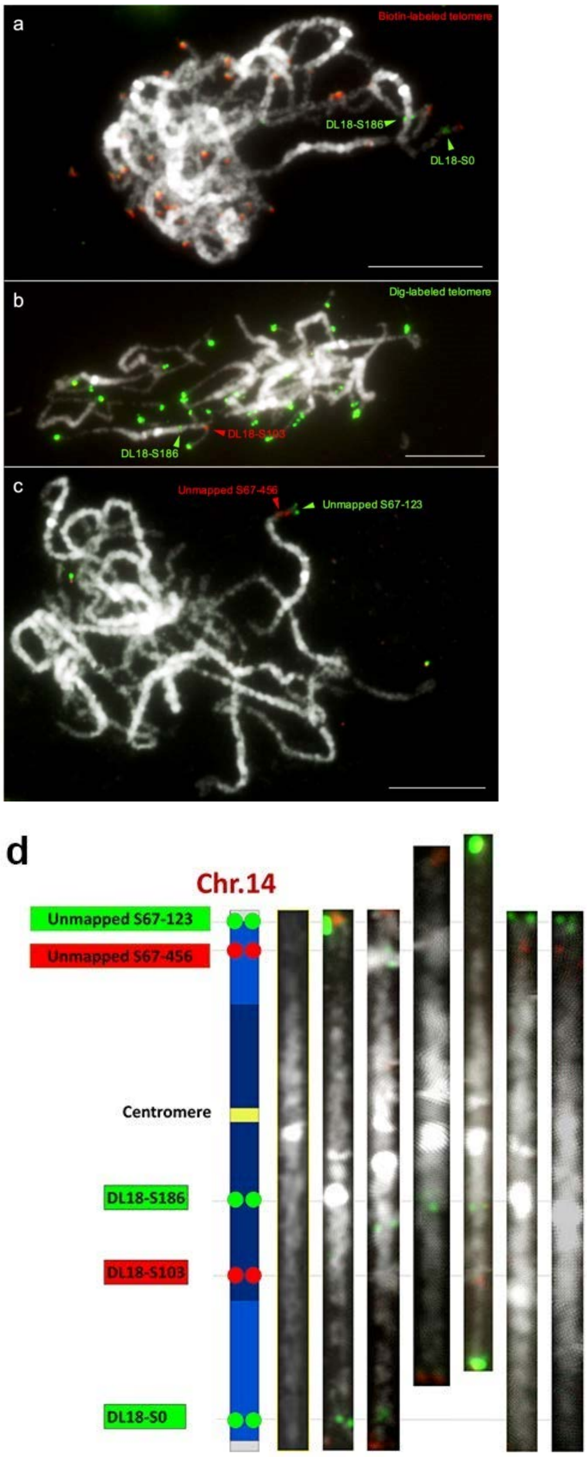

Figure S15 FISH mapping of linkage group-specific genomic probes on *P. aphrodite* chromosome 14. (a) FISH mapping of DL18-S186 and DL18-S0 probes (b) FISH mapping of DL18-S103 and DL18-S186 probes (c) FISH mapping of unmapped S67-456 and unmapped S67-123 probes on the pachytene chromosome 14. The images of DAPI-stained chromosomes were converted to black and white. Scale bar = 10 μm. (d) Seven computationally straightened chromosome 14.

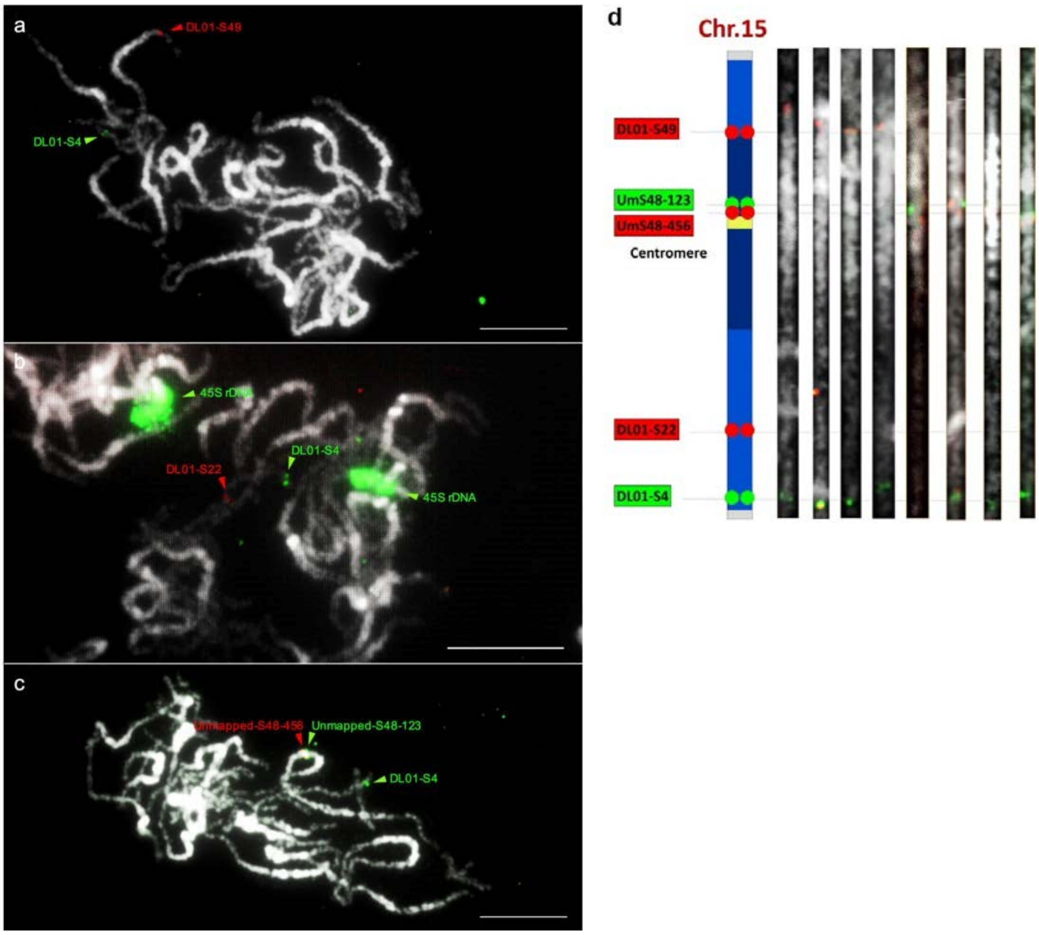

Figure S16 FISH mapping of linkage group-specific genomic probes on *P. aphrodite* chromosome 15. **(**a) FISH mapping of DL01-S49 and DL01-S4 probes (b) FISH mapping of DL01-S22 and DL01-S4 probes (c) FISH mapping of DL01S4, unmapped S48-456 and unmapped S48-123 probes on the pachytene chromosome 15. The images of DAPI-stained chromosomes were converted to black and white. Scale bar = 10 µm. (d) Eight computationally straightened chromosome 15.

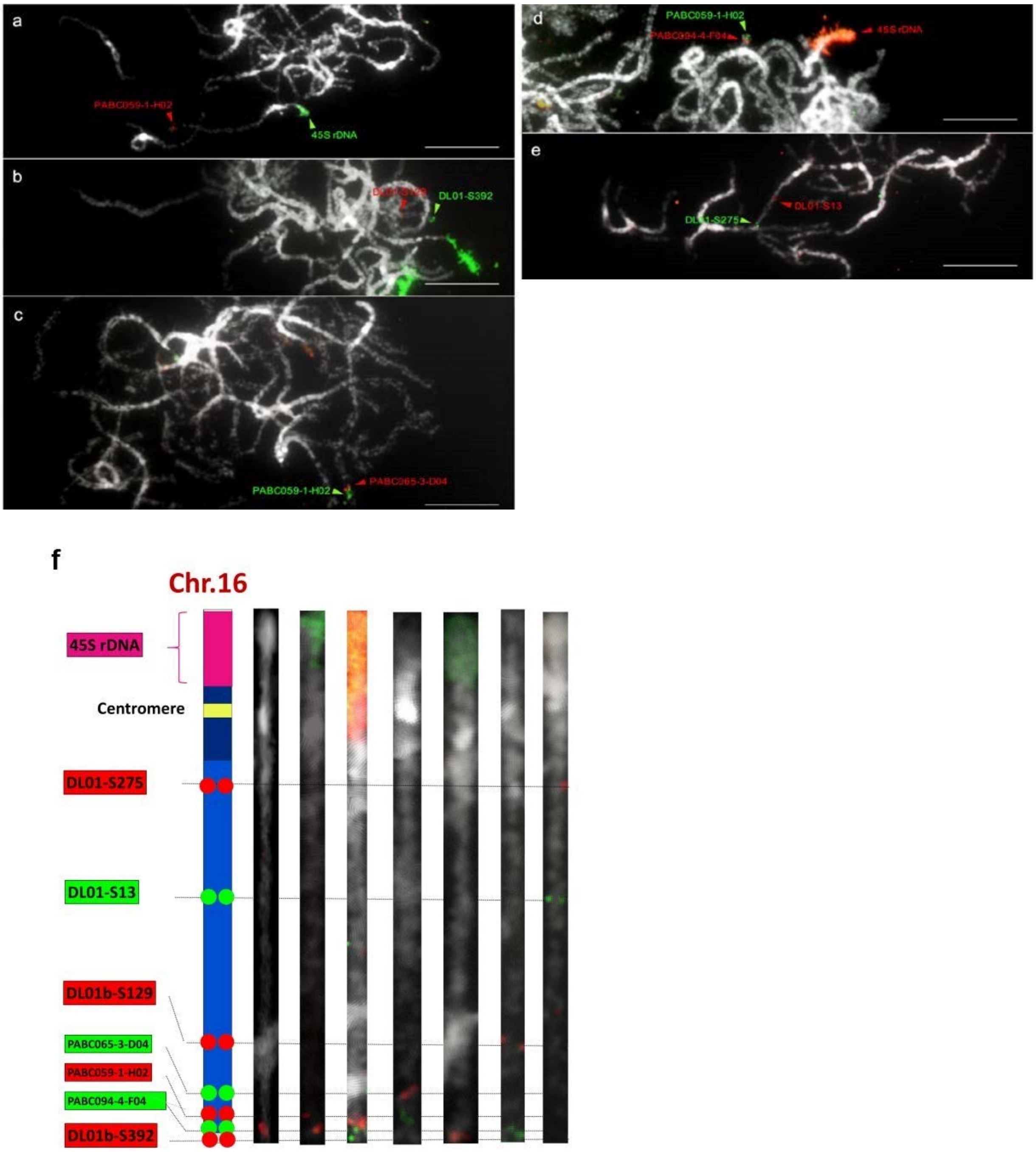

Figure S17 FISH mapping of linkage group-specific genomic probes on *P. aphrodite* chromosome 16. (a) FISH mapping of PABC059-1-H02 probe (b) FISH mapping of DL01-S129 and DL01-S392 probes (c) FISH mapping of PABC059-1-H02 and PABC065-3-D04 probes (d) FISH mapping of PABC059-H02 and PABC094-4-F04 probes (e) FISH mapping of DL01S275 and DL01-S13 probes on the pachytene chromosome 16. The images of DAPI-stained chromosomes were converted to black and white. Scale bar = 10 μm. (f) Seven computationally straightened chromosome 16.

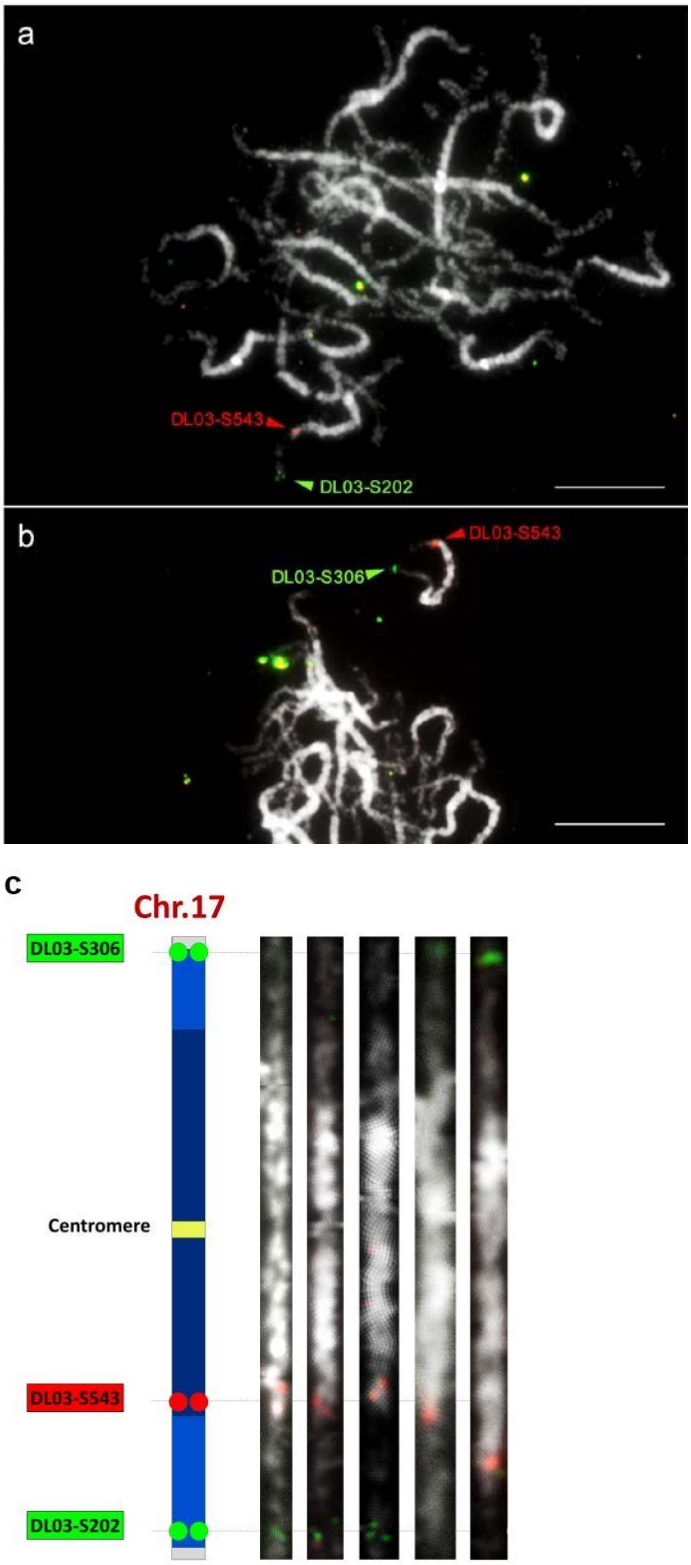

Figure S18 FISH mapping of linkage group-specific genomic probes on *P. aphrodite* chromosome 17. **(**a) FISH mapping of DL03-S543 and DL03-S202 probes (b) FISH mapping of DL03-S306 and DL03-S543 probes on the pachytene chromosome 17. The images of DAPI-stained chromosomes were converted to black and white. Scale bar = 10 μm. (c) Five computationally straightened chromosome 17.

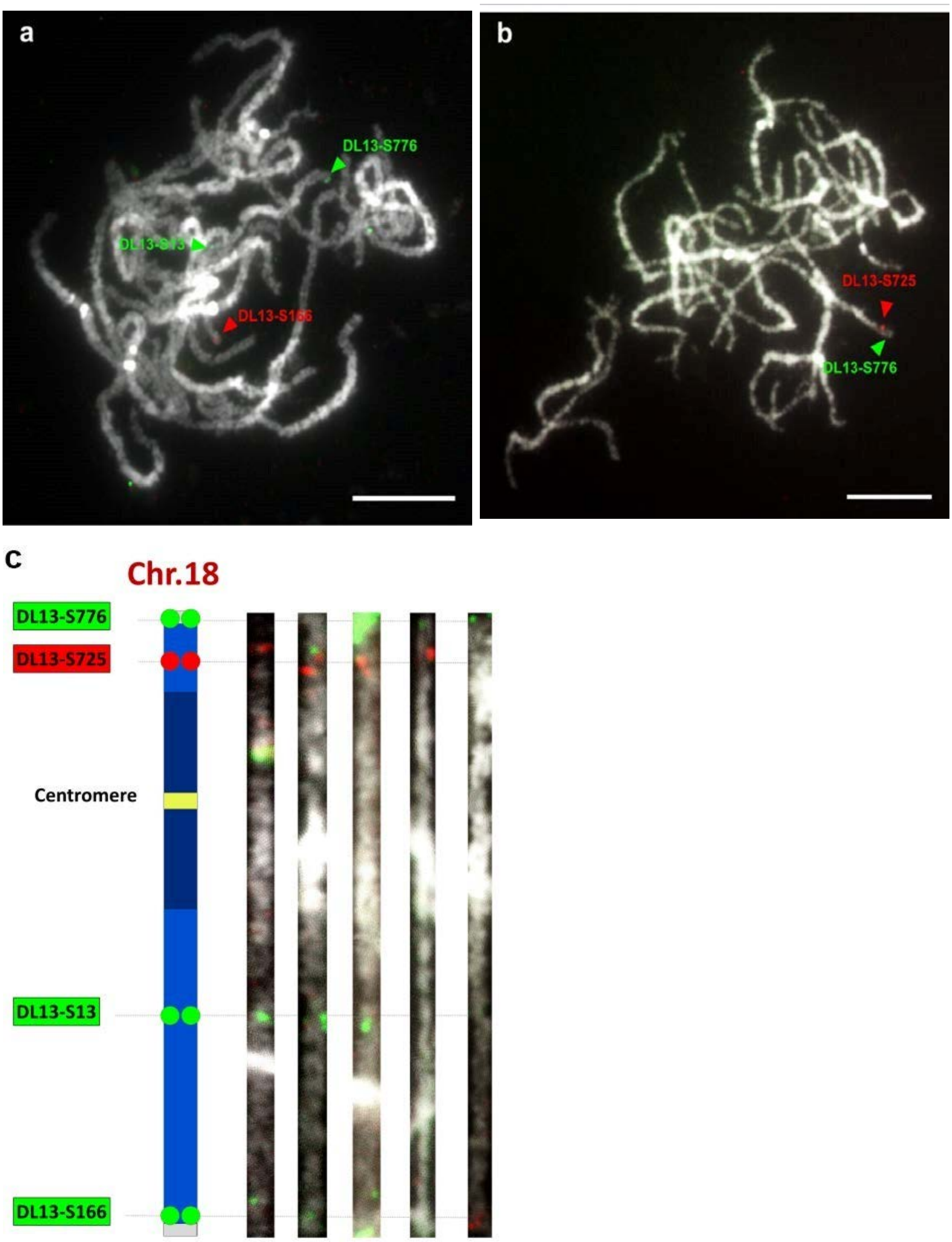

Figure S19 FISH mapping of linkage group-specific genomic probes on *P. aphrodite* chromosome 18. **(**a) FISH mapping of DL13-S13, DL13-S776 and DL13-S166 probes (b) FISH mapping of DL13-S725 and DL13-S776 probes on the pachytene chromosome 18. The images of DAPI-stained chromosomes were converted to black and white. Scale bar = 10 μm. (c) Five computationally straightened chromosome 18.

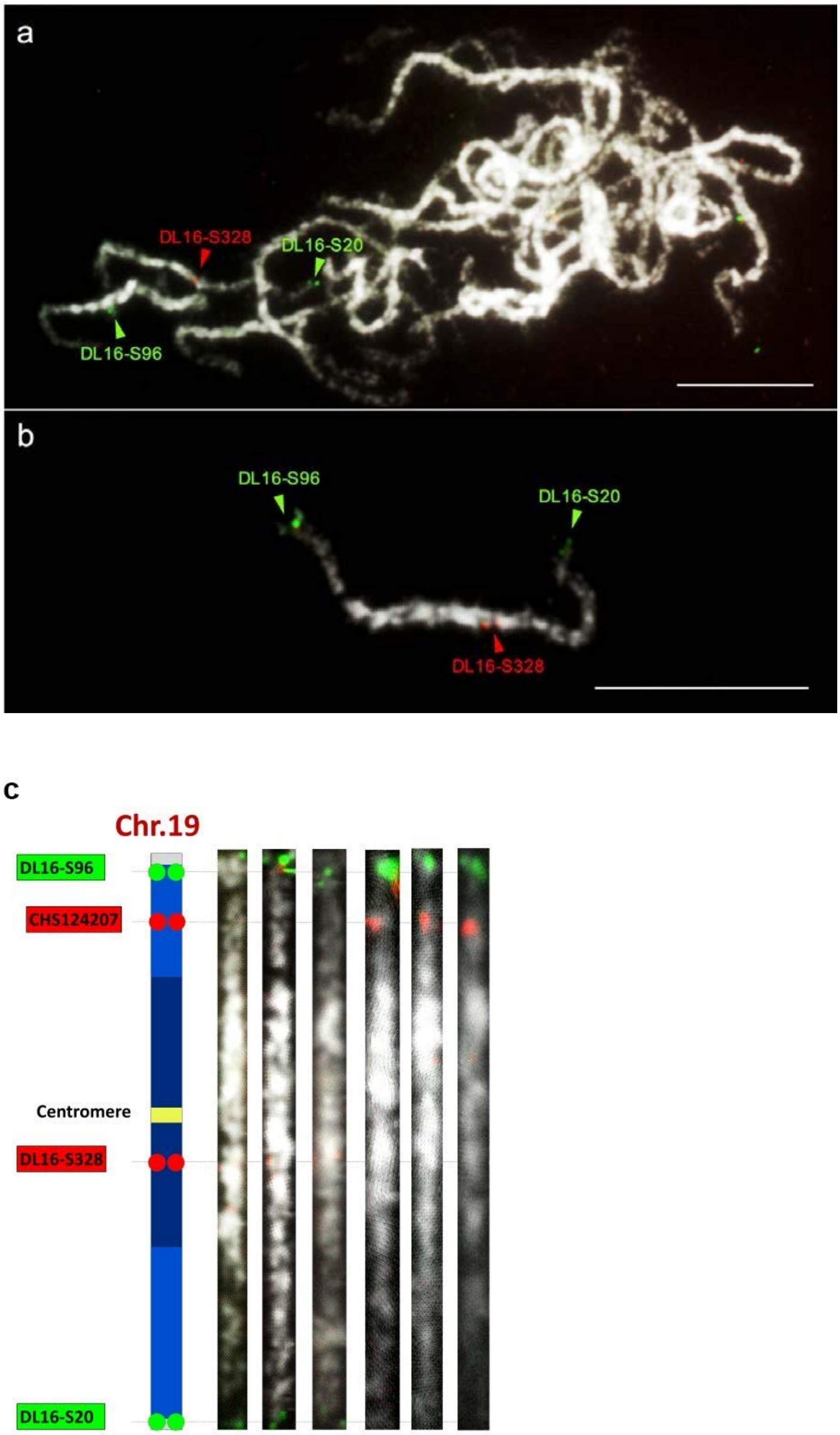

Figure S20 FISH mapping of linkage group-specific genomic probes on *P. aphrodite* chromosome 19. **(**a) FISH mapping of DL16-S96, DL16-S328 and DL16-S20 probes (b) FISH mapping of DL16-S96, DL16-S328 and DL16-S20 probes on the pachytene chromosome 19. Scale bar = 10 µm. (c) Six computationally straightened chromosome 19.

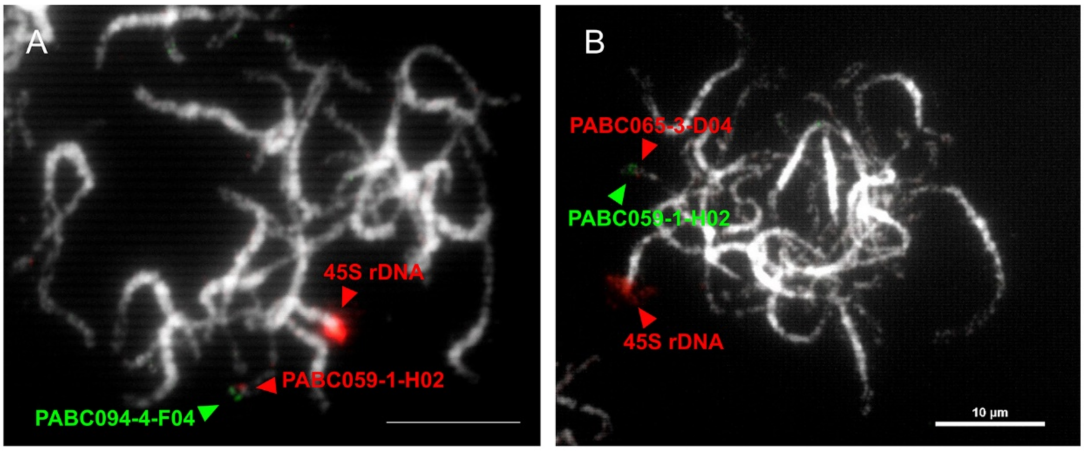

Figure S21 FISH mapping of LG16-specific genomic probes and 45S rDNA on *P. equestris* chromosome. (A) FISH mapping of PABC094-4-F04, PABC059-1-H02 and 45S rDNA probes (B) FISH mapping of PABC059-1-H02, PABC065-3-D04 and 45S rDNA probes.

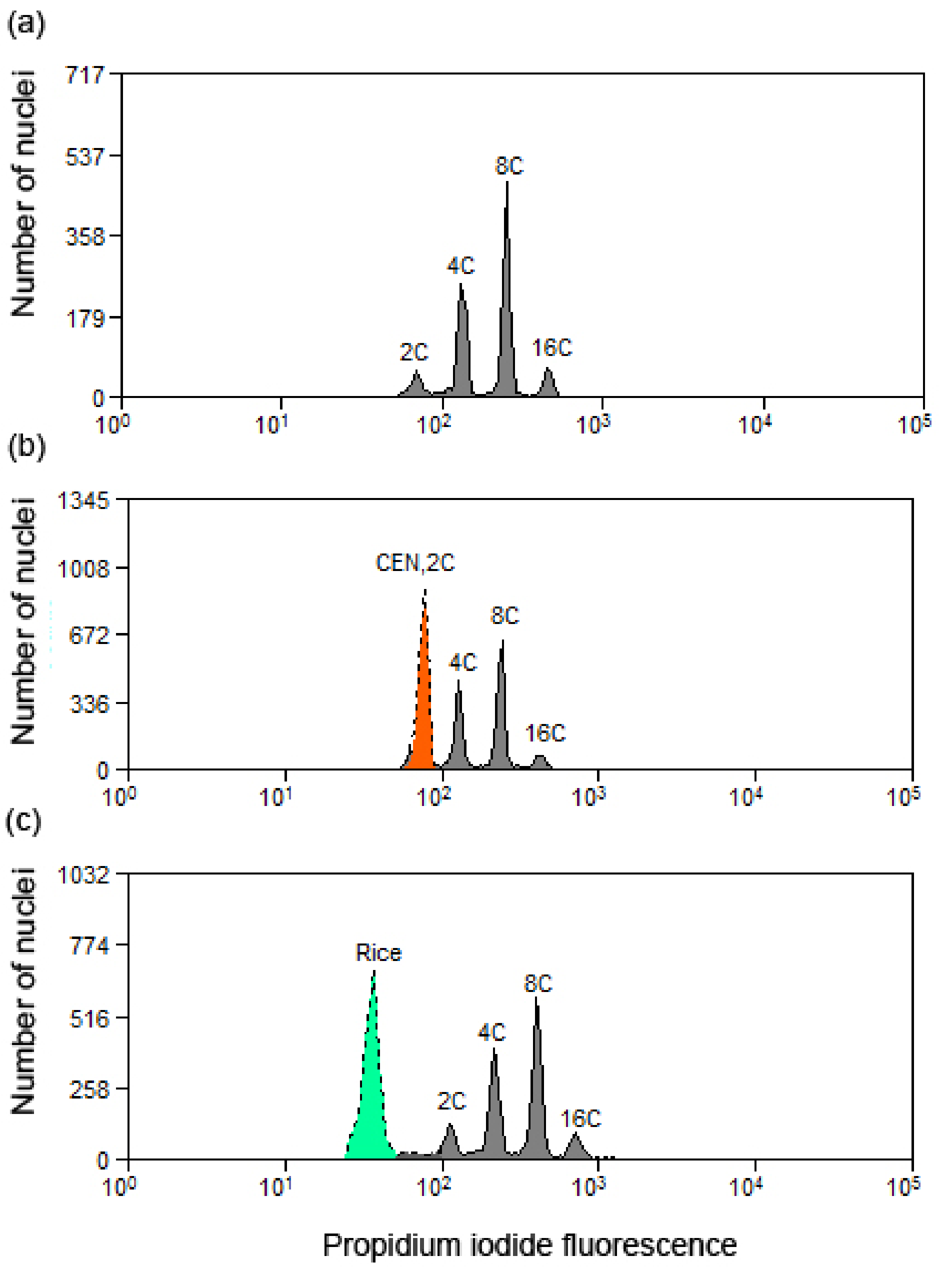

Figure S22 Flow cytometry analysis. (a) the histograms of flow cytometry of *P. aphrodite*, (b) *P. aphrodite* (gray color) mixed with Chicken Erythrocyte Nuclei (orange color), (c) *P. aphrodite* (gray color) mixed with Rice TN67 (green color).

Figure S23 Locations of recombination hotspots along each chromosome. The recombination hotspots (>55 cM/mb) are indicated by red dashed lines and the corresponding recombination rates. The cumulative genetic distance from the end of short arm to the end of long arm is shown.

Chromosome 1

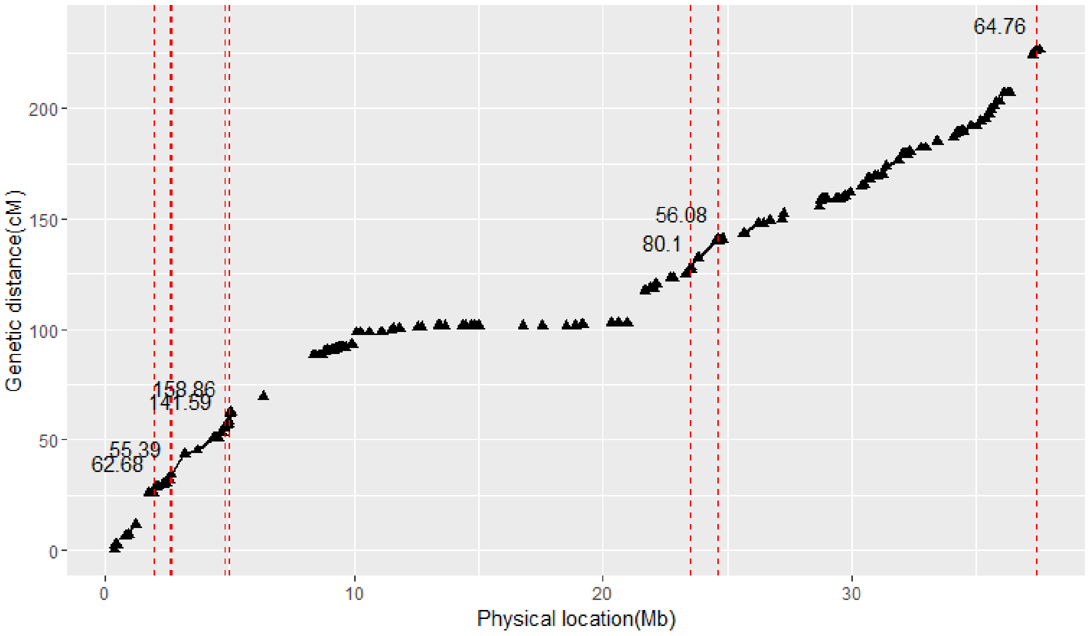

Chromosome 2

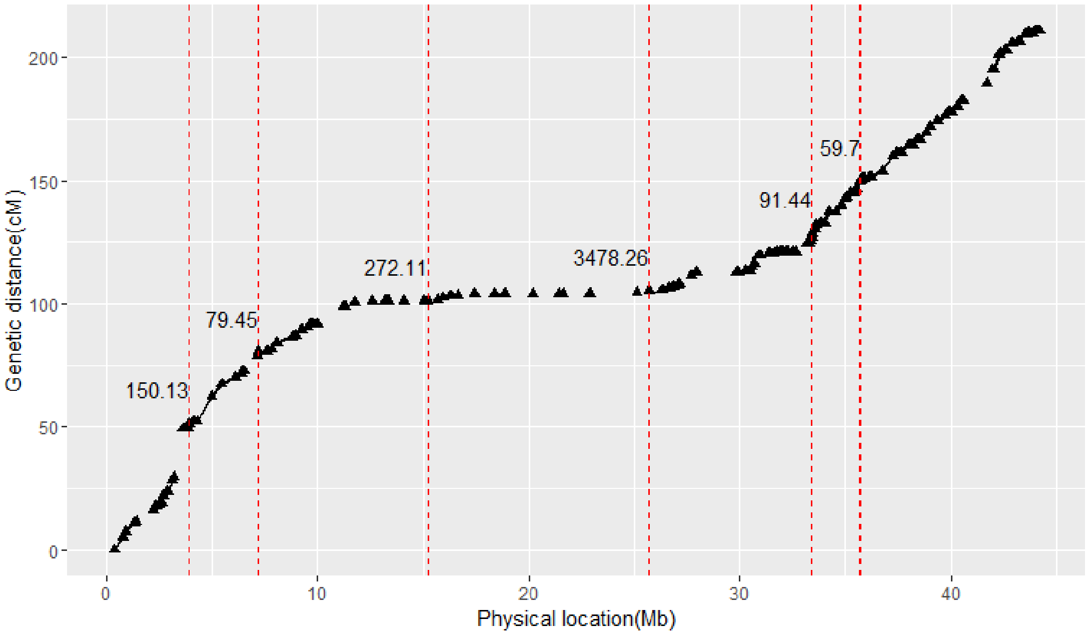

Chromosome 3

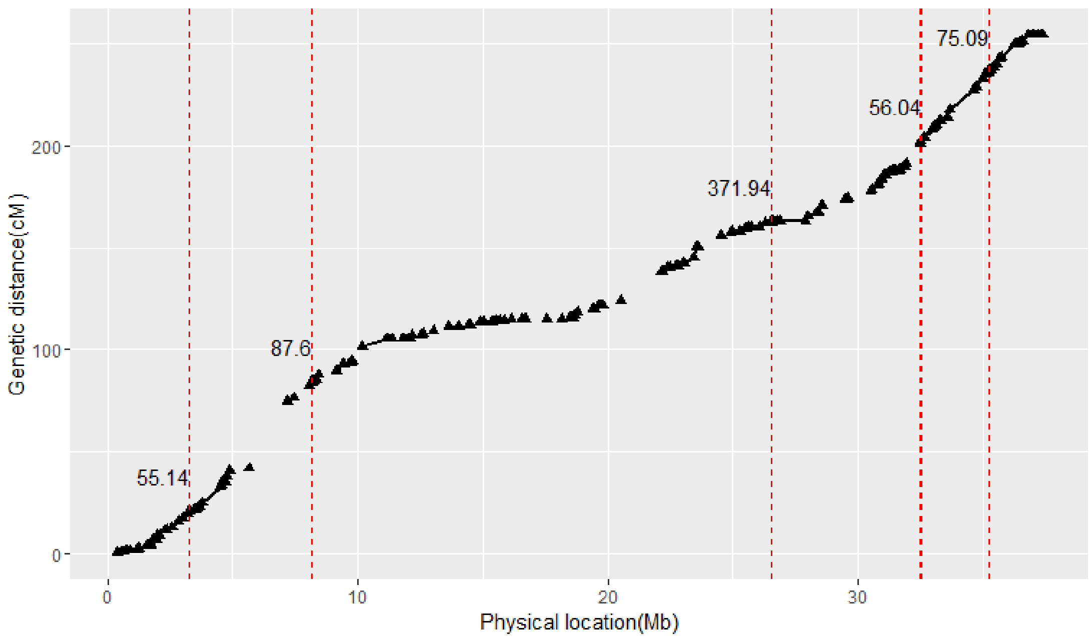

Chromosome 4

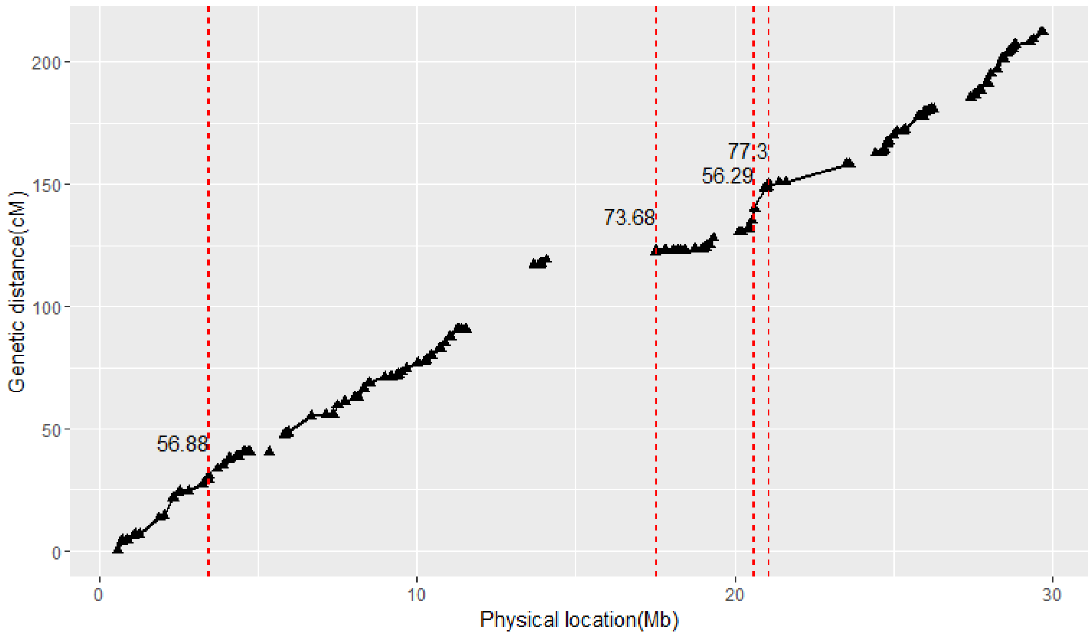

Chromosome 5, linkage group 5a

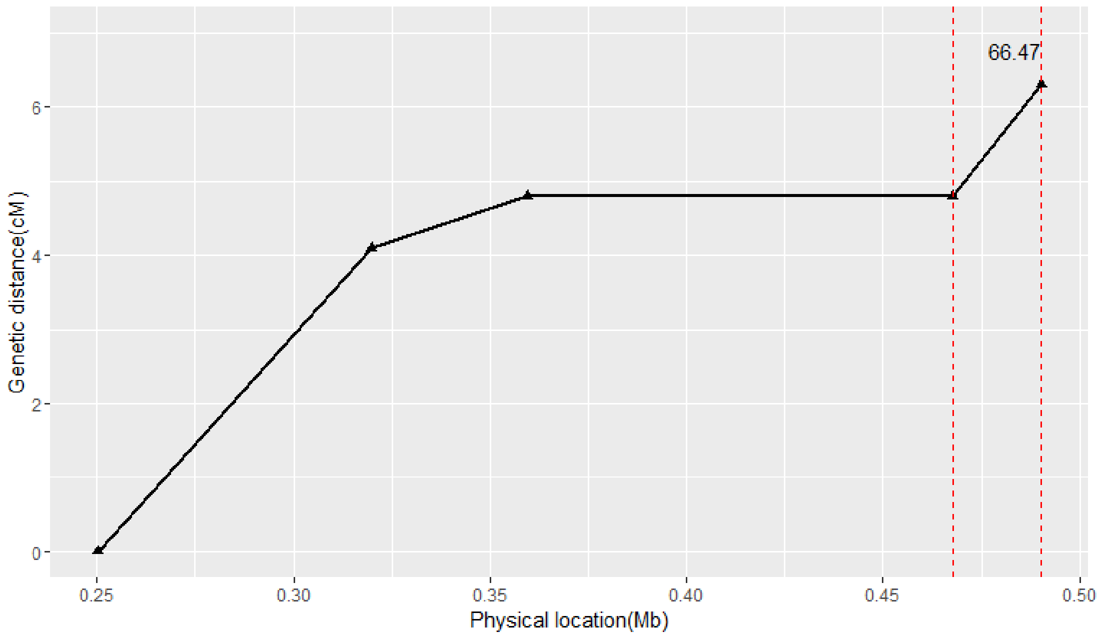

Chromosome 5, linkage group 5b

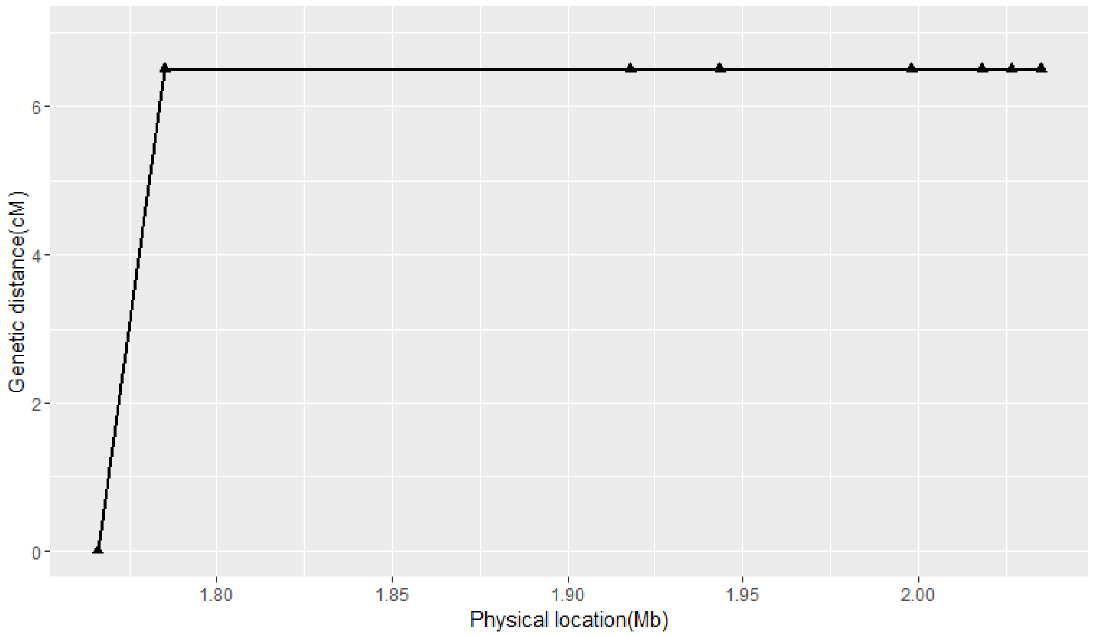

Chromosome 6

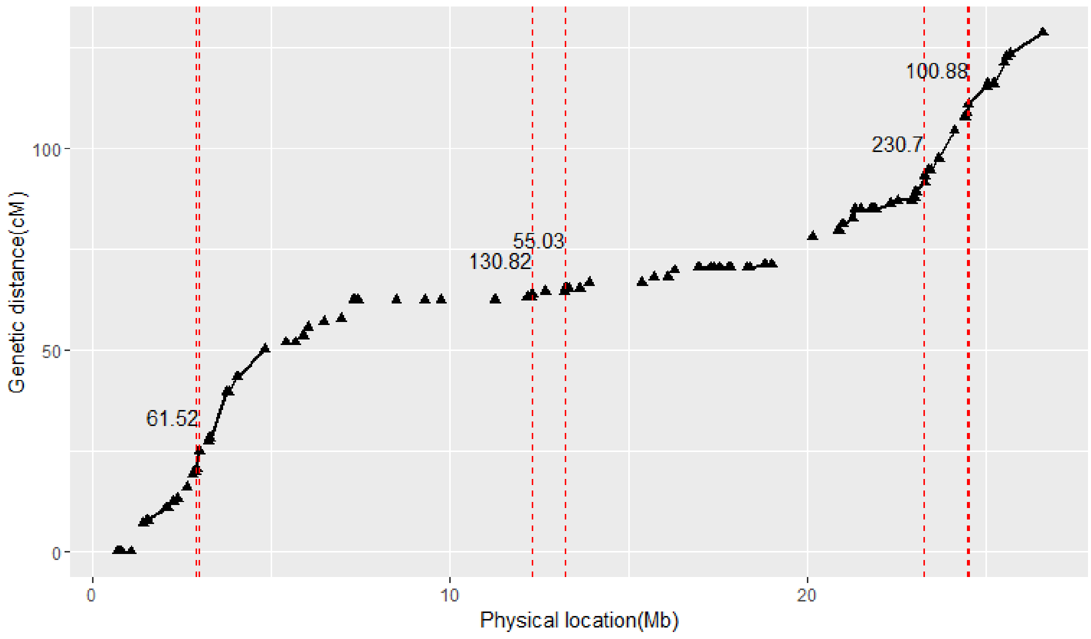

Chromosome 7

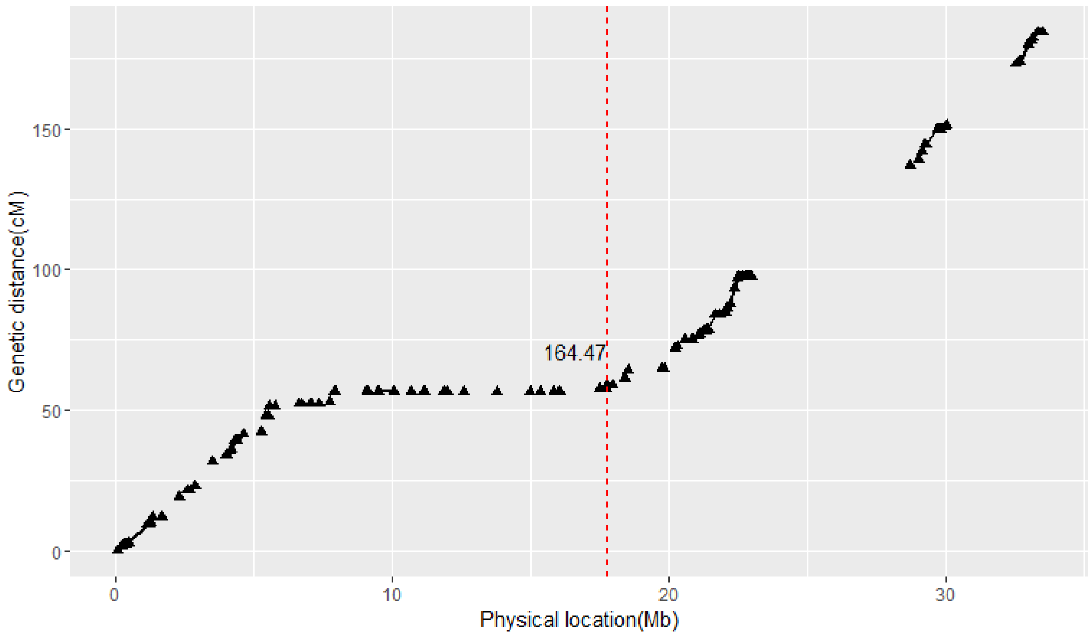

Chromosome 8, linkage group 8a

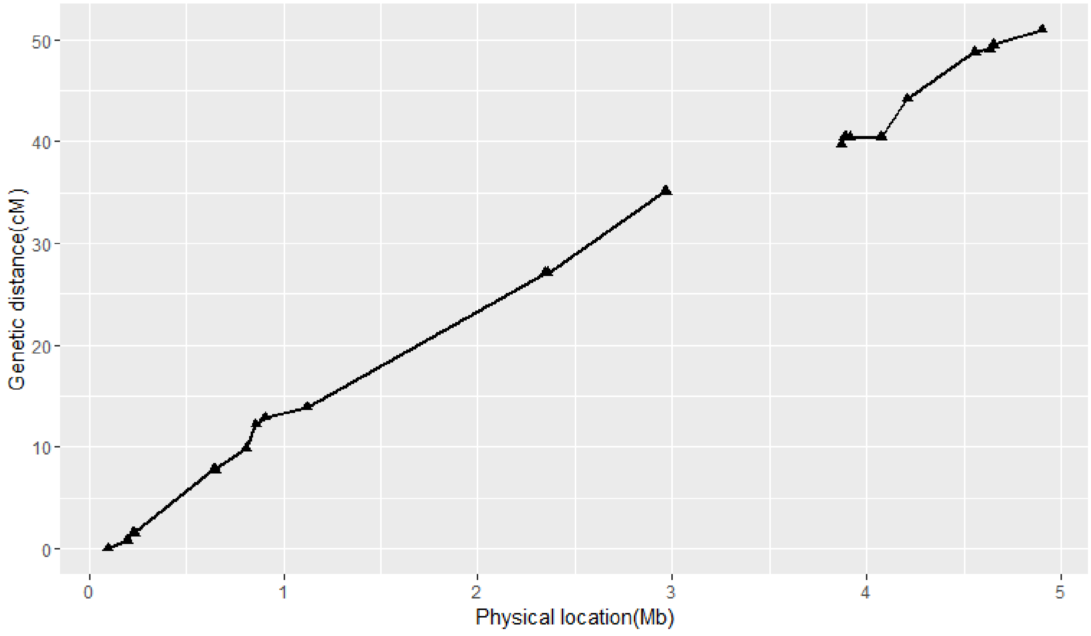

Chromosome 8, linkage group 8b

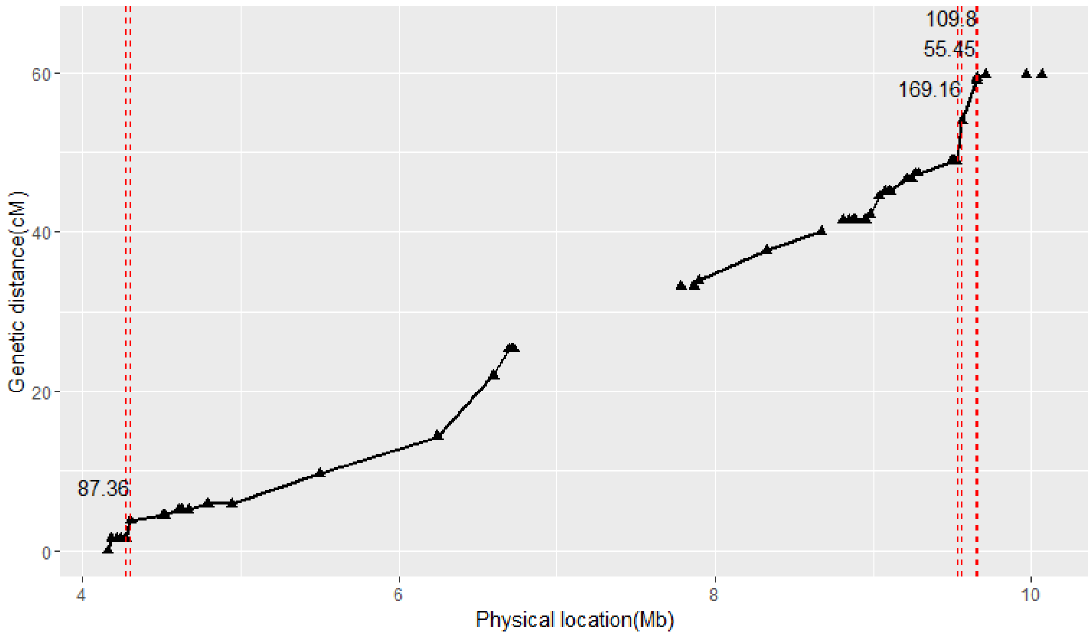

Chromosome 9

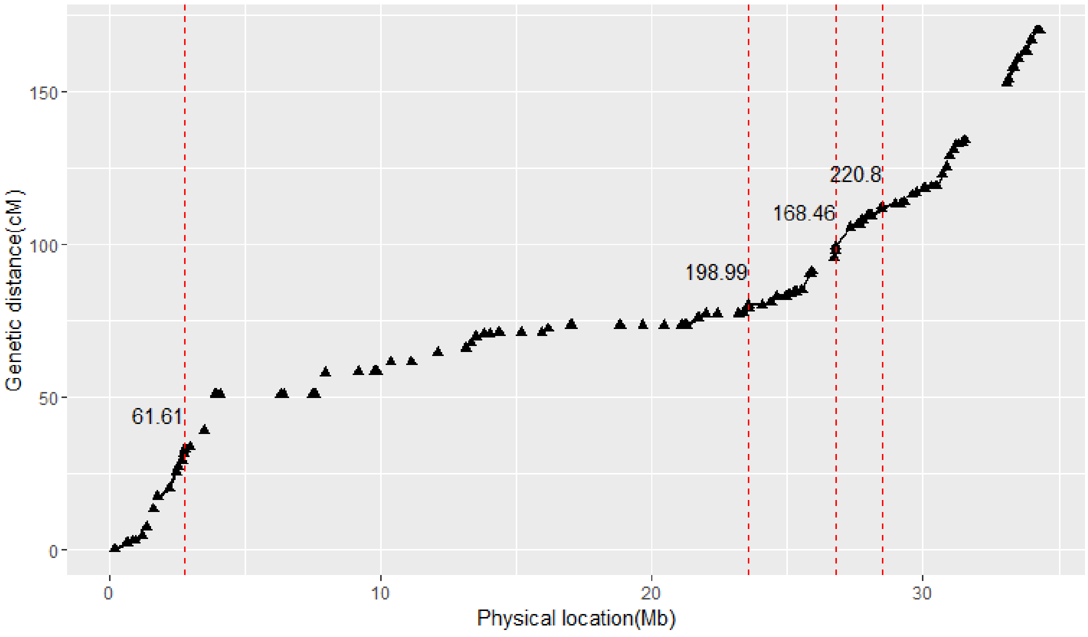

Chromosome 10, linkage group 10a

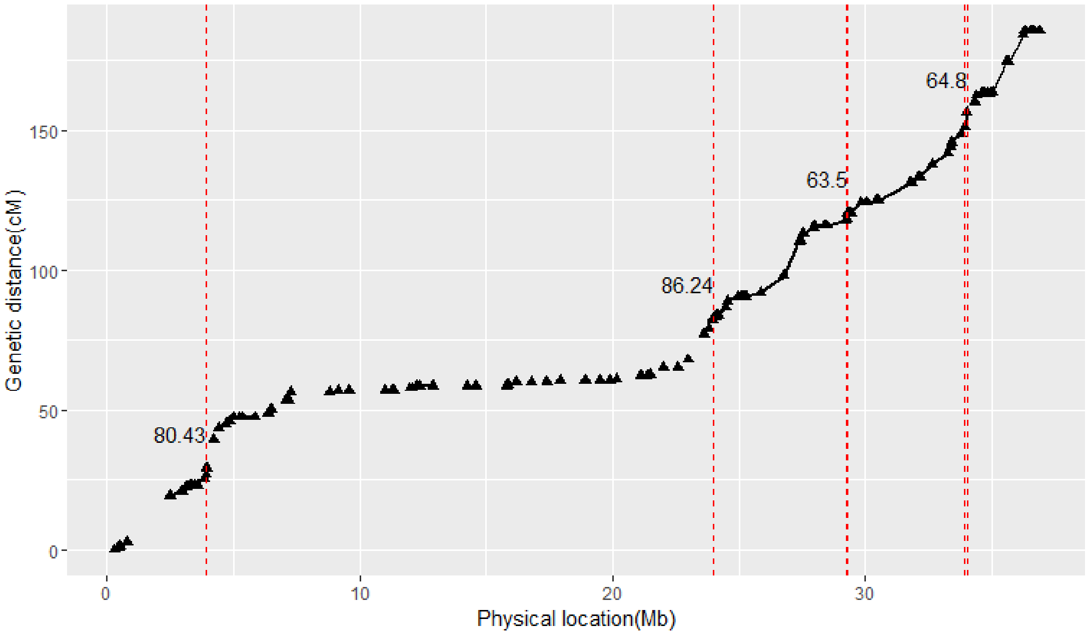

Chromosome 10, linkage group 10b

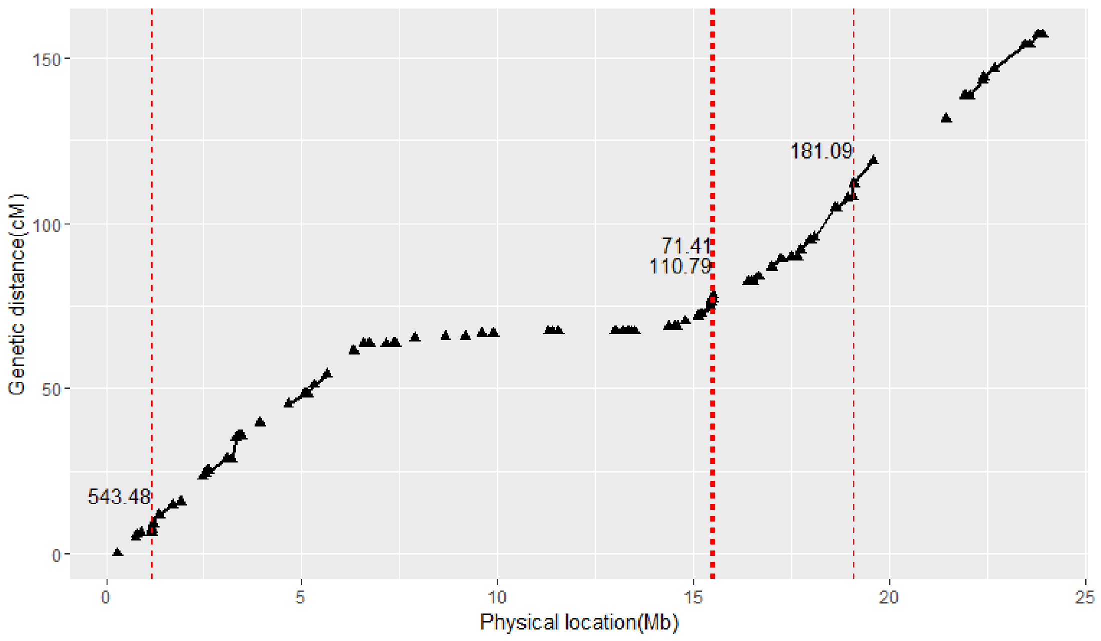

Chromosome 11

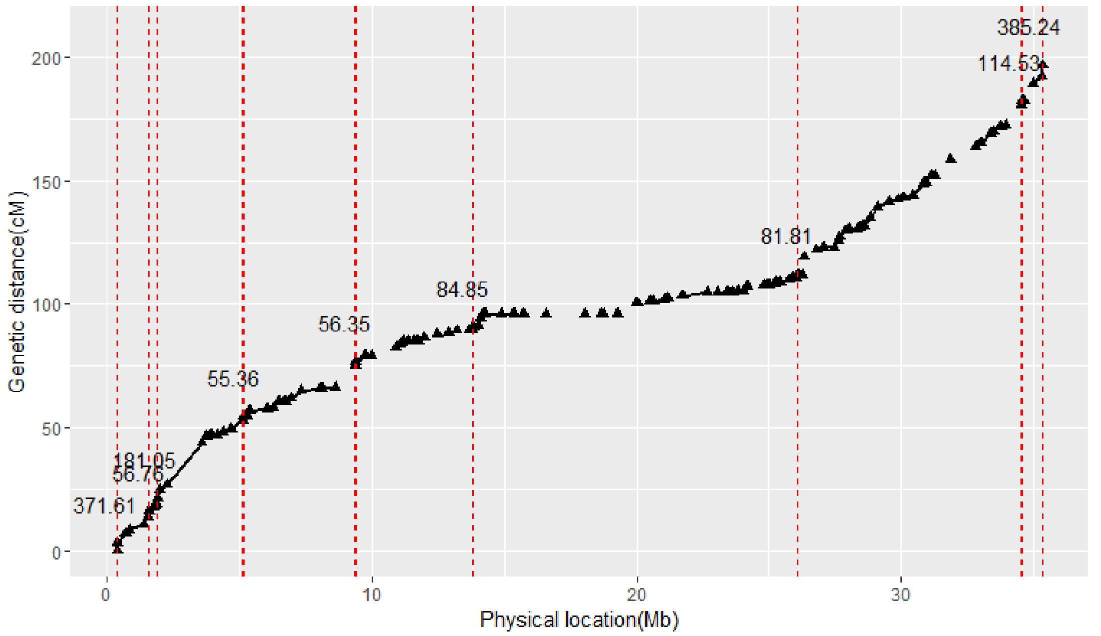

Chromosome 12

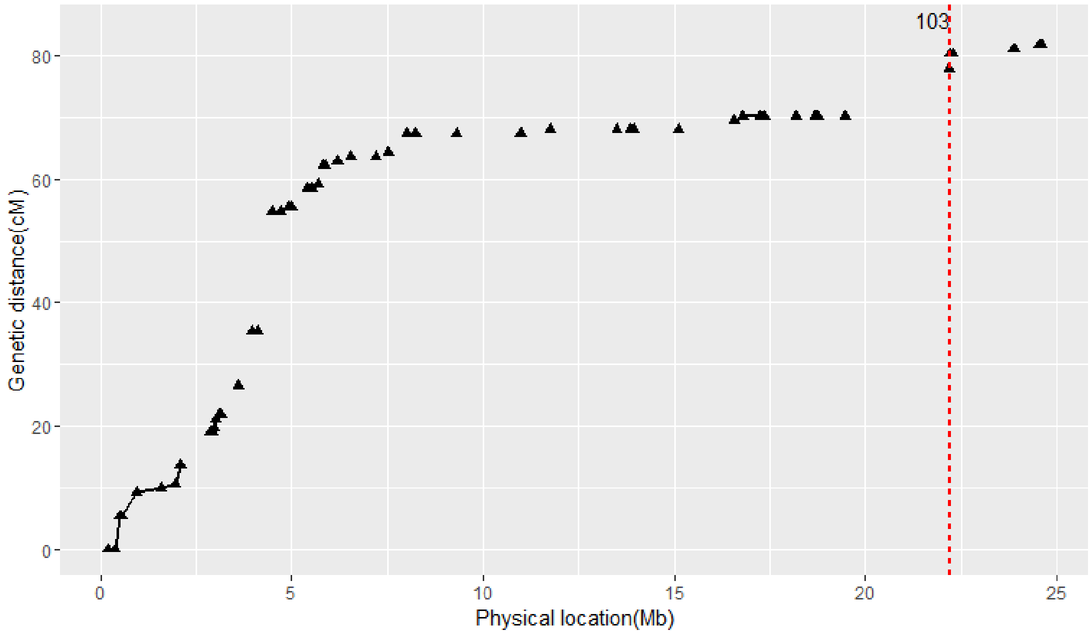

Chromosome 13

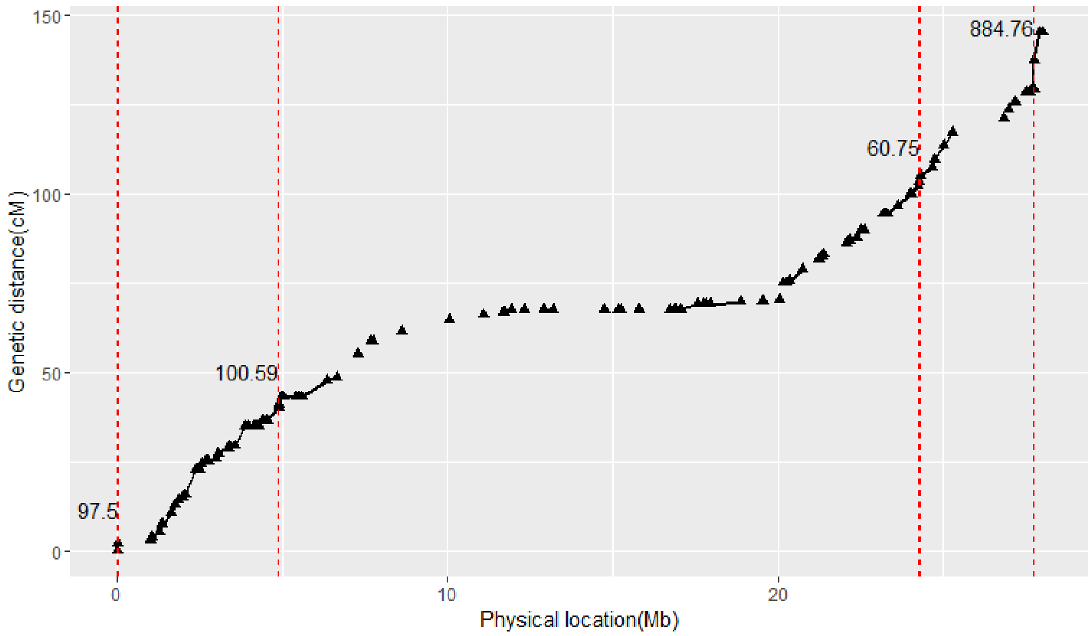

Chromosome 14

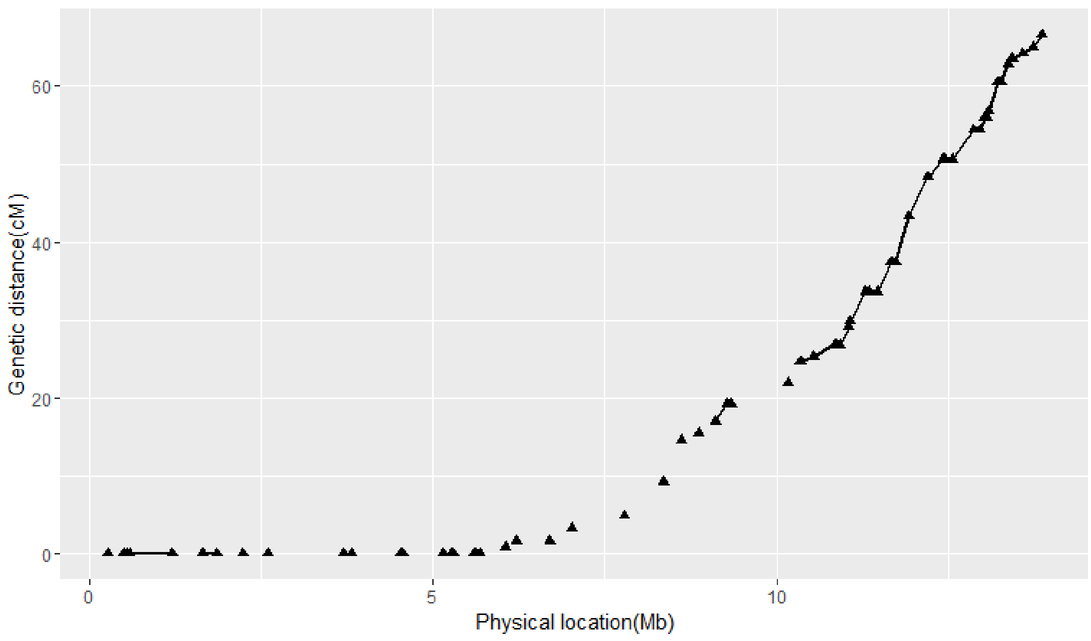

Chromosome 15

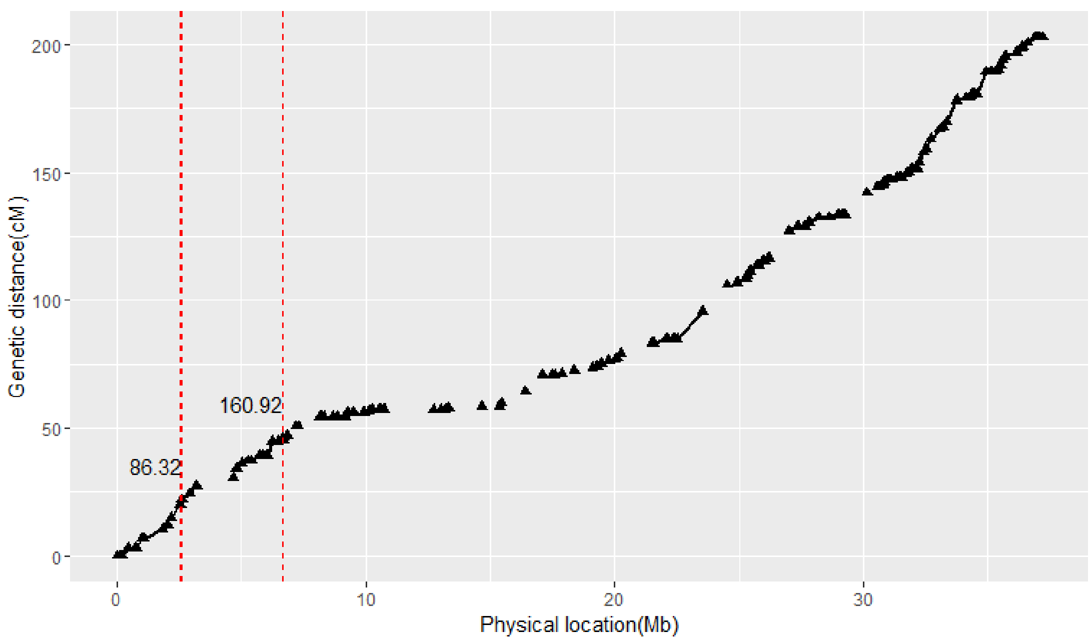

Chromosome 16

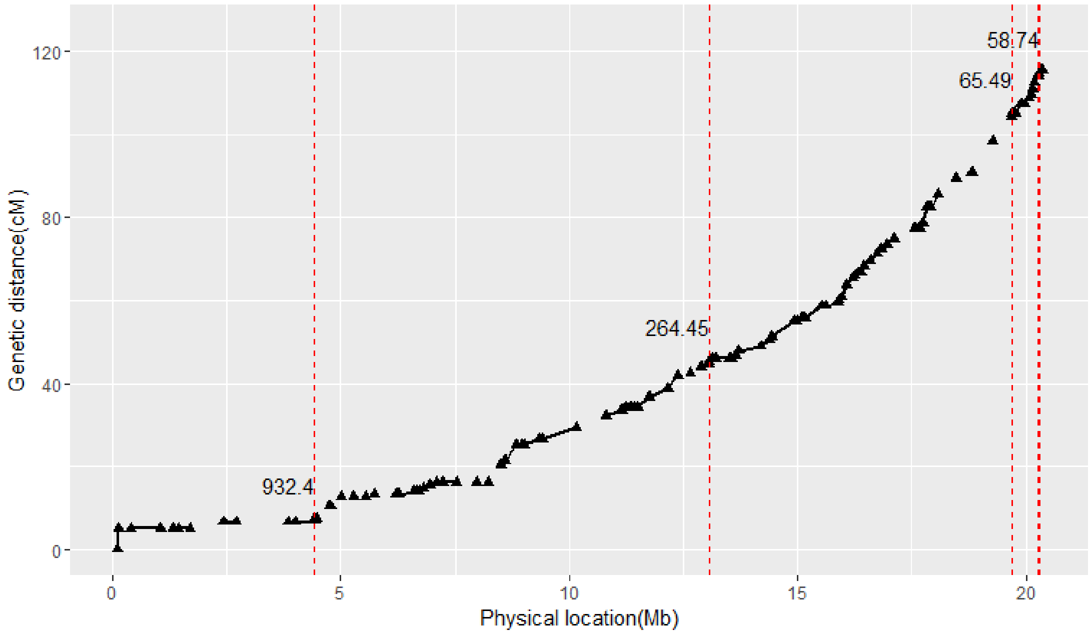

Chromosome 17

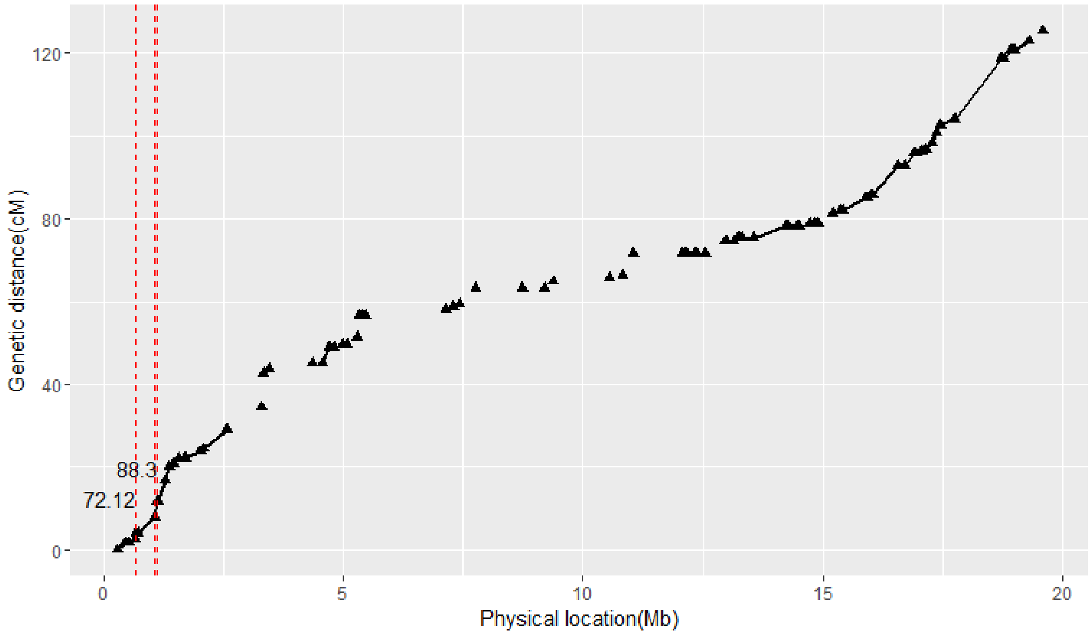

Chromosome 18

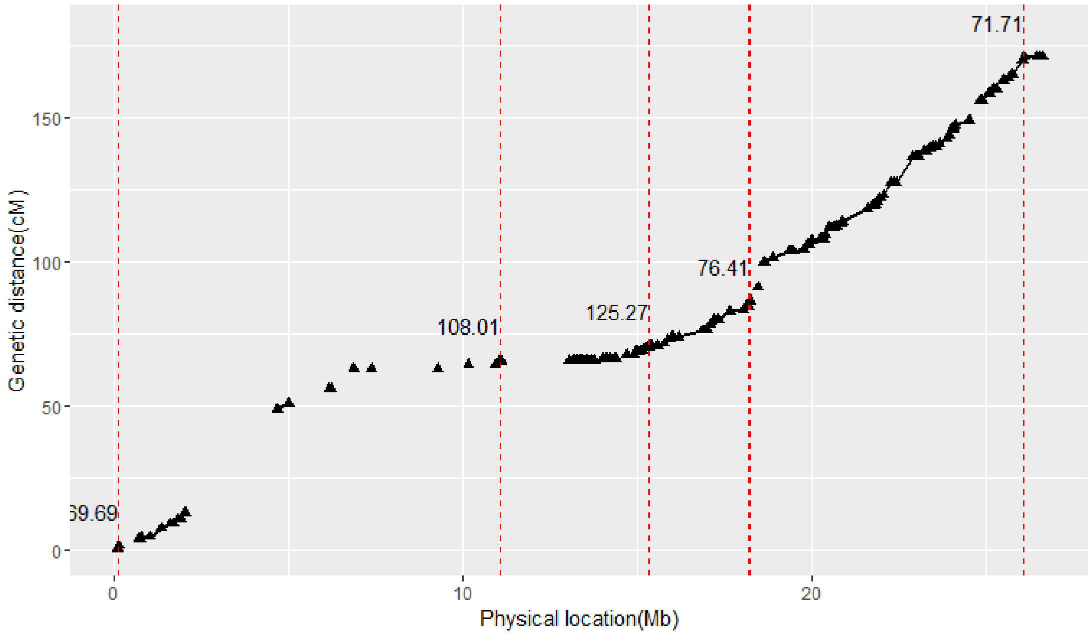

Chromosome 19

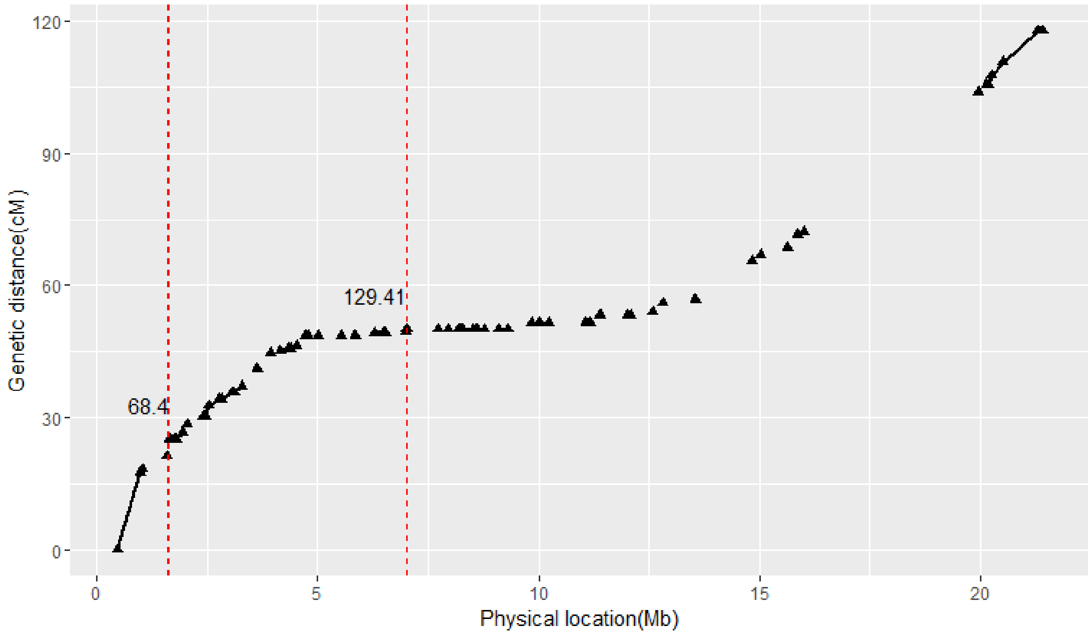

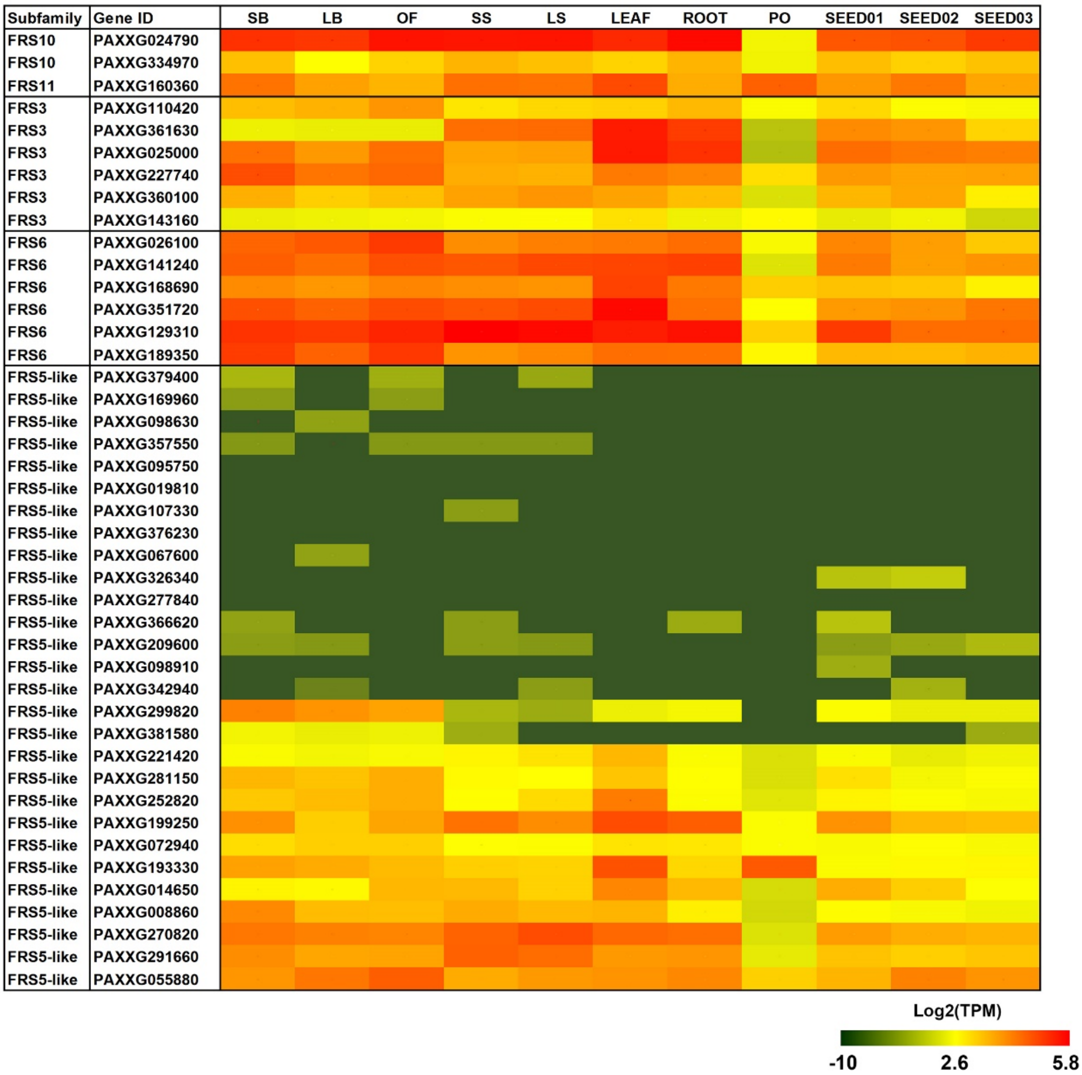

Figure S24 Gene expression heatmap for the full-length *FAR1*/*FRS* genes.

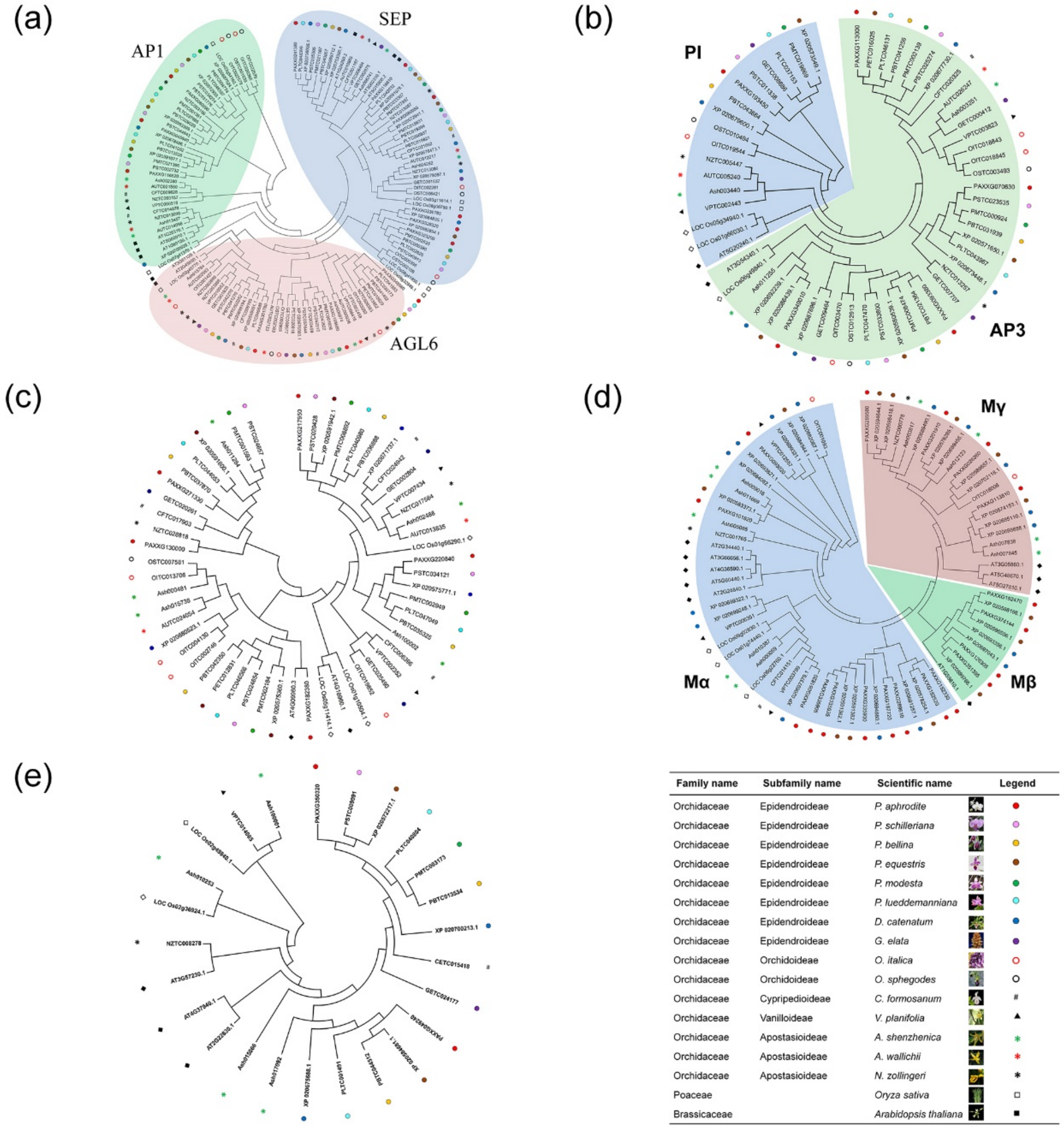

Figure S25 Phylogenetic trees of the MADS-box gene family in orchids. (a) Phylogenetic analysis of class A and E genes, *AP1*, *AGL6* and *SEP*, including 134 amino acid sequences from 17 species. (b) Phylogenetic analysis of class B genes, *PI* and *AP3*, including 57 amino acid sequences from 17 species. (c) Phylogenetic analysis of *AGL* genes, including 55 amino acid sequences from 16 species. (d) Phylogenetic analysis of type I MADS-box genes involved 10 species contain 76 amino acid sequences. (e) Phylogenetic analysis of *ANR* genes, including 25 amino acid sequences from 14 species.

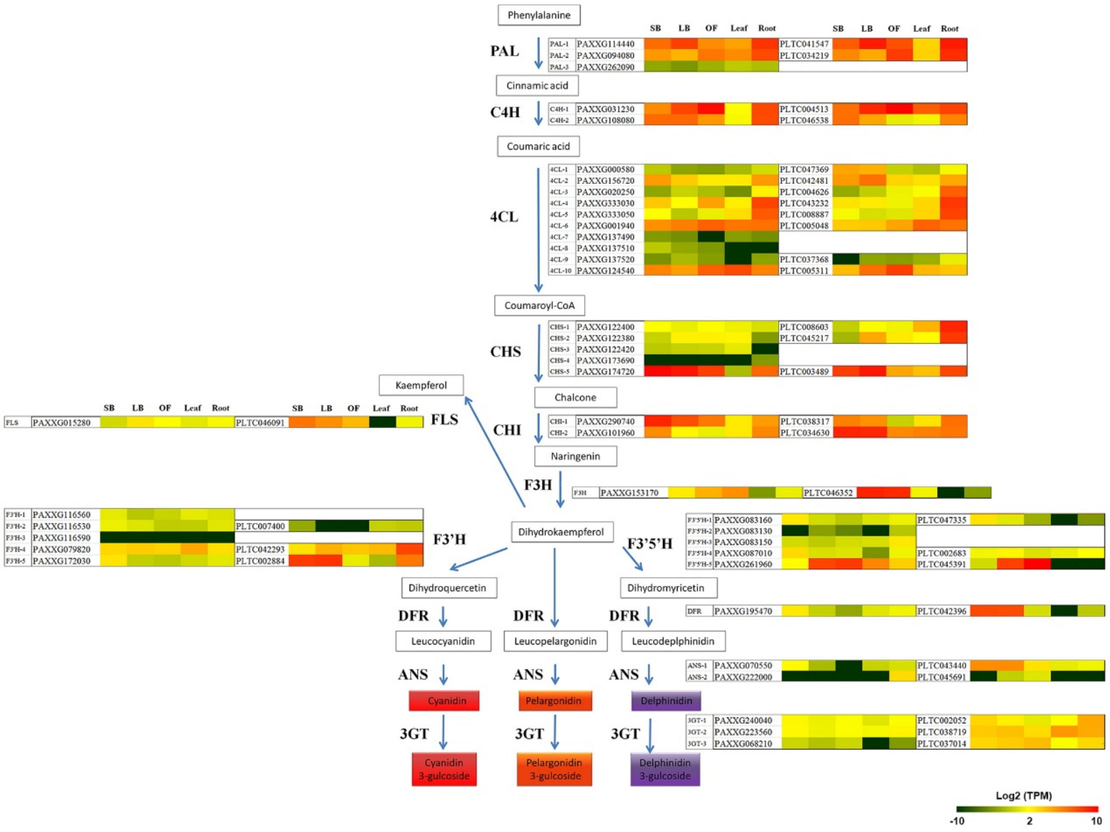

Figure S26 Expression of flavonoid biosynthetic pathway genes in *P. aphrodite* and *P. lueddemanniana*. Gene expression heatmap (TPM on log2 scale) of orthologous gene pairs: *P. aphrodite* and *P. lueddemanniana* genes are shown on the left and right, respectively. LB, large bud; SB, small bud; OF, Open flower. The enzymes involved are indicated next to the blue arrows and names are abbreviated as follows: PAL, Phe ammonia-lyase; C4H, cinnamate-4-hydroxylase; 4CL, 4-coumaroyl:CoA-ligase; CHS, chalcone synthase; CHI, chalcone isomerase; F3H, flavanone 3-hydroxylase; FLS, flavonol synthase; F3' H, flavonoid 3' hydroxylase; F3'5' H, flavonoid 3'5' hydroxylase; DFR, dihydroflavonol 4-reductase; ANS, Anthocyanidin synthase; 3GT, UDP-glucose:flavonoid 3-O-glucosyltransferase.

Supporting Tables

Table S1 Metrics for sequencing data used in SOAPdenovo2 assembly

Library type	Physical size	Raw read length (bp)	Sequencing platform	Number of reads (millions)	Total bases (Gb)	Sequence coverage
Paired-end	202 bp	120	GAIIX	151.5	15.9	13.2
	228 bp	120	GAIIX	133.3	13.9	11.5
	255 bp	120	GAIIX	219.1	23.8	19.9
	278 bp	120	GAIIX	222.8	22.9	19.1
	301 bp	120	GAIIX	195.9	21.4	17.8
	342 bp	120	GAIIX	332.0	34.4	28.7
	378 bp	120	GAIIX	314.4	32.1	26.7
	412 bp	120	GAIIX	264.0	28.5	23.7
	445 bp	120	GAIIX	160.5	16.8	14.0
	478 bp	120	GAIIX	148.8	15.6	13.0
Mate pair	3 kb	80	GAIIX	500.3	39.2	32.6
		100	HiSeq	232.3	23.0	19.2
	5 kb	200	HiSeq	357.1	45.4	37.8
	8 kb	200	HiSeq	326.1	41.0	34.2
Fosmid	40 kb	250	MiSeq	13.7	1.9	1.5

Table S2 Metrics for sequencing data used in ALLPATHS-LG assembly and SSPACE scaffolding

Library type	Physical size	Raw read length (bp)	Sequencing platform	Number of reads (millions)	Total bases (Gb)	Sequence coverage
Fragments	150 bp	150	HiSeq	170.2	22.5	18.7
(paired-end)	230 bp	200	HiSeq	208.4	39.8	33.2
Jumps	3 kb	80	GAIIX	97.6	7.6	6.3
(mate pair)		100	HiSeq	63.5	6.2	5.2
	3 kb	200	HiSeq	143.5	18.8	15.7
	5 kb	200	HiSeq	128.0	16.3	13.6
	7 kb	200	HiSeq	126.1	16.3	13.6
(mate pair)	8 kb	200	HiSeq	323.8	36.1	30.1
	10 kb	200	HiSeq	222.3	21.7	18.1
	15 kb	200	HiSeq	4.5	0.5	0.4
(fosmid)	40 kb	250	MiSeq	13.7	1.9	1.5

Table S3 Quality control metrics for the SOAPdenovo2 assembly

	Number of Contig/ Scaffolds	Average sequence length (kb)	Max sequence length (kb)	N10 (kb)	N50 (kb)	N90 (kb)	Total sequence length (Mb)
Contigs	1202535	0.86	52.85	11.07	1.84	0.30	
Scaffolds	67500	19.75	2253.18	716.22	187.75	9.77	1333.44

Table S4 Quality control metrics for the ALLPATHS-LG assembly and SSPACE scaffolding

	Number of Contig/ Scaffolds	Average sequence length (kb)	Max sequence length (kb)	N10 (kb)	N50 (kb)	N90 (kb)	Total sequence length (Mb)
Allpaths-LG de novo assembly							
Contigs	108046	8.38	271.16	58.29	18.81	3.39	
Scaffolds	20848	47.15	2777.24	1323.60	417.48	34.91	983.00
SSPACE scaffolding of Allpaths-LG assemblies and curation							
Scaffolds	13732	74.65	10391.94	3839.00	946.43	69.36	1025.10

Table S11 Orchidstra 2.0 ESTs that map to *P. aphrodite* genome

Subfamily	Species	No. of protein-coding transcripts	No. of mapped sequences (E-value <1e-10)	Mapping rate (%)
Apostasioideae	*Apostasia wallichii*	26259	23557	89.7
Apostasioideae	*Neuwiedia zollingeri*	27976	25080	89.6
Cypripedioideae	*Cypripedium formosanum*	27873	24994	89.7
Epidendroideae	*Cymbidium ensifolium*	30929	29793	96.3
Epidendroideae	*Cymbidium sinense*	28712	27659	96.3
Epidendroideae	*Dendrobium nobile*	10228	9384	91.7
Epidendroideae	*Erycina pusilla*	31454	27745	88.2
Epidendroideae	*Gastrodia elata*	19491	18110	92.9
Epidendroideae	*Oncidium 'Gower Ramsey'*	26396	23630	89.5
Epidendroideae	*Phalaenopsis bellina*	33590	30235	90.0
Epidendroideae	*Phalaenopsis equestris*	35818	33251	92.8
Epidendroideae	*Phalaenopsis lueddemanniana*	28426	24129	84.9
Epidendroideae	*Phalaenopsis modesta*	23088	21948	95.1
Epidendroideae	*Phalaenopsis schilleriana*	28893	28051	97.1
Orchidoideae	*Ophrys sphegodes*	19238	18237	94.8
Orchidoideae	*Orchis italica*	24799	23490	94.7
Vanilloideae	*Vanilla planifolia*	28886	26025	90.1
Total		452056	415318	91.9

Table S12 Annotated protein-coding genes in *P. aphrodite*

Database	Number of genes annotated
NCBI nr	28738 (99.4%)
Swiss-Prot	16364 (56.6%)
InterPro	20357 (70.4%)
Gene Ontology	25467 (88.1%)
Enzyme Commission (EC) assignments by KEGG mapping	5665 (19.6%)
No annotation	149 (0.5%)

Table S15 The number and type of non-coding RNAs in *P. aphrodite*

Type of non-coding RNA	Counts	Total Length (bp)	Fraction of genome (%)
tRNA	268	20,074	0.002
rRNA	152	63,304	0.006
C/D box snoRNAs	9,271	986,571	0.096
H/ACA box snoRNAs	35	4,657	0.000
snRNA; splicing	72	9,302	0.001
microRNA	149	15,965	0.002
Total		1,099,873	0.107

Table S16 The number of genes in shared and species-specific gene families in ten representative plant species

Species	Input genes	No. of Families	Genes in shared families	Species-specific families	Genes in species-specific families	Ungrouped genes	Data sources
Arabidopsis thaliana	27,168	13,230	19,688	866	3,120	4,360	TAIR 10, https://www.arabidopsis.org/
Amborella trichopoda	26,846	12,370	13,533	1,181	4,923	8,390	AmTr v1.0, http://amborella.huck.psu.edu/data
Brachypodium distachyon	34,122	17,354	23,120	720	2,143	8,859	Brachypodium distachyon v3.1, http://phytozome.jgi.doe.gov/
Musa acuminata	35,024	13,350	26,405	730	1,938	6,681	version 2, http://banana-genome-hub.southgreen.fr/
Oryza sativa	41,179	18,150	24,367	1,311	4,238	12,574	RGAP 7 http://rice.plantbiology.msu.edu/index.shtml
Phalaenopsis aphrodite	28,901	12,257	16,817	668	8,215	3,869	This study
Physcomitrella patens	32,623	9,395	14,196	2,275	7,654	10,773	Physcomitrella patens v3.3, http://phytozome.jgi.doe.gov/
Populus trichocarpa	42,922	15,020	28,325	1,517	4,843	9,754	Populus trichocarpa v3.0, http://phytozome.jgi.doe.gov/
Sorghum bicolor	34,148	17,763	23,524	841	2,620	8,004	Sorghum bicolor v3.1, http://phytozome.jgi.doe.gov/
Solanum lycopersicum	33,148	13,701	18,978	1,144	4,290	9,880	ITAG3.10, https://solgenomics.net/

Table S17 Number of MADS-box genes in different species

Species	Total	Type II			Type I			
		Subtotal	MIKC^{C}	MIKC*	Subtotal	M-alpha	M-beta	M-gamma
Phalaenopsis aphrodite	56	38	33	5	18	10	4	4
Phalaenopsis equestris[a]	51	29	28	1	22	10	0	12
Dendrobium catenatum[a]	63	35	32	3	28	15	0	13
Brachypodium distachyon[b]	57	39	32	7	18	9	7	2
Oryza sativa[c]	75	43	38	5	32	13	9	10
Arabidopsis thaliana[d]	106	45	39	6	61	25	20	16

[a]Data from (Zhang et al., 2017)

[b]Data from (Wei et al., 2014)

[c]Data from (Arora et al., 2007)

[d]Data from (Parenicova et al., 2003)

Table S18 Number of flavonoid biosynthesis-related genes in different species

	P. aphrodite		*P. equestris*			
Gene	Total	Full length	Total	Full length	Arabidopsis[a]	Rice[b]
PAL	3	2	3	1	4	9
C4H	2	2	2	2	1	4
C4L	10	10	7	6	10	5
CHS	5	5	5	5	4	2
CHI	2	2	2	1	4	1
F3H	1	1	1	0	5	1
FLS	1	1	1	1	6	1
F3'H	5	4	4	3	1	1
F3'5'H	5	5	8	2	3	1
DFR	1	1	1	0	4	1
ANS	2	1	2	1	2	2
3GT	3	3	3	1	12	1

[a]Data from (Solfanelli et al., 2006)

[b]Data from (Gui et al., 2011; Shih et al., 2008; Tonnessen et al., 2015) and http://www.genome.jp/kegg/kegg2.html

Table S19 Number of carotenoid biosynthesis-related genes in different species

Gene[a]	*P. aphrodite*		*P. equestris*		Arabidopsis[b]	Rice[c]
	Total	Full length	Total	Full length		
PSY	2	2	2	2	1	3
PDS	10	10	7	6	1	1
ZDS	5	5	5	5	1	1
CRTISO	2	2	2	1	2	1
LYB-beta	1	1	1	0	1	1
LYC-epsilon	1	1	1	1	1	1
BCH	5	4	4	3	2	3
ZEP	5	5	8	2	1	1

[a]: Gene names are abbreviated as follows: PSY, phytoene synthase; PDS, phytoene desaturase; ZDS, 9,9'-di-cis-zeta-carotenedesaturase; CRTISO, carotenoid isomerase; LYB-beta, Lycopene beta cyclase ; LYC-epsilon, Lycopene epsilon cyclase ; BCH, beta-carotene hydroxylase ; ZEP, zeaxanthin epoxidase.

[b]Data from (Ruiz-Sola and Rodriguez-Concepcion, 2012).

[c]Data from (Chaudhary et al., 2010).

Supplemental References

Arora, R., Agarwal, P., Ray, S., Singh, A.K., Singh, V.P., Tyagi, A.K. and Kapoor, S. (2007) MADS-box gene family in rice: genome-wide identification, organization and expression profiling during reproductive development and stress. *BMC genomics* **8**, 242.

Chaudhary, N., Nijhawan, A., Khurana, J.P. and Khurana, P. (2010) Carotenoid biosynthesis genes in rice: structural analysis, genome-wide expression profiling and phylogenetic analysis. *Molecular genetics and genomics : MGG* **283**, 13-33.

Gnerre, S., Maccallum, I., Przybylski, D., Ribeiro, F.J., Burton, J.N., Walker, B.J., Sharpe, T., Hall, G., Shea, T.P., Sykes, S., Berlin, A.M., Aird, D., Costello, M., Daza, R., Williams, L., Nicol, R., Gnirke, A., Nusbaum, C., Lander, E.S. and Jaffe, D.B. (2011) High-quality draft assemblies of mammalian genomes from massively parallel sequence data. *Proceedings of the National Academy of Sciences of the United States of America* **108**, 1513-1518.

Gui, J., Shen, J. and Li, L. (2011) Functional characterization of evolutionarily divergent 4-coumarate:coenzyme a ligases in rice. *Plant physiology* **157**, 574-586.

Luo, R., Liu, B., Xie, Y., Li, Z., Huang, W., Yuan, J., He, G., Chen, Y., Pan, Q., Liu, Y., Tang, J., Wu, G., Zhang, H., Shi, Y., Liu, Y., Yu, C., Wang, B., Lu, Y., Han, C., Cheung, D.W., Yiu, S.M., Peng, S., Xiaoqian, Z., Liu, G., Liao, X., Li, Y., Yang, H., Wang, J., Lam, T.W. and Wang, J. (2012) SOAPdenovo2: an empirically improved memory-efficient short-read de novo assembler. *GigaScience* **1**, 18.

Martin, M. (2011) Cutadapt removes adapter sequences from high-throughput sequencing reads. *2011* **17**.

O'Connell, J., Schulz-Trieglaff, O., Carlson, E., Hims, M.M., Gormley, N.A. and Cox, A.J. (2015) NxTrim: optimized trimming of Illumina mate pair reads. *Bioinformatics* **31**, 2035-2037.

Parenicova, L., de Folter, S., Kieffer, M., Horner, D.S., Favalli, C., Busscher, J., Cook, H.E., Ingram, R.M., Kater, M.M., Davies, B., Angenent, G.C. and Colombo, L. (2003) Molecular and phylogenetic analyses of the complete MADS-box transcription factor family in Arabidopsis: new openings to the MADS world. *The Plant cell* **15**, 1538-1551.

Ruiz-Sola, M.A. and Rodriguez-Concepcion, M. (2012) Carotenoid biosynthesis in Arabidopsis: a colorful pathway. *The Arabidopsis book* **10**, e0158.

Shih, C.H., Chu, H., Tang, L.K., Sakamoto, W., Maekawa, M., Chu, I.K., Wang, M. and Lo, C. (2008) Functional characterization of key structural genes in rice flavonoid biosynthesis. *Planta* **228**, 1043-1054.

Solfanelli, C., Poggi, A., Loreti, E., Alpi, A. and Perata, P. (2006) Sucrose-specific induction of the anthocyanin biosynthetic pathway in Arabidopsis. *Plant physiology* **140**, 637-646.

Tonnessen, B.W., Manosalva, P., Lang, J.M., Baraoidan, M., Bordeos, A., Mauleon, R., Oard, J., Hulbert, S., Leung, H. and Leach, J.E. (2015) Rice phenylalanine ammonia-lyase gene OsPAL4 is associated with broad spectrum disease resistance. *Plant molecular biology* **87**, 273-286.

Wei, B., Zhang, R.Z., Guo, J.J., Liu, D.M., Li, A.L., Fan, R.C., Mao, L. and Zhang, X.Q. (2014) Genome-wide analysis of the MADS-box gene family in Brachypodium distachyon. *PloS one* **9**, e84781.
