# Supplementary material for: Chromosome‐level assembly, genetic and physical mapping of Phalaenopsis aphrodite genome provides new insights into species adaptation and resources for orchid breeding
Source: Plant Biotechnol J. 2018 May 23;16(12):2027–41. doi: 10.1111/pbi.12936 (PMC6230949; doi:10.1111/pbi.12936)
Supplement: Supplementary file 2 — Appendix S2 This file contains genetic map and scaffold arrangement of chromosomes 1–19. Appendix S3 This file contains Tables S5–S10, Tables S13–S14, and Tables S20–S23. [file PBI-16-2027-s003.pdf]

S2 Appendix
Genetic map of *P. aphrodite*

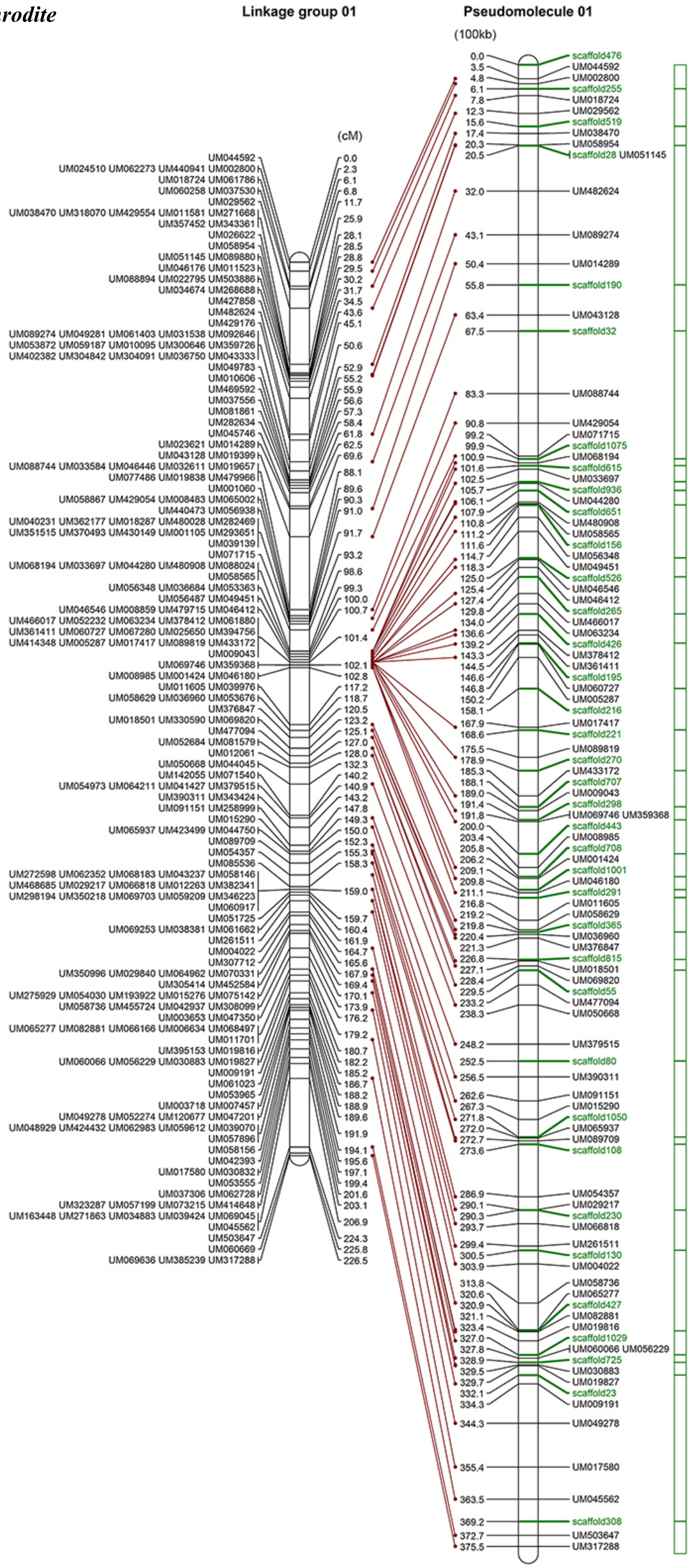

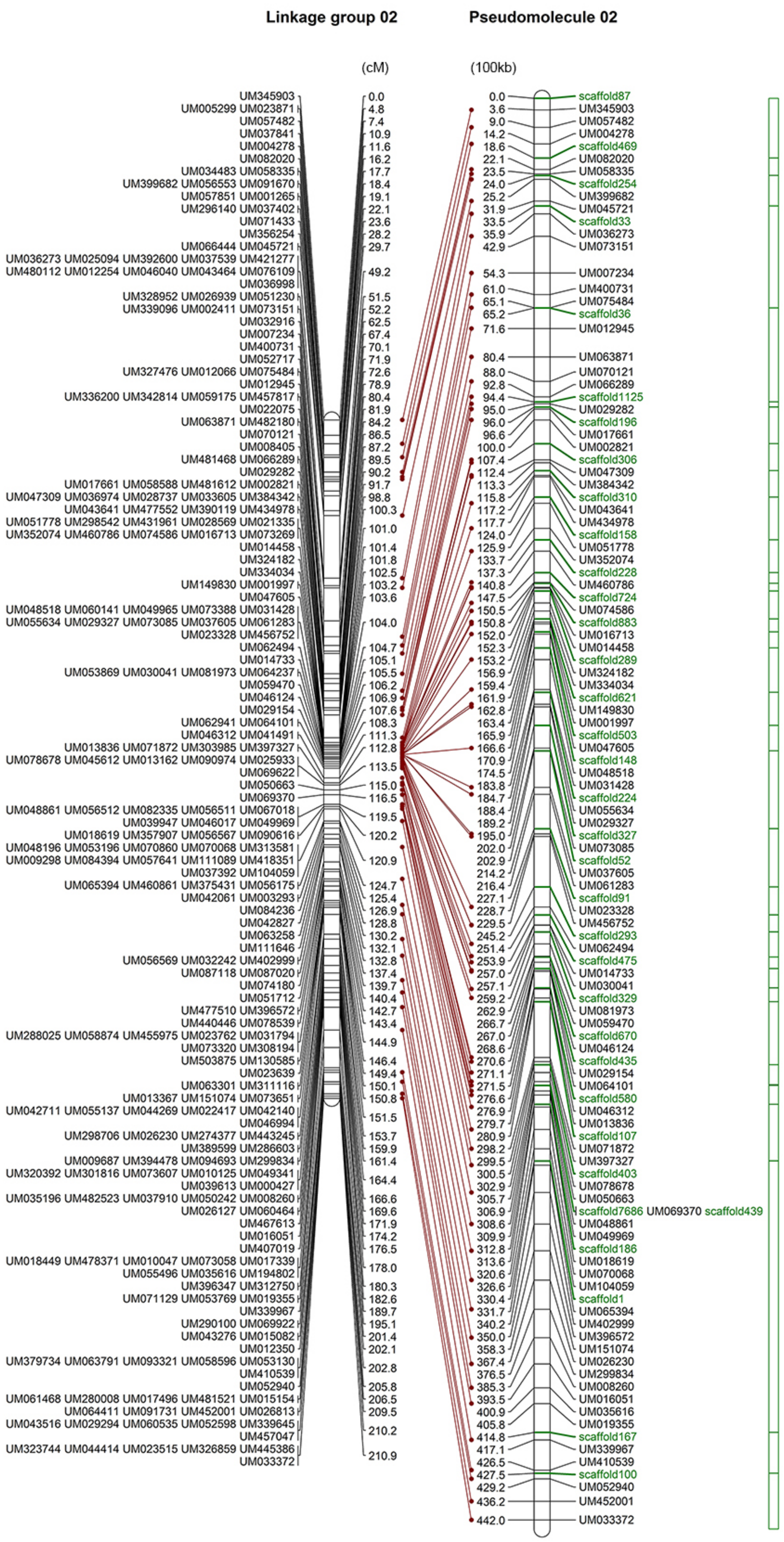

Linkage group 02
Pseudomolecule 02
(cM)
(100kb)
UM345903 0.0
UM005299 UM023871 4.8
UM057482 7.4
UM037841 10.9
UM004278 11.6
UM082020 16.2
UM034483 UM058335 17.7
UM399682 UM056553 UM091670 18.4
UM057851 UM001265 19.1
UM296140 UM037402 22.1
UM071433 23.6
UM356254 28.2
UM066444 UM045721 29.7
UM036273 UM025094 UM392600 UM037539 UM421277
UM480112 UM012254 UM046040 UM043464 UM076109 49.2
UM036998
UM328952 UM026939 UM051230 51.5
UM339096 UM002411 UM073151 52.2
UM032916 62.5
UM007234 67.4
UM400731 70.1
UM052717 71.9
UM327476 UM012066 UM075484 72.6
UM012945 78.9
UM336200 UM342814 UM059175 UM457817 80.4
UM022075 81.9
UM063871 UM482180 84.2
UM070121 86.5
UM008405 87.2
UM481468 UM066289 89.5
UM029282 90.2
UM017661 UM058588 UM481612 UM002821 91.7
UM047309 UM036974 UM028737 UM033605 UM384342 98.8
UM043641 UM477552 UM390119 UM434978 100.3
UM051778 UM298542 UM431961 UM028569 UM021335
UM352074 UM460786 UM074586 UM016713 UM073269 101.0
UM014458 101.4
UM324182 101.8
UM334034 102.5
UM149830 UM001997 103.2
UM047605 103.6
UM048518 UM060141 UM049965 UM073388 UM031428
UM055634 UM029327 UM073085 UM037605 UM061283 104.0
UM023328 UM456752
UM062494 104.7
UM014733 105.1
UM053869 UM030041 UM081973 UM064237 105.5
UM059470 106.2
UM046124 106.9
UM029154 107.6
UM062941 UM064101 108.3
UM046312 UM041491 111.3
UM013836 UM071872 UM303985 UM397327 112.8
UM078678 UM045612 UM013162 UM090974 UM025933
UM069622 113.5
UM050663 115.0
UM069370 116.5
UM048861 UM056512 UM082335 UM056511 UM067018
UM039947 UM046017 UM049969 119.5
UM018619 UM357907 UM056567 UM090616 120.2
UM048196 UM053196 UM070860 UM070068 UM313581
UM009298 UM084394 UM057641 UM111089 UM418351 120.9
UM037392 UM104059
UM065394 UM460861 UM375431 UM056175 124.7
UM042061 UM003293 125.4
UM084236 126.9
UM042827 128.8
UM063258 130.2
UM111646 132.1
UM056569 UM032242 UM402999 132.8
UM087118 UM087020 137.4
UM074180 139.7
UM051712 140.4
UM477510 UM396572 142.7
UM440446 UM078539 143.4
UM288025 UM058874 UM455975 UM023762 UM031794 144.9
UM073320 UM308194
UM503875 UM130585 146.4
UM023639 149.4
UM063301 UM311116 150.1
UM013367 UM151074 UM073651 150.8
UM042711 UM055137 UM044269 UM022417 UM042140 151.5
UM046994
UM298706 UM026230 UM274377 UM443245 153.7
UM389599 UM286603 159.9
UM009687 UM394478 UM094693 UM299834 161.4
UM320392 UM301816 UM073607 UM010125 UM049341 164.4
UM039613 UM000427
UM035196 UM482523 UM037910 UM050242 UM008260 166.6
UM026127 UM060464 169.6
UM467613 171.9
UM016051 174.2
UM407019 176.5
UM018449 UM478371 UM010047 UM073058 UM017339 178.0
UM055496 UM035616 UM194802
UM396347 UM312750 180.3
UM071129 UM053769 UM019355 182.6
UM339967 189.7
UM290100 UM069922 195.1
UM043276 UM015082 201.4
UM012350 202.1
UM379734 UM063791 UM093321 UM058596 UM053130 202.8
UM410539
UM052940 205.8
UM061468 UM280008 UM017496 UM481521 UM015154 206.5
UM064411 UM091731 UM452001 UM026813 209.5
UM043516 UM029294 UM060535 UM052598 UM339645 210.2
UM457047
UM323744 UM044414 UM023515 UM326859 UM445386 210.9
UM033372
0.0 scaffold87
3.6 UM345903
9.0 UM057482
14.2 UM004278
18.6 scaffold469
22.1 UM082020
23.5 UM058335
24.0 scaffold254
25.2 UM399682
31.9 UM045721
33.5 scaffold33
35.9 UM036273
42.9 UM073151
54.3 UM007234
61.0 UM400731
65.1 UM075484
65.2 scaffold36
71.6 UM012945
80.4 UM063871
88.0 UM070121
92.8 UM066289
94.4 scaffold1125
95.0 UM029282
96.0 scaffold196
96.6 UM017661
100.0 UM002821
107.4 scaffold306
112.4 UM047309
113.3 UM384342
115.8 scaffold310
117.2 UM043641
117.7 UM434978
124.0 scaffold158
125.9 UM051778
133.7 UM352074
137.3 scaffold228
140.8 UM460786
147.5 scaffold724
150.5 UM074586
150.8 scaffold883
152.0 UM016713
152.3 UM014458
153.2 scaffold289
156.9 UM324182
159.4 UM334034
161.9 scaffold621
162.8 UM149830
163.4 UM001997
165.9 scaffold503
166.6 UM047605
170.9 scaffold148
174.5 UM048518
183.8 UM031428
184.7 scaffold224
188.4 UM055634
189.2 UM029327
195.0 scaffold327
202.0 UM073085
202.9 scaffold52
214.2 UM037605
216.4 UM061283
227.1 scaffold91
228.7 UM023328
229.5 UM456752
245.2 scaffold293
251.4 UM062494
253.9 scaffold475
257.0 UM014733
257.1 UM030041
259.2 scaffold329
262.9 UM081973
266.7 UM059470
267.0 scaffold670
268.6 UM046124
270.6 scaffold435
271.1 UM029154
271.5 UM064101
276.6 scaffold580
276.9 UM046312
279.7 UM013836
280.9 scaffold107
298.2 UM071872
299.5 UM397327
300.5 scaffold403
302.9 UM078678
305.7 UM050663
306.9 scaffold7686 UM069370 scaffold439
308.6 UM048861
309.9 UM049969
312.8 scaffold186
313.6 UM018619
320.6 UM070068
326.6 UM104059
330.4 scaffold1
331.7 UM065394
340.2 UM402999
350.0 UM396572
358.3 UM151074
367.4 UM026230
376.5 UM299834
385.3 UM008260
393.5 UM016051
400.9 UM035616
405.8 UM019355
414.8 scaffold167
417.1 UM339967
426.5 UM410539
427.5 scaffold100
429.2 UM052940
436.2 UM452001
442.0 UM033372

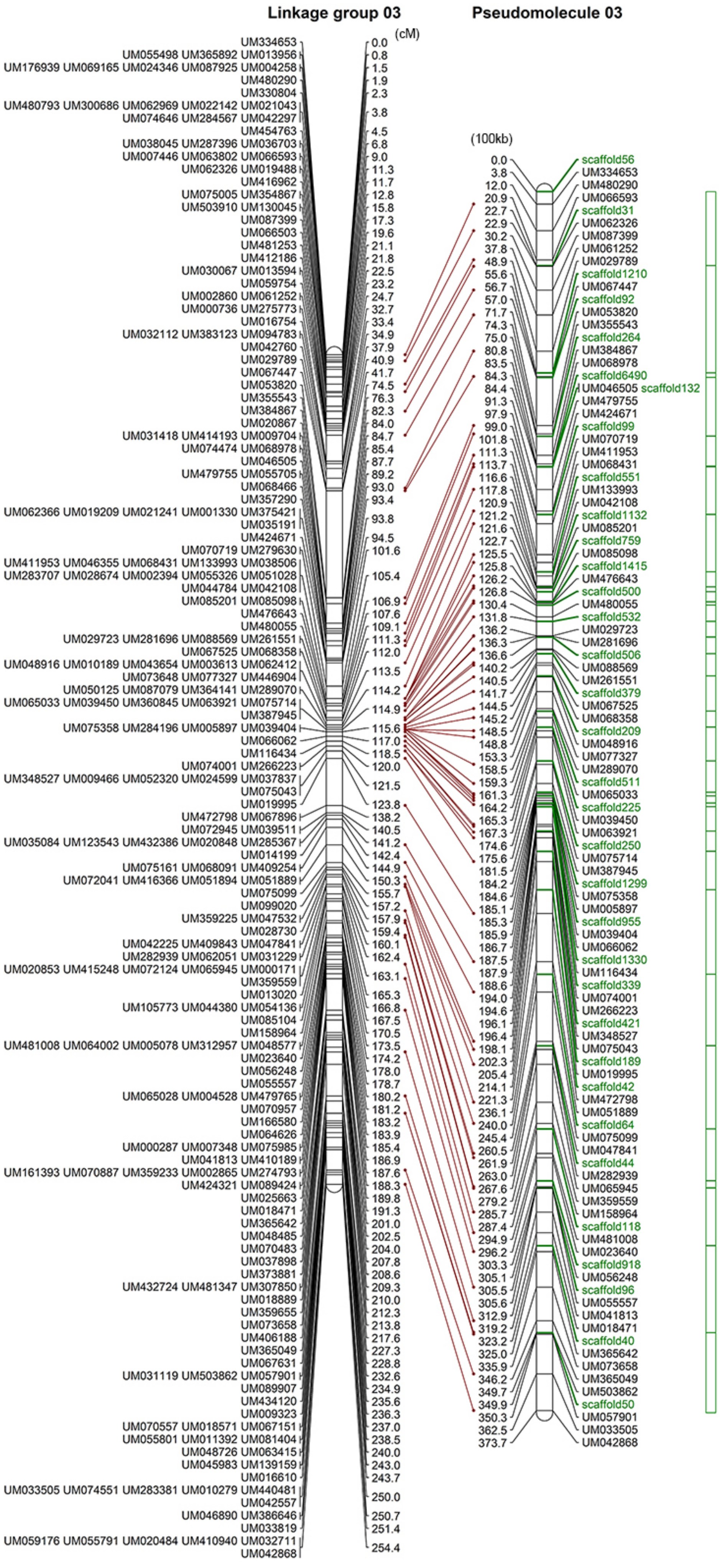

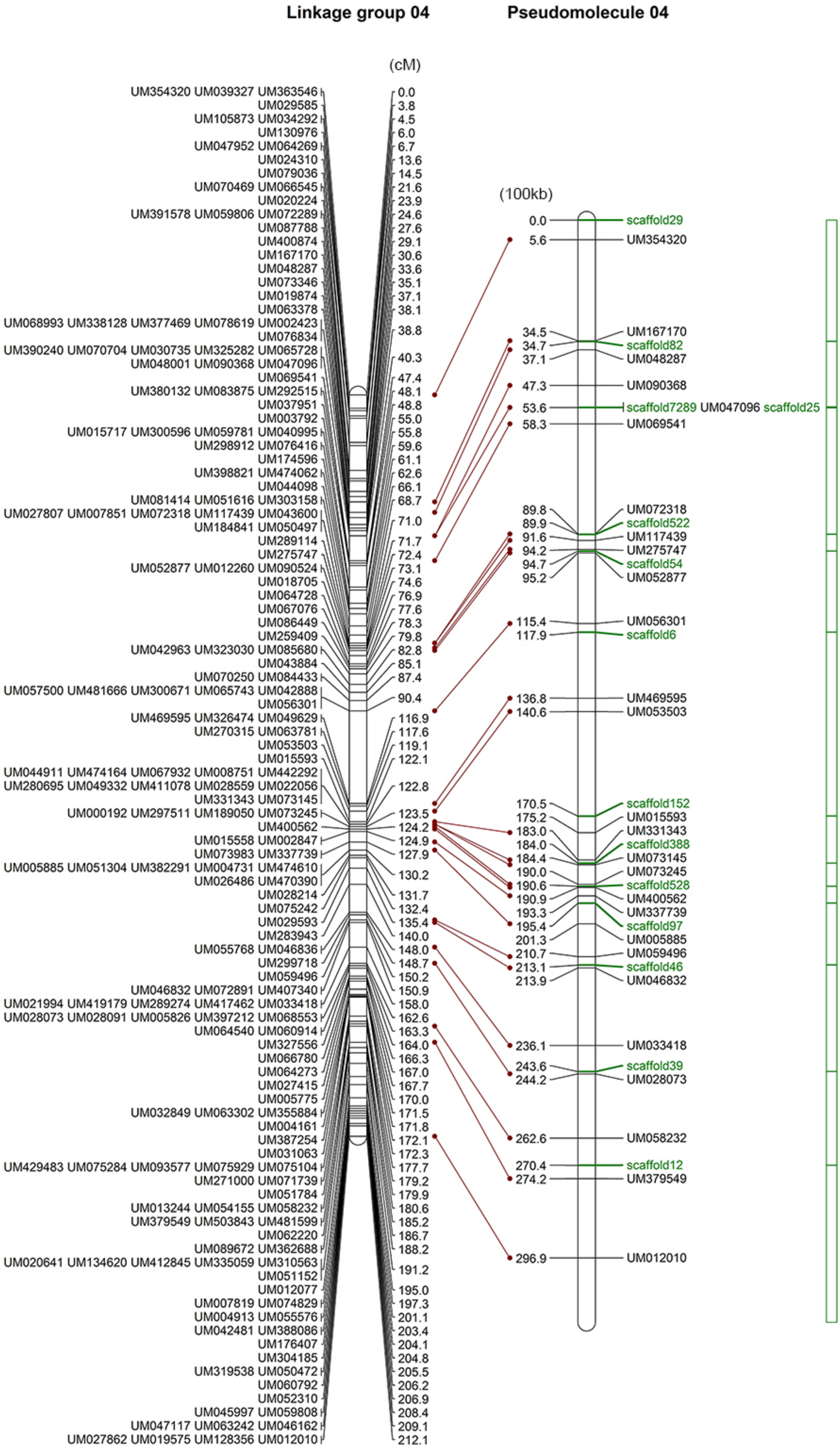
Linkage group 04
Pseudomolecule 04
(cM)
UM354320 UM039327 UM363546 0.0
UM029585 3.8
UM105873 UM034292 4.5
UM130976 6.0
UM047952 UM064269 6.7
UM024310 13.6
UM079036 14.5
UM070469 UM066545 21.6
UM020224 23.9
UM391578 UM059806 UM072289 24.6
UM087788 27.6
UM400874 29.1
UM167170 30.6
UM048287 33.6
UM073346 35.1
UM019874 37.1
UM063378 38.1
UM068993 UM338128 UM377469 UM078619 UM002423
UM076834 38.8
UM390240 UM070704 UM030735 UM325282 UM065728
UM048001 UM090368 UM047096 40.3
UM069541 47.4
UM380132 UM083875 UM292515 48.1
UM037951 48.8
UM003792 55.0
UM015717 UM300596 UM059781 UM040995 55.8
UM298912 UM076416 59.6
UM174596 61.1
UM398821 UM474062 62.6
UM044098 66.1
UM081414 UM051616 UM303158 68.7
UM027807 UM007851 UM072318 UM117439 UM043600
UM184841 UM050497 71.0
UM289114 71.7
UM275747 72.4
UM052877 UM012260 UM090524 73.1
UM018705 74.6
UM064728 76.9
UM067076 77.6
UM086449 78.3
UM259409 79.8
UM042963 UM323030 UM085680 82.8
UM043884 85.1
UM070250 UM084433 87.4
UM057500 UM481666 UM300671 UM065743 UM042888
UM056301 90.4
UM469595 UM326474 UM049629 116.9
UM270315 UM063781 117.6
UM053503 119.1
UM015593 122.1
UM044911 UM474164 UM067932 UM008751 UM442292
UM280695 UM049332 UM411078 UM028559 UM022056
UM331343 UM073145 122.8
UM000192 UM297511 UM189050 UM073245 123.5
UM400562 124.2
UM015558 UM002847 124.9
UM073983 UM337739 127.9
UM005885 UM051304 UM382291 UM004731 UM474610
UM026486 UM470390 130.2
UM028214 131.7
UM075242 132.4
UM029593 135.4
UM283943 140.0
UM055768 UM046836 148.0
UM299718 148.7
UM059496 150.2
UM046832 UM072891 UM407340 150.9
UM021994 UM419179 UM289274 UM417462 UM033418 158.0
UM028073 UM028091 UM005826 UM397212 UM068553 162.6
UM064540 UM060914 163.3
UM327556 164.0
UM066780 166.3
UM064273 167.0
UM027415 167.7
UM005775 170.0
UM032849 UM063302 UM355884 171.5
UM004161 171.8
UM387254 172.1
UM031063 172.3
UM429483 UM075284 UM093577 UM075929 UM075104 177.7
UM271000 UM071739 179.2
UM051784 179.9
UM013244 UM054155 UM058232 180.6
UM379549 UM503843 UM481599 185.2
UM062220 186.7
UM089672 UM362688 188.2
UM020641 UM134620 UM412845 UM335059 UM310563
UM051152 191.2
UM012077 195.0
UM007819 UM074829 197.3
UM004913 UM055576 201.1
UM042481 UM388086 203.4
UM176407 204.1
UM304185 204.8
UM319538 UM050472 205.5
UM060792 206.2
UM052310 206.9
UM045997 UM059808 208.4
UM047117 UM063242 UM046162 209.1
UM027862 UM019575 UM128356 UM012010 212.1
(100kb)
0.0 scaffold29
5.6 UM354320
34.5 UM167170
34.7 scaffold82
37.1 UM048287
47.3 UM090368
53.6 scaffold7289 UM047096 scaffold25
58.3 UM069541
89.8 UM072318
89.9 scaffold522
91.6 UM117439
94.2 UM275747
94.7 scaffold54
95.2 UM052877
115.4 UM056301
117.9 scaffold6
136.8 UM469595
140.6 UM053503
170.5 scaffold152
175.2 UM015593
183.0 UM331343
184.0 scaffold388
184.4 UM073145
190.0 UM073245
190.6 scaffold528
190.9 UM400562
193.3 UM337739
195.4 scaffold97
201.3 UM005885
210.7 UM059496
213.1 scaffold46
213.9 UM046832
236.1 UM033418
243.6 scaffold39
244.2 UM028073
262.6 UM058232
270.4 scaffold12
274.2 UM379549
296.9 UM012010

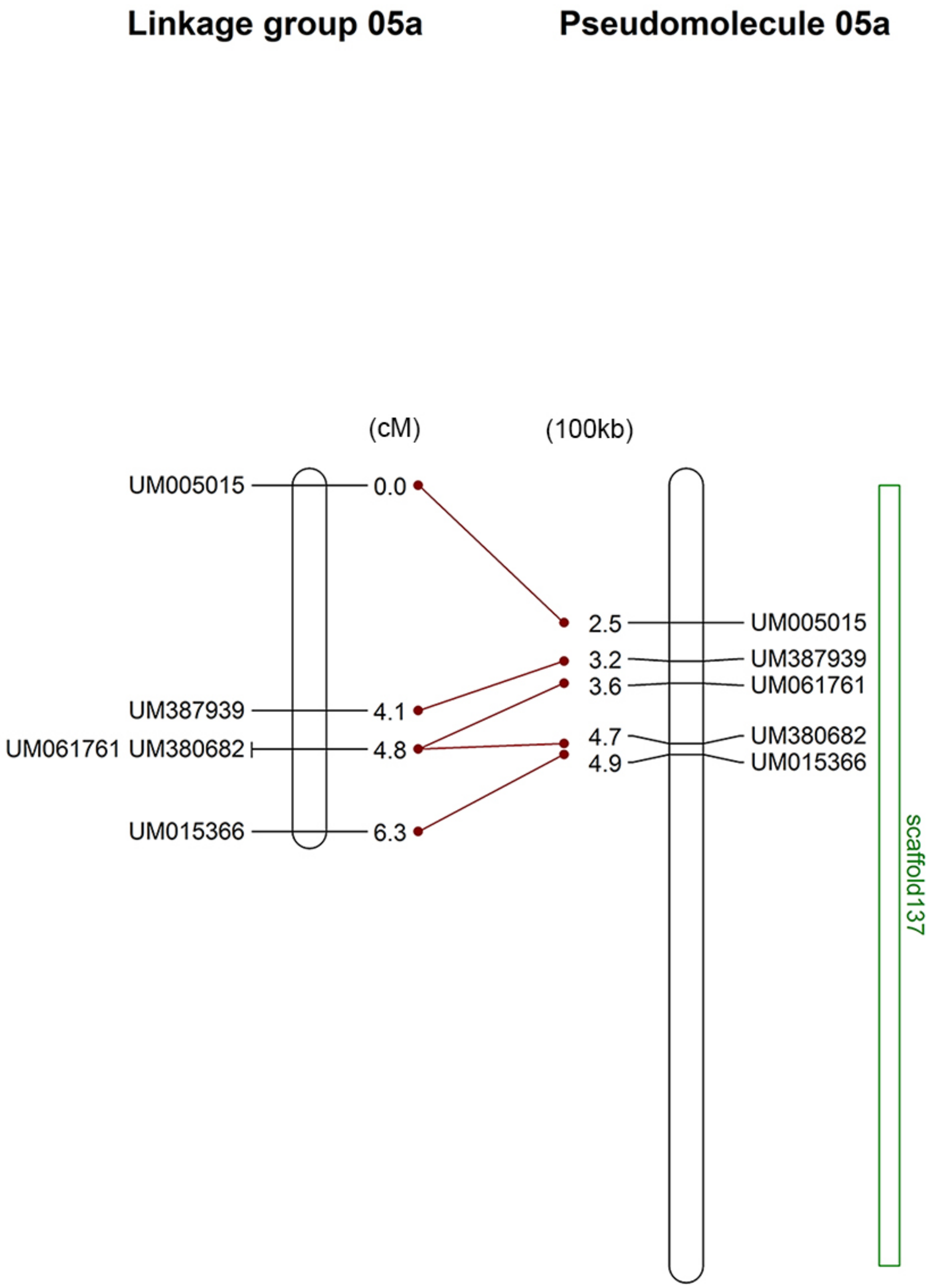

Linkage group 05a
Pseudomolecule 05a
(cM)
(100kb)
UM005015
0.0
UM387939
4.1
UM061761 UM380682
4.8
UM015366
6.3
2.5
UM005015
3.2
UM387939
3.6
UM061761
4.7
UM380682
4.9
UM015366
scaffold137

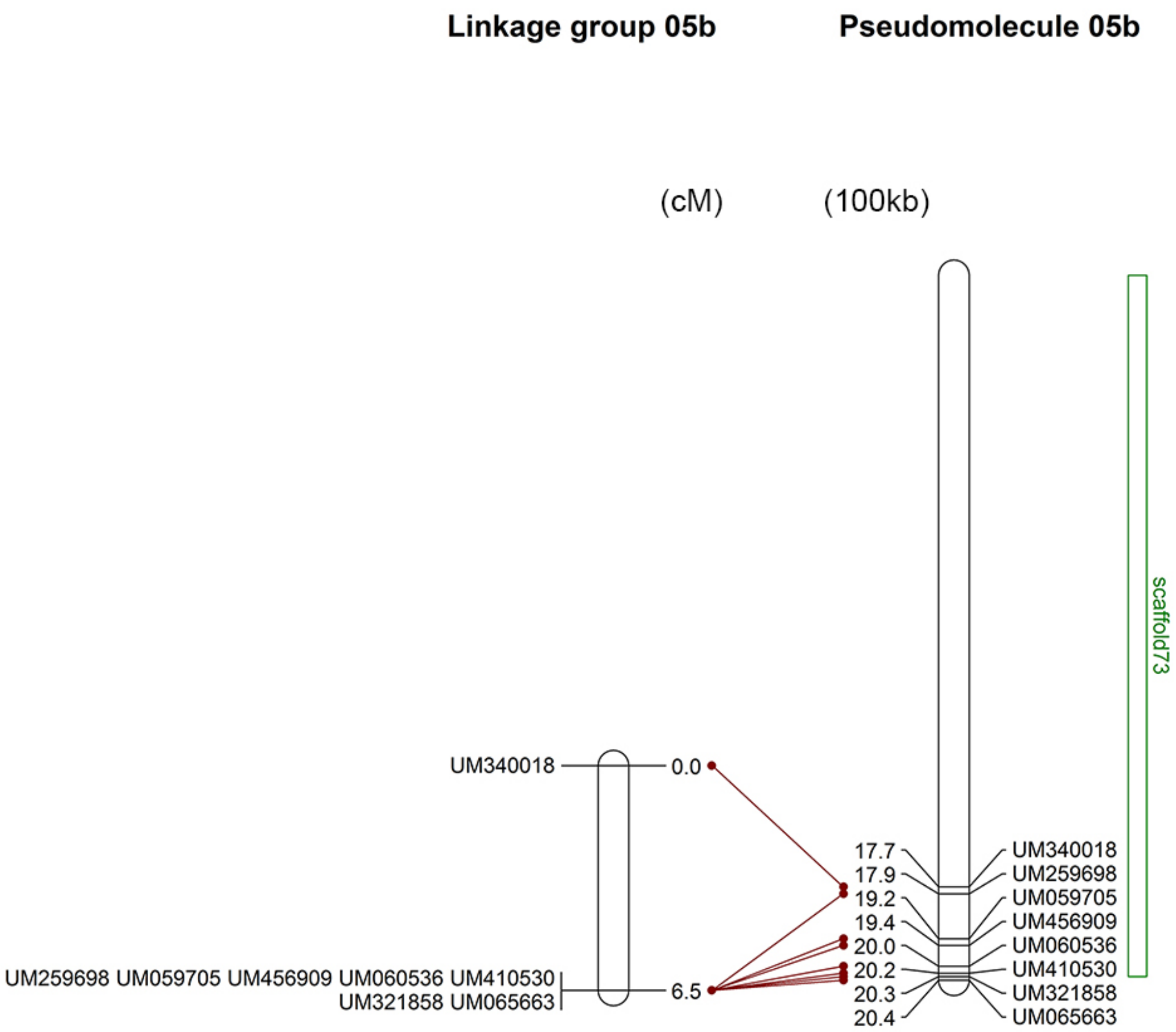
Linkage group 05b
Pseudomolecule 05b
(cM)
(100kb)
scaffold73
UM340018
0.0
UM259698 UM059705 UM456909 UM060536 UM410530
UM321858 UM065663
6.5
17.7
17.9
19.2
19.4
20.0
20.2
20.3
20.4
UM340018
UM259698
UM059705
UM456909
UM060536
UM410530
UM321858
UM065663

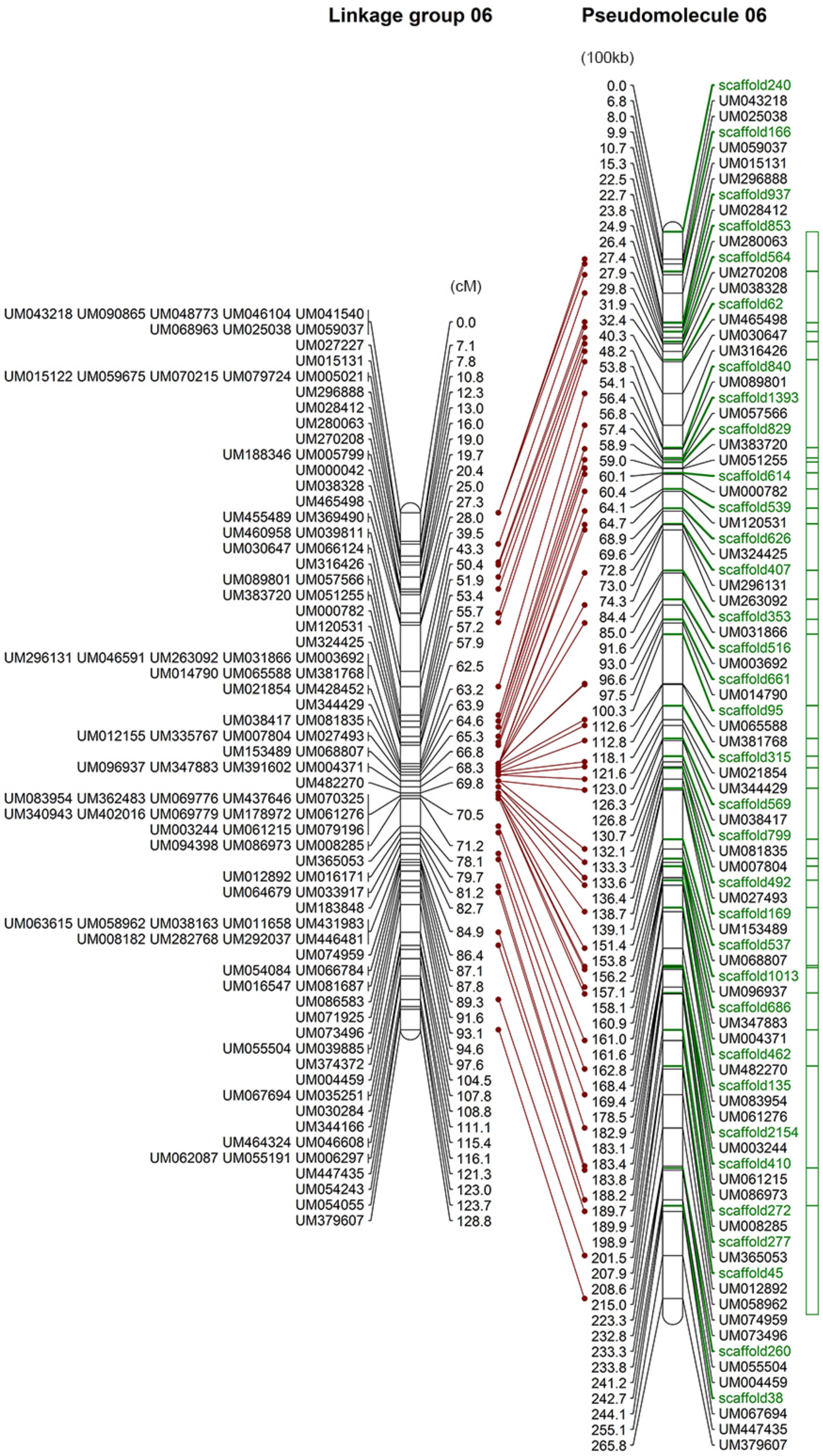
Linkage group 06
Pseudomolecule 06
(cM)
(100kb)
UM043218 UM090865 UM048773 UM046104 UM041540
UM068963 UM025038 UM059037
UM027227
UM015131
UM015122 UM059675 UM070215 UM079724 UM005021
UM296888
UM028412
UM280063
UM270208
UM188346 UM005799
UM000042
UM038328
UM465498
UM455489 UM369490
UM460958 UM039811
UM030647 UM066124
UM316426
UM089801 UM057566
UM383720 UM051255
UM000782
UM120531
UM324425
UM296131 UM046591 UM263092 UM031866 UM003692
UM014790 UM065588 UM381768
UM021854 UM428452
UM344429
UM038417 UM081835
UM012155 UM335767 UM007804 UM027493
UM153489 UM068807
UM096937 UM347883 UM391602 UM004371
UM482270
UM083954 UM362483 UM069776 UM437646 UM070325
UM340943 UM402016 UM069779 UM178972 UM061276
UM003244 UM061215 UM079196
UM094398 UM086973 UM008285
UM365053
UM012892 UM016171
UM064679 UM033917
UM183848
UM063615 UM058962 UM038163 UM011658 UM431983
UM008182 UM282768 UM292037 UM446481
UM074959
UM054084 UM066784
UM016547 UM081687
UM086583
UM071925
UM073496
UM055504 UM039885
UM374372
UM004459
UM067694 UM035251
UM030284
UM344166
UM464324 UM046608
UM062087 UM055191 UM006297
UM447435
UM054243
UM054055
UM379607
0.0
7.1
7.8
10.8
12.3
13.0
16.0
19.0
19.7
20.4
25.0
27.3
28.0
39.5
43.3
50.4
51.9
53.4
55.7
57.2
57.9
62.5
63.2
63.9
64.6
65.3
66.8
68.3
69.8
70.5
71.2
78.1
79.7
81.2
82.7
84.9
86.4
87.1
87.8
89.3
91.6
93.1
94.6
97.6
104.5
107.8
108.8
111.1
115.4
116.1
121.3
123.0
123.7
128.8
0.0
6.8
8.0
9.9
10.7
15.3
22.5
22.7
23.8
24.9
26.4
27.4
27.9
29.8
31.9
32.4
40.3
48.2
53.8
54.1
56.4
56.8
57.4
58.9
59.0
60.1
60.4
64.1
64.7
68.9
69.6
72.8
73.0
74.3
84.4
85.0
91.6
93.0
96.6
97.5
100.3
112.6
112.8
118.1
121.6
123.0
126.3
126.8
130.7
132.1
133.3
133.6
136.4
138.7
139.1
151.4
153.8
156.2
157.1
158.1
160.9
161.0
161.6
162.8
168.4
169.4
178.5
182.9
183.1
183.4
183.8
188.2
189.7
189.9
198.9
201.5
207.9
208.6
215.0
223.3
232.8
233.3
233.8
241.2
242.7
244.1
255.1
265.8
scaffold240
UM043218
UM025038
scaffold166
UM059037
UM015131
UM296888
scaffold937
UM028412
scaffold853
UM280063
scaffold564
UM270208
UM038328
scaffold62
UM465498
UM030647
UM316426
scaffold840
UM089801
scaffold1393
UM057566
scaffold829
UM383720
UM051255
scaffold614
UM000782
scaffold539
UM120531
scaffold626
UM324425
scaffold407
UM296131
UM263092
scaffold353
UM031866
scaffold516
UM003692
scaffold661
UM014790
scaffold95
UM065588
UM381768
scaffold315
UM021854
UM344429
scaffold569
UM038417
scaffold799
UM081835
UM007804
scaffold492
UM027493
scaffold169
UM153489
scaffold537
UM068807
scaffold1013
UM096937
scaffold686
UM347883
UM004371
scaffold462
UM482270
scaffold135
UM083954
UM061276
scaffold2154
UM003244
scaffold410
UM061215
UM086973
scaffold272
UM008285
scaffold277
UM365053
scaffold45
UM012892
UM058962
UM074959
UM073496
scaffold260
UM055504
UM004459
scaffold38
UM067694
UM447435
UM379607

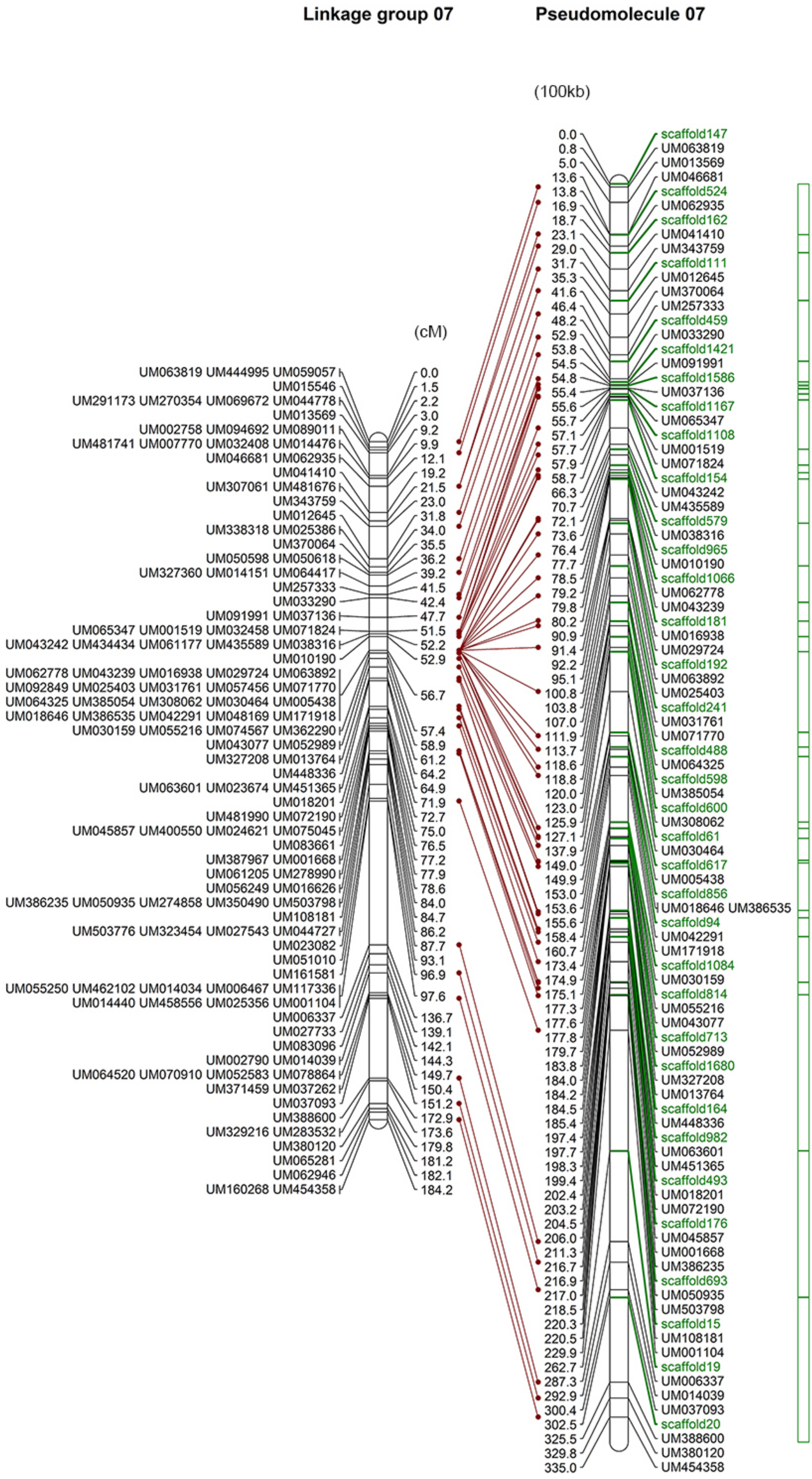

Linkage group 07
Pseudomolecule 07
(cM)
(100kb)
UM063819 UM444995 UM059057
UM015546
UM291173 UM270354 UM069672 UM044778
UM013569
UM002758 UM094692 UM089011
UM481741 UM007770 UM032408 UM014476
UM046681 UM062935
UM041410
UM307061 UM481676
UM343759
UM012645
UM338318 UM025386
UM370064
UM050598 UM050618
UM327360 UM014151 UM064417
UM257333
UM033290
UM091991 UM037136
UM065347 UM001519 UM032458 UM071824
UM043242 UM434434 UM061177 UM435589 UM038316
UM010190
UM062778 UM043239 UM016938 UM029724 UM063892
UM092849 UM025403 UM031761 UM057456 UM071770
UM064325 UM385054 UM308062 UM030464 UM005438
UM018646 UM386535 UM042291 UM048169 UM171918
UM030159 UM055216 UM074567 UM362290
UM043077 UM052989
UM327208 UM013764
UM448336
UM063601 UM023674 UM451365
UM018201
UM481990 UM072190
UM045857 UM400550 UM024621 UM075045
UM083661
UM387967 UM001668
UM061205 UM278990
UM056249 UM016626
UM386235 UM050935 UM274858 UM350490 UM503798
UM108181
UM503776 UM323454 UM027543 UM044727
UM023082
UM051010
UM161581
UM055250 UM462102 UM014034 UM006467 UM117336
UM014440 UM458556 UM025356 UM001104
UM006337
UM027733
UM083096
UM002790 UM014039
UM064520 UM070910 UM052583 UM078864
UM371459 UM037262
UM037093
UM388600
UM329216 UM283532
UM380120
UM065281
UM062946
UM160268 UM454358
0.0
1.5
2.2
3.0
9.2
9.9
12.1
19.2
21.5
23.0
31.8
34.0
35.5
36.2
39.2
41.5
42.4
47.7
51.5
52.2
52.9
56.7
57.4
58.9
61.2
64.2
64.9
71.9
72.7
75.0
76.5
77.2
77.9
78.6
84.0
84.7
86.2
87.7
93.1
96.9
97.6
136.7
139.1
142.1
144.3
149.7
150.4
151.2
172.9
173.6
179.8
181.2
182.1
184.2
0.0
0.8
5.0
13.6
13.8
16.9
18.7
23.1
29.0
31.7
35.3
41.6
46.4
48.2
52.9
53.8
54.5
54.8
55.4
55.6
55.7
57.1
57.7
57.9
58.7
66.3
70.7
72.1
73.6
76.4
77.7
78.5
79.2
79.8
80.2
90.9
91.4
92.2
95.1
100.8
103.8
107.0
111.9
113.7
118.6
118.8
120.0
123.0
125.9
127.1
137.9
149.0
149.9
153.0
153.6
155.6
158.4
160.7
173.4
174.9
175.1
177.3
177.6
177.8
179.7
183.8
184.0
184.2
184.5
185.4
197.4
197.7
198.3
199.4
202.4
203.2
204.5
206.0
211.3
216.7
216.9
217.0
218.5
220.3
220.5
229.9
262.7
287.3
292.9
300.4
302.5
325.5
329.8
335.0
scaffold147
UM063819
UM013569
UM046681
scaffold524
UM062935
scaffold162
UM041410
UM343759
scaffold111
UM012645
UM370064
UM257333
scaffold459
UM033290
scaffold1421
UM091991
scaffold1586
UM037136
scaffold1167
UM065347
scaffold1108
UM001519
UM071824
scaffold154
UM043242
UM435589
scaffold579
UM038316
scaffold965
UM010190
scaffold1066
UM062778
UM043239
scaffold181
UM016938
UM029724
scaffold192
UM063892
UM025403
scaffold241
UM031761
UM071770
scaffold488
UM064325
scaffold598
UM385054
scaffold600
UM308062
scaffold61
UM030464
scaffold617
UM005438
scaffold856
UM018646 UM386535
scaffold94
UM042291
UM171918
scaffold1084
UM030159
scaffold814
UM055216
UM043077
scaffold713
UM052989
scaffold1680
UM327208
UM013764
scaffold164
UM448336
scaffold982
UM063601
UM451365
scaffold493
UM018201
UM072190
scaffold176
UM045857
UM001668
UM386235
scaffold693
UM050935
UM503798
scaffold15
UM108181
UM001104
scaffold19
UM006337
UM014039
UM037093
scaffold20
UM388600
UM380120
UM454358

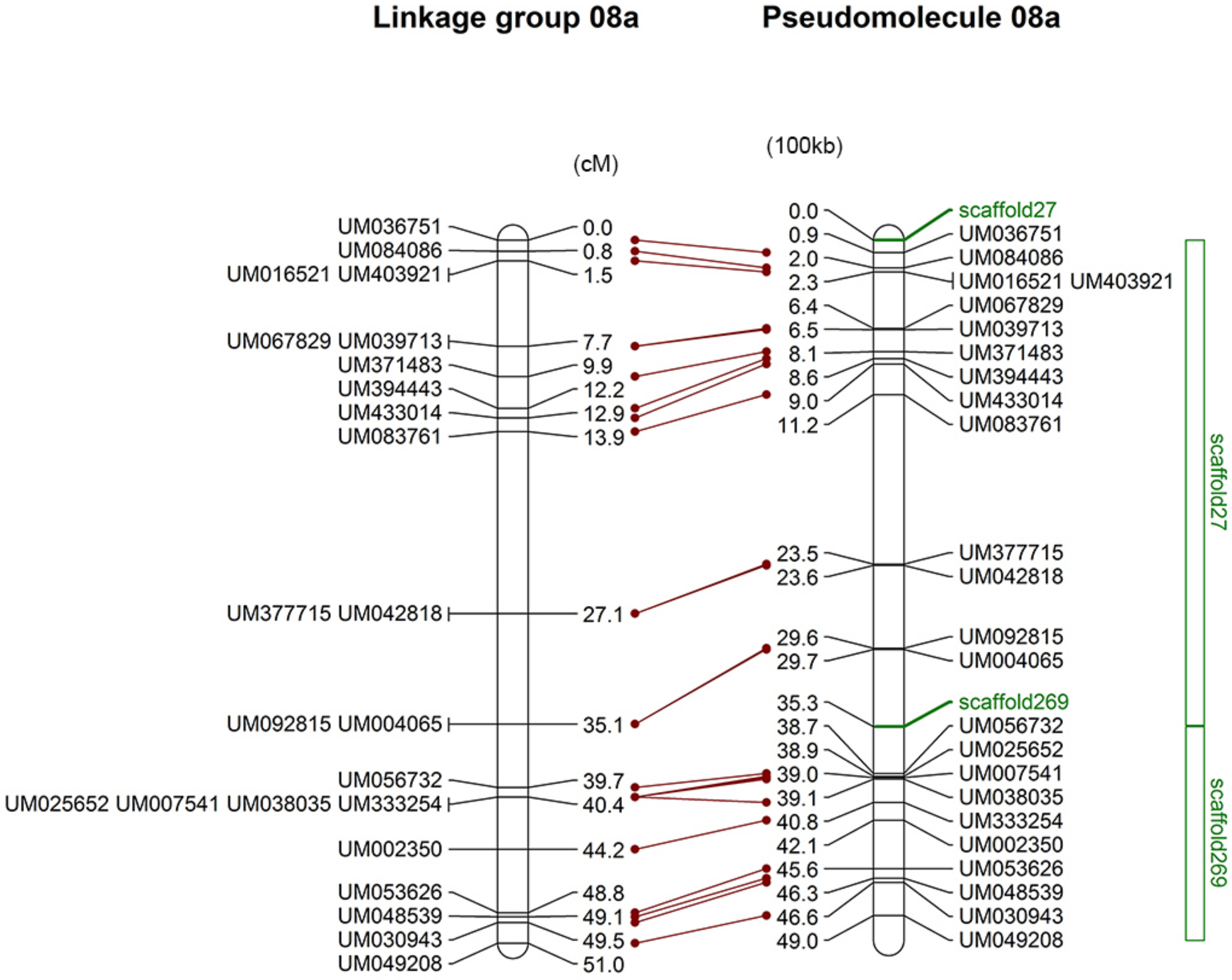

Linkage group 08a
Pseudomolecule 08a
(cM)
(100kb)
UM036751 0.0
UM084086 0.8
UM016521 UM403921 1.5
UM067829 UM039713 7.7
UM371483 9.9
UM394443 12.2
UM433014 12.9
UM083761 13.9
UM377715 UM042818 27.1
UM092815 UM004065 35.1
UM056732 39.7
UM025652 UM007541 UM038035 UM333254 40.4
UM002350 44.2
UM053626 48.8
UM048539 49.1
UM030943 49.5
UM049208 51.0
0.0 scaffold27
0.9 UM036751
2.0 UM084086
2.3 UM016521 UM403921
6.4 UM067829
6.5 UM039713
8.1 UM371483
8.6 UM394443
9.0 UM433014
11.2 UM083761
23.5 UM377715
23.6 UM042818
29.6 UM092815
29.7 UM004065
35.3 scaffold269
38.7 UM056732
38.9 UM025652
39.0 UM007541
39.1 UM038035
40.8 UM333254
42.1 UM002350
45.6 UM053626
46.3 UM048539
46.6 UM030943
49.0 UM049208
scaffold27
scaffold269

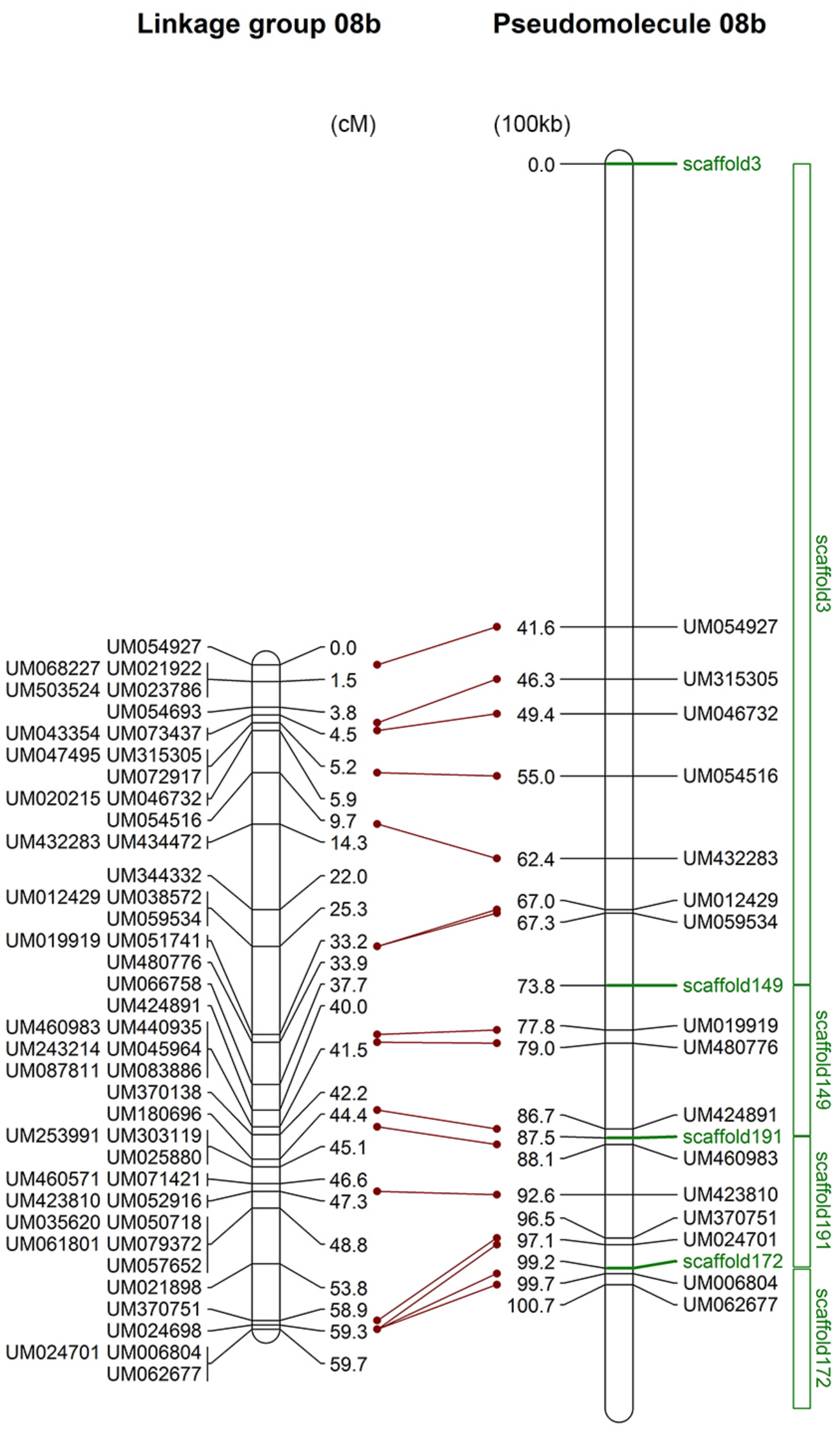

Linkage group 08b
Pseudomolecule 08b
(cM)
(100kb)
scaffold3
scaffold149
scaffold191
scaffold172
UM054927
UM068227 UM021922
UM503524 UM023786
UM054693
UM043354 UM073437
UM047495 UM315305
UM072917
UM020215 UM046732
UM054516
UM432283 UM434472
UM344332
UM012429 UM038572
UM059534
UM019919 UM051741
UM480776
UM066758
UM424891
UM460983 UM440935
UM243214 UM045964
UM087811 UM083886
UM370138
UM180696
UM253991 UM303119
UM025880
UM460571 UM071421
UM423810 UM052916
UM035620 UM050718
UM061801 UM079372
UM057652
UM021898
UM370751
UM024698
UM024701 UM006804
UM062677
0.0
1.5
3.8
4.5
5.2
5.9
9.7
14.3
22.0
25.3
33.2
33.9
37.7
40.0
41.5
42.2
44.4
45.1
46.6
47.3
48.8
53.8
58.9
59.3
59.7
0.0 scaffold3
41.6 UM054927
46.3 UM315305
49.4 UM046732
55.0 UM054516
62.4 UM432283
67.0 UM012429
67.3 UM059534
73.8 scaffold149
77.8 UM019919
79.0 UM480776
86.7 UM424891
87.5 scaffold191
88.1 UM460983
92.6 UM423810
96.5 UM370751
97.1 UM024701
99.2 scaffold172
99.7 UM006804
100.7 UM062677

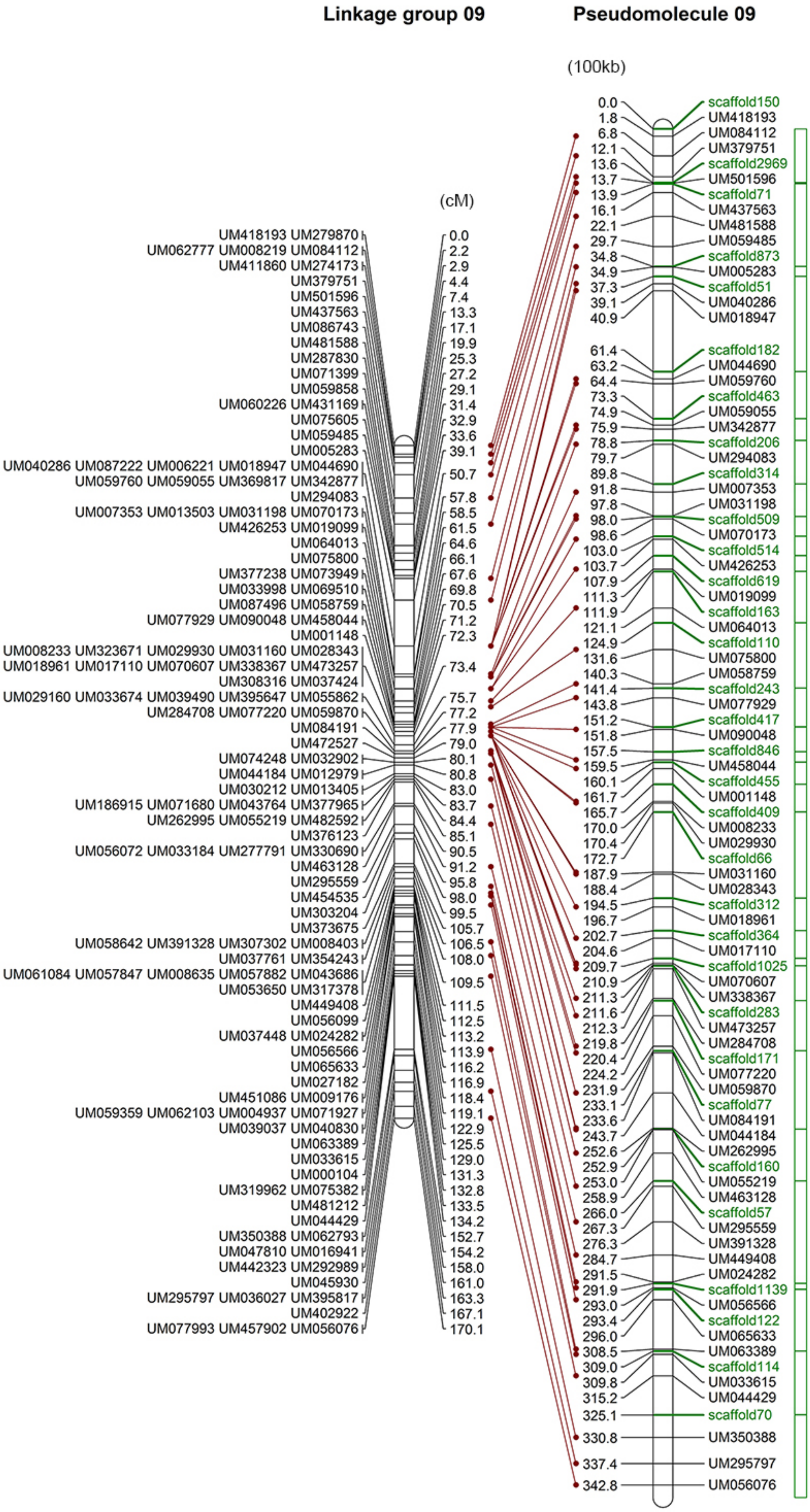

Linkage group 09
Pseudomolecule 09
(cM)
(100kb)
UM418193 UM279870 0.0
UM062777 UM008219 UM084112 2.2
UM411860 UM274173 2.9
UM379751 4.4
UM501596 7.4
UM437563 13.3
UM086743 17.1
UM481588 19.9
UM287830 25.3
UM071399 27.2
UM059858 29.1
UM060226 UM431169 31.4
UM075605 32.9
UM059485 33.6
UM005283 39.1
UM040286 UM087222 UM006221 UM018947 UM044690
UM059760 UM059055 UM369817 UM342877 50.7
UM294083 57.8
UM007353 UM013503 UM031198 UM070173 58.5
UM426253 UM019099 61.5
UM064013 64.6
UM075800 66.1
UM377238 UM073949 67.6
UM033998 UM069510 69.8
UM087496 UM058759 70.5
UM077929 UM090048 UM458044 71.2
UM001148 72.3
UM008233 UM323671 UM029930 UM031160 UM028343
UM018961 UM017110 UM070607 UM338367 UM473257 73.4
UM308316 UM037424
UM029160 UM033674 UM039490 UM395647 UM055862 75.7
UM284708 UM077220 UM059870 77.2
UM084191 77.9
UM472527 79.0
UM074248 UM032902 80.1
UM044184 UM012979 80.8
UM030212 UM013405 83.0
UM186915 UM071680 UM043764 UM377965 83.7
UM262995 UM055219 UM482592 84.4
UM376123 85.1
UM056072 UM033184 UM277791 UM330690 90.5
UM463128 91.2
UM295559 95.8
UM454535 98.0
UM303204 99.5
UM373675 105.7
UM058642 UM391328 UM307302 UM008403 106.5
UM037761 UM354243 108.0
UM061084 UM057847 UM008635 UM057882 UM043686
UM053650 UM317378 109.5
UM449408 111.5
UM056099 112.5
UM037448 UM024282 113.2
UM056566 113.9
UM065633 116.2
UM027182 116.9
UM451086 UM009176 118.4
UM059359 UM062103 UM004937 UM071927 119.1
UM039037 UM040830 122.9
UM063389 125.5
UM033615 129.0
UM000104 131.3
UM319962 UM075382 132.8
UM481212 133.5
UM044429 134.2
UM350388 UM062793 152.7
UM047810 UM016941 154.2
UM442323 UM292989 158.0
UM045930 161.0
UM295797 UM036027 UM395817 163.3
UM402922 167.1
UM077993 UM457902 UM056076 170.1
0.0 scaffold150
1.8 UM418193
6.8 UM084112
12.1 UM379751
13.6 scaffold2969
13.7 UM501596
13.9 scaffold71
16.1 UM437563
22.1 UM481588
29.7 UM059485
34.8 scaffold873
34.9 UM005283
37.3 scaffold51
39.1 UM040286
40.9 UM018947
61.4 scaffold182
63.2 UM044690
64.4 UM059760
73.3 scaffold463
74.9 UM059055
75.9 UM342877
78.8 scaffold206
79.7 UM294083
89.8 scaffold314
91.8 UM007353
97.8 UM031198
98.0 scaffold509
98.6 UM070173
103.0 scaffold514
103.7 UM426253
107.9 scaffold619
111.3 UM019099
111.9 scaffold163
121.1 UM064013
124.9 scaffold110
131.6 UM075800
140.3 UM058759
141.4 scaffold243
143.8 UM077929
151.2 scaffold417
151.8 UM090048
157.5 scaffold846
159.5 UM458044
160.1 scaffold455
161.7 UM001148
165.7 scaffold409
170.0 UM008233
170.4 UM029930
172.7 scaffold66
187.9 UM031160
188.4 UM028343
194.5 scaffold312
196.7 UM018961
202.7 scaffold364
204.6 UM017110
209.7 scaffold1025
210.9 UM070607
211.3 UM338367
211.6 scaffold283
212.3 UM473257
219.8 UM284708
220.4 scaffold171
224.2 UM077220
231.9 UM059870
233.1 scaffold77
233.6 UM084191
243.7 UM044184
252.6 UM262995
252.9 scaffold160
253.0 UM055219
258.9 UM463128
266.0 scaffold57
267.3 UM295559
276.3 UM391328
284.7 UM449408
291.5 UM024282
291.9 scaffold1139
293.0 UM056566
293.4 scaffold122
296.0 UM065633
308.5 UM063389
309.0 scaffold114
309.8 UM033615
315.2 UM044429
325.1 scaffold70
330.8 UM350388
337.4 UM295797
342.8 UM056076

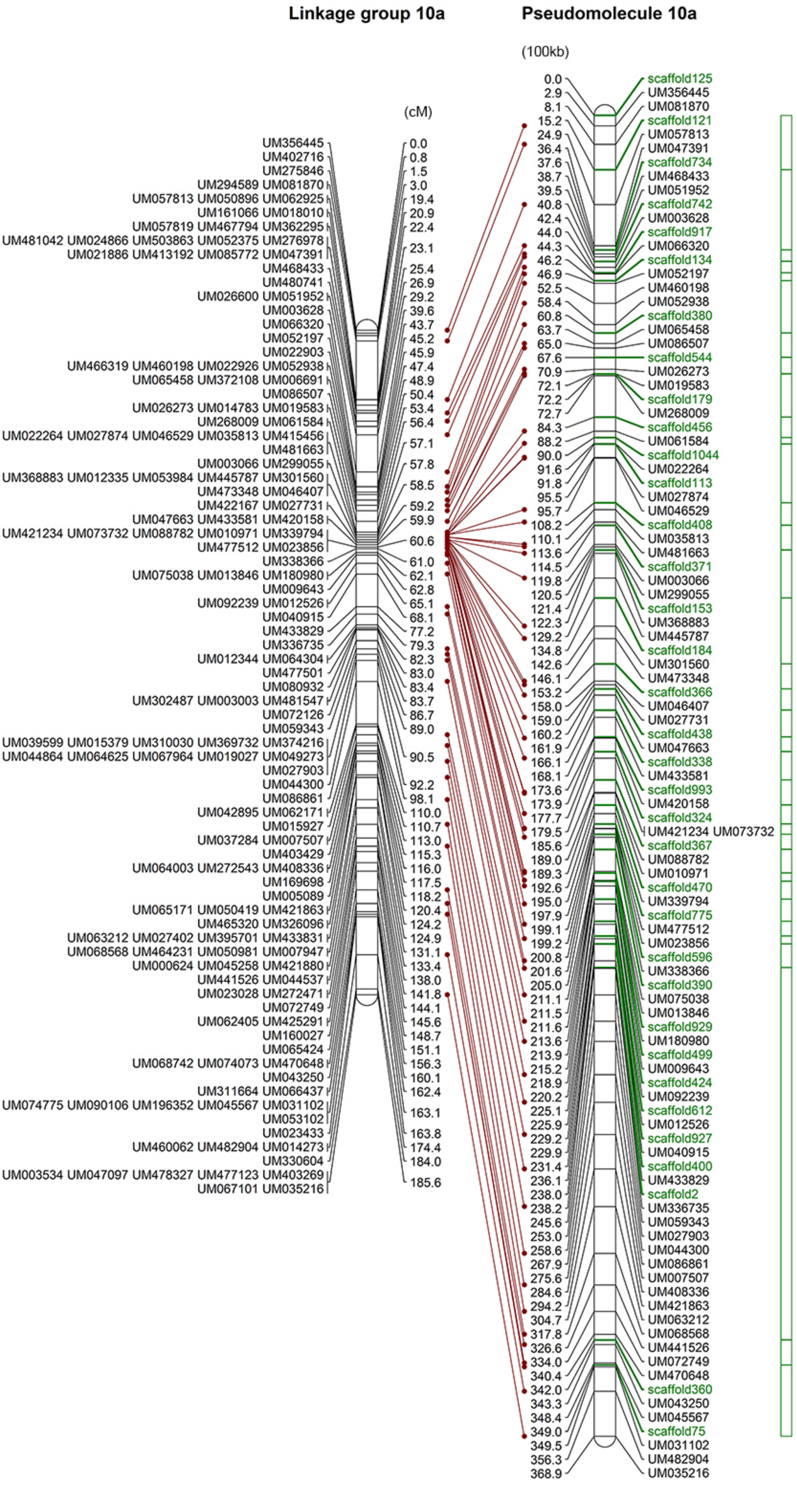
Linkage group 10a
Pseudomolecule 10a
(cM)
UM356445 0.0
UM402716 0.8
UM275846 1.5
UM294589 UM081870 3.0
UM057813 UM050896 UM062925 19.4
UM161066 UM018010 20.9
UM057819 UM467794 UM362295 22.4
UM481042 UM024866 UM503863 UM052375 UM276978
UM021886 UM413192 UM085772 UM047391 23.1
UM468433 25.4
UM480741 26.9
UM026600 UM051952 29.2
UM003628 39.6
UM066320 43.7
UM052197 45.2
UM022903 45.9
UM466319 UM460198 UM022926 UM052938 47.4
UM065458 UM372108 UM006691 48.9
UM086507 50.4
UM026273 UM014783 UM019583 53.4
UM268009 UM061584 56.4
UM022264 UM027874 UM046529 UM035813 UM415456
UM481663 57.1
UM003066 UM299055 57.8
UM368883 UM012335 UM053984 UM445787 UM301560
UM473348 UM046407 58.5
UM422167 UM027731 59.2
UM047663 UM433581 UM420158 59.9
UM421234 UM073732 UM088782 UM010971 UM339794
UM477512 UM023856 60.6
UM338366 61.0
UM075038 UM013846 UM180980 62.1
UM009643 62.8
UM092239 UM012526 65.1
UM040915 68.1
UM433829 77.2
UM336735 79.3
UM012344 UM064304 82.3
UM477501 83.0
UM080932 83.4
UM302487 UM003003 UM481547 83.7
UM072126 86.7
UM059343 89.0
UM039599 UM015379 UM310030 UM369732 UM374216
UM044864 UM064625 UM067964 UM019027 UM049273 90.5
UM027903
UM044300 92.2
UM086861 98.1
UM042895 UM062171 110.0
UM015927 110.7
UM037284 UM007507 113.0
UM403429 115.3
UM064003 UM272543 UM408336 116.0
UM169698 117.5
UM005089 118.2
UM065171 UM050419 UM421863 120.4
UM465320 UM326096 124.2
UM063212 UM027402 UM395701 UM433831 124.9
UM068568 UM464231 UM050981 UM007947 131.1
UM000624 UM045258 UM421880 133.4
UM441526 UM044537 138.0
UM023028 UM272471 141.8
UM072749 144.1
UM062405 UM425291 145.6
UM160027 148.7
UM065424 151.1
UM068742 UM074073 UM470648 156.3
UM043250 160.1
UM311664 UM066437 162.4
UM074775 UM090106 UM196352 UM045567 UM031102
UM053102 163.1
UM023433 163.8
UM460062 UM482904 UM014273 174.4
UM330604 184.0
UM003534 UM047097 UM478327 UM477123 UM403269
UM067101 UM035216 185.6
(100kb)
0.0 scaffold125
2.9 UM356445
8.1 UM081870
15.2 scaffold121
24.9 UM057813
36.4 UM047391
37.6 scaffold734
38.7 UM468433
39.5 UM051952
40.8 scaffold742
42.4 UM003628
44.0 scaffold917
44.3 UM066320
46.2 scaffold134
46.9 UM052197
52.5 UM460198
58.4 UM052938
60.8 scaffold380
63.7 UM065458
65.0 UM086507
67.6 scaffold544
70.9 UM026273
72.1 UM019583
72.2 scaffold179
72.7 UM268009
84.3 scaffold456
88.2 UM061584
90.0 scaffold1044
91.6 UM022264
91.8 scaffold113
95.5 UM027874
95.7 UM046529
108.2 scaffold408
110.1 UM035813
113.6 UM481663
114.5 scaffold371
119.8 UM003066
120.5 UM299055
121.4 scaffold153
122.3 UM368883
129.2 UM445787
134.8 scaffold184
142.6 UM301560
146.1 UM473348
153.2 scaffold366
158.0 UM046407
159.0 UM027731
160.2 scaffold438
161.9 UM047663
166.1 scaffold338
168.1 UM433581
173.6 scaffold993
173.9 UM420158
177.7 scaffold324
179.5 UM421234 UM073732
185.6 scaffold367
189.0 UM088782
189.3 UM010971
192.6 scaffold470
195.0 UM339794
197.9 scaffold775
199.1 UM477512
199.2 UM023856
200.8 scaffold596
201.6 UM338366
205.0 scaffold390
211.1 UM075038
211.5 UM013846
211.6 scaffold929
213.6 UM180980
213.9 scaffold499
215.2 UM009643
218.9 scaffold424
220.2 UM092239
225.1 scaffold612
225.9 UM012526
229.2 scaffold927
229.9 UM040915
231.4 scaffold400
236.1 UM433829
238.0 scaffold2
238.2 UM336735
245.6 UM059343
253.0 UM027903
258.6 UM044300
267.9 UM086861
275.6 UM007507
284.6 UM408336
294.2 UM421863
304.7 UM063212
317.8 UM068568
326.6 UM441526
334.0 UM072749
340.4 UM470648
342.0 scaffold360
343.3 UM043250
348.4 UM045567
349.0 scaffold75
349.5 UM031102
356.3 UM482904
368.9 UM035216

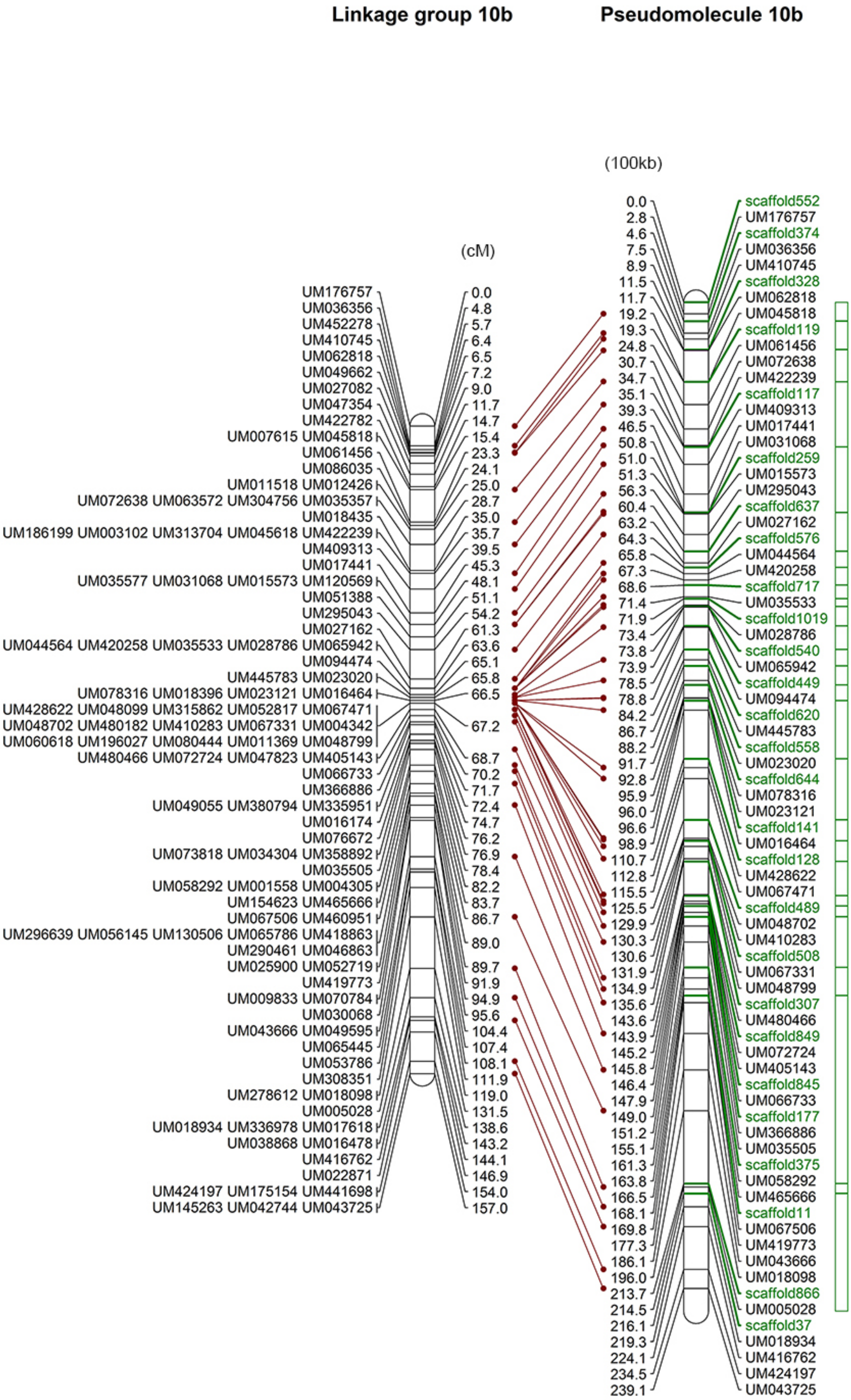

Linkage group 10b
Pseudomolecule 10b
(cM)
UM176757 0.0
UM036356 4.8
UM452278 5.7
UM410745 6.4
UM062818 6.5
UM049662 7.2
UM027082 9.0
UM047354 11.7
UM422782 14.7
UM007615 UM045818 15.4
UM061456 23.3
UM086035 24.1
UM011518 UM012426 25.0
UM072638 UM063572 UM304756 UM035357 28.7
UM018435 35.0
UM186199 UM003102 UM313704 UM045618 UM422239 35.7
UM409313 39.5
UM017441 45.3
UM035577 UM031068 UM015573 UM120569 48.1
UM051388 51.1
UM295043 54.2
UM027162 61.3
UM044564 UM420258 UM035533 UM028786 UM065942 63.6
UM094474 65.1
UM445783 UM023020 65.8
UM078316 UM018396 UM023121 UM016464 66.5
UM428622 UM048099 UM315862 UM052817 UM067471
UM048702 UM480182 UM410283 UM067331 UM004342 67.2
UM060618 UM196027 UM080444 UM011369 UM048799
UM480466 UM072724 UM047823 UM405143 68.7
UM066733 70.2
UM366886 71.7
UM049055 UM380794 UM335951 72.4
UM016174 74.7
UM076672 76.2
UM073818 UM034304 UM358892 76.9
UM035505 78.4
UM058292 UM001558 UM004305 82.2
UM154623 UM465666 83.7
UM067506 UM460951 86.7
UM296639 UM056145 UM130506 UM065786 UM418863
UM290461 UM046863 89.0
UM025900 UM052719 89.7
UM419773 91.9
UM009833 UM070784 94.9
UM030068 95.6
UM043666 UM049595 104.4
UM065445 107.4
UM053786 108.1
UM308351 111.9
UM278612 UM018098 119.0
UM005028 131.5
UM018934 UM336978 UM017618 138.6
UM038868 UM016478 143.2
UM416762 144.1
UM022871 146.9
UM424197 UM175154 UM441698 154.0
UM145263 UM042744 UM043725 157.0
(100kb)
0.0 scaffold552
2.8 UM176757
4.6 scaffold374
7.5 UM036356
8.9 UM410745
11.5 scaffold328
11.7 UM062818
19.2 UM045818
19.3 scaffold119
24.8 UM061456
30.7 UM072638
34.7 UM422239
35.1 scaffold117
39.3 UM409313
46.5 UM017441
50.8 UM031068
51.0 scaffold259
51.3 UM015573
56.3 UM295043
60.4 scaffold637
63.2 UM027162
64.3 scaffold576
65.8 UM044564
67.3 UM420258
68.6 scaffold717
71.4 UM035533
71.9 scaffold1019
73.4 UM028786
73.8 scaffold540
73.9 UM065942
78.5 scaffold449
78.8 UM094474
84.2 scaffold620
86.7 UM445783
88.2 scaffold558
91.7 UM023020
92.8 scaffold644
95.9 UM078316
96.0 UM023121
96.6 scaffold141
98.9 UM016464
110.7 scaffold128
112.8 UM428622
115.5 UM067471
125.5 scaffold489
129.9 UM048702
130.3 UM410283
130.6 scaffold508
131.9 UM067331
134.9 UM048799
135.6 scaffold307
143.6 UM480466
143.9 scaffold849
145.2 UM072724
145.8 UM405143
146.4 scaffold845
147.9 UM066733
149.0 scaffold177
151.2 UM366886
155.1 UM035505
161.3 scaffold375
163.8 UM058292
166.5 UM465666
168.1 scaffold11
169.8 UM067506
177.3 UM419773
186.1 UM043666
196.0 UM018098
213.7 scaffold866
214.5 UM005028
216.1 scaffold37
219.3 UM018934
224.1 UM416762
234.5 UM424197
239.1 UM043725

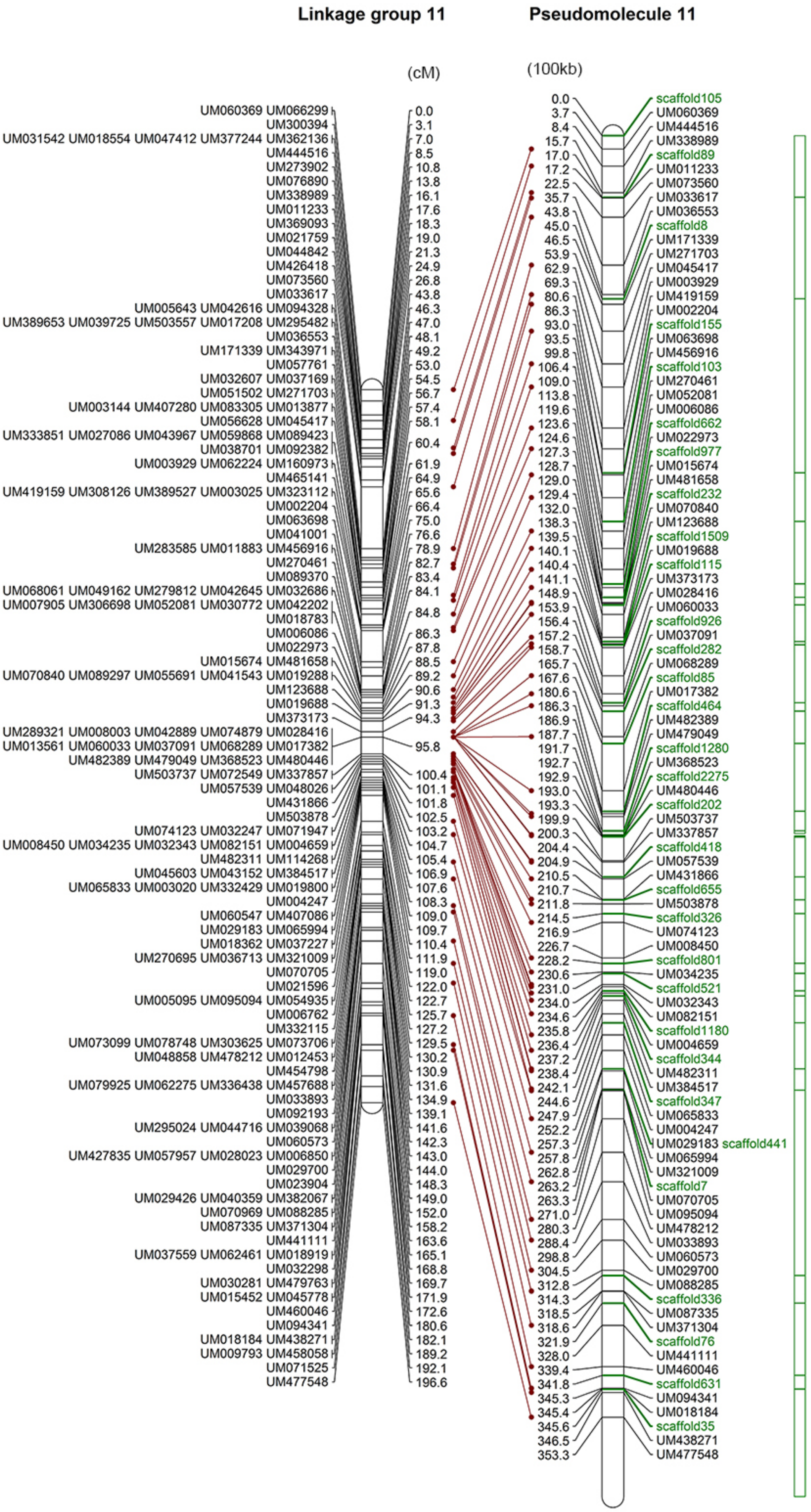
Linkage group 11
Pseudomolecule 11
(cM)
(100kb)
UM060369 UM066299 0.0
UM300394 3.1
UM031542 UM018554 UM047412 UM377244 UM362136 7.0
UM444516 8.5
UM273902 10.8
UM076890 13.8
UM338989 16.1
UM011233 17.6
UM369093 18.3
UM021759 19.0
UM044842 21.3
UM426418 24.9
UM073560 26.8
UM033617 43.8
UM005643 UM042616 UM094328 46.3
UM389653 UM039725 UM503557 UM017208 UM295482 47.0
UM036553 48.1
UM171339 UM343971 49.2
UM057761 53.0
UM032607 UM037169 54.5
UM051502 UM271703 56.7
UM003144 UM407280 UM083305 UM013877 57.4
UM056628 UM045417 58.1
UM333851 UM027086 UM043967 UM059868 UM089423
UM038701 UM092382 60.4
UM003929 UM062224 UM160973 61.9
UM465141 64.9
UM419159 UM308126 UM389527 UM003025 UM323112 65.6
UM002204 66.4
UM063698 75.0
UM041001 76.6
UM283585 UM011883 UM456916 78.9
UM270461 82.7
UM089370 83.4
UM068061 UM049162 UM279812 UM042645 UM032686 84.1
UM007905 UM306698 UM052081 UM030772 UM042202
UM018783 84.8
UM006086 86.3
UM022973 87.8
UM015674 UM481658 88.5
UM070840 UM089297 UM055691 UM041543 UM019288 89.2
UM123688 90.6
UM019688 91.3
UM373173 94.3
UM289321 UM008003 UM042889 UM074879 UM028416
UM013561 UM060033 UM037091 UM068289 UM017382 95.8
UM482389 UM479049 UM368523 UM480446
UM503737 UM072549 UM337857 100.4
UM057539 UM048026 101.1
UM431866 101.8
UM503878 102.5
UM074123 UM032247 UM071947 103.2
UM008450 UM034235 UM032343 UM082151 UM004659 104.7
UM482311 UM114268 105.4
UM045603 UM043152 UM384517 106.9
UM065833 UM003020 UM332429 UM019800 107.6
UM004247 108.3
UM060547 UM407086 109.0
UM029183 UM065994 109.7
UM018362 UM037227 110.4
UM270695 UM036713 UM321009 111.9
UM070705 119.0
UM021596 122.0
UM005095 UM095094 UM054935 122.7
UM006762 125.7
UM332115 127.2
UM073099 UM078748 UM303625 UM073706 129.5
UM048858 UM478212 UM012453 130.2
UM454798 130.9
UM079925 UM062275 UM336438 UM457688 131.6
UM033893 134.9
UM092193 139.1
UM295024 UM044716 UM039068 141.6
UM060573 142.3
UM427835 UM057957 UM028023 UM006850 143.0
UM029700 144.0
UM023904 148.3
UM029426 UM040359 UM382067 149.0
UM070969 UM088285 152.0
UM087335 UM371304 158.2
UM441111 163.6
UM037559 UM062461 UM018919 165.1
UM032298 168.8
UM030281 UM479763 169.7
UM015452 UM045778 171.9
UM460046 172.6
UM094341 180.6
UM018184 UM438271 182.1
UM009793 UM458058 189.2
UM071525 192.1
UM477548 196.6
0.0 scaffold105
3.7 UM060369
8.4 UM444516
15.7 UM338989
17.0 scaffold89
17.2 UM011233
22.5 UM073560
35.7 UM033617
43.8 UM036553
45.0 scaffold8
46.5 UM171339
53.9 UM271703
62.9 UM045417
69.3 UM003929
80.6 UM419159
86.3 UM002204
93.0 scaffold155
93.5 UM063698
99.8 UM456916
106.4 scaffold103
109.0 UM270461
113.8 UM052081
119.6 UM006086
123.6 scaffold662
124.6 UM022973
127.3 scaffold977
128.7 UM015674
129.0 UM481658
129.4 scaffold232
132.0 UM070840
138.3 UM123688
139.5 scaffold1509
140.1 UM019688
140.4 scaffold115
141.1 UM373173
148.9 UM028416
153.9 UM060033
156.4 scaffold926
157.2 UM037091
158.7 scaffold282
165.7 UM068289
167.6 scaffold85
180.6 UM017382
186.3 scaffold464
186.9 UM482389
187.7 UM479049
191.7 scaffold1280
192.7 UM368523
192.9 scaffold2275
193.0 UM480446
193.3 scaffold202
199.9 UM503737
200.3 UM337857
204.4 scaffold418
204.9 UM057539
210.5 UM431866
210.7 scaffold655
211.8 UM503878
214.5 scaffold326
216.9 UM074123
226.7 UM008450
228.2 scaffold801
230.6 UM034235
231.0 scaffold521
234.0 UM032343
234.6 UM082151
235.8 scaffold1180
236.4 UM004659
237.2 scaffold344
238.4 UM482311
242.1 UM384517
244.6 scaffold347
247.9 UM065833
252.2 UM004247
257.3 UM029183 scaffold441
257.8 UM065994
262.8 UM321009
263.2 scaffold7
263.3 UM070705
271.0 UM095094
280.3 UM478212
288.4 UM033893
298.8 UM060573
304.5 UM029700
312.8 UM088285
314.3 scaffold336
318.5 UM087335
318.6 UM371304
321.9 scaffold76
328.0 UM441111
339.4 UM460046
341.8 scaffold631
345.3 UM094341
345.4 UM018184
345.6 scaffold35
346.5 UM438271
353.3 UM477548

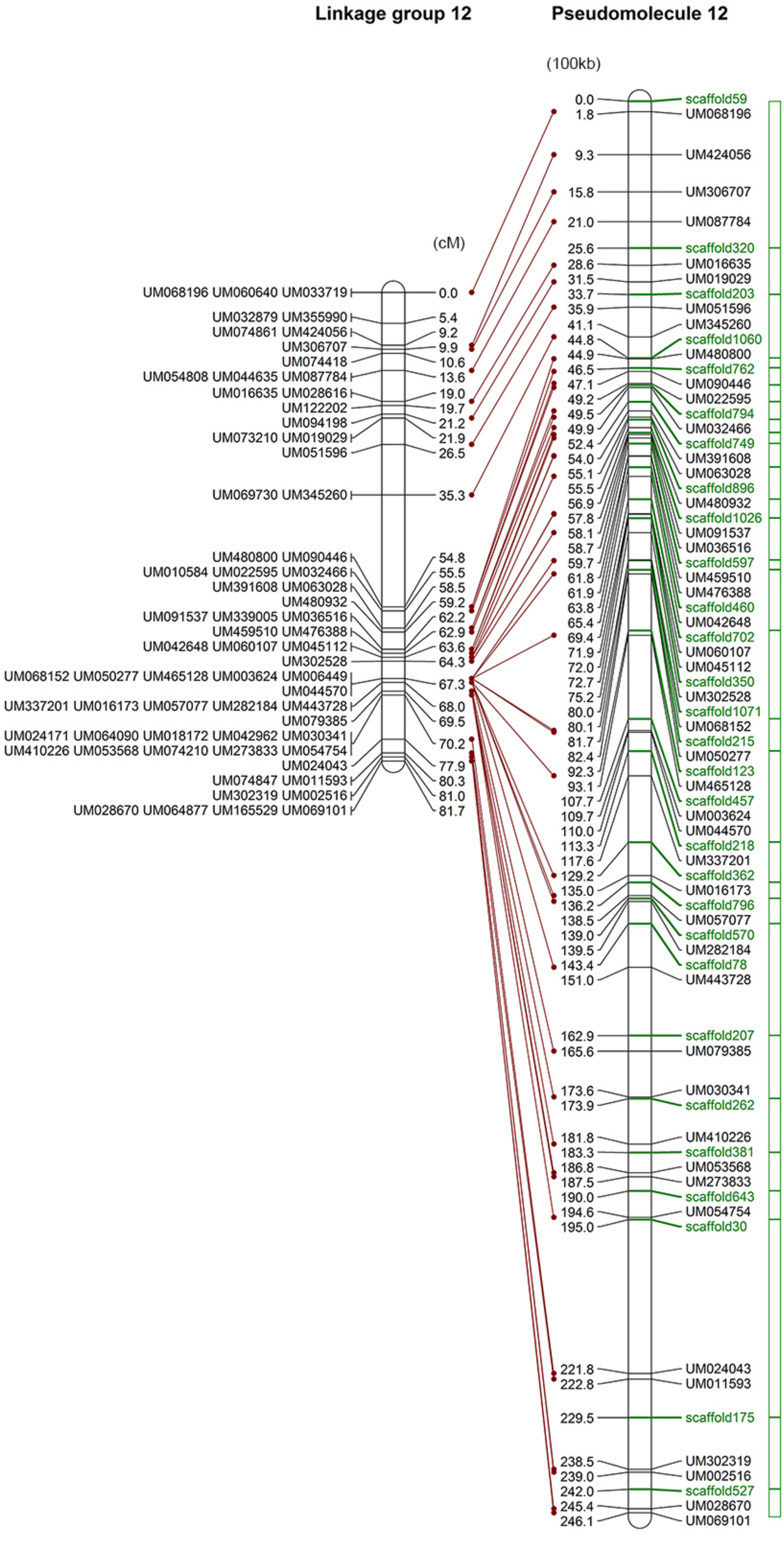

Linkage group 12
Pseudomolecule 12
(cM)
(100kb)
UM068196 UM060640 UM033719
UM032879 UM355990
UM074861 UM424056
UM306707
UM074418
UM054808 UM044635 UM087784
UM016635 UM028616
UM122202
UM094198
UM073210 UM019029
UM051596
UM069730 UM345260
UM480800 UM090446
UM010584 UM022595 UM032466
UM391608 UM063028
UM480932
UM091537 UM339005 UM036516
UM459510 UM476388
UM042648 UM060107 UM045112
UM302528
UM068152 UM050277 UM465128 UM003624 UM006449
UM044570
UM337201 UM016173 UM057077 UM282184 UM443728
UM079385
UM024171 UM064090 UM018172 UM042962 UM030341
UM410226 UM053568 UM074210 UM273833 UM054754
UM024043
UM074847 UM011593
UM302319 UM002516
UM028670 UM064877 UM165529 UM069101
scaffold59
scaffold320
scaffold203
scaffold1060
scaffold762
scaffold794
scaffold749
scaffold896
scaffold1026
scaffold597
scaffold460
scaffold702
scaffold350
scaffold1071
scaffold215
scaffold123
scaffold457
scaffold218
scaffold362
scaffold796
scaffold570
scaffold78
scaffold207
scaffold262
scaffold381
scaffold643
scaffold30
scaffold175
scaffold527

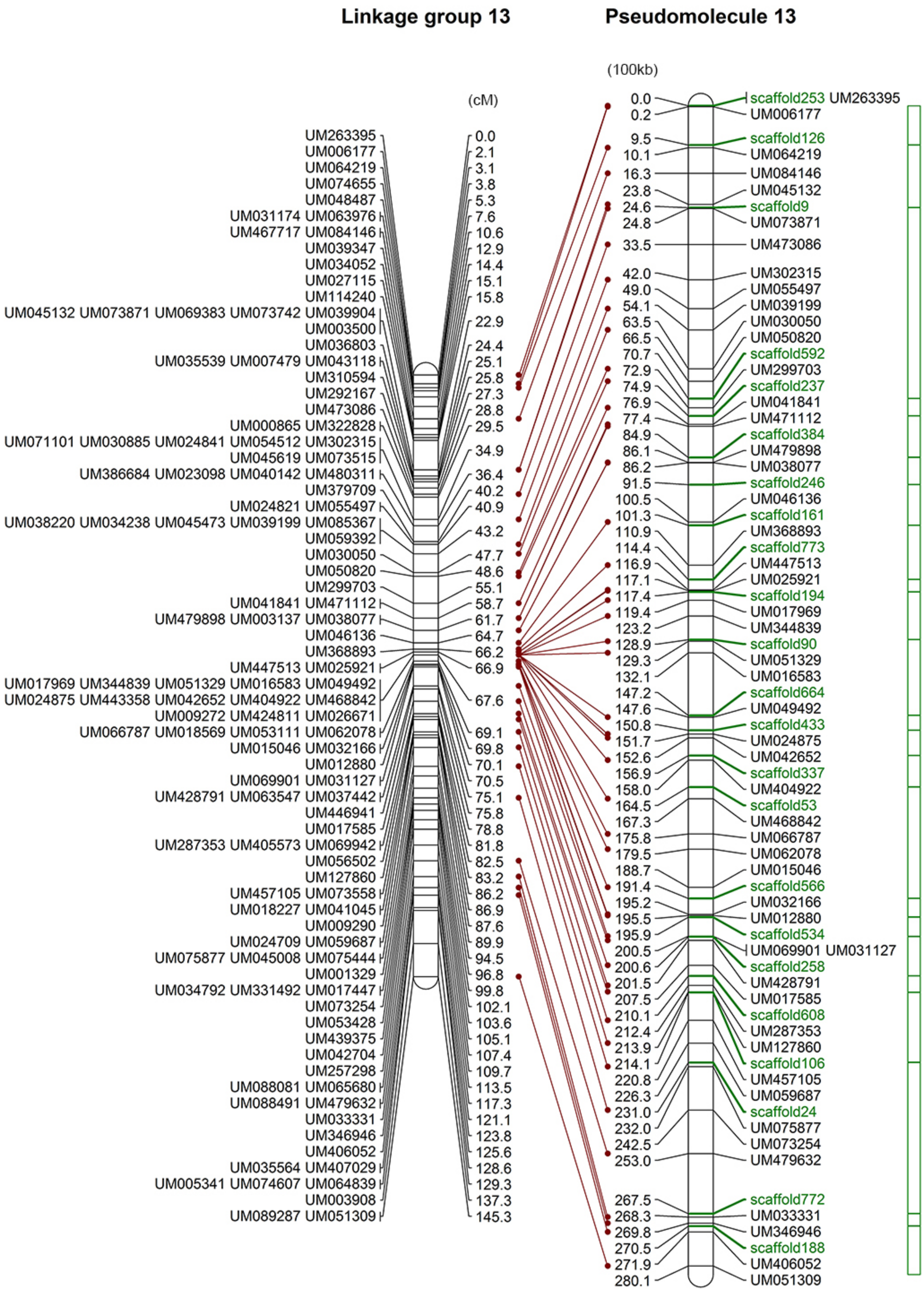
Linkage group 13
Pseudomolecule 13
(cM)
(100kb)
UM263395
UM006177
UM064219
UM074655
UM048487
UM031174 UM063976
UM467717 UM084146
UM039347
UM034052
UM027115
UM114240
UM045132 UM073871 UM069383 UM073742 UM039904
UM003500
UM036803
UM035539 UM007479 UM043118
UM310594
UM292167
UM473086
UM000865 UM322828
UM071101 UM030885 UM024841 UM054512 UM302315
UM045619 UM073515
UM386684 UM023098 UM040142 UM480311
UM379709
UM024821 UM055497
UM038220 UM034238 UM045473 UM039199 UM085367
UM059392
UM030050
UM050820
UM299703
UM041841 UM471112
UM479898 UM003137 UM038077
UM046136
UM368893
UM447513 UM025921
UM017969 UM344839 UM051329 UM016583 UM049492
UM024875 UM443358 UM042652 UM404922 UM468842
UM009272 UM424811 UM026671
UM066787 UM018569 UM053111 UM062078
UM015046 UM032166
UM012880
UM069901 UM031127
UM428791 UM063547 UM037442
UM446941
UM017585
UM287353 UM405573 UM069942
UM056502
UM127860
UM457105 UM073558
UM018227 UM041045
UM009290
UM024709 UM059687
UM075877 UM045008 UM075444
UM001329
UM034792 UM331492 UM017447
UM073254
UM053428
UM439375
UM042704
UM257298
UM088081 UM065680
UM088491 UM479632
UM033331
UM346946
UM406052
UM035564 UM407029
UM005341 UM074607 UM064839
UM003908
UM089287 UM051309
0.0
2.1
3.1
3.8
5.3
7.6
10.6
12.9
14.4
15.1
15.8
22.9
24.4
25.1
25.8
27.3
28.8
29.5
34.9
36.4
40.2
40.9
43.2
47.7
48.6
55.1
58.7
61.7
64.7
66.2
66.9
67.6
69.1
69.8
70.1
70.5
75.1
75.8
78.8
81.8
82.5
83.2
86.2
86.9
87.6
89.9
94.5
96.8
99.8
102.1
103.6
105.1
107.4
109.7
113.5
117.3
121.1
123.8
125.6
128.6
129.3
137.3
145.3
0.0 scaffold253 UM263395
0.2 UM006177
9.5 scaffold126
10.1 UM064219
16.3 UM084146
23.8 UM045132
24.6 scaffold9
24.8 UM073871
33.5 UM473086
42.0 UM302315
49.0 UM055497
54.1 UM039199
63.5 UM030050
66.5 UM050820
70.7 scaffold592
72.9 UM299703
74.9 scaffold237
76.9 UM041841
77.4 UM471112
84.9 scaffold384
86.1 UM479898
86.2 UM038077
91.5 scaffold246
100.5 UM046136
101.3 scaffold161
110.9 UM368893
114.4 scaffold773
116.9 UM447513
117.1 UM025921
117.4 scaffold194
119.4 UM017969
123.2 UM344839
128.9 scaffold90
129.3 UM051329
132.1 UM016583
147.2 scaffold664
147.6 UM049492
150.8 scaffold433
151.7 UM024875
152.6 UM042652
156.9 scaffold337
158.0 UM404922
164.5 scaffold53
167.3 UM468842
175.8 UM066787
179.5 UM062078
188.7 UM015046
191.4 scaffold566
195.2 UM032166
195.5 UM012880
195.9 scaffold534
200.5 UM069901 UM031127
200.6 scaffold258
201.5 UM428791
207.5 UM017585
210.1 scaffold608
212.4 UM287353
213.9 UM127860
214.1 scaffold106
220.8 UM457105
226.3 UM059687
231.0 scaffold24
232.0 UM075877
242.5 UM073254
253.0 UM479632
267.5 scaffold772
268.3 UM033331
269.8 UM346946
270.5 scaffold188
271.9 UM406052
280.1 UM051309

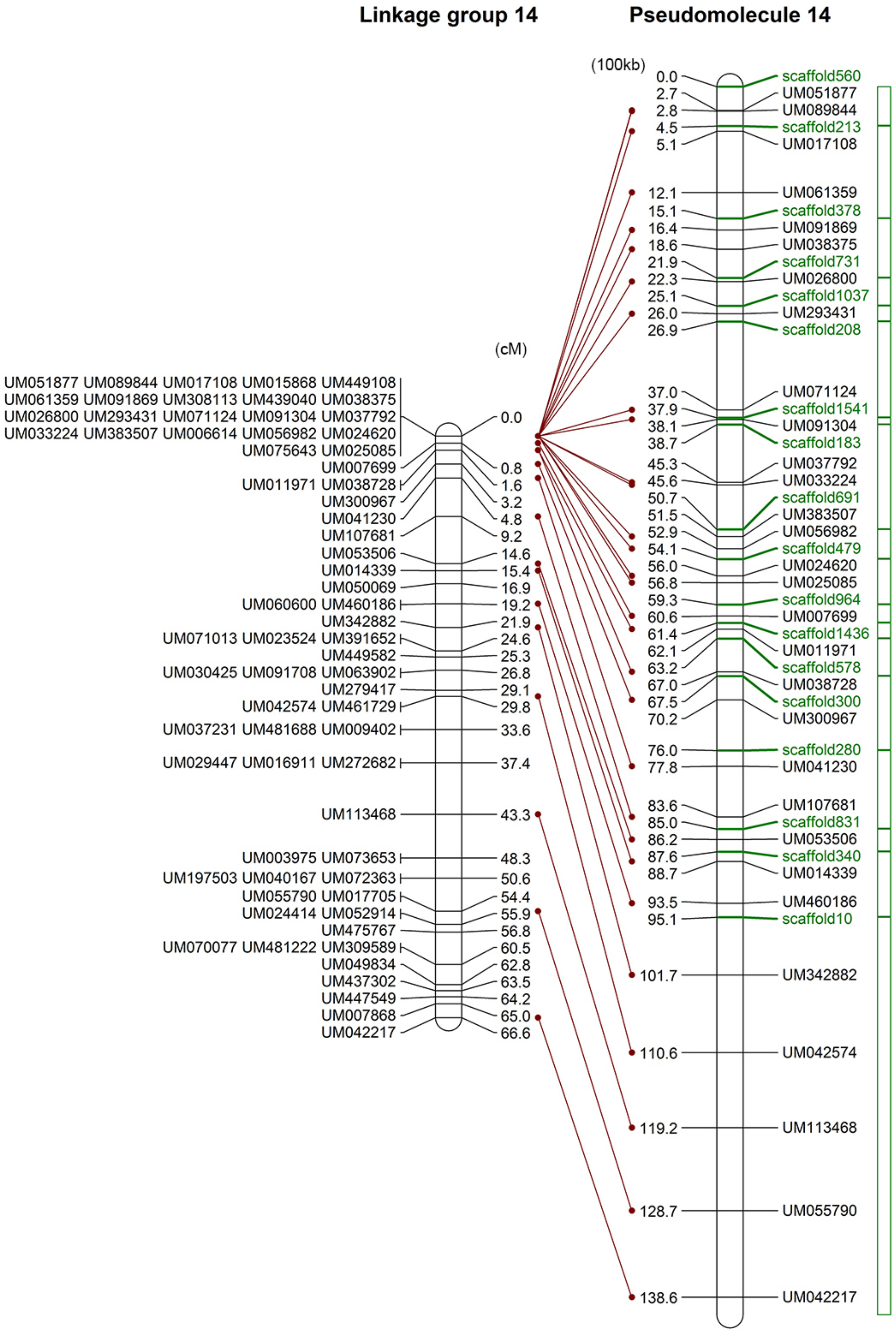

Linkage group 14
Pseudomolecule 14
(cM)
(100kb)
UM051877 UM089844 UM017108 UM015868 UM449108
UM061359 UM091869 UM308113 UM439040 UM038375
UM026800 UM293431 UM071124 UM091304 UM037792
UM033224 UM383507 UM006614 UM056982 UM024620
UM075643 UM025085
0.0
UM007699 0.8
UM011971 UM038728 1.6
UM300967 3.2
UM041230 4.8
UM107681 9.2
UM053506 14.6
UM014339 15.4
UM050069 16.9
UM060600 UM460186 19.2
UM342882 21.9
UM071013 UM023524 UM391652 24.6
UM449582 25.3
UM030425 UM091708 UM063902 26.8
UM279417 29.1
UM042574 UM461729 29.8
UM037231 UM481688 UM009402 33.6
UM029447 UM016911 UM272682 37.4
UM113468 43.3
UM003975 UM073653 48.3
UM197503 UM040167 UM072363 50.6
UM055790 UM017705 54.4
UM024414 UM052914 55.9
UM475767 56.8
UM070077 UM481222 UM309589 60.5
UM049834 62.8
UM437302 63.5
UM447549 64.2
UM007868 65.0
UM042217 66.6
0.0 scaffold560
2.7 UM051877
2.8 UM089844
4.5 scaffold213
5.1 UM017108
12.1 UM061359
15.1 scaffold378
16.4 UM091869
18.6 UM038375
21.9 scaffold731
22.3 UM026800
25.1 scaffold1037
26.0 UM293431
26.9 scaffold208
37.0 UM071124
37.9 scaffold1541
38.1 UM091304
38.7 scaffold183
45.3 UM037792
45.6 UM033224
50.7 scaffold691
51.5 UM383507
52.9 UM056982
54.1 scaffold479
56.0 UM024620
56.8 UM025085
59.3 scaffold964
60.6 UM007699
61.4 scaffold1436
62.1 UM011971
63.2 scaffold578
67.0 UM038728
67.5 scaffold300
70.2 UM300967
76.0 scaffold280
77.8 UM041230
83.6 UM107681
85.0 scaffold831
86.2 UM053506
87.6 scaffold340
88.7 UM014339
93.5 UM460186
95.1 scaffold10
101.7 UM342882
110.6 UM042574
119.2 UM113468
128.7 UM055790
138.6 UM042217

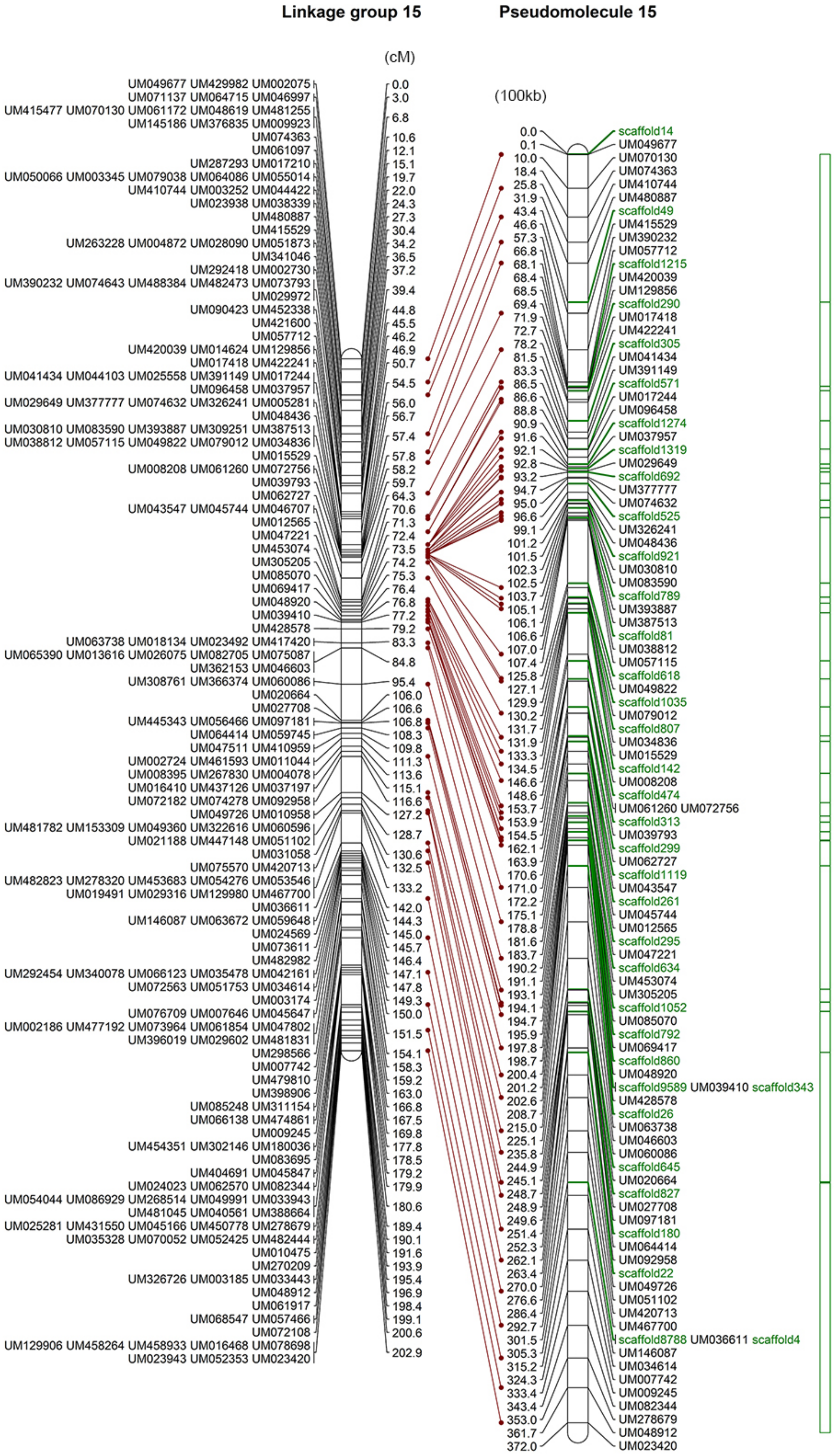

Linkage group 15
Pseudomolecule 15
(cM)
(100kb)
UM049677 UM429982 UM002075
UM071137 UM064715 UM046997
UM415477 UM070130 UM061172 UM048619 UM481255
UM145186 UM376835 UM009923
UM074363
UM061097
UM287293 UM017210
UM050066 UM003345 UM079038 UM064086 UM055014
UM410744 UM003252 UM044422
UM023938 UM038339
UM480887
UM415529
UM263228 UM004872 UM028090 UM051873
UM341046
UM292418 UM002730
UM390232 UM074643 UM488384 UM482473 UM073793
UM029972
UM090423 UM452338
UM421600
UM057712
UM420039 UM014624 UM129856
UM017418 UM422241
UM041434 UM044103 UM025558 UM391149 UM017244
UM096458 UM037957
UM029649 UM377777 UM074632 UM326241 UM005281
UM048436
UM030810 UM083590 UM393887 UM309251 UM387513
UM038812 UM057115 UM049822 UM079012 UM034836
UM015529
UM008208 UM061260 UM072756
UM039793
UM062727
UM043547 UM045744 UM046707
UM012565
UM047221
UM453074
UM305205
UM085070
UM069417
UM048920
UM039410
UM428578
UM063738 UM018134 UM023492 UM417420
UM065390 UM013616 UM026075 UM082705 UM075087
UM362153 UM046603
UM308761 UM366374 UM060086
UM020664
UM027708
UM445343 UM056466 UM097181
UM064414 UM059745
UM047511 UM410959
UM002724 UM461593 UM011044
UM008395 UM267830 UM004078
UM016410 UM437126 UM037197
UM072182 UM074278 UM092958
UM049726 UM010958
UM481782 UM153309 UM049360 UM322616 UM060596
UM021188 UM447148 UM051102
UM031058
UM075570 UM420713
UM482823 UM278320 UM453683 UM054276 UM053546
UM019491 UM029316 UM129980 UM467700
UM036611
UM146087 UM063672 UM059648
UM024569
UM073611
UM482982
UM292454 UM340078 UM066123 UM035478 UM042161
UM072563 UM051753 UM034614
UM003174
UM076709 UM007646 UM045647
UM002186 UM477192 UM073964 UM061854 UM047802
UM396019 UM029602 UM481831
UM298566
UM007742
UM479810
UM398906
UM085248 UM311154
UM066138 UM474861
UM009245
UM454351 UM302146 UM180036
UM083695
UM404691 UM045847
UM024023 UM062570 UM082344
UM054044 UM086929 UM268514 UM049991 UM033943
UM481045 UM040561 UM388664
UM025281 UM431550 UM045166 UM450778 UM278679
UM035328 UM070052 UM052425 UM482444
UM010475
UM270209
UM326726 UM003185 UM033443
UM048912
UM061917
UM068547 UM057466
UM072108
UM129906 UM458264 UM458933 UM016468 UM078698
UM023943 UM052353 UM023420
0.0
3.0
6.8
10.6
12.1
15.1
19.7
22.0
24.3
27.3
30.4
34.2
36.5
37.2
39.4
44.8
45.5
46.2
46.9
50.7
54.5
56.0
56.7
57.4
57.8
58.2
59.7
64.3
70.6
71.3
72.4
73.5
74.2
75.3
76.4
76.8
77.2
79.2
83.3
84.8
95.4
106.0
106.6
106.8
108.3
109.8
111.3
113.6
115.1
116.6
127.2
128.7
130.6
132.5
133.2
142.0
144.3
145.0
145.7
146.4
147.1
147.8
149.3
150.0
151.5
154.1
158.3
159.2
163.0
166.8
167.5
169.8
177.8
178.5
179.2
179.9
180.6
189.4
190.1
191.6
193.9
195.4
196.9
198.4
199.1
200.6
202.9
0.0
0.1
10.0
18.4
25.8
31.9
43.4
46.6
57.3
66.8
68.1
68.4
68.5
69.4
71.9
72.7
78.2
81.5
83.3
86.5
86.6
88.8
90.9
91.6
92.1
92.8
93.2
94.7
95.0
96.6
99.1
101.2
101.5
102.3
102.5
103.7
105.1
106.1
106.6
107.0
107.4
125.8
127.1
129.9
130.2
131.7
131.9
133.3
134.5
146.6
148.6
153.7
153.9
154.5
162.1
163.9
170.6
171.0
172.2
175.1
178.8
181.6
183.7
190.2
191.1
193.1
194.1
194.7
195.9
197.8
198.7
200.4
201.2
202.6
208.7
215.0
225.1
235.8
244.9
245.1
248.7
248.9
249.6
251.4
252.3
262.1
263.4
270.0
276.6
286.4
292.7
301.5
305.3
315.2
324.3
333.4
343.4
353.0
361.7
372.0
scaffold14
UM049677
UM070130
UM074363
UM410744
UM480887
scaffold49
UM415529
UM390232
UM057712
scaffold1215
UM420039
UM129856
scaffold290
UM017418
UM422241
scaffold305
UM041434
UM391149
scaffold571
UM017244
UM096458
scaffold1274
UM037957
scaffold1319
UM029649
scaffold692
UM377777
UM074632
scaffold525
UM326241
UM048436
scaffold921
UM030810
UM083590
scaffold789
UM393887
UM387513
scaffold81
UM038812
UM057115
scaffold618
UM049822
scaffold1035
UM079012
scaffold807
UM034836
UM015529
scaffold142
UM008208
scaffold474
UM061260 UM072756
scaffold313
UM039793
scaffold299
UM062727
scaffold1119
UM043547
scaffold261
UM045744
UM012565
scaffold295
UM047221
scaffold634
UM453074
UM305205
scaffold1052
UM085070
scaffold792
UM069417
scaffold860
UM048920
scaffold9589 UM039410 scaffold343
UM428578
scaffold26
UM063738
UM046603
UM060086
scaffold645
UM020664
scaffold827
UM027708
UM097181
scaffold180
UM064414
UM092958
scaffold22
UM049726
UM051102
UM420713
UM467700
scaffold8788 UM036611 scaffold4
UM146087
UM034614
UM007742
UM009245
UM082344
UM278679
UM048912
UM023420

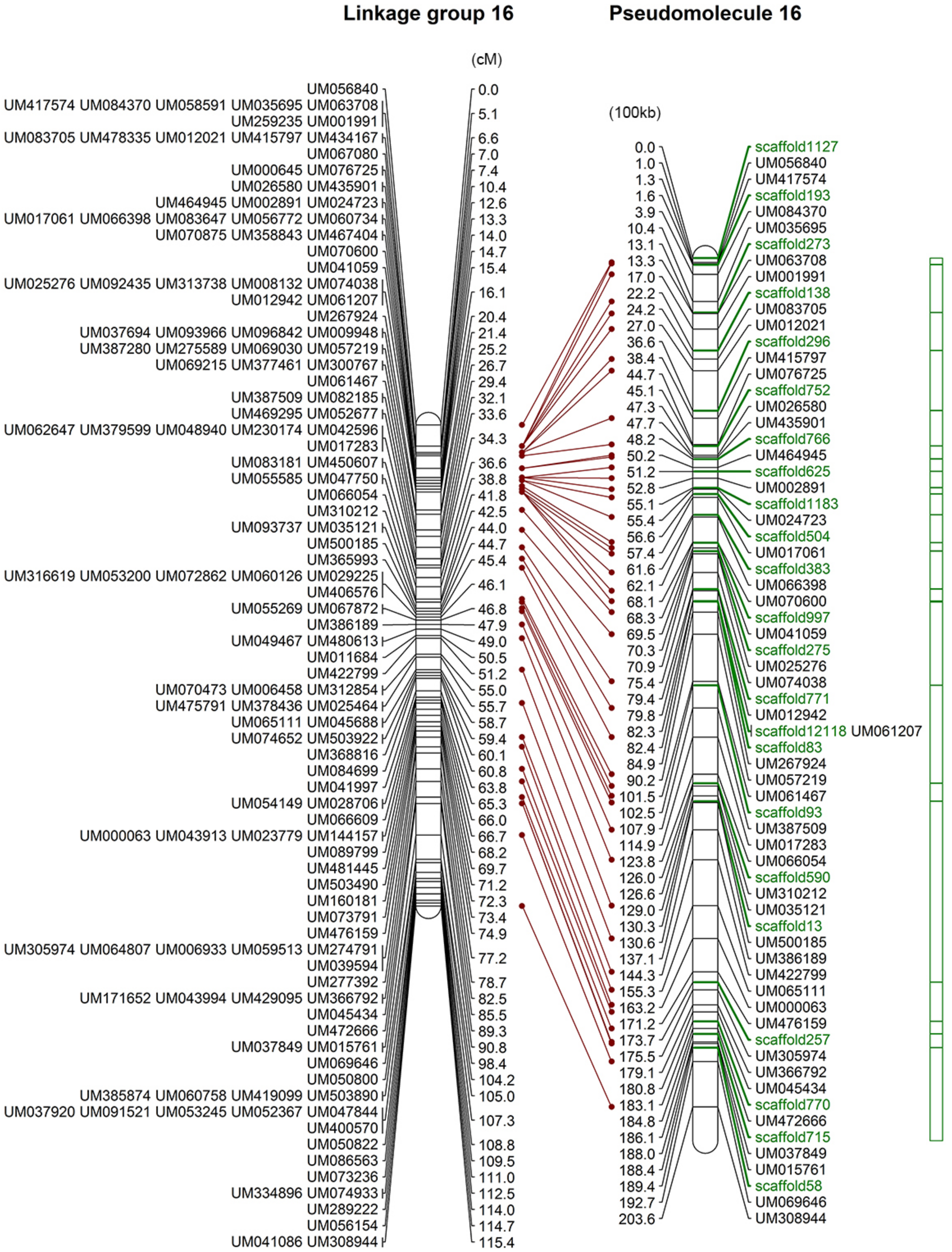
Linkage group 16
Pseudomolecule 16
(cM)
UM056840 0.0
UM417574 UM084370 UM058591 UM035695 UM063708
UM259235 UM001991 5.1
UM083705 UM478335 UM012021 UM415797 UM434167 6.6
UM067080 7.0
UM000645 UM076725 7.4
UM026580 UM435901 10.4
UM464945 UM002891 UM024723 12.6
UM017061 UM066398 UM083647 UM056772 UM060734 13.3
UM070875 UM358843 UM467404 14.0
UM070600 14.7
UM041059 15.4
UM025276 UM092435 UM313738 UM008132 UM074038
UM012942 UM061207 16.1
UM267924 20.4
UM037694 UM093966 UM096842 UM009948 21.4
UM387280 UM275589 UM069030 UM057219 25.2
UM069215 UM377461 UM300767 26.7
UM061467 29.4
UM387509 UM082185 32.1
UM469295 UM052677 33.6
UM062647 UM379599 UM048940 UM230174 UM042596
UM017283 34.3
UM083181 UM450607 36.6
UM055585 UM047750 38.8
UM066054 41.8
UM310212 42.5
UM093737 UM035121 44.0
UM500185 44.7
UM365993 45.4
UM316619 UM053200 UM072862 UM060126 UM029225
UM406576 46.1
UM055269 UM067872 46.8
UM386189 47.9
UM049467 UM480613 49.0
UM011684 50.5
UM422799 51.2
UM070473 UM006458 UM312854 55.0
UM475791 UM378436 UM025464 55.7
UM065111 UM045688 58.7
UM074652 UM503922 59.4
UM368816 60.1
UM084699 60.8
UM041997 63.8
UM054149 UM028706 65.3
UM066609 66.0
UM000063 UM043913 UM023779 UM144157 66.7
UM089799 68.2
UM481445 69.7
UM503490 71.2
UM160181 72.3
UM073791 73.4
UM476159 74.9
UM305974 UM064807 UM006933 UM059513 UM274791
UM039594 77.2
UM277392 78.7
UM171652 UM043994 UM429095 UM366792 82.5
UM045434 85.5
UM472666 89.3
UM037849 UM015761 90.8
UM069646 98.4
UM050800 104.2
UM385874 UM060758 UM419099 UM503890 105.0
UM037920 UM091521 UM053245 UM052367 UM047844
UM400570 107.3
UM050822 108.8
UM086563 109.5
UM073236 111.0
UM334896 UM074933 112.5
UM289222 114.0
UM056154 114.7
UM041086 UM308944 115.4
(100kb)
0.0 scaffold1127
1.0 UM056840
1.3 UM417574
1.6 scaffold193
3.9 UM084370
10.4 UM035695
13.1 scaffold273
13.3 UM063708
17.0 UM001991
22.2 scaffold138
24.2 UM083705
27.0 UM012021
36.6 scaffold296
38.4 UM415797
44.7 UM076725
45.1 scaffold752
47.3 UM026580
47.7 UM435901
48.2 scaffold766
50.2 UM464945
51.2 scaffold625
52.8 UM002891
55.1 scaffold1183
55.4 UM024723
56.6 scaffold504
57.4 UM017061
61.6 scaffold383
62.1 UM066398
68.1 UM070600
68.3 scaffold997
69.5 UM041059
70.3 scaffold275
70.9 UM025276
75.4 UM074038
79.4 scaffold771
79.8 UM012942
82.3 scaffold12118 UM061207
82.4 scaffold83
84.9 UM267924
90.2 UM057219
101.5 UM061467
102.5 scaffold93
107.9 UM387509
114.9 UM017283
123.8 UM066054
126.0 scaffold590
126.6 UM310212
129.0 UM035121
130.3 scaffold13
130.6 UM500185
137.1 UM386189
144.3 UM422799
155.3 UM065111
163.2 UM000063
171.2 UM476159
173.7 scaffold257
175.5 UM305974
179.1 UM366792
180.8 UM045434
183.1 scaffold770
184.8 UM472666
186.1 scaffold715
188.0 UM037849
188.4 UM015761
189.4 scaffold58
192.7 UM069646
203.6 UM308944

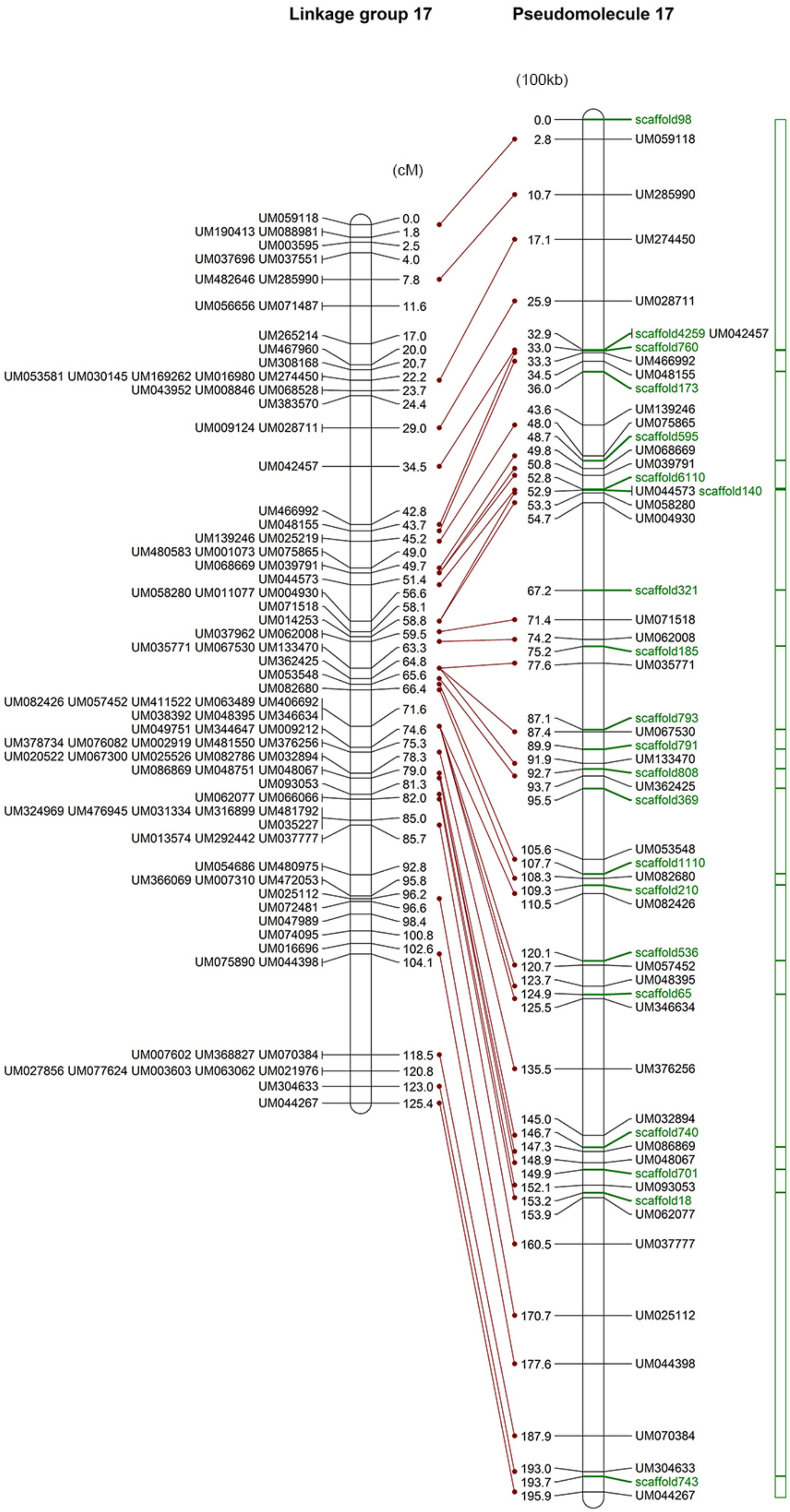

Linkage group 17
Pseudomolecule 17
(100kb)
(cM)
UM059118 0.0
UM190413 UM088981 1.8
UM003595 2.5
UM037696 UM037551 4.0
UM482646 UM285990 7.8
UM056656 UM071487 11.6
UM265214 17.0
UM467960 20.0
UM308168 20.7
UM053581 UM030145 UM169262 UM016980 UM274450 22.2
UM043952 UM008846 UM068528 23.7
UM383570 24.4
UM009124 UM028711 29.0
UM042457 34.5
UM466992 42.8
UM048155 43.7
UM139246 UM025219 45.2
UM480583 UM001073 UM075865 49.0
UM068669 UM039791 49.7
UM044573 51.4
UM058280 UM011077 UM004930 56.6
UM071518 58.1
UM014253 58.8
UM037962 UM062008 59.5
UM035771 UM067530 UM133470 63.3
UM362425 64.8
UM053548 65.6
UM082680 66.4
UM082426 UM057452 UM411522 UM063489 UM406692
UM038392 UM048395 UM346634 71.6
UM049751 UM344647 UM009212 74.6
UM378734 UM076082 UM002919 UM481550 UM376256 75.3
UM020522 UM067300 UM025526 UM082786 UM032894 78.3
UM086869 UM048751 UM048067 79.0
UM093053 81.3
UM062077 UM066066 82.0
UM324969 UM476945 UM031334 UM316899 UM481792
UM035227 85.0
UM013574 UM292442 UM037777 85.7
UM054686 UM480975 92.8
UM366069 UM007310 UM472053 95.8
UM025112 96.2
UM072481 96.6
UM047989 98.4
UM074095 100.8
UM016696 102.6
UM075890 UM044398 104.1
UM007602 UM368827 UM070384 118.5
UM027856 UM077624 UM003603 UM063062 UM021976 120.8
UM304633 123.0
UM044267 125.4
0.0 scaffold98
2.8 UM059118
10.7 UM285990
17.1 UM274450
25.9 UM028711
32.9 scaffold4259 UM042457
33.0 scaffold760
33.3 UM466992
34.5 UM048155
36.0 scaffold173
43.6 UM139246
48.0 UM075865
48.7 scaffold595
49.8 UM068669
50.8 UM039791
52.8 scaffold6110
52.9 UM044573 scaffold140
53.3 UM058280
54.7 UM004930
67.2 scaffold321
71.4 UM071518
74.2 UM062008
75.2 scaffold185
77.6 UM035771
87.1 scaffold793
87.4 UM067530
89.9 scaffold791
91.9 UM133470
92.7 scaffold808
93.7 UM362425
95.5 scaffold369
105.6 UM053548
107.7 scaffold1110
108.3 UM082680
109.3 scaffold210
110.5 UM082426
120.1 scaffold536
120.7 UM057452
123.7 UM048395
124.9 scaffold65
125.5 UM346634
135.5 UM376256
145.0 UM032894
146.7 scaffold740
147.3 UM086869
148.9 UM048067
149.9 scaffold701
152.1 UM093053
153.2 scaffold18
153.9 UM062077
160.5 UM037777
170.7 UM025112
177.6 UM044398
187.9 UM070384
193.0 UM304633
193.7 scaffold743
195.9 UM044267

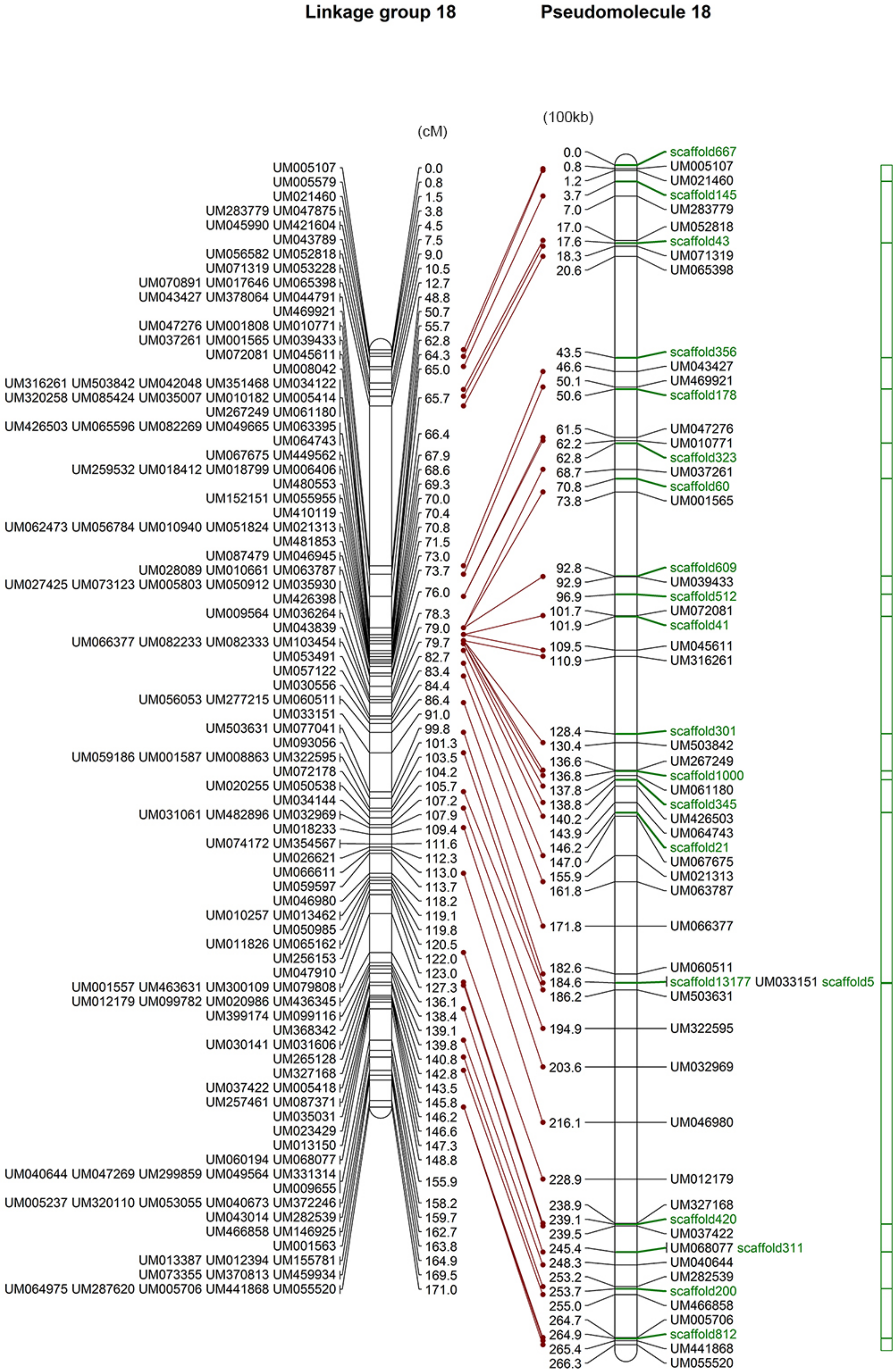

Linkage group 18
Pseudomolecule 18
(cM)
(100kb)
UM005107 0.0
UM005579 0.8
UM021460 1.5
UM283779 UM047875 3.8
UM045990 UM421604 4.5
UM043789 7.5
UM056582 UM052818 9.0
UM071319 UM053228 10.5
UM070891 UM017646 UM065398 12.7
UM043427 UM378064 UM044791 48.8
UM469921 50.7
UM047276 UM001808 UM010771 55.7
UM037261 UM001565 UM039433 62.8
UM072081 UM045611 64.3
UM008042 65.0
UM316261 UM503842 UM042048 UM351468 UM034122
UM320258 UM085424 UM035007 UM010182 UM005414 65.7
UM267249 UM061180
UM426503 UM065596 UM082269 UM049665 UM063395
UM064743 66.4
UM067675 UM449562 67.9
UM259532 UM018412 UM018799 UM006406 68.6
UM480553 69.3
UM152151 UM055955 70.0
UM410119 70.4
UM062473 UM056784 UM010940 UM051824 UM021313 70.8
UM481853 71.5
UM087479 UM046945 73.0
UM028089 UM010661 UM063787 73.7
UM027425 UM073123 UM005803 UM050912 UM035930
UM426398 76.0
UM009564 UM036264 78.3
UM043839 79.0
UM066377 UM082233 UM082333 UM103454 79.7
UM053491 82.7
UM057122 83.4
UM030556 84.4
UM056053 UM277215 UM060511 86.4
UM033151 91.0
UM503631 UM077041 99.8
UM093056 101.3
UM059186 UM001587 UM008863 UM322595 103.5
UM072178 104.2
UM020255 UM050538 105.7
UM034144 107.2
UM031061 UM482896 UM032969 107.9
UM018233 109.4
UM074172 UM354567 111.6
UM026621 112.3
UM066611 113.0
UM059597 113.7
UM046980 118.2
UM010257 UM013462 119.1
UM050985 119.8
UM011826 UM065162 120.5
UM256153 122.0
UM047910 123.0
UM001557 UM463631 UM300109 UM079808 127.3
UM012179 UM099782 UM020986 UM436345 136.1
UM399174 UM099116 138.4
UM368342 139.1
UM030141 UM031606 139.8
UM265128 140.8
UM327168 142.8
UM037422 UM005418 143.5
UM257461 UM087371 145.8
UM035031 146.2
UM023429 146.6
UM013150 147.3
UM060194 UM068077 148.8
UM040644 UM047269 UM299859 UM049564 UM331314
UM009655 155.9
UM005237 UM320110 UM053055 UM040673 UM372246 158.2
UM043014 UM282539 159.7
UM466858 UM146925 162.7
UM001563 163.8
UM013387 UM012394 UM155781 164.9
UM073355 UM370813 UM459934 169.5
UM064975 UM287620 UM005706 UM441868 UM055520 171.0
0.0 scaffold667
0.8 UM005107
1.2 UM021460
3.7 scaffold145
7.0 UM283779
17.0 UM052818
17.6 scaffold43
18.3 UM071319
20.6 UM065398
43.5 scaffold356
46.6 UM043427
50.1 UM469921
50.6 scaffold178
61.5 UM047276
62.2 UM010771
62.8 scaffold323
68.7 UM037261
70.8 scaffold60
73.8 UM001565
92.8 scaffold609
92.9 UM039433
96.9 scaffold512
101.7 UM072081
101.9 scaffold41
109.5 UM045611
110.9 UM316261
128.4 scaffold301
130.4 UM503842
136.6 UM267249
136.8 scaffold1000
137.8 UM061180
138.8 scaffold345
140.2 UM426503
143.9 UM064743
146.2 scaffold21
147.0 UM067675
155.9 UM021313
161.8 UM063787
171.8 UM066377
182.6 UM060511
184.6 scaffold13177 UM033151 scaffold5
186.2 UM503631
194.9 UM322595
203.6 UM032969
216.1 UM046980
228.9 UM012179
238.9 UM327168
239.1 scaffold420
239.5 UM037422
245.4 UM068077 scaffold311
248.3 UM040644
253.2 UM282539
253.7 scaffold200
255.0 UM466858
264.7 UM005706
264.9 scaffold812
265.4 UM441868
266.3 UM055520

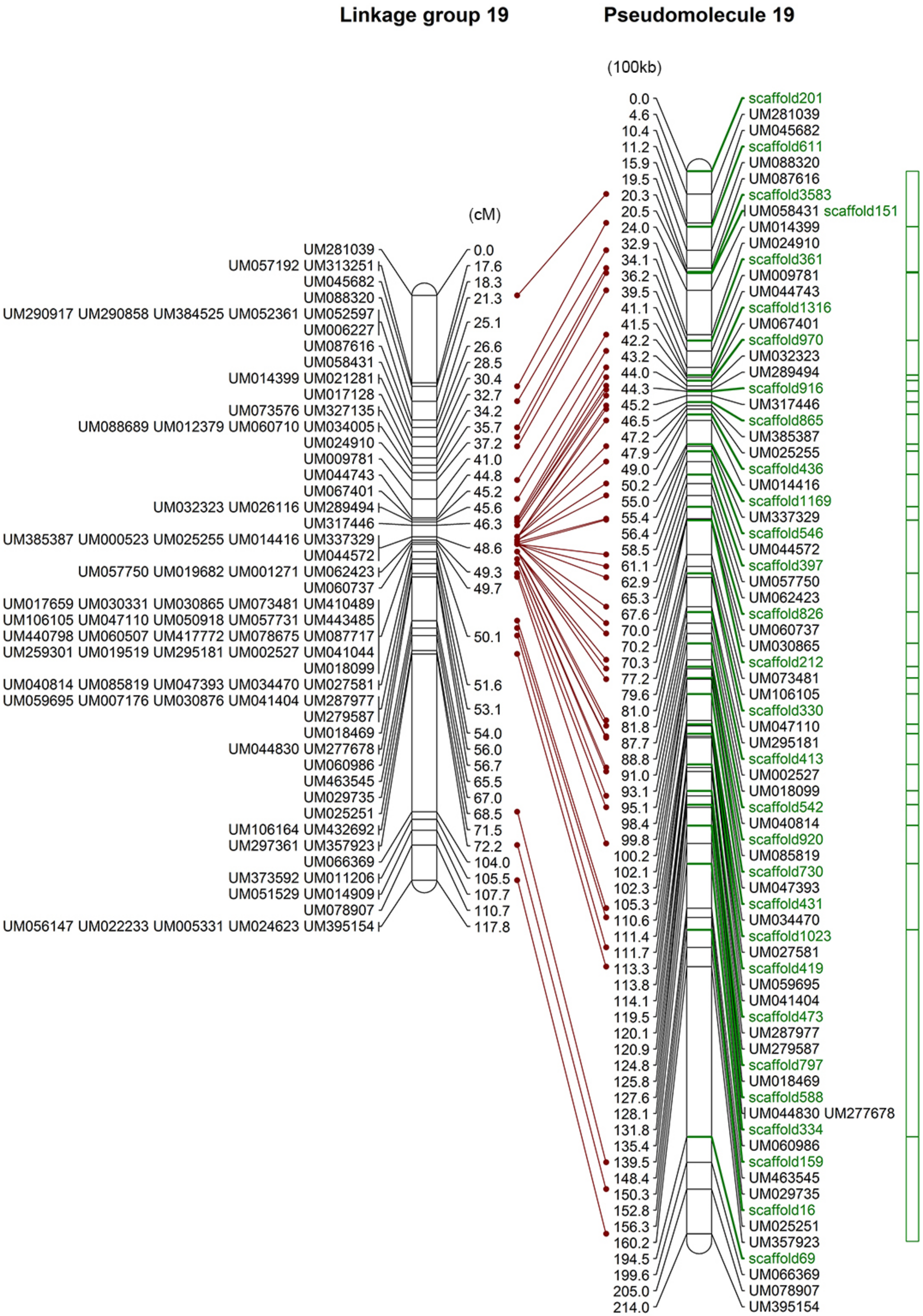

Linkage group 19
Pseudomolecule 19
(cM)
UM281039 0.0
UM057192 UM313251 17.6
UM045682 18.3
UM088320 21.3
UM290917 UM290858 UM384525 UM052361 UM052597
UM006227 25.1
UM087616 26.6
UM058431 28.5
UM014399 UM021281 30.4
UM017128 32.7
UM073576 UM327135 34.2
UM088689 UM012379 UM060710 UM034005 35.7
UM024910 37.2
UM009781 41.0
UM044743 44.8
UM067401 45.2
UM032323 UM026116 UM289494 45.6
UM317446 46.3
UM385387 UM000523 UM025255 UM014416 UM337329
UM044572 48.6
UM057750 UM019682 UM001271 UM062423 49.3
UM060737 49.7
UM017659 UM030331 UM030865 UM073481 UM410489
UM106105 UM047110 UM050918 UM057731 UM443485
UM440798 UM060507 UM417772 UM078675 UM087717 50.1
UM259301 UM019519 UM295181 UM002527 UM041044
UM018099
UM040814 UM085819 UM047393 UM034470 UM027581 51.6
UM059695 UM007176 UM030876 UM041404 UM287977
UM279587 53.1
UM018469 54.0
UM044830 UM277678 56.0
UM060986 56.7
UM463545 65.5
UM029735 67.0
UM025251 68.5
UM106164 UM432692 71.5
UM297361 UM357923 72.2
UM066369 104.0
UM373592 UM011206 105.5
UM051529 UM014909 107.7
UM078907 110.7
UM056147 UM022233 UM005331 UM024623 UM395154 117.8
(100kb)
0.0 scaffold201
4.6 UM281039
10.4 UM045682
11.2 scaffold611
15.9 UM088320
19.5 UM087616
20.3 scaffold3583
20.5 UM058431 scaffold151
24.0 UM014399
32.9 UM024910
34.1 scaffold361
36.2 UM009781
39.5 UM044743
41.1 scaffold1316
41.5 UM067401
42.2 scaffold970
43.2 UM032323
44.0 UM289494
44.3 scaffold916
45.2 UM317446
46.5 scaffold865
47.2 UM385387
47.9 UM025255
49.0 scaffold436
50.2 UM014416
55.0 scaffold1169
55.4 UM337329
56.4 scaffold546
58.5 UM044572
61.1 scaffold397
62.9 UM057750
65.3 UM062423
67.6 scaffold826
70.0 UM060737
70.2 UM030865
70.3 scaffold212
77.2 UM073481
79.6 UM106105
81.0 scaffold330
81.8 UM047110
87.7 UM295181
88.8 scaffold413
91.0 UM002527
93.1 UM018099
95.1 scaffold542
98.4 UM040814
99.8 scaffold920
100.2 UM085819
102.1 scaffold730
102.3 UM047393
105.3 scaffold431
110.6 UM034470
111.4 scaffold1023
111.7 UM027581
113.3 scaffold419
113.8 UM059695
114.1 UM041404
119.5 scaffold473
120.1 UM287977
120.9 UM279587
124.8 scaffold797
125.8 UM018469
127.6 scaffold588
128.1 UM044830 UM277678
131.8 scaffold334
135.4 UM060986
139.5 scaffold159
148.4 UM463545
150.3 UM029735
152.8 scaffold16
156.3 UM025251
160.2 UM357923
194.5 scaffold69
199.6 UM066369
205.0 UM078907
214.0 UM395154
