# Supplementary material for: Chromosome‐level assembly, genetic and physical mapping of Phalaenopsis aphrodite genome provides new insights into species adaptation and resources for orchid breeding
Source: Plant Biotechnol J. 2018 May 23;16(12):2027–41. doi: 10.1111/pbi.12936 (PMC6230949; doi:10.1111/pbi.12936)
Supplement: Supplementary file 3 — Table S5 List of FISH probes. Table S6 Relative positions of FISH probes on pachytene chromosomes. Table S7 Coordinates of recombination hot spots, list of genes within the hot spots and gene density. Table S8 Annotation of genes within the recombination hot spots. Table S9 List of recombination cold spots and gene density. Table S10 List of genes within the cold hot spots and gene annotation. Table S11 Orchidstra 2.0 ESTs that map to P. aphrodite genome. Table S12 Annotated protein‐coding genes in P. aphrodite. Table S13 List of transcription factors in P. aphrodite. Table S14 List of transporters in P. aphrodite. Table S15 The number and type of non‐coding RNAs in P. aphrodite. Table S16 The number of genes in shared and species‐specific gene families in ten representative plant species. Table S17 Number of MADS‐box genes in different species. Table S18 Number of flavonoid biosynthesis‐related genes in different species. Table S19 Number of carotenoid biosynthesis‐related genes in different species. Table S20 Annotation of color, scent and flowering genes. Table S21 Differential expression analysis with DESeq2 for comparison between the tissues in P. aphrodite. Table S22 Differential expression analysis with DESeq2 for comparison between the tissues in P. lueddemanniana. Table S23 Using inkage group‐specific clones to identify the misassembled scaffolds in P. equestris draft genome. Appendix S1 This file contains Supporting Text, Figures S1–S26, and Tables S1–S4, S11–S12, S15–S19. [file PBI-16-2027-s001.pdf]

Table S5 List of FISH probes

Marker	Clone name	Forward primer (5'-3')	Reverse primer (5'-3')	Amplicon size (kb)
DL14-S43	DL14-S43-1	cattcgcacatcactccaac	ttgcgaatatcacggaattg	2.7
	DL14-S43-2	gccaaatcacgtaaatctgtgt	agcaaaagcctcaccgttta	2.2
	DL14-S43-3	cgaagccatttacatcttcttaca	gggggttaaaatcgctacatg	3.7
	DL14-S43-4	tttaccgatgtgattttcattgtt	cccaacccactaaattccaag	2.8
DL14-S80	DL14-S80-1	ccggaggcttaaactcggaga	ttccagccttgttgctgctc	3.6
	DL14-S80-2	agtggtgaggttttccccca	gaaacacgcatggatgcaca	3.4
	DL14-S80-3	aaatgttgctgagtcactaggacga	ccccgacttggtcaacactg	2.5
DL14-S8	DL14-S8-1	ttttggttctgccactggat	aacggaattggattgtacgc	2.6
	DL14-S8-2	tattggtacatcttacacggttgg	attgagcggcgtagttctgt	3.0
	DL14-S8-3	cctttatcacgcatcaaaacc	gctttgccattcagttggtt	2.0
	DL14-S8-4	ccaaagagcagtcgagcaat	aaatcagagcaccttttgatcc	3.8
DL14-S308	DL14-S308-1	ccagagggcgatttatcaaact	gacggtgtaaactgccgtcata	3.1
	DL14-S308-2	cgagacaccactagtccatgattt	cgcgtatccaccagacactaaa	3.3
	DL14-S308-3	cattactagccccttccatca	tcaaacaattttaaagttcgaatgtc	2.7
DL08-S176	DL08-S176-1	tgaatggctagacgagagaagcaa	catggatttgcccaccaaga	4.1
	DL08-S176-2	caaacaaacccttaatcggaag	tgtgagcatcccaaacaaaagg	3.6
	DL08-S176-3	gtaagcacattgccaaaccttgt	tcctgctccaattcagtcca	3.5
DL08-S535	DL08-S535-1	tgtaatcgcaggctatgatgacc	ccgccaagaaacaaaattaccc	3.9
	DL08-S535-2	ttctagggaacaccttgattctgc	cagaaatggaagagccatttatca	3.4
	DL08-S535-3	caattcgagcggtagatgagtct	gactgtcgttggttggattacga	3.4
DL08-S19	DL08-S19-1	aacacttgttggcatgttcgg	tgacgggaaaatggagactgg	2.5
	DL08-S19-2	ccgcttcaatcatctctcctaaaa	aatttggagctggcaggtatttt	4.1
	DL08-S19-3	gacttgttgttagggatttacttaggc	ctaggttagcatcaatttcggtga	3.8
DL05-S874	DL05-S874-1	ttcacaagggtggcattctc	tatggctgcaaggaaaagga	3.9
	DL05-S874-2	ccaaaacccttaactttctccc	aaggccatttgggatctttt	3.0
	DL05-S874-3	atgtttgtgatttggagcttttc	ggcctgcatggaagtcatta	4.0
DL05-S830	DL05-S830-1	gagcgaaggtagatggctgaa	tcaagtctcaggtcgggtcat	4.6
	DL05-S830-2	cccgaaattggattgatttg	tttacctgtcacaagatgggaga	4.3
	DL05-S830-3	aaggcttcgagcctattcaaaa	atcccctggttttgagaagc	4.3
DL05-S9	DL05-S9-1	ccccttgtaacacatctccttg	tgaagagctgaggccaagtt	2.1
	DL05-S9-2	aatgccacaccgttgatgtt	tgaccttcaccaagccagat	4.0
	DL05-S9-3	agggctgaagtccaaatgct	ccggaggtaacatttttctaaca	4.4
DL05-S347	DL05-S347-1	tggtagcaagttttgatatttgct	aaacgtcgcgttcaaatacc	3.1
	DL05-S347-2	ttaagtccccctgtcaatcg	cgaaactcaatgcaagtcca	3.9
	DL05-S347-3	cacgttggagggaatcactt	tttggcacacgaggaagaat	4.0
DL05-S271	DL05-S271-1	catttcgattggatagccct	taaatgtcggggacctgaac	3.7
	DL05-S271-2	aacttggcccccacattatt	cggttcggatgaactctagc	3.8
	DL05-S271-3	tatccagtttgttggcatcg	tccaatgccagtctcaaatg	3.9
DL05-S240	DL05-S240-1	cgccaaacagacccttagtg	ggagaaattcagtgtagcaaagc	2.5
	DL05-S240-2	ggtttcttgtttgggtcgaa	tcaaacactgagagccaaacc	4.0
	DL05-S240-3	cttgggctcagggtgaacta	gaaatgtggcaggtttcgat	5.0
DL12-S18	DL12-S18-1	catttcgatgtgcgtcgtta	cccaatccactgagtctcca	3.6
	DL12-S18-2	tgctaagctgcagactttgtttt	aaataaacttggccacataatagttga	3.2
	DL12-S18-3	tgtttcacacacatttgctctattg	cgcttaaatctttcagcaatatcaa	3.1
DL12-S319	DL12-S319-1	cctggagctaaaattcctcctc	tccactcatgatttggctga	3.5
	DL12-S319-2	ccccgctgtcactatcatct	ggctttcgatggttcctctt	3.9
	DL12-S319-3	taggtgtcaccacaggagca	ggtgcaggagtaacgcatct	2.9
DL12-S2	DL12-S2-1	tcagttgccacgataggtca	cttaaacgggcacttggtgt	3.6
	DL12-S2-2	tatggactctcgaccggaag	gatatccttcattcttaatgctgacat	3.5
	DL12-S2-3	caccagtgagaacgcttgaa	aatgatggccgtaacaaacaa	3.3
DL07-S1768	DL07-S1768-1	ttcgcaaacaaataacaaatgga	gcatctgcccatacttgccta	3.7
	DL07-S1768-2	tggcctaaagattgaagggga	ggggtgagtcattttggttttg	3.3
	DL07-S1768-3	cttcccaagaatttattcatccca	tccgcctccactaataatgc	4.4
DL07-S84	DL07-S84-1	caatcccgatcagggagttttag	gagaagggggattagggtttga	3.1
	DL07-S84-2	caacactctacattcgggggaa	tgtccaaccaacccatttttg	4.0
	DL07-S84-3	tttcaaacgcacaaaccaaaca	tgacattcttgcgtgctttttt	3.1
DL07-S64	DL07-S64-1	gaaaacaacaaacatgaaacaacagtag	accctcggatctttgtagcca	3.6

	DL07-S64-2	taggatgctctggaccatcactt	ctcaatgcaaatctccttgcac	3.7
	DL07-S64-3	tgtctgctgccttagtttgtgc	tggattctgcaagtggtttgagt	3.8
DL15-S120	DL15-S120-1	gcacccttttcttccatttttc	gtaattcgtgatctttgttcataattgaa	3.5
	DL15-S120-2	ggggctagggttggtttagatt	tcacccacctgacaagaggatg	4.0
	DL15-S120-3	gctgcattgtgtgagggtctct	ggcacccaacccatttaagag	2.4
DL15-S130	DL15-S130-1	cgaggcacctcatgtcaacac	caataagcaatcaccgcaccta	4.3
	DL15-S130-2	gcatatcctgttgcttgtgtaaaatg	taatcattaggctcaaacgaatgc	2.5
	DL15-S130-3	ctttgctatgctgcctgaattg	caccctggcttcaaccaaatag	3.8
DL15-S326	DL15-S326-1	ggccattgatctcgtatcagga	cgagcatatcttcttgcccaac	4.0
	DL15-S326-2	tctcctcaaggggactttactgc	tgggtgcgttctggaaatga	3.3
	DL15-S326-3	acgaccgacagacacataataaggta	gaggcgcttcttggtaaacaga	3.4
DL10-S1191	DL10-S1191-1	tagagactaatataagacgcatcggat	gttggtcaaattgccgaactt	3.3
	DL10-S1191-2	gccaccgcatctagcttatca	cccaaatgactcggaaatgttc	3.1
	DL10-S1191-3	tgggagaggatctggctgtaag	ttcggcccttgagtaatgagaa	3.7
DL10-S850	DL10-S850-1	tgtcgagtgatggcttggttg	tggttagccaacgtggtaatgg	3.7
	DL10-S850-2	accacaggcatctcagcagggt	cgaatcatgggccgactaaagga	3.3
	DL10-S850-3	tgcaacctgtgacgatttgaaa	gccctgggcaatagatcgaa	3.9
CHS125905	125905-1	ggaggagacaaggcaggagtt	acataggcggatttgggttg	4.1
	125905-2	cgatattaacaactactccaacggaac	gaattcaaagtcacctgccta	1.4
	125905-3	gaaatttgccatccctcgaa	ttttcgtcagcatttgttttatttg	4.4
DL10-S27	DL10-S27-1	ccgtgaagcaccttaaatggtt	gacggtttcattcccttgaaga	4.4
	DL10-S27-2	cacgatgaaatgccacaatga	taatatgcaccacaacctctttttat	2.3
	DL10-S27-3	cagcaaaaaagatcacaagattgg	gacccccagacatgagcattat	3.3
DL10-S31	DL10-S31-1	ttacgcgccaacaaatacaca	gttgcctctaactactgggcaaa	3.8
	DL10-S31-2	gtttgagtgtgcagtgagtttctctt	acacttacaactttgggccatctac	2.6
	DL10-S31-3	aatgtggctgagtaccattgctatt	gccacggctttcttgttgttt	4.5
DL19-S52	DL19-S52-1	ggattcgaacgcttccaaaa	accgtcaccttggatttgct	3.6
	DL19-S52-2	caaccttccctagatgctcca	tttcctcacatgccggtttt	3.5
	DL19-S52-3	agtttgccgtccacctcaac	cctcttgcacctccagctct	3.5
DL19-S36	DL19-S36-1	tgcgcaggctaatcttgaaa	accaaggcatttgccaagaa	3.6
	DL19-S36-2	tggagcccaaaaacatttcc	atacgaaccccgcaaaaaca	3.5
	DL19-S36-3	tctgcatacgagtgcatggat	tgaccgaagcaagatcagga	3.3
DL19-S345	DL19-S345-1	tatgtggtcgtagccggaag	ccaaactgctggtttgctca	3.8
	DL19-S345-2	agcctattggaagcagctttg	ttgtacacacctcaccaaaattga	3.5
	DL19-S345-3	tggtaccaaggatgcaaacg	ccaagatcctcccaaggtga	3.2
Unmapped-S68-456	S68-4	cgtttgccaatgccaactttc	tgaggagggtggtgattgctc	3.7
	S68-5	cttggacggagggagtcagc	tcaccccttcagctccacct	2.8
	S68-6	ggataaagaggaagatgctacgatcc	gaatcccctttgctttgaactttta	2.9
Unmapped-S68-123	S68-1	agagtgacagtggaagccttatgc	tggagtcgcacaacaacaaaaa	2.8
	S68-2	tacgcatttcccgtcgtcct	ggggctacaatagcgtcatttca	3.6
	S68-3	cacccaatcttctaagggactagg	tggggcatagaagtgggctta	2.7
DL11-S23	DL11-S23-1	ctccagttcttcaagggaacca	ccggtcaacttttgatctgttg	2.3
	DL11-S23-2	acgagcgattgacgaactgct	tctggaggtagggacgagcaa	3.2
	DL11-S23-3	ttctctgtctcccataatcaatacgaa	ccccacttggtgggataaagg	4.1
DL11-S165	DL11-S165-1	aaccaccgcacgagaaaacc	tgatttgcgtccaagggaca	3.5
	DL11-S165-2	atgtgaacctcaaaatcgcaaata	gcatgcaggtcatgcttcttg	1.5
	DL11-S165-3	catgaagaatgtgatgccctttt	ggaaatcatcacagcccaacc	4.2
DL11-S92	DL11-S92-1	attttgttttgtgccggttgg	atgtcggcccaaggaatttg	3.8
	DL11-S92-2	tgtggtgccgaccactgttt	tgtgcatgaatcaacatgcaaat	3.9
	DL11-S92-3	atggcctccctgtgtcaacc	caagggagcagcaccttcaa	4.1
CHS125513	125513-1	cttgcgttgtggagttcagtg	ccatcaggtgctcgctgttg	3.8
	125513-2	cattggggtcagtggtccat	tttacagctttggcgtggtg	2.1
	125513-3	gttcttcctgagcaacgtcctc	cgttggaccgatgagagatgatg	3.5
DL09-S401	DL09-S401-1	ccgtcgaactaaatccaaacat	tgagcccattggctaaattc	3.8
	DL09-S401-2	gcgcttttcatacggtgaat	tctcttgctctcaagggattg	3.2
	DL09-S401-3	agcgtgaccactcttgctct	gaggaccacgtgcgtataaaa	3.5
SSPACE-B2	B2-1	agctgtcaagggatcaacga	gattgcagctgttgttcgga	3.1
	B2-2	tgtactgagtttgagcccga	acagttcgcggaaaaccatc	2.7
	B2-3	tgttagacaagcggtgagga	cctcaatttgttgcaacggg	2.2
	B2-4	tcgtgtgatgcggaatagtt	tgagtaagtttgtcgcacca	3.2

SSPACE-B3	B3-1	gggcttaggtggtacgacaa	aggttcgattggtcgttttg	3.1
	B3-2	accgattgactcgaagtgct	gagcgctacgtttctctgct	3.8
	B3-3	cctcttttggtcctctgcaa	cgctgtgcgttcatttct	1.4
	B3-4	tcgcttcatcttcactcagg	gctgccctttcctttttctt	1.8
DL09-S1582	DL09-S1582-1	ttgacatttgcctttccaaga	tttggcatgaaaacatgctg	3.7
	DL09-S1582-2	ggtttgcaagtcgaaaagga	taggcaagttgcgaaagacc	3.6
	DL09-S1582-3	caaacccaatcacaggtgaac	ttttaggagcgcaattcaatg	3.2
DL09-S869	DL09-S869-1	catctgcagccacagtgagt	aaatcaagtggccaatagttgaat	3.4
	DL09-S869-2	tttgcttgaactggctcgat	gcattagaggggatgcttca	3.3
	DL09-S869-3	tcgtgtgaaggagcatggta	cgcaggagatgaagtcaggt	3.1
DL04-S363	DL04-S363-1	actgtgcgtctttcattcgagtc	gaagcaattatggaaaccgctct	4.2
	DL04-S363-2	catgccgaaatcctaagtgacat	ttggaaatccacttcctgatgc	3.8
	DL04-S363-3	gaagaagatgcagcctttttgtg	caatgtgaagcgcatctttcaa	4.0
DL04-S487	DL04-S487-1	tgtcaaccgaacgaattgaaca	tgttaaaggaactcgccaatcc	3.8
	DL04-S487-2	tgcaataactgttcggcttgg	gtttttctggcgatcttggaca	3.8
	DL04-S487-3	tggaaagcaaatgacatagtgaatg	ctgagcactgtcgggaagaaga	3.3
DL04-S246	DL04-NS-246-1	cgtcgaaacaaaatcagccatt	cccgccttgagtaagaacagc	3.1
	DL04-NS-246-2	tgggctatggctgtgtctgc	gcgagctttcgcaaattcct	3.6
	DL04-NS-246-3	tgcaccaaaaagcattcagatgt	caccattaagcctcccgttca	3.3
DL04-S337	DL04-S337-1	aaatcgttactgttggatgatgttacc	cggcgggttgaaacacaat	2.2
	DL04-S337-2	attccgacggacctgctgag	tgacattggagtaaaatgggcg	3.0
	DL04-S337-3	ttggtgaaaatggcagtgaagg	ttgccaccattggtcattgaa	2.8
	DL04-S337-4	cgctctctttctctctctctcaca	tcatcaatggacccatcacca	1.4
DL04-S445	DL04-S445-1	aagctccgttcatcgtcaattt	gtggcttctgacgatgctctct	3.3
	DL04-S445-2	tgttgacatggactgccaagtac	gtcgaatctagggcgcttacaa	4.4
	DL04-S445-3	aagtctagcccgttgcatctttt	gaaatgtgccgtatgaaatgca	3.9
DL06-S141	DL06-S141-1	accccctcttcttactttccaaaact	tgtcaaattcggtactaggcaaca	4.2
	DL06-S141-2	caaaatgtccccgtgaaatgc	tcattggaaaggccgattgag	3.4
	DL06-S141-3	cgaatcaaccccccttttaagc	ccttcgtacctttgggtgcat	4.0
DL06-S93	DL06-S93-1	tgaactggcataaactgtcacctt	gaggcgagtaaccaggtgtga	4.8
	DL06-S93-2	gaggggctaaagcgtctagtaact	gtccaacccagctcaatagctc	2.1
	DL06-S93-3	gtatttgttcgcacccatagct	aatgaaaacaaaatatgagggatgatt	3.5
DL06-S243	DL06-S243-1	taaaagcccgcagccttagag	cagactaccctttctaccatacatcacta	3.8
	DL06-S243-2	agcatccacgagttgatagcttt	aggaaattggcagagatccaaa	3.9
	DL06-S243-3	gaatccatgcacttggaatgtg	tgctacaggcatcgacagagc	3.3
DL17-S30	DL17-S30-1	ggttggaggttgacggttatt	atttgtgatggcccatgagt	3.5
	DL17-S30-2	acagttatcaaggtgggtcaga	gggcatagaaaggggagaaa	3.3
	DL17-S30-3	cagagtcatcggaggagcaa	ccattgccagacagcccc	3.3
DL17-S959	DL17-S959-1	aagtatgaagcacgagcaagaag	gatgagagagcaaagtccacc	2.9
	DL17-S959-2	gtgtgcttcagagtccttcaatc	cgttggatactcttgaccgc	3.9
	DL17-S959-3	gcgaaacaaaagagatgaagc	atcattcgtctcccagtcgt	3.2
DL17-S545	DL17-S545-1	ctggtggggtggatgaaa	cgttgatagaaggtcaaccaca	2.9
	DL17-S545-2	ttgtgaatgcggacttggt	acctcgccagtagatgcca	3.7
	DL17-S545-3	acggttgcttctgtgcttct	agtgatgaagaaccaataccctc	3.6
Unmapped-S17-123	S17-1	ttgtgctgggaagctacgtc	ttctcggtgtcccctgactt	3.1
	S17-2	caaaggcggctgcttaagat	tgctcattggaaggagtgga	3.4
	S17-3	gggtccttctcggcctagtt	aagcgagaaacaggctccaa	3.9
Unmapped-S17-456	S17-4	ggcccaatattgattaccagaca	ttgaagacgaatgggcactg	3.3
	S17-5	aggatggggaaaacatcgaa	agtgtttgcgtgaccactcg	3.2
	S17-6	gtttcgaggcgatcagtacg	tccaagctcagctccctctc	3.2
DL02-S169	DL02-S169-1	ttggatcaattttgggttcca	ccaacgagaccctcctctcc	3.4
	DL02-S169-2	ctgctgaggtcagatctctcg	gcgtgcattacactttgcac	1.5
	DL02-S169-3	tgcttgttaaacgtgcttcg	caagccacaaggccagttat	3.2
	DL02-S169-4	attttgtgcgatccatataggtg	tggccttgcaacagtgagta	1.8
DL02-S21	DL02-S21-1	tttaggtcagctgatttggtcct	actggcgtggttcatgttctt	2.7
	DL02-S21-2	gaggtgacaaaccgctaccttagag	ttgatgacagaggcgtcgag	1.4
	DL02-S21-3	tcctggaagtcattcatcgtg	cccgagcgatatgttcctct	2.2
	DL02-S21-4	tttcatccaaggggatctcg	ccaattgcaatgatgattatctctc	3.0
DL02-S46	DL02-S46-1	ggagacgctgacctagaacg	cagtacaccgtcgagcagaa	3.4
	DL02-S46-2	acaatgtcctacgcgtgcta	cgttctaggtcagcgtctcc	1.9

	DL02-S46-3	tccaagaccaaccactcgat	gaggtactggagagcgcttg	2.5
	DL02-S46-4	cctccgctgcttacaaagtc	gtggctggtgcgttaaaaat	2.8
Unmapped-S67-S12	S67-1	ttgttcgccggattatagggg	ctgacaatcgcctgacatcc	3.3
	S67-2	tgttggagatcttgactggactt	gcacgcggattacaatctca	4.0
	S67-3	ctcatgctcgaatccccttc	acgagatgggaagcgatgtt	3.3
Unmapped-S67-S45	S67-4	gtgggaggacagtcgaggag	ttgcttgcaaacgcaaaaac	3.0
	S67-5	tgcatcactcacatccatttca	cctccattaaccgtttgggata	3.3
	S67-6	atgattttggaagggggaaact	caacacgaccagaaactccttg	3.6
DL18-S186	DL18-S186-1	ccttcaagtgttttgggggaca	tttggtatgggacgtctttgg	2.7
	DL18-S186-2	tttccccatccgatagaagg	cgagacacctataaaattttctctca	3.3
	DL18-S186-3	ttgggttgatgagttctaggag	tgaggtcaactgtttcatattactttc	3.5
DL18-S103	DL18-S103-1	agggcattacccatcctgag	gctggaaggttgcacaaagac	3.6
	DL18-S103-2	gacacgcgccactgttttaat	tggattttgcaggactgtgc	3.0
	DL18-S103-3	gtgccagcttccagtagacc	tgcccacacaagaatagctt	3.5
DL18-S0	DL18-S0-1	attgcgttggatgattaggc	cgaacccccacattacttga	3.8
	DL18-S0-2	aaatttgaccggcatttgtc	taaggttttgcggcaagttt	3.0
	DL18-S0-3	cgtggacactcgagaacacc	ttggctctggtttggttcct	3.6
DL01-S49	DL01-S49-1	cccatcatctcccgaatttca	ataagctgggggtcttaggtgaa	3.9
	DL01-S49-2	ggctggtggaagcagctttt	ccgctcactgaccctctttga	3.4
	DL01-S49-3	cgacataactgttgccccctttt	gcattgctcaagtccccttca	2.4
Unmapped-S48-123	S48-1	ttgttctgcgtccgtttctca	ggaaggattgtggggagcaa	4.0
	S48-2	cccttttgagccaccaacc	ggaatgcccgcttgaaacat	2.3
	S48-3	gcatcccctatgggaaacaca	caagacatcgcgtccaccat	3.1
Unmapped-S48-456	S48-4	tggataagggaggaatctcaaaatga	ttgggttggtccacatcgttt	2.6
	S48-5	cccacaaatgaatccaagacga	caatcctacccactatgggttttt	3.2
	S48-6	tttaatcattagcccctttgtcctt	aaggcataactttaatcaagagtgacc	3.2
DL01-S22	DL01-S22-1	atttgcgtcatccccaaacc	cctcctgctgtgctcactgaat	2.7
	DL01-S22-2	ttcatgaagcttttgaggaaaaa	atagggccccacatcactcg	2.4
	DL01-S22-3	gagtgccaacgtcgtcctga	tgcagtcacaaaatcccatcaa	1.8
	DL01-S22-4	tccaccattgcctttaaggttt	gagcaagggagttgctccaa	2.8
DL01-S4	DL01-S4-1	aatcaacctgtttgccccatag	tggatcgtaacaccaattgagc	3.3
	DL01-S4-2	atcggcttggatcaattcgt	catagttctcacatgccatgaaaa	3.4
	DL01-S4-3	tgagtgacttgatttcggtagatgt	gcaaatgacgacatatcaacaca	3.0
DL01-S275	DL01-S275-1	tgcgttttcgaacttcgtgtat	acaggttgtgtttgaggccttt	3.7
	DL01-S275-2	ctcagcttaagcgatggaggat	gcgtacttgaggggctctctta	3.8
	DL01-S275-3	tgcaaacctatgagaacgatgg	tcttggataacatgcaggagga	3.6
DL01-S13	DL01-S13-1	tgtttgacctgcggacttactc	ctgcgcttctagttcctggttt	3.3
	DL01-S13-2	taatggtagcgaagacgaccaa	aaacacaggccctaagagcatc	3.1
	DL01-S13-3	cgggacggagtctgataatagg	aggaagggagcatatgaaggtt	3.1
DL01-S129	DL01-S129-1	ttgatttgactcggattggac	ttctccgggcaataattctg	1.9
	DL01-S129-2	aaaggtggcagtgaaattgg	catgaatcggggctttattt	3.5
	DL01-S129-3	gcattggatgatttcattattttc	agagacttgatcggctccat	2.7
	DL01-S129-4	tagccacactaagcccaagc	cttccaggttggttggttgt	3.0
DL01-S392	DL01-S392-1	ttgaacgacatccttgtcca	tcggcatgattacagcctta	3.6
	DL01-S392-2	tcgtttatgatattccctttaacc	ttcataggccccagtcacat	2.7
	DL01-S392-3	gagtggtcccccacaagtta	gacaaggaggggaggaaaag	2.1
	DL01-S392-4	tggcattgaacagtgttggt	agacagattcttccgcttcg	1.9
DL03-S306	DL03-S306-1	tggctgcatggggtctaata	tcatgcacaatcagtgatcaga	3.1
	DL03-S306-2	ttgctgcggttctatttgttc	cttgatgcaattatcaaagcaaaa	3.0
	DL03-S306-3	ccaaacaatcattcatcacatca	tttgtcacatcttcattttcacg	4.1
DL03-S543	DL03-S543-1	tgcatcagaatagactgcatcataa	atttgaattcgggggctgt	3.5
	DL03-S543-2	tcatggcactaaatgacagttcc	tttctgatccttcccattgc	3.0
	DL03-S543-3	gattcccccggaaaaagata	gagttcaagccggatcaaga	3.1
DL03-S202	DL03-S202-1	gggagagcctaccatccatt	ccatcattcacctgggagag	3.5
	DL03-S202-2	gcaaatcgtctcgacttcgt	ctcgcagtttgagccatctt	3.5
	DL03-S202-3	gggtgcaaatgaattatctcaaa	gagcttgggtgcatggttta	3.5
DL13-S776	DL13-S776-1	ttgtaggttacaggtaaatccctca	ccgtcggtttagcgcttatt	3.6
	DL13-S776-2	tgatgtgaaagagttgcatggt	agcccaatagaagggttcca	2.9
	DL13-S776-3	aggcggctatcaaccacttt	tgctccttagaacaaccacctt	3.1
DL13-S725	DL13-S725-1	agaaacaatggctccaatcg	ggagcctgttgttgttcgtaa	3.9

	DL13-S725-2	ttttgggggctaagaggagt	caatcgatggggatatggag	3.5
	DL13-S725-3	taacatgaatgtgtgaatccataa	gaaaatttagggcacctttgaa	2.5
DL13-S13	DL13-S13-1	gcaagtcagttgttatccacctc	caataaggcctaatatacccacca	3.3
	DL13-S13-2	taatgcgtggacactccaaa	gcgagcataacctcacgttt	3.1
	DL13-S13-3	attgagctacactgcgtgctt	ctcgtgtttccaaaaccagaa	3.1
DL13-S166	DL13-S166-1	gccaacatttgagaccgaaa	agtttgcaaatcacgcttacg	3.4
	DL13-S166-2	tccattatccctatgctgatgc	atgcaagtagttcacctttgttca	3.5
	DL13-S166-3	caaatgtaccctcgcaaagc	ccgaaatcatatgaaatgttgaga	3.2
DL16-S96	DL16-S96-1	caagcatggaggttctttgc	cccaaatggagcctaatagaatg	3.5
	DL16-S96-2	tctggaggagctaacatctgg	agcgaatgaacccatgaaac	3.3
	DL16-S96-3	agcaagcaccagctagaagc	agaggcggtcgagatagtca	3.7
CHS124207	124207-1	ccaggtttacgtgtttgatatgg	tgcaccgtactttcctgtgtt	3.5
	124207-2	tgtgttcgaggcaggtgtagt	cttctgcacacatcccattg	3.7
	124207-3	acaagtcgcgatacgaaagc	ccattggtcctcgctaggtat	2.9
DL16-S328	DL16-S328-1	caaccaacctgcctggatac	ccataaagtggaagtgggtga	2.9
	DL16-S328-2	tattccagtcaatttgtgggttg	attaactttaacatcaatgtccgttgc	3.0
	DL16-S328-3	cagtttgggcatctactttgg	aatgtgagccccaacttcat	4.0
DL16-S20	DL16-S20-1	atgaaaatttgccgcacaac	acatggctgcattctttcct	3.4
	DL16-S20-2	tgtttactttccaaatttcgttttt	ctcgacaagcaaaaccatga	3.3
	DL16-S20-3	gcctagtgaaatgggatgga	ttaaaaggcaaccctgaacg	3.3
5S rDNA	PCR product	gatcccatcagaacttc	ggtgctttagtgctggtat	0.7

Marker	Clone name	Description
45S rDNA	pTa71	DNA clone pTa71 contains a fragment of 45S rDNA of Triticum aestivum cloned into vector pAC184.
PABC059-1-H02	PABC059-1-H02	BAC clone PABC059-1-H02 was screened from a BAC library of Phalaenopsis
PABC065-3-D04	PABC065-3-D04	BAC clone PABC065-3-D04 was screened from a BAC library of Phalaenopsis
PABC094-4-F04	PABC094-4-F04	BAC clone PABC094-4-F04 was screened from a BAC library of Phalaenopsis
PEPC gene	PEPC-J14-E7K, PEPC-J14-H7K	Two DNA clones, PEPC-J14-E7K and PEPC-J14-H7K together contain a partial genome sequence of PEPC digested by EcoRI or HindIII from a BAC clone of
EFS gene	HBAC-1031-M24	BAC clone HBAC-1031-M24 was screened from a BAC library of Phalaenopsis

Table S6 Relative positions of FISH probes on pachytene chromosomes

Chromosome	Chr Length (um)	Marker	Relative distance (%)
1	40.52	DL14-S43	12.46
1	40.52	heterochromatin	18.57
1	40.52	centromere	30.63
1	40.52	heterochromatin end	63.10
1	40.52	DL14-S80	69.44
1	40.52	DL14-S8	98.57
1	40.52	DL14-S308	100.00
2	40.11	DL08-S176	9.12
2	40.11	heterochromatin	29.28
2	40.11	centromere	47.12
2	40.11	heterochromatin end	65.20
2	40.11	DL08-S535	66.32
2	40.11	DL08-S19	96.96
3	36.97	DL05-S874	1.73
3	36.97	DL05-S830	5.98
3	36.97	DL05-S9	15.09
3	36.97	heterochromatin	31.92
3	36.97	centromere	46.05
3	36.97	DL05-S347	49.78
3	36.97	heterochromatin end	57.50
3	36.97	DL05-S271	81.96
3	36.97	DL05-S240	91.24
4	36.86	DL12-S18	2.18
4	36.86	DL12-S319	24.13
4	36.86	heterochromatin	26.31
4	36.86	centromere	43.38
4	36.86	heterochromatin end	65.51
4	36.86	DL12-S2	89.20
5	33.7	heterochromatin	10.36
5	33.7	centromere	39.35
5	33.7	heterochromatin end	55.23
5	33.7	DL07-S1768	83.75
5	33.7	DL07-S84	86.69
5	33.7	DL07-S64	98.76
6	33.47	DL15-S120	9.00
6	33.47	heterochromatin	18.49
6	33.47	centromere	37.93
6	33.47	DL15-S130	70.11
6	33.47	heterochromatin end	72.03
6	33.47	DL15-S326	95.40
7	32.23	DL10-S1191	4.56
7	32.23	heterochromatin	18.65
7	32.23	centromere	30.95
7	32.23	heterochromatin end	53.67
7	32.23	DL10-S850	55.95

7	32.23	CHS125905	74.11
7	32.23	DL10-S27	84.62
7	32.23	DL10-S31	94.74
8	31.31	DL19-S52	2.04
8	31.31	DL19-S36	12.99
8	31.31	DL19-S345	20.55
8	31.31	heterochromatin	22.90
8	31.31	centromere	33.03
8	31.31	Unmapped-S68-456	60.33
8	31.31	heterochromatin end	60.53
8	31.31	Unmapped-S68-123	65.54
8	31.31	DL11-S23	76.99
8	31.31	DL11-S165	82.72
8	31.31	DL11-S92	91.21
9	31.22	CHS125513	0.00
9	31.22	DL09-S401	3.69
9	31.22	SSPACE-B2	6.26
9	31.22	SSPACE-B3	12.72
9	31.22	heterochromatin	20.62
9	31.22	centromere	36.21
9	31.22	DL09-S1582	53.03
9	31.22	heterochromatin end	53.64
9	31.22	DL09-S869	85.95
10	31.01	DL04-S363	2.07
10	31.01	DL04-S487	19.79
10	31.01	heterochromatin	21.04
10	31.01	centromere	33.47
10	31.01	heterochromatin end	59.79
10	31.01	DL04-S246	70.23
10	31.01	DL04-S337	90.67
10	31.01	DL04-S445	95.23
11	29.75	DL06-S141	4.95
11	29.75	heterochromatin	21.08
11	29.75	DL06-S93	27.31
11	29.75	centromere	36.34
11	29.75	heterochromatin end	52.47
11	29.75	DL06-S243	91.72
12	25.54	DL17-S30	3.79
12	25.54	PEPC gene	9.34
12	25.54	heterochromatin	15.03
12	25.54	DL17-S959	17.68
12	25.54	centromere	34.34
12	25.54	DL17-S545	59.97
12	25.54	heterochromatin end	73.36
12	25.54	Unmapped-S17-123	77.65
12	25.54	Unmapped-S17-456	88.89
13	24.3	DL02-S169	2.64
13	24.3	heterochromatin	28.80

13	24.3	centromere	49.01
13	24.3	DL02-S21	57.99
13	24.3	heterochromatin end	72.39
13	24.3	DL02-S46	95.64
13	24.3	EFS gene	99.34
14	23.78	Unmapped-S67-S123	2.30
14	23.78	Unmapped-S67-S456	8.54
14	23.78	heterochromatin	16.94
14	23.78	centromere	39.16
14	23.78	DL18-S186	53.66
14	23.78	DL18-S103	68.16
14	23.78	heterochromatin end	72.49
14	23.78	DL18-S0	94.99
15	23.38	DL01-S49	12.20
15	23.38	heterochromatin	18.16
15	23.38	centromere	37.67
15	23.38	Unmapped-S48-123	41.60
15	23.38	Unmapped-S48-456	44.17
15	23.38	heterochromatin end	58.81
15	23.38	DL01-S22	81.30
15	23.38	DL01-S4	95.80
16	20.75	heterochromatin	10.66
16	20.75	centromere	26.28
16	20.75	heterochromatin end	35.39
16	20.75	DL01-S275	32.92
16	20.75	DL01-S13	53.79
16	20.75	DL01-S129	80.83
16	20.75	PABC065-3-D04	90.42
16	20.75	PABC059-1-H02	94.28
16	20.75	PABC094-4-F04	96.91
16	20.75	DL01-S392	98.92
17	18.14	DL03-S306	2.30
17	18.14	heterochromatin	13.96
17	18.14	centromere	46.29
17	18.14	DL03-S543	74.56
17	18.14	heterochromatin end	76.33
17	18.14	DL03-S202	95.58
18	17.72	DL13-S776	2.36
18	17.72	DL13-S725	9.26
18	17.72	heterochromatin	12.89
18	17.72	centromere	31.40
18	17.72	heterochromatin end	48.46
18	17.72	DL13-S13	65.52
18	17.72	DL13-S166	97.28
19	16.97	DL16-S96	4.33
19	16.97	CHS124207	13.94
19	16.97	heterochromatin	23.35
19	16.97	centromere	45.95

19	16.97	DL16-S328	54.05
19	16.97	heterochromatin end	75.52
19	16.97	DL16-S20	99.06

Table S7 Coordinates of recombination hot spots, list of genes within the hot spots and gene density

Chromosome	Linkage group	Hot spot start	Hot spot end	Gene density in the 100kb interval containing hot spot (No. genes per 100kb)	No. genes in hotspots	List of genes within the hot spots
1	L01	1949474	1984763	8	3	PAXXG290120,PAXXG290130,PAXXG290140
1	L01	2596454	2647009	5	2	PAXXG064210,PAXXG064220
1	L01	4774746	4779878	5	2	PAXXG065140,PAXXG065150
1	L01	4964049	4985545	4	1	PAXXG065230
1	L01	23528215	23540699	4	3	PAXXG098150,PAXXG098170,PAXXG098190
1	L01	24627311	24639888	5	1	PAXXG098610
1	L01	37403999	37414996	6	3	PAXXG238990,PAXXG239000,PAXXG239010
2	L02	3884628	3899948	5	3	PAXXG072490,PAXXG072500,PAXXG072510
2	L02	7159044	7178112	4	2	PAXXG076870,PAXXG076880
2	L02	15232359	15233923	5	0	
2	L02	25705153	25705456	2	1	PAXXG282700
2	L02	33415649	33436427	6	2	PAXXG003660,PAXXG003670
2	L02	35681123	35693037	7	2	PAXXG002710,PAXXG002720
3	L03	3236581	3263973	8	4	PAXXG069060,PAXXG069070,PAXXG069080,PAXXG069090
3	L03	8164516	8172601	4	1	PAXXG224410
3	L03	26549156	26551226	4	1	PAXXG086650
3	L03	32501016	32527971	5	2	PAXXG082330,PAXXG082340
3	L03	35228553	35237969	2	1	PAXXG093420
4	L04	3426346	3452716	7	2	PAXXG065460,PAXXG065470
4	L04	17519437	17529126	5	2	PAXXG175080,PAXXG175100
4	L04	20540906	20622714	6	4	PAXXG137890,PAXXG137900,PAXXG137910,PAXXG137920
4	L04	21053760	21073258	3	0	
5	L05a	467814	490380	8	2	PAXXG166180,PAXXG166190
6	L06	2910223	2985091	7	5	PAXXG297690,PAXXG297700,PAXXG297710,PAXXG297720,PAXXG297730
6	L06	12294214	12299565	3	1	PAXXG241440
6	L06	13216087	13228902	3	1	PAXXG327780
6	L06	23273869	23280465	5	1	PAXXG088770
6	L06	24481280	24504267	8	2	PAXXG079240,PAXXG079250
7	L07	17756283	17765497	4	1	PAXXG329260
8	L08b	4274065	4300486	8	1	PAXXG010560
8	L08b	9529997	9559460	6	2	PAXXG195170,PAXXG195180
8	L08b	9559461	9651533	6	6	PAXXG195180,PAXXG195190,PAXXG195200,PAXXG195210,PAXXG195220,PAXXG195230
8	L08b	9651534	9655177	5	1	PAXXG195230
9	L09	2769073	2793418	5	2	PAXXG115640,PAXXG115650
9	L09	23562885	23568507	1	0	
9	L09	26774725	26783723	4	1	PAXXG100030
9	L09	28468014	28472637	12	2	PAXXG100905,PAXXG100910
10	L10a	3924397	3952995	6	4	PAXXG321020,PAXXG321030,PAXXG321035,PAXXG321040
10	L10a	23976375	23984680	2	1	PAXXG003920
10	L10a	29260355	29295095	4	2	PAXXG006210,PAXXG006220
10	L10a	33933495	34013740	7	6	PAXXG008470,PAXXG008480,PAXXG008490,PAXXG008500,PAXXG008510,PAXXG008520
10	L10b	1166421	1167897	9	1	PAXXG246420
10	L10b	15453117	15459529	3	1	PAXXG188200
10	L10b	15493467	15514472	2	2	PAXXG188200,PAXXG188210
10	L10b	19071903	19092981	4	2	PAXXG031580,PAXXG031590
11	L11	373355	381697	7	1	PAXXG144870
11	L11	1529667	1570286	4	2	PAXXG145630,PAXXG145640
11	L11	1881052	1893850	8	2	PAXXG130770,PAXXG130780
11	L11	5108133	5135227	5	3	PAXXG024050,PAXXG024060,PAXXG024070
11	L11	9347798	9376288	2	2	PAXXG176330,PAXXG176340
11	L11	13810481	13826980	4	2	PAXXG212580,PAXXG212590
11	L11	26060990	26079325	4	1	PAXXG275120
11	L11	34530664	34543855	6	0	
11	L11	35325776	35337551	6	1	PAXXG075720
12	L12	22177653	22201047	1	1	PAXXG068330
13	L13	3101	21633	5	1	PAXXG219520
13	L13	4889990	4897043	8	1	PAXXG026850
13	L13	24254730	24279517	7	2	PAXXG058150,PAXXG058170
13	L13	27754489	27763625	9	2	PAXXG193500,PAXXG193510
15	L15	2552424	2579162	6	3	PAXXG038310,PAXXG038320,PAXXG038330
15	L15	6672653	6677096	4	1	PAXXG092340
16	L16	4426819	4427436	3	1	PAXXG235440
16	L16	13058135	13060970	5	1	PAXXG035200
16	L16	19685402	19697805	7	2	PAXXG102250,PAXXG102260
16	L16	20279649	20291566	13	3	PAXXG101820,PAXXG101830,PAXXG101840
17	L17	648068	668961	9	1	PAXXG139920
17	L17	1067731	1110768	9	3	PAXXG139510,PAXXG139520,PAXXG139530
18	L18	109113	119252	9	1	PAXXG313180
18	L18	11081818	11088487	4	3	PAXXG084120,PAXXG084130,PAXXG084140
18	L18	15338357	15341550	2	1	PAXXG053520
18	L18	18198271	18224446	7	4	PAXXG052220,PAXXG052240,PAXXG052250,PAXXG052260
18	L18	26068180	26089193	8	2	PAXXG199460,PAXXG199470
19	L19	1588821	1644378	5	2	PAXXG305360,PAXXG305370
19	L19	7003478	7006663	0	0	

Table S8 Annotation of genes within the recombination hot spots

Gene ID	Description
PAXXG290120	BRCA1-associated RING domain protein
PAXXG290130	Cyanate hydratase
PAXXG290140	hypothetical protein
PAXXG064210	GARP-G2-like transcription factor
PAXXG064220	C2H2 transcription factor
PAXXG065140	Unknown protein, supported by RNASeq data
PAXXG065150	Diphthamide biosynthesis protein
PAXXG065230	Trihelix transcription factor
PAXXG098150	Phospholipid:diacylglycerol acyltransferase
PAXXG098170	Similar to uncharacterized protein LOC102625288 isoform X1
PAXXG098190	linoleate 13S-lipoxygenase
PAXXG098610	Unknown protein, supported by RNASeq data
PAXXG238990	Granule-bound starch synthase-like
PAXXG239000	apyrase
PAXXG239010	DMP1 protein
PAXXG072490	Hydroxyphenylpyruvate reductase
PAXXG072500	3-methyl-2-oxobutanoate hydroxymethyltransferase
PAXXG072510	HEAT repeat-containing protein
PAXXG076870	Ribosome biogenesis protein
PAXXG076880	beta-carotene hydroxylase
PAXXG282700	CBS domain-containing protein
PAXXG003660	Galactan beta-1,4-galactosyltransferase
PAXXG003670	Similar to uncharacterized protein LOC104220161
PAXXG002710	Similar to predicted protein
PAXXG002720	Phytolongin Phyl1.1
PAXXG069060	Hydrophobic protein
PAXXG069070	Unknown protein, supported by RNASeq data
PAXXG069080	Pentatricopeptide repeat-containing protein
PAXXG069090	AUGMIN subunit 8
PAXXG224410	Protein FLX-like
PAXXG086650	MYB transcription factor
PAXXG082330	Transporter, the Mitochondrial Carrier (MC) Family
PAXXG082340	26S proteasome non-ATPase regulatory subunit 3
PAXXG093420	Kinesin-like protein
PAXXG065460	Pentatricopeptide repeat-containing protein
PAXXG065470	Unknown protein, supported by RNASeq data
PAXXG175080	Transporter, the ATP-binding Cassette (ABC) Superfamily
PAXXG175100	uncharacterized protein
PAXXG137890	cinnamoyl-CoA reductase
PAXXG137900	Eukaryotic translation initiation factor 5A-4
PAXXG137910	Chorismate mutase
PAXXG137920	unknown protein, supported by RNASeq data
PAXXG166180	N-acetyltransferase 9-like protein
PAXXG166190	U-box domain-containing protein
PAXXG297690	Histone-lysine N-methyltransferase
PAXXG297700	Protein trichome birefringence-like
PAXXG297710	unknown protein, supported by RNASeq data
PAXXG297720	GDSL esterase/lipase
PAXXG297730	TCP transcription factor family, CIN subfamily
PAXXG241440	Pollen-specific leucine-rich repeat extensin-like protein
PAXXG327780	bifunctional methylthioribulose-1-phosphate dehydratase/enolase-phosphatase E1

PAXXG088770	Cycloartenol-C-24-methyltransferase
PAXXG079240	Unknown protein, supported by RNASeq data
PAXXG079250	Strigolactone esterase
PAXXG329260	Alpha-ketoglutarate-dependent dioxygenase
PAXXG010560	HB-HD-ZIP transcription factor
PAXXG195170	Transporter, the Major Intrinsic Protein (MIP) Family
PAXXG195180	Transporter, the Major Intrinsic Protein (MIP) Family
PAXXG195190	Pentatricopeptide repeat-containing protein
PAXXG195200	oxidation resistance protein
PAXXG195210	Putative pumilio homolog
PAXXG195220	unknown protein, supported by RNASeq data
PAXXG195230	Endoplasmic reticulum-Golgi intermediate compartment protein
PAXXG115640	unknown protein, supported by RNASeq data
PAXXG115650	Beta-glucosidase 18-like
PAXXG100030	Transporter, the Drug/Metabolite Transporter (DMT) Superfamily
PAXXG100905	Unknown protein, supported by RNASeq data
PAXXG100910	Pentatricopeptide repeat-containing protein
PAXXG321020	CRS2-associated factor
PAXXG321030	Transporter, the Major Facilitator Superfamily (MFS)
PAXXG321035	unknown protein, supported by RNASeq data
PAXXG321040	Carotenoid 9,10(9',10')-cleavage dioxygenase
PAXXG003920	Unknown protein, supported by RNASeq data
PAXXG006210	TWIN LOV Protein
PAXXG006220	28S rRNA (cytosine-C(5))-methyltransferase
PAXXG008470	Unknown protein, supported by RNASeq data
PAXXG008480	Unknown protein, supported by RNASeq data
PAXXG008490	Pentatricopeptide repeat-containing protein
PAXXG008500	Photosystem I subunit O
PAXXG008510	F-box protein
PAXXG008520	Phosphatase 1 regulatory subunit
PAXXG246420	Powdery mildew resistance protein 5-like
PAXXG188200	Neuroguidin-like
PAXXG188210	NEP1-interacting protein 1-like
PAXXG031580	CBL-interacting protein kinase
PAXXG031590	Unknown protein, supported by RNASeq data
PAXXG144870	CCT domain protein
PAXXG145630	C3H transcription factor
PAXXG145640	MYB transcription factor
PAXXG130770	unknown protein, supported by RNASeq data
PAXXG130780	C2H2 transcription factor
PAXXG024050	Lysine-specific demethylase
PAXXG024060	Stearoyl-[acyl-carrier-protein] 9-desaturase
PAXXG024070	Lysine-specific demethylase
PAXXG176330	uncharacterized protein
PAXXG176340	GARP-G2-like transcription factor
PAXXG212580	trans-2-hexenal reductase,oxidoreductase
PAXXG212590	trans-2-hexenal reductase,oxidoreductase
PAXXG275120	F-box protein
PAXXG075720	Plant cysteine oxidase
PAXXG068330	U1 small nuclear ribonucleoprotein C
PAXXG219520	phospholipase A I-like
PAXXG026850	Cinnamoyl-CoA reductase
PAXXG058150	Subtilisin-like protease

PAXXG058170	bHLH transcription factor
PAXXG193500	Glycolipid transfer protein
PAXXG193510	Serine/arginine-rich splicing factor
PAXXG038310	Regulator of telomere elongation helicase
PAXXG038320	OFP transcription factor
PAXXG038330	Serine/threonine-protein kinase
PAXXG092340	Transcription factor bHLH
PAXXG235440	Transcription-associated protein 1
PAXXG035200	Caffeoylshikimate esterase
PAXXG102250	F-box protein
PAXXG102260	UDP-sulfoquinovose synthase
PAXXG101820	M-alpha type, PaMalpha2
PAXXG101830	Unknown protein, supported by RNASeq data
PAXXG101840	UPSTREAM OF FLC
PAXXG139920	WPP domain-interacting tail-anchored protein
PAXXG139510	CBL-interacting protein kinase
PAXXG139520	Leucine-rich repeat protein
PAXXG139530	SNF1-related protein kinase regulatory subunit beta-1
PAXXG313180	HB-HD-ZIP transcription factor
PAXXG084120	Similar to hypothetical protein POPTR_0016s10800g
PAXXG084130	Unknown protein, supported by RNASeq data
PAXXG084140	GRAS transcription factor
PAXXG053520	Transporter, the ATP-binding Cassette (ABC) Superfamily
PAXXG052220	Transporter, the Major Facilitator Superfamily (MFS)
PAXXG052240	Unknown protein, supported by RNASeq data
PAXXG052250	OFP transcription factor
PAXXG052260	Unknown protein, supported by RNASeq data
PAXXG199460	B-BOX domain protein
PAXXG199470	Universal stress protein
PAXXG305360	homogentisate phytyltransferase
PAXXG305370	Serine/threonine-protein kinase

Table S9 List of recombination cold spots and gene density

Chromosome	Linkage group	Cold spot start	Cold spot end	No. genes in cold spot	Gene density (No. genes per 100kb)
7	L07	7925296	16071964	155	1.90
2	L02	17451706	22951198	78	1.42
1	L01	13404324	18900535	91	1.66
14	L14	268409	5685122	107	1.98
13	L13	11937160	17071711	92	1.79
11	L11	14173526	19306527	101	1.97
9	L09	17003521	21281543	66	1.54
6	L06	7305145	11284910	90	2.26
9	L09	3906838	7589586	135	3.67
10	L10a	12235612	15801812	56	1.57
12	L12	11762533	15106456	57	1.70
12	L12	8014645	11006066	49	1.64
15	L15	10232403	13195728	103	3.48
18	L18	11088488	13781140	56	2.08
12	L12	16801094	19462902	42	1.58
2	L02	12595353	15232453	52	1.97
18	L18	6871150	9295678	55	2.27
19	L19	7006569	9312937	44	1.91
10	L10b	11281837	13491100	47	2.13
10	L10a	9160567	11360027	43	1.96
3	L03	16133241	18154789	35	1.73
2	L02	27969064	29948067	42	2.12
10	L10a	17949901	19917738	38	1.93
9	L09	14378046	15950517	31	1.97
16	L16	130295	1702364	39	2.48
16	L16	2422375	3990997	36	2.30
10	L10a	7269810	8816154	45	2.91
17	L17	11049261	12549030	25	1.67
6	L06	13907976	15378833	33	2.24
6	L06	16947208	18382461	28	1.95
17	L17	7762538	9191927	27	1.89
3	L03	26551227	27918979	56	4.09
19	L19	9844529	11174194	35	2.63
10	L10a	16194077	17395927	28	2.33
9	L09	21987186	23187808	29	2.42
16	L16	7092819	8236452	37	3.24
19	L19	4717985	5847233	21	1.86
1	L01	10090555	11119372	20	1.94
15	L15	8147145	9165339	24	2.36

Table S10 List of genes within the cold hot spots and gene annotation

Chromosome	Linkage group	cold spot region	Gene ID	Description
7	L07	7925296~16071964	PAXXG346250	uncharacterized protein, homologue of XP_020572384.1
			PAXXG346260	Sphingosine-1-phosphate lyase
			PAXXG189990	RNA demethylase ALKBH5-like
			PAXXG190000	Urease
			PAXXG190010	ataxia telangiectasia mutated family protein
			PAXXG190020	RNA demethylase ALKBH5-like
			PAXXG190030	expansin-B16-like
			PAXXG190040	uncharacterized protein, homologue of XP_020581221.1
			PAXXG190060	Embryonic stem cell-specific 5-hydroxymethylcytosine-binding protein
			PAXXG190070	conserved protein of unknown function, supported by RNASeq data
			PAXXG190080	1,4-dihydroxy-2-naphthoyl-CoA synthase, peroxisomal
			PAXXG190090	Pentatricopeptide repeat-containing protein
			PAXXG190100	Eukaryotic translation initiation factor 4B1
			PAXXG190110	uncharacterized protein, homologue of XP_020696325.1
			PAXXG190120	uncharacterized protein, homologue of XP_020595531.1
			PAXXG190130	Variant surface antigen E
			PAXXG190140	RNA demethylase ALKBH5-like
			PAXXG190150	ribulose-1,5 bisphosphate carboxylase/oxygenase large subunit N-methyltransferase, chloroplastic
			PAXXG190160	Coilin-like
			PAXXG190170	transcriptional regulator SLK3-like
			PAXXG190180	Coilin-like
			PAXXG190190	FAR1 transcription factor
			PAXXG190200	protein FAR1-RELATED SEQUENCE 1-like
			PAXXG195430	RNA demethylase ALKBH5-like
			PAXXG195470	dihydroflavonol 4-reductase
			PAXXG195490	conserved hypothetical protein homologue of PKU67230.1
			PAXXG195500	expansin-B16-like
			PAXXG195570	GTPase ERA
			PAXXG195620	uncharacterized protein, homologue of XP_020597546.1
			PAXXG195660	RNA demethylase ALKBH5
			PAXXG195670	RNA demethylase ALKBH5
			PAXXG195680	Similar to RNA demethylase
			PAXXG195720	Unknown protein, supported by RNA-Seq data
			PAXXG195750	RNA demethylase ALKBH5-like
			PAXXG195760	uncharacterized protein, homologue of XP_020598317.1
			PAXXG195790	uncharacterized protein, homologue of XP_020598317.1
			PAXXG195800	conserved hypothetical protein homologue of PKU67230.1
			PAXXG195810	nucleotidyltransferase, Ribonuclease H
			PAXXG195820	conserved protein of unknown function, supported by RNASeq data
			PAXXG195830	Mannan endo-1,4-beta-mannosidase
			PAXXG195860	Similar to RNA demethylase ALKBH5
			PAXXG195870	Similar to Oxidoreductase
			PAXXG195880	Pentatricopeptide repeat-containing protein
			PAXXG195920	Similar to RNA demethylase ALKBH5
			PAXXG195960	uncharacterized protein, homologue of XP_020595548.1
			PAXXG195990	uncharacterized protein, homologue of XP_020678337.1
			PAXXG196000	Similar to RNA demethylase ALKBH5-like
			PAXXG196030	conserved protein of unknown function, supported by RNASeq data
			PAXXG196040	uncharacterized protein, homologue of XP_020585569.1
			PAXXG196130	uncharacterized protein, homologue of XP_020570899.1
			PAXXG215390	WRKY transcription factor
			PAXXG215420	expansin-B16-like
			PAXXG215440	RNA demethylase ALKBH5-like
			PAXXG215450	L10-interacting MYB domain-containing protein
			PAXXG215465	uncharacterized protein, homologue of XP_020582885.1
			PAXXG215470	Aspartic proteinase oryzasin
			PAXXG215480	uncharacterized protein, homologue of XP_020599770.1
			PAXXG215500	uncharacterized protein, homologue of XP_020584790.1
			PAXXG215550	Carboxyl-terminal-processing peptidase
			PAXXG215555	conserved hypothetical protein homologue of PKU68566.1
			PAXXG215560	Transporter, the CorA Metal Ion Transporter (MIT) Family
			PAXXG215570	uncharacterized protein, homologue of XP_020599657.1
			PAXXG215590	uncharacterized protein, homologue of XP_020596678.1
			PAXXG215600	Chlorophyll a-b binding protein
			PAXXG284800	TLC domain-containing protein
			PAXXG284810	Stromal 70 kDa heat shock-related protein
			PAXXG284820	uncharacterized protein, homologue of XP_020598333.1
			PAXXG284830	uncharacterized protein, homologue of XP_020703149.1
			PAXXG284840	Unknown protein, supported by RNA-Seq data, weak similar to XP_020599657.1
			PAXXG284850	conserved hypothetical protein homologue of PKU67230.1
			PAXXG284870	Trihelix transcription factor
			PAXXG284880	Similar to RNA demethylase ALKBH5
			PAXXG284900	uncharacterized protein, homologue of XP_020599657.1
			PAXXG284910	RNA demethylase ALKBH5-like
			PAXXG284920	uncharacterized protein, homologue of XP_020599657.1
			PAXXG284930	F-box protein
			PAXXG303300	Pre-mRNA-splicing factor ATP-dependent RNA helicase DEAH4
			PAXXG303320	Scarecrow-like protein
			PAXXG303340	uncharacterized protein, homologue of XP_020599700.1
			PAXXG303350	Flavin mononucleotide hydrolase
			PAXXG303370	Sister chromatid cohesion protein SCC4
			PAXXG303380	ataxia telangiectasia mutated family protein
			PAXXG303390	Similar to retrovirus-related Pol polyprotein from transposon TNT 1-94
			PAXXG303410	Zinc finger BED domain-containing protein
			PAXXG303430	Unknown protein, supported by RNA-Seq data, weak similar to XP_020599657.1
			PAXXG303620	Similar to RNA demethylase ALKBH5
			PAXXG303630	Plant UBX domain-containing protein
			PAXXG303650	non-specific lipid-transfer protein-like 1
			PAXXG303670	conserved hypothetical protein homologue of PKU67230.1
			PAXXG303680	Caltractin

			PAXXG303690	uncharacterized protein, homologue of XP_020588287.1
			PAXXG104680	RNA demethylase ALKBH5-like
			PAXXG104720	conserved protein of unknown function, supported by RNASeq data
			PAXXG104740	RNA demethylase ALKBH5-like
			PAXXG104770	uncharacterized protein, homologue of XP_020595656.1
			PAXXG104870	RNA demethylase ALKBH5-like
			PAXXG104880	uncharacterized protein, homologue of XP_020598667.1
			PAXXG104940	RNA demethylase ALKBH5-like
			PAXXG104950	uncharacterized protein, homologue of XP_020596333.1
			PAXXG104970	RNA demethylase ALKBH5-like
			PAXXG105030	uncharacterized protein, homologue of XP_020595829.1
			PAXXG105050	uncharacterized protein, homologue of XP_020598565.1
			PAXXG105060	uncharacterized protein, homologue of XP_020582885.1
			PAXXG105080	uncharacterized protein, homologue of XP_020581952.1
			PAXXG105140	uncharacterized protein, homologue of XP_020598467.1
			PAXXG105150	uncharacterized protein, homologue of XP_020598165.1
			PAXXG105160	endonuclease/exonuclease/phosphatase family protein
			PAXXG105170	uncharacterized protein, homologue of XP_020599700.1
			PAXXG105220	uncharacterized protein, homologue of XP_020598403.1
			PAXXG105240	uncharacterized protein, homologue of XP_020599770.1
			PAXXG105250	uncharacterized protein, homologue of XP_020598113.1
			PAXXG105270	RNA demethylase ALKBH5-like
			PAXXG105280	RNA demethylase ALKBH5-like
			PAXXG105310	GDSL esterase/lipase
			PAXXG105330	conserved hypothetical protein homologue of PKU67230.1
			PAXXG105380	uncharacterized protein, homologue of XP_020597249.1
			PAXXG105390	uncharacterized protein, homologue of XP_020599258.1
			PAXXG105400	RNA demethylase ALKBH5-like
			PAXXG105480	RNA demethylase ALKBH5-like
			PAXXG105510	RNA-directed DNA polymerase like
			PAXXG105530	RNA demethylase ALKBH5-like
			PAXXG105540	Probable serine/threonine-protein kinase WNK2
			PAXXG105550	Cleavage and polyadenylation specificity factor subunit 2
			PAXXG105560	60S ribosomal protein L26
			PAXXG105570	unknown protein, supported by RNASeq data
			PAXXG105580	Mediator of RNA polymerase II transcription subunit
			PAXXG105610	RNA demethylase ALKBH5-like
			PAXXG105630	Hypothetical protein, supported by RNA-Seq data
			PAXXG105660	Peptidyl-tRNA hydrolase, mitochondrial
			PAXXG105665	BTB/POZ domain and ankyrin repeat-containing protein NPR5-like
			PAXXG105670	Hypothetical protein, supported by RNA-Seq data
			PAXXG105680	Fanconi-associated nuclease 1 homolog
			PAXXG105700	uncharacterized protein, homologue of XP_020599770.1
			PAXXG105720	uncharacterized protein, homologue of XP_020588171.1
			PAXXG306170	RNA demethylase ALKBH5-like
			PAXXG306200	conserved protein of unknown function, supported by RNASeq data
			PAXXG306220	uncharacterized protein, homologue of XP_020580828.1
			PAXXG306240	Similar to Oxidoreductase
			PAXXG306310	Similar to RNA demethylase ALKBH5
			PAXXG306320	conserved hypothetical protein homologue of PKU67230.1
			PAXXG306390	RNA demethylase ALKBH5-like
			PAXXG332730	Phosphoglucan phosphatase
			PAXXG332735	uncharacterized protein, homologue of XP_020588853.1
			PAXXG332740	uncharacterized protein, homologue of XP_020596620.1
			PAXXG135260	RNA demethylase ALKBH5-like
			PAXXG135270	receptor protein kinase-like protein ZAR1
			PAXXG135280	uncharacterized protein, homologue of XP_020597778.1
			PAXXG135310	Lipid phosphate phosphatase
			PAXXG135320	RNA demethylase ALKBH5-like
			PAXXG135350	Isocitrate dehydrogenase regulatory subunit 1, mitochondrial
			PAXXG135360	conserved hypothetical protein homologue of PKU67230.1
			PAXXG135370	Chloride conductance regulatory protein
			PAXXG135380	protein FAR1-RELATED SEQUENCE 5-like
			PAXXG135390	expansin-B16-like
			PAXXG135400	uncharacterized protein, homologue of XP_020591584.1
2	L02	17451706~22951198	PAXXG172700	centromere/kinetochore protein zw10 homolog
			PAXXG172720	expansin-B16-like
			PAXXG172730	uncharacterized protein, homologue of XP_020582885.1
			PAXXG172770	RNA demethylase ALKBH5-like
			PAXXG172810	uncharacterized protein, homologue of XP_020593563.1
			PAXXG172820	RNA demethylase ALKBH5-like
			PAXXG172830	Unknown protein, supported by RNA-Seq data
			PAXXG172840	chromatin remodeling protein EBS-like
			PAXXG172850	E3 ubiquitin-protein ligase BRE1-like
			PAXXG172900	Transporter, the Drug/Metabolite Transporter (DMT) Superfamily
			PAXXG172910	protein argonaute 16-like isoform X1
			PAXXG172920	C3H transcription factor
			PAXXG209750	uncharacterized protein, homologue of XP_020598317.1
			PAXXG209760	uncharacterized protein, homologue of XP_020598766.1
			PAXXG209800	RNA demethylase ALKBH5-like
			PAXXG209820	uncharacterized protein, homologue of XP_020573235.1
			PAXXG209840	uncharacterized protein, homologue of XP_020593563.1
			PAXXG209850	uncharacterized protein, homologue of XP_020598317.1
			PAXXG209870	RNA demethylase ALKBH5-like
			PAXXG209880	probable isoprenylcysteine alpha-carbonyl methylesterase ICMEL1 isoform X1
			PAXXG209900	uncharacterized protein, homologue of XP_020595831.1
			PAXXG209910	uncharacterized protein, homologue of XP_020596122.1
			PAXXG209920	arabinosyltransferase XEG113 isoform X2
			PAXXG209930	Transporter, the P-type ATPase (P-ATPase) Superfamily
			PAXXG209940	conserved protein of unknown function, supported by RNASeq data
			PAXXG209950	laccase-14-like
			PAXXG209960	G patch domain-containing protein TGH

			PAXXG209970	RNA-directed DNA polymerase
			PAXXG209990	conserved protein of unknown function, supported by RNASeq data
			PAXXG245750	uncharacterized protein, homologue of XP_020592939.1
			PAXXG245760	RNA demethylase ALKBH5-like
			PAXXG245810	Unknown protein, supported by RNA-Seq data, weak similar to XP_020599657.1
			PAXXG245830	conserved hypothetical protein homologue of PKU67230.1
			PAXXG245840	mediator of RNA polymerase II transcription subunit 11
			PAXXG245870	subtilisin-like protease SBT1.7
			PAXXG245880	RNA demethylase ALKBH5-like
			PAXXG245940	uncharacterized protein, homologue of XP_020596157.1
			PAXXG245950	pumilio homolog 5
			PAXXG245960	conserved protein of unknown function, supported by RNASeq data
			PAXXG095410	RNA demethylase ALKBH5-like
			PAXXG095420	uncharacterized protein, homologue of XP_020595915.1
			PAXXG095430	uncharacterized protein, homologue of XP_020595656.1
			PAXXG095440	uncharacterized protein, homologue of XP_020595835.1
			PAXXG095470	RNA demethylase ALKBH5-like
			PAXXG095480	uncharacterized protein, homologue of XP_020593563.1
			PAXXG095510	RNA demethylase ALKBH5-like
			PAXXG095520	uncharacterized protein, homologue of XP_020592067.1
			PAXXG095530	uncharacterized protein, homologue of XP_020599182.1
			PAXXG095540	RNA demethylase ALKBH5-like
			PAXXG095560	probable alpha-mannosidase At5g13980
			PAXXG095570	leucine--tRNA ligase, chloroplastic/mitochondrial isoform X1
			PAXXG095580	Unknown protein, supported by RNA-Seq data, weak similar to PKU67230.1
			PAXXG095630	uncharacterized protein, homologue of XP_020596058.1
			PAXXG095650	uncharacterized protein, homologue of XP_020598165.1
			PAXXG095660	Transporter, the Multidrug/Oligosaccharidyl-lipid/Polysaccharide (MOP) Flippase Superfamily
			PAXXG095670	uncharacterized protein, homologue of XP_020598317.1
			PAXXG095750	FAR1 transcription factor
			PAXXG095790	RNA demethylase ALKBH5-like
			PAXXG095820	uncharacterized protein, homologue of XP_020595915.1
			PAXXG095860	uncharacterized protein, homologue of XP_020598859.1
			PAXXG095890	2-hydroxyacyl-CoA lyase
			PAXXG095900	serine/threonine-protein kinase haspin homolog isoform X2
			PAXXG095910	uncharacterized protein, homologue of XP_020599223.1
			PAXXG095920	uncharacterized protein, homologue of XP_020597516.1
			PAXXG095930	uncharacterized protein, homologue of XP_020599500.1
			PAXXG095940	uncharacterized protein, homologue of XP_020598420.1
			PAXXG095960	uncharacterized protein, homologue of XP_020595835.1
			PAXXG096000	uncharacterized protein, homologue of XP_020596058.1
			PAXXG096010	uncharacterized protein, homologue of XP_020680661.1
			PAXXG096050	uncharacterized protein, homologue of XP_020596058.1
			PAXXG096060	uncharacterized protein, homologue of XP_020598317.1
			PAXXG096070	uncharacterized protein, homologue of XP_020598317.1
			PAXXG096170	uncharacterized protein, homologue of XP_020598317.1
			PAXXG096210	uncharacterized protein, homologue of XP_020585184.1
			PAXXG132900	Transporter, the H+- or Na+-translocating F-type, V-type and A-type ATPase (F-ATPase) Superfamily
			PAXXG132920	RNA demethylase ALKBH5-like
			PAXXG132930	conserved protein of unknown function, supported by RNASeq data
			PAXXG132940	pyridoxal kinase isoform X1
1	L01	13404324~18900535	PAXXG224870	SWR1-complex protein 4 isoform X2
			PAXXG224880	Similar to retrovirus-related Pol polyprotein from transposon TNT 1-94
			PAXXG224910	uncharacterized protein, homologue of XP_020593563.1
			PAXXG224920	conserved protein of unknown function, supported by RNASeq data
			PAXXG224930	protein ROOT HAIR DEFECTIVE 3-like isoform X1
			PAXXG224940	transmembrane 9 superfamily member 7-like
			PAXXG271670	RNA demethylase ALKBH5-like
			PAXXG271680	RNA demethylase ALKBH5-like
			PAXXG271720	uncharacterized protein, homologue of XP_020592205.1
			PAXXG271740	conserved protein of unknown function, supported by RNASeq data
			PAXXG271750	uncharacterized protein, homologue of XP_020596058.1
			PAXXG271760	HSF transcription factor
			PAXXG271780	MYB-related transcription factor
			PAXXG271790	C2H2 transcription factor
			PAXXG271800	developmentally-regulated G-protein 2
			PAXXG271810	FAR1 transcription factor
			PAXXG197080	expansin-B16-like
			PAXXG197090	MYB-related transcription factor
			PAXXG197110	probable magnesium transporter NIPA8
			PAXXG197120	uncharacterized protein, homologue of XP_020597778.1
			PAXXG197130	uncharacterized protein, homologue of XP_020696325.1
			PAXXG197150	casein kinase I-like isoform X1
			PAXXG197190	uncharacterized protein, homologue of XP_020582885.1
			PAXXG197200	IQ domain-containing protein IQM1-like
			PAXXG197210	uncharacterized protein, homologue of XP_020596678.1
			PAXXG197220	RNA demethylase ALKBH5-like
			PAXXG197240	conserved hypothetical protein homologue of PKU67230.1
			PAXXG197330	uncharacterized protein, homologue of XP_020597546.1
			PAXXG197380	Unknown protein, supported by RNA-Seq data, weak similar to PKU67230.1
			PAXXG197410	uncharacterized protein, homologue of XP_020598317.1
			PAXXG197440	Transporter, Vesicle transport v-SNARE family protein
			PAXXG197460	RNA demethylase ALKBH5-like
			PAXXG197490	conserved hypothetical protein homologue of PKU67230.1
			PAXXG197510	uncharacterized protein, homologue of XP_020599741.1
			PAXXG206270	beclin-1-like protein
			PAXXG206280	uncharacterized protein, homologue of XP_020595548.1
			PAXXG206320	uncharacterized protein, homologue of XP_020598333.1
			PAXXG206330	uncharacterized protein, homologue of XP_020599657.1
			PAXXG206340	probable polygalacturonase
			PAXXG206350	putative pentatricopeptide repeat-containing protein At5g59200, chloroplastic
			PAXXG206360	RNA demethylase ALKBH5-like

			PAXXG206440	RNA demethylase ALKBH5-like
			PAXXG206450	probable protein phosphatase 2C 51
			PAXXG206470	conserved protein of unknown function, supported by RNASeq data
			PAXXG206510	hypothetical protein, weak similar to PKU67230.1
			PAXXG206520	protein SEH1 isoform X1
			PAXXG206580	uncharacterized protein, homologue of XP_020598766.1
			PAXXG206600	beclin-1-like protein
			PAXXG206610	FAR1 transcription factor
			PAXXG206650	RNA demethylase ALKBH5-like
			PAXXG206660	uncharacterized protein, homologue of XP_020592251.1
			PAXXG206670	spermine synthase isoform X2
			PAXXG206700	RNA demethylase ALKBH5-like
			PAXXG206710	uncharacterized protein, homologue of XP_020599372.1
			PAXXG208500	Developmentally regulated G-protein 2
			PAXXG208510	RNA demethylase ALKBH5-like
			PAXXG208560	uncharacterized protein, homologue of XP_020597778.1
			PAXXG208570	RNA demethylase ALKBH5-like
			PAXXG208610	Transporter, the Type II (General) Secretory Pathway (IISP) Family
			PAXXG208630	conserved protein of unknown function, supported by RNASeq data
			PAXXG208650	uncharacterized protein, homologue of XP_020599700.1
			PAXXG208710	uncharacterized protein, homologue of XP_020598317.1
			PAXXG208740	RNA demethylase ALKBH5-like
			PAXXG208790	bHLH transcription factor
			PAXXG208800	RNA demethylase ALKBH5-like
			PAXXG208850	bHLH transcription factor
			PAXXG208880	RNA demethylase ALKBH5-like
			PAXXG208980	uncharacterized protein, homologue of XP_020598317.1
			PAXXG208990	uncharacterized protein, homologue of XP_020598317.1
			PAXXG226730	uncharacterized protein, homologue of XP_020599700.1
			PAXXG226740	uncharacterized protein, homologue of XP_020581221.1
			PAXXG226750	RNA-directed DNA polymerase like
			PAXXG226780	uncharacterized protein, homologue of XP_020596058.1
			PAXXG226790	RNA demethylase ALKBH5-like
			PAXXG226810	uncharacterized protein, homologue of XP_020593563.1
			PAXXG226830	conserved hypothetical protein homologue of PKU82262.1
			PAXXG226880	conserved hypothetical protein homologue of PKA64917.1
			PAXXG226890	uncharacterized protein, homologue of XP_020596664.1
			PAXXG226960	uncharacterized protein, homologue of XP_020580415.1
			PAXXG227020	myosin ID heavy chain
			PAXXG227030	RNA demethylase ALKBH5-like
			PAXXG227060	uncharacterized protein, homologue of XP_020598468.1
			PAXXG227090	uncharacterized protein, homologue of XP_020599868.1
			PAXXG227110	uncharacterized protein, homologue of XP_020597516.1
			PAXXG227120	uncharacterized protein, homologue of XP_020595915.1
			PAXXG227130	uncharacterized protein, homologue of XP_020599700.1
			PAXXG227150	uncharacterized protein, homologue of XP_020593230.1
			PAXXG227160	conserved hypothetical protein homologue of PKA65083.1
			PAXXG317600	NADH-ubiquinone oxidoreductase chain 2
			PAXXG317640	conserved protein of unknown function, supported by RNASeq data
			PAXXG317650	IAA hydrolase
14	L14	268409~5685122	PAXXG297140	rhomboid protein Phaam_RBL10
			PAXXG297150	uncharacterized protein, homologue of XP_020593394.1
			PAXXG297160	conserved protein of unknown function, supported by RNASeq data
			PAXXG297170	pectinesterase inhibitor 9-like
			PAXXG297200	uncharacterized protein At1g04910 isoform X1
			PAXXG204980	serine/threonine-protein kinase CTR1 isoform X2
			PAXXG204990	conserved protein of unknown function, supported by RNASeq data
			PAXXG205010	conserved hypothetical protein homologue of PKU67230.1
			PAXXG205050	Hypothetical protein, supported by RNA-Seq data
			PAXXG205060	VIN3-like protein 2 isoform X2
			PAXXG205070	uncharacterized protein, homologue of XP_020597234.1
			PAXXG205120	uncharacterized protein, homologue of XP_020597546.1
			PAXXG205140	uncharacterized protein, homologue of XP_020576865.1
			PAXXG205200	uncharacterized protein, homologue of XP_020598317.1
			PAXXG205220	uncharacterized protein, homologue of XP_020596869.1
			PAXXG205240	uncharacterized protein, homologue of XP_020599372.1
			PAXXG205250	uncharacterized protein, homologue of XP_020598859.1
			PAXXG205340	uncharacterized protein, homologue of XP_020598317.1
			PAXXG205350	putative mitochondrial protein
			PAXXG205380	uncharacterized protein, homologue of XP_020595829.1
			PAXXG260730	expansin-B16-like
			PAXXG260740	protein OPI10 homolog
			PAXXG260770	cytochrome c oxidase assembly protein COX11, mitochondrial isoform X1
			PAXXG260780	conserved protein of unknown function, supported by RNASeq data
			PAXXG260790	conserved protein of unknown function, supported by RNASeq data
			PAXXG260800	conserved protein of unknown function, supported by RNASeq data
			PAXXG260810	protein FAR1-RELATED SEQUENCE 5-like
			PAXXG260820	anaphase-promoting complex subunit 6
			PAXXG260830	uncharacterized protein, homologue of XP_020599770.1
			PAXXG260840	conserved protein of unknown function, supported by RNASeq data
			PAXXG260850	uncharacterized protein, homologue of XP_020596815.1
			PAXXG260860	Transporter, the Proton-dependent Oligopeptide Transporter (POT) Family
			PAXXG260870	expansin-B16-like
			PAXXG260880	uncharacterized protein, homologue of XP_020598499.1
			PAXXG260890	RNA demethylase ALKBH5-like
			PAXXG320610	abscisic acid 8'-hydroxylase 3-like
			PAXXG320650	RNA demethylase ALKBH5-like
			PAXXG320670	uncharacterized protein, homologue of XP_020599644.1
			PAXXG320720	RNA demethylase ALKBH5-like
			PAXXG320730	uncharacterized protein, homologue of XP_020598333.1
			PAXXG320750	RING finger protein 44-like
			PAXXG320760	uncharacterized protein, homologue of XP_020596058.1

			PAXXG344570	RNA demethylase ALKBH5-like
			PAXXG344580	ent-kaur-16-ene synthase, chloroplastic-like
			PAXXG203140	uncharacterized protein, homologue of XP_020598317.1
			PAXXG203150	uncharacterized protein, homologue of XP_020593262.1
			PAXXG203160	uncharacterized protein, homologue of XP_020593563.1
			PAXXG203190	RNA demethylase ALKBH5-like
			PAXXG203200	uncharacterized protein, homologue of XP_020592067.1
			PAXXG203210	RNA demethylase ALKBH5-like
			PAXXG203290	uncharacterized protein, homologue of XP_020587211.1
			PAXXG203310	uncharacterized protein, homologue of XP_020581952.1
			PAXXG203320	conserved protein of unknown function, supported by RNASeq data
			PAXXG203330	RNA demethylase ALKBH5-like
			PAXXG203370	RNA demethylase ALKBH5-like
			PAXXG203380	uncharacterized protein, homologue of XP_020598403.1
			PAXXG203400	uncharacterized protein, homologue of XP_020587211.1
			PAXXG203410	uncharacterized protein, homologue of XP_020582885.1
			PAXXG203420	uncharacterized protein, homologue of XP_020582885.1
			PAXXG203430	RNA demethylase ALKBH5-like
			PAXXG203460	choline-phosphate cytidylyltransferase 2-like
			PAXXG203480	uncharacterized protein, homologue of XP_020597433.1
			PAXXG203490	uncharacterized protein, homologue of XP_020576750.1
			PAXXG203510	uncharacterized protein, homologue of XP_020599700.1
			PAXXG361000	disease resistance protein RGA2-like
			PAXXG361010	uncharacterized protein, homologue of XP_020597999.1
			PAXXG361020	uncharacterized protein, homologue of XP_020594922.1
			PAXXG361030	FAR1 transcription factor
			PAXXG361050	uncharacterized protein, homologue of XP_020595757.1
			PAXXG190660	uncharacterized protein, homologue of XP_020592067.1
			PAXXG190700	RNA demethylase ALKBH5-like
			PAXXG190710	RNA demethylase ALKBH5-like
			PAXXG190740	uncharacterized protein, homologue of XP_020685009.1
			PAXXG190760	RNA demethylase ALKBH5-like
			PAXXG190770	uncharacterized protein, homologue of XP_020593563.1
			PAXXG190780	L10-interacting MYB domain-containing protein-like
			PAXXG190810	uncharacterized protein, homologue of XP_020582885.1
			PAXXG190830	conserved hypothetical protein homologue of PKU67230.1
			PAXXG190840	abscisic acid 8'-hydroxylase 3-like
			PAXXG190850	uncharacterized protein, homologue of XP_020585518.1
			PAXXG190880	uncharacterized protein, homologue of XP_020598317.1
			PAXXG190890	uncharacterized protein, homologue of XP_020575046.1
			PAXXG190900	thioredoxin-like 4, chloroplastic
			PAXXG190910	alpha-amylase isozyme 3C
			PAXXG190920	Unknown protein, supported by RNA-Seq data
			PAXXG190930	uncharacterized protein, homologue of XP_020596058.1
			PAXXG191000	protein WEAK CHLOROPLAST MOVEMENT UNDER BLUE LIGHT 1-like
			PAXXG191030	uncharacterized protein, homologue of XP_020596058.1
			PAXXG191040	conserved hypothetical protein homologue of PKU67230.1
			PAXXG191050	conserved hypothetical protein homologue of PKU67230.1
			PAXXG191060	conserved protein of unknown function, supported by RNASeq data
			PAXXG191080	RNA demethylase ALKBH5-like
			PAXXG191090	probable protein phosphatase 2C 12 isoform X1
			PAXXG191100	conserved hypothetical protein homologue of PKU67230.1
			PAXXG191110	conserved hypothetical protein homologue of PKU67230.1
			PAXXG315860	conserved protein of unknown function, supported by RNASeq data
			PAXXG315880	methionine aminopeptidase 1D, chloroplastic/mitochondrial
			PAXXG315885	uncharacterized protein, homologue of XP_020593905.1
			PAXXG315890	uncharacterized protein, homologue of XP_020593563.1
			PAXXG315900	Pentatricopeptide repeat-containing protein
			PAXXG315910	uncharacterized protein, homologue of XP_020577273.1
			PAXXG283470	uncharacterized protein, homologue of XP_020596058.1
			PAXXG283490	polyadenylate-binding protein RBP47-like
			PAXXG283500	serine carboxypeptidase II-3-like
			PAXXG283530	conserved protein of unknown function, supported by RNASeq data
			PAXXG283540	alternative NAD(P)H-ubiquinone oxidoreductase C1, chloroplastic/mitochondrial
			PAXXG283550	L10-interacting MYB domain-containing protein-like
13	L13	11937160~17071711	PAXXG196590	RNA demethylase ALKBH5-like
			PAXXG196620	uncharacterized protein, homologue of XP_020595835.1
			PAXXG196630	RNA demethylase ALKBH5-like
			PAXXG196650	expansin-B16-like
			PAXXG196690	conserved protein of unknown function, supported by RNASeq data
			PAXXG196700	extra-large guanine nucleotide-binding protein 3
			PAXXG196730	uncharacterized protein, homologue of XP_020595835.1
			PAXXG196780	26S proteasome non-ATPase regulatory subunit 13 homolog B-like
			PAXXG196810	RNA demethylase ALKBH5-like
			PAXXG196840	uncharacterized protein, homologue of XP_020592225.1
			PAXXG196850	uncharacterized protein, homologue of XP_020596374.1
			PAXXG196860	uncharacterized protein, homologue of XP_020597793.1
			PAXXG196880	uncharacterized protein, homologue of XP_020599770.1
			PAXXG196910	uncharacterized protein, homologue of XP_020581870.1
			PAXXG196940	uncharacterized protein, homologue of XP_020599700.1
			PAXXG196960	FAR1 transcription factor
			PAXXG197010	uncharacterized protein, homologue of XP_020574258.1
			PAXXG197020	uncharacterized protein, homologue of XP_020595835.1
			PAXXG197040	uncharacterized protein, homologue of XP_020596374.1
			PAXXG197070	uncharacterized protein, homologue of XP_020599372.1
			PAXXG132230	Transporter, the YggT or Fanciful K+ Uptake-B (FkuB; YggT) Family
			PAXXG132240	uncharacterized protein, homologue of XP_020599834.1
			PAXXG132250	RING-H2 finger protein ATL8-like
			PAXXG132270	uncharacterized protein, homologue of XP_020592251.1
			PAXXG132320	uncharacterized protein, homologue of XP_020588634.1
			PAXXG132370	RNA demethylase ALKBH5-like
			PAXXG132380	uncharacterized protein, homologue of XP_020596333.1

			PAXXG132400	Transporter, the K+ Uptake Permease (KUP) Family
			PAXXG132410	RNA demethylase ALKBH5-like
			PAXXG132420	uncharacterized protein, homologue of XP_020582885.1
			PAXXG132450	RNA demethylase ALKBH5-like
			PAXXG132460	protein ROS1-like
			PAXXG132490	N-alpha-acetyltransferase 40 isoform X2
			PAXXG132510	uncharacterized protein, homologue of XP_020598317.1
			PAXXG132520	uncharacterized protein, homologue of XP_020593563.1
			PAXXG132530	RNA demethylase ALKBH5-like
			PAXXG132550	actin
			PAXXG132570	apoptosis-inducing factor homolog B-like
			PAXXG132580	uncharacterized protein, homologue of XP_020584155.1
			PAXXG132590	uncharacterized protein, homologue of XP_020594067.1
			PAXXG132630	G-type lectin S-receptor-like serine/threonine-protein kinase At5g24080
			PAXXG132650	uncharacterized protein, homologue of XP_020586152.1
			PAXXG132690	RNA demethylase ALKBH5-like
			PAXXG132710	uncharacterized protein, homologue of XP_020594658.1
			PAXXG132750	Hypothetical protein, supported by RNA-Seq data
			PAXXG132780	uncharacterized protein, homologue of XP_020598333.1
			PAXXG132790	RNA demethylase ALKBH5-like
			PAXXG132800	RNA demethylase ALKBH5-like
			PAXXG132850	conserved hypothetical protein homologue of PKU60200.1
			PAXXG312790	vacuolar protein sorting-associated protein 41 homolog
			PAXXG312800	uncharacterized protein, homologue of XP_020593563.1
			PAXXG312820	conserved hypothetical protein homologue of PKU67230.1
			PAXXG312830	hypothetical protein, weak similar to PKU67230.1
			PAXXG312840	RNA demethylase ALKBH5-like
			PAXXG273410	hypothetical protein, weak similar to PKU67230.1
			PAXXG273420	uncharacterized protein, homologue of XP_020585184.1
			PAXXG273450	uncharacterized protein, homologue of XP_020593358.1
			PAXXG273460	Unknown protein, supported by RNA-Seq data, weak similar to PKU64705.1
			PAXXG273470	uncharacterized protein, homologue of XP_020596058.1
			PAXXG273520	uncharacterized protein, homologue of XP_020596058.1
			PAXXG273560	HEAT repeat-containing protein 6 isoform X1
			PAXXG273570	uncharacterized protein, homologue of XP_020593905.1
			PAXXG273580	ruBisCO large subunit-binding protein subunit alpha-like
			PAXXG273600	ataxia telangiectasia mutated family protein
			PAXXG273610	uncharacterized protein, homologue of XP_020592067.1
			PAXXG248750	uncharacterized protein, homologue of XP_020599258.1
			PAXXG248810	uncharacterized protein, homologue of XP_020595835.1
			PAXXG248840	uncharacterized protein, homologue of XP_020595915.1
			PAXXG248850	uncharacterized protein, homologue of XP_020587211.1
			PAXXG248860	uncharacterized protein, homologue of XP_020595915.1
			PAXXG248880	uncharacterized protein, homologue of XP_020595835.1
			PAXXG248900	RNA demethylase ALKBH5-like
			PAXXG248940	uncharacterized protein, homologue of XP_020581952.1
			PAXXG248970	conserved hypothetical protein homologue of PKU60757.1
			PAXXG248980	uncharacterized protein, homologue of XP_020595656.1
			PAXXG248990	RNA demethylase ALKBH5-like
			PAXXG249010	uncharacterized protein, homologue of XP_020598317.1
			PAXXG249060	conserved protein of unknown function, supported by RNASeq data
			PAXXG249070	uncharacterized protein, homologue of XP_020591803.1
			PAXXG249090	FAR1 transcription factor
			PAXXG249100	HSF transcription factor
			PAXXG096220	DEAD-box ATP-dependent RNA helicase 10-like
			PAXXG096230	dolichyl-diphosphooligosaccharide--protein glycosyltransferase subunit 1A isoform X1
			PAXXG096240	protein kinase and PP2C-like domain-containing protein isoform X3
			PAXXG096250	uncharacterized protein, homologue of XP_020599657.1
			PAXXG096280	conserved protein of unknown function, supported by RNASeq data
			PAXXG096310	uncharacterized protein, homologue of XP_020598908.1
			PAXXG096320	RNA demethylase ALKBH5-like
			PAXXG096340	protein TPR2-like isoform X2
			PAXXG096350	RNA demethylase ALKBH5-like
			PAXXG096360	uncharacterized protein, homologue of XP_020573320.1
			PAXXG096390	probable hydroxyacylglutathione hydrolase 2, chloroplastic isoform X1
11	L11	14173526~19306527	PAXXG151320	uncharacterized protein, homologue of XP_020582885.1
			PAXXG151360	uncharacterized protein, homologue of XP_020582885.1
			PAXXG151370	uncharacterized protein, homologue of XP_020582885.1
			PAXXG151380	uncharacterized protein, homologue of XP_020591844.1
			PAXXG151430	protein MODIFIER OF SNC1 11
			PAXXG151440	uncharacterized protein, homologue of XP_020597161.1
			PAXXG151450	uncharacterized protein, homologue of XP_020599834.1
			PAXXG151460	uncharacterized protein, homologue of XP_020590139.1
			PAXXG151480	conserved hypothetical protein homologue of PKU67230.1
			PAXXG151490	uncharacterized protein, homologue of XP_020598317.1
			PAXXG151530	DNA repair protein RAD51 homolog 4 isoform X2
			PAXXG151540	uncharacterized protein, homologue of XP_020596175.1
			PAXXG151550	Unknown protein, supported by RNA-Seq data, weak similar to PKU67230.1
			PAXXG151580	uncharacterized protein, homologue of XP_020593563.1
			PAXXG151590	uncharacterized protein, homologue of XP_020598317.1
			PAXXG151600	Similar to retrovirus-related Pol polyprotein from transposon TNT 1-94
			PAXXG151630	Similar to retrovirus-related Pol polyprotein from transposon TNT 1-94
			PAXXG151650	uncharacterized protein, homologue of XP_020598667.1
			PAXXG151670	uncharacterized protein, homologue of XP_020681907.1
			PAXXG151680	uncharacterized protein, homologue of XP_020596815.1
			PAXXG151690	receptor-like protein kinase HSL1
			PAXXG151695	Unknown protein, supported by RNA-Seq data
			PAXXG151700	uncharacterized protein, homologue of XP_020595835.1
			PAXXG151710	protein FAR1-RELATED SEQUENCE 3-like
			PAXXG151720	SAC3 family protein A isoform X1
			PAXXG151740	RNA demethylase ALKBH5-like
			PAXXG337920	selT-like protein

			PAXXG337930	monothiol glutaredoxin-S12, chloroplastic
			PAXXG337950	uncharacterized protein, homologue of XP_020596664.1
			PAXXG337960	FAR1 transcription factor
			PAXXG337970	ran-binding protein 1 homolog a-like
			PAXXG337980	conserved hypothetical protein homologue of PKU67230.1
			PAXXG337990	uncharacterized protein, homologue of XP_020592067.1
			PAXXG230710	calcium-dependent protein kinase 3-like
			PAXXG230730	uncharacterized protein, homologue of XP_020599346.1
			PAXXG230740	Transporter, the Mitochondrial Carrier (MC) Family
			PAXXG230750	uncharacterized protein, homologue of XP_020598413.1
			PAXXG230780	uncharacterized protein, homologue of XP_020584790.1
			PAXXG230790	NF-YB transcription factor
			PAXXG230810	putative serine carboxypeptidase-like 23
			PAXXG230820	Transporter, the Sulfate Permease (SulP) Family
			PAXXG230860	RNA demethylase ALKBH5-like
			PAXXG230870	RNA demethylase ALKBH5-like
			PAXXG230890	phosphatidate phosphatase PAH1-like
			PAXXG127150	RNA demethylase ALKBH5-like
			PAXXG127160	conserved hypothetical protein homologue of PKU67230.1
			PAXXG127200	RNA demethylase ALKBH5-like
			PAXXG127210	uncharacterized protein, homologue of XP_020593563.1
			PAXXG127260	RNA demethylase ALKBH5-like
			PAXXG127270	uncharacterized protein, homologue of XP_020596225.1
			PAXXG127280	uncharacterized protein, homologue of XP_020596146.1
			PAXXG127290	CCT domain protein
			PAXXG127300	conserved hypothetical protein homologue of PKA52186.1
			PAXXG127320	uncharacterized protein, homologue of XP_020595915.1
			PAXXG127390	RNA demethylase ALKBH5-like
			PAXXG127410	uncharacterized protein, homologue of XP_020598333.1
			PAXXG127420	RNA demethylase ALKBH5-like
			PAXXG127430	conserved hypothetical protein homologue of PKU67230.1
			PAXXG127490	uncharacterized protein, homologue of XP_020596058.1
			PAXXG127540	conserved hypothetical protein homologue of PKU67230.1
			PAXXG127560	uncharacterized protein, homologue of XP_020597185.1
			PAXXG127620	ataxia telangiectasia mutated family protein
			PAXXG127630	dirigent protein 17-like
			PAXXG127650	putative respiratory burst oxidase homolog protein H isoform X1
			PAXXG127670	uncharacterized protein, homologue of XP_020599700.1
			PAXXG127710	uncharacterized protein, homologue of XP_020596058.1
			PAXXG127750	RNA demethylase ALKBH5-like
			PAXXG127760	ataxia telangiectasia mutated family protein
			PAXXG127780	uncharacterized protein, homologue of XP_020598165.1
			PAXXG127790	uncharacterized protein, homologue of XP_020585569.1
			PAXXG127820	protein TONNEAU 1b-like
			PAXXG127830	conserved hypothetical protein homologue of PKU67230.1
			PAXXG127840	glycine-rich protein A3-like isoform X1
			PAXXG127850	uncharacterized protein, homologue of XP_020584155.1
			PAXXG127870	uncharacterized protein, homologue of XP_020599372.1
			PAXXG127880	uncharacterized protein, homologue of XP_020592067.1
			PAXXG127910	conserved protein of unknown function, supported by RNASeq data
			PAXXG127920	uncharacterized protein, homologue of XP_020595915.1
			PAXXG127940	RNA demethylase ALKBH5-like
			PAXXG128050	uncharacterized protein, homologue of XP_020598317.1
			PAXXG128090	conserved hypothetical protein homologue of PKU67230.1
			PAXXG128100	RNA demethylase ALKBH5-like
			PAXXG128110	serine/arginine-rich splicing factor RS2Z33-like
			PAXXG128140	conserved protein of unknown function, supported by RNASeq data
			PAXXG128180	uncharacterized protein, homologue of XP_020593563.1
			PAXXG128210	conserved protein of unknown function, supported by RNASeq data
			PAXXG128215	stearoyl-
			PAXXG280020	conserved hypothetical protein homologue of PKU67230.1
			PAXXG280030	conserved hypothetical protein homologue of PKA47723.1
			PAXXG280040	expansin-A4-like
			PAXXG280050	Hypothetical protein, supported by RNA-Seq data
			PAXXG280070	uncharacterized protein, homologue of XP_020598317.1
			PAXXG280080	conserved protein of unknown function, supported by RNASeq data
			PAXXG280100	uncharacterized protein, homologue of XP_020590406.1
			PAXXG280110	uncharacterized protein, homologue of XP_020598318.1
			PAXXG280120	conserved protein of unknown function, supported by RNASeq data
			PAXXG280200	telomere repeat-binding protein 2-like
			PAXXG280240	uncharacterized protein, homologue of XP_020685009.1
			PAXXG280270	oxysterol-binding protein-related protein 3A-like
			PAXXG354710	protein MALE DISCOVERER 2-like
			PAXXG370710	uncharacterized protein At4g08330, chloroplastic-like, partial
9	L09	17003521~21281543	PAXXG268230	Transporter, the ATP-binding Cassette (ABC) Superfamily
			PAXXG268240	uncharacterized protein, homologue of XP_020595677.1
			PAXXG268250	RNA demethylase ALKBH5-like
			PAXXG268290	RNA demethylase ALKBH5-like
			PAXXG268310	uncharacterized protein, homologue of XP_020595835.1
			PAXXG109110	probable receptor-like protein kinase At5g24010
			PAXXG109130	uncharacterized protein, homologue of XP_020599223.1
			PAXXG109140	uncharacterized protein, homologue of XP_020595915.1
			PAXXG109160	uncharacterized protein, homologue of XP_020591912.1
			PAXXG109170	uncharacterized protein, homologue of XP_020597156.1
			PAXXG109180	uncharacterized protein, homologue of XP_020599640.1
			PAXXG109200	uncharacterized protein, homologue of XP_020595548.1
			PAXXG109250	RNA demethylase ALKBH5-like
			PAXXG109310	uncharacterized protein, homologue of XP_020595915.1
			PAXXG109350	conserved protein of unknown function, supported by RNASeq data
			PAXXG109410	RNA demethylase ALKBH5-like
			PAXXG109440	uncharacterized protein, homologue of XP_020597778.1
			PAXXG109500	uncharacterized protein, homologue of XP_020570899.1

			PAXXG109560	uncharacterized protein, homologue of XP_020595915.1
			PAXXG109570	uncharacterized protein, homologue of XP_020598859.1
			PAXXG109590	E3 ubiquitin-protein ligase PUB23-like
			PAXXG109600	uncharacterized protein, homologue of XP_020596058.1
			PAXXG109690	RNA demethylase ALKBH5-like
			PAXXG109730	uncharacterized protein, homologue of XP_020592251.1
			PAXXG109750	RNA demethylase ALKBH5-like
			PAXXG109800	RNA demethylase ALKBH5-like
			PAXXG109810	uncharacterized protein, homologue of XP_020694248.1
			PAXXG109820	uncharacterized protein, homologue of XP_020593563.1
			PAXXG109830	Similar to retrovirus-related Pol polyprotein from transposon TNT 1-94
			PAXXG109840	Unknown protein, supported by RNA-Seq data
			PAXXG109850	Unknown protein, supported by RNA-Seq data, weak similar to PKU67230.1
			PAXXG109860	uncharacterized protein, homologue of XP_020598317.1
			PAXXG109870	3-hydroxyisobutyryl-CoA hydrolase 1-like
			PAXXG109920	putative mitochondrial protein
			PAXXG240400	uncharacterized protein, homologue of XP_020596058.1
			PAXXG240420	RNA demethylase ALKBH5-like
			PAXXG240470	uncharacterized protein, homologue of XP_020598859.1
			PAXXG240480	uncharacterized protein, homologue of XP_020595955.1
			PAXXG240490	bifunctional dTDP-4-dehydrorhamnose 3,5-epimerase/dTDP-4-dehydrorhamnose reductase isoform X1
			PAXXG240530	uncharacterized protein, homologue of XP_020599500.1
			PAXXG240540	uncharacterized protein, homologue of XP_020599868.1
			PAXXG240610	AP2/ERF-ERF transcription factor
			PAXXG240620	uncharacterized protein, homologue of XP_020582885.1
			PAXXG240640	uncharacterized protein, homologue of XP_020598333.1
			PAXXG240650	uncharacterized protein, homologue of XP_020598333.1
			PAXXG240670	RNA demethylase ALKBH5-like
			PAXXG240700	conserved protein of unknown function, supported by RNASeq data
			PAXXG240730	Unknown protein, supported by RNA-Seq data
			PAXXG240740	hypothetical protein, weak similar to XP_020599657.1
			PAXXG240760	uncharacterized protein, homologue of XP_020593563.1
			PAXXG240770	uncharacterized protein, homologue of XP_020598333.1
			PAXXG240810	RNA demethylase ALKBH5-like
			PAXXG240820	uncharacterized protein, homologue of XP_020598318.1
			PAXXG240850	RNA demethylase ALKBH5-like
			PAXXG256470	uncharacterized protein, homologue of XP_020576306.1
			PAXXG256480	Transporter, the Nucleobase:Cation Symporter-2 (NCS2) Family
			PAXXG256490	Hypothetical protein, supported by RNA-Seq data
			PAXXG256500	WD repeat-containing protein WRAP73
			PAXXG256510	uncharacterized protein, homologue of XP_020573320.1
			PAXXG256540	deSI-like protein At4g17486 isoform X3
			PAXXG256550	cysteine-rich receptor-like protein kinase 2
			PAXXG344010	uncharacterized protein At3g06530 isoform X1
			PAXXG344030	RNA demethylase ALKBH5-like
			PAXXG231080	ceramide kinase isoform X1
			PAXXG231090	protein ENL-like
			PAXXG231100	phosphatidylinositol N-acetylglucosaminyltransferase subunit C
6	L06	7305145~11284910	PAXXG267480	CMP-sialic acid transporter 4-like isoform X2
			PAXXG267490	Similar to retrovirus-related Pol polyprotein from transposon TNT 1-94
			PAXXG267510	F-box protein At1g47056
			PAXXG267520	uncharacterized protein, homologue of XP_020575838.1
			PAXXG267530	Unknown protein, supported by RNA-Seq data, weak similar to PKU69516.1
			PAXXG267540	probable histone chaperone ASF1A
			PAXXG267560	uncharacterized protein, homologue of XP_020596744.1
			PAXXG267590	RNA demethylase ALKBH5-like
			PAXXG267610	uncharacterized protein, homologue of XP_020596058.1
			PAXXG267620	NAC transcription factor
			PAXXG267630	uncharacterized protein, homologue of XP_020599372.1
			PAXXG267660	uncharacterized protein, homologue of XP_020595829.1
			PAXXG267710	uncharacterized protein, homologue of XP_020598744.1
			PAXXG267750	uncharacterized protein, homologue of XP_020595915.1
			PAXXG267770	uncharacterized protein, homologue of XP_020592387.1
			PAXXG267780	probable apyrase 6
			PAXXG267790	probable apyrase 6
			PAXXG267810	RNA demethylase ALKBH5-like
			PAXXG267840	conserved protein of unknown function, supported by RNASeq data
			PAXXG267850	conserved protein of unknown function, supported by RNASeq data
			PAXXG267880	FAR1 transcription factor
			PAXXG267900	probable inactive ATP-dependent zinc metalloprotease FTSHI 2, chloroplastic
			PAXXG253520	conserved protein of unknown function, supported by RNASeq data
			PAXXG253530	Unknown protein, supported by RNA-Seq data, weak similar to XP_020588264.1
			PAXXG253540	SNF1-related protein kinase regulatory subunit gamma-1
			PAXXG253550	uncharacterized protein, homologue of XP_020571831.1
			PAXXG253570	general transcription factor IIH subunit 2
			PAXXG253620	Unknown protein, supported by RNA-Seq data, weak similar to PKU67230.1
			PAXXG253630	conserved hypothetical protein homologue of PKU67230.1
			PAXXG253640	fidgetin-like protein 1
			PAXXG253650	protein FAR1-RELATED SEQUENCE 5-like
			PAXXG253660	RNA demethylase ALKBH5-like
			PAXXG253690	Transporter, the ATP-binding Cassette (ABC) Superfamily
			PAXXG253720	RNA demethylase ALKBH5-like
			PAXXG253730	conserved protein of unknown function, supported by RNASeq data
			PAXXG289650	uncharacterized protein, homologue of XP_020596058.1
			PAXXG289690	glucose-6-phosphate isomerase 1, chloroplastic isoform X1
			PAXXG289720	RNA demethylase ALKBH5-like
			PAXXG289740	RNA demethylase ALKBH5-like
			PAXXG289750	uncharacterized protein, homologue of XP_020596848.1
			PAXXG289760	RNA demethylase ALKBH5-like
			PAXXG289770	uncharacterized protein, homologue of XP_020596058.1
			PAXXG289800	uncharacterized protein, homologue of XP_020570899.1
			PAXXG289820	RNA demethylase ALKBH5-like

			PAXXG289830	uncharacterized protein, homologue of XP_020596058.1
			PAXXG289860	expansin-B16-like
			PAXXG312450	uncharacterized protein, homologue of XP_020598317.1
			PAXXG312480	uncharacterized protein, homologue of XP_020598317.1
			PAXXG312490	CMP-sialic acid transporter 4-like isoform X1
			PAXXG312500	uncharacterized protein, homologue of XP_020599770.1
			PAXXG312510	uncharacterized protein, homologue of XP_020581952.1
			PAXXG312520	zinc finger HIT domain-containing protein 2
			PAXXG312530	uncharacterized protein, homologue of XP_020588913.1
			PAXXG312540	conserved hypothetical protein homologue of PKU67230.1
			PAXXG135780	L10-interacting MYB domain-containing protein-like
			PAXXG135800	uncharacterized protein, homologue of XP_020577803.1
			PAXXG135810	uncharacterized protein, homologue of XP_020590272.1
			PAXXG135830	uncharacterized protein, homologue of XP_020599700.1
			PAXXG135850	uncharacterized protein, homologue of XP_020595915.1
			PAXXG135860	scopoletin glucosyltransferase-like
			PAXXG135890	uncharacterized protein, homologue of XP_020598317.1
			PAXXG135900	uncharacterized protein, homologue of XP_020593563.1
			PAXXG135910	uncharacterized protein, homologue of XP_020598317.1
			PAXXG135940	Unknown protein, supported by RNA-Seq data, weak similar to PKU67230.1
			PAXXG135960	phosphatidylinositol/phosphatidylcholine transfer protein SFH9-like isoform X2
			PAXXG135980	conserved hypothetical protein homologue of PKA48829.1
			PAXXG135990	conserved protein of unknown function, supported by RNASeq data
			PAXXG136030	uncharacterized protein, homologue of XP_020593563.1
			PAXXG136040	eukaryotic translation initiation factor 4E-1-like isoform X1
			PAXXG136050	conserved hypothetical protein homologue of PKU67230.1
			PAXXG136060	conserved hypothetical protein homologue of PKU67230.1
			PAXXG136070	conserved hypothetical protein homologue of PKU67230.1
			PAXXG136080	RNA demethylase ALKBH5-like
			PAXXG136090	hypothetical protein, weak similar to PKU67230.1
			PAXXG136100	ubiquitin-protein ligase E3 C
			PAXXG136110	uncharacterized protein At4g22758-like
			PAXXG136120	RNA demethylase ALKBH5-like
			PAXXG136150	uncharacterized protein, homologue of XP_020596869.1
			PAXXG136160	RNA demethylase ALKBH5-like
			PAXXG136180	uncharacterized protein, homologue of XP_020596848.1
			PAXXG136210	FAR1 transcription factor
			PAXXG136220	uncharacterized protein At4g22758-like
			PAXXG136230	cysteine-rich receptor-like protein kinase 2
			PAXXG136260	RNA demethylase ALKBH5-like
			PAXXG136270	uncharacterized protein, homologue of XP_020598766.1
			PAXXG136280	conserved protein of unknown function, supported by RNASeq data
			PAXXG136290	RNA demethylase ALKBH5-like
			PAXXG136330	uncharacterized protein, homologue of XP_020598333.1
			PAXXG136340	conserved protein of unknown function, supported by RNASeq data
			PAXXG136380	AT-hook motif nuclear-localized protein 10-like
9	L09	3906838~7589586	PAXXG094450	DNA mismatch repair protein MSH4
			PAXXG094460	CCT domain protein
			PAXXG094470	uncharacterized protein, homologue of XP_020585246.1
			PAXXG094500	E3 ubiquitin-protein ligase BRE1-like 1
			PAXXG094510	uncharacterized protein, homologue of XP_020585221.1
			PAXXG094520	uncharacterized protein, homologue of XP_020585228.1
			PAXXG094525	Similar to PKA53923.1
			PAXXG094540	Transporter, the Oligopeptide Transporter (OPT) Family
			PAXXG094550	Kinesin-like protein KIN-14E
			PAXXG094560	Serine/threonine-protein kinase fray2
			PAXXG094570	Flap endonuclease GEN-like 2
			PAXXG094580	uncharacterized protein, homologue of XP_020595915.1
			PAXXG094590	FAR1 transcription factor
			PAXXG094600	AAA-ATPase
			PAXXG094610	La-related protein 1B
			PAXXG094620	DNA-directed RNA polymerases II, IV and V subunit 9A
			PAXXG094630	DNA-directed RNA polymerases II, IV and V subunit 9B
			PAXXG094640	exonuclease mut-7 homolog
			PAXXG094650	WD repeat-containing protein
			PAXXG094660	MYB transcription factor
			PAXXG094670	Pyridoxine/pyridoxamine 5'-phosphate oxidase 2
			PAXXG094680	Transporter, the Cytochrome Oxidase Biogenesis (Oxa1) Family
			PAXXG094690	FAR1 transcription factor
			PAXXG094700	HB-HD-ZIP transcription factor
			PAXXG094710	RHOMBOID-like protein
			PAXXG094720	methyltransferase PMT13-like
			PAXXG094730	Protein IQ-DOMAIN 32
			PAXXG094740	Serine/threonine-protein kinase
			PAXXG094750	COP9 signalosome complex subunit
			PAXXG094760	Cytochrome P450
			PAXXG094770	putative mitochondrial protein
			PAXXG094780	probable methyltransferase PMT15
			PAXXG094790	E3 ubiquitin ligase BIG BROTHER-like isoform X1
			PAXXG094800	chitin elicitor-binding protein
			PAXXG094810	probable flavin-containing monooxygenase 1
			PAXXG094820	Similar to retrovirus-related Pol polyprotein from transposon TNT 1-94
			PAXXG094840	probable flavin-containing monooxygenase 1
			PAXXG094850	F-box protein PP2-A13-like isoform X1
			PAXXG094860	probable transcriptional regulator SLK3
			PAXXG094870	BAG family molecular chaperone regulator 2-like
			PAXXG094880	protein CELLULOSE SYNTHASE INTERACTIVE 3-like
			PAXXG094890	uncharacterized protein, homologue of XP_020579458.1
			PAXXG094900	NDR1/HIN1-like protein 13
			PAXXG094910	uncharacterized protein, homologue of XP_020585649.1
			PAXXG094920	lipid transfer-like protein VAS
			PAXXG094930	uncharacterized protein, homologue of XP_020585655.1

			PAXXG094940	uncharacterized protein, homologue of XP_020585650.1
			PAXXG094950	pentatricopeptide repeat-containing protein At4g25270, chloroplastic
			PAXXG094960	uncharacterized protein, homologue of XP_020577809.1
			PAXXG094980	uncharacterized protein C594.04c isoform X1
			PAXXG095000	uncharacterized protein, homologue of XP_020577869.1
			PAXXG095010	probable cinnamyl alcohol dehydrogenase 1 isoform X2
			PAXXG095020	cinnamyl-alcohol dehydrogenase
			PAXXG095030	probable cinnamyl alcohol dehydrogenase 1 isoform X3
			PAXXG095040	Transporter, the Drug/Metabolite Transporter (DMT) Superfamily
			PAXXG095050	ubiquitin-conjugating enzyme E2 variant 1C-like isoform X1
			PAXXG095060	uncharacterized protein, homologue of XP_020577868.1
			PAXXG095070	uncharacterized protein, homologue of XP_020694460.1
			PAXXG095080	uncharacterized calcium-binding protein At1g02270
			PAXXG095085	uncharacterized protein, homologue of XP_020592436.1
			PAXXG095090	NAC transcription factor
			PAXXG095110	heavy metal-associated isoprenylated plant protein 9 isoform X1
			PAXXG095150	1-acyl-sn-glycerol-3-phosphate acyltransferase 1, chloroplastic-like
			PAXXG095160	heavy metal-associated isoprenylated plant protein 28
			PAXXG095170	Transporter, Heavy metal transport/detoxification superfamily protein
			PAXXG095190	B3 transcription factor
			PAXXG095200	endoribonuclease Dicer homolog 1
			PAXXG095210	uncharacterized protein, homologue of XP_020577880.1
			PAXXG095220	MYB-related transcription factor
			PAXXG095230	flavonol 3'-O-methyltransferase
			PAXXG095240	Unknown protein, supported by RNA-Seq data
			PAXXG095250	uncharacterized protein At5g39865-like, partial
			PAXXG095260	small heat shock protein, chloroplastic-like
			PAXXG095270	glutathione S-transferase U17-like
			PAXXG095280	glutathione S-transferase U18-like
			PAXXG095290	Hypothetical protein, supported by RNA-Seq data
			PAXXG095300	glutathione S-transferase U18-like
			PAXXG095310	Hypothetical protein, supported by RNA-Seq data
			PAXXG095320	glutathione S-transferase U18-like
			PAXXG095330	glutathione S-transferase U15-like
			PAXXG095350	glutathione S-transferase U18-like
			PAXXG095355	Hypothetical protein, supported by RNA-Seq data
			PAXXG095360	CDGSH iron-sulfur domain-containing protein NEET
			PAXXG095370	Transporter, SLAC1 HOMOLOGUE
			PAXXG095380	Unknown protein, supported by RNA-Seq data
			PAXXG095390	protein TPX2-like
			PAXXG095400	FAR1 transcription factor
			PAXXG190220	pEARLI1-like lipid transfer protein
			PAXXG190230	14 kDa proline-rich protein
			PAXXG190240	Oxysterol-binding protein-related protein
			PAXXG190250	Unknown protein, supported by RNASeq data
			PAXXG190260	Lipid phosphate phosphatase beta
			PAXXG190270	Unknown protein, supported by RNASeq data
			PAXXG190280	Flavin-containing monooxygenase
			PAXXG190290	BAG family molecular chaperone regulator
			PAXXG190300	PLATZ transcription factor
			PAXXG190310	uncharacterized protein, homologue of XP_020586896.1
			PAXXG190320	probable xyloglucan galactosyltransferase GT19
			PAXXG190330	probable trehalose-phosphate phosphatase F
			PAXXG190340	uncharacterized protein, homologue of XP_020691250.1
			PAXXG190350	uncharacterized protein, homologue of XP_020704910.1
			PAXXG190360	C2C2-YABBY transcription factor
			PAXXG190370	uncharacterized protein At2g29880-like
			PAXXG190380	probable plastid-lipid-associated protein 10, chloroplastic
			PAXXG190390	cysteine-rich repeat secretory protein 15-like
			PAXXG190400	auxin-responsive protein SAUR36
			PAXXG190410	probable methyltransferase PMT17 isoform X2
			PAXXG190430	transmembrane and coiled-coil domain-containing protein 4-like isoform X1
			PAXXG190440	C2C2-GATA transcription factor
			PAXXG190450	uncharacterized protein, homologue of XP_020592545.1
			PAXXG190460	dual specificity protein phosphatase 1-like
			PAXXG190470	conserved hypothetical protein homologue of PKA48168.1
			PAXXG190480	B-BOX domain protein
			PAXXG190500	aldehyde dehydrogenase family 3 member F1-like
			PAXXG190510	FAR1 transcription factor
			PAXXG190530	Unknown protein, supported by RNA-Seq data, weak similar to PKU67230.1
			PAXXG190540	aldehyde dehydrogenase family 3 member F1-like
			PAXXG190550	uncharacterized protein, homologue of XP_020573320.1
			PAXXG190560	probable inactive leucine-rich repeat receptor kinase XIAO
			PAXXG190570	uncharacterized protein, homologue of XP_020592561.1
			PAXXG190580	FAR1 transcription factor
			PAXXG190590	FAR1-RELATED SEQUENCE 9-like
			PAXXG190600	CCR4-NOT transcription complex subunit 1 isoform X1
			PAXXG190610	uncharacterized protein, homologue of XP_020599770.1
			PAXXG190620	ataxia telangiectasia mutated family protein
			PAXXG190640	conserved hypothetical protein homologue of PKU67230.1
			PAXXG190650	conserved protein of unknown function, supported by RNASeq data
			PAXXG279860	ADP-ribosylation factor-like
			PAXXG279870	conserved protein of unknown function, supported by RNASeq data
			PAXXG279880	uncharacterized protein, homologue of XP_020597493.1
			PAXXG279890	OFP transcription factor
			PAXXG279900	probable protein S-acyltransferase 12
			PAXXG279910	probable lysophospholipase BODYGUARD 3
			PAXXG279920	rho GTPase-activating protein 3-like
			PAXXG279930	uncharacterized protein, homologue of XP_020585235.1
10	L10a	12235612~15801812	PAXXG175380	WAT1-related protein At5g64700-like
			PAXXG175390	L10-interacting MYB domain-containing protein-like
			PAXXG175430	uncharacterized protein, homologue of XP_020598317.1

			PAXXG175440	Transporter, the ATP-binding Cassette (ABC) Superfamily
			PAXXG175480	uncharacterized protein, homologue of XP_020593563.1
			PAXXG175490	L10-interacting MYB domain-containing protein-like
			PAXXG175520	uncharacterized protein, homologue of XP_020596848.1
			PAXXG175560	uncharacterized protein, homologue of XP_020584790.1
			PAXXG175570	uncharacterized protein, homologue of XP_020599700.1
			PAXXG175580	uncharacterized protein, homologue of XP_020598113.1
			PAXXG175600	uncharacterized protein, homologue of XP_020597161.1
			PAXXG175610	uncharacterized protein, homologue of XP_020597903.1
			PAXXG175630	uncharacterized protein, homologue of XP_020595829.1
			PAXXG175660	uncharacterized protein, homologue of XP_020598317.1
			PAXXG175670	uncharacterized protein, homologue of XP_020586896.1
			PAXXG175680	uncharacterized protein, homologue of XP_020586142.1
			PAXXG175690	uncharacterized protein, homologue of XP_020599223.1
			PAXXG175710	uncharacterized protein, homologue of XP_020596374.1
			PAXXG175720	ASC1-like protein
			PAXXG175730	RNA demethylase ALKBH5-like
			PAXXG175770	RNA demethylase ALKBH5-like
			PAXXG175810	uncharacterized protein, homologue of XP_020596058.1
			PAXXG191120	Unknown protein, supported by RNA-Seq data, weak similar to XP_020588264.1
			PAXXG191160	tobamovirus multiplication protein 2B isoform X2
			PAXXG191180	uncharacterized protein, homologue of XP_020585184.1
			PAXXG191200	RNA demethylase ALKBH5-like
			PAXXG191205	RNA demethylase ALKBH5-like
			PAXXG191220	ribose-phosphate pyrophosphokinase 1 isoform X2
			PAXXG191230	60S ribosomal protein L7-2
			PAXXG191240	uncharacterized protein, homologue of XP_020596744.1
			PAXXG191270	uncharacterized protein, homologue of XP_020581871.1
			PAXXG191280	integrator complex subunit 11
			PAXXG191350	glycerol-3-phosphate acyltransferase 1-like
			PAXXG191360	RNA demethylase ALKBH5-like
			PAXXG191370	RNA demethylase ALKBH5-like
			PAXXG191390	uncharacterized protein, homologue of XP_020593563.1
			PAXXG191420	uncharacterized protein, homologue of XP_020595915.1
			PAXXG191460	uncharacterized protein, homologue of XP_020599770.1
			PAXXG191500	RNA demethylase ALKBH5-like
			PAXXG191510	Unknown protein, supported by RNA-Seq data, weak similar to PKU64705.1
			PAXXG191530	RNA demethylase ALKBH5-like
			PAXXG191560	uncharacterized protein, homologue of XP_020582885.1
			PAXXG191570	uncharacterized protein, homologue of XP_020598333.1
			PAXXG191575	conserved protein of unknown function, supported by RNASeq data
			PAXXG191600	conserved hypothetical protein homologue of PKU82912.1
			PAXXG191610	protein MEI2-like 2 isoform X1
			PAXXG191620	Transporter, the Folate-Biopterin Transporter (FBT) Family
			PAXXG191630	conserved hypothetical protein homologue of PKU67230.1
			PAXXG191660	conserved protein of unknown function, supported by RNASeq data
			PAXXG191680	RNA demethylase ALKBH5-like
			PAXXG191690	RNA demethylase ALKBH5-like
			PAXXG256860	uncharacterized protein At2g34460, chloroplastic
			PAXXG256910	RNA-directed DNA polymerase like
			PAXXG256920	conserved hypothetical protein homologue of PKU67230.1
			PAXXG256940	putative disease resistance protein RGA3
			PAXXG257000	uncharacterized protein, homologue of XP_020598317.1
12	L12	11762533~15106456	PAXXG207305	Polyadenylate-binding protein 2
			PAXXG207310	late embryogenesis abundant protein, group 3-like
			PAXXG207320	uncharacterized protein, homologue of XP_020596058.1
			PAXXG207330	uncharacterized protein, homologue of XP_020598333.1
			PAXXG207360	putative cytochrome c oxidase subunit 5b-like
			PAXXG207380	uncharacterized protein, homologue of XP_020584155.1
			PAXXG207410	protein PARTING DANCERS
			PAXXG207480	uncharacterized protein, homologue of XP_020598333.1
			PAXXG207490	RNA demethylase ALKBH5-like
			PAXXG207550	L10-interacting MYB domain-containing protein-like
			PAXXG207570	RNA demethylase ALKBH5-like
			PAXXG207610	uncharacterized protein, homologue of XP_020582885.1
			PAXXG207630	uncharacterized protein, homologue of XP_020598766.1
			PAXXG207660	uncharacterized protein, homologue of XP_020598317.1
			PAXXG207670	uncharacterized protein, homologue of XP_020581952.1
			PAXXG207700	uncharacterized protein, homologue of XP_020575046.1
			PAXXG207710	probable indole-3-pyruvate monooxygenase YUCCA4
			PAXXG207720	RNA demethylase ALKBH5-like
			PAXXG207760	RNA demethylase ALKBH5-like
			PAXXG255990	uncharacterized protein, homologue of XP_020595835.1
			PAXXG256000	uncharacterized protein, homologue of XP_020598317.1
			PAXXG256020	conserved protein of unknown function, supported by RNASeq data
			PAXXG256030	ataxia telangiectasia mutated family protein
			PAXXG256040	hydroquinone glucosyltransferase-like
			PAXXG256050	hydroquinone glucosyltransferase-like
			PAXXG256070	uncharacterized protein, homologue of XP_020598317.1
			PAXXG256160	RNA demethylase ALKBH5-like
			PAXXG256190	DBP transcription factor
			PAXXG256200	ataxia telangiectasia mutated family protein
			PAXXG256220	uncharacterized protein, homologue of XP_020592251.1
			PAXXG256240	uncharacterized protein, homologue of XP_020597546.1
			PAXXG256250	FAR1 transcription factor
			PAXXG327450	conserved hypothetical protein homologue of PKU67230.1
			PAXXG298610	uncharacterized protein, homologue of XP_020705139.1
			PAXXG298620	uncharacterized protein, homologue of XP_020598673.1
			PAXXG298640	ataxia telangiectasia mutated family protein
			PAXXG298650	DNA-binding protein DDB_G0278111 isoform X2
			PAXXG298690	uncharacterized protein, homologue of XP_020596744.1
			PAXXG298710	RNA-directed DNA polymerase like

			PAXXG121080	uncharacterized protein, homologue of XP_020598317.1
			PAXXG121090	DNA replication licensing factor MCM4 isoform X2
			PAXXG121100	RNA demethylase ALKBH5-like
			PAXXG121120	uncharacterized protein, homologue of XP_020595829.1
			PAXXG121130	uncharacterized protein, homologue of XP_020596705.1
			PAXXG121150	uncharacterized protein, homologue of XP_020599700.1
			PAXXG121170	uncharacterized protein, homologue of XP_020599770.1
			PAXXG121190	uncharacterized protein, homologue of XP_020597185.1
			PAXXG121260	uncharacterized protein, homologue of XP_020596058.1
			PAXXG121270	RNA demethylase ALKBH5-like
			PAXXG121275	conserved hypothetical protein homologue of PKU84411.1
			PAXXG121280	phosphatidylcholine:diacylglycerol cholinephosphotransferase 1-like
			PAXXG121310	uncharacterized protein, homologue of XP_020593563.1
			PAXXG121320	uncharacterized protein, homologue of XP_020593563.1
			PAXXG121350	ataxia telangiectasia mutated family protein
			PAXXG121360	uncharacterized protein, homologue of XP_020597165.1
			PAXXG121370	expansin-B16-like
			PAXXG121380	splicing factor SF3a60 homolog isoform X1
12	L12	8014645~11006066	PAXXG346460	uncharacterized protein, homologue of XP_020598355.1
			PAXXG346490	uncharacterized protein, homologue of XP_020596058.1
			PAXXG346510	RNA demethylase ALKBH5-like
			PAXXG346520	uncharacterized protein, homologue of XP_020599469.1
			PAXXG205990	Unknown protein, supported by RNA-Seq data
			PAXXG206000	caffeoylshikimate esterase isoform X1
			PAXXG206030	RNA demethylase ALKBH5-like
			PAXXG206040	L10-interacting MYB domain-containing protein-like
			PAXXG206060	uncharacterized protein, homologue of XP_020593563.1
			PAXXG206090	uncharacterized protein, homologue of XP_020598318.1
			PAXXG206100	uncharacterized protein, homologue of XP_020598317.1
			PAXXG206110	uncharacterized protein, homologue of XP_020598333.1
			PAXXG206150	Unknown protein, supported by RNA-Seq data, weak similar to XP_020588264.1
			PAXXG206170	conserved hypothetical protein homologue of PKU67230.1
			PAXXG206180	conserved protein of unknown function, supported by RNASeq data
			PAXXG206190	polygalacturonase-like
			PAXXG206210	uncharacterized protein, homologue of XP_020592067.1
			PAXXG206220	uncharacterized protein, homologue of XP_020598317.1
			PAXXG206240	uncharacterized protein K02A2.6-like
			PAXXG206250	RNA demethylase ALKBH5-like
			PAXXG157050	uncharacterized protein, homologue of XP_020596043.1
			PAXXG157060	RNA demethylase ALKBH5-like
			PAXXG157070	conserved hypothetical protein homologue of PKU67230.1
			PAXXG157080	RNA demethylase ALKBH5-like
			PAXXG157120	RNA demethylase ALKBH5-like
			PAXXG157130	uncharacterized protein, homologue of XP_020598317.1
			PAXXG157150	conserved protein of unknown function, supported by RNASeq data
			PAXXG157190	uncharacterized protein, homologue of XP_020587505.1
			PAXXG157200	protein LURP-one-related 5
			PAXXG157310	RNA demethylase ALKBH5-like
			PAXXG157320	RNA demethylase ALKBH5-like
			PAXXG157360	bZIP transcription factor
			PAXXG157370	RNA demethylase ALKBH5-like
			PAXXG157400	RNA demethylase ALKBH5-like
			PAXXG157430	uncharacterized protein, homologue of XP_020598317.1
			PAXXG157480	RNA demethylase ALKBH5-like
			PAXXG157540	uncharacterized protein, homologue of XP_020595835.1
			PAXXG157550	conserved protein of unknown function, supported by RNASeq data
			PAXXG157565	uncharacterized protein, homologue of XP_020598674.1
			PAXXG157570	conserved hypothetical protein homologue of PKU67230.1
			PAXXG157580	protein FAM135B-like isoform X1
			PAXXG157590	uncharacterized protein, homologue of XP_020592067.1
			PAXXG157610	RNA demethylase ALKBH5-like
			PAXXG157630	RING-H2 finger protein ATL72-like
			PAXXG157640	uncharacterized protein, homologue of XP_020596058.1
			PAXXG278650	cysteine--tRNA ligase, chloroplastic/mitochondrial-like
			PAXXG278670	conserved protein of unknown function, supported by RNASeq data
			PAXXG278680	protein TOO MANY MOUTHS
			PAXXG278690	protein ABIL1
15	L15	10232403~13195728	PAXXG337640	autophagy-related protein 13b
			PAXXG337710	Transporter, the Vacuolar Iron Transporter (VIT) Family
			PAXXG326680	conserved protein of unknown function, supported by RNASeq data
			PAXXG326690	uncharacterized protein, homologue of XP_020598506.1
			PAXXG326700	putative D-cysteine desulfhydrase 1, mitochondrial
			PAXXG326710	protein CELLULOSE SYNTHASE INTERACTIVE 3
			PAXXG123580	uncharacterized protein, homologue of XP_020593149.1
			PAXXG123600	protein downstream neighbor of Son-like
			PAXXG123605	MADS box protein
			PAXXG123630	RNA demethylase ALKBH5-like
			PAXXG123680	uncharacterized protein, homologue of XP_020599685.1
			PAXXG123690	uncharacterized protein, homologue of XP_020595835.1
			PAXXG123720	Similar to Oxidoreductase
			PAXXG123730	Unknown protein, supported by RNASeq data
			PAXXG123760	Root UVB sensitive
			PAXXG123770	Abscisic acid 8'-hydroxylase
			PAXXG123800	Unknown protein, supported by RNASeq data
			PAXXG123820	Pentatricopeptide repeat-containing protein
			PAXXG123830	Dual specificity protein kinase
			PAXXG123840	Cleavage and polyadenylation specificity factor subunit
			PAXXG123850	Pentatricopeptide repeat-containing protein
			PAXXG123860	Transporter, the Major Facilitator Superfamily (MFS)
			PAXXG123865	Similar to Os12g0106525
			PAXXG123870	Serine/threonine-protein kinase
			PAXXG123880	Pentatricopeptide repeat-containing protein

			PAXXG123890	Unknown protein, supported by RNASeq data
			PAXXG123900	Unknown protein, supported by RNASeq data
			PAXXG123910	Syntaxin-22
			PAXXG123920	Cyclin-dependent kinase
			PAXXG123930	Tankyrase-2
			PAXXG123940	Transporter, the Amino Acid-Polyamine-Organocation (APC) Family
			PAXXG123950	UMP-CMP kinase
			PAXXG123960	Ycf20-like protein
			PAXXG123970	mRNA-capping enzyme
			PAXXG123980	50S ribosomal protein L31
			PAXXG123990	Protein GRAVITROPIC IN THE LIGHT
			PAXXG124000	Unknown protein, supported by RNASeq data
			PAXXG124010	Ovomucoid
			PAXXG124020	MYB-related transcription factor
			PAXXG124030	Similar to retrovirus-related Pol polyprotein from transposon TNT 1-94
			PAXXG124040	24-methylenesterol C-methyltransferase
			PAXXG124050	Unknown protein, supported by RNASeq data
			PAXXG124060	TELO2-interacting protein
			PAXXG124080	Protein IQ-DOMAIN
			PAXXG124090	Unknown protein, supported by RNASeq data
			PAXXG124100	Copper chaperone for superoxide dismutase
			PAXXG124110	Fasciclin-like arabinogalactan protein
			PAXXG124120	Transporter, the Monovalent Cation:Proton Antiporter-2 (CPA2) Family
			PAXXG124130	LRR receptor-like serine/threonine-protein kinase
			PAXXG124140	TVP38/TMEM64 family membrane protein
			PAXXG124150	BBR-BPC transcription factor
			PAXXG124160	Polyprenol reductase
			PAXXG124180	Lysine-specific histone demethylase
			PAXXG124190	Uncharacterized metal-dependent hydrolase
			PAXXG124200	Heavy metal-associated isoprenylated plant protein
			PAXXG124210	Protein-lysine methyltransferase
			PAXXG124230	Unknown protein, supported by RNASeq data
			PAXXG124240	Similar to hypothetical protein AALP_AA4G124900 [Arabis alpina]
			PAXXG124250	Unknown protein, supported by RNASeq data
			PAXXG124260	Pentatricopeptide repeat-containing protein
			PAXXG124270	Coatomer subunit gamma
			PAXXG124280	TPR repeat-containing thioredoxin
			PAXXG124290	Protein COFACTOR ASSEMBLY OF COMPLEX C SUBUNIT
			PAXXG124300	Transcriptional regulator
			PAXXG124310	Similar to PREDICTED: uncharacterized protein LOC107178506 [Citrus sinensis]
			PAXXG124320	Cytochrome P450
			PAXXG124330	Protein IQ-DOMAIN
			PAXXG124340	E3 ubiquitin-protein ligase
			PAXXG124350	Transporter, the Cytochrome Oxidase Biogenesis (Oxa1) Family
			PAXXG124360	HSF transcription factor
			PAXXG124370	Bifunctional UDP-glucose 4-epimerase and UDP-xylose 4-epimerase
			PAXXG124380	3-hexulose-6-phosphate isomerase
			PAXXG124390	Short-chain dehydrogenase
			PAXXG124400	MYB transcription factor
			PAXXG124410	WD repeat-containing protein
			PAXXG124420	ORM1-like protein
			PAXXG124425	uncharacterized protein, homologue of XP_020676612.1
			PAXXG124430	Transporter, the Aromatic Acid Exporter (ArAE) Family
			PAXXG124440	Calcium-binding protein
			PAXXG124450	Unknown protein, supported by RNASeq data
			PAXXG124460	Transporter, the Mitochondrial Protein Translocase (MPT) Family
			PAXXG124470	UPF0160 protein
			PAXXG124480	AAA-ATPase
			PAXXG306410	hypothetical protein, weak similar to PKU67230.1
			PAXXG306420	putative 1-phosphatidylinositol-3-phosphate 5-kinase FAB1D isoform X2
			PAXXG306430	protein FAR1-RELATED SEQUENCE 5-like
			PAXXG306435	protein FAR1-RELATED SEQUENCE 3-like
			PAXXG306440	protein FAR1-RELATED SEQUENCE 3-like
			PAXXG306450	histone-lysine N-methyltransferase ASHR1
			PAXXG306460	conserved hypothetical protein homologue of PKA46090.1
			PAXXG306470	protein EI24 homolog isoform X1
			PAXXG306490	conserved protein of unknown function, supported by RNASeq data
			PAXXG344430	RNA demethylase ALKBH5-like
			PAXXG344440	uncharacterized protein, homologue of XP_020592067.1
			PAXXG344450	G-type lectin S-receptor-like serine/threonine-protein kinase LECRK2
			PAXXG344460	glycine-rich RNA-binding protein RZ1A
			PAXXG344470	G-type lectin S-receptor-like serine/threonine-protein kinase LECRK2
			PAXXG344480	glycine-rich RNA-binding protein RZ1A
			PAXXG344490	uncharacterized protein, homologue of XP_020599770.1
			PAXXG344500	conserved protein of unknown function, supported by RNASeq data
			PAXXG344520	conserved hypothetical protein homologue of PKU67230.1
			PAXXG344530	uncharacterized protein, homologue of XP_020595915.1
			PAXXG328570	probable E3 ubiquitin-protein ligase RNF144A
18	L18	11088488~13781140	PAXXG083550	RNA demethylase ALKBH5-like
			PAXXG083580	uncharacterized protein, homologue of XP_020598859.1
			PAXXG083630	uncharacterized protein, homologue of XP_020595547.1
			PAXXG083660	RNA demethylase ALKBH5-like
			PAXXG083680	MYB-related transcription factor
			PAXXG083690	protein DEFECTIVE IN EXINE FORMATION 1
			PAXXG083700	aspartic proteinase oryzasin-1-like
			PAXXG083710	uncharacterized protein, homologue of XP_020593563.1
			PAXXG083770	uncharacterized protein, homologue of XP_020595536.1
			PAXXG083790	Transporter, the Copper Transporter (Ctr) Family
			PAXXG083800	RNA demethylase ALKBH5-like
			PAXXG083830	probable serine/threonine-protein kinase PBL10
			PAXXG083835	conserved protein of unknown function, supported by RNASeq data
			PAXXG083840	RNA demethylase ALKBH5-like

			PAXXG083890	universal stress protein PHOS32-like
			PAXXG083910	uncharacterized protein, homologue of XP_020598674.1
			PAXXG083920	peptidyl-prolyl cis-trans isomerase PASTICCINO1
			PAXXG084000	glycerol-3-phosphate acyltransferase 5-like
			PAXXG084010	Unknown protein, supported by RNA-Seq data
			PAXXG084020	uncharacterized protein, homologue of XP_020598333.1
			PAXXG084030	uncharacterized protein, homologue of XP_020596848.1
			PAXXG084050	Unknown protein, supported by RNA-Seq data, weak similar to XP_020588264.1
			PAXXG084060	uncharacterized protein, homologue of XP_020596120.1
			PAXXG084070	Similar to retrovirus-related Pol polyprotein from transposon TNT 1-94
			PAXXG084080	protein FIZZY-RELATED 3
			PAXXG236840	conserved protein of unknown function, supported by RNASeq data
			PAXXG236850	glutaredoxin-C1-like
			PAXXG236860	uncharacterized protein, homologue of XP_020700321.1
			PAXXG236870	long chain acyl-CoA synthetase 6, peroxisomal-like
			PAXXG236880	uncharacterized protein, homologue of XP_020597611.1
			PAXXG236890	RNA demethylase ALKBH5-like
			PAXXG236900	putative glutaredoxin-C14
			PAXXG236910	conserved hypothetical protein homologue of PKA48167.1
			PAXXG236920	protein phosphatase inhibitor 2
			PAXXG236930	cytochrome P450 71A9-like
			PAXXG236940	uncharacterized protein, homologue of XP_020597599.1
			PAXXG236950	sucrose nonfermenting 4-like protein isoform X1
			PAXXG236960	splicing factor 3A subunit 2
			PAXXG236970	uncharacterized protein, homologue of XP_020592778.1
			PAXXG236980	cullin-1 isoform X1
			PAXXG236990	dephospho-CoA kinase isoform X1
			PAXXG237000	vesicle-associated membrane protein 724-like
			PAXXG237010	elongator complex protein 5
			PAXXG237020	uncharacterized protein, homologue of XP_020579422.1
			PAXXG237030	CAP-Gly domain-containing linker protein 1
			PAXXG237040	B3 domain-containing protein At2g31420-like
			PAXXG237050	rapid alkalinization factor-like
			PAXXG237060	uncharacterized protein, homologue of XP_020583459.1
			PAXXG237070	protein YLS7
			PAXXG237080	stress-related protein
			PAXXG237120	pentatricopeptide repeat-containing protein At3g06920
			PAXXG237130	kinesin-like protein KIN-13A
			PAXXG342820	Unknown protein, supported by RNA-Seq data
			PAXXG342830	uncharacterized protein, homologue of XP_020599770.1
			PAXXG342860	putative glutaredoxin-C14
			PAXXG342870	BTB/POZ and MATH domain-containing protein 1-like
12	L12	16801094~19462902	PAXXG202980	uncharacterized protein YNL011C isoform X1
			PAXXG202990	expansin-B16-like
			PAXXG203000	protein trichome birefringence-like 37
			PAXXG203010	uncharacterized protein, homologue of XP_020570899.1
			PAXXG203020	hypothetical protein, weak similar to PKU67230.1
			PAXXG203030	uncharacterized protein, homologue of XP_020594913.1
			PAXXG203050	conserved protein of unknown function, supported by RNASeq data
			PAXXG203070	protein KINESIN LIGHT CHAIN-RELATED 1
			PAXXG203080	RNA demethylase ALKBH5-like
			PAXXG203110	conserved hypothetical protein homologue of PKU62872.1
			PAXXG223660	protein ALP1-like
			PAXXG223670	RNA demethylase ALKBH5-like
			PAXXG223730	protein ABIL3-like
			PAXXG223770	uncharacterized protein K02A2.6-like
			PAXXG223780	uncharacterized protein, homologue of XP_020598317.1
			PAXXG223790	conserved hypothetical protein homologue of PKU67230.1
			PAXXG223800	conserved hypothetical protein homologue of PKU67230.1
			PAXXG223810	conserved hypothetical protein homologue of PKU67230.1
			PAXXG223820	uncharacterized protein, homologue of XP_020599644.1
			PAXXG223830	uncharacterized protein, homologue of XP_020582885.1
			PAXXG223840	bHLH transcription factor
			PAXXG223850	uncharacterized protein, homologue of XP_020588264.1
			PAXXG223860	uncharacterized protein, homologue of XP_020590139.1
			PAXXG223870	conserved hypothetical protein homologue of PKU67230.1
			PAXXG223890	Unknown protein, supported by RNA-Seq data, weak similar to PKU69516.1
			PAXXG223900	MYB transcription factor
			PAXXG223930	Transporter, SWEET gene family
			PAXXG223940	conserved protein of unknown function, supported by RNASeq data
			PAXXG223950	conserved hypothetical protein homologue of PKU84154.1
			PAXXG261330	leucine-rich repeat receptor-like serine/threonine-protein kinase BAM3
			PAXXG261340	uncharacterized protein, homologue of XP_020592067.1
			PAXXG261350	uncharacterized protein, homologue of XP_020596500.1
			PAXXG261360	glycosyltransferase-like At2g41451
			PAXXG261370	Transporter, the Voltage-gated Ion Channel (VIC) Superfamily
			PAXXG261380	putative pentatricopeptide repeat-containing protein At5g13230, mitochondrial
			PAXXG261390	DEAD-box ATP-dependent RNA helicase 10-like
			PAXXG310070	nucleoside diphosphate kinase 3-like
			PAXXG310080	CCT domain protein
			PAXXG310120	O-acyltransferase WSD1-like
			PAXXG310150	uncharacterized protein, homologue of XP_020596815.1
			PAXXG310200	origin of replication complex subunit 1
			PAXXG310210	ataxia telangiectasia mutated family protein
2	L02	12595353~15232453	PAXXG177610	serine/threonine-protein kinase HT1-like
			PAXXG177640	uncharacterized protein, homologue of XP_020598499.1
			PAXXG177650	RNA demethylase ALKBH5-like
			PAXXG177660	RNA demethylase ALKBH5-like
			PAXXG177710	uncharacterized protein, homologue of XP_020598333.1
			PAXXG177720	uncharacterized protein, homologue of XP_020593563.1
			PAXXG177750	uncharacterized protein, homologue of XP_020592067.1
			PAXXG177760	Transporter, SWEET gene family

			PAXXG177810	uncharacterized protein, homologue of XP_020597778.1
			PAXXG177840	deoxynucleoside triphosphate triphosphohydrolase SAMHD1 homolog
			PAXXG177850	uncharacterized protein, homologue of XP_020599770.1
			PAXXG177860	uncharacterized protein, homologue of XP_020585390.1
			PAXXG177870	RNA-directed DNA polymerase like
			PAXXG177880	RNA demethylase ALKBH5-like
			PAXXG177910	E3 ubiquitin-protein ligase SIS3 isoform X1
			PAXXG177920	RNA demethylase ALKBH5-like
			PAXXG177930	RNA demethylase ALKBH5-like
			PAXXG177940	uncharacterized protein, homologue of XP_020593563.1
			PAXXG177950	conserved protein of unknown function, supported by RNASeq data
			PAXXG178020	uncharacterized protein, homologue of XP_020596333.1
			PAXXG178030	protein EXECUTER 2, chloroplastic
			PAXXG210790	DEAD-box ATP-dependent RNA helicase 10-like
			PAXXG210800	probable glycosyltransferase At5g03795
			PAXXG210810	uncharacterized protein, homologue of XP_020682038.1
			PAXXG210820	omega-hydroxypalmitate O-feruloyl transferase
			PAXXG210830	cysteine-rich receptor-like protein kinase 2
			PAXXG210860	conserved protein of unknown function, supported by RNASeq data
			PAXXG210870	conserved protein of unknown function, supported by RNASeq data
			PAXXG210880	cytochrome P450 709B2-like
			PAXXG210900	chitinase 10-like
			PAXXG210905	conserved protein of unknown function, supported by RNASeq data
			PAXXG210910	ornithine carbamoyltransferase, chloroplastic
			PAXXG210920	uncharacterized protein, homologue of XP_020596058.1
			PAXXG210950	bifunctional nuclease 2 isoform X1
			PAXXG210960	uncharacterized protein, homologue of XP_020595835.1
			PAXXG210980	uncharacterized protein, homologue of XP_020582058.1
			PAXXG210990	phosphatidylinositol/phosphatidylcholine transfer protein SFH6-like
			PAXXG211040	RNA demethylase ALKBH5-like
			PAXXG211060	uncharacterized protein, homologue of XP_020593563.1
			PAXXG211070	conserved protein of unknown function, supported by RNASeq data
			PAXXG319650	RNA demethylase ALKBH5-like
			PAXXG319660	uncharacterized protein, homologue of XP_020582885.1
			PAXXG319690	MYB transcription factor
			PAXXG319700	pentatricopeptide repeat-containing protein At1g56690, mitochondrial-like
			PAXXG319760	uncharacterized protein, homologue of XP_020593563.1
			PAXXG319770	uncharacterized protein, homologue of XP_020596531.1
			PAXXG335070	RNA demethylase ALKBH5-like
			PAXXG335080	RNA demethylase ALKBH5-like
			PAXXG335110	hypothetical protein
			PAXXG335120	hypothetical protein
			PAXXG335130	hypothetical protein
			PAXXG335140	DnaJ homolog subfamily C member
18	L18	6871150~9295678	PAXXG244170	non-specific lipid-transfer protein P5-like
			PAXXG244180	non-specific lipid-transfer protein 1-like
			PAXXG244230	uncharacterized protein, homologue of XP_020598766.1
			PAXXG244240	uncharacterized protein, homologue of XP_020596058.1
			PAXXG244310	conserved protein of unknown function, supported by RNASeq data
			PAXXG244350	RING-H2 finger protein ATL74-like
			PAXXG244360	conserved hypothetical protein homologue of PKU67230.1
			PAXXG104080	uncharacterized protein, homologue of XP_020599770.1
			PAXXG104090	uncharacterized protein, homologue of XP_020599770.1
			PAXXG104100	uncharacterized protein, homologue of XP_020597338.1
			PAXXG104110	actin
			PAXXG104130	uncharacterized protein, homologue of XP_020597339.1
			PAXXG104140	conserved protein of unknown function, supported by RNASeq data
			PAXXG104150	conserved protein of unknown function, supported by RNASeq data
			PAXXG104160	uncharacterized protein, homologue of XP_020597340.1
			PAXXG104170	uncharacterized protein, homologue of XP_020586142.1
			PAXXG104180	Similar to retrovirus-related Pol polyprotein from transposon TNT 1-94
			PAXXG104190	uncharacterized protein, homologue of XP_020597003.1
			PAXXG104200	MYB transcription factor
			PAXXG104240	zinc finger protein VAR3, chloroplastic
			PAXXG104250	60S ribosomal protein L24
			PAXXG104260	protein SLOW WALKER 1
			PAXXG104270	RNA demethylase ALKBH5-like
			PAXXG104280	conserved protein of unknown function, supported by RNASeq data
			PAXXG104290	uncharacterized protein, homologue of XP_020599583.1
			PAXXG104300	ethanolamine-phosphate cytidylyltransferase-like isoform X2
			PAXXG104305	bifunctional nitrilase/nitrile hydratase NIT4-like
			PAXXG104310	unknown protein 1-like
			PAXXG104330	Transporter, the Auxin Efflux Carrier (AEC) Family
			PAXXG104340	RING finger and transmembrane domain-containing protein 2-like
			PAXXG104350	uncharacterized protein, homologue of XP_020577803.1
			PAXXG104360	FAR1 transcription factor
			PAXXG104370	uncharacterized protein, homologue of XP_020589883.1
			PAXXG104380	sufE-like protein 2, chloroplastic
			PAXXG104390	probable LRR receptor-like serine/threonine-protein kinase At1g06840 isoform X1
			PAXXG104400	MADS box protein
			PAXXG104410	serotonin N-acetyltransferase 1, chloroplastic
			PAXXG104430	sentrin-specific protease 2-like isoform X2
			PAXXG104440	uncharacterized protein, homologue of XP_020595915.1
			PAXXG104450	uncharacterized protein, homologue of XP_020598113.1
			PAXXG104470	probable serine/threonine-protein kinase roco5
			PAXXG104480	uncharacterized protein At5g19025-like
			PAXXG104510	E3 ubiquitin-protein ligase CIP8-like
			PAXXG104520	conserved hypothetical protein homologue of PKU67230.1
			PAXXG104530	protein DGS1, mitochondrial isoform X1
			PAXXG104540	uncharacterized protein, homologue of XP_020581952.1
			PAXXG104550	uncharacterized protein, homologue of XP_020581952.1
			PAXXG104570	zf-HD transcription factor

			PAXXG104580	Hypothetical protein, supported by RNA-Seq data
			PAXXG104590	cysteine synthase-like
			PAXXG104610	uncharacterized protein, homologue of XP_020588264.1
			PAXXG104620	non-specific lipid-transfer protein 1-like
			PAXXG104640	DEAD-box ATP-dependent RNA helicase 10-like
			PAXXG304810	uncharacterized protein, homologue of XP_020595835.1
			PAXXG304820	rab GTPase-activating protein 1 isoform X1
19	L19	7006569~9312937	PAXXG330200	Pre-mRNA-processing protein 40C
			PAXXG204430	uncharacterized protein, homologue of XP_020595829.1
			PAXXG204440	uncharacterized protein, homologue of XP_020581221.1
			PAXXG204470	uncharacterized protein, homologue of XP_020595829.1
			PAXXG204510	uncharacterized protein, homologue of XP_020597903.1
			PAXXG204580	uncharacterized protein, homologue of XP_020595835.1
			PAXXG204590	uncharacterized protein, homologue of XP_020584790.1
			PAXXG204610	uncharacterized protein, homologue of XP_020599223.1
			PAXXG204620	conserved hypothetical protein homologue of PKU67230.1
			PAXXG204660	RNA demethylase ALKBH5-like
			PAXXG204670	RNA demethylase ALKBH5-like
			PAXXG204690	uncharacterized protein, homologue of XP_020674603.1
			PAXXG204700	uncharacterized protein, homologue of XP_020593563.1
			PAXXG204720	hypothetical protein, weak similar to PKU67230.1
			PAXXG204750	phytosulfokine receptor 1-like
			PAXXG204770	conserved hypothetical protein homologue of PKA50312.1
			PAXXG204790	aldo-keto reductase family 4 member C10-like
			PAXXG204800	conserved hypothetical protein homologue of PKU67230.1
			PAXXG204870	uncharacterized protein, homologue of XP_020596058.1
			PAXXG204880	RNA demethylase ALKBH5-like
			PAXXG204890	conserved protein of unknown function, supported by RNASeq data
			PAXXG204910	cysteine-rich receptor-like protein kinase 2 isoform X1
			PAXXG204920	uncharacterized protein, homologue of XP_020596374.1
			PAXXG204930	uncharacterized protein, homologue of XP_020595835.1
			PAXXG204940	uncharacterized protein, homologue of XP_020594067.1
			PAXXG204950	uncharacterized protein, homologue of XP_020584790.1
			PAXXG204970	hypothetical protein, weak similar to PKU67230.1
			PAXXG246710	uncharacterized protein, homologue of XP_020582885.1
			PAXXG246730	conserved protein of unknown function, supported by RNASeq data
			PAXXG246740	uncharacterized protein At4g15970-like
			PAXXG246750	uncharacterized protein, homologue of XP_020585184.1
			PAXXG246760	pentatricopeptide repeat-containing protein At5g04810, chloroplastic
			PAXXG246775	syntaxin-52-like
			PAXXG246840	uncharacterized protein, homologue of XP_020599150.1
			PAXXG246860	serine/threonine-protein kinase/endoribonuclease IRE1a-like
			PAXXG246890	uncharacterized protein, homologue of XP_020596058.1
			PAXXG246900	uncharacterized protein, homologue of XP_020575046.1
			PAXXG246920	protein NETWORKED 1A-like
			PAXXG246950	RNA demethylase ALKBH5-like
			PAXXG246960	Unknown protein, supported by RNA-Seq data
			PAXXG269100	DNA polymerase delta small subunit isoform X1
			PAXXG269140	uncharacterized protein, homologue of XP_020596374.1
			PAXXG269170	conserved protein of unknown function, supported by RNASeq data
			PAXXG269180	pentatricopeptide repeat-containing protein At3g54980, mitochondrial-like
10	L10b	11281837~13491100	PAXXG160790	secretory carrier-associated membrane protein 2-like
			PAXXG160800	ataxia telangiectasia mutated family protein
			PAXXG160810	TPR repeat-containing protein ZIP4 isoform X1
			PAXXG160820	beta-hexosaminidase 3-like isoform X2
			PAXXG160860	ribonuclease TUDOR 1-like
			PAXXG160870	enoyl-CoA delta isomerase 2, peroxisomal-like
			PAXXG160880	enoyl-CoA delta isomerase 2, peroxisomal-like
			PAXXG160900	FAR1 transcription factor
			PAXXG160910	protein SYM1-like
			PAXXG160920	putative cell wall protein
			PAXXG160930	FAR1 transcription factor
			PAXXG160980	RNA demethylase ALKBH5-like
			PAXXG160990	putative cell wall protein
			PAXXG161000	uncharacterized protein, homologue of XP_020598276.1
			PAXXG161030	conserved hypothetical protein homologue of PKU67230.1
			PAXXG161040	conserved protein of unknown function, supported by RNASeq data
			PAXXG161050	uncharacterized protein, homologue of XP_020595915.1
			PAXXG161090	nuclear poly(A) polymerase 1
			PAXXG161110	uncharacterized protein, homologue of XP_020595835.1
			PAXXG161120	conserved hypothetical protein homologue of PKU67230.1
			PAXXG161180	small G protein signaling modulator 1-like isoform X2
			PAXXG161190	uncharacterized protein, homologue of XP_020576865.1
			PAXXG161210	uncharacterized protein, homologue of XP_020599700.1
			PAXXG161230	uncharacterized protein, homologue of XP_020595955.1
			PAXXG161260	uncharacterized protein, homologue of XP_020593563.1
			PAXXG161270	conserved hypothetical protein homologue of PKU67230.1
			PAXXG161280	RNA demethylase ALKBH5-like
			PAXXG161290	conserved hypothetical protein homologue of PKU67230.1
			PAXXG161300	conserved protein of unknown function, supported by RNASeq data
			PAXXG161320	cyclin-B1-2-like
			PAXXG161340	RNA demethylase ALKBH5-like
			PAXXG161380	uncharacterized protein, homologue of XP_020598499.1
			PAXXG161390	RNA demethylase ALKBH5-like
			PAXXG284960	conserved protein of unknown function, supported by RNASeq data
			PAXXG284970	T-complex protein 1 subunit eta
			PAXXG284980	polygalacturonase-like
			PAXXG285000	uncharacterized protein, homologue of XP_020599372.1
			PAXXG285020	uncharacterized protein, homologue of XP_020596869.1
			PAXXG285040	inosine triphosphate pyrophosphatase
			PAXXG285050	serine/threonine-protein kinase ATM
			PAXXG288350	isoleucine--tRNA ligase, cytoplasmic

			PAXXG288355	uncharacterized protein, homologue of XP_020702838.1
			PAXXG288360	FAR1 transcription factor
			PAXXG288370	phytohormone-binding protein-like
			PAXXG288380	uncharacterized protein, homologue of XP_020571293.1
			PAXXG288390	putative pentatricopeptide repeat-containing protein At3g01580
			PAXXG288410	serine/threonine-protein kinase ATM
10	L10a	9160567~11360027	PAXXG344920	protein EMSY-LIKE 3-like
			PAXXG150080	RNA demethylase ALKBH5-like
			PAXXG150100	conserved protein of unknown function, supported by RNASeq data
			PAXXG150120	RNA demethylase ALKBH5-like
			PAXXG150130	conserved protein of unknown function, supported by RNASeq data
			PAXXG150160	conserved protein of unknown function, supported by RNASeq data
			PAXXG150190	MORC family CW-type zinc finger protein 2-like isoform X2
			PAXXG150200	uncharacterized protein, homologue of XP_020599640.1
			PAXXG150210	L10-interacting MYB domain-containing protein-like
			PAXXG150220	MORC family CW-type zinc finger protein 2-like isoform X2
			PAXXG150240	conserved protein of unknown function, supported by RNASeq data
			PAXXG150250	cellulose synthase-like protein D4
			PAXXG150260	Unknown protein, supported by RNA-Seq data
			PAXXG150270	hypothetical protein, weak similar to PKU67230.1
			PAXXG150280	RNA demethylase ALKBH5-like
			PAXXG150290	conserved protein of unknown function, supported by RNASeq data
			PAXXG150300	uncharacterized protein, homologue of XP_020599657.1
			PAXXG150320	bZIP transcription factor
			PAXXG150350	RNA-binding protein 24-like isoform X1
			PAXXG150360	large proline-rich protein BAG6 isoform X4
			PAXXG150380	kinesin-like protein KIN-14I isoform X1
			PAXXG150435	L10-interacting MYB domain-containing protein-like
			PAXXG150440	conserved protein of unknown function, supported by RNASeq data
			PAXXG150450	wall-associated receptor kinase 5-like
			PAXXG150460	wall-associated receptor kinase 5-like
			PAXXG150480	cystathionine beta-lyase, chloroplastic
			PAXXG150490	RNA demethylase ALKBH5-like
			PAXXG150510	uncharacterized protein, homologue of XP_020595536.1
			PAXXG267920	uncharacterized protein, homologue of XP_020591803.1
			PAXXG267930	3,9-dihydroxypterocarpan 6A-monooxygenase-like
			PAXXG267940	putative mitochondrial protein
			PAXXG267950	uncharacterized protein, homologue of XP_020597397.1
			PAXXG267960	uncharacterized protein, homologue of XP_020585569.1
			PAXXG267970	conserved protein of unknown function, supported by RNASeq data
			PAXXG267980	conserved protein of unknown function, supported by RNASeq data
			PAXXG267990	conserved protein of unknown function, supported by RNASeq data
			PAXXG268000	TBCC domain-containing protein 1-like
			PAXXG268010	uncharacterized protein, homologue of XP_020692040.1
			PAXXG268020	DEAD-box ATP-dependent RNA helicase 10-like
			PAXXG268050	RNA demethylase ALKBH5-like
			PAXXG268060	rhodanese-like domain-containing protein 9, chloroplastic
			PAXXG268070	proteasome subunit alpha type-2-B
			PAXXG268075	uncharacterized protein, homologue of XP_020690392.1
3	L03	16133241~18154789	PAXXG289050	RNA demethylase ALKBH5-like
			PAXXG289060	threonine--tRNA ligase, chloroplastic/mitochondrial 2-like
			PAXXG289080	conserved protein of unknown function, supported by RNASeq data
			PAXXG289090	Transporter, the Mitochondrial Carrier (MC) Family
			PAXXG289100	autophagy-related protein 13a
			PAXXG289110	uncharacterized protein, homologue of XP_020599770.1
			PAXXG289120	conserved hypothetical protein homologue of PKU82262.1
			PAXXG210020	TUB transcription factor
			PAXXG210050	uncharacterized protein, homologue of XP_020586142.1
			PAXXG210060	subtilisin-like protease SBT3.12
			PAXXG210070	subtilisin-like protease SBT3.4
			PAXXG210080	L10-interacting MYB domain-containing protein-like
			PAXXG210100	Transporter, the ATP-binding Cassette (ABC) Superfamily
			PAXXG210120	conserved hypothetical protein homologue of PKU67230.1
			PAXXG210130	uncharacterized protein, homologue of XP_020598317.1
			PAXXG210180	uncharacterized protein, homologue of XP_020598403.1
			PAXXG210200	ubinuclein-1-like
			PAXXG210210	uncharacterized protein, homologue of XP_020597516.1
			PAXXG210220	ataxia telangiectasia mutated family protein
			PAXXG210230	uncharacterized protein, homologue of XP_020599640.1
			PAXXG218750	IRK-interacting protein-like
			PAXXG218760	hypothetical chloroplast RF2
			PAXXG218770	Ycf2
			PAXXG218820	uncharacterized protein, homologue of XP_020599868.1
			PAXXG218830	uncharacterized protein, homologue of XP_020599770.1
			PAXXG218840	2-oxoglutarate (2OG) and Fe(II)-dependent oxygenase superfamily protein
			PAXXG218850	pentatricopeptide repeat-containing protein At1g09220, mitochondrial-like
			PAXXG218870	uncharacterized protein, homologue of XP_020598333.1
			PAXXG218880	uncharacterized protein, homologue of XP_020597103.1
			PAXXG218900	RNA demethylase ALKBH5-like
			PAXXG218910	conserved protein of unknown function, supported by RNASeq data
			PAXXG218930	60S ribosomal protein L31
			PAXXG218940	uncharacterized protein, homologue of XP_020588637.1
			PAXXG218970	uncharacterized protein, homologue of XP_020594067.1
			PAXXG218990	polygalacturonase ADPG2
2	L02	27969064~29948067	PAXXG300370	uncharacterized protein, homologue of XP_020595477.1
			PAXXG300400	ribosomal RNA processing protein 36 homolog isoform X2
			PAXXG146450	AP2/ERF-ERF transcription factor
			PAXXG146460	N-alpha-acetyltransferase MAK3
			PAXXG146470	protein BONZAI 1-like
			PAXXG146480	uncharacterized protein, homologue of XP_020581952.1
			PAXXG146490	Trihelix transcription factor
			PAXXG146500	stomatin-like protein 2, mitochondrial isoform X1

			PAXXG146510	uncharacterized protein, homologue of XP_020591393.1
			PAXXG146520	cytochrome c-type biogenesis CcmH-like mitochondrial protein
			PAXXG146530	ataxia telangiectasia mutated family protein
			PAXXG146540	obtusifoliol 14-alpha demethylase
			PAXXG146550	light-regulated protein
			PAXXG146560	RNA-directed DNA polymerase like
			PAXXG146570	B3-ARF transcription factor
			PAXXG146600	Histone H4
			PAXXG146610	Histone H4 (ISS)-like
			PAXXG146620	serine carboxypeptidase-like 45
			PAXXG146650	uncharacterized protein, homologue of XP_020598766.1
			PAXXG146660	RNA demethylase ALKBH5-like
			PAXXG146670	actin-depolymerizing factor 11-like
			PAXXG146680	RNA demethylase ALKBH5-like
			PAXXG146690	uncharacterized protein, homologue of XP_020599731.1
			PAXXG146700	bHLH transcription factor
			PAXXG146710	GEM-like protein 5
			PAXXG146720	uncharacterized protein, homologue of XP_020592319.1
			PAXXG146730	translocon-associated protein subunit beta
			PAXXG146740	uncharacterized protein, homologue of XP_020592321.1
			PAXXG146750	probable inactive shikimate kinase like 1, chloroplastic
			PAXXG146760	protein VAC14 homolog
			PAXXG146770	probable U3 small nucleolar RNA-associated protein 7
			PAXXG146780	uncharacterized protein, homologue of XP_020591677.1
			PAXXG146790	NAC transcription factor
			PAXXG146800	double-stranded RNA-binding protein 3-like
			PAXXG146810	protein OPAQUE1-like
			PAXXG146830	cell division cycle 20.2, cofactor of APC complex-like
			PAXXG146860	RNA demethylase ALKBH5-like
			PAXXG146870	probable LRR receptor-like serine/threonine-protein kinase At2g24230 isoform X1
			PAXXG146900	uncharacterized protein, homologue of XP_020593563.1
			PAXXG146910	putative ribosome biogenesis protein slx9-like
			PAXXG146920	cytochrome b561, DM13 and DOMON domain-containing protein At5g54830
			PAXXG146930	uncharacterized protein, homologue of XP_020678670.1
10	L10a	17949901~19917738	PAXXG244500	negative regulator of systemic acquired resistance SNI1 isoform X1
			PAXXG244510	pentatricopeptide repeat-containing protein At1g55890, mitochondrial-like
			PAXXG244530	uncharacterized protein, homologue of XP_020599657.1
			PAXXG244540	Unknown protein, supported by RNA-Seq data, weak similar to PKU67230.1
			PAXXG244560	uncharacterized protein, homologue of XP_020598333.1
			PAXXG244570	conserved hypothetical protein homologue of PKU67230.1
			PAXXG244580	rac-like GTP-binding protein 7
			PAXXG244610	uncharacterized protein, homologue of XP_020671729.1
			PAXXG244640	uncharacterized protein, homologue of XP_020595829.1
			PAXXG244660	uncharacterized protein, homologue of XP_020596072.1
			PAXXG257090	uncharacterized protein, homologue of XP_020581370.1
			PAXXG257130	hypothetical protein, weak similar to XP_020588264.1
			PAXXG257140	MLO-like protein 2
			PAXXG257160	uncharacterized protein, homologue of XP_020599372.1
			PAXXG257170	uncharacterized protein, homologue of XP_020593411.1
			PAXXG257190	uncharacterized protein, homologue of XP_020598403.1
			PAXXG257230	uncharacterized protein, homologue of XP_020599770.1
			PAXXG257250	B3-ARF transcription factor
			PAXXG257350	uncharacterized protein, homologue of XP_020581952.1
			PAXXG257360	RNA demethylase ALKBH5-like
			PAXXG257370	uncharacterized protein, homologue of XP_020598317.1
			PAXXG281390	NAC transcription factor
			PAXXG281400	RNA demethylase ALKBH5-like
			PAXXG281420	conserved hypothetical protein homologue of PKU62795.1
			PAXXG281430	Unknown protein, supported by RNA-Seq data, weak similar to XP_020588264.1
			PAXXG281470	conserved protein of unknown function, supported by RNASeq data
			PAXXG281480	uncharacterized protein, homologue of XP_020573760.1
			PAXXG281490	NAC transcription factor
			PAXXG281500	Unknown protein, supported by RNA-Seq data, weak similar to XP_020599657.1
			PAXXG281550	uncharacterized protein, homologue of XP_020596058.1
			PAXXG281560	conserved hypothetical protein homologue of PKU67230.1
			PAXXG281570	conserved hypothetical protein homologue of PKU67230.1
			PAXXG281590	conserved hypothetical protein homologue of PKU82912.1
			PAXXG281600	RNA demethylase ALKBH5-like
			PAXXG281620	RNA demethylase ALKBH5-like
			PAXXG281630	RNA demethylase ALKBH5-like
			PAXXG281660	uncharacterized protein, homologue of XP_020599770.1
			PAXXG325360	homogentisate 1,2-dioxygenase
9	L09	14378046~15950517	PAXXG216200	uncharacterized protein, homologue of XP_020596168.1
			PAXXG216230	uncharacterized protein, homologue of XP_020595835.1
			PAXXG216240	uncharacterized protein, homologue of XP_020593563.1
			PAXXG216270	uncharacterized protein, homologue of XP_020593896.1
			PAXXG216300	uncharacterized protein, homologue of XP_020593563.1
			PAXXG216310	uncharacterized protein, homologue of XP_020598317.1
			PAXXG216350	hypothetical protein, weak similar to XP_020588264.1
			PAXXG216370	calcium-dependent protein kinase 18-like
			PAXXG216380	uncharacterized protein, homologue of XP_020581952.1
			PAXXG216390	L10-interacting MYB domain-containing protein-like
			PAXXG216400	protein CDI-like
			PAXXG216420	conserved hypothetical protein homologue of PKU84750.1
			PAXXG216450	conserved protein of unknown function, supported by RNASeq data
			PAXXG216460	uncharacterized protein, homologue of XP_020598674.1
			PAXXG216470	uncharacterized protein, homologue of XP_020592067.1
			PAXXG216480	uncharacterized protein, homologue of XP_020598333.1
			PAXXG216520	RNA demethylase ALKBH5-like
			PAXXG216570	uncharacterized protein At2g29880-like
			PAXXG269710	probable serine acetyltransferase 2 isoform X1
			PAXXG269730	phosphoinositide phosphatase SAC6-like isoform X1

			PAXXG269740	40S ribosomal protein S15a
			PAXXG269750	cancer-related nucleoside-triphosphatase homolog isoform X1
			PAXXG269760	hypothetical protein, weak similar to XP_020599657.1
			PAXXG269780	uncharacterized protein, homologue of XP_020595915.1
			PAXXG269790	protein EXORDIUM-like 5
			PAXXG269800	Unknown protein, supported by RNA-Seq data
			PAXXG331770	uncharacterized protein, homologue of XP_020576144.1
			PAXXG331780	uncharacterized protein, homologue of XP_020575046.1
			PAXXG331790	peflin-like isoform X1
			PAXXG331800	uncharacterized protein, homologue of XP_020595915.1
			PAXXG331815	uncharacterized protein, homologue of XP_020582826.1
16	L16	130295~1702364	PAXXG349090	endoglucanase 24-like
			PAXXG196170	uncharacterized protein, homologue of XP_020598333.1
			PAXXG196180	uncharacterized protein, homologue of XP_020570899.1
			PAXXG196190	uncharacterized protein, homologue of XP_020593563.1
			PAXXG196200	5'-3' exoribonuclease 3 isoform X2
			PAXXG196220	hypothetical protein, weak similar to XP_020588264.1
			PAXXG196240	uncharacterized protein, homologue of XP_020576125.1
			PAXXG196250	uncharacterized RNA-binding protein C17H9.04c
			PAXXG196260	conserved protein of unknown function, supported by RNASeq data
			PAXXG196270	expansin-B16-like
			PAXXG196280	uncharacterized protein, homologue of XP_020599657.1
			PAXXG196290	L-lactate dehydrogenase A-like
			PAXXG196310	40S ribosomal protein S15a-5-like isoform X3
			PAXXG196320	Putative cytochrome c biogenesis ccmF-like mitochondrial protein
			PAXXG196350	TBC1 domain family member 15-like
			PAXXG196360	putative nuclease HARBI1
			PAXXG196370	L10-interacting MYB domain-containing protein-like
			PAXXG196380	ubiquitin-like-conjugating enzyme ATG10 isoform X1
			PAXXG196390	Unknown protein, supported by RNA-Seq data, weak similar to XP_020588264.1
			PAXXG196400	RNA demethylase ALKBH5-like
			PAXXG196440	uncharacterized protein, homologue of XP_020585184.1
			PAXXG196450	protease Do-like 10, mitochondrial isoform X2
			PAXXG196460	protease Do-like 10, mitochondrial isoform X1
			PAXXG196470	RNA demethylase ALKBH5-like
			PAXXG196480	LOW QUALITY PROTEIN: trafficking protein particle complex II-specific subunit 120 homolog
			PAXXG196490	uncharacterized protein, homologue of XP_020596815.1
			PAXXG196500	uncharacterized protein, homologue of XP_020595710.1
			PAXXG227810	E3 ubiquitin ligase BIG BROTHER-related-like
			PAXXG227830	protein N-lysine methyltransferase METTL21A isoform X2
			PAXXG227840	protein FAR1-RELATED SEQUENCE 5-like
			PAXXG227850	serine/threonine-protein kinase 19 isoform X1
			PAXXG227860	putative nuclease HARBI1
			PAXXG227870	protein FAR1-RELATED SEQUENCE 5-like
			PAXXG227910	conserved hypothetical protein homologue of PKU82262.1
			PAXXG227920	RNA demethylase ALKBH5-like
			PAXXG227940	NADH dehydrogenase
			PAXXG227950	separase-like
			PAXXG227960	eukaryotic translation initiation factor 5A-4-like
			PAXXG227965	glutathione gamma-glutamylcysteinyltransferase 1-like
16	L16	2422375~3990997	PAXXG166970	thioredoxin F1, chloroplastic-like
			PAXXG167000	uncharacterized protein, homologue of XP_020596848.1
			PAXXG167040	Fimbrin-like protein 2
			PAXXG167060	conserved hypothetical protein homologue of PKU67230.1
			PAXXG167070	uncharacterized protein, homologue of XP_020598317.1
			PAXXG167080	uncharacterized protein, homologue of XP_020593563.1
			PAXXG167100	uncharacterized protein, homologue of XP_020587366.1
			PAXXG167120	gibberellin 2-beta-dioxygenase 2-like
			PAXXG167125	conserved hypothetical protein homologue of PKA48168.1
			PAXXG167130	neutral/alkaline invertase 1, mitochondrial-like
			PAXXG167140	conserved protein of unknown function, supported by RNASeq data
			PAXXG167150	NF-YB transcription factor
			PAXXG167160	conserved hypothetical protein homologue of PKA47403.1
			PAXXG167170	sister chromatid cohesion protein PDS5 homolog A
			PAXXG167180	conserved protein of unknown function, supported by RNASeq data
			PAXXG167190	arginine/serine-rich coiled-coil protein 2 isoform X2
			PAXXG167200	protein MIZU-KUSSEI 1-like
			PAXXG167210	uncharacterized protein, homologue of XP_020598042.1
			PAXXG167220	FAR1 transcription factor
			PAXXG167230	10 kDa chaperonin, mitochondrial
			PAXXG167240	protein O-linked-mannose beta-1,4-N-acetylglucosaminyltransferase 2-like
			PAXXG167250	serine/threonine-protein kinase prpf4B isoform X1
			PAXXG167260	ataxia telangiectasia mutated family protein
			PAXXG167270	calmodulin-2/4-like
			PAXXG167280	uncharacterized protein, homologue of XP_020587486.1
			PAXXG167290	UBP1-associated protein 2A-like
			PAXXG167300	RNA demethylase ALKBH5-like
			PAXXG167310	DNA gyrase subunit A, chloroplastic/mitochondrial isoform X2
			PAXXG167320	uncharacterized protein, homologue of XP_020599770.1
			PAXXG235210	RNA demethylase ALKBH5-like
			PAXXG235230	RNA demethylase ALKBH5-like
			PAXXG235250	Transporter, the Mitochondrial Protein Translocase (MPT) Family
			PAXXG235260	uncharacterized protein, homologue of XP_020592205.1
			PAXXG235270	transcription termination factor MTERF4, chloroplastic
			PAXXG235280	methyl-CpG-binding domain protein 4-like protein
			PAXXG235310	uncharacterized protein, homologue of XP_020598317.1
10	L10a	7269810~8816154	PAXXG188780	uncharacterized protein, homologue of XP_020673836.1
			PAXXG188790	65-kDa microtubule-associated protein 1-like
			PAXXG188800	conserved protein of unknown function, supported by RNASeq data
			PAXXG188820	adenylate kinase 5, chloroplastic-like isoform X2
			PAXXG188830	uncharacterized protein, homologue of XP_020588922.1
			PAXXG188840	probable receptor-like protein kinase At1g80640 isoform X1

			PAXXG188850	Transporter, the Monovalent Cation:Proton Antiporter-1 (CPA1) Family
			PAXXG188860	uncharacterized protein, homologue of XP_020599770.1
			PAXXG188870	putative 4-hydroxy-4-methyl-2-oxoglutarate aldolase 3
			PAXXG188880	ultraviolet-B receptor UVR8
			PAXXG188890	uncharacterized protein, homologue of XP_020593905.1
			PAXXG188910	putative membrane-bound O-acyltransferase C24H6.01c
			PAXXG188925	uncharacterized protein, homologue of XP_020571831.1
			PAXXG188930	uncharacterized protein, homologue of XP_020673723.1
			PAXXG188940	RNA-directed DNA polymerase
			PAXXG188950	ubiquitin-conjugating enzyme E2 5A-like
			PAXXG188980	uncharacterized protein, homologue of XP_020599657.1
			PAXXG188990	Unknown protein, supported by RNA-Seq data, weak similar to PKU67230.1
			PAXXG189000	FAR1 transcription factor
			PAXXG189010	actin-related protein 8 isoform X1
			PAXXG189020	Unknown protein, supported by RNA-Seq data
			PAXXG189040	L10-interacting MYB domain-containing protein-like
			PAXXG189050	GARP-G2-like transcription factor
			PAXXG189060	FAR1 transcription factor
			PAXXG189070	uncharacterized protein, homologue of XP_020576607.1
			PAXXG189100	uncharacterized protein, homologue of XP_020596058.1
			PAXXG189110	NF-YC transcription factor
			PAXXG189120	Unknown protein, supported by RNA-Seq data
			PAXXG189130	exosome complex component RRP45A-like
			PAXXG189140	conserved protein of unknown function, supported by RNASeq data
			PAXXG189150	glucan endo-1,3-beta-glucosidase 5
			PAXXG189160	E3 ubiquitin-protein ligase RGLG5-like
			PAXXG189170	UPF0481 protein At3g47200-like isoform X1
			PAXXG189180	uncharacterized protein, homologue of XP_020572696.1
			PAXXG189190	UPF0481 protein At3g47200-like isoform X1
			PAXXG189200	uncharacterized protein, homologue of XP_020592067.1
			PAXXG278520	uncharacterized protein, homologue of XP_020571363.1
			PAXXG278530	3,9-dihydroxypterocarpan 6A-monooxygenase-like
			PAXXG278540	26S proteasome non-ATPase regulatory subunit 4 homolog
			PAXXG278550	RNA demethylase ALKBH5-like
			PAXXG278560	RNA demethylase ALKBH5-like
			PAXXG278570	cytochrome P450 93A2-like
			PAXXG278580	DNA repair helicase XPD
			PAXXG278590	uncharacterized protein, homologue of XP_020599840.1
			PAXXG278600	protein FLUORESCENT IN BLUE LIGHT, chloroplastic-like
17	L17	11049261~12549030	PAXXG203840	putative deoxyribonuclease TATDN1
			PAXXG203843	uncharacterized protein, homologue of XP_020597781.1
			PAXXG203847	zinc finger MYM-type protein 1-like
			PAXXG203850	uncharacterized protein, homologue of XP_020582885.1
			PAXXG203860	cis epoxycarotenoid dioxygenase
			PAXXG203870	uncharacterized protein, homologue of XP_020595831.1
			PAXXG203880	conserved hypothetical protein homologue of PKA48168.1
			PAXXG203920	conserved protein of unknown function, supported by RNASeq data
			PAXXG203930	conserved protein of unknown function, supported by RNASeq data
			PAXXG203950	translation initiation factor eIF-2B subunit delta-like isoform X1
			PAXXG203990	uncharacterized protein, homologue of XP_020592067.1
			PAXXG204020	zinc finger protein KNUCKLES-like
			PAXXG204030	uncharacterized protein, homologue of XP_020592067.1
			PAXXG204040	conserved hypothetical protein homologue of PKU82262.1
			PAXXG293140	conserved hypothetical protein homologue of PKU67230.1
			PAXXG293150	nitric oxide synthase-interacting protein
			PAXXG293160	NADH dehydrogenase
			PAXXG293180	putative phosphatidylglycerol/phosphatidylinositol transfer protein DDB_G0282179
			PAXXG293195	NADPH-dependent alkenal/one oxidoreductase, chloroplastic
			PAXXG293200	protein IQ-DOMAIN 1 isoform X3
			PAXXG293210	protoheme IX farnesyltransferase, mitochondrial-like
			PAXXG293220	syntaxin-121-like
			PAXXG293240	putative deoxyribonuclease TATDN1
			PAXXG108460	conserved protein of unknown function, supported by RNASeq data
			PAXXG108470	uncharacterized protein, homologue of XP_020598548.1
6	L06	13907976~15378833	PAXXG183720	Transporter, the Major Facilitator Superfamily (MFS)
			PAXXG183770	uncharacterized protein, homologue of XP_020593563.1
			PAXXG183780	zf-HD transcription factor
			PAXXG183790	uncharacterized protein, homologue of XP_020595835.1
			PAXXG183820	conserved hypothetical protein homologue of PKU67230.1
			PAXXG183830	RNA demethylase ALKBH5-like
			PAXXG183880	uncharacterized protein, homologue of XP_020597793.1
			PAXXG183890	uncharacterized protein, homologue of XP_020593563.1
			PAXXG183900	MYB-related transcription factor
			PAXXG183910	uncharacterized protein, homologue of XP_020593563.1
			PAXXG183960	uncharacterized protein, homologue of XP_020598497.1
			PAXXG183990	uncharacterized protein, homologue of XP_020592067.1
			PAXXG184060	cytochrome P450 71A1-like
			PAXXG184080	conserved protein of unknown function, supported by RNASeq data
			PAXXG184090	uncharacterized protein, homologue of XP_020597626.1
			PAXXG184100	RNA demethylase ALKBH5-like
			PAXXG184110	BTB/POZ domain and ankyrin repeat-containing protein NPR5-like
			PAXXG184120	cytochrome P450 71A1-like
			PAXXG184130	heat shock 70 kDa protein 14-like
			PAXXG184140	cytochrome P450 71A1-like
			PAXXG184160	conserved hypothetical protein homologue of PKU67230.1
			PAXXG184170	uncharacterized protein, homologue of XP_020599073.1
			PAXXG184180	uncharacterized protein, homologue of XP_020598227.1
			PAXXG184190	uncharacterized protein, homologue of XP_020599770.1
			PAXXG184200	uncharacterized protein, homologue of XP_020599770.1
			PAXXG184210	uncharacterized protein, homologue of XP_020595915.1
			PAXXG184220	RNA demethylase ALKBH5-like
			PAXXG184240	uncharacterized protein, homologue of XP_020599657.1

			PAXXG293270	uncharacterized protein, homologue of XP_020680661.1
			PAXXG293280	RNA demethylase ALKBH5-like
			PAXXG293290	protein FAR1-RELATED SEQUENCE 1-like
			PAXXG293300	carbonic anhydrase 2-like isoform X1
			PAXXG293310	probable acyl-
6	L06	16947208~18382461	PAXXG165170	nicastrin
			PAXXG165200	tRNA threonylcarbamoyladenosine dehydratase isoform X4
			PAXXG165240	VQ motif-containing protein 4-like
			PAXXG165250	uncharacterized protein, homologue of XP_020598317.1
			PAXXG165260	RNA demethylase ALKBH5-like
			PAXXG165270	mediator of RNA polymerase II transcription subunit 9-like
			PAXXG165290	Transporter, the H+- or Na+-translocating F-type, V-type and A-type ATPase (F-ATPase) Superfamily
			PAXXG165300	Transporter, the Voltage-gated Ion Channel (VIC) Superfamily
			PAXXG165310	uncharacterized protein, homologue of XP_020595915.1
			PAXXG165320	probable histidine kinase 6
			PAXXG165330	protein MODIFIER OF SNC1 1 isoform X1
			PAXXG165340	probable sodium/metabolite cotransporter BASS3, chloroplastic
			PAXXG165360	protein FAR1-RELATED SEQUENCE 1-like
			PAXXG165370	putative methyltransferase NSUN6 isoform X1
			PAXXG165380	zinc finger BED domain-containing protein RICESLEEPER 3-like
			PAXXG165390	conserved hypothetical protein homologue of PKU62491.1
			PAXXG165400	3-deoxy-manno-octulosonate cytidylyltransferase, mitochondrial isoform X1
			PAXXG165410	TGF-beta-activated kinase 1 and MAP3K7-binding protein 3-like
			PAXXG165430	non-canonical poly(A) RNA polymerase PAPD5
			PAXXG165440	aspartic proteinase CDR1-like
			PAXXG165450	protein NUCLEAR FUSION DEFECTIVE 6, chloroplastic/mitochondrial-like isoform X2
			PAXXG165470	uncharacterized protein, homologue of XP_020595397.1
			PAXXG165485	putative pentatricopeptide repeat-containing protein At3g23330
			PAXXG165490	phospho-N-acetylmuramoyl-pentapeptide-transferase homolog
			PAXXG165500	uncharacterized protein, homologue of XP_020596058.1
			PAXXG165520	cysteine-rich receptor-like protein kinase 2
			PAXXG165530	Transporter, the Voltage-gated Ion Channel (VIC) Superfamily
			PAXXG268600	dihydropyrimidine dehydrogenase (NADP(+)), chloroplastic
17	L17	7762538~9191927	PAXXG191760	microtubule-associated protein TORTIFOLIA1-like
			PAXXG191770	cysteine-rich receptor-like protein kinase 2
			PAXXG191780	uncharacterized protein, homologue of XP_020590252.1
			PAXXG191790	protein FATTY ACID EXPORT 5-like isoform X1
			PAXXG191800	orf192 (mitochondrion)
			PAXXG191830	expansin-B16-like
			PAXXG191850	RNA demethylase ALKBH5-like
			PAXXG191860	alpha carbonic anhydrase 7-like
			PAXXG191870	bifunctional monodehydroascorbate reductase and carbonic anhydrase nectarin-3-like
			PAXXG191880	uncharacterized protein, homologue of XP_020593522.1
			PAXXG191890	abnormal spindle-like microcephaly-associated protein homolog
			PAXXG191900	uncharacterized transmembrane protein DDB_G0289901-like
			PAXXG191910	condensin-2 complex subunit H2 isoform X1
			PAXXG191920	uncharacterized protein, homologue of XP_020596236.1
			PAXXG191930	zinc finger BED domain-containing protein RICESLEEPER 3-like
			PAXXG191940	DEAD-box ATP-dependent RNA helicase 32
			PAXXG191950	Transporter, the Multidrug/Oligosaccharidyl-lipid/Polysaccharide (MOP) Flippase Superfamily
			PAXXG191960	uncharacterized GPI-anchored protein At1g61900 isoform X1
			PAXXG326960	conserved protein of unknown function, supported by RNASeq data
			PAXXG326980	uncharacterized protein, homologue of XP_020571831.1
			PAXXG326990	cysteine-rich receptor-like protein kinase 10
			PAXXG327000	uncharacterized protein, homologue of XP_020582885.1
			PAXXG327020	conserved hypothetical protein homologue of PKU67230.1
			PAXXG327030	putative mitochondrial protein
			PAXXG326790	transcription factor bHLH52-like
			PAXXG326820	uncharacterized protein, homologue of XP_020599770.1
			PAXXG326890	RNA demethylase ALKBH5-like
3	L03	26551227~27918979	PAXXG086680	PHYTOCHROME KINASE SUBSTRATE 4-like
			PAXXG086700	EIL transcription factor
			PAXXG086710	trans-2-hexenal reductase
			PAXXG086720	Late embryogenesis abundant protein
			PAXXG086730	Transcription termination factor
			PAXXG086740	Transcription termination factor
			PAXXG086750	Protein argonaute 4B
			PAXXG086770	E3 ubiquitin-protein ligase RHA2B-like
			PAXXG086780	protein RETICULATA-RELATED 3, chloroplastic-like
			PAXXG086790	octanoyltransferase
			PAXXG086800	1-phosphatidylinositol-3-phosphate 5-kinase
			PAXXG086810	unknown protein, supported by RNASeq data
			PAXXG086820	uncharacterized protein, homologue of XP_020583537.1
			PAXXG086830	uncharacterized protein, homologue of PKU68428.1
			PAXXG086840	Transporter, the Lysosomal Cystine Transporter (LCT) Family
			PAXXG086850	cell number regulator 5
			PAXXG086870	uncharacterized protein, homologue of XP_020583520.1
			PAXXG086880	Transporter, the Type II (General) Secretory Pathway (IISP) Family
			PAXXG086890	calmodulin-binding protein 25-like
			PAXXG086900	MYB transcription factor
			PAXXG086940	RINT1-like protein MAG2L
			PAXXG086950	Transporter, the Drug/Metabolite Transporter (DMT) Superfamily
			PAXXG086960	30S ribosomal protein S4
			PAXXG086970	CBL-interacting protein kinase
			PAXXG086980	sigma factor binding protein 1, chloroplastic-like
			PAXXG086990	calmodulin-like
			PAXXG087010	flavonoid 3'5'-hydroxylase
			PAXXG087015	Unknown protein, supported by RNA-Seq data
			PAXXG087020	pentatricopeptide repeat-containing protein
			PAXXG087030	uncharacterized protein, homologue of XP_020598403.1
			PAXXG087040	translin-associated protein X
			PAXXG087050	DNA gyrase subunit B, chloroplastic/mitochondrial

			PAXXG087060	nitrilase-like protein
			PAXXG087070	putative nuclease HARBI1
			PAXXG087080	putative nuclease HARBI1
			PAXXG087090	Similar to BTB/POZ domain-containing protein
			PAXXG087100	uncharacterized protein, homologue of PKA58483.1
			PAXXG087110	transmembrane 9 superfamily member 1-like
			PAXXG087120	kinesin-related protein-like
			PAXXG087130	glycine-rich protein A3-like [Phoenix dactylifera]
			PAXXG087140	Protein NETWORKED 2A
			PAXXG087150	fructose-bisphosphate aldolase-lysine N-methyltransferase, chloroplastic
			PAXXG087160	uncharacterized protein, homologue of XP_020597354.1
			PAXXG087170	uncharacterized protein, homologue of PKU64702.1
			PAXXG087180	Transporter, the ATP-binding Cassette (ABC) Superfamily
			PAXXG087190	unknown protein, supported by RNASeq data
			PAXXG087200	Similar to Protein SHORT-ROOT INTERACTING EMBRYONIC LETHAL
			PAXXG087210	uncharacterized protein, homologue of XP_020599770.1
			PAXXG087220	uncharacterized protein, homologue of XP_020672437.1
			PAXXG087230	probable membrane-associated kinase regulator 4
			PAXXG087235	homologue of PKU65335.1, conserved hypothetical protein
			PAXXG087240	Similar to Symplekin
			PAXXG087250	E3 UFM1-protein ligase 1 homolog
			PAXXG087260	E3 UFM1-protein ligase 1 homolog
			PAXXG087270	Nudix hydrolase 9
			PAXXG087280	UDP-glucuronic acid decarboxylase 1
19	L19	9844529~11174194	PAXXG294060	ras-related protein RABF1
			PAXXG294070	Transporter, the ATP-binding Cassette (ABC) Superfamily
			PAXXG294080	40S ribosomal protein S17-3-like
			PAXXG337540	MYB transcription factor
			PAXXG337550	uncharacterized protein, homologue of XP_020598499.1
			PAXXG337610	uncharacterized protein, homologue of XP_020596058.1
			PAXXG320520	serine carboxypeptidase-like 34
			PAXXG320540	uncharacterized protein, homologue of XP_020596058.1
			PAXXG320550	uncharacterized protein, homologue of XP_020598318.1
			PAXXG320570	uncharacterized protein, homologue of XP_020597234.1
			PAXXG320580	uncharacterized protein, homologue of XP_020596848.1
			PAXXG320590	uncharacterized protein, homologue of XP_020593563.1
			PAXXG320600	uncharacterized protein, homologue of XP_020598318.1
			PAXXG272950	Transporter, heavy metal-associated isoprenylated plant protein 3-like
			PAXXG272960	conserved hypothetical protein homologue of PKU67230.1
			PAXXG272970	conserved protein of unknown function, supported by RNASeq data
			PAXXG272980	ADP-ribosylation factor
			PAXXG273000	uncharacterized protein, homologue of XP_020592067.1
			PAXXG273010	uncharacterized protein, homologue of XP_020592067.1
			PAXXG273020	4-coumarate--CoA ligase-like 7
			PAXXG273040	hypothetical protein, weak similar to PKU69516.1
			PAXXG273050	conserved hypothetical protein homologue of PKU67230.1
			PAXXG273060	RNA demethylase ALKBH5-like
			PAXXG273070	RNA demethylase ALKBH5-like
			PAXXG273080	uncharacterized protein, homologue of XP_020598333.1
			PAXXG273100	hydroxyethylthiazole kinase-like
			PAXXG273110	uncharacterized protein, homologue of XP_020593563.1
			PAXXG273150	uncharacterized protein, homologue of XP_020675309.1
			PAXXG273160	uncharacterized protein, homologue of XP_020598674.1
			PAXXG273170	uncharacterized protein, homologue of XP_020582885.1
			PAXXG273190	uncharacterized protein, homologue of XP_020599380.1
			PAXXG273200	uncharacterized protein, homologue of XP_020598333.1
			PAXXG273220	uncharacterized protein, homologue of XP_020585229.1
			PAXXG343890	cell division cycle protein 48 homolog
			PAXXG343895	Unknown protein, supported by RNA-Seq data
10	L10a	16194077~17395927	PAXXG274460	uncharacterized protein, homologue of XP_020593563.1
			PAXXG274480	RNA demethylase ALKBH5-like
			PAXXG274490	uncharacterized protein, homologue of XP_020598317.1
			PAXXG274500	conserved hypothetical protein homologue of PKU82262.1
			PAXXG274550	uncharacterized protein, homologue of XP_020598674.1
			PAXXG274570	uncharacterized protein, homologue of XP_020598333.1
			PAXXG274580	uncharacterized protein, homologue of XP_020598317.1
			PAXXG274610	RNA demethylase ALKBH5-like
			PAXXG274630	RNA demethylase ALKBH5-like
			PAXXG249120	uncharacterized protein, homologue of XP_020592939.1
			PAXXG249130	uncharacterized protein, homologue of XP_020575870.1
			PAXXG249150	uncharacterized protein, homologue of XP_020582885.1
			PAXXG249170	RNA demethylase ALKBH5-like
			PAXXG249180	glucan endo-1,3-beta-D-glucosidase-like
			PAXXG249190	peptide methionine sulfoxide reductase B1, chloroplastic isoform X1
			PAXXG249200	RNA demethylase ALKBH5-like
			PAXXG249210	uncharacterized protein, homologue of XP_020595829.1
			PAXXG249220	conserved protein of unknown function, supported by RNASeq data
			PAXXG249230	uncharacterized protein, homologue of XP_020597778.1
			PAXXG249260	uncharacterized protein, homologue of XP_020671729.1
			PAXXG249280	uncharacterized protein, homologue of XP_020596142.1
			PAXXG249290	uncharacterized protein, homologue of XP_020593563.1
			PAXXG249320	RNA demethylase ALKBH5-like
			PAXXG249330	RNA demethylase ALKBH5-like
			PAXXG249360	uncharacterized protein, homologue of XP_020599644.1
			PAXXG249370	RNA demethylase ALKBH5-like
			PAXXG249420	uncharacterized protein, homologue of XP_020598317.1
			PAXXG342330	C2H2 transcription factor
9	L09	21987186~23187808	PAXXG230910	3-phosphoshikimate1-carboxyvinyl transferase
			PAXXG184900	uncharacterized protein, homologue of XP_020596995.1
			PAXXG184910	uncharacterized protein, homologue of XP_020598825.1
			PAXXG184920	synaptotagmin-5
			PAXXG184930	protein MICRORCHIDIA 2-like isoform X1

			PAXXG184940	subtilisin-like protease SBT1.2
			PAXXG184950	Transporter, the Multidrug/Oligosaccharidyl-lipid/Polysaccharide (MOP) Flippase Superfamily
			PAXXG184960	protein DETOXIFICATION 14-like
			PAXXG184970	nudix hydrolase 14, chloroplastic
			PAXXG184990	cytochrome P450 704C1-like
			PAXXG185000	pentatricopeptide repeat-containing protein At5g66520-like
			PAXXG185010	uncharacterized protein, homologue of XP_020597892.1
			PAXXG185020	probable aminotransferase ACS12
			PAXXG185050	uncharacterized protein, homologue of XP_020597896.1
			PAXXG185060	alkylated DNA repair protein alkB 5
			PAXXG185080	bHLH transcription factor
			PAXXG185090	Transporter, the H+- or Na+-translocating F-type, V-type and A-type ATPase (F-ATPase) Superfamily
			PAXXG185100	Unknown protein, supported by RNA-Seq data
			PAXXG185110	RNA-directed DNA polymerase like
			PAXXG185120	arginine decarboxylase-like
			PAXXG185130	NADP-dependent D-sorbitol-6-phosphate dehydrogenase-like
			PAXXG185150	uncharacterized protein, homologue of XP_020596058.1
			PAXXG185160	conserved protein of unknown function, supported by RNASeq data
			PAXXG185180	60S acidic ribosomal protein P1-like
			PAXXG185190	ataxia telangiectasia mutated family protein
			PAXXG185200	putative mitochondrial protein
			PAXXG185210	hypothetical protein, weak similar to PKA60916.1
			PAXXG185220	uncharacterized protein, homologue of XP_020582663.1
			PAXXG185230	conserved protein of unknown function, supported by RNASeq data
16	L16	7092819~8236452	PAXXG228490	AGL6-like, PaAGL9
			PAXXG228500	caffeic acid 3-O-methyltransferase-like
			PAXXG228510	caffeic acid 3-O-methyltransferase-like
			PAXXG228520	probable carboxylesterase 2
			PAXXG228530	uncharacterized protein, homologue of XP_020579262.1
			PAXXG228550	60S ribosomal protein L12
			PAXXG228560	uncharacterized protein, homologue of XP_020685333.1
			PAXXG228570	cellulose synthase-like protein D2
			PAXXG228580	binding partner of ACD11 1-like
			PAXXG228590	uncharacterized protein, homologue of XP_020595280.1
			PAXXG228600	pentatricopeptide repeat-containing protein At1g71210
			PAXXG228610	mRNA-decapping enzyme subunit 2
			PAXXG228630	Transporter, the HlyC/CorC (HCC) Family
			PAXXG228640	uncharacterized protein, homologue of XP_020599770.1
			PAXXG228660	calcium-dependent protein kinase 18-like
			PAXXG228670	auxin response factor 5-like
			PAXXG228680	conserved hypothetical protein homologue of PKA59528.1
			PAXXG228690	B3 transcription factor
			PAXXG228700	uncharacterized protein ycf37 isoform X1
			PAXXG228710	peptide methionine sulfoxide reductase A5 isoform X1
			PAXXG228720	uncharacterized protein, homologue of XP_020579249.1
			PAXXG228730	C2C2-GATA transcription factor
			PAXXG228740	uncharacterized protein, homologue of XP_020579286.1
			PAXXG228750	conserved protein of unknown function, supported by RNASeq data
			PAXXG228760	uncharacterized protein, homologue of XP_020579276.1
			PAXXG228770	conserved protein of unknown function, supported by RNASeq data
			PAXXG228790	enoyl-CoA hydratase 2, peroxisomal isoform X2
			PAXXG228795	Unknown protein, supported by RNA-Seq data, weak similar to PKU85612.1
			PAXXG228810	serine/threonine-protein kinase STY46-like isoform X1
			PAXXG324820	DE-ETIOLATED 1,a repressor of photomorphogenesis
			PAXXG324830	cell number regulator 6-like
			PAXXG324850	uncharacterized protein, homologue of XP_020580825.1
			PAXXG324860	histone deacetylase complex subunit SAP18
			PAXXG324870	uncharacterized protein, homologue of XP_020590010.1
			PAXXG324880	pseudo-response regulator
			PAXXG324890	uncharacterized protein, homologue of XP_020592521.1
			PAXXG324900	Pentatricopeptide repeat-containing protein
19	L19	4717985~5847233	PAXXG333490	phosphoglucan phosphatase DSP4, amyloplastic
			PAXXG333495	L10-interacting MYB domain-containing protein-like
			PAXXG333510	conserved protein of unknown function, supported by RNASeq data
			PAXXG274080	RNA demethylase ALKBH5-like
			PAXXG274090	uncharacterized protein, homologue of XP_020593563.1
			PAXXG274110	uncharacterized protein, homologue of XP_020572381.1
			PAXXG274120	uncharacterized protein, homologue of XP_020588389.1
			PAXXG274125	phosphatidylinositol-glycan biosynthesis class X protein
			PAXXG274130	uncharacterized protein, homologue of XP_020588389.1
			PAXXG274145	uncharacterized protein, homologue of XP_020598766.1
			PAXXG274180	serine carboxypeptidase-like
			PAXXG274190	UDP-glycosyltransferase 91C1-like
			PAXXG274200	putative UDP-rhamnose:rhamnosyltransferase 1
			PAXXG274210	putative UDP-rhamnose:rhamnosyltransferase 1
			PAXXG274220	Transporter, One of secondary transporter type family
			PAXXG351060	pentatricopeptide repeat-containing protein At5g39350-like
			PAXXG351080	Transporter, the P-type ATPase (P-ATPase) Superfamily
			PAXXG294680	conserved hypothetical protein homologue of PKU67230.1
			PAXXG294700	conserved hypothetical protein homologue of PKU67230.1
			PAXXG294720	methylsterol monooxygenase 1-1-like
			PAXXG294730	a homolog of human Lysine-Specific Demethylase1
1	L01	10090555~11119372	PAXXG346670	pentatricopeptide repeat-containing protein At2g03880, mitochondrial
			PAXXG346680	putative pentatricopeptide repeat-containing protein At5g40405
			PAXXG305970	Ycf2
			PAXXG305980	Hypothetical protein, supported by RNA-Seq data
			PAXXG306010	plant cysteine oxidase 2
			PAXXG306020	Transporter, the Drug/Metabolite Transporter (DMT) Superfamily
			PAXXG306030	uncharacterized protein, homologue of XP_020591022.1
			PAXXG306050	acyl-CoA-binding domain-containing protein 3-like
			PAXXG306060	probable L-ascorbate peroxidase 4
			PAXXG306070	putative mitochondrial protein

			PAXXG306080	ribosomal RNA small subunit methyltransferase, chloroplastic isoform X3
			PAXXG338780	uncharacterized protein, homologue of XP_020595829.1
			PAXXG338800	WRKY transcription factor
			PAXXG338810	conserved protein of unknown function, supported by RNASeq data
			PAXXG311290	serine/threonine-protein phosphatase PP2A-1 catalytic subunit
			PAXXG311300	protein CLP1 homolog
			PAXXG311310	sucrose transport protein SUT4-like isoform X1
			PAXXG311330	ras-related protein Rab7-like isoform X1
			PAXXG311340	uncharacterized protein, homologue of XP_020597084.1
			PAXXG311350	RNA polymerase sigma factor sigB
15	L15	8147145~9165339	PAXXG238130	uncharacterized protein, homologue of XP_020594067.1
			PAXXG238160	alpha-aminoadipic semialdehyde synthase
			PAXXG238170	uncharacterized protein, homologue of XP_020599770.1
			PAXXG238180	RING-H2 finger protein ATL60-like
			PAXXG238190	bZIP transcription factor
			PAXXG238200	uncharacterized protein, homologue of XP_020685009.1
			PAXXG238210	xin actin-binding repeat-containing protein 2
			PAXXG298720	CAMTA transcription factor
			PAXXG298740	uncharacterized protein, homologue of XP_020579458.1
			PAXXG298750	uncharacterized protein, homologue of XP_020575840.1
			PAXXG298760	ras-related protein RABH1e
			PAXXG298770	pentatricopeptide repeat-containing protein At4g19890
			PAXXG298790	uncharacterized protein, homologue of XP_020579458.1
			PAXXG298800	conserved protein of unknown function, supported by RNASeq data
			PAXXG298810	Transporter, the Annexin (Annexin) Family
			PAXXG298820	uncharacterized protein, homologue of XP_020589922.1
			PAXXG298830	eukaryotic translation initiation factor 3 subunit I-like
			PAXXG298840	L10-interacting MYB domain-containing protein-like
			PAXXG298860	conserved hypothetical protein homologue of PKU67230.1
			PAXXG298870	protein piccolo-like
			PAXXG298880	RNA demethylase ALKBH5-like
			PAXXG354470	uncharacterized protein, homologue of XP_020571831.1
			PAXXG354480	multifunctional methyltransferase subunit TRM112-like protein At1g22270
			PAXXG354490	uncharacterized protein, homologue of XP_020575870.1

Table S13 List of transcription factors in *P. aphrodite*

Gene ID	TF Family	Chromosome	Scaffold	start	end	strand
PAXXG027180	Alfin-like	Chr13	scaffold9	3297998	3355680	+
PAXXG037960	Alfin-like	Chr15	scaffold14	1049260	1082729	+
PAXXG049070	Alfin-like	Chr07	scaffold19	1249918	1267751	-
PAXXG187480	Alfin-like	Chr07	scaffold176	108614	116667	-
PAXXG200590	Alfin-like	Chr11	scaffold202	236376	281818	-
PAXXG220900	Alfin-like	Chr01	scaffold255	382732	395270	-
PAXXG272830	Alfin-like	unmapped	scaffold430	325946	352329	+
PAXXG017870	AP2/ERF-AP2	Chr18	scaffold5	1798083	1804298	-
PAXXG051950	AP2/ERF-AP2	Chr07	scaffold20	3604099	3608534	-
PAXXG069170	AP2/ERF-AP2	Chr03	scaffold31	1131523	1134850	-
PAXXG069560	AP2/ERF-AP2	Chr03	scaffold31	1899027	1902762	+
PAXXG123020	AP2/ERF-AP2	Chr01	scaffold80	702100	709365	+
PAXXG130540	AP2/ERF-AP2	unmapped	scaffold88	1532623	1540578	+
PAXXG130680	AP2/ERF-AP2	Chr11	scaffold89	44237	47169	+
PAXXG185280	AP2/ERF-AP2	Chr08	scaffold172	1028	10437	+
PAXXG201440	AP2/ERF-AP2	unmapped	scaffold204	106454	109790	-
PAXXG221920	AP2/ERF-AP2	Chr16	scaffold257	568733	574556	-
PAXXG248340	AP2/ERF-AP2	Chr11	scaffold336	88684	91884	+
PAXXG251750	AP2/ERF-AP2	Chr11	scaffold347	429	3923	+
PAXXG333170	AP2/ERF-AP2	unmapped	scaffold862	11253	17696	-
PAXXG001190	AP2/ERF-ERF	Chr02	scaffold1	2327326	2328245	-
PAXXG001200	AP2/ERF-ERF	Chr02	scaffold1	2337433	2338390	+
PAXXG002200	AP2/ERF-ERF	Chr02	scaffold1	4577991	4579046	-
PAXXG009550	AP2/ERF-ERF	Chr08	scaffold3	1552110	1554936	+
PAXXG010890	AP2/ERF-ERF	Chr08	scaffold3	3754504	3755459	+
PAXXG013980	AP2/ERF-ERF	Chr15	scaffold4	2655607	2658832	-
PAXXG015350	AP2/ERF-ERF	Chr15	scaffold4	4946008	4946971	+
PAXXG015360	AP2/ERF-ERF	Chr15	scaffold4	4956016	4956805	+
PAXXG016990	AP2/ERF-ERF	Chr18	scaffold5	145578	146682	+
PAXXG018360	AP2/ERF-ERF	Chr18	scaffold5	2863982	2865239	-
PAXXG019030	AP2/ERF-ERF	Chr18	scaffold5	4161928	4162986	-
PAXXG028820	AP2/ERF-ERF	Chr14	scaffold10	1325768	1326186	+
PAXXG028830	AP2/ERF-ERF	Chr14	scaffold10	1335187	1336116	+
PAXXG029340	AP2/ERF-ERF	Chr14	scaffold10	2283353	2284187	-
PAXXG036150	AP2/ERF-ERF	Chr16	scaffold13	1982094	1984384	+
PAXXG037480	AP2/ERF-ERF	Chr15	scaffold14	161052	174993	+
PAXXG044460	AP2/ERF-ERF	Chr12*	scaffold17	1789162	1790448	-
PAXXG048700	AP2/ERF-ERF	Chr07	scaffold19	291052	291588	-
PAXXG054530	AP2/ERF-ERF	Chr15	scaffold22	1385897	1401100	-
PAXXG055170	AP2/ERF-ERF	Chr15	scaffold22	2792491	2801203	+
PAXXG055800	AP2/ERF-ERF	Chr01	scaffold23	939946	942112	+
PAXXG064500	AP2/ERF-ERF	Chr01	scaffold28	1039256	1040134	+
PAXXG068120	AP2/ERF-ERF	Chr12	scaffold30	2000366	2021164	-
PAXXG076650	AP2/ERF-ERF	Chr02	scaffold36	1510735	1511390	-
PAXXG077090	AP2/ERF-ERF	Chr02	scaffold36	2881487	2883923	-
PAXXG084990	AP2/ERF-ERF	Chr03	scaffold42	1035690	1056047	+

PAXXG089810	AP2/ERF-ERF	Chr04	scaffold46	2027424	2028576	+
PAXXG097260	AP2/ERF-ERF	Chr04	scaffold54	817933	818775	+
PAXXG097280	AP2/ERF-ERF	Chr04	scaffold54	837738	838552	+
PAXXG097770	AP2/ERF-ERF	Chr04	scaffold54	1916627	1917085	+
PAXXG100110	AP2/ERF-ERF	Chr09	scaffold57	377732	378574	-
PAXXG102660	AP2/ERF-ERF	Chr16	scaffold58	2189280	2190355	+
PAXXG103670	AP2/ERF-ERF	Chr12	scaffold59	1650229	1651859	+
PAXXG106570	AP2/ERF-ERF	Chr06	scaffold62	1909256	1910708	+
PAXXG108170	AP2/ERF-ERF	Chr03	scaffold64	1341582	1342869	-
PAXXG111610	AP2/ERF-ERF	Chr08*	scaffold68	630310	632970	-
PAXXG112890	AP2/ERF-ERF	Chr19	scaffold69	919820	920488	+
PAXXG114100	AP2/ERF-ERF	Chr09	scaffold70	776637	778807	-
PAXXG114490	AP2/ERF-ERF	Chr09	scaffold70	1413422	1414277	+
PAXXG125250	AP2/ERF-ERF	Chr04	scaffold82	1511318	1511835	-
PAXXG128820	AP2/ERF-ERF	Chr02	scaffold87	153204	154728	+
PAXXG137350	AP2/ERF-ERF	Chr03	scaffold96	1285889	1286823	-
PAXXG140890	AP2/ERF-ERF	Chr03	scaffold99	1490777	1491952	-
PAXXG145370	AP2/ERF-ERF	Chr11	scaffold105	1172374	1173744	+
PAXXG146450	AP2/ERF-ERF	Chr02	scaffold107	154259	155998	-
PAXXG151180	AP2/ERF-ERF	Chr09	scaffold114	1448354	1449490	-
PAXXG151190	AP2/ERF-ERF	Chr09	scaffold114	1454415	1455936	-
PAXXG153000	AP2/ERF-ERF	Chr03	scaffold118	222578	224042	-
PAXXG153020	AP2/ERF-ERF	Chr03	scaffold118	271178	272195	-
PAXXG153040	AP2/ERF-ERF	Chr03	scaffold118	309622	310397	+
PAXXG153750	AP2/ERF-ERF	Chr10	scaffold119	285146	292581	+
PAXXG153760	AP2/ERF-ERF	Chr10	scaffold119	293591	296845	+
PAXXG154440	AP2/ERF-ERF	Chr10	scaffold119	1473933	1484406	-
PAXXG155980	AP2/ERF-ERF	Chr10	scaffold121	1634985	1636809	+
PAXXG156380	AP2/ERF-ERF	Chr10	scaffold121	2237180	2241418	-
PAXXG162830	AP2/ERF-ERF	Chr01	scaffold130	1693195	1694524	+
PAXXG163960	AP2/ERF-ERF	Chr03	scaffold132	1383732	1386149	-
PAXXG166830	AP2/ERF-ERF	Chr05	scaffold137	1378952	1379713	+
PAXXG168110	AP2/ERF-ERF	Chr17	scaffold140	407439	408614	-
PAXXG168120	AP2/ERF-ERF	Chr17	scaffold140	429151	430520	-
PAXXG169490	AP2/ERF-ERF	Chr15	scaffold142	1343450	1345237	-
PAXXG173070	AP2/ERF-ERF	Chr08	scaffold149	313775	314501	-
PAXXG174710	AP2/ERF-ERF	Chr19	scaffold151	907679	908137	-
PAXXG174740	AP2/ERF-ERF	Chr19	scaffold151	955118	955911	+
PAXXG179770	AP2/ERF-ERF	Chr07	scaffold162	448830	449612	+
PAXXG180420	AP2/ERF-ERF	Chr09	scaffold163	633747	634904	-
PAXXG180460	AP2/ERF-ERF	Chr09	scaffold163	856991	857734	-
PAXXG180470	AP2/ERF-ERF	Chr09	scaffold163	862329	863556	-
PAXXG182310	AP2/ERF-ERF	Chr06	scaffold166	851751	853419	-
PAXXG183020	AP2/ERF-ERF	Chr02	scaffold167	887407	887754	-
PAXXG186940	AP2/ERF-ERF	unmapped	scaffold174	856530	858166	-
PAXXG187280	AP2/ERF-ERF	Chr12	scaffold175	817507	818240	+
PAXXG187770	AP2/ERF-ERF	Chr07	scaffold176	736384	737172	-
PAXXG189370	AP2/ERF-ERF	Chr15	scaffold180	273381	274204	-

PAXXG193010	AP2/ERF-ERF	Chr13	scaffold188	6496	7509	-
PAXXG201470	AP2/ERF-ERF	unmapped	scaffold204	186222	186879	-
PAXXG206960	AP2/ERF-ERF	unmapped	scaffold217	525895	527115	-
PAXXG218050	AP2/ERF-ERF	unmapped	scaffold248	520950	521954	+
PAXXG219790	AP2/ERF-ERF	Chr13	scaffold253	418139	419119	-
PAXXG220450	AP2/ERF-ERF	Chr02	scaffold254	435112	437059	+
PAXXG229060	AP2/ERF-ERF	unmapped	scaffold276	575279	576599	+
PAXXG231450	AP2/ERF-ERF	unmapped	scaffold284	625549	626235	+
PAXXG233400	AP2/ERF-ERF	Chr15	scaffold290	487084	488950	+
PAXXG240610	AP2/ERF-ERF	Chr09	scaffold312	378325	378891	+
PAXXG249740	AP2/ERF-ERF	Chr14	scaffold340	288665	290379	+
PAXXG259770	AP2/ERF-ERF	Chr10	scaffold374	415405	416142	-
PAXXG260120	AP2/ERF-ERF	Chr10	scaffold375	415631	417068	-
PAXXG265960	AP2/ERF-ERF	unmapped	scaffold398	608054	609115	+
PAXXG270410	AP2/ERF-ERF	Chr18	scaffold420	361074	361861	-
PAXXG274060	AP2/ERF-ERF	Chr02	scaffold435	524223	525506	+
PAXXG282680	AP2/ERF-ERF	Chr02	scaffold475	122209	123092	-
PAXXG291050	AP2/ERF-ERF	Chr04	scaffold522	347849	349056	-
PAXXG308700	AP2/ERF-ERF	Chr15	scaffold634	353394	354227	-
PAXXG324910	AP2/ERF-ERF	Chr13	scaffold772	4871	8030	+
PAXXG326370	AP2/ERF-ERF	unmapped	scaffold784	99574	100210	+
PAXXG326910	AP2/ERF-ERF	Chr15	scaffold792	123284	124598	-
PAXXG349610	AP2/ERF-ERF	unmapped	scaffold1137	44019	45270	-
PAXXG382460	AP2/ERF-ERF	unmapped	scaffold4748	7695	8882	-
PAXXG043830	AP2/ERF-RAV	Chr12*	scaffold17	519913	521216	+
PAXXG122510	AP2/ERF-RAV	Chr07*	scaffold79	1358161	1359445	-
PAXXG222020	AP2/ERF-RAV	Chr16	scaffold257	903458	904991	+
PAXXG000740	B3	Chr02	scaffold1	1228632	1231731	-
PAXXG006850	B3	Chr10	scaffold2	6830030	6840674	+
PAXXG006860	B3	Chr10	scaffold2	6841800	6848633	+
PAXXG011010	B3	Chr08	scaffold3	4069103	4077962	-
PAXXG022220	B3	Chr11	scaffold7	923870	930076	+
PAXXG038580	B3	Chr15	scaffold14	2275347	2281240	-
PAXXG047260	B3	Chr17	scaffold18	2949119	2980207	-
PAXXG067700	B3	Chr12	scaffold30	868922	881501	+
PAXXG082950	B3	Chr03	scaffold40	1408669	1411599	-
PAXXG087550	B3	Chr03	scaffold44	2216745	2222001	+
PAXXG089740	B3	Chr04	scaffold46	1939433	1970545	+
PAXXG095190	B3	Chr09	scaffold51	2054288	2060896	+
PAXXG100950	B3	Chr09	scaffold57	1921826	1958819	-
PAXXG137180	B3	Chr03	scaffold96	878429	887280	+
PAXXG140840	B3	Chr03	scaffold99	1358758	1384847	+
PAXXG168460	B3	Chr10	scaffold141	209122	280127	+
PAXXG181480	B3	unmapped	scaffold165	817921	904788	+
PAXXG193700	B3	Chr13	scaffold188	1100266	1101883	-
PAXXG193710	B3	Chr13	scaffold188	1103563	1104732	-
PAXXG193720	B3	Chr13	scaffold188	1107550	1114041	-
PAXXG193730	B3	Chr13	scaffold188	1118629	1120067	-

PAXXG193740	B3	Chr13	scaffold188	1125565	1127286	-
PAXXG193750	B3	Chr13	scaffold188	1134566	1136082	-
PAXXG193760	B3	Chr13	scaffold188	1138443	1139986	-
PAXXG193770	B3	Chr13	scaffold188	1143398	1144285	-
PAXXG193780	B3	Chr13	scaffold188	1151461	1152351	-
PAXXG193790	B3	Chr13	scaffold188	1159960	1160826	-
PAXXG193800	B3	Chr13	scaffold188	1163759	1164574	-
PAXXG201710	B3	unmapped	scaffold204	642710	644891	+
PAXXG222040	B3	Chr16	scaffold257	913848	914989	+
PAXXG222050	B3	Chr16	scaffold257	921361	922227	+
PAXXG222060	B3	Chr16	scaffold257	924786	926161	+
PAXXG222070	B3	Chr16	scaffold257	937076	943199	+
PAXXG226540	B3	Chr08	scaffold269	1274606	1276803	-
PAXXG228690	B3	Chr16	scaffold275	339475	348557	-
PAXXG231270	B3	unmapped	scaffold284	341269	345600	+
PAXXG242760	B3	unmapped	scaffold319	547832	612379	+
PAXXG268420	B3	Chr06	scaffold410	226083	226939	-
PAXXG270290	B3	Chr18	scaffold420	165697	170881	+
PAXXG329060	B3	Chr18	scaffold812	135780	221142	-
PAXXG002950	B3-ARF	Chr02	scaffold1	6218513	6257174	+
PAXXG032340	B3-ARF	Chr10	scaffold11	3805845	3807954	+
PAXXG032350	B3-ARF	Chr10	scaffold11	3818384	3828839	+
PAXXG059450	B3-ARF	Chr04	scaffold25	1362049	1385170	+
PAXXG071970	B3-ARF	Chr02	scaffold33	1779325	1783258	-
PAXXG098760	B3-ARF	Chr01	scaffold55	2034472	2100169	+
PAXXG146200	B3-ARF	Chr13	scaffold106	1002332	1007286	-
PAXXG146570	B3-ARF	Chr02	scaffold107	616759	622560	+
PAXXG160100	B3-ARF	unmapped	scaffold127	44866	60698	+
PAXXG182870	B3-ARF	Chr02	scaffold167	557345	563823	+
PAXXG189800	B3-ARF	Chr15	scaffold180	903699	911419	-
PAXXG201900	B3-ARF	unmapped	scaffold204	973285	985003	-
PAXXG224100	B3-ARF	unmapped	scaffold263	324014	328410	-
PAXXG257250	B3-ARF	Chr10	scaffold367	317995	367036	-
PAXXG275340	B3-ARF	unmapped	scaffold442	160938	164923	+
PAXXG285910	B3-ARF	unmapped	scaffold494	85408	89046	+
PAXXG328840	B3-ARF	unmapped	scaffold810	88542	140549	-
PAXXG015100	BBR-BPC	Chr15	scaffold4	4471514	4473112	-
PAXXG065770	BBR-BPC	Chr04	scaffold29	775192	776199	+
PAXXG124150	BBR-BPC	Chr15	scaffold81	1287811	1315721	-
PAXXG162270	BBR-BPC	Chr01	scaffold130	733622	735048	-
PAXXG173380	BBR-BPC	Chr08	scaffold149	768022	769585	+
PAXXG050450	BES1	Chr07	scaffold20	1155797	1158131	-
PAXXG112360	BES1	Chr19	scaffold69	166957	180852	+
PAXXG166350	BES1	Chr05	scaffold137	698451	700812	-
PAXXG194510	BES1	Chr01	scaffold190	429969	503929	+
PAXXG242820	BES1	Chr12	scaffold320	97696	101300	-
PAXXG001550	bHLH	Chr02	scaffold1	3198192	3199906	-
PAXXG009200	bHLH	Chr08	scaffold3	1006717	1008962	+

PAXXG010930	bHLH	Chr08	scaffold3	3901015	3901919	+
PAXXG011930	bHLH	Chr08	scaffold3	5931368	5942165	-
PAXXG015740	bHLH	Chr15	scaffold4	5620878	5622551	+
PAXXG017590	bHLH	Chr18	scaffold5	1344636	1348913	+
PAXXG019000	bHLH	Chr18	scaffold5	4088762	4119472	+
PAXXG019660	bHLH	Chr04	scaffold6	222532	252950	+
PAXXG023540	bHLH	Chr11	scaffold7	4440733	4443747	-
PAXXG030200	bHLH	Chr14	scaffold10	3828527	3829366	-
PAXXG031560	bHLH	Chr10	scaffold11	2178968	2185641	-
PAXXG032310	bHLH	Chr10	scaffold11	3743737	3762300	+
PAXXG033020	bHLH	Chr04	scaffold12	670221	672956	-
PAXXG034830	bHLH	Chr04	scaffold12	3768315	3773477	-
PAXXG038800	bHLH	Chr15	scaffold14	2821023	2826480	+
PAXXG038970	bHLH	Chr15	scaffold14	3119879	3121087	+
PAXXG040450	bHLH	Chr07	scaffold15	1803422	1823210	+
PAXXG048230	bHLH	Chr01	scaffold251	465910	467224	-
PAXXG051730	bHLH	Chr07	scaffold20	3172978	3175759	+
PAXXG052970	bHLH	Chr18	scaffold21	1902210	1933983	-
PAXXG053140	bHLH	Chr18	scaffold21	2367066	2367669	-
PAXXG053580	bHLH	Chr18	scaffold21	3276330	3315307	-
PAXXG058170	bHLH	Chr13	scaffold24	2498787	2517095	+
PAXXG059840	bHLH	Chr04	scaffold25	2360631	2362022	-
PAXXG065660	bHLH	Chr04	scaffold29	577011	580176	-
PAXXG068320	bHLH	Chr12	scaffold30	2620261	2623082	+
PAXXG079320	bHLH	Chr06	scaffold38	299316	300764	-
PAXXG079480	bHLH	Chr06	scaffold38	551269	558478	-
PAXXG079590	bHLH	Chr06	scaffold38	730123	730948	+
PAXXG080390	bHLH	Chr06	scaffold38	1988915	1991777	+
PAXXG080500	bHLH	Chr06	scaffold38	2119360	2121773	-
PAXXG080960	bHLH	Chr04	scaffold39	244319	268407	-
PAXXG085670	bHLH	Chr18	scaffold43	487352	488856	+
PAXXG086140	bHLH	Chr18	scaffold43	1585466	1588512	+
PAXXG093760	bHLH	Chr03	scaffold50	970814	975535	+
PAXXG103860	bHLH	Chr12	scaffold59	1973962	1974954	+
PAXXG106100	bHLH	Chr06	scaffold62	1024065	1032128	-
PAXXG112830	bHLH	Chr19	scaffold69	823176	829545	-
PAXXG120060	bHLH	Chr11	scaffold76	1505913	1509226	+
PAXXG122980	bHLH	Chr01	scaffold80	626962	628606	+
PAXXG130900	bHLH	Chr11	scaffold89	434993	437848	-
PAXXG131810	bHLH	Chr11	scaffold89	2068439	2074624	+
PAXXG133480	bHLH	Chr02	scaffold91	1779817	1782658	-
PAXXG136710	bHLH	Chr03	scaffold96	161221	163693	+
PAXXG141700	bHLH	Chr02	scaffold100	1087711	1090299	+
PAXXG143860	bHLH	unmapped	scaffold104	115823	147188	+
PAXXG146700	bHLH	Chr02	scaffold107	1123321	1127789	-
PAXXG149370	bHLH	Chr07	scaffold111	1232481	1233384	+
PAXXG150520	bHLH	Chr09	scaffold114	14875	17344	-
PAXXG150610	bHLH	Chr09	scaffold114	169840	173613	-

PAXXG150860	bHLH	Chr09	scaffold114	618663	632123	+
PAXXG153570	bHLH	Chr03	scaffold118	1462231	1463939	+
PAXXG165950	bHLH	unmapped	scaffold136	1330636	1334788	+
PAXXG166560	bHLH	Chr05	scaffold137	937009	938337	-
PAXXG173670	bHLH	Chr09	scaffold150	26497	47467	+
PAXXG181800	bHLH	Chr06	scaffold166	107130	108444	-
PAXXG182880	bHLH	Chr02	scaffold167	568369	572292	-
PAXXG185080	bHLH	Chr09	scaffold171	721652	723136	+
PAXXG185770	bHLH	Chr08	scaffold172	732748	734258	-
PAXXG186600	bHLH	unmapped	scaffold174	3322	6947	-
PAXXG189770	bHLH	Chr15	scaffold180	717428	718300	-
PAXXG194490	bHLH	Chr01	scaffold190	410079	419368	-
PAXXG207080	bHLH	unmapped	scaffold217	870835	873732	-
PAXXG208790	bHLH	Chr01	scaffold221	569157	570203	+
PAXXG208850	bHLH	Chr01	scaffold221	679738	699717	+
PAXXG211300	bHLH	unmapped	scaffold229	781361	783135	-
PAXXG211830	bHLH	Chr01	scaffold230	882155	883043	-
PAXXG213650	bHLH	unmapped	scaffold236	826810	867723	+
PAXXG214950	bHLH	Chr06	scaffold240	529367	531812	+
PAXXG216940	bHLH	unmapped	scaffold245	436544	442042	+
PAXXG220360	bHLH	Chr02	scaffold254	228006	232812	-
PAXXG221150	bHLH	Chr01	scaffold255	781659	784396	-
PAXXG222670	bHLH	Chr10	scaffold259	510496	515159	+
PAXXG223840	bHLH	Chr12	scaffold262	550582	553194	+
PAXXG224550	bHLH	Chr03	scaffold264	639518	642350	-
PAXXG236540	bHLH	Chr14	scaffold300	242006	244419	+
PAXXG239050	bHLH	Chr01	scaffold308	429870	430771	-
PAXXG247250	bHLH	unmapped	scaffold331	1254160	1258334	+
PAXXG251330	bHLH	Chr18	scaffold345	540528	544655	-
PAXXG263910	bHLH	unmapped	scaffold391	114295	115057	+
PAXXG280400	bHLH	unmapped	scaffold465	373202	375559	-
PAXXG282330	bHLH	unmapped	scaffold472	508448	509395	+
PAXXG292200	bHLH	unmapped	scaffold529	161238	161768	-
PAXXG299690	bHLH	unmapped	scaffold575	82598	83460	-
PAXXG327520	bHLH	Chr19	scaffold797	96454	100078	+
PAXXG332590	bHLH	unmapped	scaffold854	247244	250869	-
PAXXG337010	bHLH	unmapped	scaffold913	137476	139919	+
PAXXG338640	bHLH	unmapped	scaffold935	51702	53562	+
PAXXG341680	bHLH	Chr07	scaffold982	102553	105433	+
PAXXG353190	bHLH	unmapped	scaffold1227	6929	9367	+
PAXXG365810	bHLH	unmapped	scaffold1825	13314	15771	+
PAXXG383370	bHLH	unmapped	scaffold5016	2212	4569	+
PAXXG053630	BSD	Chr18	scaffold21	3448739	3518374	+
PAXXG001660	bZIP	Chr02	scaffold1	3357167	3365509	+
PAXXG008370	bZIP	Chr10	scaffold2	9890262	9898658	-
PAXXG017200	bZIP	Chr18	scaffold5	452708	467232	-
PAXXG020480	bZIP	Chr04	scaffold6	2247028	2248147	-
PAXXG023930	bZIP	Chr11	scaffold8	229354	229972	-

PAXXG024400	bZIP	Chr11	scaffold8	1543072	1569414	-
PAXXG027970	bZIP	Chr14	scaffold10	239604	241606	+
PAXXG028030	bZIP	Chr14	scaffold10	301599	309919	-
PAXXG034360	bZIP	Chr04	scaffold12	2965880	2966398	+
PAXXG036230	bZIP	Chr16	scaffold13	2094691	2124282	-
PAXXG039120	bZIP	Chr15	scaffold14	3443071	3449379	+
PAXXG044200	bZIP	Chr12*	scaffold17	1401670	1404328	-
PAXXG047250	bZIP	Chr17	scaffold18	2927139	2947597	-
PAXXG047270	bZIP	Chr17	scaffold18	2989402	2992911	-
PAXXG049330	bZIP	Chr07	scaffold19	1801481	1806404	+
PAXXG054240	bZIP	Chr15	scaffold22	754007	791171	-
PAXXG057300	bZIP	Chr13	scaffold24	739431	754288	+
PAXXG061060	bZIP	Chr15	scaffold26	1559568	1560594	+
PAXXG061550	bZIP	Chr15	scaffold26	2560381	2565817	+
PAXXG062480	bZIP	Chr08	scaffold27	728163	738153	+
PAXXG077160	bZIP	Chr10	scaffold37	80784	102811	+
PAXXG080630	bZIP	Chr06	scaffold38	2338881	2339846	-
PAXXG082310	bZIP	Chr03	scaffold40	118674	121394	-
PAXXG082760	bZIP	Chr03	scaffold40	1126140	1128966	+
PAXXG086080	bZIP	Chr18	scaffold43	1440659	1447254	+
PAXXG102130	bZIP	Chr16	scaffold58	1291522	1312736	+
PAXXG110970	bZIP	Chr14*	scaffold67	1578583	1586851	-
PAXXG111490	bZIP	Chr08*	scaffold68	232598	259431	-
PAXXG117250	bZIP	Chr05	scaffold73	1356018	1357038	-
PAXXG118330	bZIP	Chr10	scaffold75	429205	430585	-
PAXXG150320	bZIP	Chr10	scaffold113	697648	699943	+
PAXXG150800	bZIP	Chr09	scaffold114	528811	534646	-
PAXXG157360	bZIP	Chr12	scaffold123	666560	669360	-
PAXXG162740	bZIP	Chr01	scaffold130	1576925	1589291	+
PAXXG165890	bZIP	unmapped	scaffold136	996762	997430	-
PAXXG173120	bZIP	Chr08	scaffold149	436502	457785	-
PAXXG196580	bZIP	Chr13	scaffold194	163903	198877	+
PAXXG214600	bZIP	unmapped	scaffold239	911174	912396	-
PAXXG215190	bZIP	Chr06	scaffold240	838110	839069	+
PAXXG233800	bZIP	Chr01	scaffold291	709416	736718	+
PAXXG238190	bZIP	Chr15	scaffold305	400648	407976	+
PAXXG248410	bZIP	Chr11	scaffold336	205617	206045	-
PAXXG255350	bZIP	Chr10	scaffold360	62181	77792	-
PAXXG259450	bZIP	unmapped	scaffold373	283801	285240	+
PAXXG270610	bZIP	Chr03	scaffold421	328912	340133	-
PAXXG275220	bZIP	Chr11	scaffold441	488802	510370	-
PAXXG285750	bZIP	Chr07	scaffold493	211490	212946	+
PAXXG289490	bZIP	Chr09	scaffold514	302090	303405	+
PAXXG292990	bZIP	Chr13	scaffold534	460620	468321	-
PAXXG297760	bZIP	Chr06	scaffold564	306091	312915	+
PAXXG316480	bZIP	unmapped	scaffold696	164586	178244	+
PAXXG325030	bZIP	Chr13	scaffold772	115081	124749	+
PAXXG325420	bZIP	unmapped	scaffold776	21763	23012	+

PAXXG334300	bZIP	Chr09	scaffold873	71404	79718	-
PAXXG341620	bZIP	unmapped	scaffold981	159188	171671	+
PAXXG345290	bZIP	Chr01	scaffold1050	134347	172660	+
PAXXG364440	bZIP	unmapped	scaffold1732	26550	27686	-
PAXXG365360	bZIP	unmapped	scaffold1805	1177	19632	+
PAXXG365420	bZIP	unmapped	scaffold1808	1015	2238	-
PAXXG386560	bZIP	unmapped	scaffold6532	1336	2100	+
PAXXG010150	C2C2-CO-like	Chr08	scaffold3	2438744	2440701	-
PAXXG012810	C2C2-CO-like	Chr15	scaffold4	262708	264589	-
PAXXG018120	C2C2-CO-like	Chr18	scaffold5	2373178	2374698	-
PAXXG141050	C2C2-CO-like	Chr02	scaffold100	107260	117730	-
PAXXG176690	C2C2-CO-like	Chr11	scaffold155	1050159	1051222	-
PAXXG207970	C2C2-CO-like	unmapped	scaffold219	726779	751806	-
PAXXG253980	C2C2-CO-like	Chr18	scaffold356	43859	64876	+
PAXXG275020	C2C2-CO-like	Chr11	scaffold441	104811	127250	+
PAXXG358340	C2C2-CO-like	Chr07	scaffold1421	71989	73807	-
PAXXG000370	C2C2-Dof	Chr02	scaffold1	657760	659419	+
PAXXG022030	C2C2-Dof	Chr11	scaffold7	678057	679094	+
PAXXG022190	C2C2-Dof	Chr11	scaffold7	865179	865960	-
PAXXG028530	C2C2-Dof	Chr14	scaffold10	930655	932903	+
PAXXG029700	C2C2-Dof	Chr14	scaffold10	3076030	3078245	-
PAXXG030690	C2C2-Dof	Chr10	scaffold11	319210	332139	+
PAXXG036270	C2C2-Dof	Chr16	scaffold13	2254787	2256009	+
PAXXG042450	C2C2-Dof	Chr19	scaffold16	1121374	1123703	-
PAXXG044150	C2C2-Dof	Chr12*	scaffold17	1345425	1345989	+
PAXXG045570	C2C2-Dof	Chr12*	scaffold17	3924245	3925646	+
PAXXG052880	C2C2-Dof	Chr18	scaffold21	1823714	1824912	-
PAXXG066780	C2C2-Dof	Chr04	scaffold29	2449642	2451450	+
PAXXG081940	C2C2-Dof	Chr04	scaffold39	1896447	1898426	+
PAXXG083340	C2C2-Dof	Chr03	scaffold40	2314098	2315146	+
PAXXG121880	C2C2-Dof	Chr07*	scaffold79	309296	309891	-
PAXXG123300	C2C2-Dof	Chr01	scaffold80	1235853	1238528	+
PAXXG133780	C2C2-Dof	Chr03	scaffold92	738971	739891	+
PAXXG139200	C2C2-Dof	Chr17	scaffold98	1587150	1600717	-
PAXXG168010	C2C2-Dof	Chr17	scaffold140	45736	46499	+
PAXXG220540	C2C2-Dof	Chr02	scaffold254	567190	568544	-
PAXXG228940	C2C2-Dof	unmapped	scaffold276	253654	255569	-
PAXXG259990	C2C2-Dof	Chr10	scaffold375	23245	24810	-
PAXXG266060	C2C2-Dof	Chr10	scaffold400	196748	197733	+
PAXXG273790	C2C2-Dof	unmapped	scaffold434	344046	346307	-
PAXXG273850	C2C2-Dof	unmapped	scaffold434	489789	510361	-
PAXXG273870	C2C2-Dof	unmapped	scaffold434	546813	549270	-
PAXXG294530	C2C2-Dof	unmapped	scaffold545	339956	340674	+
PAXXG294640	C2C2-Dof	unmapped	scaffold545	454379	454896	+
PAXXG350710	C2C2-Dof	unmapped	scaffold1161	62433	63466	-
PAXXG360470	C2C2-Dof	unmapped	scaffold1513	39782	41458	+
PAXXG018580	C2C2-GATA	Chr18	scaffold5	3270482	3281836	-
PAXXG045290	C2C2-GATA	Chr12*	scaffold17	3578181	3580113	-

PAXXG059400	C2C2-GATA	Chr04	scaffold25	1213040	1216606	-
PAXXG065100	C2C2-GATA	Chr01	scaffold28	2634700	2635730	-
PAXXG075580	C2C2-GATA	Chr11	scaffold35	2035953	2037674	-
PAXXG087600	C2C2-GATA	Chr03	scaffold44	2330792	2343926	-
PAXXG088460	C2C2-GATA	Chr06	scaffold45	1984514	1997202	+
PAXXG093910	C2C2-GATA	Chr03	scaffold50	1301306	1302686	-
PAXXG106080	C2C2-GATA	Chr06	scaffold62	925763	926961	-
PAXXG114760	C2C2-GATA	Chr09	scaffold70	1902895	1904304	+
PAXXG119000	C2C2-GATA	Chr10	scaffold75	1742996	1748954	-
PAXXG144870	C2C2-GATA	Chr11	scaffold105	379447	392855	+
PAXXG148420	C2C2-GATA	Chr09	scaffold110	655575	672404	+
PAXXG190440	C2C2-GATA	Chr09	scaffold182	713889	715858	+
PAXXG220650	C2C2-GATA	Chr02	scaffold254	878454	880969	+
PAXXG225840	C2C2-GATA	unmapped	scaffold268	528975	530194	-
PAXXG228730	C2C2-GATA	Chr16	scaffold275	436196	437527	-
PAXXG234900	C2C2-GATA	Chr15	scaffold295	32370	33740	+
PAXXG236580	C2C2-GATA	Chr14	scaffold300	291518	292746	-
PAXXG247960	C2C2-GATA	Chr19	scaffold334	369484	370576	+
PAXXG271470	C2C2-GATA	unmapped	scaffold425	17036	17966	-
PAXXG288510	C2C2-GATA	Chr09	scaffold509	330836	333363	+
PAXXG014430	C2C2-LSD	Chr15	scaffold4	3337460	3364216	+
PAXXG057150	C2C2-LSD	Chr13	scaffold24	265699	285826	+
PAXXG171050	C2C2-LSD	Chr18	scaffold145	688295	716963	-
PAXXG262000	C2C2-LSD	Chr13	scaffold384	9889	38686	+
PAXXG055705	C2C2-YABBY	Chr01	scaffold23	649715	671668	-
PAXXG074390	C2C2-YABBY	Chr11	scaffold35	191425	192575	+
PAXXG074420	C2C2-YABBY	Chr11	scaffold35	221280	221825	+
PAXXG080020	C2C2-YABBY	Chr06	scaffold38	1301682	1309698	+
PAXXG093620	C2C2-YABBY	Chr03	scaffold50	657793	660180	+
PAXXG159750	C2C2-YABBY	Chr13	scaffold126	1022779	1026940	-
PAXXG190360	C2C2-YABBY	Chr09	scaffold182	422456	424180	+
PAXXG238100	C2C2-YABBY	unmapped	scaffold304	678462	744410	+
PAXXG343680	C2C2-YABBY	Chr10	scaffold1019	137766	162411	+
PAXXG000090	C2H2	Chr02	scaffold1	283573	284518	+
PAXXG002340	C2H2	Chr02	scaffold1	4903857	4904438	+
PAXXG007470	C2H2	Chr10	scaffold2	8245088	8275803	+
PAXXG007860	C2H2	Chr10	scaffold2	9015431	9017683	-
PAXXG015080	C2H2	Chr15	scaffold4	4433449	4435641	+
PAXXG017440	C2H2	Chr18	scaffold5	1085006	1085685	-
PAXXG021850	C2H2	Chr11	scaffold7	252812	253941	-
PAXXG023610	C2H2	Chr11	scaffold7	4575461	4577595	-
PAXXG025430	C2H2	Chr11	scaffold8	4291928	4294955	+
PAXXG031440	C2H2	Chr10	scaffold11	1900144	1901329	-
PAXXG031880	C2H2	Chr10	scaffold11	2820615	2839820	+
PAXXG036060	C2H2	Chr16	scaffold13	1846538	1848996	-
PAXXG036130	C2H2	Chr16	scaffold13	1913883	1915807	-
PAXXG037710	C2H2	Chr15	scaffold14	610432	627032	-
PAXXG037770	C2H2	Chr15	scaffold14	694834	695601	+

PAXXG037970	C2H2	Chr15	scaffold14	1103331	1104260	+
PAXXG043120	C2H2	Chr19	scaffold16	2577602	2578225	+
PAXXG045170	C2H2	Chr12*	scaffold17	3338599	3343318	-
PAXXG046800	C2H2	Chr17	scaffold18	2261400	2264380	-
PAXXG049120	C2H2	Chr07	scaffold19	1365200	1365904	+
PAXXG051240	C2H2	Chr07	scaffold20	2342787	2344128	-
PAXXG053930	C2H2	Chr15	scaffold22	177870	181438	-
PAXXG056420	C2H2	Chr01	scaffold23	2181209	2192918	-
PAXXG064220	C2H2	Chr01	scaffold28	586909	592102	-
PAXXG064530	C2H2	Chr01	scaffold28	1120610	1135775	+
PAXXG066570	C2H2	Chr04	scaffold29	2146355	2146953	-
PAXXG069460	C2H2	Chr03	scaffold31	1693346	1693648	+
PAXXG070960	C2H2	Chr01	scaffold32	2599538	2600032	-
PAXXG071920	C2H2	Chr02	scaffold33	1634205	1638506	-
PAXXG074070	C2H2	unmapped	scaffold34	2673963	2674589	+
PAXXG074770	C2H2	Chr11	scaffold35	795036	796941	+
PAXXG077530	C2H2	Chr10	scaffold37	678537	679249	-
PAXXG077560	C2H2	Chr10	scaffold37	701152	701718	+
PAXXG078260	C2H2	Chr10	scaffold37	1592162	1592629	-
PAXXG078270	C2H2	Chr10	scaffold37	1613486	1614816	-
PAXXG079510	C2H2	Chr06	scaffold38	585191	585789	+
PAXXG079700	C2H2	Chr06	scaffold38	834336	835789	-
PAXXG079710	C2H2	Chr06	scaffold38	839781	841369	-
PAXXG079720	C2H2	Chr06	scaffold38	844844	845989	-
PAXXG079740	C2H2	Chr06	scaffold38	855359	856510	-
PAXXG085630	C2H2	Chr18	scaffold43	429002	429788	+
PAXXG085640	C2H2	Chr18	scaffold43	432644	436478	-
PAXXG088980	C2H2	Chr04	scaffold46	448546	452107	-
PAXXG090260	C2H2	Chr04	scaffold46	2822955	2824411	-
PAXXG093820	C2H2	Chr03	scaffold50	1150922	1152127	-
PAXXG093830	C2H2	Chr03	scaffold50	1167660	1168629	-
PAXXG093840	C2H2	Chr03	scaffold50	1181345	1182622	-
PAXXG093860	C2H2	Chr03	scaffold50	1188941	1190666	-
PAXXG100100	C2H2	Chr09	scaffold57	367381	367914	-
PAXXG106200	C2H2	Chr06	scaffold62	1293648	1296152	-
PAXXG114600	C2H2	Chr09	scaffold70	1601843	1603118	+
PAXXG115210	C2H2	Chr09	scaffold71	598172	610997	+
PAXXG116500	C2H2	Chr05	scaffold73	201899	203072	-
PAXXG116680	C2H2	Chr05	scaffold73	508945	514020	+
PAXXG122540	C2H2	Chr07*	scaffold79	1414279	1416957	-
PAXXG124640	C2H2	Chr04	scaffold82	215662	231143	+
PAXXG130780	C2H2	Chr11	scaffold89	189033	189875	-
PAXXG134020	C2H2	Chr03	scaffold92	1293836	1294963	+
PAXXG135030	C2H2	Chr16	scaffold93	1735769	1749573	+
PAXXG138610	C2H2	Chr17	scaffold98	657125	657742	-
PAXXG139700	C2H2	Chr17	scaffold98	2409569	2410195	-
PAXXG140160	C2H2	Chr17	scaffold98	2945867	2946436	+
PAXXG154050	C2H2	Chr10	scaffold119	839458	854478	-

PAXXG157710	C2H2	unmapped	scaffold124	162947	165179	+
PAXXG162260	C2H2	Chr01	scaffold130	709028	711467	+
PAXXG166570	C2H2	Chr05	scaffold137	961092	962446	+
PAXXG171780	C2H2	Chr07	scaffold147	90474	91263	+
PAXXG175170	C2H2	Chr04	scaffold152	639982	674788	+
PAXXG177540	C2H2	unmapped	scaffold157	1300300	1325417	+
PAXXG178800	C2H2	Chr09	scaffold160	983517	984540	+
PAXXG178860	C2H2	Chr09	scaffold160	1161542	1162679	-
PAXXG179530	C2H2	Chr07	scaffold162	97698	100301	-
PAXXG183120	C2H2	Chr02	scaffold167	1051036	1052223	+
PAXXG187830	C2H2	Chr07	scaffold176	849399	855734	-
PAXXG187920	C2H2	Chr07	scaffold176	1067433	1069002	+
PAXXG200070	C2H2	Chr19	scaffold201	146996	147614	+
PAXXG200970	C2H2	Chr12	scaffold203	306812	309653	+
PAXXG212370	C2H2	Chr11	scaffold232	266584	267444	+
PAXXG214330	C2H2	unmapped	scaffold238	471458	562039	-
PAXXG220610	C2H2	Chr02	scaffold254	733947	737743	+
PAXXG223030	C2H2	Chr06	scaffold260	276704	279668	-
PAXXG224020	C2H2	unmapped	scaffold263	187116	189861	+
PAXXG230180	C2H2	unmapped	scaffold279	1452466	1453309	-
PAXXG230220	C2H2	Chr14	scaffold280	123153	124432	+
PAXXG231320	C2H2	unmapped	scaffold284	414631	415188	+
PAXXG231560	C2H2	unmapped	scaffold284	812568	813451	-
PAXXG231590	C2H2	unmapped	scaffold284	826294	832142	+
PAXXG231610	C2H2	unmapped	scaffold284	842091	842924	+
PAXXG246300	C2H2	Chr10	scaffold328	594455	596158	+
PAXXG246690	C2H2	Chr02	scaffold329	726468	727378	-
PAXXG250450	C2H2	unmapped	scaffold342	153885	156255	-
PAXXG264560	C2H2	unmapped	scaffold393	396605	398651	-
PAXXG270280	C2H2	Chr18	scaffold420	154899	164261	+
PAXXG271790	C2H2	Chr01	scaffold426	533293	536598	+
PAXXG300280	C2H2	Chr07	scaffold579	110849	131453	-
PAXXG326900	C2H2	Chr15	scaffold792	34271	46100	-
PAXXG331700	C2H2	Chr10	scaffold845	42637	44887	+
PAXXG342330	C2H2	Chr10	scaffold993	26819	85692	-
PAXXG355840	C2H2	Chr03	scaffold1330	14072	16097	+
PAXXG368070	C2H2	unmapped	scaffold1995	6149	33804	+
PAXXG371380	C2H2	unmapped	scaffold2364	27967	33677	+
PAXXG001310	C3H	Chr02	scaffold1	2672478	2691047	-
PAXXG010490	C3H	Chr08	scaffold3	2990861	3014810	-
PAXXG012480	C3H	Chr08	scaffold3	7074855	7095689	+
PAXXG018940	C3H	Chr18	scaffold5	3994472	4026729	+
PAXXG025860	C3H	Chr13	scaffold9	394873	439186	-
PAXXG026280	C3H	Chr13	scaffold9	1262993	1344038	-
PAXXG032370	C3H	Chr10	scaffold11	3868198	3870658	+
PAXXG038830	C3H	Chr15	scaffold14	2856470	2877631	-
PAXXG050070	C3H	Chr07	scaffold20	340023	344102	+
PAXXG056810	C3H	Chr01	scaffold23	2984295	3030480	+

PAXXG058990	C3H	Chr04	scaffold25	371632	376389	+
PAXXG067680	C3H	Chr12	scaffold30	790160	838228	+
PAXXG067685	C3H	Chr12	scaffold30	839555	858643	+
PAXXG069120	C3H	Chr03	scaffold31	1008947	1026529	-
PAXXG079980	C3H	Chr06	scaffold38	1205188	1232597	-
PAXXG085000	C3H	Chr03	scaffold42	1074476	1108172	+
PAXXG100460	C3H	Chr09	scaffold57	1165426	1178543	+
PAXXG103380	C3H	Chr12	scaffold59	1019888	1022807	+
PAXXG110940	C3H	Chr14*	scaffold67	1540992	1543780	-
PAXXG118750	C3H	Chr10	scaffold75	1225420	1226080	+
PAXXG120240	C3H	Chr11	scaffold76	1805771	1809699	+
PAXXG134540	C3H	Chr16	scaffold93	387841	410396	-
PAXXG136810	C3H	Chr03	scaffold96	298335	320724	+
PAXXG137400	C3H	Chr03	scaffold96	1423512	1441297	-
PAXXG137640	C3H	Chr04	scaffold97	136206	143166	-
PAXXG145340	C3H	Chr11	scaffold105	1108790	1126423	-
PAXXG145630	C3H	Chr11	scaffold105	1499107	1529714	-
PAXXG146120	C3H	Chr13	scaffold106	856660	887423	-
PAXXG158310	C3H	Chr10	scaffold125	340902	361085	+
PAXXG159380	C3H	Chr13	scaffold126	408967	415724	-
PAXXG172920	C3H	Chr02	scaffold148	1261974	1291149	-
PAXXG179840	C3H	Chr07	scaffold162	530433	534012	-
PAXXG184790	C3H	unmapped	scaffold170	998461	999644	+
PAXXG217770	C3H	unmapped	scaffold247	649078	690987	+
PAXXG218090	C3H	unmapped	scaffold248	646960	670148	-
PAXXG219300	C3H	unmapped	scaffold252	576732	594651	+
PAXXG222010	C3H	Chr16	scaffold257	893943	896429	+
PAXXG225390	C3H	unmapped	scaffold266	813475	839831	-
PAXXG226160	C3H	Chr08	scaffold269	263585	264890	-
PAXXG231500	C3H	unmapped	scaffold284	697263	710786	-
PAXXG233110	C3H	Chr02	scaffold289	537084	554943	-
PAXXG242370	C3H	unmapped	scaffold318	308826	310778	-
PAXXG246360	C3H	Chr10	scaffold328	685715	688127	+
PAXXG260130	C3H	Chr10	scaffold375	436856	516223	-
PAXXG297590	C3H	Chr06	scaffold564	30161	53996	-
PAXXG310820	C3H	unmapped	scaffold647	121783	124137	-
PAXXG316120	C3H	Chr07	scaffold693	155064	178595	-
PAXXG325600	C3H	unmapped	scaffold777	240091	240309	-
PAXXG357590	C3H	Chr06	scaffold1393	34700	36087	-
PAXXG363230	C3H	unmapped	scaffold1662	62885	63133	+
PAXXG077440	CAMTA	Chr10	scaffold37	496542	620925	-
PAXXG111100	CAMTA	Chr14*	scaffold67	1741332	1768866	+
PAXXG200200	CAMTA	Chr19	scaffold201	361592	460033	-
PAXXG266800	CAMTA	unmapped	scaffold404	107165	248724	-
PAXXG298720	CAMTA	Chr15	scaffold571	11635	44723	-
PAXXG317170	CAMTA	Chr12	scaffold702	208587	289662	-
PAXXG025130	CPP	Chr11	scaffold8	3423014	3449779	-
PAXXG075300	CPP	Chr11	scaffold35	1501701	1550645	-

PAXXG158520	CPP	Chr10	scaffold125	584943	616775	-
PAXXG264660	CPP	unmapped	scaffold393	627282	640088	-
PAXXG013540	CSD	Chr15	scaffold4	1663924	1677831	-
PAXXG029030	CSD	Chr14	scaffold10	1730965	1731969	-
PAXXG178080	CSD	Chr19	scaffold159	95478	96854	-
PAXXG193050	CSD	Chr13	scaffold188	43659	45088	-
PAXXG070680	DBB	Chr01	scaffold32	1412397	1452298	-
PAXXG115270	DBP	Chr09	scaffold71	732563	736452	-
PAXXG256190	DBP	Chr12	scaffold362	550351	589076	+
PAXXG052290	E2F-DP	Chr18	scaffold21	364404	388976	-
PAXXG057950	E2F-DP	Chr13	scaffold24	2058861	2101625	+
PAXXG063800	E2F-DP	Chr08	scaffold27	3279907	3315676	-
PAXXG158630	E2F-DP	Chr10	scaffold125	774872	798610	+
PAXXG247540	E2F-DP	unmapped	scaffold332	704328	715956	+
PAXXG280830	E2F-DP	unmapped	scaffold468	202967	242964	+
PAXXG349120	E2F-DP	Chr16	scaffold1127	132498	141790	-
PAXXG030580	EIL	Chr10	scaffold11	143064	149861	+
PAXXG086070	EIL	Chr18	scaffold43	1406708	1409739	-
PAXXG086700	EIL	Chr03	scaffold44	449151	450576	-
PAXXG102350	EIL	Chr16	scaffold58	1622412	1625799	+
PAXXG004690	FAR1	Chr10	scaffold2	2475900	2477012	+
PAXXG006810	FAR1	Chr10	scaffold2	6755407	6756745	-
PAXXG007640	FAR1	Chr10	scaffold2	8621788	8623286	-
PAXXG008860	FAR1	Chr08	scaffold3	403884	425407	-
PAXXG008930	FAR1	Chr08	scaffold3	536539	538273	-
PAXXG014100	FAR1	Chr15	scaffold4	2797489	2799542	-
PAXXG014650	FAR1	Chr15	scaffold4	3749714	3765000	+
PAXXG019810	FAR1	Chr04	scaffold6	625218	628221	+
PAXXG019890	FAR1	Chr04	scaffold6	880115	881688	+
PAXXG022670	FAR1	Chr11	scaffold7	2185970	2188866	-
PAXXG023200	FAR1	Chr11	scaffold7	3451096	3452598	-
PAXXG024520	FAR1	Chr11	scaffold8	2067837	2069660	-
PAXXG024790	FAR1	Chr11	scaffold8	2666214	2682160	-
PAXXG025000	FAR1	Chr11	scaffold8	3004899	3007591	-
PAXXG026100	FAR1	Chr13	scaffold9	910086	916952	+
PAXXG026580	FAR1	Chr13	scaffold9	1773612	1778469	-
PAXXG027580	FAR1	Chr13	scaffold9	4265345	4268568	-
PAXXG027700	FAR1	Chr13	scaffold9	4591090	4593597	-
PAXXG032950	FAR1	Chr04	scaffold12	498878	499240	+
PAXXG043460	FAR1	Chr19	scaffold16	3638574	3639603	+
PAXXG055370	FAR1	Chr15	scaffold22	3216668	3235126	+
PAXXG055880	FAR1	Chr01	scaffold23	1054741	1079630	-
PAXXG056870	FAR1	Chr01	scaffold23	3091058	3092914	-
PAXXG060590	FAR1	Chr15	scaffold26	517635	519241	+
PAXXG060950	FAR1	Chr15	scaffold26	1434449	1437053	-
PAXXG060990	FAR1	Chr15	scaffold26	1489543	1490783	+
PAXXG063880	FAR1	Chr08	scaffold27	3490943	3494785	-
PAXXG067600	FAR1	Chr12	scaffold30	554203	557224	-

PAXXG071800	FAR1	Chr02	scaffold33	1410757	1412078	-
PAXXG072560	FAR1	Chr02	scaffold33	2845537	2850753	+
PAXXG072940	FAR1	unmapped	scaffold34	481525	495051	+
PAXXG075950	FAR1	Chr11	scaffold35	2649833	2651093	-
PAXXG075980	FAR1	Chr11	scaffold35	2671815	2672989	+
PAXXG076460	FAR1	Chr02	scaffold36	873636	876423	-
PAXXG079040	FAR1	Chr10	scaffold37	2706961	2721522	-
PAXXG088300	FAR1	Chr06	scaffold45	1547243	1548097	+
PAXXG094590	FAR1	Chr09	scaffold51	581093	584485	-
PAXXG094690	FAR1	Chr09	scaffold51	834469	835853	-
PAXXG095400	FAR1	Chr09	scaffold51	2402839	2403997	-
PAXXG095750	FAR1	Chr02	scaffold52	839863	864350	-
PAXXG097730	FAR1	Chr04	scaffold54	1844545	1845576	-
PAXXG098630	FAR1	Chr01	scaffold55	1709788	1712440	+
PAXXG098910	FAR1	Chr03	scaffold56	46861	50567	-
PAXXG101390	FAR1	Chr16	scaffold58	147831	149610	-
PAXXG104360	FAR1	Chr18	scaffold60	1228960	1230037	+
PAXXG105750	FAR1	Chr06	scaffold62	95971	97500	+
PAXXG107330	FAR1	unmapped	scaffold63	1295976	1308423	+
PAXXG107370	FAR1	unmapped	scaffold63	1478675	1487404	+
PAXXG108690	FAR1	Chr17	scaffold65	803347	824694	-
PAXXG110420	FAR1	Chr14*	scaffold67	765774	774579	-
PAXXG122140	FAR1	Chr07*	scaffold79	859052	859820	-
PAXXG126170	FAR1	Chr16	scaffold83	1285886	1286961	+
PAXXG129310	FAR1	Chr02	scaffold87	693761	714119	-
PAXXG130630	FAR1	unmapped	scaffold88	1771527	1772991	-
PAXXG136210	FAR1	Chr06	scaffold95	923258	929116	+
PAXXG141240	FAR1	Chr02	scaffold100	455836	461882	+
PAXXG143160	FAR1	unmapped	scaffold102	1523602	1526248	+
PAXXG143400	FAR1	Chr11	scaffold103	598801	599204	+
PAXXG143880	FAR1	unmapped	scaffold104	149545	150125	-
PAXXG143920	FAR1	unmapped	scaffold104	220439	221749	+
PAXXG145960	FAR1	Chr13	scaffold106	457296	458918	-
PAXXG147850	FAR1	unmapped	scaffold109	550842	552175	+
PAXXG152160	FAR1	unmapped	scaffold116	1152503	1153783	+
PAXXG160210	FAR1	unmapped	scaffold127	245840	247679	-
PAXXG160360	FAR1	unmapped	scaffold127	565573	579864	+
PAXXG160900	FAR1	Chr10	scaffold128	474203	475204	-
PAXXG160930	FAR1	Chr10	scaffold128	487459	488612	+
PAXXG164320	FAR1	unmapped	scaffold133	999952	1017171	+
PAXXG164800	FAR1	Chr10	scaffold134	793751	795005	+
PAXXG165900	FAR1	unmapped	scaffold136	1093757	1096021	+
PAXXG167220	FAR1	Chr16	scaffold138	1102383	1103105	+
PAXXG168690	FAR1	Chr10	scaffold141	868315	979651	-
PAXXG169390	FAR1	Chr15	scaffold142	1145767	1147245	-
PAXXG169960	FAR1	unmapped	scaffold143	1127597	1130324	+
PAXXG170930	FAR1	Chr18	scaffold145	480589	481810	-
PAXXG171430	FAR1	unmapped	scaffold146	136535	139293	-

PAXXG171610	FAR1	unmapped	scaffold146	982016	984507	+
PAXXG173790	FAR1	Chr09	scaffold150	363367	404030	-
PAXXG174400	FAR1	Chr19	scaffold151	201590	203102	-
PAXXG175270	FAR1	Chr04	scaffold152	931994	940294	-
PAXXG180170	FAR1	Chr07	scaffold162	1215746	1217305	-
PAXXG180180	FAR1	Chr07	scaffold162	1242078	1243613	-
PAXXG189000	FAR1	Chr10	scaffold179	650555	652633	-
PAXXG189060	FAR1	Chr10	scaffold179	808194	844545	+
PAXXG189280	FAR1	Chr15	scaffold180	49779	50692	+
PAXXG189350	FAR1	Chr15	scaffold180	243096	253170	+
PAXXG190190	FAR1	Chr07	scaffold181	1154925	1162696	+
PAXXG190510	FAR1	Chr09	scaffold182	860984	862693	+
PAXXG190580	FAR1	Chr09	scaffold182	1013658	1015319	+
PAXXG193330	FAR1	Chr13	scaffold188	464832	467824	-
PAXXG196960	FAR1	Chr13	scaffold194	1027359	1028883	+
PAXXG199250	FAR1	Chr18	scaffold200	21633	37632	+
PAXXG206610	FAR1	Chr01	scaffold216	845669	846647	-
PAXXG209600	FAR1	unmapped	scaffold223	620311	622678	-
PAXXG212730	FAR1	unmapped	scaffold233	240146	241529	+
PAXXG214020	FAR1	Chr13	scaffold237	667325	670762	-
PAXXG215910	FAR1	unmapped	scaffold242	480272	480663	-
PAXXG219190	FAR1	unmapped	scaffold252	248055	267745	-
PAXXG221420	FAR1	unmapped	scaffold256	397333	409032	+
PAXXG221500	FAR1	unmapped	scaffold256	629741	631098	+
PAXXG223360	FAR1	Chr06	scaffold260	850724	861178	+
PAXXG227740	FAR1	Chr06	scaffold272	677459	682517	+
PAXXG231890	FAR1	unmapped	scaffold285	852121	853863	+
PAXXG233130	FAR1	Chr02	scaffold289	562859	584600	-
PAXXG233580	FAR1	Chr01	scaffold291	141649	142218	+
PAXXG238230	FAR1	Chr15	scaffold305	543335	545890	-
PAXXG239950	FAR1	Chr18	scaffold311	209664	210665	+
PAXXG241270	FAR1	Chr09	scaffold314	362316	363646	+
PAXXG241810	FAR1	unmapped	scaffold316	797278	801451	+
PAXXG242750	FAR1	unmapped	scaffold319	512942	526392	+
PAXXG246590	FAR1	Chr02	scaffold329	362521	364019	+
PAXXG249090	FAR1	Chr13	scaffold337	725609	726471	+
PAXXG252820	FAR1	Chr12	scaffold350	416087	424200	+
PAXXG254750	FAR1	unmapped	scaffold358	226470	229487	-
PAXXG256250	FAR1	Chr12	scaffold362	679556	681006	+
PAXXG258700	FAR1	unmapped	scaffold370	475424	497641	+
PAXXG259650	FAR1	Chr10	scaffold374	165528	175284	+
PAXXG263530	FAR1	unmapped	scaffold389	410517	419202	-
PAXXG265490	FAR1	unmapped	scaffold396	465583	466757	-
PAXXG266570	FAR1	Chr02	scaffold403	480866	481893	+
PAXXG266680	FAR1	Chr02	scaffold403	606981	608777	+
PAXXG266740	FAR1	unmapped	scaffold404	37922	57093	+
PAXXG266920	FAR1	unmapped	scaffold404	520253	522626	-
PAXXG267880	FAR1	Chr06	scaffold407	1091952	1092991	+

PAXXG270820	FAR1	unmapped	scaffold422	377303	397646	+
PAXXG271810	FAR1	Chr01	scaffold426	652145	653382	+
PAXXG273690	FAR1	unmapped	scaffold434	117180	121460	-
PAXXG277840	FAR1	unmapped	scaffold453	393341	434203	-
PAXXG279360	FAR1	Chr12	scaffold460	128986	130198	-
PAXXG281150	FAR1	Chr02	scaffold469	169130	174538	-
PAXXG286580	FAR1	unmapped	scaffold497	75252	77007	-
PAXXG288360	FAR1	Chr10	scaffold508	196257	198564	+
PAXXG291660	FAR1	Chr15	scaffold525	435278	443189	+
PAXXG292490	FAR1	unmapped	scaffold531	118636	119244	+
PAXXG297520	FAR1	unmapped	scaffold563	86043	87447	-
PAXXG298250	FAR1	unmapped	scaffold567	255654	256277	-
PAXXG299300	FAR1	unmapped	scaffold573	332563	334268	+
PAXXG299820	FAR1	Chr10	scaffold576	60212	66461	+
PAXXG303910	FAR1	unmapped	scaffold602	224257	249831	-
PAXXG310710	FAR1	unmapped	scaffold646	133319	136110	-
PAXXG312140	FAR1	unmapped	scaffold658	358646	363775	+
PAXXG315020	FAR1	unmapped	scaffold685	213399	214188	+
PAXXG319510	FAR1	unmapped	scaffold722	221201	223566	+
PAXXG321230	FAR1	unmapped	scaffold735	266553	267705	-
PAXXG322780	FAR1	unmapped	scaffold751	60564	63638	-
PAXXG324270	FAR1	Chr16	scaffold766	235422	236566	-
PAXXG326340	FAR1	unmapped	scaffold783	337593	341498	-
PAXXG328850	FAR1	unmapped	scaffold810	141946	143745	+
PAXXG329250	FAR1	Chr07	scaffold814	16947	17447	-
PAXXG329530	FAR1	unmapped	scaffold817	248207	249789	+
PAXXG331980	FAR1	unmapped	scaffold848	60093	69427	-
PAXXG332280	FAR1	unmapped	scaffold851	20667	26336	+
PAXXG334970	FAR1	unmapped	scaffold881	218978	231274	-
PAXXG336230	FAR1	unmapped	scaffold900	94734	96079	+
PAXXG337960	FAR1	Chr11	scaffold926	170749	172050	-
PAXXG338010	FAR1	Chr10	scaffold927	189471	189884	-
PAXXG340720	FAR1	Chr14	scaffold964	178002	181730	+
PAXXG342130	FAR1	unmapped	scaffold989	181289	182438	+
PAXXG342940	FAR1	Chr01	scaffold1001	160275	162174	+
PAXXG347200	FAR1	unmapped	scaffold1085	104681	106705	+
PAXXG351720	FAR1	unmapped	scaffold1186	102849	108589	+
PAXXG357550	FAR1	unmapped	scaffold1392	28994	52952	+
PAXXG357560	FAR1	unmapped	scaffold1392	71936	78730	-
PAXXG360100	FAR1	unmapped	scaffold1495	31957	35136	-
PAXXG361030	FAR1	Chr14	scaffold1541	65163	66428	+
PAXXG361630	FAR1	unmapped	scaffold1570	20281	22457	-
PAXXG363210	FAR1	unmapped	scaffold1662	14530	15646	+
PAXXG365430	FAR1	unmapped	scaffold1808	14086	15809	-
PAXXG365700	FAR1	unmapped	scaffold1821	2573	5132	+
PAXXG366620	FAR1	unmapped	scaffold1878	36608	43843	-
PAXXG367630	FAR1	unmapped	scaffold1958	13869	14790	+
PAXXG367720	FAR1	unmapped	scaffold1962	34324	35798	+

PAXXG369160	FAR1	unmapped	scaffold2086	6873	19481	-
PAXXG372190	FAR1	unmapped	scaffold2470	537	3587	+
PAXXG372470	FAR1	unmapped	scaffold2501	22760	23936	-
PAXXG374240	FAR1	unmapped	scaffold2777	2041	3153	+
PAXXG374570	FAR1	unmapped	scaffold2833	5412	9614	+
PAXXG375030	FAR1	unmapped	scaffold2910	12603	24638	+
PAXXG375360	FAR1	unmapped	scaffold2961	786	2875	+
PAXXG375600	FAR1	unmapped	scaffold2986	17789	19443	+
PAXXG375730	FAR1	unmapped	scaffold3017	12966	14631	+
PAXXG376140	FAR1	unmapped	scaffold3092	5635	8275	-
PAXXG376230	FAR1	unmapped	scaffold3108	8277	11007	+
PAXXG376400	FAR1	unmapped	scaffold3131	4946	6987	+
PAXXG376410	FAR1	unmapped	scaffold3135	7655	9881	+
PAXXG376950	FAR1	unmapped	scaffold3228	978	3281	+
PAXXG377010	FAR1	unmapped	scaffold3235	6787	8492	-
PAXXG377450	FAR1	unmapped	scaffold3310	8270	10738	-
PAXXG377770	FAR1	unmapped	scaffold3389	128	4067	+
PAXXG378150	FAR1	unmapped	scaffold3477	16152	17182	+
PAXXG378470	FAR1	unmapped	scaffold3570	525	955	-
PAXXG379320	FAR1	unmapped	scaffold3825	5420	6768	+
PAXXG379400	FAR1	unmapped	scaffold3840	1607	13499	+
PAXXG381010	FAR1	unmapped	scaffold4338	407	2105	+
PAXXG381460	FAR1	unmapped	scaffold4445	9	1425	-
PAXXG381580	FAR1	unmapped	scaffold4502	7517	10429	-
PAXXG381750	FAR1	unmapped	scaffold4543	9353	9661	+
PAXXG381990	FAR1	unmapped	scaffold4625	3279	5213	+
PAXXG382090	FAR1	unmapped	scaffold4653	7576	8499	-
PAXXG382200	FAR1	unmapped	scaffold4693	217	1243	+
PAXXG382510	FAR1	unmapped	scaffold4768	797	2353	+
PAXXG383460	FAR1	unmapped	scaffold5042	417	1150	+
PAXXG383810	FAR1	unmapped	scaffold5151	10	1044	+
PAXXG383980	FAR1	unmapped	scaffold5215	3952	5716	-
PAXXG384090	FAR1	unmapped	scaffold5261	4297	6614	-
PAXXG384130	FAR1	unmapped	scaffold5270	1625	3471	+
PAXXG384250	FAR1	unmapped	scaffold5315	849	2803	+
PAXXG384600	FAR1	unmapped	scaffold5503	4320	5749	+
PAXXG386580	FAR1	unmapped	scaffold6552	2158	3900	-
PAXXG386750	FAR1	unmapped	scaffold6676	450	779	+
PAXXG387320	FAR1	unmapped	scaffold7059	250	1661	-
PAXXG387530	FAR1	unmapped	scaffold7245	376	996	+
PAXXG388390	FAR1	unmapped	scaffold8190	409	1605	+
PAXXG388450	FAR1	unmapped	scaffold8268	71	1372	-
PAXXG389570	FAR1	unmapped	scaffold9925	179	1048	+
PAXXG390120	FAR1	unmapped	scaffold11086	425	883	+
PAXXG000890	GARP-ARR-B	Chr02	scaffold1	1496152	1505248	-
PAXXG017500	GARP-ARR-B	Chr18	scaffold5	1153582	1166668	+
PAXXG018250	GARP-ARR-B	Chr18	scaffold5	2642555	2656437	+
PAXXG044400	GARP-ARR-B	Chr12*	scaffold17	1697122	1712016	+

PAXXG054830	GARP-ARR-B	Chr15	scaffold22	2093988	2099382	-
PAXXG333060	GARP-ARR-B	Chr15	scaffold860	214712	244768	-
PAXXG350420	GARP-ARR-B	unmapped	scaffold1155	39579	50686	-
PAXXG369650	GARP-ARR-B	unmapped	scaffold2138	4758	25124	+
PAXXG002800	GARP-G2-like	Chr02	scaffold1	5870330	5884455	-
PAXXG005030	GARP-G2-like	Chr10	scaffold2	2929694	2932026	+
PAXXG014900	GARP-G2-like	Chr15	scaffold4	4121562	4123712	+
PAXXG015120	GARP-G2-like	Chr15	scaffold4	4498872	4507658	-
PAXXG021940	GARP-G2-like	Chr11	scaffold7	536123	538925	-
PAXXG024810	GARP-G2-like	Chr11	scaffold8	2724010	2726554	-
PAXXG025610	GARP-G2-like	Chr11	scaffold8	4706072	4751000	-
PAXXG030670	GARP-G2-like	Chr10	scaffold11	274071	278365	+
PAXXG036760	GARP-G2-like	Chr16	scaffold13	3200392	3202150	+
PAXXG037190	GARP-G2-like	Chr16	scaffold13	3999477	4002825	+
PAXXG040200	GARP-G2-like	Chr07	scaffold15	1180362	1194990	+
PAXXG047280	GARP-G2-like	Chr17	scaffold18	3001740	3003323	-
PAXXG064210	GARP-G2-like	Chr01	scaffold28	548002	550227	-
PAXXG065900	GARP-G2-like	Chr04	scaffold29	965275	971299	-
PAXXG067040	GARP-G2-like	Chr04	scaffold29	2934037	2935347	+
PAXXG078130	GARP-G2-like	Chr10	scaffold37	1436709	1437887	+
PAXXG102370	GARP-G2-like	Chr16	scaffold58	1645105	1671386	+
PAXXG103790	GARP-G2-like	Chr12	scaffold59	1862945	1866149	-
PAXXG115510	GARP-G2-like	Chr09	scaffold71	1079649	1095078	+
PAXXG115990	GARP-G2-like	Chr09	scaffold71	1986463	2000893	-
PAXXG120820	GARP-G2-like	Chr09	scaffold77	1202191	1214876	+
PAXXG139220	GARP-G2-like	Chr17	scaffold98	1639575	1643941	-
PAXXG156670	GARP-G2-like	Chr09	scaffold122	686728	691786	+
PAXXG158330	GARP-G2-like	Chr10	scaffold125	380851	391721	-
PAXXG164360	GARP-G2-like	unmapped	scaffold133	1189570	1206227	+
PAXXG176340	GARP-G2-like	Chr11	scaffold155	52870	79126	-
PAXXG185500	GARP-G2-like	Chr08	scaffold172	362377	363611	-
PAXXG189050	GARP-G2-like	Chr10	scaffold179	789092	808264	-
PAXXG200390	GARP-G2-like	Chr19	scaffold201	668100	694605	-
PAXXG207030	GARP-G2-like	unmapped	scaffold217	733393	744740	+
PAXXG242780	GARP-G2-like	unmapped	scaffold319	707825	714010	+
PAXXG242920	GARP-G2-like	Chr12	scaffold320	303357	312070	+
PAXXG265280	GARP-G2-like	unmapped	scaffold396	94793	97014	+
PAXXG272150	GARP-G2-like	Chr01	scaffold427	443177	445225	+
PAXXG277570	GARP-G2-like	unmapped	scaffold451	524707	539794	+
PAXXG297400	GARP-G2-like	unmapped	scaffold562	19999	33161	+
PAXXG306150	GARP-G2-like	unmapped	scaffold616	339091	343430	-
PAXXG321120	GARP-G2-like	unmapped	scaffold735	54493	56192	-
PAXXG338840	GARP-G2-like	Chr06	scaffold937	27718	31500	-
PAXXG341590	GARP-G2-like	unmapped	scaffold981	12970	38137	+
PAXXG052580	GeBP	Chr18	scaffold21	1017634	1018993	+
PAXXG071225	GeBP	Chr01	scaffold32	3169556	3169848	-
PAXXG130890	GeBP	Chr11	scaffold89	423771	426990	-
PAXXG145820	GeBP	Chr13	scaffold106	208172	209805	+

PAXXG221250	GeBP	unmapped	scaffold256	7785	9327	+
PAXXG289600	GeBP	unmapped	scaffold515	352700	354752	+
PAXXG010750	GRAS	Chr08	scaffold3	3392809	3395246	-
PAXXG010900	GRAS	Chr08	scaffold3	3775816	3777380	-
PAXXG010910	GRAS	Chr08	scaffold3	3806759	3808250	-
PAXXG013680	GRAS	Chr15	scaffold4	2091096	2096790	+
PAXXG024980	GRAS	Chr11	scaffold8	2985738	2987436	-
PAXXG025530	GRAS	Chr11	scaffold8	4490751	4491920	+
PAXXG028580	GRAS	Chr14	scaffold10	997882	1005422	+
PAXXG035580	GRAS	Chr16	scaffold13	779518	810077	-
PAXXG038030	GRAS	Chr15	scaffold14	1186137	1187571	+
PAXXG048650	GRAS	Chr07	scaffold19	235688	237109	+
PAXXG050250	GRAS	Chr07	scaffold20	686362	688716	-
PAXXG050900	GRAS	Chr07	scaffold20	1812826	1814145	+
PAXXG056080	GRAS	Chr01	scaffold23	1419249	1420877	-
PAXXG056990	GRAS	Chr01	scaffold23	3385026	3386936	-
PAXXG066950	GRAS	Chr04	scaffold29	2780326	2784659	+
PAXXG074200	GRAS	unmapped	scaffold34	2894163	2896790	-
PAXXG076420	GRAS	Chr02	scaffold36	771918	774779	+
PAXXG077010	GRAS	Chr02	scaffold36	2617352	2619832	+
PAXXG079370	GRAS	Chr06	scaffold38	407156	408902	+
PAXXG081470	GRAS	Chr04	scaffold39	1144357	1146501	-
PAXXG082510	GRAS	Chr03	scaffold40	636870	639039	-
PAXXG084140	GRAS	Chr18	scaffold41	1755335	1758116	+
PAXXG092620	GRAS	Chr15	scaffold49	966876	968201	-
PAXXG092630	GRAS	Chr15	scaffold49	1067463	1068848	+
PAXXG092940	GRAS	Chr15	scaffold49	1819256	1821955	+
PAXXG093750	GRAS	Chr03	scaffold50	944353	947313	+
PAXXG107730	GRAS	Chr03	scaffold64	214348	215881	-
PAXXG114650	GRAS	Chr09	scaffold70	1704814	1707146	-
PAXXG117030	GRAS	Chr05	scaffold73	1020619	1022716	+
PAXXG140600	GRAS	Chr03	scaffold99	782495	785394	-
PAXXG144560	GRAS	unmapped	scaffold104	1537438	1538784	-
PAXXG154650	GRAS	unmapped	scaffold120	365021	366864	-
PAXXG157730	GRAS	unmapped	scaffold124	192551	194530	-
PAXXG166330	GRAS	Chr05	scaffold137	663591	665397	+
PAXXG166420	GRAS	Chr05	scaffold137	784097	786118	+
PAXXG166610	GRAS	Chr05	scaffold137	1061210	1063543	+
PAXXG179640	GRAS	Chr07	scaffold162	253311	258295	+
PAXXG211450	GRAS	Chr01	scaffold230	204185	206800	-
PAXXG219980	GRAS	Chr13	scaffold253	690769	692351	+
PAXXG239790	GRAS	Chr18	scaffold311	64470	65939	-
PAXXG279080	GRAS	Chr07	scaffold459	52202	52444	-
PAXXG335000	GRAS	unmapped	scaffold882	176193	195131	-
PAXXG340550	GRAS	unmapped	scaffold962	149275	152166	-
PAXXG350070	GRAS	unmapped	scaffold1148	71841	74218	+
PAXXG375190	GRAS	unmapped	scaffold2932	20855	22159	+
PAXXG375200	GRAS	unmapped	scaffold2932	22539	24294	+

PAXXG376860	GRAS	unmapped	scaffold3215	13373	16264	+
PAXXG012220	GRF	Chr08	scaffold3	6452344	6453836	+
PAXXG049020	GRF	Chr07	scaffold19	1153305	1154646	+
PAXXG052010	GRF	Chr07	scaffold20	3684307	3694292	+
PAXXG082810	GRF	Chr03	scaffold40	1217682	1221532	-
PAXXG112290	GRF	Chr19	scaffold69	98439	111009	-
PAXXG115940	GRF	Chr09	scaffold71	1906115	1910863	+
PAXXG119150	GRF	Chr11	scaffold76	10518	14838	+
PAXXG172520	GRF	Chr07	scaffold147	1308758	1312489	+
PAXXG307840	GRF	unmapped	scaffold629	222620	223774	+
PAXXG007030	HB-BELL	Chr10	scaffold2	7227947	7233661	-
PAXXG014330	HB-BELL	Chr15	scaffold4	3134982	3140043	-
PAXXG017260	HB-BELL	Chr18	scaffold5	633979	642207	+
PAXXG031490	HB-BELL	Chr10	scaffold11	1997842	2001619	-
PAXXG076950	HB-BELL	Chr02	scaffold36	2433698	2437785	-
PAXXG078510	HB-BELL	Chr10	scaffold37	1953751	1957312	-
PAXXG082560	HB-BELL	Chr03	scaffold40	709295	715639	+
PAXXG101340	HB-BELL	Chr16	scaffold58	112651	117391	+
PAXXG117410	HB-BELL	Chr05	scaffold73	1857261	1862559	+
PAXXG124940	HB-BELL	Chr04	scaffold82	687212	700952	+
PAXXG202670	HB-BELL	Chr09	scaffold206	444601	491703	-
PAXXG242260	HB-BELL	unmapped	scaffold318	90117	100893	+
PAXXG256660	HB-BELL	Chr01	scaffold365	294786	299180	+
PAXXG270240	HB-BELL	Chr18	scaffold420	91050	105134	-
PAXXG000710	HB-HD-ZIP	Chr02	scaffold1	1178621	1185700	-
PAXXG010140	HB-HD-ZIP	Chr08	scaffold3	2392288	2396458	-
PAXXG010560	HB-HD-ZIP	Chr08	scaffold3	3100272	3109483	+
PAXXG011800	HB-HD-ZIP	Chr08	scaffold3	5718922	5720707	-
PAXXG012580	HB-HD-ZIP	Chr08	scaffold3	7212337	7217564	-
PAXXG013360	HB-HD-ZIP	Chr15	scaffold4	1387322	1391538	+
PAXXG015870	HB-HD-ZIP	Chr15	scaffold4	5835164	5836449	+
PAXXG030260	HB-HD-ZIP	Chr14	scaffold10	4033894	4035849	+
PAXXG034910	HB-HD-ZIP	Chr04	scaffold12	3916185	3919371	+
PAXXG051860	HB-HD-ZIP	Chr07	scaffold20	3360481	3362408	+
PAXXG070750	HB-HD-ZIP	Chr01	scaffold32	1787629	1788920	+
PAXXG083270	HB-HD-ZIP	Chr03	scaffold40	2039609	2046255	-
PAXXG091600	HB-HD-ZIP	Chr15*	scaffold48	903187	924374	+
PAXXG092210	HB-HD-ZIP	Chr15*	scaffold48	2336819	2338661	-
PAXXG094700	HB-HD-ZIP	Chr09	scaffold51	945210	950643	+
PAXXG098060	HB-HD-ZIP	Chr01	scaffold55	280087	282474	+
PAXXG110930	HB-HD-ZIP	Chr14*	scaffold67	1514806	1516392	+
PAXXG119230	HB-HD-ZIP	Chr11	scaffold76	203457	204886	+
PAXXG120220	HB-HD-ZIP	Chr11	scaffold76	1756473	1786140	-
PAXXG132150	HB-HD-ZIP	Chr11	scaffold89	2682043	2696021	-
PAXXG152850	HB-HD-ZIP	Chr10	scaffold117	1348354	1354107	+
PAXXG153120	HB-HD-ZIP	Chr03	scaffold118	500846	502547	-
PAXXG182060	HB-HD-ZIP	Chr06	scaffold166	494485	497454	-
PAXXG185760	HB-HD-ZIP	Chr08	scaffold172	717598	718936	-

PAXXG194920	HB-HD-ZIP	Chr08	scaffold191	392552	394010	+
PAXXG209270	HB-HD-ZIP	unmapped	scaffold222	872444	888098	-
PAXXG228980	HB-HD-ZIP	unmapped	scaffold276	412260	418132	-
PAXXG239110	HB-HD-ZIP	Chr01	scaffold308	507358	509480	+
PAXXG308610	HB-HD-ZIP	Chr15	scaffold634	178605	179727	+
PAXXG313180	HB-HD-ZIP	Chr18	scaffold667	117409	119528	+
PAXXG343560	HB-HD-ZIP	unmapped	scaffold1015	179495	181721	+
PAXXG003440	HB-KNOX	Chr02	scaffold1	7352477	7361044	+
PAXXG060110	HB-KNOX	Chr04	scaffold25	3073947	3099481	+
PAXXG117170	HB-KNOX	Chr05	scaffold73	1238488	1274481	-
PAXXG119250	HB-KNOX	Chr11	scaffold76	251188	267732	+
PAXXG143420	HB-KNOX	Chr11	scaffold103	685049	698973	-
PAXXG288460	HB-KNOX	Chr09	scaffold509	159229	221491	+
PAXXG339720	HB-KNOX	unmapped	scaffold949	57757	74983	+
PAXXG027460	HB-other	Chr13	scaffold9	3870567	3873285	+
PAXXG032390	HB-other	Chr10	scaffold11	3909436	3911470	-
PAXXG050420	HB-other	Chr07	scaffold20	1083675	1084738	-
PAXXG055440	HB-other	Chr15	scaffold22	3623131	3654473	-
PAXXG082220	HB-other	Chr04	scaffold39	2615287	2648072	-
PAXXG179490	HB-other	Chr07	scaffold162	49204	50588	+
PAXXG281240	HB-other	Chr02	scaffold469	286389	332566	-
PAXXG285270	HB-other	unmapped	scaffold491	91496	108987	-
PAXXG054010	HB-PHD	Chr15	scaffold22	315228	335913	+
PAXXG061900	HB-PHD	Chr15	scaffold26	3290379	3350056	-
PAXXG002120	HB-WOX	Chr02	scaffold1	4345776	4356141	+
PAXXG030700	HB-WOX	Chr10	scaffold11	338574	340099	-
PAXXG041820	HB-WOX	Chr19	scaffold16	187726	191594	-
PAXXG077270	HB-WOX	Chr10	scaffold37	223167	224460	+
PAXXG078960	HB-WOX	Chr10	scaffold37	2544900	2545928	-
PAXXG086600	HB-WOX	Chr03	scaffold44	300363	302818	+
PAXXG102000	HB-WOX	Chr16	scaffold58	1065795	1066980	-
PAXXG174940	HB-WOX	Chr19	scaffold151	1280277	1284206	-
PAXXG242140	HB-WOX	unmapped	scaffold317	778425	779745	+
PAXXG322900	HB-WOX	Chr16	scaffold752	177247	178945	-
PAXXG141220	HRT	Chr02	scaffold100	415989	431133	+
PAXXG000760	HSF	Chr02	scaffold1	1241699	1272043	-
PAXXG019830	HSF	Chr04	scaffold6	660842	663589	-
PAXXG038870	HSF	Chr15	scaffold14	2931422	2932317	-
PAXXG041450	HSF	Chr07	scaffold15	3705910	3707904	-
PAXXG042470	HSF	Chr19	scaffold16	1140335	1141896	+
PAXXG068280	HSF	Chr12	scaffold30	2388661	2390352	-
PAXXG077220	HSF	Chr10	scaffold37	151920	153540	+
PAXXG088110	HSF	Chr06	scaffold45	983952	989864	-
PAXXG102060	HSF	Chr16	scaffold58	1132468	1133819	-
PAXXG124360	HSF	Chr15	scaffold81	1741528	1742875	-
PAXXG129990	HSF	Chr02	scaffold87	1784886	1786094	+
PAXXG162710	HSF	Chr01	scaffold130	1523603	1525082	+
PAXXG182470	HSF	Chr06	scaffold166	1226916	1228949	+

PAXXG249100	HSF	Chr13	scaffold337	727507	745386	-
PAXXG255540	HSF	Chr10	scaffold360	452222	458955	+
PAXXG266200	HSF	Chr10	scaffold400	603621	604896	+
PAXXG271760	HSF	Chr01	scaffold426	212958	256199	-
PAXXG277750	HSF	unmapped	scaffold453	196220	214176	-
PAXXG307060	HSF	unmapped	scaffold623	335449	340648	+
PAXXG343620	HSF	unmapped	scaffold1018	70349	76678	+
PAXXG356860	HSF	unmapped	scaffold1359	88156	106160	+
PAXXG378880	HSF	unmapped	scaffold3662	12983	14674	-
PAXXG345890	LFY	Chr12	scaffold1060	87113	91135	-
PAXXG064570	LIM	Chr01	scaffold28	1200523	1202361	-
PAXXG073110	LIM	unmapped	scaffold34	898863	925677	+
PAXXG166470	LIM	Chr05	scaffold137	843653	845683	+
PAXXG353940	LIM	unmapped	scaffold1253	68230	80493	+
PAXXG003890	LOB	Chr10	scaffold2	117391	118079	+
PAXXG038140	LOB	Chr15	scaffold14	1369626	1371032	+
PAXXG047040	LOB	Chr17	scaffold18	2721234	2723078	-
PAXXG050890	LOB	Chr07	scaffold20	1763640	1765355	+
PAXXG065750	LOB	Chr04	scaffold29	713304	716068	+
PAXXG069940	LOB	Chr03	scaffold31	2588789	2589941	+
PAXXG075700	LOB	Chr11	scaffold35	2170644	2172175	+
PAXXG085710	LOB	Chr18	scaffold43	598668	604063	+
PAXXG100010	LOB	Chr09	scaffold57	123420	124292	+
PAXXG100020	LOB	Chr09	scaffold57	153213	156624	-
PAXXG103600	LOB	Chr12	scaffold59	1531216	1531716	-
PAXXG106130	LOB	Chr06	scaffold62	1145842	1155447	-
PAXXG119630	LOB	Chr11	scaffold76	889576	891469	+
PAXXG137130	LOB	Chr03	scaffold96	756192	758517	+
PAXXG142980	LOB	unmapped	scaffold102	1060892	1061853	-
PAXXG156010	LOB	Chr10	scaffold121	1688702	1690266	-
PAXXG163840	LOB	Chr03	scaffold132	882416	884324	+
PAXXG171730	LOB	Chr07	scaffold147	20839	22239	+
PAXXG187630	LOB	Chr07	scaffold176	450164	453037	-
PAXXG193990	LOB	Chr03	scaffold189	496278	497609	+
PAXXG201000	LOB	Chr12	scaffold203	394134	397130	-
PAXXG221930	LOB	Chr16	scaffold257	615184	616060	-
PAXXG224450	LOB	Chr03	scaffold264	363691	365392	+
PAXXG246090	LOB	Chr10	scaffold328	251350	253253	-
PAXXG272570	LOB	unmapped	scaffold429	58172	59252	+
PAXXG275410	LOB	unmapped	scaffold442	334154	334854	+
PAXXG275420	LOB	unmapped	scaffold442	363425	364402	+
PAXXG305190	LOB	Chr19	scaffold611	159021	159691	-
PAXXG310540	LOB	Chr15	scaffold645	150545	151114	-
PAXXG008810	MADS-MIKC	Chr08	scaffold3	316922	321350	+
PAXXG045840	MADS-MIKC	Chr17	scaffold18	252429	294733	+
PAXXG049240	MADS-MIKC	Chr07	scaffold19	1560628	1580957	-
PAXXG057830	MADS-MIKC	Chr13	scaffold24	1743611	1782090	+
PAXXG063360	MADS-MIKC	Chr08	scaffold27	2429065	2433039	-

PAXXG065170	MADS-MIKC	Chr01	scaffold28	2778775	2818907	+
PAXXG070630	MADS-MIKC	Chr01	scaffold32	1199993	1213235	-
PAXXG080050	MADS-MIKC	Chr06	scaffold38	1331452	1363405	+
PAXXG080090	MADS-MIKC	Chr06	scaffold38	1423895	1480121	+
PAXXG093380	MADS-MIKC	Chr03	scaffold50	66579	70348	+
PAXXG113000	MADS-MIKC	Chr19	scaffold69	1103962	1118809	-
PAXXG116810	MADS-MIKC	Chr05	scaffold73	709533	739471	+
PAXXG116820	MADS-MIKC	Chr05	scaffold73	755655	784975	+
PAXXG130000	MADS-MIKC	Chr02	scaffold87	1798895	1814924	-
PAXXG182380	MADS-MIKC	Chr06	scaffold166	971555	983826	-
PAXXG193450	MADS-MIKC	Chr13	scaffold188	666003	671529	+
PAXXG194760	MADS-MIKC	Chr08	scaffold191	207401	235611	+
PAXXG198660	MADS-MIKC	unmapped	scaffold198	963849	1007689	+
PAXXG217950	MADS-MIKC	unmapped	scaffold248	159697	232560	-
PAXXG220840	MADS-MIKC	Chr01	scaffold255	341922	357497	+
PAXXG236780	MADS-MIKC	Chr14	scaffold300	730392	737115	+
PAXXG241380	MADS-MIKC	Chr06	scaffold315	138644	216247	+
PAXXG271330	MADS-MIKC	Chr10	scaffold424	91558	143326	+
PAXXG301780	MADS-MIKC	Chr19	scaffold588	340509	378757	-
PAXXG323200	MADS-MIKC	unmapped	scaffold755	236916	300259	-
PAXXG349010	MADS-MIKC	Chr02	scaffold1125	123346	130996	+
PAXXG350320	MADS-MIKC	unmapped	scaffold1153	12263	95246	+
PAXXG220800	MADS-MIKC	Chr01	scaffold255	261997	264707	-
PAXXG228490	MADS-MIKC	Chr16	scaffold275	592	27933	+
PAXXG198670	MADS-MIKC	unmapped	scaffold198	1014758	1043194	+
PAXXG314420	MADS-MIKC	unmapped	scaffold680	197345	201793	-
PAXXG016180	MADS-M-type	Chr15	scaffold4	6180591	6184434	+
PAXXG026260	MADS-M-type	Chr13	scaffold9	1227897	1228425	-
PAXXG051820	MADS-M-type	Chr07	scaffold20	3273883	3275171	+
PAXXG093020	MADS-M-type	Chr15	scaffold49	2061380	2064116	+
PAXXG101820	MADS-M-type	Chr16	scaffold58	888784	890535	-
PAXXG113810	MADS-M-type	Chr09	scaffold70	428117	428925	-
PAXXG123370	MADS-M-type	Chr01	scaffold80	1340350	1378833	+
PAXXG126305	MADS-M-type	Chr16	scaffold83	1780700	1780990	-
PAXXG152330	MADS-M-type	Chr10	scaffold117	108741	113022	+
PAXXG152470	MADS-M-type	Chr10	scaffold117	414456	416157	-
PAXXG152520	MADS-M-type	Chr10	scaffold117	538018	538611	-
PAXXG185550	MADS-M-type	Chr08	scaffold172	396110	406746	+
PAXXG187720	MADS-M-type	Chr07	scaffold176	599658	600064	+
PAXXG201910	MADS-M-type	unmapped	scaffold204	1021991	1023722	+
PAXXG220830	MADS-M-type	Chr01	scaffold255	339615	340168	+
PAXXG289580	MADS-M-type	unmapped	scaffold515	215687	216433	-
PAXXG289610	MADS-M-type	unmapped	scaffold515	410038	410691	+
PAXXG328320	MADS-M-type	unmapped	scaffold805	4500	5033	-
PAXXG335905	MADS-M-type	Chr12	scaffold896	40643	41181	-
PAXXG335930	MADS-M-type	Chr12	scaffold896	82268	82714	-
PAXXG335935	MADS-M-type	Chr12	scaffold896	103637	104083	-
PAXXG335970	MADS-M-type	Chr12	scaffold896	124533	125209	-

PAXXG348560	MADS-M-type	unmapped	scaffold1116	96180	123773	+
PAXXG351395	MADS-M-type	unmapped	scaffold1178	140360	141227	+
PAXXG374144	MADS-M-type	unmapped	scaffold2758	8690	8995	+
PAXXG000780	MYB	Chr02	scaffold1	1330343	1332356	+
PAXXG005570	MYB	Chr10	scaffold2	4030368	4072654	-
PAXXG006110	MYB	Chr10	scaffold2	5212055	5214221	-
PAXXG012280	MYB	Chr08	scaffold3	6578262	6595729	+
PAXXG012650	MYB	Chr08	scaffold3	7314197	7315880	+
PAXXG016050	MYB	Chr15	scaffold4	6040700	6043395	+
PAXXG016480	MYB	Chr15	scaffold4	6596048	6600494	-
PAXXG016570	MYB	Chr15	scaffold4	6706800	6708673	-
PAXXG018180	MYB	Chr18	scaffold5	2489607	2491538	-
PAXXG019490	MYB	Chr18	scaffold5	5329921	5331386	-
PAXXG020210	MYB	Chr04	scaffold6	1633561	1673532	+
PAXXG029600	MYB	Chr14	scaffold10	2749067	2751156	-
PAXXG029670	MYB	Chr14	scaffold10	3026428	3028446	+
PAXXG030620	MYB	Chr10	scaffold11	221490	222497	+
PAXXG030750	MYB	Chr10	scaffold11	418310	419870	+
PAXXG032580	MYB	Chr10	scaffold11	4399856	4401536	-
PAXXG032710	MYB	Chr04	scaffold12	191976	193573	+
PAXXG034640	MYB	Chr04	scaffold12	3366043	3414554	-
PAXXG035040	MYB	Chr04	scaffold12	4198826	4202115	-
PAXXG035110	MYB	Chr04	scaffold12	4363053	4368118	-
PAXXG035800	MYB	Chr16	scaffold13	1447968	1450001	-
PAXXG036460	MYB	Chr16	scaffold13	2582326	2583189	-
PAXXG037080	MYB	Chr16	scaffold13	3726528	3729661	+
PAXXG039520	MYB	Chr15	scaffold14	4109097	4110617	-
PAXXG039790	MYB	Chr07	scaffold15	425773	427453	-
PAXXG039810	MYB	Chr07	scaffold15	436298	437659	-
PAXXG043380	MYB	Chr19	scaffold16	3320442	3323643	-
PAXXG043940	MYB	Chr12*	scaffold17	773555	776552	+
PAXXG045780	MYB	Chr17	scaffold18	100837	140300	-
PAXXG049360	MYB	Chr07	scaffold19	1874559	1876245	-
PAXXG050100	MYB	Chr07	scaffold20	384790	386390	-
PAXXG051890	MYB	Chr07	scaffold20	3425136	3427022	+
PAXXG052550	MYB	Chr18	scaffold21	926417	929013	-
PAXXG057720	MYB	Chr13	scaffold24	1546582	1548186	-
PAXXG058610	MYB	Chr13	scaffold24	3378285	3379512	-
PAXXG059220	MYB	Chr04	scaffold25	713005	727550	-
PAXXG060180	MYB	Chr04	scaffold25	3191014	3194694	-
PAXXG064080	MYB	Chr01	scaffold28	354777	356461	+
PAXXG065450	MYB	Chr04	scaffold29	9573	12880	-
PAXXG065970	MYB	Chr04	scaffold29	1059512	1060591	-
PAXXG066530	MYB	Chr04	scaffold29	2114420	2115322	-
PAXXG070520	MYB	Chr01	scaffold32	965163	967145	-
PAXXG071610	MYB	Chr02	scaffold33	910620	911787	+
PAXXG073450	MYB	unmapped	scaffold34	1504919	1507109	-
PAXXG076310	MYB	Chr02	scaffold36	365765	367384	+

PAXXG076770	MYB	Chr02	scaffold36	1843055	1845752	-
PAXXG077510	MYB	Chr10	scaffold37	665455	666596	-
PAXXG081530	MYB	Chr04	scaffold39	1234321	1254944	-
PAXXG086530	MYB	Chr03	scaffold44	139461	141365	-
PAXXG086650	MYB	Chr03	scaffold44	358916	361090	-
PAXXG086900	MYB	Chr03	scaffold44	830811	831449	-
PAXXG094110	MYB	Chr03	scaffold50	1823676	1824737	-
PAXXG094660	MYB	Chr09	scaffold51	787268	790096	+
PAXXG099050	MYB	Chr03	scaffold56	371473	373226	+
PAXXG100080	MYB	Chr09	scaffold57	275378	278150	+
PAXXG100085	MYB	Chr09	scaffold57	316922	318674	+
PAXXG101860	MYB	Chr16	scaffold58	918293	919414	+
PAXXG102200	MYB	Chr16	scaffold58	1371284	1379697	+
PAXXG102390	MYB	Chr16	scaffold58	1686612	1688505	-
PAXXG104200	MYB	Chr18	scaffold60	635735	637153	+
PAXXG112540	MYB	Chr19	scaffold69	407749	408912	-
PAXXG114410	MYB	Chr09	scaffold70	1210253	1212454	+
PAXXG114460	MYB	Chr09	scaffold70	1373575	1375233	+
PAXXG119190	MYB	Chr11	scaffold76	133663	136009	-
PAXXG120700	MYB	Chr09	scaffold77	925654	927439	-
PAXXG120810	MYB	Chr09	scaffold77	1124693	1126220	-
PAXXG122360	MYB	Chr07*	scaffold79	1093660	1097788	-
PAXXG123320	MYB	Chr01	scaffold80	1286371	1288520	-
PAXXG123420	MYB	Chr01	scaffold80	1556470	1558545	+
PAXXG124400	MYB	Chr15	scaffold81	1793603	1794620	-
PAXXG134010	MYB	Chr03	scaffold92	1275763	1277124	-
PAXXG139190	MYB	Chr17	scaffold98	1581593	1583424	+
PAXXG140220	MYB	Chr17	scaffold98	3016949	3036321	+
PAXXG143730	MYB	Chr11	scaffold103	1601055	1603882	+
PAXXG145640	MYB	Chr11	scaffold105	1557120	1558473	-
PAXXG156540	MYB	Chr09	scaffold122	417165	418795	-
PAXXG156600	MYB	Chr09	scaffold122	548265	549604	+
PAXXG162210	MYB	Chr01	scaffold130	453684	455593	+
PAXXG165560	MYB	unmapped	scaffold136	99060	103868	+
PAXXG173940	MYB	Chr09	scaffold150	536122	539758	-
PAXXG185380	MYB	Chr08	scaffold172	144386	147001	-
PAXXG185930	MYB	Chr08	scaffold172	936255	938574	-
PAXXG188610	MYB	Chr18	scaffold178	658465	662414	-
PAXXG199350	MYB	Chr18	scaffold200	216143	217577	+
PAXXG212220	MYB	unmapped	scaffold231	838292	839906	+
PAXXG212230	MYB	unmapped	scaffold231	844804	846065	+
PAXXG212240	MYB	unmapped	scaffold231	859375	860811	+
PAXXG216740	MYB	unmapped	scaffold244	610316	611809	+
PAXXG219600	MYB	Chr13	scaffold253	96468	105381	+
PAXXG221140	MYB	Chr01	scaffold255	744534	746386	+
PAXXG221190	MYB	Chr01	scaffold255	843495	847514	-
PAXXG223570	MYB	Chr15	scaffold261	693587	695157	-
PAXXG223900	MYB	Chr12	scaffold262	699610	727177	+

PAXXG240250	MYB	Chr18	scaffold311	659127	660859	+
PAXXG242360	MYB	unmapped	scaffold318	278530	280003	-
PAXXG243400	MYB	Chr17	scaffold321	444298	446018	-
PAXXG259710	MYB	Chr10	scaffold374	356396	357522	-
PAXXG262150	MYB	Chr13	scaffold384	353342	355369	-
PAXXG277560	MYB	unmapped	scaffold451	506316	507401	-
PAXXG284530	MYB	unmapped	scaffold486	425136	459720	+
PAXXG289520	MYB	unmapped	scaffold515	86371	89306	-
PAXXG290370	MYB	Chr01	scaffold519	422452	424167	-
PAXXG301890	MYB	unmapped	scaffold589	193265	194485	-
PAXXG304660	MYB	Chr13	scaffold608	64905	66459	-
PAXXG313160	MYB	Chr18	scaffold667	87248	88424	-
PAXXG313220	MYB	Chr18	scaffold667	163448	165574	+
PAXXG319690	MYB	Chr02	scaffold724	171238	172499	-
PAXXG329710	MYB	unmapped	scaffold820	59090	62816	-
PAXXG337540	MYB	Chr19	scaffold920	36506	38049	-
PAXXG344110	MYB	unmapped	scaffold1027	160887	162717	-
PAXXG344790	MYB	unmapped	scaffold1043	11399	12737	-
PAXXG001130	MYB-related	Chr02	scaffold1	2174976	2185628	-
PAXXG006960	MYB-related	Chr10	scaffold2	7085835	7090205	+
PAXXG007220	MYB-related	Chr10	scaffold2	7626099	7658464	-
PAXXG012300	MYB-related	Chr08	scaffold3	6615321	6643880	-
PAXXG014950	MYB-related	Chr15	scaffold4	4198503	4212427	+
PAXXG018220	MYB-related	Chr18	scaffold5	2572087	2631850	+
PAXXG023270	MYB-related	Chr11	scaffold7	3731165	3735019	+
PAXXG046480	MYB-related	Chr17	scaffold18	1645665	1659903	-
PAXXG052470	MYB-related	Chr18	scaffold21	725581	727562	+
PAXXG059920	MYB-related	Chr04	scaffold25	2659214	2666606	+
PAXXG064620	MYB-related	Chr01	scaffold28	1377194	1384000	-
PAXXG064920	MYB-related	Chr01	scaffold28	2130170	2131843	-
PAXXG065250	MYB-related	Chr01	scaffold28	2954040	3002762	-
PAXXG076710	MYB-related	Chr02	scaffold36	1649426	1666970	-
PAXXG083680	MYB-related	Chr18	scaffold41	378588	393738	+
PAXXG095220	MYB-related	Chr09	scaffold51	2180281	2197074	-
PAXXG096870	MYB-related	Chr13	scaffold53	2305445	2309074	+
PAXXG099140	MYB-related	Chr03	scaffold56	528860	529753	+
PAXXG103190	MYB-related	Chr12	scaffold59	719000	721853	+
PAXXG114290	MYB-related	Chr09	scaffold70	1015019	1022323	+
PAXXG114470	MYB-related	Chr09	scaffold70	1381262	1387887	+
PAXXG115590	MYB-related	Chr09	scaffold71	1293433	1300402	+
PAXXG121560	MYB-related	Chr12	scaffold78	1528525	1532213	-
PAXXG124020	MYB-related	Chr15	scaffold81	1025452	1039436	-
PAXXG131450	MYB-related	Chr11	scaffold89	1423772	1443619	+
PAXXG135010	MYB-related	Chr16	scaffold93	1701704	1705266	-
PAXXG144050	MYB-related	unmapped	scaffold104	489160	497095	-
PAXXG157920	MYB-related	unmapped	scaffold124	899667	900266	+
PAXXG169780	MYB-related	unmapped	scaffold143	665728	764767	+
PAXXG173430	MYB-related	Chr08	scaffold149	817928	845795	-

PAXXG178400	MYB-related	Chr19	scaffold159	1181459	1185330	+
PAXXG180290	MYB-related	Chr09	scaffold163	112784	119158	-
PAXXG183900	MYB-related	Chr06	scaffold169	551168	553231	+
PAXXG193120	MYB-related	Chr13	scaffold188	153636	190375	+
PAXXG197090	MYB-related	Chr01	scaffold195	23016	68108	+
PAXXG201320	MYB-related	Chr12	scaffold203	1015904	1017666	-
PAXXG214920	MYB-related	Chr06	scaffold240	498079	498396	-
PAXXG215670	MYB-related	unmapped	scaffold242	128593	129354	+
PAXXG219590	MYB-related	Chr13	scaffold253	94698	96054	-
PAXXG219610	MYB-related	Chr13	scaffold253	112261	116372	+
PAXXG221980	MYB-related	Chr16	scaffold257	767114	781954	-
PAXXG238720	MYB-related	Chr10	scaffold307	418611	477114	-
PAXXG238970	MYB-related	Chr01	scaffold308	263859	297126	-
PAXXG240320	MYB-related	Chr18	scaffold311	751931	752653	+
PAXXG241260	MYB-related	Chr09	scaffold314	347703	358528	+
PAXXG243490	MYB-related	Chr17	scaffold321	700043	700722	+
PAXXG271780	MYB-related	Chr01	scaffold426	438341	439604	+
PAXXG286850	MYB-related	Chr10	scaffold499	132840	145454	+
PAXXG315010	MYB-related	unmapped	scaffold685	155038	156886	-
PAXXG316820	MYB-related	unmapped	scaffold698	273878	276000	-
PAXXG317080	MYB-related	Chr17	scaffold701	222389	264356	+
PAXXG318620	MYB-related	Chr16	scaffold715	279689	282764	-
PAXXG320460	MYB-related	unmapped	scaffold729	244381	245424	+
PAXXG374220	MYB-related	unmapped	scaffold2769	17374	21358	+
PAXXG001990	NAC	Chr02	scaffold1	3904329	3906056	-
PAXXG002390	NAC	Chr02	scaffold1	5034077	5036418	-
PAXXG002890	NAC	Chr02	scaffold1	6079166	6080332	-
PAXXG002900	NAC	Chr02	scaffold1	6118091	6119548	-
PAXXG002910	NAC	Chr02	scaffold1	6128184	6129426	-
PAXXG006020	NAC	Chr10	scaffold2	4928887	4939724	-
PAXXG010170	NAC	Chr08	scaffold3	2469215	2503414	-
PAXXG016340	NAC	Chr15	scaffold4	6355924	6358488	-
PAXXG023240	NAC	Chr11	scaffold7	3623223	3625947	+
PAXXG029610	NAC	Chr14	scaffold10	2773785	2775508	+
PAXXG029620	NAC	Chr14	scaffold10	2827398	2829002	-
PAXXG030790	NAC	Chr10	scaffold11	549893	551799	-
PAXXG030810	NAC	Chr10	scaffold11	619173	621875	-
PAXXG037830	NAC	Chr15	scaffold14	872713	884074	+
PAXXG040090	NAC	Chr07	scaffold15	831057	833190	+
PAXXG040230	NAC	Chr07	scaffold15	1249090	1260881	-
PAXXG047470	NAC	Chr17	scaffold18	3213863	3215276	+
PAXXG059350	NAC	Chr04	scaffold25	1039353	1042265	-
PAXXG061850	NAC	Chr15	scaffold26	3225752	3227229	-
PAXXG071950	NAC	Chr02	scaffold33	1714086	1733127	+
PAXXG072060	NAC	Chr02	scaffold33	1932482	1934709	-
PAXXG082750	NAC	Chr03	scaffold40	1102174	1107569	-
PAXXG089550	NAC	Chr04	scaffold46	1632249	1634271	-
PAXXG095090	NAC	Chr09	scaffold51	1880261	1883920	+

PAXXG101070	NAC	Chr09	scaffold57	2183728	2192014	+
PAXXG102470	NAC	Chr16	scaffold58	1815039	1816574	-
PAXXG102480	NAC	Chr16	scaffold58	1844589	1846049	+
PAXXG103700	NAC	Chr12	scaffold59	1711521	1715797	+
PAXXG108390	NAC	Chr03	scaffold64	2047162	2049660	+
PAXXG108420	NAC	Chr03	scaffold64	2124986	2127030	+
PAXXG115710	NAC	Chr09	scaffold71	1541542	1544604	+
PAXXG119590	NAC	Chr11	scaffold76	760415	762123	+
PAXXG123380	NAC	Chr01	scaffold80	1449405	1450968	+
PAXXG137480	NAC	Chr03	scaffold96	1639883	1641278	+
PAXXG139140	NAC	Chr17	scaffold98	1512369	1514273	-
PAXXG139160	NAC	Chr17	scaffold98	1549667	1552081	+
PAXXG140380	NAC	Chr03	scaffold99	40508	41979	+
PAXXG146790	NAC	Chr02	scaffold107	1451868	1454965	-
PAXXG147790	NAC	unmapped	scaffold109	393163	399709	-
PAXXG150970	NAC	Chr09	scaffold114	854534	855027	+
PAXXG153380	NAC	Chr03	scaffold118	1098165	1100345	-
PAXXG158380	NAC	Chr10	scaffold125	428842	433465	+
PAXXG160750	NAC	unmapped	scaffold127	1447155	1448044	-
PAXXG164820	NAC	Chr10	scaffold134	825772	827823	+
PAXXG166360	NAC	Chr05	scaffold137	709457	722451	+
PAXXG169650	NAC	unmapped	scaffold143	414002	416290	-
PAXXG171030	NAC	Chr18	scaffold145	678613	680984	+
PAXXG180560	NAC	Chr09	scaffold163	1103544	1106860	-
PAXXG181060	NAC	Chr07	scaffold164	1210426	1246550	-
PAXXG183090	NAC	Chr02	scaffold167	1022433	1036912	+
PAXXG183260	NAC	unmapped	scaffold168	171648	175821	+
PAXXG185430	NAC	Chr08	scaffold172	204562	206860	-
PAXXG207940	NAC	unmapped	scaffold219	591366	593177	+
PAXXG213590	NAC	unmapped	scaffold236	687177	706792	+
PAXXG215210	NAC	Chr06	scaffold240	854997	859265	-
PAXXG220280	NAC	Chr02	scaffold254	105356	112992	+
PAXXG224230	NAC	unmapped	scaffold263	631304	633619	-
PAXXG224280	NAC	unmapped	scaffold263	692592	703730	-
PAXXG230100	NAC	unmapped	scaffold279	1145873	1147010	+
PAXXG242300	NAC	unmapped	scaffold318	149972	154492	-
PAXXG250220	NAC	unmapped	scaffold341	478562	480165	+
PAXXG252010	NAC	Chr11	scaffold347	546361	549021	-
PAXXG252030	NAC	Chr11	scaffold347	614108	615582	-
PAXXG252040	NAC	Chr11	scaffold347	699578	701318	+
PAXXG267460	NAC	unmapped	scaffold406	615519	617084	-
PAXXG267620	NAC	Chr06	scaffold407	565859	613125	-
PAXXG273990	NAC	Chr02	scaffold435	238216	239808	-
PAXXG281390	NAC	Chr10	scaffold470	72299	73416	-
PAXXG281490	NAC	Chr10	scaffold470	240833	256115	+
PAXXG290320	NAC	Chr01	scaffold519	351964	354978	+
PAXXG300950	NAC	unmapped	scaffold584	208994	210886	-
PAXXG303710	NAC	unmapped	scaffold601	303	683	-

PAXXG310350	NAC	Chr10	scaffold644	324472	326112	+
PAXXG314690	NAC	unmapped	scaffold682	82703	85583	+
PAXXG316070	NAC	Chr07	scaffold693	78512	88797	-
PAXXG317800	NAC	Chr01	scaffold708	191199	193391	-
PAXXG329470	NAC	unmapped	scaffold817	14197	17035	+
PAXXG335290	NAC	unmapped	scaffold885	159620	161179	-
PAXXG046580	NF-X1	Chr17	scaffold18	1839377	1873821	-
PAXXG223310	NF-X1	Chr06	scaffold260	792112	795891	-
PAXXG013570	NF-YA	Chr15	scaffold4	1756707	1799833	+
PAXXG049090	NF-YA	Chr07	scaffold19	1279497	1298786	+
PAXXG050040	NF-YA	Chr07	scaffold20	267800	276886	-
PAXXG139410	NF-YA	Chr17	scaffold98	2018756	2019389	+
PAXXG176040	NF-YA	Chr07	scaffold154	536471	564660	-
PAXXG241750	NF-YA	unmapped	scaffold316	563352	584277	-
PAXXG045820	NF-YB	Chr17	scaffold18	215269	216799	+
PAXXG081980	NF-YB	Chr04	scaffold39	1957866	1992029	-
PAXXG116890	NF-YB	Chr05	scaffold73	904673	905903	-
PAXXG122800	NF-YB	Chr01	scaffold80	79578	80733	+
PAXXG134445	NF-YB	Chr16	scaffold93	239332	239620	+
PAXXG134450	NF-YB	Chr16	scaffold93	244213	244650	+
PAXXG167150	NF-YB	Chr16	scaffold138	801426	802252	+
PAXXG181750	NF-YB	Chr06	scaffold166	59471	72853	+
PAXXG230790	NF-YB	Chr11	scaffold282	540010	553041	-
PAXXG355190	NF-YB	Chr03	scaffold1299	29489	44380	+
PAXXG031120	NF-YC	Chr10	scaffold11	1365866	1386363	+
PAXXG048710	NF-YC	Chr07	scaffold19	309027	324371	-
PAXXG087450	NF-YC	Chr03	scaffold44	2072461	2076624	-
PAXXG089210	NF-YC	Chr04	scaffold46	934078	935319	-
PAXXG101130	NF-YC	Chr09	scaffold57	2244242	2246255	-
PAXXG114960	NF-YC	Chr09	scaffold71	150208	150558	+
PAXXG144500	NF-YC	unmapped	scaffold104	1468280	1500202	+
PAXXG189110	NF-YC	Chr10	scaffold179	932309	933448	-
PAXXG197940	NF-YC	unmapped	scaffold197	262356	263329	-
PAXXG240220	NF-YC	Chr18	scaffold311	616796	627490	+
PAXXG271990	NF-YC	Chr01	scaffold427	179081	200289	-
PAXXG008570	OFP	Chr10	scaffold2	10307334	10308210	+
PAXXG036410	OFP	Chr16	scaffold13	2460096	2461165	-
PAXXG038320	OFP	Chr15	scaffold14	1776901	1777634	-
PAXXG045270	OFP	Chr12*	scaffold17	3542626	3543596	+
PAXXG047230	OFP	Chr17	scaffold18	2914324	2915013	-
PAXXG047520	OFP	Chr17	scaffold18	3298671	3299389	+
PAXXG050360	OFP	Chr07	scaffold20	856396	857476	+
PAXXG052250	OFP	Chr18	scaffold21	246841	250834	+
PAXXG063410	OFP	Chr08	scaffold27	2542878	2543633	+
PAXXG063750	OFP	Chr08	scaffold27	3202474	3203400	+
PAXXG063760	OFP	Chr08	scaffold27	3218487	3219588	-
PAXXG072580	OFP	Chr02	scaffold33	2941141	2942193	+
PAXXG088370	OFP	Chr06	scaffold45	1799403	1800360	+

PAXXG151070	OFP	Chr09	scaffold114	1237919	1259168	+
PAXXG193880	OFP	Chr03	scaffold189	221763	223176	-
PAXXG202690	OFP	Chr09	scaffold206	586967	587707	+
PAXXG223080	OFP	Chr06	scaffold260	374900	375801	-
PAXXG279890	OFP	Chr09	scaffold463	111549	112245	+
PAXXG295880	OFP	Chr10	scaffold552	446396	447344	-
PAXXG346370	OFP	unmapped	scaffold1067	131340	132341	-
PAXXG351490	OFP	Chr11	scaffold1180	123463	124834	+
PAXXG372980	OFP	unmapped	scaffold2571	29491	30346	+
PAXXG383170	OFP	unmapped	scaffold4959	264	1332	-
PAXXG014730	PLATZ	Chr15	scaffold4	3922971	3933012	+
PAXXG014740	PLATZ	Chr15	scaffold4	3932665	3935850	-
PAXXG014750	PLATZ	Chr15	scaffold4	3936570	3941349	-
PAXXG014760	PLATZ	Chr15	scaffold4	3949091	3950699	-
PAXXG014770	PLATZ	Chr15	scaffold4	3969926	3971721	-
PAXXG014780	PLATZ	Chr15	scaffold4	3979913	3981597	-
PAXXG014790	PLATZ	Chr15	scaffold4	3985377	3988176	-
PAXXG014800	PLATZ	Chr15	scaffold4	3992431	3994111	-
PAXXG022900	PLATZ	Chr11	scaffold7	2787545	2789204	+
PAXXG071660	PLATZ	Chr02	scaffold33	1155658	1157871	+
PAXXG088740	PLATZ	Chr06	scaffold45	2465749	2466904	-
PAXXG150550	PLATZ	Chr09	scaffold114	94122	95440	+
PAXXG162490	PLATZ	Chr01	scaffold130	1064739	1066142	-
PAXXG173590	PLATZ	Chr08	scaffold149	1082552	1085345	-
PAXXG178730	PLATZ	Chr09	scaffold160	710539	711799	+
PAXXG190300	PLATZ	Chr09	scaffold182	331637	332860	+
PAXXG230460	PLATZ	Chr14	scaffold280	812368	812864	-
PAXXG242590	PLATZ	unmapped	scaffold319	45738	46144	+
PAXXG254890	PLATZ	unmapped	scaffold358	558832	559641	+
PAXXG260680	PLATZ	unmapped	scaffold377	559023	560951	+
PAXXG363670	PLATZ	unmapped	scaffold1684	46836	47843	-
PAXXG387900	PLATZ	unmapped	scaffold7571	1061	1762	+
PAXXG002050	RWP-RK	Chr02	scaffold1	4027333	4037799	+
PAXXG049060	RWP-RK	Chr07	scaffold19	1240695	1251422	+
PAXXG057110	RWP-RK	Chr13	scaffold24	179185	215587	+
PAXXG123180	RWP-RK	Chr01	scaffold80	1069565	1074448	+
PAXXG142950	RWP-RK	unmapped	scaffold102	830587	873303	+
PAXXG186180	RWP-RK	Chr17	scaffold173	206450	210370	+
PAXXG264680	RWP-RK	unmapped	scaffold394	68352	102426	-
PAXXG081990	S1Fa-like	Chr04	scaffold39	1994614	2008202	-
PAXXG296900	SAP	Chr10	scaffold558	262327	278973	+
PAXXG018830	SBP	Chr18	scaffold5	3788482	3797017	-
PAXXG023010	SBP	Chr11	scaffold7	3000929	3006576	-
PAXXG023380	SBP	Chr11	scaffold7	3996906	4025178	-
PAXXG069710	SBP	Chr03	scaffold31	2187989	2191104	-
PAXXG088850	SBP	Chr04	scaffold46	169671	196422	+
PAXXG141350	SBP	Chr02	scaffold100	580328	635042	+
PAXXG145850	SBP	Chr13	scaffold106	271977	292007	-

PAXXG201790	SBP	unmapped	scaffold204	808699	814774	-
PAXXG219700	SBP	Chr13	scaffold253	237446	240030	+
PAXXG236750	SBP	Chr14	scaffold300	701131	707606	+
PAXXG282960	SBP	Chr01	scaffold476	208528	240345	+
PAXXG288520	SBP	Chr09	scaffold509	382265	384536	-
PAXXG288530	SBP	Chr09	scaffold509	393585	397528	-
PAXXG324040	SBP	unmapped	scaffold764	289855	295450	-
PAXXG328340	SBP	unmapped	scaffold805	45566	56327	-
PAXXG346560	SBP	unmapped	scaffold1072	84419	127182	-
PAXXG346570	SBP	unmapped	scaffold1072	135650	136684	-
PAXXG348780	SBP	unmapped	scaffold1121	31296	36138	+
PAXXG093360	SRS	Chr03	scaffold50	34910	35893	+
PAXXG114800	SRS	Chr09	scaffold70	1974564	1976204	-
PAXXG221770	SRS	Chr16	scaffold257	220964	222855	+
PAXXG241700	SRS	unmapped	scaffold316	336964	338982	+
PAXXG207960	STAT	unmapped	scaffold219	682839	698066	+
PAXXG002620	TCP	Chr02	scaffold1	5670561	5671546	-
PAXXG010770	TCP	Chr08	scaffold3	3456063	3456929	+
PAXXG030160	TCP	Chr14	scaffold10	3762063	3762951	+
PAXXG051330	TCP	Chr07	scaffold20	2472173	2474790	+
PAXXG056230	TCP	Chr01	scaffold23	1847819	1872300	-
PAXXG058310	TCP	Chr13	scaffold24	2661078	2662667	-
PAXXG059160	TCP	Chr04	scaffold25	636201	636893	+
PAXXG074530	TCP	Chr11	scaffold35	501086	503780	-
PAXXG092900	TCP	Chr15	scaffold49	1714572	1715576	-
PAXXG092910	TCP	Chr15	scaffold49	1723978	1725009	-
PAXXG110140	TCP	Chr14*	scaffold67	252263	278453	+
PAXXG116740	TCP	Chr05	scaffold73	587941	623079	+
PAXXG121830	TCP	Chr07*	scaffold79	118949	121462	+
PAXXG123030	TCP	Chr01	scaffold80	730254	731524	-
PAXXG136950	TCP	Chr03	scaffold96	519085	520346	+
PAXXG157820	TCP	unmapped	scaffold124	553171	555052	+
PAXXG174050	TCP	Chr09	scaffold150	679663	680735	-
PAXXG189640	TCP	Chr15	scaffold180	544491	545405	+
PAXXG221270	TCP	unmapped	scaffold256	22605	39771	+
PAXXG243080	TCP	Chr12	scaffold320	564104	566037	+
PAXXG297730	TCP	Chr06	scaffold564	279341	280383	+
PAXXG312310	TCP	unmapped	scaffold660	12718	14606	+
PAXXG351590	TCP	Chr16	scaffold1183	107093	108878	+
PAXXG009620	Tify	Chr08	scaffold3	1653306	1655887	-
PAXXG009920	Tify	Chr08	scaffold3	2153681	2154743	+
PAXXG013450	Tify	Chr15	scaffold4	1589741	1593871	-
PAXXG013820	Tify	Chr15	scaffold4	2318261	2320965	+
PAXXG029240	Tify	Chr14	scaffold10	2121071	2128475	+
PAXXG049350	Tify	Chr07	scaffold19	1823461	1826429	-
PAXXG055930	Tify	Chr01	scaffold23	1144504	1146333	-
PAXXG088150	Tify	Chr06	scaffold45	1191801	1198875	+
PAXXG098670	Tify	Chr01	scaffold55	1815258	1817826	+

PAXXG116340	Tify	unmapped	scaffold72	1701837	1731543	-
PAXXG123530	Tify	Chr01	scaffold80	1844708	1856938	-
PAXXG152710	Tify	Chr10	scaffold117	874687	876585	-
PAXXG209060	Tify	unmapped	scaffold222	160932	164070	-
PAXXG229230	Tify	Chr06	scaffold277	158954	162380	+
PAXXG308040	Tify	Chr11	scaffold631	88945	89549	+
PAXXG348960	Tify	unmapped	scaffold1124	118147	128047	-
PAXXG006630	Trihelix	Chr10	scaffold2	6418365	6486538	+
PAXXG008000	Trihelix	Chr10	scaffold2	9240155	9241688	+
PAXXG015960	Trihelix	Chr15	scaffold4	5962536	5963819	-
PAXXG023620	Trihelix	Chr11	scaffold7	4594274	4601596	-
PAXXG025420	Trihelix	Chr11	scaffold8	4220226	4221711	-
PAXXG027760	Trihelix	Chr14	scaffold10	38815	40369	+
PAXXG028170	Trihelix	Chr14	scaffold10	505295	510007	-
PAXXG029350	Trihelix	Chr14	scaffold10	2303668	2304800	+
PAXXG037200	Trihelix	Chr16	scaffold13	4016489	4034445	-
PAXXG047350	Trihelix	Chr17	scaffold18	3106233	3107849	-
PAXXG057880	Trihelix	Chr13	scaffold24	1872095	1876311	-
PAXXG065230	Trihelix	Chr01	scaffold28	2914917	2920058	-
PAXXG084710	Trihelix	Chr03	scaffold42	291586	292749	-
PAXXG087510	Trihelix	Chr03	scaffold44	2167414	2168735	-
PAXXG113410	Trihelix	Chr19	scaffold69	1820127	1823614	+
PAXXG125550	Trihelix	Chr16	scaffold83	10086	12940	+
PAXXG125710	Trihelix	Chr16	scaffold83	189277	199268	-
PAXXG131390	Trihelix	Chr11	scaffold89	1258709	1264472	+
PAXXG146490	Trihelix	Chr02	scaffold107	411183	413472	+
PAXXG147090	Trihelix	Chr01	scaffold108	309914	317093	-
PAXXG148560	Trihelix	Chr09	scaffold110	1089183	1090741	-
PAXXG187840	Trihelix	Chr07	scaffold176	861410	875703	-
PAXXG214590	Trihelix	unmapped	scaffold239	775554	815517	+
PAXXG233730	Trihelix	Chr01	scaffold291	574694	576910	-
PAXXG242950	Trihelix	Chr12	scaffold320	335699	337495	+
PAXXG284870	Trihelix	Chr07	scaffold488	325847	365708	-
PAXXG313810	Trihelix	unmapped	scaffold674	206746	208400	+
PAXXG332480	Trihelix	Chr06	scaffold853	185903	212580	-
PAXXG355650	Trihelix	Chr15	scaffold1319	69894	71189	+
PAXXG097400	TUB	Chr04	scaffold54	1276033	1283906	-
PAXXG135000	TUB	Chr16	scaffold93	1684490	1693088	+
PAXXG164640	TUB	Chr10	scaffold134	366623	378620	+
PAXXG210020	TUB	Chr03	scaffold225	92819	112699	-
PAXXG259260	TUB	unmapped	scaffold372	540290	561871	+
PAXXG281220	TUB	Chr02	scaffold469	271995	276683	+
PAXXG285450	TUB	unmapped	scaffold491	350971	365766	-
PAXXG305540	TUB	Chr10	scaffold612	137682	149072	-
PAXXG013210	ULT	Chr15	scaffold4	1087901	1099533	+
PAXXG355770	ULT	unmapped	scaffold1323	91922	101400	-
PAXXG155270	VOZ	Chr10	scaffold121	329585	349503	-
PAXXG027110	Whirly	Chr13	scaffold9	3115405	3131406	+

PAXXG182110	Whirly	Chr06	scaffold166	597810	611225	-
PAXXG365690	Whirly	unmapped	scaffold1821	352	2747	-
PAXXG000440	WRKY	Chr02	scaffold1	785271	788270	-
PAXXG001090	WRKY	Chr02	scaffold1	2086723	2094130	-
PAXXG003100	WRKY	Chr02	scaffold1	6566750	6569491	-
PAXXG004220	WRKY	Chr10	scaffold2	944456	946283	-
PAXXG005620	WRKY	Chr10	scaffold2	4128932	4130329	+
PAXXG005780	WRKY	Chr10	scaffold2	4348517	4354245	+
PAXXG006200	WRKY	Chr10	scaffold2	5431589	5437896	-
PAXXG017060	WRKY	Chr18	scaffold5	240555	244355	-
PAXXG017620	WRKY	Chr18	scaffold5	1392079	1393880	-
PAXXG018550	WRKY	Chr18	scaffold5	3225533	3227553	+
PAXXG031720	WRKY	Chr10	scaffold11	2504735	2513953	+
PAXXG036860	WRKY	Chr16	scaffold13	3392515	3395321	-
PAXXG038480	WRKY	Chr15	scaffold14	2083445	2084883	-
PAXXG040320	WRKY	Chr07	scaffold15	1509874	1512552	+
PAXXG050240	WRKY	Chr07	scaffold20	652634	681002	+
PAXXG054710	WRKY	Chr15	scaffold22	1839304	1841184	+
PAXXG062600	WRKY	Chr08	scaffold27	873407	886150	-
PAXXG063520	WRKY	Chr08	scaffold27	2809068	2811740	-
PAXXG063620	WRKY	Chr08	scaffold27	2984629	2986369	+
PAXXG065760	WRKY	Chr04	scaffold29	721835	725493	-
PAXXG066970	WRKY	Chr04	scaffold29	2796747	2799701	-
PAXXG069880	WRKY	Chr03	scaffold31	2504177	2505517	+
PAXXG074790	WRKY	Chr11	scaffold35	840127	844690	-
PAXXG075560	WRKY	Chr11	scaffold35	2013459	2014818	+
PAXXG078080	WRKY	Chr10	scaffold37	1383933	1386802	-
PAXXG089140	WRKY	Chr04	scaffold46	787597	805817	+
PAXXG089850	WRKY	Chr04	scaffold46	2134142	2135341	+
PAXXG103560	WRKY	Chr12	scaffold59	1327473	1337088	-
PAXXG106690	WRKY	Chr06	scaffold62	2105853	2107682	-
PAXXG108050	WRKY	Chr03	scaffold64	1134236	1136721	-
PAXXG120980	WRKY	Chr09	scaffold77	1631052	1636097	-
PAXXG122110	WRKY	Chr07*	scaffold79	823349	825882	+
PAXXG129620	WRKY	Chr02	scaffold87	1251221	1256386	+
PAXXG133720	WRKY	Chr03	scaffold92	539193	560781	-
PAXXG135080	WRKY	Chr16	scaffold93	1851378	1856890	-
PAXXG138400	WRKY	Chr17	scaffold98	204294	213530	-
PAXXG140960	WRKY	Chr03	scaffold99	1694745	1697173	+
PAXXG144380	WRKY	unmapped	scaffold104	1221457	1223712	+
PAXXG163600	WRKY	Chr03	scaffold132	116516	118129	+
PAXXG163940	WRKY	Chr03	scaffold132	1358680	1362805	+
PAXXG164430	WRKY	unmapped	scaffold133	1341658	1345534	-
PAXXG192190	WRKY	Chr02	scaffold186	554578	558996	+
PAXXG201210	WRKY	Chr12	scaffold203	836893	850270	+
PAXXG201310	WRKY	Chr12	scaffold203	998843	1000842	-
PAXXG202380	WRKY	unmapped	scaffold205	905171	906940	-
PAXXG212670	WRKY	unmapped	scaffold233	92223	93463	-

PAXXG215390	WRKY	Chr07	scaffold241	311526	386992	+
PAXXG222270	WRKY	Chr13	scaffold258	446714	448046	-
PAXXG245730	WRKY	Chr11	scaffold326	1293148	1294860	+
PAXXG252420	WRKY	unmapped	scaffold348	599334	601136	+
PAXXG255590	WRKY	Chr10	scaffold360	529704	531691	-
PAXXG258370	WRKY	Chr17	scaffold369	753924	756570	-
PAXXG266540	WRKY	Chr02	scaffold403	442927	444723	+
PAXXG266560	WRKY	Chr02	scaffold403	477645	478857	+
PAXXG275660	WRKY	unmapped	scaffold444	92590	95842	-
PAXXG282250	WRKY	unmapped	scaffold472	296910	299477	+
PAXXG290780	WRKY	Chr11	scaffold521	152950	154956	+
PAXXG293570	WRKY	Chr06	scaffold539	20417	21859	-
PAXXG299430	WRKY	unmapped	scaffold573	756670	782284	+
PAXXG318610	WRKY	Chr16	scaffold715	260471	263267	-
PAXXG331350	WRKY	Chr06	scaffold840	199393	200656	-
PAXXG338800	WRKY	Chr01	scaffold936	136855	138203	-
PAXXG347570	WRKY	unmapped	scaffold1094	129084	132382	+
PAXXG370840	WRKY	unmapped	scaffold2300	152	2170	+
PAXXG015110	zf-HD	Chr15	scaffold4	4485082	4485649	+
PAXXG029930	zf-HD	Chr14	scaffold10	3377866	3378689	-
PAXXG037790	zf-HD	Chr15	scaffold14	775491	777654	+
PAXXG103230	zf-HD	Chr12	scaffold59	754844	756620	+
PAXXG104570	zf-HD	Chr18	scaffold60	1886126	1887361	+
PAXXG123100	zf-HD	Chr01	scaffold80	892791	894227	+
PAXXG123110	zf-HD	Chr01	scaffold80	907384	907854	-
PAXXG183780	zf-HD	Chr06	scaffold169	136938	138270	+
PAXXG193300	zf-HD	Chr13	scaffold188	457192	458130	+
PAXXG194830	zf-HD	Chr08	scaffold191	275339	276574	+
PAXXG211670	zf-HD	Chr01	scaffold230	644548	645718	-
PAXXG217860	zf-HD	unmapped	scaffold248	15128	16426	+
PAXXG251780	zf-HD	Chr11	scaffold347	127851	128423	+
PAXXG284240	zf-HD	unmapped	scaffold484	370510	371517	-
PAXXG364740	zf-HD	unmapped	scaffold1755	61056	62354	+

**Table S14 List of transporters in *P. aphrodite***						
Gene ID	chromosome	scaffold	start	end	strand	Family Name
PAXXG062950	Chr08	scaffold27	1594701	1620541	+	auxin transport protein (BIG)
PAXXG013000	Chr15	scaffold4	600757	610426	+	Belongs to GPH or MFS family
PAXXG018870	Chr18	scaffold5	3887044	3891161	-	Belongs to GPH or MFS family
PAXXG018880	Chr18	scaffold5	3899069	3903007	-	Belongs to GPH or MFS family
PAXXG030250	Chr14	scaffold10	4009190	4025927	+	Belongs to GPH or MFS family
PAXXG040410	Chr07	scaffold15	1732938	1734036	+	Belongs to GPH or MFS family
PAXXG094040	Chr03	scaffold50	1707294	1709022	-	Belongs to GPH or MFS family
PAXXG143030	unmapped	scaffold102	1203454	1204131	-	Belongs to GPH or MFS family
PAXXG148670	Chr09	scaffold110	1235560	1235901	+	Belongs to GPH or MFS family
PAXXG172420	Chr07	scaffold147	1097232	1118151	-	Belongs to GPH or MFS family
PAXXG226170	Chr08	scaffold269	269286	271135	-	Belongs to GPH or MFS family
PAXXG231130	unmapped	scaffold284	5860	7685	-	Belongs to GPH or MFS family
PAXXG328390	unmapped	scaffold805	152343	156360	-	Belongs to GPH or MFS family
PAXXG328410	unmapped	scaffold805	168448	170446	-	Belongs to GPH or MFS family
PAXXG376010	unmapped	scaffold3057	14920	15706	-	Belongs to GPH or MFS family
PAXXG143210	unmapped	scaffold102	1697296	1699186	-	copper ion transmembrane transporters
PAXXG044720	Chr12*	scaffold17	2442403	2442841	+	Copper transport protein family
PAXXG114940	Chr09	scaffold71	110181	111260	+	Copper transport protein family
PAXXG310680	Chr15	scaffold645	354222	375556	+	electron transport SCO1/SenC family protein
PAXXG120200	Chr11	scaffold76	1703795	1745892	+	Endoplasmic reticulum vesicle transporter protein
PAXXG056780	Chr01	scaffold23	2907614	2916460	+	Golgi transport complex protein-related
PAXXG220320	Chr02	scaffold254	153831	169070	+	Got1/Sft2-like vescicle transport protein family
PAXXG277370	unmapped	scaffold451	45605	59595	+	Got1/Sft2-like vescicle transport protein family
PAXXG030440	Chr14	scaffold10	4456210	4457258	+	Heavy metal transport/detoxification superfamily protein
PAXXG047980	Chr01	scaffold251	62861	63978	-	Heavy metal transport/detoxification superfamily protein
PAXXG070340	Chr01	scaffold32	207644	208999	+	Heavy metal transport/detoxification superfamily protein
PAXXG095170	Chr09	scaffold51	1990316	1994455	-	Heavy metal transport/detoxification superfamily protein
PAXXG097110	Chr04	scaffold54	354476	355933	-	Heavy metal transport/detoxification superfamily protein
PAXXG125160	Chr04	scaffold82	1162782	1169926	-	Heavy metal transport/detoxification superfamily protein
PAXXG147460	Chr01	scaffold108	1148722	1149979	-	Heavy metal transport/detoxification superfamily protein
PAXXG156810	Chr09	scaffold122	1175056	1176113	-	Heavy metal transport/detoxification superfamily protein
PAXXG159470	Chr13	scaffold126	582478	583888	+	Heavy metal transport/detoxification superfamily protein
PAXXG200160	Chr19	scaffold201	305505	306845	-	Heavy metal transport/detoxification superfamily protein
PAXXG231300	unmapped	scaffold284	374960	391208	+	Heavy metal transport/detoxification superfamily protein
PAXXG245680	Chr11	scaffold326	1120206	1121087	+	Heavy metal transport/detoxification superfamily protein
PAXXG220410	Chr02	scaffold254	341797	344657	+	intracellular protein transport protein USO1-related
PAXXG153810	Chr10	scaffold119	363958	365309	+	nr: abscisic acid receptor PYL4-like
PAXXG259860	Chr10	scaffold374	527838	528580	-	nr: abscisic acid receptor PYL4-like
PAXXG080990	Chr04	scaffold39	314835	336264	+	nr: exocyst complex component EXO84B-like
PAXXG192490	Chr02	scaffold186	1427445	1429317	+	nr: heavy metal-associated isoprenylated plant protein
PAXXG100780	Chr09	scaffold57	1715002	1716426	-	nr: heavy metal-associated isoprenylated plant protein 3-like
PAXXG272950	Chr19	scaffold431	320	6725	+	nr: heavy metal-associated isoprenylated plant protein 3-like
PAXXG307660	unmapped	scaffold627	380155	386560	+	nr: heavy metal-associated isoprenylated plant protein 3-like
PAXXG174070	Chr09	scaffold150	749547	752339	-	nr: heavy metal-associated isoprenylated plant protein 5-like
PAXXG134570	Chr16	scaffold93	466537	485973	-	nr: nuclear transport factor 2-like
PAXXG323990	unmapped	scaffold764	182159	189739	+	nr: nuclear transport factor 2-like
PAXXG303890	unmapped	scaffold602	104677	147710	-	nr: protein translocase subunit SECA2, chloroplastic
PAXXG049580	Chr07	scaffold19	2452063	2468826	+	nr: protein transport protein sec23-1
PAXXG068290	Chr12	scaffold30	2403215	2436792	+	nr: protein transport protein SEC23-like
PAXXG162970	Chr01	scaffold130	1993996	2010773	+	nr: protein transport protein SEC23-like
PAXXG201840	unmapped	scaffold204	883500	893739	+	nr: StAR-related lipid transfer protein 7, mitochondrial
PAXXG213480	unmapped	scaffold236	127890	160088	+	nr: vesicle transport protein GOT1-like
PAXXG036880	Chr16	scaffold13	3432566	3432994	-	Nuclear transport factor 2 (NTF2) family protein
PAXXG036890	Chr16	scaffold13	3458418	3481257	+	Nuclear transport factor 2 (NTF2) family protein
PAXXG047100	Chr17	scaffold18	2769733	2778207	-	Nuclear transport factor 2 (NTF2) family protein
PAXXG218020	unmapped	scaffold248	434759	479962	-	Nuclear transport factor 2 (NTF2) family protein
PAXXG241180	Chr09	scaffold314	186335	192912	+	Nuclear transport factor 2 (NTF2) family protein
PAXXG042930	Chr19	scaffold16	1982361	1994419	-	One of secondary transporter type family
PAXXG064270	Chr01	scaffold28	713989	718558	+	One of secondary transporter type family
PAXXG178880	Chr09	scaffold160	1202654	1212984	+	One of secondary transporter type family
PAXXG194580	Chr01	scaffold190	630670	652242	+	One of secondary transporter type family
PAXXG274220	Chr19	scaffold436	473643	477431	-	One of secondary transporter type family
PAXXG283430	unmapped	scaffold478	316033	352801	+	PF03364 - Polyketide cyclase / dehydrase and lipid transport (Polyketide_cyc) (1 of 6)
PAXXG280950	unmapped	scaffold468	381843	398437	-	PF08318 - COG4 transport protein (COG4) (1 of 1)
PAXXG086310	Chr18	scaffold43	1973160	2011719	+	phosphate transporter traffic facilitator1
PAXXG110440	Chr14*	scaffold67	802694	812273	-	Polyketide cyclase / dehydrase and lipid transport protein
PAXXG061300	Chr15	scaffold26	2157424	2182818	-	Polyketide cyclase/dehydrase and lipid transport superfamily protein
PAXXG071870	Chr02	scaffold33	1521184	1531761	+	Polyketide cyclase/dehydrase and lipid transport superfamily protein
PAXXG254960	unmapped	scaffold358	680357	681315	+	Polyketide cyclase/dehydrase and lipid transport superfamily protein
PAXXG280940	unmapped	scaffold468	368231	371795	-	Polyketide cyclase/dehydrase and lipid transport superfamily protein
PAXXG322440	unmapped	scaffold746	113069	113902	-	Polyketide cyclase/dehydrase and lipid transport superfamily protein
PAXXG337380	Chr03	scaffold918	189418	190302	-	Polyketide cyclase/dehydrase and lipid transport superfamily protein
PAXXG073500	unmapped	scaffold34	1546806	1592045	-	Sec23/Sec24 protein transport family protein
PAXXG144240	unmapped	scaffold104	955261	976492	+	Sec23/Sec24 protein transport family protein
PAXXG259940	Chr10	scaffold374	600361	620313	+	sec23/sec24 transport family protein
PAXXG007910	Chr10	scaffold2	9121033	9121862	-	secE/sec61-gamma protein transport protein
PAXXG005860	Chr10	scaffold2	4593200	4605583	+	SLAC1 HOMOLOGUE
PAXXG095370	Chr09	scaffold51	2382978	2385831	+	SLAC1 HOMOLOGUE
PAXXG226080	Chr08	scaffold269	63273	69093	+	SLAC1 HOMOLOGUE
PAXXG305870	Chr06	scaffold614	241413	243594	-	SLAC1 HOMOLOGUE
PAXXG305910	Chr06	scaffold614	299695	317567	+	SLAC1 HOMOLOGUE
PAXXG305930	Chr06	scaffold614	322119	326502	+	SLAC1 HOMOLOGUE
PAXXG026640	Chr13	scaffold9	1923131	1929053	+	SWEET gene family

PAXXG103040	Chr12	scaffold59	485247	493118	+	SWEET gene family
PAXXG103050	Chr12	scaffold59	494342	496253	-	SWEET gene family
PAXXG134520	Chr16	scaffold93	330514	332680	-	SWEET gene family
PAXXG134630	Chr16	scaffold93	613740	617618	-	SWEET gene family
PAXXG141820	Chr02	scaffold100	1267081	1269398	+	SWEET gene family
PAXXG146070	Chr13	scaffold106	775119	780293	+	SWEET gene family
PAXXG159670	Chr13	scaffold126	865185	869435	-	SWEET gene family
PAXXG177570	Chr02	scaffold158	31933	33888	-	SWEET gene family
PAXXG177580	Chr02	scaffold158	43224	45224	+	SWEET gene family
PAXXG177760	Chr02	scaffold158	476776	478679	-	SWEET gene family
PAXXG189690	Chr15	scaffold180	600402	617403	+	SWEET gene family
PAXXG189700	Chr15	scaffold180	629557	630719	+	SWEET gene family
PAXXG207870	unmapped	scaffold219	268343	270591	-	SWEET gene family
PAXXG209710	unmapped	scaffold223	1001729	1003343	-	SWEET gene family
PAXXG223930	Chr12	scaffold262	789729	793285	-	SWEET gene family
PAXXG256720	Chr01	scaffold365	427873	430989	-	SWEET gene family
PAXXG275360	unmapped	scaffold442	235184	237441	-	SWEET gene family
PAXXG275370	unmapped	scaffold442	266467	267954	+	SWEET gene family
PAXXG275380	unmapped	scaffold442	310626	312725	+	SWEET gene family
PAXXG285710	Chr07	scaffold493	109799	111442	+	SWEET gene family
PAXXG306100	unmapped	scaffold616	6079	7779	-	SWEET gene family
PAXXG306110	unmapped	scaffold616	51176	56962	-	SWEET gene family
PAXXG306120	unmapped	scaffold616	191583	193189	-	SWEET gene family
PAXXG324220	Chr16	scaffold766	173410	176618	+	SWEET gene family
PAXXG339350	unmapped	scaffold945	122385	124503	+	SWEET gene family
PAXXG339360	unmapped	scaffold945	162478	164172	+	SWEET gene family
PAXXG002130	Chr02	scaffold1	4359352	4361120	-	The Amino Acid/Auxin Permease (AAAP) Family
PAXXG006570	Chr10	scaffold2	6292858	6296668	-	The Amino Acid/Auxin Permease (AAAP) Family
PAXXG018040	Chr18	scaffold5	2099116	2185101	+	The Amino Acid/Auxin Permease (AAAP) Family
PAXXG029840	Chr14	scaffold10	3235400	3238171	-	The Amino Acid/Auxin Permease (AAAP) Family
PAXXG035680	Chr16	scaffold13	1058502	1062206	+	The Amino Acid/Auxin Permease (AAAP) Family
PAXXG039900	Chr07	scaffold15	571646	576498	+	The Amino Acid/Auxin Permease (AAAP) Family
PAXXG042500	Chr19	scaffold16	1169055	1171212	+	The Amino Acid/Auxin Permease (AAAP) Family
PAXXG065340	Chr01	scaffold28	3212272	3226676	-	The Amino Acid/Auxin Permease (AAAP) Family
PAXXG073330	unmapped	scaffold34	1277326	1283464	-	The Amino Acid/Auxin Permease (AAAP) Family
PAXXG074150	unmapped	scaffold34	2791179	2796685	+	The Amino Acid/Auxin Permease (AAAP) Family
PAXXG081140	Chr04	scaffold39	581437	596059	-	The Amino Acid/Auxin Permease (AAAP) Family
PAXXG089190	Chr04	scaffold46	896876	918741	+	The Amino Acid/Auxin Permease (AAAP) Family
PAXXG090010	Chr04	scaffold46	2385371	2390695	-	The Amino Acid/Auxin Permease (AAAP) Family
PAXXG099560	Chr03	scaffold56	1507867	1511837	+	The Amino Acid/Auxin Permease (AAAP) Family
PAXXG119360	Chr11	scaffold76	340696	343341	-	The Amino Acid/Auxin Permease (AAAP) Family
PAXXG129700	Chr02	scaffold87	1391536	1394955	-	The Amino Acid/Auxin Permease (AAAP) Family
PAXXG137760	Chr04	scaffold97	420559	466658	-	The Amino Acid/Auxin Permease (AAAP) Family
PAXXG137810	Chr04	scaffold97	508701	514302	+	The Amino Acid/Auxin Permease (AAAP) Family
PAXXG143340	Chr11	scaffold103	418235	424350	+	The Amino Acid/Auxin Permease (AAAP) Family
PAXXG143390	Chr11	scaffold103	583626	597978	-	The Amino Acid/Auxin Permease (AAAP) Family
PAXXG154030	Chr10	scaffold119	752195	779157	-	The Amino Acid/Auxin Permease (AAAP) Family
PAXXG163910	Chr03	scaffold132	1199256	1219299	-	The Amino Acid/Auxin Permease (AAAP) Family
PAXXG165960	unmapped	scaffold136	1350872	1355193	+	The Amino Acid/Auxin Permease (AAAP) Family
PAXXG165970	unmapped	scaffold136	1362221	1367429	+	The Amino Acid/Auxin Permease (AAAP) Family
PAXXG178070	Chr19	scaffold159	41776	47081	-	The Amino Acid/Auxin Permease (AAAP) Family
PAXXG185460	Chr08	scaffold172	263899	267265	-	The Amino Acid/Auxin Permease (AAAP) Family
PAXXG186200	Chr17	scaffold173	299069	303590	-	The Amino Acid/Auxin Permease (AAAP) Family
PAXXG193460	Chr13	scaffold188	681860	684128	+	The Amino Acid/Auxin Permease (AAAP) Family
PAXXG194360	Chr01	scaffold190	247911	249336	-	The Amino Acid/Auxin Permease (AAAP) Family
PAXXG214560	unmapped	scaffold239	550045	555223	-	The Amino Acid/Auxin Permease (AAAP) Family
PAXXG218120	unmapped	scaffold248	794156	796731	+	The Amino Acid/Auxin Permease (AAAP) Family
PAXXG229810	unmapped	scaffold279	110368	116653	-	The Amino Acid/Auxin Permease (AAAP) Family
PAXXG249620	Chr03	scaffold339	570920	599365	+	The Amino Acid/Auxin Permease (AAAP) Family
PAXXG262260	unmapped	scaffold385	120	3154	+	The Amino Acid/Auxin Permease (AAAP) Family
PAXXG270040	Chr19	scaffold419	95268	120656	-	The Amino Acid/Auxin Permease (AAAP) Family
PAXXG321790	unmapped	scaffold741	196560	199159	-	The Amino Acid/Auxin Permease (AAAP) Family
PAXXG324510	unmapped	scaffold768	80506	100549	+	The Amino Acid/Auxin Permease (AAAP) Family
PAXXG338180	Chr10	scaffold929	108361	111920	-	The Amino Acid/Auxin Permease (AAAP) Family
PAXXG001360	Chr02	scaffold1	2766285	2784619	-	The Amino Acid-Polyamine-Organocation (APC) Family
PAXXG028260	Chr14	scaffold10	599087	621955	-	The Amino Acid-Polyamine-Organocation (APC) Family
PAXXG031340	Chr10	scaffold11	1803065	1811079	-	The Amino Acid-Polyamine-Organocation (APC) Family
PAXXG039390	Chr15	scaffold14	3929083	3935448	+	The Amino Acid-Polyamine-Organocation (APC) Family
PAXXG045480	Chr12*	scaffold17	3788169	3797505	-	The Amino Acid-Polyamine-Organocation (APC) Family
PAXXG047710	Chr17	scaffold18	3602983	3609199	+	The Amino Acid-Polyamine-Organocation (APC) Family
PAXXG075690	Chr11	scaffold35	2141888	2156154	-	The Amino Acid-Polyamine-Organocation (APC) Family
PAXXG102930	Chr12	scaffold59	331509	334180	+	The Amino Acid-Polyamine-Organocation (APC) Family
PAXXG120250	Chr11	scaffold76	1813321	1825524	-	The Amino Acid-Polyamine-Organocation (APC) Family
PAXXG123940	Chr15	scaffold81	875176	877502	-	The Amino Acid-Polyamine-Organocation (APC) Family
PAXXG135200	Chr16	scaffold93	2254724	2258478	+	The Amino Acid-Polyamine-Organocation (APC) Family
PAXXG158060	unmapped	scaffold124	1416415	1437506	+	The Amino Acid-Polyamine-Organocation (APC) Family
PAXXG226550	Chr08	scaffold269	1310210	1319421	-	The Amino Acid-Polyamine-Organocation (APC) Family
PAXXG236620	Chr14	scaffold300	424595	430637	+	The Amino Acid-Polyamine-Organocation (APC) Family
PAXXG281290	Chr02	scaffold469	431167	445186	-	The Amino Acid-Polyamine-Organocation (APC) Family
PAXXG318140	Chr07	scaffold713	70819	95278	-	The Amino Acid-Polyamine-Organocation (APC) Family
PAXXG332400	Chr06	scaffold853	15016	18548	-	The Amino Acid-Polyamine-Organocation (APC) Family
PAXXG374090	unmapped	scaffold2744	16675	18608	+	The Amino Acid-Polyamine-Organocation (APC) Family
PAXXG064030	Chr01	scaffold28	231641	233712	-	The Ammonia Transporter Channel (Amt) Family
PAXXG115470	Chr09	scaffold71	1017500	1019268	+	The Ammonia Transporter Channel (Amt) Family
PAXXG259880	Chr10	scaffold374	541782	543874	-	The Ammonia Transporter Channel (Amt) Family
PAXXG259900	Chr10	scaffold374	554348	559499	-	The Ammonia Transporter Channel (Amt) Family

PAXXG004050	Chr10	scaffold2	610284	614942	+	The Anion Exchanger (AE) Family
PAXXG061230	Chr15	scaffold26	2076246	2084953	+	The Anion Exchanger (AE) Family
PAXXG099040	Chr03	scaffold56	346428	350994	-	The Anion Exchanger (AE) Family
PAXXG129040	Chr02	scaffold87	407582	411655	-	The Anion Exchanger (AE) Family
PAXXG011440	Chr08	scaffold3	5106390	5113694	-	The Annexin (Annexin) Family
PAXXG053560	Chr18	scaffold21	3239780	3243620	+	The Annexin (Annexin) Family
PAXXG147410	Chr01	scaffold108	1041998	1083623	-	The Annexin (Annexin) Family
PAXXG221670	Chr16	scaffold257	83220	85935	-	The Annexin (Annexin) Family
PAXXG221680	Chr16	scaffold257	94854	106211	+	The Annexin (Annexin) Family
PAXXG249890	Chr14	scaffold340	654937	660194	-	The Annexin (Annexin) Family
PAXXG298810	Chr15	scaffold571	191844	195021	+	The Annexin (Annexin) Family
PAXXG042430	Chr19	scaffold16	1085288	1098266	-	The Aromatic Acid Exporter (ArAE) Family
PAXXG045190	Chr12*	scaffold17	3377882	3390173	+	The Aromatic Acid Exporter (ArAE) Family
PAXXG070790	Chr01	scaffold32	1925203	1942473	+	The Aromatic Acid Exporter (ArAE) Family
PAXXG108110	Chr03	scaffold64	1258011	1258553	-	The Aromatic Acid Exporter (ArAE) Family
PAXXG124430	Chr15	scaffold81	1865198	1868652	+	The Aromatic Acid Exporter (ArAE) Family
PAXXG149100	Chr07	scaffold111	715782	719861	+	The Aromatic Acid Exporter (ArAE) Family
PAXXG231250	unmapped	scaffold284	313691	317622	-	The Aromatic Acid Exporter (ArAE) Family
PAXXG251260	Chr18	scaffold345	305374	310506	+	The Aromatic Acid Exporter (ArAE) Family
PAXXG261900	Chr16	scaffold383	457127	466033	+	The Aromatic Acid Exporter (ArAE) Family
PAXXG264480	unmapped	scaffold393	155493	158596	+	The Aromatic Acid Exporter (ArAE) Family
PAXXG312090	unmapped	scaffold658	208201	249842	-	The Aromatic Acid Exporter (ArAE) Family
PAXXG323190	unmapped	scaffold755	202399	204167	+	The Aromatic Acid Exporter (ArAE) Family
PAXXG329350	Chr01	scaffold815	183011	187015	+	The Aromatic Acid Exporter (ArAE) Family
PAXXG029870	Chr14	scaffold10	3257963	3277996	-	The Arsenite-Antimonite (ArsAB) Efflux Family
PAXXG032960	Chr04	scaffold12	507567	534952	-	The Arsenite-Antimonite (ArsAB) Efflux Family
PAXXG042610	Chr19	scaffold16	1354522	1373189	+	The Arsenite-Antimonite (ArsAB) Efflux Family
PAXXG129130	Chr02	scaffold87	489402	503460	+	The ATP:ADP Antiporter (AAA) Family
PAXXG004150	Chr10	scaffold2	907211	916110	+	The ATP-binding Cassette (ABC) Superfamily
PAXXG004170	Chr10	scaffold2	921595	924305	+	The ATP-binding Cassette (ABC) Superfamily
PAXXG004210	Chr10	scaffold2	928679	930094	+	The ATP-binding Cassette (ABC) Superfamily
PAXXG005170	Chr10	scaffold2	3205427	3213306	-	The ATP-binding Cassette (ABC) Superfamily
PAXXG005180	Chr10	scaffold2	3255249	3262081	-	The ATP-binding Cassette (ABC) Superfamily
PAXXG005980	Chr10	scaffold2	4834230	4847356	-	The ATP-binding Cassette (ABC) Superfamily
PAXXG006000	Chr10	scaffold2	4865454	4881962	-	The ATP-binding Cassette (ABC) Superfamily
PAXXG006320	Chr10	scaffold2	5772284	5781937	+	The ATP-binding Cassette (ABC) Superfamily
PAXXG006340	Chr10	scaffold2	5796292	5811567	+	The ATP-binding Cassette (ABC) Superfamily
PAXXG008280	Chr10	scaffold2	9712219	9724601	+	The ATP-binding Cassette (ABC) Superfamily
PAXXG010540	Chr08	scaffold3	3062238	3063952	+	The ATP-binding Cassette (ABC) Superfamily
PAXXG013870	Chr15	scaffold4	2400859	2430154	+	The ATP-binding Cassette (ABC) Superfamily
PAXXG015930	Chr15	scaffold4	5945591	5945986	+	The ATP-binding Cassette (ABC) Superfamily
PAXXG015940	Chr15	scaffold4	5950235	5957044	+	The ATP-binding Cassette (ABC) Superfamily
PAXXG024360	Chr11	scaffold8	1509878	1516367	+	The ATP-binding Cassette (ABC) Superfamily
PAXXG024370	Chr11	scaffold8	1519184	1536085	-	The ATP-binding Cassette (ABC) Superfamily
PAXXG024420	Chr11	scaffold8	1576583	1623674	-	The ATP-binding Cassette (ABC) Superfamily
PAXXG026210	Chr13	scaffold9	1127638	1153611	+	The ATP-binding Cassette (ABC) Superfamily
PAXXG027450	Chr13	scaffold9	3846422	3848060	-	The ATP-binding Cassette (ABC) Superfamily
PAXXG027470	Chr13	scaffold9	3882933	3893054	-	The ATP-binding Cassette (ABC) Superfamily
PAXXG028280	Chr14	scaffold10	645300	647927	-	The ATP-binding Cassette (ABC) Superfamily
PAXXG028335	Chr14	scaffold10	675284	675890	-	The ATP-binding Cassette (ABC) Superfamily
PAXXG031320	Chr10	scaffold11	1757447	1777385	+	The ATP-binding Cassette (ABC) Superfamily
PAXXG036680	Chr16	scaffold13	2998988	3032124	+	The ATP-binding Cassette (ABC) Superfamily
PAXXG037510	Chr15	scaffold14	197030	208072	-	The ATP-binding Cassette (ABC) Superfamily
PAXXG039100	Chr15	scaffold14	3390299	3406603	-	The ATP-binding Cassette (ABC) Superfamily
PAXXG053520	Chr18	scaffold21	2980825	3158950	-	The ATP-binding Cassette (ABC) Superfamily
PAXXG054610	Chr15	scaffold22	1495143	1661940	-	The ATP-binding Cassette (ABC) Superfamily
PAXXG055380	Chr15	scaffold22	3247052	3293266	+	The ATP-binding Cassette (ABC) Superfamily
PAXXG055610	Chr01	scaffold23	372705	393710	+	The ATP-binding Cassette (ABC) Superfamily
PAXXG056170	Chr01	scaffold23	1637516	1737221	-	The ATP-binding Cassette (ABC) Superfamily
PAXXG059470	Chr04	scaffold25	1463801	1496600	-	The ATP-binding Cassette (ABC) Superfamily
PAXXG059680	Chr04	scaffold25	1937819	1956364	+	The ATP-binding Cassette (ABC) Superfamily
PAXXG061560	Chr15	scaffold26	2574416	2588863	-	The ATP-binding Cassette (ABC) Superfamily
PAXXG062380	Chr08	scaffold27	485161	491911	-	The ATP-binding Cassette (ABC) Superfamily
PAXXG065270	Chr01	scaffold28	3019723	3102123	-	The ATP-binding Cassette (ABC) Superfamily
PAXXG065550	Chr04	scaffold29	209911	299738	-	The ATP-binding Cassette (ABC) Superfamily
PAXXG066070	Chr04	scaffold29	1183982	1193933	+	The ATP-binding Cassette (ABC) Superfamily
PAXXG066480	Chr04	scaffold29	2040688	2050258	+	The ATP-binding Cassette (ABC) Superfamily
PAXXG068840	Chr03	scaffold31	401640	408989	-	The ATP-binding Cassette (ABC) Superfamily
PAXXG071270	Chr02	scaffold33	68346	72444	-	The ATP-binding Cassette (ABC) Superfamily
PAXXG071580	Chr02	scaffold33	817416	849246	-	The ATP-binding Cassette (ABC) Superfamily
PAXXG072230	Chr02	scaffold33	2217322	2228591	+	The ATP-binding Cassette (ABC) Superfamily
PAXXG074940	Chr11	scaffold35	1017981	1032607	+	The ATP-binding Cassette (ABC) Superfamily
PAXXG076560	Chr02	scaffold36	1250183	1251908	+	The ATP-binding Cassette (ABC) Superfamily
PAXXG077320	Chr10	scaffold37	251473	254114	-	The ATP-binding Cassette (ABC) Superfamily
PAXXG081550	Chr04	scaffold39	1267514	1286676	+	The ATP-binding Cassette (ABC) Superfamily
PAXXG087180	Chr03	scaffold44	1330237	1335442	-	The ATP-binding Cassette (ABC) Superfamily
PAXXG089360	Chr04	scaffold46	1215503	1286488	-	The ATP-binding Cassette (ABC) Superfamily
PAXXG097360	Chr04	scaffold54	1128440	1132450	-	The ATP-binding Cassette (ABC) Superfamily
PAXXG099010	Chr03	scaffold56	244568	251474	+	The ATP-binding Cassette (ABC) Superfamily
PAXXG100230	Chr09	scaffold57	633825	644084	-	The ATP-binding Cassette (ABC) Superfamily
PAXXG100790	Chr09	scaffold57	1736922	1762351	+	The ATP-binding Cassette (ABC) Superfamily
PAXXG101640	Chr16	scaffold58	647036	666397	-	The ATP-binding Cassette (ABC) Superfamily
PAXXG101890	Chr16	scaffold58	948460	963771	-	The ATP-binding Cassette (ABC) Superfamily
PAXXG102600	Chr16	scaffold58	2027774	2118431	+	The ATP-binding Cassette (ABC) Superfamily
PAXXG112420	Chr19	scaffold69	233264	237768	-	The ATP-binding Cassette (ABC) Superfamily
PAXXG115890	Chr09	scaffold71	1803930	1809520	+	The ATP-binding Cassette (ABC) Superfamily

PAXXG118960	Chr10	scaffold75	1609359	1631816	+	The ATP-binding Cassette (ABC) Superfamily
PAXXG122930	Chr01	scaffold80	443832	454434	-	The ATP-binding Cassette (ABC) Superfamily
PAXXG129290	Chr02	scaffold87	656132	665377	+	The ATP-binding Cassette (ABC) Superfamily
PAXXG129570	Chr02	scaffold87	1138322	1153376	-	The ATP-binding Cassette (ABC) Superfamily
PAXXG129650	Chr02	scaffold87	1298177	1299569	-	The ATP-binding Cassette (ABC) Superfamily
PAXXG129660	Chr02	scaffold87	1301669	1315562	-	The ATP-binding Cassette (ABC) Superfamily
PAXXG129740	Chr02	scaffold87	1427274	1436475	+	The ATP-binding Cassette (ABC) Superfamily
PAXXG129760	Chr02	scaffold87	1446596	1459719	+	The ATP-binding Cassette (ABC) Superfamily
PAXXG129770	Chr02	scaffold87	1469964	1486392	+	The ATP-binding Cassette (ABC) Superfamily
PAXXG129800	Chr02	scaffold87	1520156	1534098	+	The ATP-binding Cassette (ABC) Superfamily
PAXXG131040	Chr11	scaffold89	646982	665229	-	The ATP-binding Cassette (ABC) Superfamily
PAXXG131090	Chr11	scaffold89	684893	712547	-	The ATP-binding Cassette (ABC) Superfamily
PAXXG133610	Chr03	scaffold92	364155	370242	+	The ATP-binding Cassette (ABC) Superfamily
PAXXG135630	Chr07	scaffold94	1446163	1453812	-	The ATP-binding Cassette (ABC) Superfamily
PAXXG138820	Chr17	scaffold98	1010995	1018828	+	The ATP-binding Cassette (ABC) Superfamily
PAXXG141140	Chr02	scaffold100	270147	276770	+	The ATP-binding Cassette (ABC) Superfamily
PAXXG144110	unmapped	scaffold104	652429	707325	+	The ATP-binding Cassette (ABC) Superfamily
PAXXG145210	Chr11	scaffold105	979484	983542	+	The ATP-binding Cassette (ABC) Superfamily
PAXXG145830	Chr13	scaffold106	220390	239611	+	The ATP-binding Cassette (ABC) Superfamily
PAXXG147070	Chr01	scaffold108	266778	273496	+	The ATP-binding Cassette (ABC) Superfamily
PAXXG150050	unmapped	scaffold112	1483819	1494723	+	The ATP-binding Cassette (ABC) Superfamily
PAXXG153580	Chr03	scaffold118	1495897	1520158	+	The ATP-binding Cassette (ABC) Superfamily
PAXXG155640	Chr10	scaffold121	1113766	1161700	-	The ATP-binding Cassette (ABC) Superfamily
PAXXG155660	Chr10	scaffold121	1183934	1209413	-	The ATP-binding Cassette (ABC) Superfamily
PAXXG155700	Chr10	scaffold121	1240705	1248758	-	The ATP-binding Cassette (ABC) Superfamily
PAXXG156450	Chr09	scaffold122	183705	195130	+	The ATP-binding Cassette (ABC) Superfamily
PAXXG156740	Chr09	scaffold122	973353	986827	+	The ATP-binding Cassette (ABC) Superfamily
PAXXG156940	Chr09	scaffold122	1383206	1392626	+	The ATP-binding Cassette (ABC) Superfamily
PAXXG157980	unmapped	scaffold124	1085274	1168914	-	The ATP-binding Cassette (ABC) Superfamily
PAXXG158770	Chr10	scaffold125	995575	1011035	+	The ATP-binding Cassette (ABC) Superfamily
PAXXG169820	unmapped	scaffold143	821194	830360	-	The ATP-binding Cassette (ABC) Superfamily
PAXXG175080	Chr04	scaffold152	469406	480178	+	The ATP-binding Cassette (ABC) Superfamily
PAXXG175440	Chr10	scaffold153	243045	250767	+	The ATP-binding Cassette (ABC) Superfamily
PAXXG192790	unmapped	scaffold187	498099	500107	-	The ATP-binding Cassette (ABC) Superfamily
PAXXG193240	Chr13	scaffold188	355140	360516	-	The ATP-binding Cassette (ABC) Superfamily
PAXXG210100	Chr03	scaffold225	450956	452999	-	The ATP-binding Cassette (ABC) Superfamily
PAXXG220420	Chr02	scaffold254	345597	355999	-	The ATP-binding Cassette (ABC) Superfamily
PAXXG221220	Chr01	scaffold255	866117	911776	-	The ATP-binding Cassette (ABC) Superfamily
PAXXG226050	Chr08	scaffold269	7245	9397	-	The ATP-binding Cassette (ABC) Superfamily
PAXXG226370	Chr08	scaffold269	829289	846039	+	The ATP-binding Cassette (ABC) Superfamily
PAXXG227470	unmapped	scaffold271	745832	786363	-	The ATP-binding Cassette (ABC) Superfamily
PAXXG230400	Chr14	scaffold280	528270	533615	-	The ATP-binding Cassette (ABC) Superfamily
PAXXG236720	Chr14	scaffold300	570194	578893	+	The ATP-binding Cassette (ABC) Superfamily
PAXXG239190	Chr01	scaffold308	598558	628908	-	The ATP-binding Cassette (ABC) Superfamily
PAXXG252140	Chr11	scaffold347	923041	946335	+	The ATP-binding Cassette (ABC) Superfamily
PAXXG253690	Chr06	scaffold353	467373	469325	-	The ATP-binding Cassette (ABC) Superfamily
PAXXG254090	Chr18	scaffold356	288447	332597	-	The ATP-binding Cassette (ABC) Superfamily
PAXXG255780	Chr19	scaffold361	307779	340160	+	The ATP-binding Cassette (ABC) Superfamily
PAXXG268230	Chr09	scaffold409	421189	473291	+	The ATP-binding Cassette (ABC) Superfamily
PAXXG275260	Chr11	scaffold441	541590	559470	-	The ATP-binding Cassette (ABC) Superfamily
PAXXG282920	Chr01	scaffold476	176074	190087	+	The ATP-binding Cassette (ABC) Superfamily
PAXXG282970	Chr01	scaffold476	242840	257378	-	The ATP-binding Cassette (ABC) Superfamily
PAXXG294070	Chr19	scaffold542	360062	414904	-	The ATP-binding Cassette (ABC) Superfamily
PAXXG296310	unmapped	scaffold555	179971	191997	-	The ATP-binding Cassette (ABC) Superfamily
PAXXG297670	Chr06	scaffold564	156941	182580	-	The ATP-binding Cassette (ABC) Superfamily
PAXXG302140	Chr16	scaffold590	333852	368978	-	The ATP-binding Cassette (ABC) Superfamily
PAXXG304700	Chr13	scaffold608	237325	240269	+	The ATP-binding Cassette (ABC) Superfamily
PAXXG324330	unmapped	scaffold767	112447	119755	-	The ATP-binding Cassette (ABC) Superfamily
PAXXG346110	unmapped	scaffold1063	156474	161723	-	The ATP-binding Cassette (ABC) Superfamily
PAXXG346280	unmapped	scaffold1067	1057	3209	+	The ATP-binding Cassette (ABC) Superfamily
PAXXG354310	unmapped	scaffold1267	53277	63768	-	The ATP-binding Cassette (ABC) Superfamily
PAXXG354320	unmapped	scaffold1267	66984	67561	-	The ATP-binding Cassette (ABC) Superfamily
PAXXG373580	unmapped	scaffold2673	13001	14189	+	The ATP-binding Cassette (ABC) Superfamily
PAXXG044800	Chr12*	scaffold17	2670222	2674899	+	The Auxin Efflux Carrier (AEC) Family
PAXXG102990	Chr12	scaffold59	416428	425829	+	The Auxin Efflux Carrier (AEC) Family
PAXXG104330	Chr18	scaffold60	1033739	1089844	+	The Auxin Efflux Carrier (AEC) Family
PAXXG115320	Chr09	scaffold71	808561	819426	-	The Auxin Efflux Carrier (AEC) Family
PAXXG131700	Chr11	scaffold89	1871583	1897115	+	The Auxin Efflux Carrier (AEC) Family
PAXXG134720	Chr16	scaffold93	850027	852326	-	The Auxin Efflux Carrier (AEC) Family
PAXXG137655	Chr04	scaffold97	192350	202838	+	The Auxin Efflux Carrier (AEC) Family
PAXXG137660	Chr04	scaffold97	210428	231652	-	The Auxin Efflux Carrier (AEC) Family
PAXXG137670	Chr04	scaffold97	274251	289438	+	The Auxin Efflux Carrier (AEC) Family
PAXXG139080	Chr17	scaffold98	1423506	1440293	-	The Auxin Efflux Carrier (AEC) Family
PAXXG149500	unmapped	scaffold112	21627	37711	-	The Auxin Efflux Carrier (AEC) Family
PAXXG152490	Chr10	scaffold117	483501	486822	+	The Auxin Efflux Carrier (AEC) Family
PAXXG229350	Chr06	scaffold277	618747	621470	-	The Auxin Efflux Carrier (AEC) Family
PAXXG251040	Chr11	scaffold344	21245	128479	+	The Auxin Efflux Carrier (AEC) Family
PAXXG252230	Chr11	scaffold347	1225099	1247531	+	The Auxin Efflux Carrier (AEC) Family
PAXXG298380	unmapped	scaffold568	294578	299201	+	The Auxin Efflux Carrier (AEC) Family
PAXXG379310	unmapped	scaffold3803	482	16037	-	The Auxin Efflux Carrier (AEC) Family
PAXXG001690	Chr02	scaffold1	3382482	3424325	-	The Bile Acid:Na+ Symporter (BASS) Family
PAXXG024700	Chr11	scaffold8	2420100	2429915	-	The Bile Acid:Na+ Symporter (BASS) Family
PAXXG067340	Chr12	scaffold30	19388	38058	+	The Bile Acid:Na+ Symporter (BASS) Family
PAXXG193140	Chr13	scaffold188	202535	211219	-	The Bile Acid:Na+ Symporter (BASS) Family
PAXXG226500	Chr08	scaffold269	1083438	1137389	-	The Bile Acid:Na+ Symporter (BASS) Family
PAXXG000570	Chr02	scaffold1	961951	962747	+	The Ca2+:Cation Antiporter (CaCA) Family

PAXXG009440	Chr08	scaffold3	1380925	1402581	+	The Ca2+:Cation Antiporter (CaCA) Family
PAXXG013760	Chr15	scaffold4	2196318	2198786	-	The Ca2+:Cation Antiporter (CaCA) Family
PAXXG029560	Chr14	scaffold10	2654934	2666119	-	The Ca2+:Cation Antiporter (CaCA) Family
PAXXG034010	Chr04	scaffold12	2341558	2350904	+	The Ca2+:Cation Antiporter (CaCA) Family
PAXXG039490	Chr15	scaffold14	4020606	4022970	-	The Ca2+:Cation Antiporter (CaCA) Family
PAXXG047730	Chr17	scaffold18	3659718	3661908	+	The Ca2+:Cation Antiporter (CaCA) Family
PAXXG049590	Chr07	scaffold19	2490523	2493113	+	The Ca2+:Cation Antiporter (CaCA) Family
PAXXG075640	Chr11	scaffold35	2112187	2114131	-	The Ca2+:Cation Antiporter (CaCA) Family
PAXXG075650	Chr11	scaffold35	2121496	2123303	-	The Ca2+:Cation Antiporter (CaCA) Family
PAXXG123450	Chr01	scaffold80	1659527	1678188	+	The Ca2+:Cation Antiporter (CaCA) Family
PAXXG152360	Chr10	scaffold117	152127	154026	-	The Ca2+:Cation Antiporter (CaCA) Family
PAXXG185600	Chr08	scaffold172	450131	452856	+	The Ca2+:Cation Antiporter (CaCA) Family
PAXXG201410	unmapped	scaffold204	39310	46638	+	The Ca2+:Cation Antiporter (CaCA) Family
PAXXG209170	unmapped	scaffold222	520419	522765	+	The Ca2+:Cation Antiporter (CaCA) Family
PAXXG237950	unmapped	scaffold304	193446	216906	-	The Ca2+:Cation Antiporter (CaCA) Family
PAXXG281330	Chr02	scaffold469	506445	516637	+	The Ca2+:Cation Antiporter (CaCA) Family
PAXXG307210	Chr16	scaffold625	300994	308859	-	The Ca2+:Cation Antiporter (CaCA) Family
PAXXG007040	Chr10	scaffold2	7244415	7273040	-	The Cation Diffusion Facilitator (CDF) Family
PAXXG012510	Chr08	scaffold3	7134483	7138816	-	The Cation Diffusion Facilitator (CDF) Family
PAXXG044630	Chr12*	scaffold17	2129426	2155369	-	The Cation Diffusion Facilitator (CDF) Family
PAXXG046620	Chr17	scaffold18	1924605	1929332	+	The Cation Diffusion Facilitator (CDF) Family
PAXXG130850	Chr11	scaffold89	319266	332629	-	The Cation Diffusion Facilitator (CDF) Family
PAXXG133580	Chr03	scaffold92	287622	290650	-	The Cation Diffusion Facilitator (CDF) Family
PAXXG133950	Chr03	scaffold92	1119057	1126162	-	The Cation Diffusion Facilitator (CDF) Family
PAXXG164710	Chr10	scaffold134	526258	541498	-	The Cation Diffusion Facilitator (CDF) Family
PAXXG189420	Chr15	scaffold180	313498	331303	+	The Cation Diffusion Facilitator (CDF) Family
PAXXG200080	Chr19	scaffold201	151727	166186	+	The Cation Diffusion Facilitator (CDF) Family
PAXXG274290	unmapped	scaffold437	259080	263097	+	The Cation Diffusion Facilitator (CDF) Family
PAXXG099490	Chr03	scaffold56	1199089	1262168	-	The Cation-Chloride Cotransporter (CCC) Family
PAXXG022830	Chr11	scaffold7	2575012	2603983	+	The Chloride Carrier/Channel (ClC) Family
PAXXG024530	Chr11	scaffold8	2075170	2080734	-	The Chloride Carrier/Channel (ClC) Family
PAXXG033510	Chr04	scaffold12	1619701	1624991	+	The Chloride Carrier/Channel (ClC) Family
PAXXG049900	Chr07	scaffold20	56948	99145	-	The Chloride Carrier/Channel (ClC) Family
PAXXG072950	unmapped	scaffold34	498016	503163	-	The Chloride Carrier/Channel (ClC) Family
PAXXG160300	unmapped	scaffold127	509844	527514	-	The Chloride Carrier/Channel (ClC) Family
PAXXG164420	unmapped	scaffold133	1306190	1326889	+	The Chloride Carrier/Channel (ClC) Family
PAXXG071510	Chr02	scaffold33	610795	658310	+	The Chloroplast Envelope Anion Channel-forming Tic110 (Tic110) Family
PAXXG024460	Chr11	scaffold8	1881600	1905540	-	The Chloroplast Envelope Protein Translocase (CEPT or Tic-Toc) Family
PAXXG025870	Chr13	scaffold9	454846	456553	-	The Chloroplast Envelope Protein Translocase (CEPT or Tic-Toc) Family
PAXXG029060	Chr14	scaffold10	1762345	1766454	+	The Chloroplast Envelope Protein Translocase (CEPT or Tic-Toc) Family
PAXXG056980	Chr01	scaffold23	3345309	3382252	+	The Chloroplast Envelope Protein Translocase (CEPT or Tic-Toc) Family
PAXXG097070	Chr04	scaffold54	253406	257992	+	The Chloroplast Envelope Protein Translocase (CEPT or Tic-Toc) Family
PAXXG097870	Chr04	scaffold54	2111999	2129728	+	The Chloroplast Envelope Protein Translocase (CEPT or Tic-Toc) Family
PAXXG099750	Chr03	scaffold56	1807363	1828086	+	The Chloroplast Envelope Protein Translocase (CEPT or Tic-Toc) Family
PAXXG110600	Chr14*	scaffold67	1021681	1029787	+	The Chloroplast Envelope Protein Translocase (CEPT or Tic-Toc) Family
PAXXG173050	Chr08	scaffold149	267453	286895	+	The Chloroplast Envelope Protein Translocase (CEPT or Tic-Toc) Family
PAXXG195140	Chr08	scaffold191	655522	709212	-	The Chloroplast Envelope Protein Translocase (CEPT or Tic-Toc) Family
PAXXG268590	Chr06	scaffold410	552810	574539	+	The Chloroplast Envelope Protein Translocase (CEPT or Tic-Toc) Family
PAXXG112350	Chr19	scaffold69	158330	164876	-	The Chloroplast Maltose Exporter (MEX) Family
PAXXG115860	Chr09	scaffold71	1773372	1784947	-	The Chloroplast Maltose Exporter (MEX) Family
PAXXG043530	Chr19	scaffold16	3854566	3878802	-	The Choline Transporter Like (CTL) Family
PAXXG154390	Chr10	scaffold119	1401860	1404470	+	The Choline Transporter Like (CTL) Family
PAXXG157000	Chr09	scaffold122	1482086	1497074	-	The Choline Transporter Like (CTL) Family
PAXXG312060	unmapped	scaffold658	80399	91633	-	The Choline Transporter Like (CTL) Family
PAXXG083790	Chr18	scaffold41	755879	756628	+	The Copper Transporter (Ctr) Family
PAXXG098600	Chr01	scaffold55	1655532	1656645	-	The Copper Transporter (Ctr) Family
PAXXG138140	Chr04	scaffold97	1355231	1356014	-	The Copper Transporter (Ctr) Family
PAXXG138150	Chr04	scaffold97	1389957	1390871	-	The Copper Transporter (Ctr) Family
PAXXG020670	Chr04	scaffold6	2656440	2704413	-	The CorA Metal Ion Transporter (MIT) Family
PAXXG032220	Chr10	scaffold11	3581565	3582960	+	The CorA Metal Ion Transporter (MIT) Family
PAXXG032240	Chr10	scaffold11	3602843	3604141	+	The CorA Metal Ion Transporter (MIT) Family
PAXXG054390	Chr15	scaffold22	1165996	1183420	+	The CorA Metal Ion Transporter (MIT) Family
PAXXG057980	Chr13	scaffold24	2122659	2131783	-	The CorA Metal Ion Transporter (MIT) Family
PAXXG087910	Chr06	scaffold45	497984	534259	-	The CorA Metal Ion Transporter (MIT) Family
PAXXG119900	Chr11	scaffold76	1370933	1380918	-	The CorA Metal Ion Transporter (MIT) Family
PAXXG137860	Chr04	scaffold97	591496	607928	+	The CorA Metal Ion Transporter (MIT) Family
PAXXG149280	Chr07	scaffold111	1114439	1145891	+	The CorA Metal Ion Transporter (MIT) Family
PAXXG149330	Chr07	scaffold111	1193565	1195535	+	The CorA Metal Ion Transporter (MIT) Family
PAXXG154240	Chr10	scaffold119	1174642	1203845	+	The CorA Metal Ion Transporter (MIT) Family
PAXXG170790	Chr18	scaffold145	332377	340706	-	The CorA Metal Ion Transporter (MIT) Family
PAXXG215560	Chr07	scaffold241	845413	882908	+	The CorA Metal Ion Transporter (MIT) Family
PAXXG307730	unmapped	scaffold628	313920	316874	+	The CorA Metal Ion Transporter (MIT) Family
PAXXG315250	unmapped	scaffold687	95045	108871	-	The CorA Metal Ion Transporter (MIT) Family
PAXXG337270	Chr10	scaffold917	182379	217511	+	The CorA Metal Ion Transporter (MIT) Family
PAXXG386520	unmapped	scaffold6504	2749	3981	-	The CorA Metal Ion Transporter (MIT) Family
PAXXG045050	Chr12*	scaffold17	3080767	3104569	-	The Cytochrome Oxidase Biogenesis (Oxa1) Family
PAXXG077780	Chr10	scaffold37	983578	991360	-	The Cytochrome Oxidase Biogenesis (Oxa1) Family
PAXXG094680	Chr09	scaffold51	828917	906356	+	The Cytochrome Oxidase Biogenesis (Oxa1) Family
PAXXG124350	Chr15	scaffold81	1721181	1734033	-	The Cytochrome Oxidase Biogenesis (Oxa1) Family
PAXXG089010	Chr04	scaffold46	505091	507981	-	The Divalent Anion:Na+ Symporter (DASS) Family
PAXXG194910	Chr08	scaffold191	367339	373519	+	The Divalent Anion:Na+ Symporter (DASS) Family
PAXXG223350	Chr06	scaffold260	838824	867879	-	The Divalent Anion:Na+ Symporter (DASS) Family
PAXXG002300	Chr02	scaffold1	4833449	4834786	-	The Drug/Metabolite Transporter (DMT) Superfamily
PAXXG003290	Chr02	scaffold1	7071850	7072724	-	The Drug/Metabolite Transporter (DMT) Superfamily
PAXXG003540	Chr02	scaffold1	7581567	7596557	-	The Drug/Metabolite Transporter (DMT) Superfamily
PAXXG005200	Chr10	scaffold2	3290000	3291964	-	The Drug/Metabolite Transporter (DMT) Superfamily

PAXXG006700	Chr10	scaffold2	6566841	6571788	-	The Drug/Metabolite Transporter (DMT) Superfamily
PAXXG010570	Chr08	scaffold3	3113763	3119658	+	The Drug/Metabolite Transporter (DMT) Superfamily
PAXXG015220	Chr15	scaffold4	4740433	4744204	+	The Drug/Metabolite Transporter (DMT) Superfamily
PAXXG015690	Chr15	scaffold4	5565212	5566457	+	The Drug/Metabolite Transporter (DMT) Superfamily
PAXXG015700	Chr15	scaffold4	5570139	5572119	+	The Drug/Metabolite Transporter (DMT) Superfamily
PAXXG020500	Chr04	scaffold6	2274208	2276223	-	The Drug/Metabolite Transporter (DMT) Superfamily
PAXXG021730	Chr04	scaffold6	4952165	4991956	-	The Drug/Metabolite Transporter (DMT) Superfamily
PAXXG022680	Chr11	scaffold7	2202537	2203660	-	The Drug/Metabolite Transporter (DMT) Superfamily
PAXXG022800	Chr11	scaffold7	2524772	2555094	+	The Drug/Metabolite Transporter (DMT) Superfamily
PAXXG023190	Chr11	scaffold7	3427087	3436425	-	The Drug/Metabolite Transporter (DMT) Superfamily
PAXXG028610	Chr14	scaffold10	1041651	1045737	+	The Drug/Metabolite Transporter (DMT) Superfamily
PAXXG034320	Chr04	scaffold12	2914287	2916254	+	The Drug/Metabolite Transporter (DMT) Superfamily
PAXXG036770	Chr16	scaffold13	3209063	3236790	+	The Drug/Metabolite Transporter (DMT) Superfamily
PAXXG038070	Chr15	scaffold14	1237924	1238737	-	The Drug/Metabolite Transporter (DMT) Superfamily
PAXXG038080	Chr15	scaffold14	1238792	1239451	-	The Drug/Metabolite Transporter (DMT) Superfamily
PAXXG043260	Chr19	scaffold16	2890205	2892755	+	The Drug/Metabolite Transporter (DMT) Superfamily
PAXXG043270	Chr19	scaffold16	2900401	2903321	+	The Drug/Metabolite Transporter (DMT) Superfamily
PAXXG051690	Chr07	scaffold20	3046172	3065072	-	The Drug/Metabolite Transporter (DMT) Superfamily
PAXXG054920	Chr15	scaffold22	2304189	2309999	-	The Drug/Metabolite Transporter (DMT) Superfamily
PAXXG060010	Chr04	scaffold25	2782948	2784404	+	The Drug/Metabolite Transporter (DMT) Superfamily
PAXXG060620	Chr15	scaffold26	556169	576283	+	The Drug/Metabolite Transporter (DMT) Superfamily
PAXXG063500	Chr08	scaffold27	2735281	2757871	+	The Drug/Metabolite Transporter (DMT) Superfamily
PAXXG066270	Chr04	scaffold29	1515344	1524875	-	The Drug/Metabolite Transporter (DMT) Superfamily
PAXXG066280	Chr04	scaffold29	1525117	1529768	-	The Drug/Metabolite Transporter (DMT) Superfamily
PAXXG066290	Chr04	scaffold29	1530889	1544835	-	The Drug/Metabolite Transporter (DMT) Superfamily
PAXXG066460	Chr04	scaffold29	1992929	2008543	-	The Drug/Metabolite Transporter (DMT) Superfamily
PAXXG069910	Chr03	scaffold31	2532393	2566745	-	The Drug/Metabolite Transporter (DMT) Superfamily
PAXXG070220	Chr03	scaffold31	3104510	3122944	+	The Drug/Metabolite Transporter (DMT) Superfamily
PAXXG077490	Chr10	scaffold37	622684	636842	-	The Drug/Metabolite Transporter (DMT) Superfamily
PAXXG083010	Chr03	scaffold40	1566309	1571705	-	The Drug/Metabolite Transporter (DMT) Superfamily
PAXXG083040	Chr03	scaffold40	1609824	1612417	+	The Drug/Metabolite Transporter (DMT) Superfamily
PAXXG085300	Chr03	scaffold42	2043178	2111188	+	The Drug/Metabolite Transporter (DMT) Superfamily
PAXXG086950	Chr03	scaffold44	874431	885544	-	The Drug/Metabolite Transporter (DMT) Superfamily
PAXXG087360	Chr03	scaffold44	1900922	1906884	-	The Drug/Metabolite Transporter (DMT) Superfamily
PAXXG087370	Chr03	scaffold44	1914924	1918357	-	The Drug/Metabolite Transporter (DMT) Superfamily
PAXXG087380	Chr03	scaffold44	1929499	1934647	-	The Drug/Metabolite Transporter (DMT) Superfamily
PAXXG087390	Chr03	scaffold44	1937018	1943254	-	The Drug/Metabolite Transporter (DMT) Superfamily
PAXXG089900	Chr04	scaffold46	2178261	2207588	+	The Drug/Metabolite Transporter (DMT) Superfamily
PAXXG092660	Chr15	scaffold49	1114732	1118783	-	The Drug/Metabolite Transporter (DMT) Superfamily
PAXXG093810	Chr03	scaffold50	1134211	1140612	+	The Drug/Metabolite Transporter (DMT) Superfamily
PAXXG094280	Chr03	scaffold50	2184309	2195479	+	The Drug/Metabolite Transporter (DMT) Superfamily
PAXXG095040	Chr09	scaffold51	1752254	1758561	+	The Drug/Metabolite Transporter (DMT) Superfamily
PAXXG100030	Chr09	scaffold57	175112	178174	+	The Drug/Metabolite Transporter (DMT) Superfamily
PAXXG101900	Chr16	scaffold58	975289	977358	+	The Drug/Metabolite Transporter (DMT) Superfamily
PAXXG101930	Chr16	scaffold58	990888	993202	-	The Drug/Metabolite Transporter (DMT) Superfamily
PAXXG101940	Chr16	scaffold58	999276	1001643	-	The Drug/Metabolite Transporter (DMT) Superfamily
PAXXG102220	Chr16	scaffold58	1398252	1411964	+	The Drug/Metabolite Transporter (DMT) Superfamily
PAXXG102860	Chr12	scaffold59	247035	249463	-	The Drug/Metabolite Transporter (DMT) Superfamily
PAXXG107960	Chr03	scaffold64	858868	861057	-	The Drug/Metabolite Transporter (DMT) Superfamily
PAXXG110710	Chr14*	scaffold67	1183610	1194139	+	The Drug/Metabolite Transporter (DMT) Superfamily
PAXXG111560	Chr08*	scaffold68	443254	454228	-	The Drug/Metabolite Transporter (DMT) Superfamily
PAXXG111580	Chr08*	scaffold68	488377	491724	-	The Drug/Metabolite Transporter (DMT) Superfamily
PAXXG115790	Chr09	scaffold71	1671348	1673065	+	The Drug/Metabolite Transporter (DMT) Superfamily
PAXXG116120	unmapped	scaffold72	847983	859901	-	The Drug/Metabolite Transporter (DMT) Superfamily
PAXXG116660	Chr05	scaffold73	411466	419318	+	The Drug/Metabolite Transporter (DMT) Superfamily
PAXXG120450	Chr09	scaffold77	376362	379513	+	The Drug/Metabolite Transporter (DMT) Superfamily
PAXXG120470	Chr09	scaffold77	391661	401753	+	The Drug/Metabolite Transporter (DMT) Superfamily
PAXXG125600	Chr16	scaffold83	68351	73062	+	The Drug/Metabolite Transporter (DMT) Superfamily
PAXXG125620	Chr16	scaffold83	83052	88025	+	The Drug/Metabolite Transporter (DMT) Superfamily
PAXXG131370	Chr11	scaffold89	1189346	1193581	-	The Drug/Metabolite Transporter (DMT) Superfamily
PAXXG133790	Chr03	scaffold92	763399	789362	-	The Drug/Metabolite Transporter (DMT) Superfamily
PAXXG134440	Chr16	scaffold93	206295	230367	+	The Drug/Metabolite Transporter (DMT) Superfamily
PAXXG138850	Chr17	scaffold98	1089574	1092409	-	The Drug/Metabolite Transporter (DMT) Superfamily
PAXXG143820	unmapped	scaffold104	57206	65456	+	The Drug/Metabolite Transporter (DMT) Superfamily
PAXXG153600	Chr03	scaffold118	1528840	1530680	-	The Drug/Metabolite Transporter (DMT) Superfamily
PAXXG155410	Chr10	scaffold121	681536	682444	-	The Drug/Metabolite Transporter (DMT) Superfamily
PAXXG160400	unmapped	scaffold127	726955	781953	-	The Drug/Metabolite Transporter (DMT) Superfamily
PAXXG162530	Chr01	scaffold130	1144609	1146840	-	The Drug/Metabolite Transporter (DMT) Superfamily
PAXXG162600	Chr01	scaffold130	1230825	1250720	+	The Drug/Metabolite Transporter (DMT) Superfamily
PAXXG162620	Chr01	scaffold130	1276761	1280551	+	The Drug/Metabolite Transporter (DMT) Superfamily
PAXXG163700	Chr03	scaffold132	383084	415826	+	The Drug/Metabolite Transporter (DMT) Superfamily
PAXXG172050	Chr07	scaffold147	723120	728845	+	The Drug/Metabolite Transporter (DMT) Superfamily
PAXXG172900	Chr02	scaffold148	1027548	1134938	+	The Drug/Metabolite Transporter (DMT) Superfamily
PAXXG175850	Chr07	scaffold154	123033	273895	-	The Drug/Metabolite Transporter (DMT) Superfamily
PAXXG178770	Chr09	scaffold160	931477	936529	+	The Drug/Metabolite Transporter (DMT) Superfamily
PAXXG182570	Chr02	scaffold167	93590	96844	-	The Drug/Metabolite Transporter (DMT) Superfamily
PAXXG211550	Chr01	scaffold230	434222	440022	+	The Drug/Metabolite Transporter (DMT) Superfamily
PAXXG211570	Chr01	scaffold230	467725	469719	+	The Drug/Metabolite Transporter (DMT) Superfamily
PAXXG213970	Chr13	scaffold237	497551	502778	+	The Drug/Metabolite Transporter (DMT) Superfamily
PAXXG219840	Chr13	scaffold253	512207	514351	-	The Drug/Metabolite Transporter (DMT) Superfamily
PAXXG220480	Chr02	scaffold254	475225	496506	-	The Drug/Metabolite Transporter (DMT) Superfamily
PAXXG221280	unmapped	scaffold256	41360	114560	-	The Drug/Metabolite Transporter (DMT) Superfamily
PAXXG222970	Chr06	scaffold260	140404	171165	-	The Drug/Metabolite Transporter (DMT) Superfamily
PAXXG222980	Chr06	scaffold260	179350	183309	+	The Drug/Metabolite Transporter (DMT) Superfamily
PAXXG254050	Chr18	scaffold356	196128	219620	+	The Drug/Metabolite Transporter (DMT) Superfamily
PAXXG254970	unmapped	scaffold359	37290	44570	-	The Drug/Metabolite Transporter (DMT) Superfamily

PAXXG259220	unmapped	scaffold372	436648	438328	+	The Drug/Metabolite Transporter (DMT) Superfamily
PAXXG260570	unmapped	scaffold377	222352	242508	+	The Drug/Metabolite Transporter (DMT) Superfamily
PAXXG284700	unmapped	scaffold487	237633	240658	+	The Drug/Metabolite Transporter (DMT) Superfamily
PAXXG285130	unmapped	scaffold490	216287	246600	-	The Drug/Metabolite Transporter (DMT) Superfamily
PAXXG285870	unmapped	scaffold494	27204	34944	+	The Drug/Metabolite Transporter (DMT) Superfamily
PAXXG286730	unmapped	scaffold498	209411	250710	-	The Drug/Metabolite Transporter (DMT) Superfamily
PAXXG304750	Chr13	scaffold608	299058	304273	-	The Drug/Metabolite Transporter (DMT) Superfamily
PAXXG304760	Chr13	scaffold608	336733	340738	-	The Drug/Metabolite Transporter (DMT) Superfamily
PAXXG306020	Chr01	scaffold615	159216	180267	+	The Drug/Metabolite Transporter (DMT) Superfamily
PAXXG321980	Chr10	scaffold742	281459	292325	-	The Drug/Metabolite Transporter (DMT) Superfamily
PAXXG322610	unmapped	scaffold748	137671	140016	+	The Drug/Metabolite Transporter (DMT) Superfamily
PAXXG322620	unmapped	scaffold748	167693	169973	-	The Drug/Metabolite Transporter (DMT) Superfamily
PAXXG322630	unmapped	scaffold748	188249	190340	-	The Drug/Metabolite Transporter (DMT) Superfamily
PAXXG322650	unmapped	scaffold748	249346	252392	-	The Drug/Metabolite Transporter (DMT) Superfamily
PAXXG322660	unmapped	scaffold748	271542	275245	-	The Drug/Metabolite Transporter (DMT) Superfamily
PAXXG330620	Chr14	scaffold831	56912	70612	-	The Drug/Metabolite Transporter (DMT) Superfamily
PAXXG340150	unmapped	scaffold957	132036	133649	+	The Drug/Metabolite Transporter (DMT) Superfamily
PAXXG353460	unmapped	scaffold1236	124722	126047	+	The Drug/Metabolite Transporter (DMT) Superfamily
PAXXG382890	unmapped	scaffold4889	690	2628	+	The Drug/Metabolite Transporter (DMT) Superfamily
PAXXG385740	unmapped	scaffold6012	700	3474	-	The Drug/Metabolite Transporter (DMT) Superfamily
PAXXG387680	unmapped	scaffold7327	272	2305	+	The Drug/Metabolite Transporter (DMT) Superfamily
PAXXG159290	Chr13	scaffold126	288273	310574	+	The Equilibrative Nucleoside Transporter (ENT) Family
PAXXG163180	unmapped	scaffold131	418619	445422	+	The Equilibrative Nucleoside Transporter (ENT) Family
PAXXG182620	Chr02	scaffold167	141619	154179	-	The Equilibrative Nucleoside Transporter (ENT) Family
PAXXG182630	Chr02	scaffold167	156773	157372	-	The Equilibrative Nucleoside Transporter (ENT) Family
PAXXG199410	Chr18	scaffold200	288732	293006	-	The Equilibrative Nucleoside Transporter (ENT) Family
PAXXG153670	Chr10	scaffold119	51274	73319	+	The Ferroportin (FP) Family
PAXXG174800	Chr19	scaffold151	1011151	1020454	-	The Ferroportin (FP) Family
PAXXG055210	Chr15	scaffold22	2862387	2874413	-	The Folate-Biopterin Transporter (FBT) Family
PAXXG075370	Chr11	scaffold35	1691580	1714518	-	The Folate-Biopterin Transporter (FBT) Family
PAXXG164660	Chr10	scaffold134	397509	401240	-	The Folate-Biopterin Transporter (FBT) Family
PAXXG191620	Chr10	scaffold184	1592255	1667610	-	The Folate-Biopterin Transporter (FBT) Family
PAXXG348680	Chr15	scaffold1119	39359	95022	-	The Folate-Biopterin Transporter (FBT) Family
PAXXG377420	unmapped	scaffold3305	3610	4396	+	The Folate-Biopterin Transporter (FBT) Family
PAXXG012790	Chr15	scaffold4	214178	221005	-	The Glutamate-gated Ion Channel (GIC) Family of Neurotransmitter Receptors
PAXXG033000	Chr04	scaffold12	612329	613912	+	The Glutamate-gated Ion Channel (GIC) Family of Neurotransmitter Receptors
PAXXG056730	Chr01	scaffold23	2792368	2845706	-	The Glutamate-gated Ion Channel (GIC) Family of Neurotransmitter Receptors
PAXXG058540	Chr13	scaffold24	3253695	3280467	+	The Glutamate-gated Ion Channel (GIC) Family of Neurotransmitter Receptors
PAXXG069360	Chr03	scaffold31	1519671	1523510	-	The Glutamate-gated Ion Channel (GIC) Family of Neurotransmitter Receptors
PAXXG069390	Chr03	scaffold31	1616124	1620855	-	The Glutamate-gated Ion Channel (GIC) Family of Neurotransmitter Receptors
PAXXG159150	Chr13	scaffold126	15326	22023	+	The Glutamate-gated Ion Channel (GIC) Family of Neurotransmitter Receptors
PAXXG027740	Chr14	scaffold10	11431	35674	+	The Glycerol Uptake (GUP) Family
PAXXG106320	Chr06	scaffold62	1468648	1531765	-	The Glycerol Uptake (GUP) Family
PAXXG295090	unmapped	scaffold549	269270	271053	-	The Glycerol Uptake (GUP) Family
PAXXG016490	Chr15	scaffold4	6608899	6614298	-	The Glycoside-Pentoside-Hexuronide (GPH):Cation Symporter Family
PAXXG030240	Chr14	scaffold10	3968762	3996831	+	The Glycoside-Pentoside-Hexuronide (GPH):Cation Symporter Family
PAXXG032140	Chr10	scaffold11	3422233	3443571	+	The Glycoside-Pentoside-Hexuronide (GPH):Cation Symporter Family
PAXXG065630	Chr04	scaffold29	489859	525773	+	The Glycoside-Pentoside-Hexuronide (GPH):Cation Symporter Family
PAXXG195390	Chr08	scaffold191	1112434	1116264	-	The Glycoside-Pentoside-Hexuronide (GPH):Cation Symporter Family
PAXXG239080	Chr01	scaffold308	470756	478682	+	The Glycoside-Pentoside-Hexuronide (GPH):Cation Symporter Family
PAXXG000970	Chr02	scaffold1	1676837	1701174	-	The H+- or Na+-translocating F-type, V-type and A-type ATPase (F-ATPase) Superfamily
PAXXG007560	Chr10	scaffold2	8423415	8427011	+	The H+- or Na+-translocating F-type, V-type and A-type ATPase (F-ATPase) Superfamily
PAXXG022210	Chr11	scaffold7	923589	928742	-	The H+- or Na+-translocating F-type, V-type and A-type ATPase (F-ATPase) Superfamily
PAXXG023950	Chr11	scaffold8	337533	338748	-	The H+- or Na+-translocating F-type, V-type and A-type ATPase (F-ATPase) Superfamily
PAXXG025010	Chr11	scaffold8	3011479	3031401	+	The H+- or Na+-translocating F-type, V-type and A-type ATPase (F-ATPase) Superfamily
PAXXG026440	Chr13	scaffold9	1464688	1467107	+	The H+- or Na+-translocating F-type, V-type and A-type ATPase (F-ATPase) Superfamily
PAXXG028560	Chr14	scaffold10	978103	982842	-	The H+- or Na+-translocating F-type, V-type and A-type ATPase (F-ATPase) Superfamily
PAXXG030350	Chr14	scaffold10	4284519	4285679	+	The H+- or Na+-translocating F-type, V-type and A-type ATPase (F-ATPase) Superfamily
PAXXG038560	Chr15	scaffold14	2228310	2267627	-	The H+- or Na+-translocating F-type, V-type and A-type ATPase (F-ATPase) Superfamily
PAXXG042480	Chr19	scaffold16	1148005	1166803	-	The H+- or Na+-translocating F-type, V-type and A-type ATPase (F-ATPase) Superfamily
PAXXG053680	Chr18	scaffold21	3674901	3675426	+	The H+- or Na+-translocating F-type, V-type and A-type ATPase (F-ATPase) Superfamily
PAXXG053690	Chr18	scaffold21	3675436	3675879	+	The H+- or Na+-translocating F-type, V-type and A-type ATPase (F-ATPase) Superfamily
PAXXG053710	Chr18	scaffold21	3687669	3688523	+	The H+- or Na+-translocating F-type, V-type and A-type ATPase (F-ATPase) Superfamily
PAXXG053760	Chr18	scaffold21	3732382	3733772	+	The H+- or Na+-translocating F-type, V-type and A-type ATPase (F-ATPase) Superfamily
PAXXG062500	Chr08	scaffold27	760075	777337	-	The H+- or Na+-translocating F-type, V-type and A-type ATPase (F-ATPase) Superfamily
PAXXG079870	Chr06	scaffold38	1030377	1056022	+	The H+- or Na+-translocating F-type, V-type and A-type ATPase (F-ATPase) Superfamily
PAXXG087480	Chr03	scaffold44	2115263	2117861	-	The H+- or Na+-translocating F-type, V-type and A-type ATPase (F-ATPase) Superfamily
PAXXG106480	Chr06	scaffold62	1801266	1820072	-	The H+- or Na+-translocating F-type, V-type and A-type ATPase (F-ATPase) Superfamily
PAXXG110790	Chr14*	scaffold67	1307413	1388393	+	The H+- or Na+-translocating F-type, V-type and A-type ATPase (F-ATPase) Superfamily
PAXXG130870	Chr11	scaffold89	361112	383101	-	The H+- or Na+-translocating F-type, V-type and A-type ATPase (F-ATPase) Superfamily
PAXXG132900	Chr02	scaffold91	24323	32561	+	The H+- or Na+-translocating F-type, V-type and A-type ATPase (F-ATPase) Superfamily
PAXXG133590	Chr03	scaffold92	297752	311238	+	The H+- or Na+-translocating F-type, V-type and A-type ATPase (F-ATPase) Superfamily
PAXXG142970	unmapped	scaffold102	971720	1031967	-	The H+- or Na+-translocating F-type, V-type and A-type ATPase (F-ATPase) Superfamily
PAXXG147480	Chr01	scaffold108	1162959	1209694	-	The H+- or Na+-translocating F-type, V-type and A-type ATPase (F-ATPase) Superfamily
PAXXG158750	Chr10	scaffold125	978941	985096	+	The H+- or Na+-translocating F-type, V-type and A-type ATPase (F-ATPase) Superfamily
PAXXG165290	Chr06	scaffold135	433406	464977	+	The H+- or Na+-translocating F-type, V-type and A-type ATPase (F-ATPase) Superfamily
PAXXG169020	Chr15	scaffold142	329470	365769	+	The H+- or Na+-translocating F-type, V-type and A-type ATPase (F-ATPase) Superfamily
PAXXG170770	Chr18	scaffold145	286878	305568	-	The H+- or Na+-translocating F-type, V-type and A-type ATPase (F-ATPase) Superfamily
PAXXG181970	Chr06	scaffold166	364608	400178	-	The H+- or Na+-translocating F-type, V-type and A-type ATPase (F-ATPase) Superfamily
PAXXG185090	Chr09	scaffold171	730162	751016	+	The H+- or Na+-translocating F-type, V-type and A-type ATPase (F-ATPase) Superfamily
PAXXG193100	Chr13	scaffold188	113496	121760	-	The H+- or Na+-translocating F-type, V-type and A-type ATPase (F-ATPase) Superfamily
PAXXG214680	Chr06	scaffold240	32395	40428	-	The H+- or Na+-translocating F-type, V-type and A-type ATPase (F-ATPase) Superfamily
PAXXG219570	Chr13	scaffold253	71894	73356	+	The H+- or Na+-translocating F-type, V-type and A-type ATPase (F-ATPase) Superfamily
PAXXG253900	unmapped	scaffold355	733237	738842	-	The H+- or Na+-translocating F-type, V-type and A-type ATPase (F-ATPase) Superfamily
PAXXG314440	unmapped	scaffold680	252801	271204	+	The H+- or Na+-translocating F-type, V-type and A-type ATPase (F-ATPase) Superfamily
PAXXG337370	Chr03	scaffold918	137873	178141	+	The H+- or Na+-translocating F-type, V-type and A-type ATPase (F-ATPase) Superfamily

PAXXG345310	unmapped	scaffold1051	54801	64224	-	The H+- or Na+-translocating F-type, V-type and A-type ATPase (F-ATPase) Superfamily
PAXXG358510	unmapped	scaffold1428	4628	24018	-	The H+- or Na+-translocating F-type, V-type and A-type ATPase (F-ATPase) Superfamily
PAXXG371040	unmapped	scaffold2325	16066	17499	+	The H+- or Na+-translocating F-type, V-type and A-type ATPase (F-ATPase) Superfamily
PAXXG380090	unmapped	scaffold4070	4	3110	-	The H+- or Na+-translocating F-type, V-type and A-type ATPase (F-ATPase) Superfamily
PAXXG024140	Chr11	scaffold8	818799	824034	+	The H+-translocating Pyrophosphatase (H+-PPase) Family
PAXXG027380	Chr13	scaffold9	3725768	3728995	+	The H+-translocating Pyrophosphatase (H+-PPase) Family
PAXXG033580	Chr04	scaffold12	1724232	1731306	+	The H+-translocating Pyrophosphatase (H+-PPase) Family
PAXXG042710	Chr19	scaffold16	1555155	1592990	-	The H+-translocating Pyrophosphatase (H+-PPase) Family
PAXXG000210	Chr02	scaffold1	458635	464529	-	The HlyC/CorC (HCC) Family
PAXXG085480	Chr18	scaffold43	119901	178379	-	The HlyC/CorC (HCC) Family
PAXXG097520	Chr04	scaffold54	1512843	1540529	-	The HlyC/CorC (HCC) Family
PAXXG114350	Chr09	scaffold70	1135755	1167025	+	The HlyC/CorC (HCC) Family
PAXXG119180	Chr11	scaffold76	85515	102549	-	The HlyC/CorC (HCC) Family
PAXXG228630	Chr16	scaffold275	265197	277556	-	The HlyC/CorC (HCC) Family
PAXXG031160	Chr10	scaffold11	1441776	1482891	+	The Hydroxy/Aromatic Amino Acid Permease (HAAAP) Family
PAXXG040140	Chr07	scaffold15	995947	1007587	-	The Inorganic Phosphate Transporter (PiT) Family
PAXXG157960	unmapped	scaffold124	1035828	1036255	-	The Inorganic Phosphate Transporter (PiT) Family
PAXXG229080	unmapped	scaffold276	632209	634803	+	The Integral Membrane Peroxisomal Protein Importer-2 (PPI2) Family
PAXXG279950	Chr09	scaffold463	316527	334356	+	The Integral Membrane Peroxisomal Protein Importer-2 (PPI2) Family
PAXXG317150	Chr12	scaffold702	132129	159741	+	The Integral Membrane Peroxisomal Protein Importer-2 (PPI2) Family
PAXXG049300	Chr07	scaffold19	1696105	1736493	-	The Intracellular Chloride Channel (CLIC) Family
PAXXG077250	Chr10	scaffold37	180612	183803	+	The Intracellular Chloride Channel (CLIC) Family
PAXXG108860	Chr17	scaffold65	1399169	1400483	-	The Intracellular Chloride Channel (CLIC) Family
PAXXG112170	Chr08*	scaffold68	2098388	2108336	-	The Intracellular Chloride Channel (CLIC) Family
PAXXG133920	Chr03	scaffold92	1067258	1086904	+	The Intracellular Chloride Channel (CLIC) Family
PAXXG250620	unmapped	scaffold342	575431	603152	-	The Intracellular Chloride Channel (CLIC) Family
PAXXG006940	Chr10	scaffold2	7051317	7063044	-	The Iron/Lead Transporter (ILT) Superfamily
PAXXG022020	Chr11	scaffold7	662114	665509	+	The Iron/Lead Transporter (ILT) Superfamily
PAXXG030460	Chr14	scaffold10	4503267	4506689	+	The Iron/Lead Transporter (ILT) Superfamily
PAXXG047050	Chr17	scaffold18	2737119	2739811	-	The Iron/Lead Transporter (ILT) Superfamily
PAXXG047060	Chr17	scaffold18	2740496	2743441	-	The Iron/Lead Transporter (ILT) Superfamily
PAXXG067120	Chr04	scaffold29	3100965	3104657	+	The Iron/Lead Transporter (ILT) Superfamily
PAXXG073460	unmapped	scaffold34	1515017	1519325	-	The Iron/Lead Transporter (ILT) Superfamily
PAXXG073550	unmapped	scaffold34	1649749	1655675	-	The Iron/Lead Transporter (ILT) Superfamily
PAXXG073570	unmapped	scaffold34	1677890	1682653	-	The Iron/Lead Transporter (ILT) Superfamily
PAXXG078090	Chr10	scaffold37	1395574	1399043	+	The Iron/Lead Transporter (ILT) Superfamily
PAXXG082170	Chr04	scaffold39	2418082	2421356	-	The Iron/Lead Transporter (ILT) Superfamily
PAXXG106110	Chr06	scaffold62	1068279	1094734	-	The Iron/Lead Transporter (ILT) Superfamily
PAXXG113320	Chr19	scaffold69	1663987	1670739	+	The Iron/Lead Transporter (ILT) Superfamily
PAXXG122230	Chr07*	scaffold79	957062	960863	-	The Iron/Lead Transporter (ILT) Superfamily
PAXXG140540	Chr03	scaffold99	624094	641731	-	The Iron/Lead Transporter (ILT) Superfamily
PAXXG140580	Chr03	scaffold99	722809	725835	+	The Iron/Lead Transporter (ILT) Superfamily
PAXXG143750	Chr11	scaffold103	1634477	1665201	-	The Iron/Lead Transporter (ILT) Superfamily
PAXXG147450	Chr01	scaffold108	1131121	1134933	-	The Iron/Lead Transporter (ILT) Superfamily
PAXXG153700	Chr10	scaffold119	132763	137763	-	The Iron/Lead Transporter (ILT) Superfamily
PAXXG207120	unmapped	scaffold217	957390	994010	-	The Iron/Lead Transporter (ILT) Superfamily
PAXXG229190	unmapped	scaffold276	871435	882313	-	The Iron/Lead Transporter (ILT) Superfamily
PAXXG240200	Chr18	scaffold311	530404	534443	+	The Iron/Lead Transporter (ILT) Superfamily
PAXXG269920	Chr11	scaffold418	374130	376845	-	The Iron/Lead Transporter (ILT) Superfamily
PAXXG287530	Chr16	scaffold504	172305	174762	+	The Iron/Lead Transporter (ILT) Superfamily
PAXXG287540	Chr16	scaffold504	204430	206470	+	The Iron/Lead Transporter (ILT) Superfamily
PAXXG287560	Chr16	scaffold504	251937	255299	+	The Iron/Lead Transporter (ILT) Superfamily
PAXXG302400	Chr13	scaffold592	199604	204029	+	The Iron/Lead Transporter (ILT) Superfamily
PAXXG306950	unmapped	scaffold622	296901	300560	+	The Iron/Lead Transporter (ILT) Superfamily
PAXXG329500	unmapped	scaffold817	119544	133624	-	The Iron/Lead Transporter (ILT) Superfamily
PAXXG368810	unmapped	scaffold2047	34099	48085	-	The Iron/Lead Transporter (ILT) Superfamily
PAXXG379560	unmapped	scaffold3874	1907	3145	-	The Iron/Lead Transporter (ILT) Superfamily
PAXXG008740	Chr08	scaffold3	195797	211318	-	The K+ Uptake Permease (KUP) Family
PAXXG008890	Chr08	scaffold3	498745	515748	+	The K+ Uptake Permease (KUP) Family
PAXXG014680	Chr15	scaffold4	3793945	3824342	+	The K+ Uptake Permease (KUP) Family
PAXXG026230	Chr13	scaffold9	1165765	1192303	+	The K+ Uptake Permease (KUP) Family
PAXXG032510	Chr10	scaffold11	4260399	4271460	+	The K+ Uptake Permease (KUP) Family
PAXXG034960	Chr04	scaffold12	3994444	4018053	+	The K+ Uptake Permease (KUP) Family
PAXXG046910	Chr17	scaffold18	2454194	2460568	+	The K+ Uptake Permease (KUP) Family
PAXXG053240	Chr18	scaffold21	2523191	2540785	-	The K+ Uptake Permease (KUP) Family
PAXXG057470	Chr13	scaffold24	1278729	1285470	-	The K+ Uptake Permease (KUP) Family
PAXXG073180	unmapped	scaffold34	1020843	1033619	+	The K+ Uptake Permease (KUP) Family
PAXXG128650	unmapped	scaffold86	1391188	1395352	+	The K+ Uptake Permease (KUP) Family
PAXXG132400	Chr13	scaffold90	615979	620367	+	The K+ Uptake Permease (KUP) Family
PAXXG174790	Chr19	scaffold151	1002452	1009954	+	The K+ Uptake Permease (KUP) Family
PAXXG219640	Chr13	scaffold253	157101	189415	+	The K+ Uptake Permease (KUP) Family
PAXXG240100	Chr18	scaffold311	372480	428043	-	The K+ Uptake Permease (KUP) Family
PAXXG245410	Chr11	scaffold326	135205	146315	+	The K+ Uptake Permease (KUP) Family
PAXXG248205	unmapped	scaffold335	234927	238057	-	The K+ Uptake Permease (KUP) Family
PAXXG255760	Chr19	scaffold361	283457	289903	-	The K+ Uptake Permease (KUP) Family
PAXXG271980	Chr01	scaffold427	164286	177826	+	The K+ Uptake Permease (KUP) Family
PAXXG295810	Chr10	scaffold552	307372	361427	-	The K+ Uptake Permease (KUP) Family
PAXXG368220	unmapped	scaffold2011	44476	44724	+	The K+ Uptake Permease (KUP) Family
PAXXG041000	Chr07	scaffold15	2920777	2930162	+	The Lysosomal Cystine Transporter (LCT) Family
PAXXG052110	Chr07	scaffold20	3918589	3940968	+	The Lysosomal Cystine Transporter (LCT) Family
PAXXG071340	Chr02	scaffold33	257788	318509	-	The Lysosomal Cystine Transporter (LCT) Family
PAXXG086840	Chr03	scaffold44	651103	674613	-	The Lysosomal Cystine Transporter (LCT) Family
PAXXG253000	unmapped	scaffold351	252599	253576	+	The Magnesium Transporter1 (MagT1) Family
PAXXG260270	unmapped	scaffold376	82864	84409	-	The Magnesium Transporter1 (MagT1) Family
PAXXG000480	Chr02	scaffold1	815181	903273	+	The Major Facilitator Superfamily (MFS)
PAXXG002140	Chr02	scaffold1	4369852	4424889	-	The Major Facilitator Superfamily (MFS)

PAXXG017320	Chr18	scaffold5	740696	752205	-	The Major Facilitator Superfamily (MFS)
PAXXG018900	Chr18	scaffold5	3920123	3933365	-	The Major Facilitator Superfamily (MFS)
PAXXG020040	Chr04	scaffold6	1251011	1322506	-	The Major Facilitator Superfamily (MFS)
PAXXG022790	Chr11	scaffold7	2490942	2494001	-	The Major Facilitator Superfamily (MFS)
PAXXG024430	Chr11	scaffold8	1649633	1766707	-	The Major Facilitator Superfamily (MFS)
PAXXG025950	Chr13	scaffold9	609395	610975	-	The Major Facilitator Superfamily (MFS)
PAXXG025960	Chr13	scaffold9	624481	626106	-	The Major Facilitator Superfamily (MFS)
PAXXG025970	Chr13	scaffold9	631163	640058	-	The Major Facilitator Superfamily (MFS)
PAXXG026040	Chr13	scaffold9	711344	712915	+	The Major Facilitator Superfamily (MFS)
PAXXG028090	Chr14	scaffold10	377156	381841	+	The Major Facilitator Superfamily (MFS)
PAXXG028640	Chr14	scaffold10	1075360	1089070	+	The Major Facilitator Superfamily (MFS)
PAXXG028840	Chr14	scaffold10	1347010	1349230	+	The Major Facilitator Superfamily (MFS)
PAXXG032380	Chr10	scaffold11	3883196	3901469	-	The Major Facilitator Superfamily (MFS)
PAXXG035160	Chr04	scaffold12	4454379	4471735	+	The Major Facilitator Superfamily (MFS)
PAXXG035180	Chr04	scaffold12	4484749	4488757	+	The Major Facilitator Superfamily (MFS)
PAXXG037440	Chr15	scaffold14	96083	103426	-	The Major Facilitator Superfamily (MFS)
PAXXG045340	Chr12*	scaffold17	3618166	3627804	+	The Major Facilitator Superfamily (MFS)
PAXXG045690	Chr12*	scaffold17	4124550	4132264	-	The Major Facilitator Superfamily (MFS)
PAXXG047820	Chr17	scaffold18	3750977	3757844	-	The Major Facilitator Superfamily (MFS)
PAXXG050350	Chr07	scaffold20	838867	841570	-	The Major Facilitator Superfamily (MFS)
PAXXG052220	Chr18	scaffold21	185230	238518	+	The Major Facilitator Superfamily (MFS)
PAXXG054550	Chr15	scaffold22	1436534	1443624	+	The Major Facilitator Superfamily (MFS)
PAXXG056430	Chr01	scaffold23	2203136	2204908	+	The Major Facilitator Superfamily (MFS)
PAXXG056450	Chr01	scaffold23	2211168	2212706	-	The Major Facilitator Superfamily (MFS)
PAXXG056640	Chr01	scaffold23	2516975	2522021	-	The Major Facilitator Superfamily (MFS)
PAXXG058340	Chr13	scaffold24	2695861	2747362	-	The Major Facilitator Superfamily (MFS)
PAXXG061030	Chr15	scaffold26	1523782	1545331	-	The Major Facilitator Superfamily (MFS)
PAXXG062140	Chr08	scaffold27	22901	41035	+	The Major Facilitator Superfamily (MFS)
PAXXG064160	Chr01	scaffold28	445111	477571	+	The Major Facilitator Superfamily (MFS)
PAXXG064510	Chr01	scaffold28	1050753	1053014	-	The Major Facilitator Superfamily (MFS)
PAXXG071210	Chr01	scaffold32	3133101	3143142	-	The Major Facilitator Superfamily (MFS)
PAXXG081060	Chr04	scaffold39	411015	418770	+	The Major Facilitator Superfamily (MFS)
PAXXG082900	Chr03	scaffold40	1318445	1327516	+	The Major Facilitator Superfamily (MFS)
PAXXG086270	Chr18	scaffold43	1896422	1898513	+	The Major Facilitator Superfamily (MFS)
PAXXG090300	Chr04	scaffold46	2992900	3009006	+	The Major Facilitator Superfamily (MFS)
PAXXG094060	Chr03	scaffold50	1721154	1723014	+	The Major Facilitator Superfamily (MFS)
PAXXG099350	Chr03	scaffold56	844995	846327	-	The Major Facilitator Superfamily (MFS)
PAXXG100850	Chr09	scaffold57	1829452	1831377	-	The Major Facilitator Superfamily (MFS)
PAXXG100880	Chr09	scaffold57	1837561	1839600	-	The Major Facilitator Superfamily (MFS)
PAXXG110550	Chr14*	scaffold67	953151	960997	-	The Major Facilitator Superfamily (MFS)
PAXXG122270	Chr07*	scaffold79	992766	1013823	+	The Major Facilitator Superfamily (MFS)
PAXXG122630	Chr07*	scaffold79	1554159	1557255	-	The Major Facilitator Superfamily (MFS)
PAXXG123860	Chr15	scaffold81	733478	736761	+	The Major Facilitator Superfamily (MFS)
PAXXG140110	Chr17	scaffold98	2852123	2889638	-	The Major Facilitator Superfamily (MFS)
PAXXG141590	Chr02	scaffold100	942463	945403	+	The Major Facilitator Superfamily (MFS)
PAXXG143900	unmapped	scaffold104	173958	179384	+	The Major Facilitator Superfamily (MFS)
PAXXG147360	Chr01	scaffold108	901524	914601	-	The Major Facilitator Superfamily (MFS)
PAXXG150890	Chr09	scaffold114	713733	733217	+	The Major Facilitator Superfamily (MFS)
PAXXG152570	Chr10	scaffold117	676350	682560	-	The Major Facilitator Superfamily (MFS)
PAXXG156930	Chr09	scaffold122	1373056	1375977	+	The Major Facilitator Superfamily (MFS)
PAXXG159480	Chr13	scaffold126	603144	604853	+	The Major Facilitator Superfamily (MFS)
PAXXG163870	Chr03	scaffold132	1041776	1042869	-	The Major Facilitator Superfamily (MFS)
PAXXG168320	Chr17	scaffold140	1151119	1154508	+	The Major Facilitator Superfamily (MFS)
PAXXG183140	Chr02	scaffold167	1065174	1110563	-	The Major Facilitator Superfamily (MFS)
PAXXG183720	Chr06	scaffold169	8789	46661	+	The Major Facilitator Superfamily (MFS)
PAXXG186090	Chr17	scaffold173	9031	13412	+	The Major Facilitator Superfamily (MFS)
PAXXG200065	Chr19	scaffold201	105936	107176	-	The Major Facilitator Superfamily (MFS)
PAXXG200380	Chr19	scaffold201	656693	659748	-	The Major Facilitator Superfamily (MFS)
PAXXG202770	Chr09	scaffold206	919100	927067	+	The Major Facilitator Superfamily (MFS)
PAXXG202810	Chr09	scaffold206	962403	965599	-	The Major Facilitator Superfamily (MFS)
PAXXG211970	unmapped	scaffold231	207002	210516	+	The Major Facilitator Superfamily (MFS)
PAXXG212060	unmapped	scaffold231	584459	589803	+	The Major Facilitator Superfamily (MFS)
PAXXG214080	Chr13	scaffold237	887972	911177	+	The Major Facilitator Superfamily (MFS)
PAXXG220770	Chr01	scaffold255	107612	118038	+	The Major Facilitator Superfamily (MFS)
PAXXG228320	unmapped	scaffold274	605752	621707	-	The Major Facilitator Superfamily (MFS)
PAXXG230670	unmapped	scaffold281	647360	664129	-	The Major Facilitator Superfamily (MFS)
PAXXG237290	unmapped	scaffold302	469248	517083	-	The Major Facilitator Superfamily (MFS)
PAXXG245570	Chr11	scaffold326	619491	621996	+	The Major Facilitator Superfamily (MFS)
PAXXG245580	Chr11	scaffold326	626434	634041	+	The Major Facilitator Superfamily (MFS)
PAXXG245590	Chr11	scaffold326	714581	716594	-	The Major Facilitator Superfamily (MFS)
PAXXG270300	Chr18	scaffold420	172526	178846	+	The Major Facilitator Superfamily (MFS)
PAXXG303040	Chr17	scaffold595	348158	348767	+	The Major Facilitator Superfamily (MFS)
PAXXG304270	unmapped	scaffold604	313500	323850	-	The Major Facilitator Superfamily (MFS)
PAXXG317270	unmapped	scaffold703	251820	252311	-	The Major Facilitator Superfamily (MFS)
PAXXG321030	Chr10	scaffold734	133428	137441	-	The Major Facilitator Superfamily (MFS)
PAXXG324230	Chr16	scaffold766	192062	196699	+	The Major Facilitator Superfamily (MFS)
PAXXG333620	Chr10	scaffold866	215109	242126	+	The Major Facilitator Superfamily (MFS)
PAXXG353150	unmapped	scaffold1226	36825	39910	+	The Major Facilitator Superfamily (MFS)
PAXXG371950	unmapped	scaffold2440	24275	31087	+	The Major Facilitator Superfamily (MFS)
PAXXG384530	unmapped	scaffold5467	4980	6112	+	The Major Facilitator Superfamily (MFS)
PAXXG029880	Chr14	scaffold10	3288861	3292369	+	The Major Intrinsic Protein (MIP) Family
PAXXG033790	Chr04	scaffold12	2020149	2023291	+	The Major Intrinsic Protein (MIP) Family
PAXXG042870	Chr19	scaffold16	1885280	1887376	+	The Major Intrinsic Protein (MIP) Family
PAXXG099330	Chr03	scaffold56	813914	825874	-	The Major Intrinsic Protein (MIP) Family
PAXXG114540	Chr09	scaffold70	1481681	1482583	+	The Major Intrinsic Protein (MIP) Family
PAXXG123140	Chr01	scaffold80	958888	962914	-	The Major Intrinsic Protein (MIP) Family

PAXXG124650	Chr04	scaffold82	240636	243267	+	The Major Intrinsic Protein (MIP) Family
PAXXG141530	Chr02	scaffold100	892906	907740	+	The Major Intrinsic Protein (MIP) Family
PAXXG178700	Chr09	scaffold160	656031	658737	+	The Major Intrinsic Protein (MIP) Family
PAXXG183100	Chr02	scaffold167	1037953	1038876	+	The Major Intrinsic Protein (MIP) Family
PAXXG183150	Chr02	scaffold167	1112127	1116612	-	The Major Intrinsic Protein (MIP) Family
PAXXG195170	Chr08	scaffold191	782128	789274	-	The Major Intrinsic Protein (MIP) Family
PAXXG195180	Chr08	scaffold191	795632	819271	-	The Major Intrinsic Protein (MIP) Family
PAXXG255850	Chr19	scaffold361	463422	463928	+	The Major Intrinsic Protein (MIP) Family
PAXXG283880	unmapped	scaffold482	196522	198466	+	The Major Intrinsic Protein (MIP) Family
PAXXG285680	Chr07	scaffold493	48037	49994	-	The Major Intrinsic Protein (MIP) Family
PAXXG285980	unmapped	scaffold494	201325	204686	+	The Major Intrinsic Protein (MIP) Family
PAXXG288200	unmapped	scaffold507	166255	168023	+	The Major Intrinsic Protein (MIP) Family
PAXXG288240	unmapped	scaffold507	242442	245029	+	The Major Intrinsic Protein (MIP) Family
PAXXG292520	unmapped	scaffold531	235379	255399	-	The Major Intrinsic Protein (MIP) Family
PAXXG301810	unmapped	scaffold589	28195	34667	-	The Major Intrinsic Protein (MIP) Family
PAXXG301820	unmapped	scaffold589	53306	54861	+	The Major Intrinsic Protein (MIP) Family
PAXXG307390	Chr06	scaffold626	271574	273838	+	The Major Intrinsic Protein (MIP) Family
PAXXG313260	Chr18	scaffold667	230531	232145	-	The Major Intrinsic Protein (MIP) Family
PAXXG313270	Chr18	scaffold667	258550	260185	-	The Major Intrinsic Protein (MIP) Family
PAXXG333600	Chr10	scaffold866	143929	156446	+	The Major Intrinsic Protein (MIP) Family
PAXXG381520	unmapped	scaffold4469	636	2304	+	The Major Intrinsic Protein (MIP) Family
PAXXG027410	Chr13	scaffold9	3766886	3773004	+	The Metal Ion (Mn2+-iron) Transporter (Nramp) Family
PAXXG053980	Chr15	scaffold22	245825	264932	-	The Metal Ion (Mn2+-iron) Transporter (Nramp) Family
PAXXG079540	Chr06	scaffold38	619448	631379	-	The Metal Ion (Mn2+-iron) Transporter (Nramp) Family
PAXXG166780	Chr05	scaffold137	1268782	1283606	-	The Metal Ion (Mn2+-iron) Transporter (Nramp) Family
PAXXG198160	unmapped	scaffold197	1027731	1079184	+	The Metal Ion (Mn2+-iron) Transporter (Nramp) Family
PAXXG344180	Chr01	scaffold1029	65645	71988	-	The Metal Ion (Mn2+-iron) Transporter (Nramp) Family
PAXXG004470	Chr10	scaffold2	1722107	1725185	+	The Mitochondrial Carrier (MC) Family
PAXXG004710	Chr10	scaffold2	2510458	2514563	-	The Mitochondrial Carrier (MC) Family
PAXXG015370	Chr15	scaffold4	4959468	4961345	+	The Mitochondrial Carrier (MC) Family
PAXXG015620	Chr15	scaffold4	5419506	5420848	+	The Mitochondrial Carrier (MC) Family
PAXXG016070	Chr15	scaffold4	6067860	6078306	-	The Mitochondrial Carrier (MC) Family
PAXXG024250	Chr11	scaffold8	1163633	1177068	+	The Mitochondrial Carrier (MC) Family
PAXXG031860	Chr10	scaffold11	2747376	2795520	-	The Mitochondrial Carrier (MC) Family
PAXXG038630	Chr15	scaffold14	2403191	2428338	-	The Mitochondrial Carrier (MC) Family
PAXXG038820	Chr15	scaffold14	2846744	2855967	+	The Mitochondrial Carrier (MC) Family
PAXXG044550	Chr12*	scaffold17	1980144	1981796	-	The Mitochondrial Carrier (MC) Family
PAXXG046890	Chr17	scaffold18	2424922	2446773	+	The Mitochondrial Carrier (MC) Family
PAXXG047010	Chr17	scaffold18	2659952	2694285	-	The Mitochondrial Carrier (MC) Family
PAXXG057320	Chr13	scaffold24	791453	806175	+	The Mitochondrial Carrier (MC) Family
PAXXG073250	unmapped	scaffold34	1141562	1163030	+	The Mitochondrial Carrier (MC) Family
PAXXG073440	unmapped	scaffold34	1463938	1496317	+	The Mitochondrial Carrier (MC) Family
PAXXG073510	unmapped	scaffold34	1609275	1624713	+	The Mitochondrial Carrier (MC) Family
PAXXG074470	Chr11	scaffold35	283882	318378	+	The Mitochondrial Carrier (MC) Family
PAXXG079860	Chr06	scaffold38	1011396	1029251	-	The Mitochondrial Carrier (MC) Family
PAXXG082330	Chr03	scaffold40	173652	184803	-	The Mitochondrial Carrier (MC) Family
PAXXG097160	Chr04	scaffold54	434551	451483	+	The Mitochondrial Carrier (MC) Family
PAXXG103440	Chr12	scaffold59	1109528	1114817	+	The Mitochondrial Carrier (MC) Family
PAXXG111440	Chr08*	scaffold68	110167	111774	-	The Mitochondrial Carrier (MC) Family
PAXXG113760	Chr09	scaffold70	387126	401042	+	The Mitochondrial Carrier (MC) Family
PAXXG130320	unmapped	scaffold88	771014	803762	-	The Mitochondrial Carrier (MC) Family
PAXXG130700	Chr11	scaffold89	81417	102850	+	The Mitochondrial Carrier (MC) Family
PAXXG140240	Chr17	scaffold98	3060159	3078364	+	The Mitochondrial Carrier (MC) Family
PAXXG148900	Chr07	scaffold111	177831	239973	+	The Mitochondrial Carrier (MC) Family
PAXXG150630	Chr09	scaffold114	202032	215911	-	The Mitochondrial Carrier (MC) Family
PAXXG171420	unmapped	scaffold146	118720	147027	+	The Mitochondrial Carrier (MC) Family
PAXXG173160	Chr08	scaffold149	486835	492096	-	The Mitochondrial Carrier (MC) Family
PAXXG194820	Chr08	scaffold191	255307	256898	+	The Mitochondrial Carrier (MC) Family
PAXXG200680	Chr11	scaffold202	681191	701075	-	The Mitochondrial Carrier (MC) Family
PAXXG221630	Chr16	scaffold257	36577	46310	-	The Mitochondrial Carrier (MC) Family
PAXXG226100	Chr08	scaffold269	138483	152851	+	The Mitochondrial Carrier (MC) Family
PAXXG227600	Chr06	scaffold272	330285	332000	-	The Mitochondrial Carrier (MC) Family
PAXXG228480	unmapped	scaffold274	891189	894517	-	The Mitochondrial Carrier (MC) Family
PAXXG230740	Chr11	scaffold282	404534	435855	+	The Mitochondrial Carrier (MC) Family
PAXXG240000	Chr18	scaffold311	259113	277969	+	The Mitochondrial Carrier (MC) Family
PAXXG240070	Chr18	scaffold311	339879	344853	-	The Mitochondrial Carrier (MC) Family
PAXXG242860	Chr12	scaffold320	195978	209244	+	The Mitochondrial Carrier (MC) Family
PAXXG261920	Chr16	scaffold383	487431	529568	-	The Mitochondrial Carrier (MC) Family
PAXXG289090	Chr03	scaffold511	422412	429020	-	The Mitochondrial Carrier (MC) Family
PAXXG305590	Chr10	scaffold612	242542	274560	-	The Mitochondrial Carrier (MC) Family
PAXXG320160	unmapped	scaffold727	199614	215911	+	The Mitochondrial Carrier (MC) Family
PAXXG322770	unmapped	scaffold751	52391	112749	+	The Mitochondrial Carrier (MC) Family
PAXXG334530	unmapped	scaffold876	158611	175156	+	The Mitochondrial Carrier (MC) Family
PAXXG338450	unmapped	scaffold932	158833	191979	-	The Mitochondrial Carrier (MC) Family
PAXXG341790	unmapped	scaffold983	151621	162235	+	The Mitochondrial Carrier (MC) Family
PAXXG345230	unmapped	scaffold1049	99496	110532	+	The Mitochondrial Carrier (MC) Family
PAXXG363450	unmapped	scaffold1674	46669	60560	-	The Mitochondrial Carrier (MC) Family
PAXXG363960	unmapped	scaffold1701	57287	58599	-	The Mitochondrial Carrier (MC) Family
PAXXG017680	Chr18	scaffold5	1509147	1513718	-	The Mitochondrial Protein Translocase (MPT) Family
PAXXG025360	Chr11	scaffold8	4120412	4144216	+	The Mitochondrial Protein Translocase (MPT) Family
PAXXG026060	Chr13	scaffold9	750073	778143	+	The Mitochondrial Protein Translocase (MPT) Family
PAXXG026830	Chr13	scaffold9	2414968	2420356	-	The Mitochondrial Protein Translocase (MPT) Family
PAXXG042040	Chr19	scaffold16	462465	480612	-	The Mitochondrial Protein Translocase (MPT) Family
PAXXG050190	Chr07	scaffold20	527108	556447	-	The Mitochondrial Protein Translocase (MPT) Family
PAXXG060030	Chr04	scaffold25	2792903	2817468	+	The Mitochondrial Protein Translocase (MPT) Family
PAXXG063180	Chr08	scaffold27	2055947	2061167	-	The Mitochondrial Protein Translocase (MPT) Family

PAXXG073590	unmapped	scaffold34	1746493	1754059	+	The Mitochondrial Protein Translocase (MPT) Family
PAXXG077360	Chr10	scaffold37	313894	350085	+	The Mitochondrial Protein Translocase (MPT) Family
PAXXG083400	Chr03	scaffold40	2402393	2403652	+	The Mitochondrial Protein Translocase (MPT) Family
PAXXG084570	Chr03	scaffold42	10342	64220	-	The Mitochondrial Protein Translocase (MPT) Family
PAXXG097850	Chr04	scaffold54	2082034	2091608	-	The Mitochondrial Protein Translocase (MPT) Family
PAXXG102330	Chr16	scaffold58	1556613	1582294	+	The Mitochondrial Protein Translocase (MPT) Family
PAXXG111740	Chr08*	scaffold68	912364	942096	-	The Mitochondrial Protein Translocase (MPT) Family
PAXXG124460	Chr15	scaffold81	1895024	1896180	+	The Mitochondrial Protein Translocase (MPT) Family
PAXXG128700	unmapped	scaffold86	1708890	1730832	-	The Mitochondrial Protein Translocase (MPT) Family
PAXXG134320	Chr03	scaffold92	1745555	1756347	+	The Mitochondrial Protein Translocase (MPT) Family
PAXXG148520	Chr09	scaffold110	944636	961890	-	The Mitochondrial Protein Translocase (MPT) Family
PAXXG171240	Chr18	scaffold145	1086801	1091835	-	The Mitochondrial Protein Translocase (MPT) Family
PAXXG182660	Chr02	scaffold167	230565	244453	-	The Mitochondrial Protein Translocase (MPT) Family
PAXXG189720	Chr15	scaffold180	634860	657621	-	The Mitochondrial Protein Translocase (MPT) Family
PAXXG235250	Chr16	scaffold296	177353	242546	+	The Mitochondrial Protein Translocase (MPT) Family
PAXXG246120	Chr10	scaffold328	292100	312003	-	The Mitochondrial Protein Translocase (MPT) Family
PAXXG255630	Chr10	scaffold360	616622	617448	+	The Mitochondrial Protein Translocase (MPT) Family
PAXXG255640	Chr10	scaffold360	624134	642027	-	The Mitochondrial Protein Translocase (MPT) Family
PAXXG259840	Chr10	scaffold374	499352	519514	-	The Mitochondrial Protein Translocase (MPT) Family
PAXXG318070	unmapped	scaffold712	196086	242795	+	The Mitochondrial Protein Translocase (MPT) Family
PAXXG339480	unmapped	scaffold946	104105	144155	-	The Mitochondrial Protein Translocase (MPT) Family
PAXXG036850	Chr16	scaffold13	3364684	3374686	-	The Monovalent Cation:Proton Antiporter (CPA1 or CPA2) Family
PAXXG047080	Chr17	scaffold18	2754602	2757174	+	The Monovalent Cation:Proton Antiporter (CPA1 or CPA2) Family
PAXXG085130	Chr03	scaffold42	1670522	1681344	-	The Monovalent Cation:Proton Antiporter (CPA1 or CPA2) Family
PAXXG154470	Chr10	scaffold119	1535597	1539530	+	The Monovalent Cation:Proton Antiporter (CPA1 or CPA2) Family
PAXXG025270	Chr11	scaffold8	3831530	3924306	-	The Monovalent Cation:Proton Antiporter-1 (CPA1) Family
PAXXG068700	Chr03	scaffold31	128106	139626	-	The Monovalent Cation:Proton Antiporter-1 (CPA1) Family
PAXXG134980	Chr16	scaffold93	1624691	1678993	+	The Monovalent Cation:Proton Antiporter-1 (CPA1) Family
PAXXG162220	Chr01	scaffold130	462863	642264	-	The Monovalent Cation:Proton Antiporter-1 (CPA1) Family
PAXXG188850	Chr10	scaffold179	309025	409918	+	The Monovalent Cation:Proton Antiporter-1 (CPA1) Family
PAXXG259010	unmapped	scaffold372	59361	82018	+	The Monovalent Cation:Proton Antiporter-1 (CPA1) Family
PAXXG312170	unmapped	scaffold659	73979	110896	-	The Monovalent Cation:Proton Antiporter-1 (CPA1) Family
PAXXG000160	Chr02	scaffold1	398400	427473	+	The Monovalent Cation:Proton Antiporter-2 (CPA2) Family
PAXXG001430	Chr02	scaffold1	2889588	2893706	+	The Monovalent Cation:Proton Antiporter-2 (CPA2) Family
PAXXG030850	Chr10	scaffold11	714700	717912	-	The Monovalent Cation:Proton Antiporter-2 (CPA2) Family
PAXXG049990	Chr07	scaffold20	202750	205670	+	The Monovalent Cation:Proton Antiporter-2 (CPA2) Family
PAXXG061580	Chr15	scaffold26	2651790	2659097	+	The Monovalent Cation:Proton Antiporter-2 (CPA2) Family
PAXXG076090	Chr11	scaffold35	2832780	2885639	+	The Monovalent Cation:Proton Antiporter-2 (CPA2) Family
PAXXG077620	Chr10	scaffold37	805697	808918	+	The Monovalent Cation:Proton Antiporter-2 (CPA2) Family
PAXXG124120	Chr15	scaffold81	1206478	1252524	+	The Monovalent Cation:Proton Antiporter-2 (CPA2) Family
PAXXG139100	Chr17	scaffold98	1460669	1469705	+	The Monovalent Cation:Proton Antiporter-2 (CPA2) Family
PAXXG186540	Chr17	scaffold173	1094314	1098254	+	The Monovalent Cation:Proton Antiporter-2 (CPA2) Family
PAXXG225520	unmapped	scaffold267	222907	230846	+	The Monovalent Cation:Proton Antiporter-2 (CPA2) Family
PAXXG252150	Chr11	scaffold347	962206	966140	-	The Monovalent Cation:Proton Antiporter-2 (CPA2) Family
PAXXG324050	unmapped	scaffold765	9680	65962	-	The Monovalent Cation:Proton Antiporter-2 (CPA2) Family
PAXXG000770	Chr02	scaffold1	1281742	1297724	+	The Multidrug/Oligosaccharidyl-lipid/Polysaccharide (MOP) Flippase Superfamily
PAXXG002250	Chr02	scaffold1	4673232	4674998	-	The Multidrug/Oligosaccharidyl-lipid/Polysaccharide (MOP) Flippase Superfamily
PAXXG027350	Chr13	scaffold9	3690089	3691871	-	The Multidrug/Oligosaccharidyl-lipid/Polysaccharide (MOP) Flippase Superfamily
PAXXG027770	Chr14	scaffold10	47871	49674	+	The Multidrug/Oligosaccharidyl-lipid/Polysaccharide (MOP) Flippase Superfamily
PAXXG042250	Chr19	scaffold16	821449	874092	-	The Multidrug/Oligosaccharidyl-lipid/Polysaccharide (MOP) Flippase Superfamily
PAXXG050090	Chr07	scaffold20	366584	378964	+	The Multidrug/Oligosaccharidyl-lipid/Polysaccharide (MOP) Flippase Superfamily
PAXXG076620	Chr02	scaffold36	1371627	1409000	-	The Multidrug/Oligosaccharidyl-lipid/Polysaccharide (MOP) Flippase Superfamily
PAXXG080360	Chr06	scaffold38	1832510	1834153	-	The Multidrug/Oligosaccharidyl-lipid/Polysaccharide (MOP) Flippase Superfamily
PAXXG089440	Chr04	scaffold46	1463978	1474508	+	The Multidrug/Oligosaccharidyl-lipid/Polysaccharide (MOP) Flippase Superfamily
PAXXG095660	Chr02	scaffold52	747706	751531	+	The Multidrug/Oligosaccharidyl-lipid/Polysaccharide (MOP) Flippase Superfamily
PAXXG099390	Chr03	scaffold56	978044	980701	+	The Multidrug/Oligosaccharidyl-lipid/Polysaccharide (MOP) Flippase Superfamily
PAXXG111230	Chr14*	scaffold67	1894195	1914954	-	The Multidrug/Oligosaccharidyl-lipid/Polysaccharide (MOP) Flippase Superfamily
PAXXG112640	Chr19	scaffold69	525968	532068	-	The Multidrug/Oligosaccharidyl-lipid/Polysaccharide (MOP) Flippase Superfamily
PAXXG120290	Chr11	scaffold76	1878141	1878903	-	The Multidrug/Oligosaccharidyl-lipid/Polysaccharide (MOP) Flippase Superfamily
PAXXG124850	Chr04	scaffold82	535780	546364	-	The Multidrug/Oligosaccharidyl-lipid/Polysaccharide (MOP) Flippase Superfamily
PAXXG126130	Chr16	scaffold83	1198598	1203014	+	The Multidrug/Oligosaccharidyl-lipid/Polysaccharide (MOP) Flippase Superfamily
PAXXG137080	Chr03	scaffold96	716502	718113	+	The Multidrug/Oligosaccharidyl-lipid/Polysaccharide (MOP) Flippase Superfamily
PAXXG137330	Chr03	scaffold96	1196899	1199080	+	The Multidrug/Oligosaccharidyl-lipid/Polysaccharide (MOP) Flippase Superfamily
PAXXG163970	Chr03	scaffold132	1392107	1428556	-	The Multidrug/Oligosaccharidyl-lipid/Polysaccharide (MOP) Flippase Superfamily
PAXXG171680	unmapped	scaffold146	1275606	1277587	+	The Multidrug/Oligosaccharidyl-lipid/Polysaccharide (MOP) Flippase Superfamily
PAXXG183060	Chr02	scaffold167	942837	961145	-	The Multidrug/Oligosaccharidyl-lipid/Polysaccharide (MOP) Flippase Superfamily
PAXXG184950	Chr09	scaffold171	317425	319095	+	The Multidrug/Oligosaccharidyl-lipid/Polysaccharide (MOP) Flippase Superfamily
PAXXG188170	Chr10	scaffold177	470918	478140	-	The Multidrug/Oligosaccharidyl-lipid/Polysaccharide (MOP) Flippase Superfamily
PAXXG191950	Chr17	scaffold185	820632	828693	-	The Multidrug/Oligosaccharidyl-lipid/Polysaccharide (MOP) Flippase Superfamily
PAXXG223340	Chr06	scaffold260	821373	829164	-	The Multidrug/Oligosaccharidyl-lipid/Polysaccharide (MOP) Flippase Superfamily
PAXXG231390	unmapped	scaffold284	560210	561736	+	The Multidrug/Oligosaccharidyl-lipid/Polysaccharide (MOP) Flippase Superfamily
PAXXG260070	Chr10	scaffold375	231523	235871	+	The Multidrug/Oligosaccharidyl-lipid/Polysaccharide (MOP) Flippase Superfamily
PAXXG319550	unmapped	scaffold723	49048	55538	+	The Multidrug/Oligosaccharidyl-lipid/Polysaccharide (MOP) Flippase Superfamily
PAXXG319600	unmapped	scaffold723	226497	300509	+	The Multidrug/Oligosaccharidyl-lipid/Polysaccharide (MOP) Flippase Superfamily
PAXXG319630	unmapped	scaffold723	314787	319372	+	The Multidrug/Oligosaccharidyl-lipid/Polysaccharide (MOP) Flippase Superfamily
PAXXG326330	unmapped	scaffold783	332775	352760	+	The Multidrug/Oligosaccharidyl-lipid/Polysaccharide (MOP) Flippase Superfamily
PAXXG353580	unmapped	scaffold1242	84772	87260	+	The Multidrug/Oligosaccharidyl-lipid/Polysaccharide (MOP) Flippase Superfamily
PAXXG145550	Chr11	scaffold105	1379301	1392138	+	The NhaD Na+:H+ Antiporter (NhaD) Family
PAXXG306570	Chr09	scaffold619	252993	271492	+	The Ni2+-Co2+ Transporter (NiCoT) Family
PAXXG007870	Chr10	scaffold2	9040163	9068527	+	The NIPA Mg2+ Uptake Permease (NIPA) Family
PAXXG035660	Chr16	scaffold13	1016272	1038922	+	The NIPA Mg2+ Uptake Permease (NIPA) Family
PAXXG041280	Chr07	scaffold15	3383742	3393777	-	The NIPA Mg2+ Uptake Permease (NIPA) Family
PAXXG053400	Chr18	scaffold21	2736087	2753953	-	The NIPA Mg2+ Uptake Permease (NIPA) Family
PAXXG141490	Chr02	scaffold100	835125	842838	-	The NIPA Mg2+ Uptake Permease (NIPA) Family
PAXXG158130	unmapped	scaffold124	1496438	1508188	-	The NIPA Mg2+ Uptake Permease (NIPA) Family
PAXXG178210	Chr19	scaffold159	438465	449315	+	The NIPA Mg2+ Uptake Permease (NIPA) Family

PAXXG050030	Chr07	scaffold20	233290	267325	+	The Non-selective Cation Channel-2 (NSCC2) Family
PAXXG179890	Chr07	scaffold162	669994	704387	-	The Non-selective Cation Channel-2 (NSCC2) Family
PAXXG221070	Chr01	scaffold255	651665	652950	-	The Nucleobase:Cation Symporter-1 (NCS1) Family
PAXXG304160	unmapped	scaffold604	82725	84386	+	The Nucleobase:Cation Symporter-1 (NCS1) Family
PAXXG019390	Chr18	scaffold5	5057217	5067570	-	The Nucleobase:Cation Symporter-2 (NCS2) Family
PAXXG036700	Chr16	scaffold13	3045762	3077806	-	The Nucleobase:Cation Symporter-2 (NCS2) Family
PAXXG155370	Chr10	scaffold121	615679	621428	-	The Nucleobase:Cation Symporter-2 (NCS2) Family
PAXXG256480	Chr09	scaffold364	59868	106480	+	The Nucleobase:Cation Symporter-2 (NCS2) Family
PAXXG291450	Chr07	scaffold524	315355	337726	-	The Nucleobase:Cation Symporter-2 (NCS2) Family
PAXXG295800	Chr10	scaffold552	299629	301487	-	The Nucleobase:Cation Symporter-2 (NCS2) Family
PAXXG298600	Chr06	scaffold569	421087	422854	-	The Nucleobase:Cation Symporter-2 (NCS2) Family
PAXXG007450	Chr10	scaffold2	8229254	8231793	+	The Oligopeptide Transporter (OPT) Family
PAXXG007480	Chr10	scaffold2	8276314	8280340	-	The Oligopeptide Transporter (OPT) Family
PAXXG025340	Chr11	scaffold8	4058365	4062278	-	The Oligopeptide Transporter (OPT) Family
PAXXG025350	Chr11	scaffold8	4107610	4110606	+	The Oligopeptide Transporter (OPT) Family
PAXXG051510	Chr07	scaffold20	2713507	2716818	+	The Oligopeptide Transporter (OPT) Family
PAXXG062900	Chr08	scaffold27	1496502	1502226	+	The Oligopeptide Transporter (OPT) Family
PAXXG065930	Chr04	scaffold29	1009618	1021254	+	The Oligopeptide Transporter (OPT) Family
PAXXG070770	Chr01	scaffold32	1851273	1855438	-	The Oligopeptide Transporter (OPT) Family
PAXXG074260	Chr11	scaffold35	1840	6772	-	The Oligopeptide Transporter (OPT) Family
PAXXG094540	Chr09	scaffold51	452308	456282	-	The Oligopeptide Transporter (OPT) Family
PAXXG111770	Chr08*	scaffold68	1022953	1023417	-	The Oligopeptide Transporter (OPT) Family
PAXXG131540	Chr11	scaffold89	1524254	1529166	+	The Oligopeptide Transporter (OPT) Family
PAXXG159060	Chr10	scaffold125	1346152	1367615	+	The Oligopeptide Transporter (OPT) Family
PAXXG176460	Chr11	scaffold155	610559	616419	-	The Oligopeptide Transporter (OPT) Family
PAXXG247020	unmapped	scaffold331	375998	389615	+	The Oligopeptide Transporter (OPT) Family
PAXXG254010	Chr18	scaffold356	99381	102301	+	The Oligopeptide Transporter (OPT) Family
PAXXG254020	Chr18	scaffold356	102721	105835	+	The Oligopeptide Transporter (OPT) Family
PAXXG261520	unmapped	scaffold382	326234	326644	+	The Oligopeptide Transporter (OPT) Family
PAXXG261530	unmapped	scaffold382	326819	327318	+	The Oligopeptide Transporter (OPT) Family
PAXXG272260	unmapped	scaffold428	183053	188651	-	The Oligopeptide Transporter (OPT) Family
PAXXG318005	unmapped	scaffold710	247745	249821	+	The Oligopeptide Transporter (OPT) Family
PAXXG349400	Chr03	scaffold1132	38695	43978	+	The Oligopeptide Transporter (OPT) Family
PAXXG005450	Chr10	scaffold2	3803450	3837994	+	The Peroxisomal Protein Importer (PPI) Family
PAXXG044530	Chr12*	scaffold17	1907925	1956334	+	The Peroxisomal Protein Importer (PPI) Family
PAXXG058720	Chr13	scaffold24	3549071	3584755	-	The Peroxisomal Protein Importer (PPI) Family
PAXXG329940	unmapped	scaffold823	165455	228052	+	The Peroxisomal Protein Importer (PPI) Family
PAXXG332490	Chr06	scaffold853	221976	223836	+	The Peroxisomal Protein Importer (PPI) Family
PAXXG002690	Chr02	scaffold1	5774383	5776052	+	The Presenilin ER Ca2+ Leak Channel (Presenilin) Family
PAXXG030610	Chr10	scaffold11	185452	208800	-	The Presenilin ER Ca2+ Leak Channel (Presenilin) Family
PAXXG002450	Chr02	scaffold1	5163458	5168665	+	The Proton-dependent Oligopeptide Transporter (POT) Family
PAXXG007730	Chr10	scaffold2	8831185	8835380	+	The Proton-dependent Oligopeptide Transporter (POT) Family
PAXXG017350	Chr18	scaffold5	793579	798690	+	The Proton-dependent Oligopeptide Transporter (POT) Family
PAXXG019240	Chr18	scaffold5	4688037	4692002	-	The Proton-dependent Oligopeptide Transporter (POT) Family
PAXXG022560	Chr11	scaffold7	1931619	1939560	+	The Proton-dependent Oligopeptide Transporter (POT) Family
PAXXG028660	Chr14	scaffold10	1100800	1103963	-	The Proton-dependent Oligopeptide Transporter (POT) Family
PAXXG030960	Chr10	scaffold11	1017831	1027967	+	The Proton-dependent Oligopeptide Transporter (POT) Family
PAXXG035120	Chr04	scaffold12	4397320	4403913	+	The Proton-dependent Oligopeptide Transporter (POT) Family
PAXXG035890	Chr16	scaffold13	1635188	1652592	-	The Proton-dependent Oligopeptide Transporter (POT) Family
PAXXG035930	Chr16	scaffold13	1660724	1661362	-	The Proton-dependent Oligopeptide Transporter (POT) Family
PAXXG035950	Chr16	scaffold13	1678288	1690880	-	The Proton-dependent Oligopeptide Transporter (POT) Family
PAXXG035960	Chr16	scaffold13	1707716	1713532	-	The Proton-dependent Oligopeptide Transporter (POT) Family
PAXXG035970	Chr16	scaffold13	1716288	1727417	-	The Proton-dependent Oligopeptide Transporter (POT) Family
PAXXG035980	Chr16	scaffold13	1740516	1749366	-	The Proton-dependent Oligopeptide Transporter (POT) Family
PAXXG044820	Chr12*	scaffold17	2694427	2705635	-	The Proton-dependent Oligopeptide Transporter (POT) Family
PAXXG073480	unmapped	scaffold34	1535385	1544039	+	The Proton-dependent Oligopeptide Transporter (POT) Family
PAXXG074740	Chr11	scaffold35	745476	747615	+	The Proton-dependent Oligopeptide Transporter (POT) Family
PAXXG074750	Chr11	scaffold35	755524	757402	+	The Proton-dependent Oligopeptide Transporter (POT) Family
PAXXG074760	Chr11	scaffold35	763215	765207	+	The Proton-dependent Oligopeptide Transporter (POT) Family
PAXXG078890	Chr10	scaffold37	2428422	2432764	-	The Proton-dependent Oligopeptide Transporter (POT) Family
PAXXG085830	Chr18	scaffold43	845528	856044	-	The Proton-dependent Oligopeptide Transporter (POT) Family
PAXXG088450	Chr06	scaffold45	1976222	1978680	-	The Proton-dependent Oligopeptide Transporter (POT) Family
PAXXG090080	Chr04	scaffold46	2511541	2514835	-	The Proton-dependent Oligopeptide Transporter (POT) Family
PAXXG093700	Chr03	scaffold50	789536	798774	-	The Proton-dependent Oligopeptide Transporter (POT) Family
PAXXG107980	Chr03	scaffold64	922896	933143	-	The Proton-dependent Oligopeptide Transporter (POT) Family
PAXXG107990	Chr03	scaffold64	938725	946868	-	The Proton-dependent Oligopeptide Transporter (POT) Family
PAXXG108000	Chr03	scaffold64	988023	994595	-	The Proton-dependent Oligopeptide Transporter (POT) Family
PAXXG110670	Chr14*	scaffold67	1115612	1141757	+	The Proton-dependent Oligopeptide Transporter (POT) Family
PAXXG125510	Chr04	scaffold82	1805437	1810596	-	The Proton-dependent Oligopeptide Transporter (POT) Family
PAXXG138680	Chr17	scaffold98	764634	772873	+	The Proton-dependent Oligopeptide Transporter (POT) Family
PAXXG144230	unmapped	scaffold104	926162	928649	+	The Proton-dependent Oligopeptide Transporter (POT) Family
PAXXG146430	Chr13	scaffold106	1601941	1651919	-	The Proton-dependent Oligopeptide Transporter (POT) Family
PAXXG149340	Chr07	scaffold111	1205146	1206995	-	The Proton-dependent Oligopeptide Transporter (POT) Family
PAXXG149350	Chr07	scaffold111	1212783	1213539	-	The Proton-dependent Oligopeptide Transporter (POT) Family
PAXXG149360	Chr07	scaffold111	1215442	1229972	-	The Proton-dependent Oligopeptide Transporter (POT) Family
PAXXG186240	Chr17	scaffold173	360359	366992	+	The Proton-dependent Oligopeptide Transporter (POT) Family
PAXXG192700	unmapped	scaffold187	241848	248643	+	The Proton-dependent Oligopeptide Transporter (POT) Family
PAXXG217800	unmapped	scaffold247	755030	759847	-	The Proton-dependent Oligopeptide Transporter (POT) Family
PAXXG221600	Chr16	scaffold257	21791	24014	+	The Proton-dependent Oligopeptide Transporter (POT) Family
PAXXG221610	Chr16	scaffold257	27923	30247	+	The Proton-dependent Oligopeptide Transporter (POT) Family
PAXXG243220	Chr17	scaffold321	40866	42398	+	The Proton-dependent Oligopeptide Transporter (POT) Family
PAXXG243420	Chr17	scaffold321	538161	562907	-	The Proton-dependent Oligopeptide Transporter (POT) Family
PAXXG259870	Chr10	scaffold374	540350	542308	+	The Proton-dependent Oligopeptide Transporter (POT) Family
PAXXG259890	Chr10	scaffold374	551539	553500	+	The Proton-dependent Oligopeptide Transporter (POT) Family
PAXXG260530	unmapped	scaffold377	112739	133590	+	The Proton-dependent Oligopeptide Transporter (POT) Family
PAXXG260860	Chr14	scaffold378	571577	578612	-	The Proton-dependent Oligopeptide Transporter (POT) Family

PAXXG302110	Chr16	scaffold590	278229	278612	+	The Proton-dependent Oligopeptide Transporter (POT) Family
PAXXG318590	Chr16	scaffold715	232037	235981	-	The Proton-dependent Oligopeptide Transporter (POT) Family
PAXXG331320	Chr06	scaffold840	147137	153459	+	The Proton-dependent Oligopeptide Transporter (POT) Family
PAXXG345210	unmapped	scaffold1049	85033	88279	+	The Proton-dependent Oligopeptide Transporter (POT) Family
PAXXG358110	unmapped	scaffold1409	45466	45780	+	The Proton-dependent Oligopeptide Transporter (POT) Family
PAXXG358120	unmapped	scaffold1409	45861	46321	+	The Proton-dependent Oligopeptide Transporter (POT) Family
PAXXG001390	Chr02	scaffold1	2832866	2845213	-	The P-type ATPase (P-ATPase) Superfamily
PAXXG003840	Chr10	scaffold2	72996	78994	+	The P-type ATPase (P-ATPase) Superfamily
PAXXG009780	Chr08	scaffold3	1885509	1939414	-	The P-type ATPase (P-ATPase) Superfamily
PAXXG014880	Chr15	scaffold4	4066095	4074232	+	The P-type ATPase (P-ATPase) Superfamily
PAXXG017400	Chr18	scaffold5	1031451	1034622	-	The P-type ATPase (P-ATPase) Superfamily
PAXXG018390	Chr18	scaffold5	2912416	2918784	+	The P-type ATPase (P-ATPase) Superfamily
PAXXG019550	Chr18	scaffold5	5411015	5411436	-	The P-type ATPase (P-ATPase) Superfamily
PAXXG021790	Chr04	scaffold6	5222625	5243907	+	The P-type ATPase (P-ATPase) Superfamily
PAXXG021890	Chr11	scaffold7	375823	393597	-	The P-type ATPase (P-ATPase) Superfamily
PAXXG022480	Chr11	scaffold7	1590191	1619368	-	The P-type ATPase (P-ATPase) Superfamily
PAXXG030730	Chr10	scaffold11	358406	381743	-	The P-type ATPase (P-ATPase) Superfamily
PAXXG031060	Chr10	scaffold11	1249481	1257720	-	The P-type ATPase (P-ATPase) Superfamily
PAXXG057020	Chr01	scaffold23	3476230	3553826	-	The P-type ATPase (P-ATPase) Superfamily
PAXXG062770	Chr08	scaffold27	1248493	1266460	+	The P-type ATPase (P-ATPase) Superfamily
PAXXG065840	Chr04	scaffold29	880975	882072	-	The P-type ATPase (P-ATPase) Superfamily
PAXXG069680	Chr03	scaffold31	2136268	2142800	+	The P-type ATPase (P-ATPase) Superfamily
PAXXG072960	unmapped	scaffold34	508917	574969	-	The P-type ATPase (P-ATPase) Superfamily
PAXXG074620	Chr11	scaffold35	580731	592434	+	The P-type ATPase (P-ATPase) Superfamily
PAXXG086590	Chr03	scaffold44	244987	282661	+	The P-type ATPase (P-ATPase) Superfamily
PAXXG106210	Chr06	scaffold62	1316040	1328099	-	The P-type ATPase (P-ATPase) Superfamily
PAXXG114920	Chr09	scaffold71	62295	80753	-	The P-type ATPase (P-ATPase) Superfamily
PAXXG117320	Chr05	scaffold73	1484066	1589124	-	The P-type ATPase (P-ATPase) Superfamily
PAXXG138780	Chr17	scaffold98	945263	967324	-	The P-type ATPase (P-ATPase) Superfamily
PAXXG138940	Chr17	scaffold98	1226609	1248749	-	The P-type ATPase (P-ATPase) Superfamily
PAXXG144880	Chr11	scaffold105	408628	440984	+	The P-type ATPase (P-ATPase) Superfamily
PAXXG147330	Chr01	scaffold108	776939	843811	+	The P-type ATPase (P-ATPase) Superfamily
PAXXG149470	Chr07	scaffold111	1502196	1592998	+	The P-type ATPase (P-ATPase) Superfamily
PAXXG151750	Chr11	scaffold115	1536234	1548404	+	The P-type ATPase (P-ATPase) Superfamily
PAXXG151800	unmapped	scaffold116	182746	203979	+	The P-type ATPase (P-ATPase) Superfamily
PAXXG168630	Chr10	scaffold141	736409	754987	-	The P-type ATPase (P-ATPase) Superfamily
PAXXG209930	Chr02	scaffold224	697810	703459	+	The P-type ATPase (P-ATPase) Superfamily
PAXXG210570	unmapped	scaffold226	856756	928831	+	The P-type ATPase (P-ATPase) Superfamily
PAXXG214660	Chr06	scaffold240	17647	27522	+	The P-type ATPase (P-ATPase) Superfamily
PAXXG262750	unmapped	scaffold387	6712	21834	-	The P-type ATPase (P-ATPase) Superfamily
PAXXG276410	unmapped	scaffold447	96056	110246	-	The P-type ATPase (P-ATPase) Superfamily
PAXXG291550	Chr07	scaffold524	440607	464513	+	The P-type ATPase (P-ATPase) Superfamily
PAXXG320180	unmapped	scaffold727	248730	258067	+	The P-type ATPase (P-ATPase) Superfamily
PAXXG346720	unmapped	scaffold1076	40550	46199	+	The P-type ATPase (P-ATPase) Superfamily
PAXXG351080	Chr19	scaffold1169	96520	113924	+	The P-type ATPase (P-ATPase) Superfamily
PAXXG386440	unmapped	scaffold6475	1824	2702	+	The P-type ATPase (P-ATPase) Superfamily
PAXXG005770	Chr10	scaffold2	4341815	4344881	+	The Putative 4-Toluene Sulfonate Uptake Permease (TSUP) Family
PAXXG051840	Chr07	scaffold20	3280671	3287373	-	The Putative 4-Toluene Sulfonate Uptake Permease (TSUP) Family
PAXXG063560	Chr08	scaffold27	2880597	2891259	-	The Putative 4-Toluene Sulfonate Uptake Permease (TSUP) Family
PAXXG119540	Chr11	scaffold76	611646	616210	-	The Putative 4-Toluene Sulfonate Uptake Permease (TSUP) Family
PAXXG240360	Chr18	scaffold311	785251	793409	-	The Putative 4-Toluene Sulfonate Uptake Permease (TSUP) Family
PAXXG002060	Chr02	scaffold1	4038994	4192463	-	The Resistance-Nodulation-Cell Division (RND) Superfamily
PAXXG003730	Chr02	scaffold1	8142949	8147489	-	The Resistance-Nodulation-Cell Division (RND) Superfamily
PAXXG023070	Chr11	scaffold7	3099732	3113405	-	The Resistance-Nodulation-Cell Division (RND) Superfamily
PAXXG001850	Chr02	scaffold1	3698347	3706623	+	The Small Conductance Mechanosensitive Ion Channel (MscS) Family
PAXXG028240	Chr14	scaffold10	575489	580778	-	The Small Conductance Mechanosensitive Ion Channel (MscS) Family
PAXXG050650	Chr07	scaffold20	1452353	1518096	-	The Small Conductance Mechanosensitive Ion Channel (MscS) Family
PAXXG057650	Chr13	scaffold24	1484740	1491687	+	The Small Conductance Mechanosensitive Ion Channel (MscS) Family
PAXXG202490	Chr09	scaffold206	5508	83124	+	The Small Conductance Mechanosensitive Ion Channel (MscS) Family
PAXXG241120	Chr09	scaffold314	4518	27269	-	The Small Conductance Mechanosensitive Ion Channel (MscS) Family
PAXXG291490	Chr07	scaffold524	362450	388082	-	The Small Conductance Mechanosensitive Ion Channel (MscS) Family
PAXXG332950	unmapped	scaffold859	132386	140570	-	The Small Conductance Mechanosensitive Ion Channel (MscS) Family
PAXXG094180	Chr03	scaffold50	1960935	1974027	+	The Solute:Sodium Symporter (SSS) Family
PAXXG004920	Chr10	scaffold2	2736175	2743901	-	The Sulfate Permease (SulP) Family
PAXXG011360	Chr08	scaffold3	4930755	4953648	+	The Sulfate Permease (SulP) Family
PAXXG011380	Chr08	scaffold3	4957362	4963755	+	The Sulfate Permease (SulP) Family
PAXXG016210	Chr15	scaffold4	6234688	6240143	-	The Sulfate Permease (SulP) Family
PAXXG045240	Chr12*	scaffold17	3456870	3469001	+	The Sulfate Permease (SulP) Family
PAXXG047990	Chr01	scaffold251	83481	87274	+	The Sulfate Permease (SulP) Family
PAXXG061360	Chr15	scaffold26	2258249	2260143	+	The Sulfate Permease (SulP) Family
PAXXG070370	Chr01	scaffold32	265510	306322	-	The Sulfate Permease (SulP) Family
PAXXG139690	Chr17	scaffold98	2382859	2401426	-	The Sulfate Permease (SulP) Family
PAXXG149570	unmapped	scaffold112	285815	293897	-	The Sulfate Permease (SulP) Family
PAXXG176560	Chr11	scaffold155	708214	713466	-	The Sulfate Permease (SulP) Family
PAXXG176610	Chr11	scaffold155	809884	812337	+	The Sulfate Permease (SulP) Family
PAXXG230820	Chr11	scaffold282	690293	728620	+	The Sulfate Permease (SulP) Family
PAXXG238250	Chr15	scaffold305	597610	639419	-	The Sulfate Permease (SulP) Family
PAXXG387860	unmapped	scaffold7548	354	1000	+	The Sulfate Permease (SulP) Family
PAXXG388180	unmapped	scaffold7839	354	994	+	The Sulfate Permease (SulP) Family
PAXXG139580	Chr17	scaffold98	2275112	2287083	+	The Tellurium Ion Resistance (TerC) Family
PAXXG054290	Chr15	scaffold22	873354	879753	+	The Twin Arginine Targeting (Tat) Family
PAXXG100990	Chr09	scaffold57	1992825	2027832	-	The Twin Arginine Targeting (Tat) Family
PAXXG335730	unmapped	scaffold893	67938	90667	+	The Twin Arginine Targeting (Tat) Family
PAXXG006580	Chr10	scaffold2	6309186	6337526	+	The Type II (General) Secretory Pathway (IISP) Family
PAXXG007170	Chr10	scaffold2	7539409	7539929	+	The Type II (General) Secretory Pathway (IISP) Family
PAXXG008300	Chr10	scaffold2	9745936	9764071	+	The Type II (General) Secretory Pathway (IISP) Family

PAXXG020380	Chr04	scaffold6	2035103	2038415	+	The Type II (General) Secretory Pathway (IISP) Family
PAXXG054890	Chr15	scaffold22	2253446	2284198	-	The Type II (General) Secretory Pathway (IISP) Family
PAXXG063710	Chr08	scaffold27	3118683	3153292	-	The Type II (General) Secretory Pathway (IISP) Family
PAXXG065690	Chr04	scaffold29	616832	623334	+	The Type II (General) Secretory Pathway (IISP) Family
PAXXG069260	Chr03	scaffold31	1386867	1387726	+	The Type II (General) Secretory Pathway (IISP) Family
PAXXG080190	Chr06	scaffold38	1628012	1636408	+	The Type II (General) Secretory Pathway (IISP) Family
PAXXG082000	Chr04	scaffold39	2012765	2013593	+	The Type II (General) Secretory Pathway (IISP) Family
PAXXG086880	Chr03	scaffold44	775546	799380	-	The Type II (General) Secretory Pathway (IISP) Family
PAXXG097750	Chr04	scaffold54	1853215	1905362	+	The Type II (General) Secretory Pathway (IISP) Family
PAXXG116920	Chr05	scaffold73	917624	923217	+	The Type II (General) Secretory Pathway (IISP) Family
PAXXG143040	unmapped	scaffold102	1208281	1282049	+	The Type II (General) Secretory Pathway (IISP) Family
PAXXG164510	Chr10	scaffold134	24480	68170	+	The Type II (General) Secretory Pathway (IISP) Family
PAXXG208610	Chr01	scaffold221	292654	292899	-	The Type II (General) Secretory Pathway (IISP) Family
PAXXG228950	unmapped	scaffold276	293128	312931	-	The Type II (General) Secretory Pathway (IISP) Family
PAXXG260100	Chr10	scaffold375	362149	376218	+	The Type II (General) Secretory Pathway (IISP) Family
PAXXG263220	Chr04	scaffold388	457246	481814	+	The Type II (General) Secretory Pathway (IISP) Family
PAXXG279240	Chr07	scaffold459	441782	459080	+	The Type II (General) Secretory Pathway (IISP) Family
PAXXG315630	unmapped	scaffold689	329799	338596	-	The Type II (General) Secretory Pathway (IISP) Family
PAXXG330550	Chr06	scaffold829	151114	171282	-	The Type II (General) Secretory Pathway (IISP) Family
PAXXG336450	unmapped	scaffold904	126313	152470	+	The Type II (General) Secretory Pathway (IISP) Family
PAXXG113270	Chr19	scaffold69	1589548	1602434	+	The Vacuolar Iron Transporter (VIT) Family
PAXXG125830	Chr16	scaffold83	425174	472446	-	The Vacuolar Iron Transporter (VIT) Family
PAXXG271610	unmapped	scaffold425	512893	513501	-	The Vacuolar Iron Transporter (VIT) Family
PAXXG271640	unmapped	scaffold425	548456	548890	-	The Vacuolar Iron Transporter (VIT) Family
PAXXG325020	Chr13	scaffold772	102922	103717	-	The Vacuolar Iron Transporter (VIT) Family
PAXXG337710	Chr15	scaffold921	201020	201805	-	The Vacuolar Iron Transporter (VIT) Family
PAXXG357680	unmapped	scaffold1394	66386	69411	-	The Vacuolar Iron Transporter (VIT) Family
PAXXG004060	Chr10	scaffold2	624013	631933	-	The Voltage-gated Ion Channel (VIC) Superfamily
PAXXG027310	Chr13	scaffold9	3624986	3638845	+	The Voltage-gated Ion Channel (VIC) Superfamily
PAXXG037460	Chr15	scaffold14	108123	115724	-	The Voltage-gated Ion Channel (VIC) Superfamily
PAXXG038300	Chr15	scaffold14	1702915	1731275	-	The Voltage-gated Ion Channel (VIC) Superfamily
PAXXG044290	Chr12*	scaffold17	1529646	1551902	-	The Voltage-gated Ion Channel (VIC) Superfamily
PAXXG046520	Chr17	scaffold18	1721714	1732301	-	The Voltage-gated Ion Channel (VIC) Superfamily
PAXXG051100	Chr07	scaffold20	2184017	2205197	+	The Voltage-gated Ion Channel (VIC) Superfamily
PAXXG054900	Chr15	scaffold22	2291033	2303652	+	The Voltage-gated Ion Channel (VIC) Superfamily
PAXXG057260	Chr13	scaffold24	589375	606656	-	The Voltage-gated Ion Channel (VIC) Superfamily
PAXXG057400	Chr13	scaffold24	956924	965070	-	The Voltage-gated Ion Channel (VIC) Superfamily
PAXXG069240	Chr03	scaffold31	1287495	1371218	-	The Voltage-gated Ion Channel (VIC) Superfamily
PAXXG126870	unmapped	scaffold84	967662	1019900	-	The Voltage-gated Ion Channel (VIC) Superfamily
PAXXG138420	Chr17	scaffold98	242590	255266	+	The Voltage-gated Ion Channel (VIC) Superfamily
PAXXG143190	unmapped	scaffold102	1621457	1627804	-	The Voltage-gated Ion Channel (VIC) Superfamily
PAXXG159320	Chr13	scaffold126	326467	334916	-	The Voltage-gated Ion Channel (VIC) Superfamily
PAXXG165300	Chr06	scaffold135	489525	535960	+	The Voltage-gated Ion Channel (VIC) Superfamily
PAXXG165530	Chr06	scaffold135	1418982	1427544	+	The Voltage-gated Ion Channel (VIC) Superfamily
PAXXG232850	unmapped	scaffold288	836046	836504	-	The Voltage-gated Ion Channel (VIC) Superfamily
PAXXG252370	unmapped	scaffold348	428629	480699	+	The Voltage-gated Ion Channel (VIC) Superfamily
PAXXG261370	Chr12	scaffold381	331886	447436	+	The Voltage-gated Ion Channel (VIC) Superfamily
PAXXG281320	Chr02	scaffold469	482386	486916	-	The Voltage-gated Ion Channel (VIC) Superfamily
PAXXG284400	unmapped	scaffold485	425270	438316	-	The Voltage-gated Ion Channel (VIC) Superfamily
PAXXG287730	unmapped	scaffold505	293309	321002	-	The Voltage-gated Ion Channel (VIC) Superfamily
PAXXG297790	Chr06	scaffold564	354215	360796	+	The Voltage-gated Ion Channel (VIC) Superfamily
PAXXG315450	unmapped	scaffold688	137506	235769	-	The Voltage-gated Ion Channel (VIC) Superfamily
PAXXG321830	Chr10	scaffold742	76120	116121	+	The Voltage-gated Ion Channel (VIC) Superfamily
PAXXG378220	unmapped	scaffold3484	1074	1866	+	The Voltage-gated Ion Channel (VIC) Superfamily
PAXXG379930	unmapped	scaffold4019	14202	14620	+	The Voltage-gated Ion Channel (VIC) Superfamily
PAXXG009810	Chr08	scaffold3	1943363	1949553	+	The YggT or Fanciful K+ Uptake-B (FkuB; YggT) Family
PAXXG132230	Chr13	scaffold90	21722	42082	+	The YggT or Fanciful K+ Uptake-B (FkuB; YggT) Family
PAXXG251810	Chr11	scaffold347	160810	169408	-	The YggT or Fanciful K+ Uptake-B (FkuB; YggT) Family
PAXXG003850	Chr10	scaffold2	86281	90028	+	The Zinc (Zn2+)-Iron (Fe2+) Permease (ZIP) Family
PAXXG014830	Chr15	scaffold4	4029882	4033769	+	The Zinc (Zn2+)-Iron (Fe2+) Permease (ZIP) Family
PAXXG030910	Chr10	scaffold11	856925	874102	-	The Zinc (Zn2+)-Iron (Fe2+) Permease (ZIP) Family
PAXXG051520	Chr07	scaffold20	2727831	2734610	+	The Zinc (Zn2+)-Iron (Fe2+) Permease (ZIP) Family
PAXXG173540	Chr08	scaffold149	981314	983997	-	The Zinc (Zn2+)-Iron (Fe2+) Permease (ZIP) Family
PAXXG200710	Chr11	scaffold202	747300	750170	-	The Zinc (Zn2+)-Iron (Fe2+) Permease (ZIP) Family
PAXXG264540	unmapped	scaffold393	337295	381374	+	The Zinc (Zn2+)-Iron (Fe2+) Permease (ZIP) Family
PAXXG321110	unmapped	scaffold735	7718	19550	-	The Zinc (Zn2+)-Iron (Fe2+) Permease (ZIP) Family
PAXXG328090	unmapped	scaffold802	27991	88175	+	The Zinc (Zn2+)-Iron (Fe2+) Permease (ZIP) Family
PAXXG013270	Chr15	scaffold4	1154002	1183115	+	Transport protein particle (TRAPP) component
PAXXG192040	Chr02	scaffold186	89424	105111	-	Transport protein particle (TRAPP) component
PAXXG197440	Chr01	scaffold195	992535	995325	-	Vesicle transport v-SNARE family protein

Table 20 Annotation of color, scent and flowering genes

**Flavonoid biosynthesis pathway**							
Gene ID	Scaffold ID	Start	End	strand	Chromosome	Description	Arabidopsis ortholog
PAXXG114440	scaffold70	1342107	1345969	-	Chr09	phenylalanine ammonia-lyase	AT2G37040,AT3G10340,AT3G53260,AT5G04230
PAXXG094080	scaffold50	1784213	1787520	+	Chr03	phenylalanine ammonia-lyase	AT2G37040,AT3G10340,AT3G53260,AT5G04230
PAXXG262090	scaffold384	319239	320798	+	Chr13	phenylalanine ammonia-lyase	N/A
PAXXG031230	scaffold11	1587866	1592658	+	Chr10	cinnamate-4-hydroxylase	AT2G30490
PAXXG108080	scaffold64	1184022	1188622	+	Chr03	cinnamate-4-hydroxylase	AT2G30490
PAXXG137490	scaffold96	1650088	1662712	-	Chr03	4-coumarate:CoA ligase	AT1G20480,AT1G20490,AT1G20500,AT1G20510,AT5G38120
PAXXG137510	scaffold96	1674458	1679340	+	Chr03	4-coumarate:CoA ligase	AT1G20480,AT1G20490,AT1G20500,AT1G20510,AT5G38120
PAXXG001940	scaffold1	3811590	3814799	+	Chr02	4-coumarate:CoA ligase	AT1G20480,AT1G20490,AT1G20500,AT1G20510,AT5G38120
PAXXG137520	scaffold96	1681614	1703595	-	Chr03	4-coumarate:CoA ligase	AT1G20480,AT1G20490,AT1G20500,AT1G20510,AT5G38120
PAXXG000580	scaffold1	975785	982759	+	Chr02	4-coumarate:CoA ligase	AT1G51680,AT1G65060,AT3G21230,AT3G21240
PAXXG020250	scaffold6	1759271	1762017	-	Chr04	4-coumarate:CoA ligase	AT1G51680,AT1G65060,AT3G21230,AT3G21240
PAXXG124540	scaffold82	57686	60426	-	Chr04	4-coumarate:CoA ligase	AT3G48990
PAXXG156720	scaffold122	898233	906048	-	Chr09	4-coumarate:CoA ligase	AT1G51680,AT1G65060,AT3G21230,AT3G21240
PAXXG333030	scaffold860	149851	155124	-	Chr15	4-coumarate:CoA ligase	AT1G51680,AT1G65060,AT3G21230,AT3G21240
PAXXG333050	scaffold860	183722	196048	-	Chr15	4-coumarate:CoA ligase	AT1G51680,AT1G65060,AT3G21230,AT3G21240
PAXXG122400	scaffold79	1114522	1116097	-	Chr07*	chalcone sybthase	AT5G13930
PAXXG174720	scaffold151	914562	916265	+	Chr19	chalcone sybthase	AT5G13930
PAXXG122380	scaffold79	1105589	1107111	-	Chr07*	chalcone sybthase	AT5G13930
PAXXG122420	scaffold79	1129306	1130581	-	Chr07*	chalcone sybthase	AT5G13930
PAXXG173690	scaffold150	153984	155367	+	Chr09	chalcone sybthase	N/A
PAXXG290740	scaffold521	45701	65318	-	Chr11	chalcone isomerase	AT5G05270
PAXXG101960	scaffold58	1027886	1029422	+	Chr16	chalcone isomerase	AT3G55120
PAXXG153170	scaffold118	615184	617327	+	Chr03	flavanone 3-hydroxylase	AT3G51240
PAXXG015280	scaffold4	4840210	4843484	+	Chr15	flavonol synthase	AT5G08640
PAXXG079820	scaffold38	962389	964596	+	Chr06	flavonoid 3'-hydroxylase	AT5G07990
PAXXG172030	scaffold147	590799	593700	+	Chr07	flavonoid 3'-hydroxylase	AT5G07990
PAXXG116590	scaffold73	294487	295430	+	Chr05	flavonoid 3'-hydroxylase	N/A
PAXXG116560	scaffold73	271866	273796	+	Chr05	flavonoid 3'-hydroxylase	N/A
PAXXG116530	scaffold73	254498	256419	+	Chr05	flavonoid 3'-hydroxylase	N/A
PAXXG261960	scaffold383	632235	635643	+	Chr16	flavonoid 3'5'-hydroxylase	AT4G12300,AT4G12310,AT4G12320
PAXXG083130	scaffold40	1783521	1788183	+	Chr03	flavonoid 3'5'-hydroxylase	AT4G12300,AT4G12310,AT4G12320
PAXXG083150	scaffold40	1796315	1799725	+	Chr03	flavonoid 3'5'-hydroxylase	AT4G12300,AT4G12310,AT4G12320
PAXXG083160	scaffold40	1832528	1837124	+	Chr03	flavonoid 3'5'-hydroxylase	AT4G12300,AT4G12310,AT4G12320
PAXXG087010	scaffold44	962392	964512	-	Chr03	flavonoid 3'5'-hydroxylase	N/A
PAXXG070550	scaffold32	1050111	1054262	-	Chr01	anthocyanin synthase/leucoanthocyanidin dioxygenase	AT4G22880
PAXXG222000	scaffold257	855325	857936	+	Chr16	anthocyanin synthase/leucoanthocyanidin dioxygenase	N/A
PAXXG195470	scaffold192	70186	76496	-	Chr07	dihydroflavonol 4-reductase	AT5G42800
PAXXG106640	scaffold62	2061770	2066731	+	Chr06	anthocyanidin reductase	N/A
PAXXG068210	scaffold30	2256123	2257580	+	Chr12	UDP-glucosyltransferase	AT1G50580,AT1G64910,AT1G64920,AT2G22930,AT3G29630,AT4G09500,AT4G27560,AT4G27570,AT5G53990,AT5G54010,AT5G54060
PAXXG240040	scaffold311	297866	299321	-	Chr18	UDP-glucosyltransferase	AT1G07240,AT1G07250,AT1G07260,AT2G29710,AT2G29730,AT2G29740,AT2G29750,AT3G21750,AT3G21760,AT3G21780,AT3G21790,AT3G21800,AT4G15260,AT4G15280
PAXXG223560	scaffold261	649664	665300	+	Chr15	UDP-glucosyltransferase	AT1G30530,AT5G17030,AT5G17040,AT5G17050
PAXXG043240	scaffold16	2824576	2826581	-	Chr19	malonyl-CoA:anthocyanidin 5-O-glucoside-6''-O-malonyltransferase	AT3G29590
PAXXG095230	scaffold51	2239038	2241294	+	Chr09	flavonol 3'-O-methyltransferase	AT5G54160
PAXXG012280	scaffold3	6578262	6595729	+	Chr08	anthocyanin related red Myb	AT5G35550
PAXXG057720	scaffold24	1546582	1548186	-	Chr13	anthocyanin related red Myb	N/A
PAXXG057850	scaffold24	1817647	1819144	-	Chr13	Transducin/WD40 repeat-like superfamily protein	AT5G24520
PAXXG053580	scaffold21	3276330	3315307	-	Chr18	bHLH transcription factor	AT4G09820
PAXXG228220	scaffold274	208012	214211	+	unmapped	2-oxoglutarate (2OG) and Fe(II)-dependent oxygenase superfamily protein	AT4G16330
PAXXG246350	scaffold328	669202	674163	-	Chr10	2-oxoglutarate (2OG) and Fe(II)-dependent oxygenase superfamily protein	AT3G11180,AT5G05600
PAXXG046560	scaffold18	1757389	1762190	+	Chr17	2-oxoglutarate (2OG) and Fe(II)-dependent oxygenase superfamily protein	AT3G11180,AT5G05600
PAXXG084730	scaffold42	314315	316553	-	Chr03	2-oxoglutarate (2OG) and Fe(II)-dependent oxygenase superfamily protein	AT3G11180,AT5G05600
PAXXG325320	scaffold774	200696	204274	+	unmapped	2-oxoglutarate (2OG) and Fe(II)-dependent oxygenase superfamily protein	AT3G11180,AT5G05600
PAXXG033590	scaffold12	1739581	1760389	-	Chr04	2-oxoglutarate (2OG) and Fe(II)-dependent oxygenase superfamily protein	AT1G49390,AT5G20400,AT5G20550,AT5G54000
PAXXG033610	scaffold12	1765683	1791048	-	Chr04	2-oxoglutarate (2OG) and Fe(II)-dependent oxygenase superfamily protein	AT1G49390,AT5G20400,AT5G20550,AT5G54000

Carotenoid biosynthesis pathway

Gene ID	Scaffold ID	Start	End	strand	Chromosome	Description	Arabidopsis ortholog
PAXXG022660	scaffold7	2161241	2165570	+	Chr11	1-deoxy-D-xylulose 5-phosphate synthase	AT3G21500,AT4G15560,AT5G11380
PAXXG141100	scaffold100	152519	167875	-	Chr02	1-deoxy-D-xylulose 5-phosphate synthase	AT3G21500,AT4G15560,AT5G11380
PAXXG376110	scaffold3090	67	10354	+	unmapped	1-deoxy-D-xylulose 5-phosphate synthase	AT3G21500,AT4G15560,AT5G11380
PAXXG022640	scaffold7	2063708	2088057	+	Chr11	1-deoxy-D-xylulose 5-phosphate synthase	AT3G21500,AT4G15560,AT5G11380
PAXXG214790	scaffold240	185932	193529	+	Chr06	1-deoxy-D-xylulose 5-phosphate reductoisomerase	AT5G62790
PAXXG097790	scaffold54	1966672	2020752	+	Chr04	2-C-methyl-D-erythritol 4-phosphatecytidylyltransferase	AT2G02500
PAXXG031150	scaffold11	1418653	1432280	-	Chr10	4-(cytidine 5_-diphospho)-2-C-methyl-D-erythritol kinase	AT2G26930
PAXXG113210	scaffold69	1514294	1521932	+	Chr19	2-C-methyl-D-erythritol 2,4-cyclodiphosphate synthase	AT1G63970
PAXXG059480	scaffold25	1498069	1550654	-	Chr04	4-hydroxy-3-methylbut-2-en-1-yl diphosphate synthase	AT5G60600
PAXXG117380	scaffold73	1708459	1717863	-	Chr05	1-hydroxy-2-methyl-2-(E)-butenyl 4-diphosphate reductase	AT4G34350
PAXXG040330	scaffold15	1541828	1550744	+	Chr07	isopentenyl-diphosphatedelta-isomerase	AT3G02780,AT5G16440
PAXXG203860	scaffold210	298942	301373	-	Chr17	cis epoxycarotenoid dioxygenase	AT1G30100,AT1G78390,AT3G14440, AT3G24220,AT4G18350
PAXXG308020	scaffold631	41942	44220	-	Chr11	cis epoxycarotenoid dioxygenase	AT1G30100,AT1G78390,AT3G14440, AT3G24220,AT4G18350
PAXXG050460	scaffold20	1163611	1166006	-	Chr07	geranylgeranyl pyrophosphate synthase	AT1G49530,AT2G18620,AT2G18640, AT2G23800,AT3G20160,AT4G36810
PAXXG223000	scaffold260	217615	219080	+	Chr06	geranylgeranyl pyrophosphate synthase	AT1G49530,AT2G18620,AT2G18640, AT2G23800,AT3G20160,AT4G36810
PAXXG265290	scaffold396	134428	152193	-	unmapped	phytoene synthase	AT5G17230
PAXXG171220	scaffold145	1080495	1084310	-	Chr18	phytoene synthase	AT5G17230
PAXXG009450	scaffold3	1404149	1417900	-	Chr08	phytoene desaturase	AT4G14210
PAXXG270380	scaffold420	306491	316298	-	Chr18	9,9'-di-cis-zeta-carotenedesaturase	AT3G04870
PAXXG055620	scaffold23	395432	455635	-	Chr01	carotenoid isomerase	AT1G06820
PAXXG026610	scaffold9	1835487	1837241	-	Chr13	Lycopene beta cyclase	AT3G10230
PAXXG237630	scaffold303	523651	576219	-	unmapped	Lycopene epsilon cyclase	AT5G57030
PAXXG076880	scaffold36	2279980	2283594	+	Chr02	beta-carotene hydroxylase	AT4G25700,AT5G52570
PAXXG082590	scaffold40	782130	785418	-	Chr03	beta-carotene hydroxylase	N/A
PAXXG015300	scaffold4	4865325	4878828	+	Chr15	zeaxanthin epoxidase	AT5G67030

Scent-related pathway

Gene ID	Scaffold ID	Start	End	strand	Chromosome	Description	Arabidopsis ortholog
PAXXG023340	scaffold7	3890470	3901886	+	Chr11	linoleate 13S-lipoxygenase	AT1G17420,AT1G67560,AT1G72520, AT3G45140
PAXXG045670	scaffold17	4105465	4106831	-	Chr12*	linoleate 13S-lipoxygenase	AT1G17420,AT1G67560,AT1G72520, AT3G45140
PAXXG056000	scaffold23	1256375	1270963	-	Chr01	linoleate 13S-lipoxygenase	AT1G17420,AT1G67560,AT1G72520, AT3G45140
PAXXG088720	scaffold45	2429558	2434463	-	Chr06	linoleate 13S-lipoxygenase	AT1G17420,AT1G67560,AT1G72520, AT3G45140
PAXXG098190	scaffold55	586526	591942	-	Chr01	linoleate 13S-lipoxygenase	AT1G17420,AT1G67560,AT1G72520, AT3G45140
PAXXG180070	scaffold162	987231	994038	+	Chr07	linoleate 13S-lipoxygenase	AT1G17420,AT1G67560,AT1G72520, AT3G45140
PAXXG354990	scaffold1292	93848	97502	-	unmapped	linoleate 13S-lipoxygenase	AT1G17420,AT1G67560,AT1G72520, AT3G45140
PAXXG094080	scaffold50	1784213	1787520	+	Chr03	phenylalanine ammonia-lyase	AT2G37040,AT3G10340,AT3G53260, AT5G04230
PAXXG114440	scaffold70	1342107	1345969	-	Chr09	phenylalanine ammonia-lyase	AT2G37040,AT3G10340,AT3G53260, AT5G04230
PAXXG000580	scaffold1	975785	982759	+	Chr02	4-coumarate:CoA ligase	AT1G51680,AT1G65060,AT3G21230, AT3G21240
PAXXG020250	scaffold6	1759271	1762017	-	Chr04	4-coumarate:CoA ligase	AT1G51680,AT1G65060,AT3G21230, AT3G21240
PAXXG333030	scaffold860	149851	155124	-	Chr15	4-coumarate:CoA ligase	AT1G51680,AT1G65060,AT3G21230, AT3G21240
PAXXG333050	scaffold860	183722	196048	-	Chr15	4-coumarate:CoA ligase	AT1G51680,AT1G65060,AT3G21230, AT3G21240
PAXXG156720	scaffold122	898233	906048	-	Chr09	4-coumarate:CoA ligase	AT1G51680,AT1G65060,AT3G21230, AT3G21240
PAXXG252480	scaffold349	296258	347972	+	unmapped	enoyl-CoA hydratase/3-hydroxyacyl-CoA dehydrogenase	AT4G29010,AT3G06860
PAXXG290990	scaffold522	174233	221366	-	Chr04	enoyl-CoA hydratase/3-hydroxyacyl-CoA dehydrogenase	AT4G29010,AT3G06860
PAXXG228340	scaffold274	667982	700427	-	unmapped	enoyl-CoA hydratase/3-hydroxyacyl-CoA dehydrogenase	AT4G29010,AT3G06860
PAXXG228330	scaffold274	628549	655747	-	unmapped	enoyl-CoA hydratase/3-hydroxyacyl-CoA dehydrogenase	AT4G29010,AT3G06860
PAXXG023720	scaffold7	4765113	4768053	+	Chr11	S-adenosyl-L-methionine:benzoic acid carboxymethyltransferase	AT2G14060,AT3G11480,AT3G21950, AT5G04370,AT5G04380,AT5G38020, AT5G66430
PAXXG022640	scaffold7	2063708	2088057	+	Chr11	1-deoxy-D-xylulose 5-phosphate synthase	AT3G21500,AT4G15560,AT5G11380
PAXXG022660	scaffold7	2161241	2165570	+	Chr11	1-deoxy-D-xylulose 5-phosphate synthase	AT3G21500,AT4G15560,AT5G11380
PAXXG141100	scaffold100	152519	167875	-	Chr02	1-deoxy-D-xylulose-5-phosphate synthase	AT3G21500,AT4G15560,AT5G11380
PAXXG376110	scaffold3090	67	10354	+	unmapped	1-deoxy-D-xylulose-5-phosphate synthase	AT3G21500,AT4G15560,AT5G11380
PAXXG086710	scaffold44	466630	468298	+	Chr03	trans-2-hexenal reductase	AT2G24190,AT3G61220
PAXXG049850	scaffold19	3033571	3047685	+	Chr07	terpinolene synthase	AT2G24210,AT3G25810,AT3G25820, AT3G25830,AT4G16740
PAXXG149140	scaffold111	774546	783052	-	Chr07	terpinolene synthase	AT2G24210,AT3G25810,AT3G25820, AT3G25830,AT4G16740
PAXXG276730	scaffold448	36922	52860	+	Chr07#	terpinolene synthase	AT2G24210,AT3G25810,AT3G25820, AT3G25830,AT4G16740
PAXXG276750	scaffold448	78368	82143	+	Chr07#	terpinolene synthase	AT2G24210,AT3G25810,AT3G25820, AT3G25830,AT4G16740
PAXXG147080	scaffold108	284270	287136	-	Chr01	3-deoxy-7-phosphoheptulonate synthase	AT1G22410,AT4G33510,AT4G39980
PAXXG233330	scaffold290	113639	117021	+	Chr15	3-deoxy-7-phosphoheptulonate synthase	AT1G22410,AT4G33510,AT4G39980
PAXXG365830	scaffold1825	30076	53158	+	unmapped	3-deoxy-7-phosphoheptulonate synthase	AT1G22410,AT4G33510,AT4G39980

PAXXG077670	scaffold37	850940	852506	+	Chr10	cinnamyl-alcohol dehydrogenase	AT2G21730,AT2G21890,AT4G37970,AT4G37980,AT4G37990,AT4G39330
PAXXG078010	scaffold37	1319819	1321430	-	Chr10	cinnamyl-alcohol dehydrogenase	AT2G21730,AT2G21890,AT4G37970,AT4G37980,AT4G37990,AT4G39330
PAXXG078020	scaffold37	1331878	1333577	-	Chr10	cinnamyl-alcohol dehydrogenase	AT2G21730,AT2G21890,AT4G37970,AT4G37980,AT4G37990,AT4G39330
PAXXG290630	scaffold520	371476	380438	-	unmapped	cinnamyl-alcohol dehydrogenase	AT2G21730,AT2G21890,AT4G37970,AT4G37980,AT4G37990,AT4G39330
PAXXG328480	scaffold806	23199	28226	+	unmapped	cinnamyl-alcohol dehydrogenase	AT2G21730,AT2G21890,AT4G37970,AT4G37980,AT4G37990,AT4G39330
PAXXG003730	scaffold1	8142949	8147489	-	Chr02	3-hydroxy-3-methylglutaryl coenzyme A reductase	AT1G76490,AT2G17370
PAXXG023070	scaffold7	3099732	3113405	-	Chr11	3-hydroxy-3-methylglutaryl coenzyme A reductase	AT1G76490,AT2G17370
PAXXG100090	scaffold57	336841	358240	+	Chr09	thiolases	AT5G48880,AT2G33150,AT1G04710
PAXXG271000	scaffold423	119015	157957	-	unmapped	peroxisomal 3-keto-acyl-CoA thiolase	AT5G48880,AT2G33150,AT1G04710
PAXXG088420	scaffold45	1897344	1900783	+	Chr06	trans-2-hexenal reductase	AT3G04000
PAXXG152580	scaffold117	693169	694160	+	Chr10	trans-2-hexenal reductase	AT3G04000
PAXXG152610	scaffold117	733161	733905	+	Chr10	trans-2-hexenal reductase	AT3G04000
PAXXG181720	scaffold166	7837	26376	-	Chr06	acetoacetyl-CoA thiolase	AT5G47720,AT5G48230
PAXXG228900	scaffold276	172002	186593	+	unmapped	AMP-dependent synthetase and ligase family protein	AT2G17650
PAXXG042660	scaffold16	1444690	1450847	+	Chr19	3-hydroxy-3-methylglutaryl coenzyme A synthase	AT4G11820
PAXXG040330	scaffold15	1541828	1550744	+	Chr07	isopentenyl-diphosphatedelta-isomerase	AT3G02780,AT5G16440
PAXXG059020	scaffold25	441980	444217	-	Chr04	4-coumarate-CoA ligase	AT1G21530,AT1G21540,AT1G75960,AT1G77240,AT5G16340,AT5G16370
PAXXG110950	scaffold67	1550749	1559062	+	Chr14*	shikimate kinase	AT2G21940,AT4G39540
PAXXG157980	scaffold124	1085274	1168914	-	unmapped	peroxisomal protein	AT4G39850
PAXXG050460	scaffold20	1163611	1166006	-	Chr07	geranylgeranyl pyrophosphate synthase	AT1G49530,AT2G18620,AT2G18640,AT2G23800,AT3G20160,AT4G36810
PAXXG223000	scaffold260	217615	219080	+	Chr06	geranylgeranyl pyrophosphate synthase	AT1G49530,AT2G18620,AT2G18640,AT2G23800,AT3G20160,AT4G36810
PAXXG000840	scaffold1	1391843	1395566	+	Chr02	cinnamoyl-CoA reductase	AT2G23910,AT4G30470
PAXXG156520	scaffold122	325151	329982	-	Chr09	cinnamoyl-CoA reductase, 4-coumarate-CoA ligase	AT2G23910,AT4G30470,AT1G51680,AT1G65060,AT3G21230,AT3G21240
PAXXG201480	scaffold204	201778	202314	-	unmapped	shikimate O-hydroxycinnamoyltransferase	AT3G50270,AT3G50300,AT5G67150,AT5G67160
PAXXG026660	scaffold9	1995718	2063413	-	Chr13	shikimate dehydrogenase	AT3G06350
PAXXG090030	scaffold46	2419075	2427020	+	Chr04	cinnamoyl-CoA reductase	AT2G33590
PAXXG090040	scaffold46	2436423	2451596	+	Chr04	cinnamoyl-CoA reductase	AT2G33590
PAXXG081290	scaffold39	851387	854822	-	Chr04	trans-2-hexenal reductase, oxidoreductase	AT2G37770,AT2G37790
PAXXG212580	scaffold232	871824	877554	+	Chr11	trans-2-hexenal reductase, oxidoreductase	AT2G37770,AT2G37790
PAXXG212590	scaffold232	889635	895373	+	Chr11	trans-2-hexenal reductase, oxidoreductase	AT2G37770,AT2G37790
PAXXG067750	scaffold30	965004	966966	+	Chr12	caffeoyl-CoA O-methyltransferase	AT1G24735,AT1G67980,AT1G67990
PAXXG323060	scaffold754	83467	86019	+	unmapped	caffeoyl-CoA O-methyltransferase	AT1G24735,AT1G67980,AT1G67990
PAXXG305330	scaffold611	435261	445343	+	Chr19	farnesyl pyrophosphate synthase	AT4G17190,AT5G47770
PAXXG152780	scaffold117	1128545	1153536	+	Chr10	geranyl pyrophosphate synthase	AT2G34630
PAXXG156730	scaffold122	911109	942158	-	Chr09	phosphomevalonate kinase	AT1G31910
PAXXG036580	scaffold13	2750978	2770782	-	Chr16	mevalonate diphosphate decarboxylase	AT2G38700,AT3G54250
PAXXG183310	scaffold168	363879	443921	-	unmapped	caffeoyl-CoA O-methyltransferase	AT3G61990,AT3G62000
PAXXG263310	scaffold388	621992	626515	-	Chr04	p-coumaroyl shikimate/quinate 3-hydroxylase3-hydroxylase	AT2G40890
PAXXG078920	scaffold37	2505084	2507117	+	Chr10	shikimate O-hydroxycinnamoyltransferase	AT2G39980,AT5G01210
PAXXG055340	scaffold22	3156675	3167310	+	Chr15	caffeoyl-CoA:quinate O-(hydroxycinnamoyl)transferase	AT5G48930
PAXXG155180	scaffold121	33090	63774	+	Chr10	solanesyl diphosphate synthase,geranylgeranyl diphosphate synthase	AT1G17050,AT1G78510
PAXXG064240	scaffold28	650844	652886	-	Chr01	benzoate-CoA ligase	AT1G68270,AT1G66120,AT1G65890,AT1G65880
PAXXG230910	scaffold283	53254	58144	+	Chr09	3-phosphoshikimate1-carboxyvinyl transferase	AT1G48860,AT2G45300
PAXXG263500	scaffold389	341220	361333	+	unmapped	shikimate O-hydroxycinnamoyltransferase	AT3G23840,AT4G13840
PAXXG137890	scaffold97	700023	705101	-	Chr04	cinnamoyl-CoA reductase	AT1G15950,AT1G80820
PAXXG069140	scaffold31	1043831	1126572	+	Chr03	isopentenyl-diphosphatedelta-isomerase	AT1G79690
PAXXG095230	scaffold51	2239038	2241294	+	Chr09	flavonol 3'-O-methyltransferase	AT5G54160
PAXXG095020	scaffold51	1677668	1693584	+	Chr09	cinnamyl-alcohol dehydrogenase	AT1G72680
PAXXG062610	scaffold27	891360	905168	+	Chr08	chorismate mutase	AT5G10870
PAXXG093440	scaffold50	297074	300188	+	Chr03	coniferyl aldehyde 5-hydroxylase	AT4G36220,AT5G04330
PAXXG008180	scaffold2	9587454	9589588	-	Chr10	arogenate dehydratase	AT1G08250
PAXXG286040	scaffold494	321057	337503	-	unmapped	chorismate synthase	AT1G48850
PAXXG019010	scaffold5	4126406	4129375	+	Chr18	caffeoyl-CoA O-methyltransferase	AT4G34050
PAXXG165710	scaffold136	473700	513992	+	unmapped	farnesyltranstransferase	AT3G59380
PAXXG125680	scaffold83	168614	170246	-	Chr16	shikimate O-hydroxycinnamoyltransferase	AT5G07850,AT5G07870,AT5G42830
PAXXG112560	scaffold69	439336	444447	+	Chr19	3-dehydroquinate synthase	AT5G66120
PAXXG193150	scaffold188	211880	227926	+	Chr13	aspartate aminotransferase	AT2G22250
PAXXG017110	scaffold5	332793	342949	-	Chr18	chorismate mutase	AT3G29200,AT1G69370
PAXXG117380	scaffold73	1708459	1717863	-	Chr05	1-hydroxy-2-methyl-2-(E)-butenyl 4-diphosphate reductase	AT4G34350
PAXXG214790	scaffold240	185932	193529	+	Chr06	1-deoxy-D-xylulose 5-phosphate reductoisomerase	AT5G62790
PAXXG059480	scaffold25	1498069	1550654	-	Chr04	4-hydroxy-3-methylbut-2-en-1-yl diphosphate synthase	AT5G60600
PAXXG113210	scaffold69	1514294	1521932	+	Chr19	2C-methyl-D-erythritol 2,4-cyclodiphosphate synthase	AT1G63970
PAXXG068630	scaffold31	39056	44151	-	Chr03	mevalonate kinase	AT5G27450
PAXXG097790	scaffold54	1966672	2020752	+	Chr04	2-C-methyl-D-erythritol 4-phosphatecytidylyltransferase	AT2G02500
PAXXG031150	scaffold11	1418653	1432280	-	Chr10	4-(cytidine 5_-diphospho)-2-C-methyl-D-erythritol kinase	AT2G26930
PAXXG199270	scaffold200	101125	102828	-	Chr18	(Z)-3-hexen-1-ol acetyltransferase	AT3G03480,AT5G17540
PAXXG234210	scaffold293	244345	247672	+	Chr02	coniferyl alcohol dehydrogenase	AT3G19450
PAXXG183070	scaffold167	976986	980049	+	Chr02	shikimate O-hydroxycinnamoyltransferase	AT5G23940
PAXXG316420	scaffold695	299265	300475	-	unmapped	(2E,6E)-farnesyl diphosphate synthase	AT4G38460

PAXXG334760	scaffold879	21726	23053	-	unmapped	trans-2-hexenal reductase, oxidoreductase	AT1G54870,AT3G05260

Circadian rhythm pathway

Gene ID	Scaffold ID	Start	End	strand	Chromosome	Description	Arabidopsis ortholog
PAXXG106330	scaffold62	1568932	1582223	-	Chr06	encodes a nuclear protein that is expressed rhythmically and interacts with phytochrome B	AT2G25930
PAXXG049030	scaffold19	1165173	1172905	-	Chr07	EARLY FLOWERING 4 like	AT2G06255,AT1G72630,AT1G17455
PAXXG044520	scaffold17	1898805	1900696	-	Chr12*	EARLY FLOWERING 4 like	AT2G06255,AT1G72630,AT1G17455
PAXXG221150	scaffold255	781659	784396	-	Chr01	a transcription factor interacting with photoreceptors phyA and phyB	AT1G09530
PAXXG024230	scaffold8	1095013	1116762	-	Chr11	Transducin/WD40 repeat-like superfamily protein	AT2G32950
PAXXG039150	scaffold14	3477739	3507260	-	Chr15	SPA (suppressor of phyA-105) protein family	AT2G46340,AT4G11110
PAXXG103510	scaffold59	1230206	1250518	-	Chr12	GIGANTEA, promotes flowering under long days in a circadian clock-controlled flowering pathway	AT1G22770
PAXXG024400	scaffold8	1543072	1569414	-	Chr11	Basic-leucine zipper (bZIP) transcription factor family protein	AT5G11260
PAXXG057300	scaffold24	739431	754288	+	Chr13	Basic-leucine zipper (bZIP) transcription factor family protein	AT5G11260
PAXXG386560	scaffold6532	1336	2100	+	unmapped	Basic-leucine zipper (bZIP) transcription factor family protein	AT5G11260
PAXXG007220	scaffold2	7626099	7658464	-	Chr10	a MYB-related putative transcription factor involved in Circadian rhythm pathway	AT1G01060,AT2G46830
PAXXG241260	scaffold314	347703	358528	+	Chr09	a MYB-like transcription factor	AT5G52660
PAXXG046480	scaffold18	1645665	1659903	-	Chr17	a MYB-like transcription factor	AT3G09600,AT5G02840
PAXXG064920	scaffold28	2130170	2131843	-	Chr01	a MYB-like transcription factor	AT5G17300,AT5G37260
PAXXG178400	scaffold159	1181459	1185330	+	Chr19	a MYB-like transcription factor	AT5G17300,AT5G37260
PAXXG096870	scaffold53	2305445	2309074	+	Chr13	a MYB-like transcription factor	AT5G17300,AT5G37260
PAXXG021820	scaffold7	103399	147144	-	Chr11	a chloroplast localized subunit of casien kinase4	AT2G23070
PAXXG171760	scaffold147	59427	78250	+	Chr07	Casein kinase II catalytic subunit	AT5G67380,AT3G50000
PAXXG001030	scaffold1	1809523	1815654	-	Chr02	Casein kinase II beta chain, a CK2 regulatory subunit	AT2G44680,AT3G60250,AT5G47080, AT4G17640
PAXXG135170	scaffold93	2144973	2169977	-	Chr16	Casein kinase II beta chain, a CK2 regulatory subunit	AT2G44680,AT3G60250,AT5G47080, AT4G17640
PAXXG087460	scaffold44	2077380	2097139	-	Chr03	Casein kinase II beta chain, a CK2 regulatory subunit	AT2G44680,AT3G60250,AT5G47080, AT4G17640
PAXXG139200	scaffold98	1587150	1600717	-	Chr17	a Dof-type zinc finger domain-containing protein	AT5G62430
PAXXG108980	scaffold65	1790074	1811658	+	Chr17	FIO, methyltransferase	AT2G21070
PAXXG218840	scaffold250	97680	141214	-	Chr03	2-oxoglutarate (2OG) and Fe(II)-dependent oxygenase superfamily protein	AT3G20810
PAXXG067040	scaffold29	2934037	2935347	+	Chr04	a MYB-like transcription factor	AT5G59570,AT3G46640
PAXXG078130	scaffold37	1436709	1437887	+	Chr10	a MYB-like transcription factor	AT5G59570,AT3G46640
PAXXG007930	scaffold2	9145432	9147063	-	Chr10	LIGHT-REGULATED WD, a clock proteins regulating circadian period length and photoperiodic flowering	AT3G26640,AT1G12910
PAXXG201800	scaffold204	832658	846605	+	unmapped	PROTEIN ARGININE METHYLTRANSFERASE 5, involved in Vernalization pathway	AT4G31120
PAXXG141920	scaffold100	1467447	1513782	-	Chr02	POLY(ADP-RIBOSE) GLYCOHYDROLASE 1, a poly(ADPribose) glycohydrolase (PARG1)	AT2G31870
PAXXG180020	scaffold162	905173	912542	+	Chr07	TIME FOR COFFEE, a nucleus-acting plant-specific clock regulator	AT3G22380
PAXXG154560	scaffold120	111729	152525	+	unmapped	NIGHT LIGHT-INDUCIBLE AND CLOCK-REGULATED 2, agglutinin-like protein	AT3G54500
PAXXG324820	scaffold771	6069	52784	-	Chr16	DE-ETIOLATED 1, a repressor of photomorphogenesis	AT4G10180
PAXXG139630	scaffold98	2311697	2316131	-	Chr17	SENSITIVITY TO RED LIGHT REDUCED 1, is required for normal oscillator function during Circadian rhythm pathway	AT5G59560

Vernalization pathway

Gene ID	Scaffold ID	Start	End	strand	Chromosome	Description	Arabidopsis ortholog
PAXXG330340	scaffold827	221335	226659	+	Chr15	FRI, FRIGIDA	AT4G00650
PAXXG166220	scaffold137	517922	525225	+	Chr05	FRL, FRIGIDA-LIKE	AT5G16320,AT1G31814
PAXXG006370	scaffold2	5931736	6006390	+	Chr10	SNF2 domain-containing protein / helicase domain-containing protein	AT3G12810
PAXXG159550	scaffold126	676905	692135	+	Chr13	Cysteine proteinases superfamily protein	AT4G15880
PAXXG263040	scaffold388	61828	75361	+	Chr04	VIP3, Vernalization pathway independence	AT4G29830
PAXXG074670	scaffold35	643024	671877	-	Chr11	VIP4, Vernalization pathway independence	AT5G61150
PAXXG024500	scaffold8	1963610	2030404	+	Chr11	FCA, Flowering time control protein	AT4G16280
PAXXG338510	scaffold933	41645	95013	+	unmapped	FY, an mRNA processing factor	AT5G13480
PAXXG017310	scaffold5	720738	736318	-	Chr18	RNA-binding KH domain-containing protein	AT3G04610
PAXXG134400	scaffold93	119426	137841	+	Chr16	LUMINIDEPENDENS, a nuclear localized protein with similarity to transcriptional regulators.	AT4G02560
PAXXG294730	scaffold546	195468	211533	-	Chr19	a homolog of human Lysine-Specific Demethylase1	AT3G10390,AT3G13682,AT1G62830
PAXXG230340	scaffold280	364212	367218	-	Chr14	a homolog of human Lysine-Specific Demethylase1	AT3G10390,AT3G13682,AT1G62830
PAXXG013220	scaffold4	1100654	1111608	+	Chr15	a homolog of human Lysine-Specific Demethylase1	AT3G10390,AT3G13682,AT1G62830
PAXXG200990	scaffold203	369374	386533	-	Chr12	FVE, the retinoblastoma-associated protein that regulates flowering time and cold response	AT2G19520
PAXXG346200	scaffold1064	149948	163741	-	unmapped	FPA, a gene that regulates flowering time	AT2G43410
PAXXG100950	scaffold57	1921826	1958819	-	Chr09	VRN1, essential for the complete repression of FLC in vernalized plants	AT3G18990
PAXXG345890	scaffold1060	87113	91135	-	Chr12	LFY, transcriptional regulator that promotes the transition to flowering	AT5G61850

Photoreceptor

Gene ID	Scaffold ID	Start	End	strand	Chromosome	Description	Arabidopsis ortholog
PAXXG061570	scaffold26	2599519	2621897	+	Chr15	PHYTOCHROME A, a light-labile cytoplasmic red/far-red light photoreceptor	AT1G09570
PAXXG067160	scaffold29	3160102	3175281	-	Chr04	PHYTOCHROME B, a red/far-red photoreceptor	AT2G18790
PAXXG309810	scaffold641	230811	239791	-	unmapped	PHYTOCHROME C, a red/far-red photoreceptor	AT5G35840

PAXXG016060	scaffold4	6058370	6066043	+	Chr15	CRYPTOCHROME, a flavin-type blue-light photoreceptor	AT4G08920, AT1G04400
PAXXG195160	scaffold191	772872	779379	+	Chr08	CRYPTOCHROME, a flavin-type blue-light photoreceptor	AT4G08920, AT1G04400
PAXXG200460	scaffold201	900085	922570	-	Chr19	CRYPTOCHROME, a flavin-type blue-light photoreceptor	AT4G08920, AT1G04400
PAXXG069640	scaffold31	2015319	2036918	-	Chr03	adagio-like F-box protein	AT1G68050,AT5G57360,AT2G18915
PAXXG170540	scaffold144	1117806	1136047	+	unmapped	adagio-like F-box protein	AT1G68050,AT5G57360,AT2G18915
PAXXG294370	scaffold544	433218	435247	-	Chr10	adagio-like F-box protein	AT1G68050,AT5G57360,AT2G18915
PAXXG304920	scaffold609	263821	340410	+	Chr18	PHOTOTROPIN, a blue-light photoreceptor	AT3G45780,AT5G58140
PAXXG022950	scaffold7	2842670	2879771	-	Chr11	PHOTOTROPIN, a blue-light photoreceptor	AT3G45780,AT5G58140
PAXXG321800	scaffold741	242096	297354	-	unmapped	UV-B photoreceptor	AT5G63860

PEBP family

Gene ID	Scaffold ID	Start	End	strand	Chromosome	Description	Arabidopsis ortholog
PAXXG186610	scaffold174	108175	110050	+	unmapped	flowering locus T	AT1G65480,AT4G20370
PAXXG122730	scaffold79	1881593	1884299	-	Chr07*	MFT, a member of the FT and TFL1 family of phosphatidylethanolamine-binding proteins.	AT1G18100
PAXXG006730	scaffold2	6592547	6609538	-	Chr10	flowering locus T like	N/A
PAXXG313110	scaffold666	344633	347259	-	unmapped	flowering locus T like	N/A
PAXXG093260	scaffold49	2415412	2416526	-	Chr15	flowering locus T like	N/A
PAXXG182430	scaffold166	1135962	1163827	+	Chr06	flowering locus T like	N/A
PAXXG103970	scaffold59	2149825	2155274	+	Chr12	flowering locus T like	N/A

TOC1-PRR family

Gene ID	Scaffold ID	Start	End	strand	Chromosome	Description	Arabidopsis ortholog
PAXXG046380	scaffold18	1455576	1486090	-	Chr17	pseudo-response regulator	AT5G02810
PAXXG324880	scaffold771	240827	248134	-	Chr16	pseudo-response regulator	AT5G24470
PAXXG063700	scaffold27	3100666	3108800	-	Chr08	pseudo-response regulator	AT5G60100

BBX family

Gene ID	Scaffold ID	Start	End	strand	Chromosome	Description	Arabidopsis ortholog
PAXXG141050	scaffold100	107260	117730	-	Chr02	B-box type zinc finger protein with CCT domain	AT2G47890
PAXXG207970	scaffold219	726779	751806	-	unmapped	B-box type zinc finger protein with CCT domain	AT2G33500,AT1G28050
PAXXG253980	scaffold356	43859	64876	+	Chr18	B-box type zinc finger protein with CCT domain	AT3G07650,AT5G48250
PAXXG275020	scaffold441	104811	127250	+	Chr11	B-box type zinc finger protein with CCT domain	AT3G07650,AT5G48250
PAXXG012810	scaffold4	262708	264589	-	Chr15	B-box type zinc finger protein with CCT domain	AT2G24790,AT5G24930,AT5G57660
PAXXG018120	scaffold5	2373178	2374698	-	Chr18	B-box type zinc finger protein with CCT domain	AT2G24790,AT5G24930,AT5G57660
PAXXG176690	scaffold155	1050159	1051222	-	Chr11	B-box type zinc finger protein with CCT domain	AT5G15850,AT5G15840,AT3G02380
PAXXG010150	scaffold3	2438744	2440701	-	Chr08	B-box type zinc finger protein with CCT domain	AT1G73870
PAXXG358340	scaffold1421	71989	73807	-	Chr07	B-box type zinc finger protein with CCT domain	AT1G68520.1,AT1G25440.1
PAXXG070650	scaffold32	1332247	1334678	-	Chr01	B-BOX domain protein	AT4G39070,AT1G75540
PAXXG114780	scaffold70	1945238	1946544	+	Chr09	B-BOX domain protein	AT4G39070,AT1G75540
PAXXG255490	scaffold360	306941	311972	-	Chr10	B-BOX domain protein	AT1G78600
PAXXG070680	scaffold32	1412397	1452298	-	Chr01	B-BOX domain protein	AT4G38960, AT2G21320
PAXXG217350	scaffold246	271887	275200	+	Chr13	B-BOX domain protein	AT2G31380, AT1G06040
PAXXG268780	scaffold411	431157	442290	-	unmapped	B-BOX domain protein	N/A
PAXXG190480	scaffold182	804855	806042	-	Chr09	B-BOX domain protein	N/A
PAXXG199460	scaffold200	413489	417237	+	Chr18	B-BOX domain protein	N/A
PAXXG163100	scaffold131	237248	254532	+	unmapped	B-BOX domain protein	N/A
PAXXG014790	scaffold4	3985377	3988176	-	Chr15	B-BOX domain protein	N/A

CO-Like family

Gene ID	Scaffold ID	Start	End	strand	Chromosome	Description	Arabidopsis ortholog
PAXXG072100	scaffold33	2046376	2067999	+	Chr02	CCT domain protein	N/A
PAXXG018630	scaffold5	3356947	3358619	+	Chr18	CCT domain protein	N/A
PAXXG055550	scaffold23	178085	180675	+	Chr01	CCT domain protein	N/A
PAXXG055560	scaffold23	189501	191252	+	Chr01	CCT domain protein	N/A
PAXXG061890	scaffold26	3279458	3282193	+	Chr15	CCT domain protein	AT5G57180,AT4G25990
PAXXG094460	scaffold51	272420	274324	-	Chr09	CCT domain protein	N/A
PAXXG127290	scaffold85	269038	270784	+	Chr11	CCT domain protein	AT5G59990
PAXXG133650	scaffold92	404814	405920	+	Chr03	CCT domain protein	AT5G59990
PAXXG200330	scaffold201	605499	606974	+	Chr19	CCT domain protein	AT2G33350,AT1G04500
PAXXG200360	scaffold201	630576	633889	+	Chr19	CCT domain protein	AT2G33350,AT1G04500
PAXXG310080	scaffold643	92525	98198	-	Chr12	CCT domain protein	AT5G53420,AT4G27900
PAXXG354960	scaffold1292	25767	51803	-	unmapped	CCT domain protein	N/A
PAXXG374510	scaffold2825	2024	2618	+	unmapped	CCT domain protein	N/A
PAXXG374520	scaffold2825	4354	6280	+	unmapped	CCT domain protein	N/A
PAXXG374530	scaffold2825	8957	11434	+	unmapped	CCT domain protein	N/A
PAXXG018580	scaffold5	3270482	3281836	-	Chr18	CCT domain protein	N/A
PAXXG088460	scaffold45	1984514	1997202	+	Chr06	CCT domain protein	N/A
PAXXG144870	scaffold105	379447	392855	+	Chr11	CCT domain protein	AT1G51600,AT3G21175

MADS-box family

Gene ID	Scaffold ID	Start	End	strand	Chromosome	Description	Arabidopsis ortholog
PAXXG130000	scaffold87	1798895	1814924	-	Chr02	AGAMOUS-like, PaAGL1	AT2G42830,AT3G58780,AT4G18960
PAXXG271330	scaffold424	91558	143326	+	Chr10	AGAMOUS-like, PaAGL2	AT2G42830,AT3G58780,AT4G18960
PAXXG182380	scaffold166	971555	983826	-	Chr06	AGAMOUS-like, PaAGL3	AT2G42830,AT3G58780,AT4G18960

PAXXG220840	scaffold255	341922	357497	+	Chr01	AGAMOUS-like, PaAGL4	AT4G09960
PAXXG217950	scaffold248	159697	232560	-	unmapped	AGAMOUS-like, PaAGL5	AT4G09960
PAXXG220800	scaffold255	261997	264707	-	Chr01	AGAMOUS-like, PaAGL6	N/A
PAXXG198660	scaffold198	963849	1007689	+	unmapped	AGL6-like, PaAGL7	N/A
PAXXG301780	scaffold588	340509	378757	-	Chr19	AGL6-like, PaAGL8	AT2G45650
PAXXG228490	scaffold275	592	27933	+	Chr16	AGL6-like, PaAGL9	N/A
PAXXG198670	scaffold198	1014758	1043194	+	unmapped	AGL6-like, PaAGL10	N/A
PAXXG348560	scaffold1116	96180	123773	+	unmapped	AGL6-like, PaAGL11	N/A
PAXXG350320	scaffold1153	12263	95246	+	unmapped	ANR-like, PaANR_1	AT3G57230,AT4G37940,AT2G22630
PAXXG049240	scaffold19	1560628	1580957	-	Chr07	ANR-like, PaANR_2	N/A
PAXXG080090	scaffold38	1423895	1480121	+	Chr06	AP1-like, PaAP1_1	AT1G69120,AT1G26310,AT5G60910
PAXXG045840	scaffold18	252429	294733	+	Chr17	AP1-like, PaAP1_2	AT1G69120,AT1G26310,AT5G60910
PAXXG116820	scaffold73	755655	784975	+	Chr05	AP1-like, PaAP1_3	N/A
PAXXG349010	scaffold1125	123346	130996	+	Chr02	AP3-like, PaAP3_1	AT3G54340
PAXXG093380	scaffold50	66579	70348	+	Chr03	AP3-like, PaAP3_2	N/A
PAXXG113000	scaffold69	1103962	1118809	-	Chr19	AP3-like, PaAP3_3	AT3G54340
PAXXG070630	scaffold32	1199993	1213235	-	Chr01	AP3-like, PaAP3_4	AT3G54340
PAXXG008810	scaffold3	316922	321350	+	Chr08	BSISTER-like, PaBs1	AT5G23260
PAXXG063360	scaffold27	2429065	2433039	-	Chr08	OsMADS32-like, PaCFO1_1	N/A
PAXXG335970	scaffold896	124533	125209	-	Chr12	AGL62-like, PaAGL12	N/A
PAXXG185550	scaffold172	396110	406746	+	Chr08	AGL65-like, PaAGL13	AT2G03060,AT1G18750
PAXXG123370	scaffold80	1340350	1378833	+	Chr01	AGL65-like, PaAGL14	AT2G03060,AT1G18750
PAXXG016180	scaffold4	6180591	6184434	+	Chr15	AGL66-like, PaAGL15	AT1G77950,AT1G77980,AT1G22130
PAXXG220830	scaffold255	339615	340168	+	Chr01	AGL66-like, PaAGL16	N/A
PAXXG193450	scaffold188	666003	671529	+	Chr13	PI-like, PaPI1	AT5G20240
PAXXG241380	scaffold315	138644	216247	+	Chr06	SEP-like, PaSEP1	AT1G24260
PAXXG323200	scaffold755	236916	300259	-	unmapped	SEP-like, PaSEP2	AT1G24260
PAXXG116810	scaffold73	709533	739471	+	Chr05	SEP-like, PaSEP3	N/A
PAXXG080050	scaffold38	1331452	1363405	+	Chr06	SEP-like, PaSEP4	AT2G03710
PAXXG236780	scaffold300	730392	737115	+	Chr14	SEP-like, PaSEP5	N/A
PAXXG328320	scaffold805	4500	5033	-	unmapped	SEP-like, PaSEP6	N/A
PAXXG057830	scaffold24	1743611	1782090	+	Chr13	SOC1-like, PaSOC1_1	AT4G22950,AT5G62165,AT2G45660,<br>AT4G11880
PAXXG065170	scaffold28	2778775	2818907	+	Chr01	SOC1-like, PaSOC1_2	AT4G22950,AT5G62165,AT2G45660,<br>AT4G11880
PAXXG194760	scaffold191	207401	235611	+	Chr08	SVP-like, PaSVP1	AT2G22540
PAXXG314420	scaffold680	197345	201793	-	unmapped	SVP-like, PaSVP2	N/A
PAXXG093020	scaffold49	2061380	2064116	+	Chr15	M-alpha type, PaMalpha1	AT2G24840,AT5G60440,AT4G36590,<br>AT3G66656,AT2G34440
PAXXG101820	scaffold58	888784	890535	-	Chr16	M-alpha type, PaMalpha2	AT2G24840,AT5G60440,AT4G36590,<br>AT3G66656,AT2G34440
PAXXG051820	scaffold20	3273883	3275171	+	Chr07	M-alpha type, PaMalpha3	N/A
PAXXG152520	scaffold117	538018	538611	-	Chr10	M-alpha type, PaMalpha4	N/A
PAXXG152330	scaffold117	108741	113022	+	Chr10	M-alpha type, PaMalpha5	N/A
PAXXG187720	scaffold176	599658	600064	+	Chr07	M-alpha type, PaMalpha6	N/A
PAXXG335930	scaffold896	82268	82714	-	Chr12	M-alpha type, PaMalpha7	N/A
PAXXG335935	scaffold896	103637	104083	-	Chr12	M-alpha type, PaMalpha8	N/A
PAXXG335905	scaffold896	40643	41181	-	Chr12	M-alpha type, PaMalpha9	N/A
PAXXG289610	scaffold515	410038	410691	+	unmapped	M-alpha type, PaMalpha10	N/A
PAXXG289580	scaffold515	215687	216433	-	unmapped	M-gamma type, PaMgamma1	AT5G48670
PAXXG113810	scaffold70	428117	428925	-	Chr09	M-gamma type, PaMgamma2	AT5G48670
PAXXG201910	scaffold204	1021991	1023722	+	unmapped	M-gamma type, PaMgamma3	AT5G48670
PAXXG026260	scaffold9	1227897	1228425	-	Chr13	M-gamma type, PaMgamma4	N/A
PAXXG152470	scaffold117	414456	416157	-	Chr10	M-beta type, PaMbeta1	N/A
PAXXG374144	scaffold2758	8690	8995	+	unmapped	M-beta type, PaMbeta2	N/A
PAXXG126305	scaffold83	1780700	1780990	-	Chr16	M-beta type, PaMbeta3	N/A
PAXXG351395	scaffold1178	140360	141227	+	unmapped	M-beta type, PaMbeta4	N/A

TCP family

Gene ID	Scaffold ID	Start	End	strand	Chromosome	Description	Arabidopsis ortholog
PAXXG002620	scaffold1	5670561	5671546	-	Chr02	TCP transcription factor family, PCF subfamily	AT5G51910,AT2G45680
PAXXG136950	scaffold96	519085	520346	+	Chr03	TCP transcription factor family, PCF subfamily	AT5G51910,AT2G45680
PAXXG351590	scaffold1183	107093	108878	+	Chr16	TCP transcription factor family, PCF subfamily	AT3G27010
PAXXG312310	scaffold660	12718	14606	+	unmapped	TCP transcription factor family, PCF subfamily	AT3G27010
PAXXG157820	scaffold124	553171	555052	+	unmapped	TCP transcription factor family, PCF subfamily	AT1G58100,AT1G72010,AT1G35560
PAXXG030160	scaffold10	3762063	3762951	+	Chr14	TCP transcription factor family, PCF subfamily	AT1G69690,AT3G47620
PAXXG123030	scaffold80	730254	731524	-	Chr01	TCP transcription factor family, PCF subfamily	AT1G69690,AT3G47620
PAXXG059160	scaffold25	636201	636893	+	Chr04	TCP transcription factor family, PCF subfamily	AT5G23280
PAXXG058310	scaffold24	2661078	2662667	-	Chr13	TCP transcription factor family, PCF subfamily	AT5G23280
PAXXG189640	scaffold180	544491	545405	+	Chr15	TCP transcription factor family, PCF subfamily	N/A
PAXXG174050	scaffold150	679663	680735	-	Chr09	TCP transcription factor family, PCF subfamily	AT2G37000
PAXXG010770	scaffold3	3456063	3456929	+	Chr08	TCP transcription factor family, CYC subfamily	AT3G18550
PAXXG092900	scaffold49	1714572	1715576	-	Chr15	TCP transcription factor family, CYC subfamily	AT1G68800
PAXXG092910	scaffold49	1723978	1725009	-	Chr15	TCP transcription factor family, CYC subfamily	N/A
PAXXG056230	scaffold23	1847819	1872300	-	Chr01	TCP transcription factor family, CIN subfamily	AT4G18390
PAXXG110140	scaffold67	252263	278453	+	Chr14*	TCP transcription factor family, CIN subfamily	AT4G18390
PAXXG121830	scaffold79	118949	121462	+	Chr07*	TCP transcription factor family, CIN subfamily	AT3G15030,AT1G53230

PAXXG243080	scaffold320	564104	566037	+	Chr12	TCP transcription factor family, CIN subfamily	AT3G15030,AT1G53230
PAXXG051330	scaffold20	2472173	2474790	+	Chr07	TCP transcription factor family, CIN subfamily	AT3G02150
PAXXG297730	scaffold564	279341	280383	+	Chr06	TCP transcription factor family, CIN subfamily	N/A
PAXXG221270	scaffold256	22605	39771	+	unmapped	TCP transcription factor family, CIN subfamily	N/A
PAXXG074530	scaffold35	501086	503780	-	Chr11	TCP transcription factor family, CIN subfamily	N/A
PAXXG116740	scaffold73	587941	623079	+	Chr05	TCP transcription factor family, CIN subfamily	N/A

Table S21 Differential expression analysis with DESeq2 for comparison between the tissues in *P. aphrodite*

Gene	*P. aphrodite* ID	LFC[†] for OF vs LB	padj[‡] for OF vs LB	LFC for OF vs SB	padj for OF vs SB	LFC for OF vs Leaf	padj for OF vs Leaf	LFC for OF vs Root	padj for OF vs Root	LFC for LB vs SB	padj for LB vs SB	LFC for LB vs Leaf	padj for LB vs Leaf	LFC for LB vs Root	padj for LB vs Root	LFC for SB vs Leaf	padj for SB vs Leaf	LFC for SB vs Root	padj for SB vs Root	LFC for Leaf vs Root	padj for Leaf vs Root
PAL	PAXXG114440	-2.26	0	-0.89	0.15	0.46	0.48	-2.62	1.10E-06	1.37	0.02	2.72	2.04E-07	-0.36	0.64	1.35	0.01	-1.73	0	-3.08	4.20E-09
PAL	PAXXG094080	1.82	0.21	1.05	0.38	0.22	0.86	-1.43	0.23	-0.76	0.59	-1.59	0.12	-3.25	0	-0.83	0.44	-2.48	0.02	-1.65	0.12
PAL	PAXXG262090	-0.31	0.97	0.19	0.95	-3.07	0.05	-1.6	0.42	0.5	0.86	-2.76	0.07	-1.29	0.52	-3.26	0.03	-1.79	0.36	1.47	0.38
C4H	PAXXG031230	1.72	2.23E-06	3.85	8.59E-34	6.92	3.97E-104	1.59	2.71E-06	2.13	1.21E-10	5.21	6.49E-59	-0.13	0.81	3.08	5.18E-21	-2.26	4.87E-12	-5.34	1.06E-61
C4H	PAXXG108080	-1.36	0.01	-1.33	0	2.83	1.11E-10	-2.51	1.27E-08	0.03	0.98	4.19	1.23E-22	-1.15	0.02	4.16	1.99E-22	-1.18	0.02	-5.34	3.07E-36
4CL	PAXXG000580	0.36	0.94	-1.22	0.42	-1.47	0.28	-2.96	0.02	-1.58	0.31	-1.83	0.16	-3.32	0.01	-0.25	0.87	-1.74	0.21	-1.5	0.25
4CL	PAXXG156720	-0.91	0.46	-2.33	0	-0.48	0.56	-2.66	8.66E-05	-1.42	0.06	0.43	0.58	-1.75	0.02	1.86	0.01	-0.33	0.74	-2.18	0
4CL	PAXXG020250	-1.14	0.61	1.41	0.32	0.68	0.63	-4.95	1.40E-06	2.54	0.05	1.82	0.12	-3.81	0	-0.72	0.6	-6.36	4.31E-09	-5.63	6.45E-08
4CL	PAXXG333030	2.68	4.87E-10	1.18	0.01	1.91	3.25E-06	-2.95	1.02E-13	-1.5	0	-0.77	0.08	-5.64	2.16E-47	0.73	0.1	-4.14	2.45E-26	-4.87	7.54E-36
4CL	PAXXG333050	2.21	0.01	-0.89	0.26	-2.11	0	-5.93	2.23E-21	-3.1	1.36E-05	-4.32	1.17E-10	-8.14	1.14E-35	-1.22	0.08	-5.04	6.15E-16	-3.82	1.34E-09
4CL	PAXXG001940	0.95	0.14	1.5	0	0.48	0.36	0.54	0.36	0.55	0.36	-0.47	0.35	-0.42	0.49	-1.02	0.03	-0.97	0.06	0.06	0.94
4CL	PAXXG137490	-1.55	NA	-1.52	NA	-2.4	0.51	-1.5	0.75	0.03	1	-0.85	0.82	0.05	0.99	-0.88	0.81	0.02	1	0.89	0.83
4CL	PAXXG137510	-1.58	NA	-2.96	NA	3.32	0.23	1.09	0.74	-1.38	0.6	4.89	0.05	2.66	0.31	6.28	0.01	4.05	0.09	-2.23	0.48
4CL	PAXXG137520	-1.39	NA	0.66	NA	4.31	0.16	0.52	0.9	2.04	0.55	5.7	0.05	1.9	0.56	3.65	0.23	-0.14	0.97	-3.8	0.24
4CL	PAXXG124540	1.91	0	1.19	0.03	-1.31	0.01	0.7	0.26	-0.72	0.25	-3.23	1.08E-11	-1.21	0.03	-2.5	1.88E-07	-0.49	0.46	2.01	5.00E-05
CHS	PAXXG122400	0.72	0.81	-0.08	0.97	0.36	0.79	1.8	0.15	-0.81	0.6	-0.36	0.78	1.08	0.44	0.44	0.73	1.89	0.12	1.44	0.23
CHS	PAXXG122380	0.1	0.98	3.15	9.16E-06	-0.27	0.76	5.41	3.39E-11	3.05	2.69E-05	-0.37	0.65	5.3	7.79E-11	-3.42	7.72E-07	2.25	0.02	5.68	2.14E-12
CHS	PAXXG122420	0.01	1	0.58	0.87	-1.14	0.68	6.71	0.03	0.57	0.89	-1.15	0.67	6.7	0.02	-1.72	0.5	6.14	0.04	7.85	0
CHS	PAXXG173690																				
CHS	PAXXG174720	-1.19	0.02	-1.62	0	9.17	2.62E-80	1.17	0.01	-0.43	0.46	10.36	9.86E-103	2.37	8.75E-09	10.79	1.95E-111	2.8	5.14E-12	-8	5.54E-61
CHI	PAXXG290740	-1.25	0.02	-2.42	1.40E-08	2.42	1.78E-08	0.55	0.33	-1.17	0.01	3.67	1.45E-18	1.79	5.06E-05	4.84	8.53E-32	2.96	1.77E-12	-1.88	2.10E-05
CHI	PAXXG101960	-1.22	0.38	-4.33	2.72E-08	-1.75	0.04	-4.45	1.20E-08	-3.11	0	-0.53	0.57	-3.23	5.04E-05	2.58	0	-0.12	0.92	-2.7	0
F3H	PAXXG153170	1.04	0.4	2.77	0	10.23	4.50E-16	3.96	1.54E-08	1.73	0.03	9.19	2.93E-13	2.92	5.59E-05	7.46	4.59E-09	1.19	0.16	-6.27	1.65E-06
FLS	PAXXG015280	-1.06	0.78	1.82	0.29	-0.34	0.86	-0.35	0.88	2.88	0.08	0.72	0.68	0.71	0.74	-2.16	0.16	-2.17	0.21	-0.01	1
F3'H	PAXXG116560	0.57	0.83	-1.43	0.15	-1.46	0.12	-0.89	0.45	-2	0.04	-2.04	0.02	-1.46	0.17	-0.03	0.98	0.55	0.66	0.58	0.6
F3'H	PAXXG116530	-1.57	0.27	-2.35	0.01	-0.51	0.66	-2.02	0.04	-0.77	0.53	1.07	0.28	-0.44	0.73	1.84	0.04	0.33	0.8	-1.51	0.12
F3'H	PAXXG116590																				
F3'H	PAXXG079820	-0.22	0.81	0.19	0.67	-1.82	2.60E-09	0.18	0.71	0.41	0.33	-1.6	1.44E-07	0.4	0.33	-2.01	2.82E-11	-0.01	0.99	2	4.95E-11
F3'H	PAXXG172030	-0.13	0.99	-3.75	0	-0.72	0.59	-2.02	0.1	-3.62	0	-0.59	0.65	-1.89	0.13	3.03	0	1.72	0.16	-1.31	0.29
F3'5'H	PAXXG083160	-1.23	0.8	-3.74	0.05	-1.69	0.4	-2.61	0.22	-2.51	0.25	-0.46	0.83	-1.38	0.56	2.05	0.27	1.13	0.64	-0.92	0.69
F3'5'H	PAXXG083130	-0.96	NA	2.47	NA	2.33	0.74	-3.63	0.63	3.42	0.68	3.29	0.61	-2.67	0.73	-0.13	0.99	-6.09	0.37	-5.96	0.34
F3'5'H	PAXXG083150	1.1	0.65	0.13	0.95	-0.43	0.76	-2.9	0.01	-0.97	0.53	-1.54	0.19	-4.01	0	-0.56	0.66	-3.03	0.01	-2.47	0.03
F3'5'H	PAXXG087010	0.44	0.85	-2.5	0	4.68	1.28E-05	-1.07	0.24	-2.94	0	4.24	7.53E-05	-1.51	0.08	7.18	2.34E-12	1.43	0.09	-5.75	4.64E-08
F3'5'H	PAXXG261960	0.34	0.84	5.99	6.15E-32	1.71	0	4.03	6.39E-15	5.65	2.74E-28	1.37	0.01	3.69	1.06E-12	-4.28	8.12E-17	-1.96	0	2.32	1.51E-05
DFR	PAXXG195470	-2.88	0.26	-6.26	0	-4.17	0.01	-4.79	0.01	-3.38	0.04	-1.29	0.45	-1.91	0.3	2.09	0.18	1.47	0.44	-0.62	0.76
ANS/LDOX	PAXXG070550	-2.22	0.6	-6.33	0	-3.34	0.12	-4.01	0.08	-4.1	0.04	-1.12	0.61	-1.79	0.46	2.99	0.11	2.32	0.29	-0.67	0.79
ANS/LDOX	PAXXG222000	0.01	1	0.01	1	-0.13	0.99	-9.39	0.08	0	1	-0.14	0.99	-9.4	0.08	-0.13	0.99	-9.4	0.07	-9.26	0.06
3GT	PAXXG240040	0.68	0.37	-0.29	0.62	0.82	0.08	-0.24	0.71	-0.97	0.05	0.15	0.79	-0.92	0.07	1.12	0.01	0.05	0.94	-1.06	0.02
3GT	PAXXG223560	0.75	0.23	-0.24	0.66	-0.3	0.53	-1.01	0.02	-0.99	0.03	-1.05	0.01	-1.76	1.09E-05	-0.06	0.91	-0.77	0.09	-0.71	0.1
3GT	PAXXG068210	1.12	0.75	0.1	0.97	6.88	0	4.64	0.02	-1.02	0.63	5.76	0.01	3.52	0.09	6.78	0	4.54	0.02	-2.24	0.43

[†] log2 fold change

[‡] BH adjusted p-values for Wald test

Table S22 Differential expression analysis with DESeq2 for comparison between the tissues in *P. lueddemanniana*

Gene	*P. lueddemanniana* ID	LFC[†] for OF vs LB	padj[‡] for OF vs LB	LFC for OF vs SB	padj for OF vs SB	LFC for OF vs Leaf	padj for OF vs Leaf	LFC for OF vs Root	padj for OF vs Root	LFC for LB vs SB	padj for LB vs SB	LFC for LB vs Leaf	padj for LB vs Leaf	LFC for LB vs Root	padj for LB vs Root	LFC for SB vs Leaf	padj for SB vs Leaf	LFC for SB vs Root	padj for SB vs Root	LFC for Leaf vs Root	padj for Leaf vs Root
PAL	PLTC041547	-0.6	0.66	-0.58	0.62	1.02	0.35	-0.76	0.51	0.02	0.99	1.63	0.11	-0.16	0.91	1.61	0.12	-0.18	0.9	-1.78	0.11
PAL	PLTC034219	3.44	0.01	1.46	0.29	1.92	0.15	-0.19	0.91	-1.98	0.16	-1.52	0.26	-3.63	0	0.46	0.75	-1.65	0.26	-2.11	0.14
C4H	PLTC004513	0.74	0.13	2.45	7.66E-10	2.32	8.48E-09	1.79	1.22E-05	1.71	4.02E-05	1.57	0	1.04	0.02	-0.13	0.78	-0.66	0.16	-0.53	0.28
C4H	PLTC046538	-2.01	0.04	-6	2.38E-13	-1.99	0.02	-6.29	1.74E-14	-3.99	2.36E-06	0.01	0.99	-4.28	3.31E-07	4.01	1.31E-06	-0.29	0.8	-4.3	3.30E-07
4CL	PLTC047369	-3.37	0.01	-4.9	2.92E-05	-2.98	0.02	-3.14	0.01	-1.53	0.26	0.39	0.78	0.23	0.88	1.92	0.12	1.76	0.19	-0.16	0.93
4CL	PLTC042481	-3.5	2.66E-08	-4.62	4.25E-15	-3.89	6.87E-11	-4.84	1.99E-16	-1.11	0.1	-0.39	0.57	-1.34	0.04	0.73	0.26	-0.23	0.78	-0.95	0.17
4CL	PLTC004626	-1.03	0.82	18.1	4.66E-09	-4.39	0.16	-9.7	0	19.13	6.36E-10	-3.37	0.28	-8.67	0	-22.49	6.69E-14	-27.8	2.23E-20	-5.3	0.1
4CL	PLTC043232	-0.36	0.65	-2.07	0	-1.7	0	-6.84	1.71E-38	-1.72	0	-1.34	0.02	-6.48	9.77E-35	0.37	0.55	-4.77	3.39E-19	-5.14	3.81E-22
4CL	PLTC008887	-1.03	0.27	-2.4	0	-2.52	0	-7.31	1.99E-25	-1.37	0.09	-1.49	0.05	-6.28	3.67E-19	-0.12	0.9	-4.91	5.24E-12	-4.79	1.65E-11
4CL	PLTC005048	-0.3	0.57	0.38	0.38	-1.29	0	-0.53	0.21	0.68	0.11	-0.99	0.01	-0.23	0.63	-1.67	7.03E-06	-0.91	0.03	0.76	0.07
4CL	PLTC037368																				
4CL	PLTC005311	0.66	0.55	1.71	0.04	1.78	0.04	3.54	1.22E-05	1.05	0.27	1.12	0.19	2.88	0	0.07	0.94	1.83	0.04	1.76	0.05
CHS	PLTC008603	-1.51	NA	0.28	NA	-2.13	NA	-6.29	NA	1.79	NA	-0.62	NA	-4.78	NA	-2.41	NA	-6.57	NA	-4.16	NA
CHS	PLTC045217	-0.09	NA	3.39	NA	0.89	NA	-4.45	NA	3.48	NA	0.99	NA	-4.36	NA	-2.5	NA	-7.84	NA	-5.34	NA
CHS	PLTC003489	-4.36	4.10E-05	-3.99	6.20E-05	-2.09	0.05	-4.69	2.37E-06	0.37	0.79	2.27	0.03	-0.33	0.81	1.9	0.07	-0.7	0.58	-2.6	0.02
CHI	PLTC038317	-5.12	1.49E-08	-6.65	4.25E-15	-4.89	1.64E-08	-7.27	9.12E-18	-1.52	0.1	0.23	0.82	-2.14	0.02	1.76	0.04	-0.62	0.56	-2.38	0.01
CHI	PLTC034630	-2.08	0.01	-3.26	6.76E-06	-1.79	0.02	-1.75	0.02	-1.18	0.16	0.28	0.74	0.33	0.73	1.46	0.05	1.51	0.06	0.04	0.97
F3H	PLTC046352	-8.04	1.33E-52	-9.01	1.71E-67	2.51	0.01	2.25	0.01	-0.97	0.05	10.54	3.56E-36	10.29	2.15E-42	11.52	5.64E-43	11.26	1.36E-50	-0.25	0.88
FLS	PLTC046091	-1.7	4.77E-05	0.66	0.12	9.37	5.47E-13	1.91	2.40E-06	2.36	1.29E-09	11.07	6.07E-18	3.61	1.47E-20	8.71	1.84E-11	1.25	0	-7.46	2.14E-08
F3'H	PLTC007400																				
F3'H	PLTC042293	0.52	NA	-0.86	NA	-0.73	NA	-3.68	NA	-1.38	NA	-1.25	NA	-4.2	NA	0.13	NA	-2.82	NA	-2.95	NA
F3'H	PLTC002884	-8.41	2.82E-25	-8.73	2.26E-28	-0.07	0.95	-9.45	4.43E-33	-0.32	0.75	8.34	2.75E-26	-1.04	0.22	8.66	3.49E-28	-0.73	0.42	-9.39	1.73E-32
F3'5'H	PLTC047335	-1.73	0.43	-6.13	0	7.37	0	4.97	0.01	-4.4	0.01	9.1	1.15E-05	6.7	0	13.5	3.12E-11	11.1	2.51E-09	-2.4	0.39
F3'5'H	PLTC002683	-1.66	4.92E-05	-0.99	0.01	-0.78	0.06	-0.88	0.03	0.67	0.11	0.88	0.02	0.77	0.06	0.21	0.63	0.11	0.84	-0.1	0.85
F3'5'H	PLTC045391	1.17	0.33	10.7	4.71E-32	13.33	9.17E-43	13.93	3.67E-44	9.53	2.73E-25	12.16	9.84E-36	12.76	4.96E-37	2.63	0.01	3.23	0	0.6	0.7
DFR	PLTC042396	-7.55	0	-8.27	2.71E-05	4.64	0.06	-1.11	0.65	-0.72	0.79	12.19	7.96E-08	6.43	0	12.91	1.12E-08	7.16	0	-5.75	0.02
ANS/LDOX	PLTC043440	0.4	0.19	0.15	0.61	1.75	3.08E-13	1.54	2.20E-10	-0.25	0.41	1.36	2.23E-08	1.14	4.93E-06	1.6	2.44E-11	1.39	1.96E-08	-0.22	0.49
ANS/LDOX	PLTC045691																				
3GT	PLTC002052	-0.75	0.39	-1.39	0.05	-0.62	0.42	-2.47	0	-0.63	0.44	0.13	0.87	-1.71	0.02	0.77	0.29	-1.08	0.16	-1.85	0.01
3GT	PLTC038719	-0.31	0.28	-1.35	8.90E-10	-1.88	6.75E-18	-1.34	1.87E-09	-1.04	4.47E-06	-1.57	6.14E-13	-1.02	6.41E-06	-0.52	0.02	0.02	0.96	0.54	0.02
3GT	PLTC037014	-0.31	0.72	-0.31	0.66	3.83	2.67E-11	2.12	0	0	1	4.14	3.79E-13	2.42	4.18E-05	4.14	3.57E-13	2.42	5.05E-05	-1.71	0.01

† log2 fold change

‡ BH adjusted p-values for Wald test

Table S23 Using inkage group-specific clones to identify the misassembled scaffolds in *P. equestris* draft genome (Cai et al., 2015; Zhang et al., 2017)

P. equestris scaffold ID	Linkage group	Marker name	Clone name	Size (nt)	Alignment length (nt)	Alignment start position in *P. equestris* scaffold	Alignment end position in *P. equestris* scaffold	Identity (%)
fragScaff_scaffold_1009	L01	DL14-S43	DL14-S43-1	2706	2723	18090751	18094387	93.50
fragScaff_scaffold_1009	L01	DL14-S43	DL14-S43-2	2199	2267	18097274	18099496	94.67
fragScaff_scaffold_1009	L01	DL14-S43	DL14-S43-3	3667	1527	18100062	18103206	94.24
fragScaff_scaffold_1009	L01	DL14-S43	DL14-S43-4	2756	2426	18103462	18106001	93.69
fragScaff_scaffold_1009	L03	DL05-S240	DL05-S240-1	2459	2477	35128117	35130577	94.67
fragScaff_scaffold_1009	L03	DL05-S240	DL05-S240-2	4048	3338	35131000	35134311	93.62
fragScaff_scaffold_1009	L03	DL05-S240	DL05-S240-3	4995	5048	35135385	35145521	90.59
fragScaff_scaffold_1009	L19	DL16-S20	DL16-S20-3	3258	3299	47611520	47614790	94.97
fragScaff_scaffold_1009	L19	DL16-S20	DL16-S20-2	3316	3287	47614994	47618606	96.74
fragScaff_scaffold_1009	L19	DL16-S20	DL16-S20-1	3412	3415	47618644	47622054	98.68
fragScaff_scaffold_1009	L02	DL08-S19	DL08-S19-3	3845	3855	49149187	49153031	96.39
fragScaff_scaffold_1009	L02	DL08-S19	DL08-S19-2	4079	4086	49153306	49157382	96.82
fragScaff_scaffold_1009	L02	DL08-S19	DL08-S19-1	2533	2556	49164447	49167050	94.41
fragScaff_scaffold_1009	L03	DL05-S347	DL05-S347-1	3145	3159	66189686	66192875	95.06
fragScaff_scaffold_1009	L03	DL05-S347	DL05-S347-2	3932	3973	66193251	66197197	94.41
fragScaff_scaffold_1009	L03	DL05-S347	DL05-S347-3	3963	3979	66202599	66206522	94.67
fragScaff_scaffold_1009	L16	DL01-S13	DL01-S13-3	3131	3295	66950058	66953417	93.48
fragScaff_scaffold_1009	L16	DL01-S13	DL01-S13-2	3130	3151	66953446	66956583	96.41
fragScaff_scaffold_1009	L16	DL01-S13	DL01-S13-1	3337	3361	66963227	66966576	96.19
fragScaff_scaffold_1360	L14	DL18-S0	DL18-S0-3	3620	2733	305762	309063	90.16
fragScaff_scaffold_1360	L14	DL18-S0	DL18-S0-2	3013	3023	309801	317777	95.96
fragScaff_scaffold_1360	L14	DL18-S0	DL18-S0-1	3818	3888	318433	322490	95.09
fragScaff_scaffold_1360	L13	DL02-S169	DL02-S169-1	3397	2902	5623091	5625993	91.18
fragScaff_scaffold_1360	L13	DL02-S169	DL02-S169-2	1531	1529	5626099	5627611	95.03
fragScaff_scaffold_1360	L13	DL02-S169	DL02-S169-3	3178	3204	5627973	5631143	94.63
fragScaff_scaffold_1360	L13	DL02-S169	DL02-S169-4	1804	1831	5631162	5632987	95.25
original_scaffold_47438	L18	DL13-S13	DL13-S13-3	3400	3370	2907737	2911098	92.38
original_scaffold_47438	L18	DL13-S13	DL13-S13-2	3703	3719	2911201	2914908	93.90
original_scaffold_47438	L18	DL13-S13	DL13-S13-1	3338	3321	2922196	2925601	95.90
original_scaffold_47438	L07	DL10-S27	DL10-S27-3	3297	3329	5673256	5676582	96.43
original_scaffold_47438	L07	DL10-S27	DL10-S27-2	2292	2293	5676787	5679078	97.12
original_scaffold_47438	L07	DL10-S27	DL10-S27-1	4449	5483	5683642	5689113	95.51
fragScaff_scaffold_169	L01	DL14-S80	DL14-S80-1	3594	3599	534459	538033	97.33
fragScaff_scaffold_169	L01	DL14-S80	DL14-S80-2	3390	3396	538189	541573	97.64
fragScaff_scaffold_169	L01	DL14-S80	DL14-S80-3	2471	2472	542388	544854	97.53
fragScaff_scaffold_169	L11	DL06-S243	DL06-S243-3	3306	3256	2035413	2038844	92.11
fragScaff_scaffold_169	L11	DL06-S243	DL06-S243-2	3851	4029	2040931	2045092	91.61
fragScaff_scaffold_169	L11	DL06-S243	DL06-S243-1	3771	3656	2045491	2055573	94.20
fragScaff_scaffold_550	L12	Unmapped-S17-456	Unmapped-S17-4	3292	3255	592242	595494	93.79
fragScaff_scaffold_550	L12	Unmapped-S17-456	Unmapped-S17-5	3231	3188	596714	601241	93.32
fragScaff_scaffold_550	L12	Unmapped-S17-456	Unmapped-S17-6	3162	3264	603215	606537	93.10
fragScaff_scaffold_550	L05a	DL07-S1768	DL07-S1768-3	4376	3502	767686	771104	95.20
fragScaff_scaffold_550	L05a	DL07-S1768	DL07-S1768-1	3653	3615	778209	781978	94.33
fragScaff_scaffold_550	L05a	DL07-S84	DL07-S84-1	3085	3260	1164723	1168210	92.27
fragScaff_scaffold_550	L05a	DL07-S84	DL07-S84-2	4023	4180	1170552	1174954	92.32
fragScaff_scaffold_550	L05a	DL07-S84	DL07-S84-3	3129	3129	1182013	1185099	95.27
fragScaff_scaffold_550	L05b	DL07-S64	DL07-S64-1	3599	1661	4293206	4294860	95.30
fragScaff_scaffold_550	L05b	DL07-S64	DL07-S64-2	3662	3678	4295060	4298878	96.35
fragScaff_scaffold_550	L05b	DL07-S64	DL07-S64-3	3782	3371	4299272	4302714	97.06
fragScaff_scaffold_262	L04	DL12-S319	DL12-S319-3	2950	2962	358379	417975	95.54
fragScaff_scaffold_262	L04	DL12-S319	DL12-S319-2	3907	4005	360100	364053	97.26
fragScaff_scaffold_262	L04	DL12-S319	DL12-S319-1	3488	4217	364126	369897	93.43
fragScaff_scaffold_262	L16	DL01-S129	DL01-S129-4	3021	2778	906861	915641	95.79
fragScaff_scaffold_262	L16	DL01-S129	DL01-S129-3	2721	2724	920471	923188	94.31
fragScaff_scaffold_262	L16	DL01-S129	DL01-S129-2	3515	1685	925145	926815	92.82
fragScaff_scaffold_262	L16	DL01-S129	DL01-S129-1	1863	1877	927242	929096	94.41
fragScaff_scaffold_1558	L04	DL12-S18	DL12-S18-3	3128	3187	1255639	1263166	94.29
fragScaff_scaffold_1558	L04	DL12-S18	DL12-S18-2	3160	2583	1263659	1266900	90.86
fragScaff_scaffold_1558	L04	DL12-S18	DL12-S18-1	3650	3694	1266934	1270616	95.32
fragScaff_scaffold_1558	L08a	DL19-S345	DL19-S345-3	3230	3306	3622617	3628283	94.80
fragScaff_scaffold_1558	L08a	DL19-S345	DL19-S345-2	3550	3563	3630135	3633691	96.83
fragScaff_scaffold_1558	L08a	DL19-S345	DL19-S345-1	3817	3273	3642787	3646070	95.90
fragScaff_scaffold_747	L13	DL02-S46	DL02-S46-4	2821	2857	1595711	1598536	95.03
fragScaff_scaffold_747	L13	DL02-S46	DL02-S46-2	1860	1866	1599577	1601421	96.41
fragScaff_scaffold_747	L13	DL02-S46	DL02-S46-1	3413	3419	1601402	1604767	96.40
fragScaff_scaffold_747	L13	DL02-S46	DL02-S46-3	2528	2538	1604501	1607033	97.32
fragScaff_scaffold_747	L03	DL05-S874	DL05-S874-3	3992	4048	2683641	2687652	93.05
fragScaff_scaffold_747	L03	DL05-S874	DL05-S874-2	2979	2904	2687943	2690797	96.39
fragScaff_scaffold_747	L03	DL05-S874	DL05-S874-1	3851	4027	2691681	2695761	91.48
fragScaff_scaffold_945	L10a	DL04-S363	DL04-S363-3	3973	3984	939753	943792	91.69
fragScaff_scaffold_945	L10a	DL04-S363	DL04-S363-2	3797	3591	948788	955595	93.09
fragScaff_scaffold_945	L10a	DL04-S363	DL04-S363-1	4206	4122	956632	960687	90.15
fragScaff_scaffold_945	L07	DL10-S850	DL10-S850-2	3280	3356	2280871	2284179	94.16
fragScaff_scaffold_945	L07	DL10-S850	DL10-S850-1	3720	3886	2284341	2289638	93.67
fragScaff_scaffold_895	L06	DL15-S120	DL15-S120-1	3546	3587	583886	587467	95.23

fragScaff_scaffold_895	L06	DL15-S120	DL15-S120-2	4014	3919	589311	602518	93.16
fragScaff_scaffold_895	L06	DL15-S120	DL15-S120-3	2428	2569	603959	606520	92.91
fragScaff_scaffold_895	L04	DL12-S2	DL12-S2-1	3644	3676	2626769	2630398	93.09
fragScaff_scaffold_895	L04	DL12-S2	DL12-S2-2	3515	3519	2635024	2638519	96.48
fragScaff_scaffold_895	L04	DL12-S2	DL12-S2-3	3303	3242	2639142	2644962	95.71
